# Molecular Simulation of Hydrogen Systems: From Properties and Methods to Applications and Future Directions

**DOI:** 10.1021/acs.chemrev.5c00617

**Published:** 2025-12-10

**Authors:** Ahmadreza Rahbari, Thejas Hulikal Chakrapani, Fei Shuang, Panagiotis Krokidas, Parsa Habibi, V. Jelle Lagerweij, Mahinder Ramdin, Thijs J. H. Vlugt, Hadi Hajibeygi, Poulumi Dey, Ioannis N. Tsimpanogiannis, Othonas A. Moultos

**Affiliations:** † Headquarters, XINTC B.V., Loubergweg 22-24, 6961 EK Eerbeek, The Netherlands; ‡ Engineering Thermodynamics, Process & Energy Department, Faculty of Mechanical Engineering, 2860 Delft University of Technology, Leeghwaterstraat 39, 2628 CB Delft, The Netherlands; § 67387 Institute for Multiscale Thermofluids, School of Engineering, University of Edinburgh, Edinburgh EH9 3FB, United Kingdom; ∥ Department of Materials Science and Engineering, Faculty of Mechanical Engineering, Delft University of Technology, Mekelweg 2, 2628 CD Delft, The Netherlands; ⊥ National Centre for Scientific Research “Demokritos”, 15310 Aghia Paraskevi Attikis, Greece; # Geoscience and Engineering Department, Faculty of Civil Engineering and Geosciences, Delft University of Technology, Stevinweg 1, 2628 CN Delft, The Netherlands; ∇ 419215 Chemical Process & Energy Resources Institute (CPERI), Centre for Research & Technology Hellas (CERTH), 57001 Thermi-Thessaloniki, Greece

## Abstract

This extensive review highlights the central role of classical molecular simulation in advancing hydrogen (H_2_) technologies. As the transition to a sustainable energy landscape is urgently needed, the optimization of H_2_ processes, spanning production, purification, transportation, storage, safety, and utilization is essential. To this end, accurate prediction of thermodynamic, transport, structural, and interfacial properties is important for overcoming engineering challenges across the entire H_2_ value chain. Experimental measurements, despite being the traditional way of obtaining these properties, can be limited by the distinctive nature of H_2_, harsh operating conditions, safety constraints, and extensive parameter spaces. Free from such limitations, classical molecular simulations, in the general frameworks of Monte Carlo and Molecular Dynamics, provide an optimal balance between computational efficiency and accuracy, bridging the gap between quantum mechanical calculations and macro-scale modeling. This review also systematically covers molecular simulation methods and force fields for computing key properties of H_2_ systems, such as phase and adsorption equilibria and transport coefficients. Beyond property prediction, we explore how molecular simulation reveals fundamental mechanisms governing hydrate formation and dissociation, membrane permeations, and H_2_ embrittlement. When possible, data from multiple sources are compared and critically assessed, while effort is put on evaluating the force fields used and methodological approaches followed in the literature. Finally, this review aims at identifying research gaps and future opportunities, emphasizing emerging approaches, such as molecular simulation in the era of artificial intelligence.

## Graphical abstract

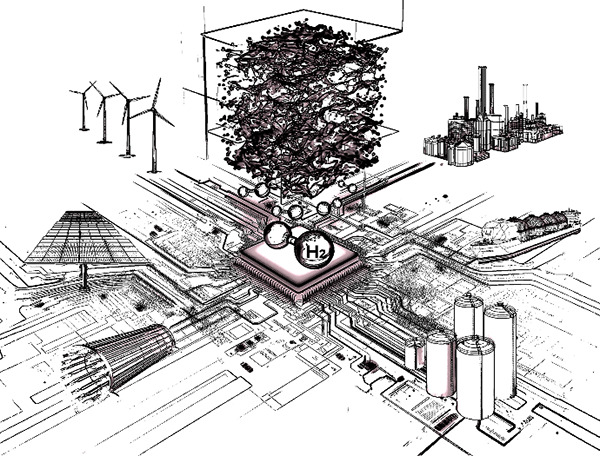

## 1 Introduction

### 1.1 Background and Motivation

Hydrogen (H_2_) is a fundamental commodity in the chemical industry, serving as a building block of numerous products and processes in many different sectors. Characteristic examples are ammonia production, methanol synthesis, hydrocracking, steel and cement production, space exploration, and aerospace propulsion systems.^1–9^ In the past decade, H_2_ has gained significant attention as a clean energy carrier because it can be stored on the TWh scale.^10^ H_2_ can be produced via various methods, such as thermochemical processes, water electrolysis, direct solar water splitting, and geological and biological pathways.^11,12^ Currently, the chemical industry primarily relies on fossil fuels to produce H_2_, mostly without treatment of the emitted CO_2_ (e.g., via natural hydrocarbon cracking, steam methane reforming, coal gasification or reforming processes).^1,13^ This is the so-called grey H_2_, which accounts for ca. 96% of the total H_2_ supply. If the CO_2_ emitted during H_2_ production from fossil fuels is captured and stored, the result is the so-called blue H_2_.^2^ The transition to a sustainable energy landscape, with H_2_ as an essential part, necessitates the efficient and economic production of the so-called green (i.e., via electrolysis of water using renewable energy sources) or low carbon H_2_, in sharp contrast to H_2_ produced from fossil sources.^14^

The European Union (EU) has reached the consensus that by 2030, 42% of the H_2_ utilized in the industrial sector must be derived from renewable non-biological sources. This figure should increase to 60% by 2035. This decision is in alignment with the EU initiative ”Fit for 55”, which aims at a 55% reduction in greenhouse gas emissions by 2030. Thus, efforts focusing on scaling up the production and use of H_2_ as a green energy carrier are becoming urgent in the context of chemical industry and mitigation of environmental impacts.^13,15–17^ Such efforts, however, are hindered by technological challenges such as the high cost of green H_2_ production,^16,18,19^ the inefficient separation/purification methods of H_2_ streams, the lack of safe transportation due to e.g., boil-off (the evaporation of liquid H_2_ due to heat leakage into cryogenic storage tanks, causing continuous product loss^20^) and H_2_ embrittlement,^17,21^ the lack of commodity high-energy-density storage systems,^22,23^ and the low efficiency, and often durability, of electrolyzers and fuel cells.^24^ These challenges span the whole H_2_ value chain, which in this review is going to be described by five different stages, i.e., H_2_ production, purification, transport, storage, and utilization as shown in Fig. 1. Although, this categorization is not unique, it offers a practical view of the predominant H_2_ technologies.

To overcome the engineering challenges in each stage of the H_2_ value chain and to devise optimized processes that will allow for H_2_ to play a central role in the energy mix of the future, accurate predictions of thermodynamic, transport, structural, mechanical, and electrochemical properties of H_2_ systems are essential.^19,22,25,26^ Such properties are phase equilibria and transport coefficients of gaseous mixtures and aqueous electrolyte solutions of H_2_ (crucial for water electrolysis, fuel cells, deoxygenation, temperature/pressure swing adsorption), adsorption and absorption energies of H_2_/adsorbents and storage capacities of different storage media (for physical storage), kinetic, structural and thermodynamic properties of hydrates (for physical storage and separations) and hydrides (chemical storage), electroosmotic drag coefficients in membranes (important for fuel cells and electrochemical compressors), interfacial tension and wettability (i.e., contact angles) of H_2_/solid/liquid/gas systems (for subsurface storage), permeabilities of H_2_ gas streams through nanoporous materials, membranes (separations of multicomponent H_2_ streams) and geological media (subsurface storage), and phenomena such as H_2_ embrittlement mechanisms in metals (safe transport in pipelines and storage in vessels). Aside from the plethora of required properties, the parameter space of temperatures, pressures, mixture compositions, and type of systems is extensive. This renders the total number of necessary physicochemical data extremely high, and therefore, makes data acquisition a challenging endeavor.

Traditionally, thermophysical properties are obtained via experimental measurements, often complemented by macroscale modeling. Nevertheless, due to the numerous different systems, the extremely broad state-point space, and the harsh conditions involved (e.g., high temperatures and/or pressures, corrosive and/or dangerous species) in H_2_ processes, experiments become costly, and often, not possible to perform at in-situ conditions due to the necessity for adherence to safety standards (e.g., Atmosphères Explosibles – ATEX) and Pressure Equipment Directive (PED) norms.^27,28^ As a consequence, thermophysical data of H_2_ systems remain scarce, especially at high pressures, temperatures, and electrolyte molalities (e.g., for alkaline/acidic electrolytes).^29,30^ The lack of such datasets is particularly important because due to the low volumetric energy density of H_2_ gas, high pressures (usually >200 bar) are necessary for many H_2_ applications. Another experimental shortcoming is the limitation in spatial resolutions of the available experimental techniques, which makes the detection of atomic hydrogen in alloys challenging.^31^ It is also important to note that experimental measurements alone, rarely guarantee deep insight into the fundamental physical and chemical mechanisms of the H_2_ systems. Understanding these mechanisms at the molecular scale is paramount for overcoming engineering hurdles, optimizing processes and materials, and coming up with novel technologies. Complementary to experimental measurements, equation of state (EoS) modeling is a popular and efficient approach, mainly for predicting the phase behavior of single- or multi-component H_2_ mixtures.^32–37^ However, cubic EoS encounter difficulty in accurately predicting the phase diagrams of H_2_ systems, while more advanced EoS such as the ones of SAFT, PC-SAFT, and Mie families, have been designed to mainly reproduce properties of long chain, polar, and hydrogen bonding molecules, and therefore, these models are not expected to have an improvement over simple cubic EoS.^38–45^ Moreover, EoS modeling alone cannot directly yield structural and transport properties of H_2_ systems.

Due to these inherent limitations of experiments and EoS modeling, molecular simulation, performed in the general frameworks of Monte Carlo (MC) and Molecular Dynamics (MD), has emerged as an alternative powerful tool for computing thermophysical properties of H_2_ systems at a wide parameter space range, and for providing unique insight into the molecular scale. Molecular simulations also guarantee safety and relatively low budget, since commodity computer clusters have become widely accessible. While performing molecular simulations to study H_2_ systems and processes is a potent research route, it is not without limitations.^46–53^ The first step in every molecular simulation is to choose the level of theory for simulating the material or system.^54^ Options span accurate quantum-mechanical potentials derived from wave function or density-functional theory (DFT), classical fully atomistic potentials fitted to DFT or experimental data, united atom two-body potentials, and coarse-grained models.^51,55–58^ The more detailed the model, the higher the accuracy, but the lower the computational efficiency. This means that although performing quantum mechanical simulations (e.g., DFT, Ab initio MD) will always yield the most precise results, such a modeling approach is only limited to small system sizes and time-scales. On the other side of the spectrum, although coarse-grained simulations (e.g., simulations using the Martini^59,60^ potentials or dissipative particle dynamics - DPD^61^) allow access to much higher length- and time-scales, the computed properties can only serve as a qualitative measure, since the atomistic detail is omitted. Combining the best of both worlds, classical molecular simulation offers distinct advantages such as relatively high computational efficiency and accurate prediction of thermophysical properties. In this sense, classical simulations play an important role in filling the gap between computationally intensive ab initio simulations and not-as-precise coarse-grained models or EoS. For these reasons, an extensive, but in some cases scattered, literature body of atomistic molecular simulations of H_2_ systems is available to date. In this review, we attempt to summarize these studies, and highlight the crucial role of molecular simulations, alongside experimental measurements, macro-scale modeling, and engineering approaches, in designing and refining H_2_ technologies. Lately, tremendous efforts by researchers in academia and industry is put in blending Machine Learning (ML) (or even broader, Artificial Intelligence - AI) with molecular simulation techniques towards improving the predictive ability of models, and accelerate simulations and/or material screening. This upcoming paradigm shift in the way we perform molecular simulations, is also discussed in this review, focusing on the case of H_2_ systems.

**Figure 1:**
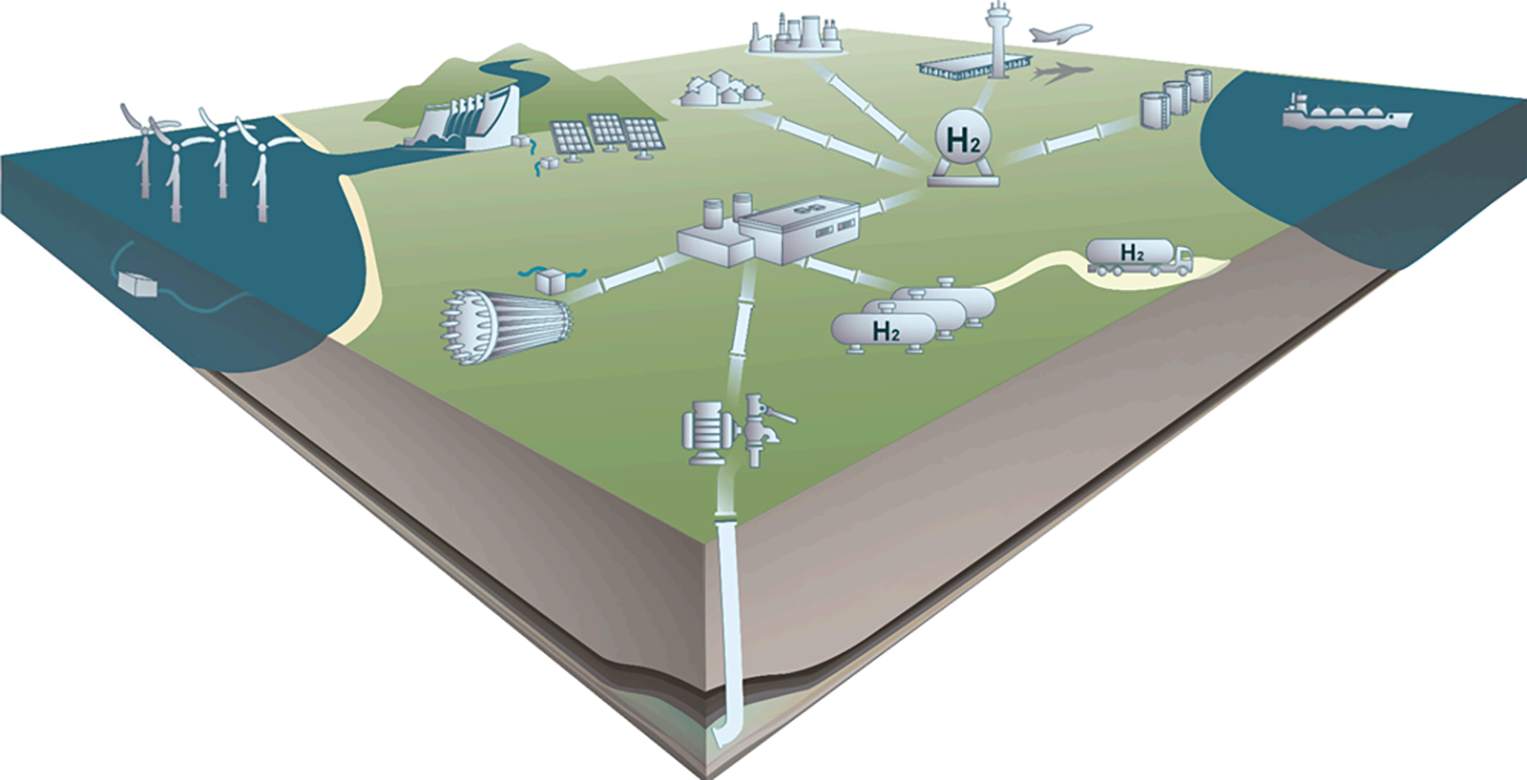
Schematic representation of a green hydrogen value chain spanning the production via water electrolysis using renewable energy sources, purification and compression of the hydrogen stream, storage in the liquid and gaseous form in tanks and in the subsurface, transportation via pipelines, trucks, and ships, and domestic and industrial utilization. Classical molecular simulation plays an important role as a reliable predictive tool for thermo-physical properties necessary for the design and optimization of the processes involved in all the stages in the hydrogen value chain.

### 1.2 What to Expect from This Review and How to Read It

The scope of this review is to showcase the importance of classical molecular simulation studies in advancing technologies spanning the whole H_2_ value chain (Fig. 1). The focus is on covering the most relevant properties of H_2_ systems that can be computed with molecular simulation throughout the different aspects of the H_2_ economy, i.e., we focus on properties important to H_2_ production via water electrolysis, storage (e.g., in porous media, in hydrates, in the subsurface), H_2_ compression for storage and mobility, membrane- and hydrate-based separations of H_2_ streams, and H_2_ embrittlement of metal pipelines. We provide a comparative assessment of the results of various properties of H_2_ systems, obtained from different sources, while stretching the limitations and possible misalignment in the available literature.

An overview of the available simulation methods, and a critical discussion on most important concepts for computing H_2_ properties is also provided. In more detail, we review different simulation techniques such as MC sampling methods for phase and adsorption equilibria, ways to compute hydrate three-phase (thermodynamic) equilibria, transport coefficients such as diffusivities, viscosities, and conductivities from MD simulations, kinetic MC simulations to obtain diffusion coefficients of H_2_ in materials, and high-throughput screening of materials using classical simulation combined with ML. Another important contribution of this review is the comprehensive analysis of the advantages and limitations of the available force fields for different species involved in H_2_ technologies (e.g., H_2_, aqueous electrolytes, substrate materials, organic components).

Based on the thorough discussion of the different H_2_ systems and properties, we also present an outlook for future research directions, especially in light of emerging approaches involving AI. When possible, we provide datasets of available properties of H_2_ systems in tables and figures. These datasets can serve both as a reference point and as a motivation for future research. The raw data along with tables summarizing the availability of different properties (and corresponding ranges) can also serve as a bridge between industrial applications and academic research, e.g., to aid constructing engineering correlations, and as feed to process design tools.

In a nutshell, the goals of this review are the following:

- Inform the reader regarding the capabilities of molecular simulation for computing thermophysical properties central to the H_2_ value chain.
- Provide an overview of the available literature and point the reader towards the appropriate molecular simulation studies relevant to various technologies across H_2_ economy.
- Introduce the main molecular simulation methods and associated techniques for computing thermodynamic, transport, and interfacial properties of H_2_ systems.
- Engage into a comprehensive discussion on the fundamental and engineering insights provided from molecular simulation of H_2_ systems.
- Contribute tables and figures containing available thermophysical data compiled from different sources.
- Bring forth a pragmatic and useful outlook that will motivate and guide future research in the field, focusing on aspects that have not been sufficiently studied or require improvement.

This review aspires to cover a substantial part of literature on molecular simulation of H_2_ systems. There are no restrictions on the years of literature (our literature review search covers studies until early 2025) or the type of systems considered. It is, however, important to note that we limit our attention only to classical molecular simulation, meaning that studies reporting quantum mechanical calculations are not discussed here (e.g., DFT, AIMD), except from some specific topics, for which the combination of DFT and classical molecular simulation is used as a norm. This choice was made due to the extremely high number of available studies using quantum mechanical approaches to study catalytic systems (e.g., H_2_ evolution reactions) and surface reactions (e.g., H_2_ storage on materials). In fact, these topics are already covered by several review articles. Nevertheless, to present a multi-scale perspective, the validity range and limitations of classical interaction potentials are discussed in detail, even for the cases where quantum effects of H_2_ play a significant role. Similarly, studies using reactive force fields or methods (e.g., quantum MC and MD) are not discussed, except for a few cases which are rendered unique or complementary to the topics presented here (e.g., liquefaction, membranes).

Another topic that is not exhaustively discussed in this review is H_2_ adsorption on all possible (two-dimensional) 2D and (three-dimensional) 3D (nano-)­porous materials. Substantial research efforts have been focused on studying H_2_ adsorption and separation using various materials, such as zeolites, metal organic frameworks (MOFs), covalent organic frameworks (COFs), graphene derivatives, borophane, boron nitride, and hydrides. Given the great number of available studies (in the order of thousands or more), we provide a focused overview primarily emphasizing recent advances in high-throughput screening using ML techniques. The permeation behavior of H_2_ through polymeric matrices and minerals is covered up to an extensive degree, with the focus being on materials and processes relevant to storage in high–pressure tanks and the subsurface. Again, a thorough analysis of all available studies involving the interaction of H_2_ with these families of materials is not feasible since a great number of polymeric and composite systems have been studied using molecular simulation to assess their performance as separation membranes. The latter application is discussed here, however, only with the purpose of providing an overview and a qualitative comparison between polymeric and other porous media for separations.

An area not discussed in this review is molecular modeling of H_2_ interactions with plasma-facing materials (PFMs), a crucial research direction for realizing fusion energy as a sustainable power source.^62^ At the atomistic scale, MD simulations have been performed by different research groups to reveal the mechanisms of H_2_ trapping and intragranular blister formation mainly in Tungsten (W) (i.e., the leading PFM material^63–65^ ) primarily using many-body (e.g., Brenner-type^66,67^ ), and recently also ML potentials.^62,64,68^ Despite its importance for fusion materials research, the study of H_2_-PFM lies outside the scope of the present review. For a comprehensive study on this topic, the reader is referred elsewhere.^63^

Given the extent and high density of information provided in this review, we have organized the manuscript in the following manner: In Section 2, the role of molecular simulation as a tool for predicting thermophysical properties, and for obtaining fundamental insight into each aspect of the H_2_ value chain is elaborated upon. Section 2 also constitutes a comprehensive map of the review, containing a brief overview of the most important properties, relevant conditions, and methods regarding each H_2_ technology considered, and referring the reader to the relevant sections in the rest of the manuscript, where more information and data are provided. Section 3 focuses on molecular simulation methods and models. Initially, an extensive discussion on the use, parameterization, and limitations of classical force fields used in MC and MD simulations is provided, followed by an outline of techniques that can be used in the frameworks of MD and MC to compute phase equilibria of gaseous and liquid mixtures of H_2_, transport coefficients, wettability and interfacial tension, hydrate properties, gas permeabilities in materials, and quantities related to H_2_ embrittlement. Section 4 constitutes a comprehensive review of available thermophysical properties of H_2_ systems computed with molecular simulation. Examples of the data reviewed and discussed are two-component vapor-liquid equilibria (VLE) of H_2_-H_2_O, three-phase (hydrate) equilibria of H_2_-H_2_O, self- and mutual diffusivities, electro-osmotic drag coefficients in membranes, adsorption of H_2_ onto materials, thermodynamic properties of wet compressed H_2_, adsorption of H_2_ onto rock formations, properties of hydrates (e.g., structural, kinetic, equilibria), liquid-vapor interfacial tensions of gas mixtures, high-throughput screening of materials for separations of H_2_ containing streams, and H_2_ embrittlement mechanisms. The discussion on the available literature also aims at identifying aspects that have not been sufficiently studied or discrepancies between different studies. Discussion on the role of AI and ML is also provided when applicable. Finally, in Section 5 a future outlook is compiled aiming to motivate new research in molecular simulations of H_2_ systems.

## 2 Role of Molecular Simulation in Advancing Hydrogen Technologies: Processes, Properties, and Relevant Conditions

In this section, we provide an overview of the role of molecular simulation in each stage of the H_2_ value chain, i.e., production, purification, transport, storage, and utilization. The focus is on the important properties that can be computed with molecular simulation and the respective relevant conditions for each technology/application. Since many thermophysical properties are relevant to multiple applications and aspects in the H_2_ value chain, this section serves as a comprehensive map for the rest of the review. To this purpose, every section below, aside from providing background information for different H_2_ technologies, it also points the reader to the appropriate segment in Section 4, where the relevant properties are reviewed in detail.

### 2.1 Hydrogen Production, Compression, and Utilization in Electrochemical Systems

H_2_ is commonly categorized based on the production pathway followed and the related emissions.^69,70^ Grey H_2_, the most prevalent form, is produced from natural gas via steam methane reforming. This process releases CO_2_ into the atmosphere. The process for producing black and brown H_2_ from coal emits both CO_2_ and CO, and is considered the most harmful to the environment. When combined with CO_2_ capture and sequestration, grey H_2_ becomes blue H_2_, where CO_2_ emissions are partly avoided. Blue H_2_ can act as a transitional stage between grey and green, enabling continued use of existing infrastructure during the transition. The production pathway via methane pyrolysis, yields the so-called turquoise H_2_, which has solid carbon as a by-product. In this review, the focus is on green H_2_ processes (production via water electrolysis using renewable sources). Nevertheless properties, and relevant methods to compute, e.g., phase equilibria, chemical reactions, and transport properties are equally applicable to the other H_2_ production routes.

The design and wide use of efficient electrolysis systems capable of replacing fossil-based H_2_ production with green H_2_ signifies a crucial step towards the future energy landscape.^13^ Achieving this necessitates the utilization of process modeling and precise understanding of the thermodynamic, electrochemical, and transport properties in various components of the electrolysers, including the balance of plant (BoP).^15,25,71–76^ However, the cost competitiveness of green H_2_ production poses a key challenge compared to fossil-based methods.^15^ Fuel cells, which convert stored H_2_ into electrical energy via the reverse process of electrolysis, complement the green H_2_ energy cycle. Fuel cell technology shares fundamental electrochemical and thermodynamic principles with water electrolysis, where modeling membrane morphology, water management, and ion transport are key areas into which molecular simulations can provide valuable insights. Similarly, electrochemical H_2_ compression (EHC),^77^ an innovative method for compressing H_2_ using electrochemical splitting and recombination of H_2_, operates on comparable principles. The physical insights into these technologies derived from molecular simulations are examined collectively in this review due to their shared fundamental principles and similar H_2_ mixtures involved.

EHC performs on similar electrochemical principles as a PEM fuel cell. The key difference is in the functions of the two devices, i.e., compressors are used to increase H_2_ pressure e.g., for storage and transport, while PEM fuel cells generate electricity and water from H_2_ and O_2_, serving as a power source.^78^ EHC requires optimal membrane hydration to maintain efficient charge transport through the membrane. This leads to a saturated H_2_ stream at the outlet of the compressor. Proper water management is therefore essential to ensure optimal performance and longevity of the membrane. The presence of even low water content in H_2_ introduces challenges for downstream of the EHC, for example the design of H_2_ refueling stations, which must adhere to the standard limit of 5 ppm water content.^79,80^ While minimizing humidity is critical for efficient H_2_ refueling, increased humidity in the inlet gas of the fuel cell improves its performance. In fuel cells, a decrease in membrane humidity leads to an increase in ionic resistance and ohmic losses.^81^ The application of molecular simulations becomes necessary in comprehending the inner workings of electrochemical compressors, wherein the fundamental mechanism involves the dissociation of H_2_ molecules to protons on the anodic side, and the subsequent formation of H_2_ on the cathodic side using electrical current.^77^

In the context of H_2_ production (electrolysis), utilization (fuel cell), and electrochemical hydrogen compression (EHC), molecular simulation is important for property prediction to aid the process modeling of these electrochemical systems. Due to the shared electrochemical principles between these technologies, our focus of discussion is mainly on electrolyzers. Without delving into the details of the different available electrolyzer technologies, the following types of electrolysis are currently available^82^ as shown in Fig. 2: alkaline water electrolysis (AWE), proton-exchange membrane water electrolysis (PEMWE), anion exchange water electrolysis (AEMWE), solid oxide electrolysis cell (SOEC), and proton conducting ceramic electrolysis (PCCEL).^82^ Using molecular simulations to predict properties of and obtain physical insight into charge transport and phase behavior of electrolyte solutions is particularly relevant since such solutions are the common element in all these electrochemical systems. The range of relevant conditions and the solution compositions vary depending on the type of electrochemical system and operating mode, as discussed in more detail later on.

**Figure 2:**
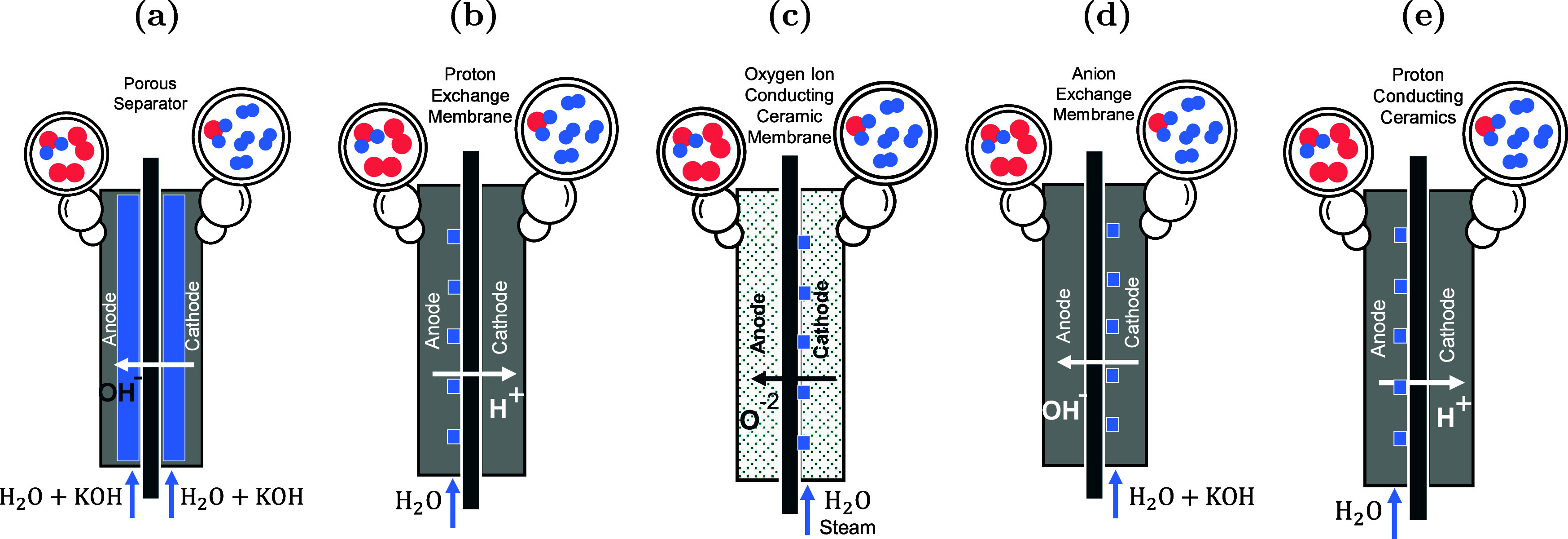
Schematic representations of different electrolyzer technologies for H_2_ production: (a) alkaline water electrolysis, (b) proton-exchange membrane water electrolysis, (c) solid oxide electrolysis cell, (d) anion exchange water electrolysis, and (e) proton conducting ceramic electrolysis. To design, optimize, and obtain fundamental understanding of hydrogen production technologies, molecular simulation is used to compute properties, common to all electrolysis processes, e.g., solubilities of hydrogen, oxygen, and electrolytes, mass transport of these species to and from the electrodes, viscosity of the aqueous electrolytes, electrical conductivity, and electro-osmotic drag in the membranes.

The high costs of green H_2_ production are largely driven by elevated electricity prices and regulatory challenges.^15^ While electricity costs are outside the scope of system design, efforts can focus on optimizing other factors impacting cost of green H_2_ such as enhancing the efficiency and safety of electrolysis systems.^73,74,83,84^ While the development of a cost-efficient electrolysis system also relies on factors such as the standardization of components, economies of scale, and efficient integration with renewable sources, these are not the focus of this review. Instead, we emphasize how molecular simulations can provide accurate thermodynamic and transport properties which are critical for optimizing green H_2_ production and utilization in electrochemical systems to further drive cost reduction. Typical thermodynamic properties necessary for process modeling of water electrolysis are electrolyte densities, solubilities of gaseous species (e.g., H_2_, O_2_) in aqueous electrolyte solutions, surface tension of concentrated liquid electrolytes, and partial pressures of water vapor.^75,76^ Thermodynamic and transport properties of aqueous potassium hydroxide (KOH), sodium hydroxide (NaOH), and sodium chloride (NaCl) solutions are particularly relevant for electrolysis and fuel cells.^85–89^ The dynamic viscosity of liquid electrolytes, mass transport expressed by the self- and mutual-diffusivities of all the species in the solution,^25,71,90,91^ and ion transport in membranes are representative examples of important transport properties required for the process modeling of electrolyzer cells. These properties can be computed with molecular simulations using different methods. A detailed list of thermodynamic and transport properties of various H_2_ systems relevant to electrochemical applications (i.e., production and utilization of H_2_) computed via molecular simulations and reported in literature is provided in Table 1 along with the respective ranges of conditions (e.g., temperatures, pressures, hydration of membranes). For each system listed in Table 1, we also refer the reader to the relevant methodology and detailed discussion in Section 3 and Section 4.

**Table 1:** Thermodynamic and Transport Properties Related to H_2_ Production and Utilization in Electrochemical Systems Computed with Molecular Simulation in Different Studies^i^

Property	System^Method^	Conditions
Fugacity Coefficient	H_2_-H_2_O^a^ ^,^ ^110^	264–323 K, 10–1000 bar
Chemical Potential	H_2_-H_2_O-KOH^a^ ^,^ ^111^	298–423 K, 10–400 bar, 0–8 mol KOH/kg H_2_O
	H_2_-H_2_O-NaCl^a^ ^,^ ^111^	298–423 K, 10–400 bar, 0–6 mol KOH/kg H_2_O
Solubility	H_2_-H_2_O^a^ ^,^ ^110^	310–423 K, 10–1000 bar
Vapor-Liquid Equilibrium,	H_2_-H_2_O^a^ ^,^ ^47^	264.21–272.4 K, 100–1000 bar
Activity	H_2_-H_2_O^b^ ^,^ ^48^	423, 477.59 K, 60.9–182.9 bar
	H_2_-H_2_O-KOH^a^ ^,^ ^111^	298–423 K, 10–400 bar, 0–8 mol KOH/kg H_2_O
	H_2_-H_2_O-NaCl^a^ ^,^ ^111^	298–423 K, 10–400 bar, 0–6 mol KOH/kg H_2_O
	H_2_/O_2_-H_2_O-KOH^a^ ^,^ ^112^	298–353 K, 1–100 bar, 0–8 mol KOH/kg H_2_O
	H_2_/O_2_-H_2_O-NaOH^a^ ^,^ ^112^	298–353 K, 1–100 bar, 0–8 mol NaOH/kg H_2_O
	H_2_-H_2_O-NaB(OH)_4_ ^a^ ^,^ ^113^	298–353 K, 1 bar, 0–5 mol NaB(OH)_4_/kg H_2_O
	O_2_-H_2_O, Nafion, Aquivion/Pt^d^ ^,^ ^114^	353.15 K, 2.92 < *λ* < 13.83
Joule-Thomson Coefficient	H_2_-H_2_O^c^ ^,^ ^47^	323 K, 100–1000 bar
Thermal Expansivity	H_2_-H_2_O^c^ ^,^ ^47^	323 K, 100–1000 bar
Heat Capacity		
Self-diffusion	Nafion, 3M, Hyflon^d^ ^,^ ^e^ ^93^	300, 353 K, 5 < *λ* < 14
	Dry Nafion^d^ ^,^ ^115^	150–600 K
	Hydrated Nafion^d^ ^,^ ^f^ ^116^	300 K, 3.44 < *λ* < 11.833
	Hydrated Nafion^d^ ^,^ ^117^	300, 350 K, 3.5 < *λ* < 16
	H_2_, O_2_, Nafion^g^ ^,^ ^118,119^	298 K, *λ* = 1
	H_2_, O_2_, H_2_O, SPEEK^d^ ^,^ ^120,121^	298–353 K, 4.9 < *λ* < 11.1
	H_2_O, H_3_O^+^, Ph-SPEEK^d^ ^,^ ^122^	300–400 K, 3.5 < *λ* < 40
	H_2_O, H_3_O^+^, CH_3_OH, Ph-SPEEK^d^ ^,^ ^123^	360 K, 8 < *λ* < 28
	H_3_O^+^, PFSA type membranes^d^ ^,^ ^124^	300 K, *λ* = 3
	H_2_O, Nafion, Aquivion^d^ ^,^ ^125^	298 K, 3 < *λ* < 18
	H_2_-H_2_O-KOH^a^ ^,^ ^112^	298–353 K, 1–100 bar, 0–8 mol KOH/kg H_2_O
	H_2_-H_2_O-NaOH^a^ ^,^ ^112^	298–353 K, 1–100 bar, 0–8 mol NaOH/kg H_2_O
	O_2_-H_2_O-KOH^a^ ^,^ ^112^	298–353 K, 1–100 bar, 0–8 mol KOH/kg H_2_O
	O_2_-H_2_O-NaOH^a^ ^,^ ^112^	298–353 K, 1–100 bar, 0–8 mol NaOH/kg H_2_O
	H_2_-H_2_O-NaB(OH)_4_ ^a^ ^,^ ^113^	298–353 K, 1 bar, 0–5 mol NaB(OH)_4_/kg H_2_O
Electrical conductivity	H_2_O-NaB(OH)_4_ ^a^ ^,^ ^113^	298–353 K, 1 bar, 0–5 mol NaB(OH)_4_/kg H_2_O
	Dry Nafion^d^ ^,^ ^115^	150–600 K
	H_2_, O_2_, Nafion^g^ ^,^ ^118,119^	298 K, *λ* = 1
	H_2_O, H_3_O^+^, Ph-SPEEK^d^ ^,^ ^122^	300–400 K, 3.5 < *λ* < 40
	H_2_O, H_3_O^+^, CH_3_OH, Ph-SPEEK^d^ ^,^ ^123^	360 K, 8 < *λ* < 28
	H_3_O^+^, PFSA type membranes^d^ ^,^ ^124^	300 K, *λ* = 3
Thermal Conductivity	H_2_O, Nafion^d^ ^,^ ^126^	300–350 K, 3 < *λ* < 20
Electro-osmotic drag	H_2_O, cylindrical pore of Nafion 117^d^ ^,^ ^127^	298 K, < 7.65 *λ* < 20.9
	H_2_O, Nafion 117^h^ ^,^ ^128^	287.15–364.65 K, *λ* = 20
	H_2_O, H_3_O^+^, Nafion^g^ ^,^ ^129^	363 K, 4.25 < *λ* < 12.75
	H_2_O, H_3_O^+^, Nafion^g^ ^,^ ^94^	330–420 K, 5 < *λ* < 20
	H_2_O, H_3_O^+^, Nafion^g^ ^,^ ^94^	350 K, 1 < *λ* < 2

a Continuous fractional component Monte Carlo.

b Monte Carlo.

c Ensemble fluctuations.

d Grand-equilibrium method; molecular dynamics.

e Multistate empirical valence bond.

f Reversible reference system propagator algorithms.

g Molecular dynamics with reactive force fields.

h Non-equilibrium molecular dynamics.

i The superscripts in the table denote the simulation method used for each system. *λ* is the water content in the systems with membranes (see Eq. ([Disp-formula eq31])). The table points the reader to the appropriate sections in this review where the relevant methods and discussion are provided. The relevant methods and in-depth discussions per property can be found in this review in the following sections: Fugacities, chemical potentials, solubilities, activities, and vapor-liquid equilibrium: Section 3.3.1 (methods), Sections 4.1.2.1, 4.1.2, and 4.1.1 (discussion). Joule-Thomson coefficient, thermal expansivity, and heat capacities: Section 3.3.4 (methods) and Section 4.1.4 (discussion). Diffusivities: Sections 3.2.4 and 3.2.3 (methods), and Section 4.1.5 and Section 4.1.7 (discussion). Electroosmotic drag: Section 3.2.12 (methods) and Section 4.1.6 (discussion).

Membrane electrode assemblies (MEAs) are critical components in electrochemical systems.^92^ The membranes allow proton transport in acidic environments and hydroxide ion transport in alkaline environments while blocking the passage of gases, ensuring efficient charge transport and water management.^93^ The conductivity of a polymer in a PEM/EHC is influenced by its crystallinity, equivalent weight, hydration level, side chain length, and morphology, making the optimization process complex and time-consuming due to the wide range of parameters involved. Effective water management in MEAs (hydration level) is essential, balancing electro-osmotic drag and back diffusion of water at different current densities. Electro-osmotic drag causes water to move across the membrane as protons migrate through the membrane under the influence of the imposed electric field.^92,94^ Concurrently, back diffusion occurs due to the difference in water concentration between the cathodic and anodic compartments. Another related critical topic is understanding and mitigating degradation in MEAs. As an example, the contamination of ionomer membranes by e.g., CO^2+^, Na^+^, can significantly disrupt the morphology of the membrane, specifically blocking ion exchange sites. The presence of contamination can impair the flow of reactant gases in the catalyst layer. Additionally, it can hinder the diffusion of hydronium ions (H_3_O^+^) and water molecules, which are critical for maintaining proton conductivity and hydration within the membrane. Such effects can be rigorously studied and understood via molecular simulations, which offer detailed insights into the structural and transport properties of ionomers under various contamination scenarios. To this purpose, molecular simulations are performed to model membrane morphologies, sorption, percolation, densities, glass transition temperatures, and ion transport.^92,94–101^ The influence of these contributions can be isolated in molecular simulations which is challenging to achieve in experiments.^93^ Classical MD simulations can capture the vehicular mechanism of charge transport, while reactive force fields and Ab-initio MD^102–105^ can potentially capture the Grotthuss mechanism, since bond breaking and formation during the simulation is required.^93^ Molecular simulations have also been used in the literature for modeling the membrane and ion transport within the membrane of electrochemical compressors.^92,97^ Additionally, molecular simulations can be used to determine the water content in and the thermodynamic properties of the compressed H_2_ downstream of the electrochemical compressor.^47,106–110^ Fig. 3 highlights various thermophysical properties which are crucial for the process design and optimization of two widely used electrochemical technologies (AWE and EHC) computed with molecular simulation.

**Figure 3:**
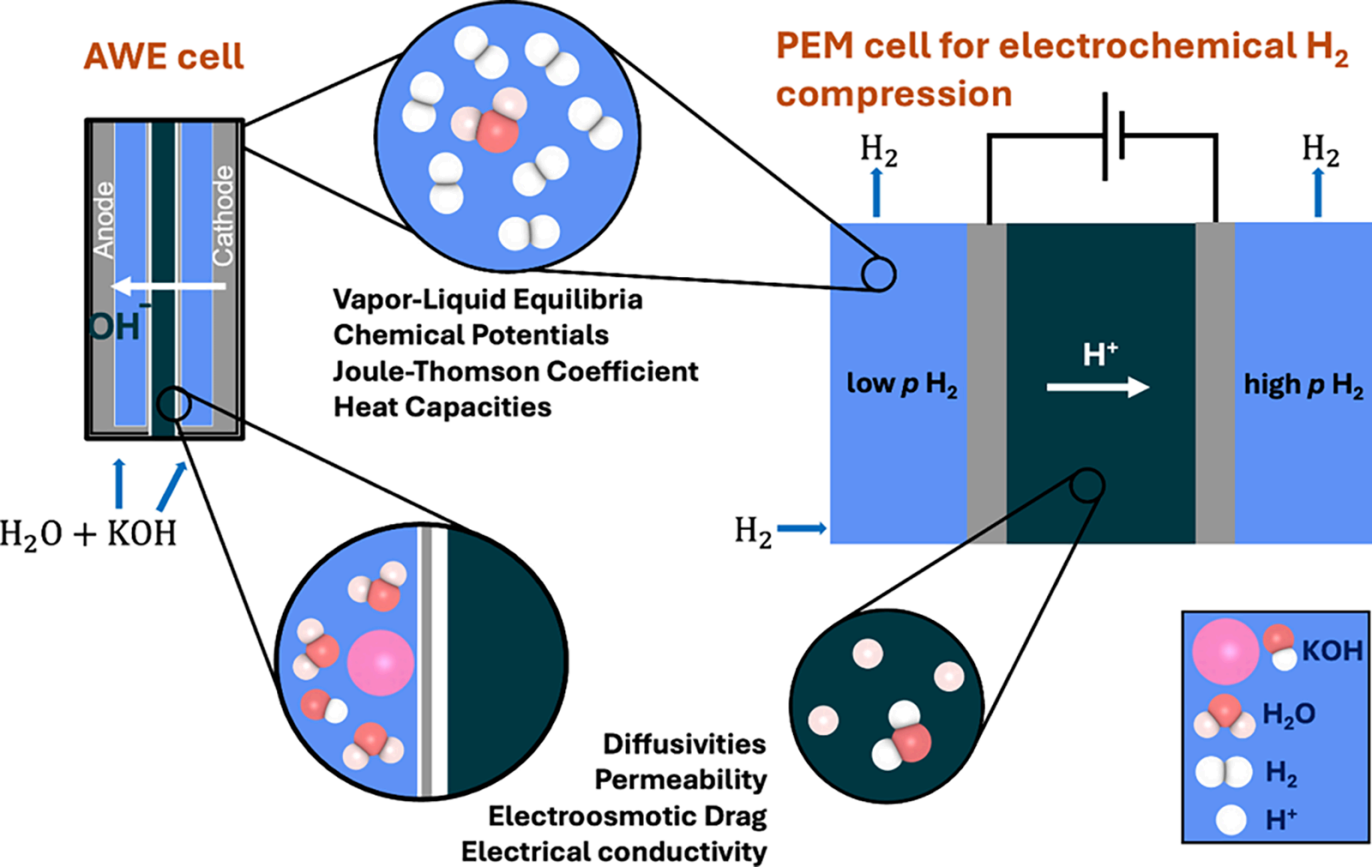
Schematic of alkaline water electrolysis and electrochemical H_2_ compression in a proton exchange membrane cell highlighting areas where molecular simulations are crucial, e.g., electrical conductivity, electro-osmotic drag, heat capacity, Joule-Thomson coefficients, and vapor-liquid equilibrium.

### 2.2 Purification and Separation of Hydrogen Streams

#### 2.2.1 Separations with Membranes

Membranes provide a potentially sustainable alternative to traditional separation methods of H_2_ streams.^130–132^ An important property of membranes is permeability (*P_i_*). Membrane permeability quantifies the molecular-level transport of substances across thin barriers and differs significantly from other types of permeabilities reported in the literature, such as Darcy’s permeability,^133^ widely used in subsurface storage and porous media research. Membrane permeability is defined as *P_i_* = *S_i_D_i_*, where *S_i_* is the solubility of species *i* (which reflects its affinity with the membrane material) and *D_i_* is the diffusivity (which is a measure of the rate of molecular transport of the species within the membrane). *P_i_* is commonly expressed in Barrer, a unit named after Richard L. Barrer,^134^ a pioneer in membrane science. One Barrer represents the permeability of a membrane of 1 cm thickness that allows the passage of molecules occupying 1 cm^3^ at standard temperature and pressure (STP: 273.15 K and 1 atm) per second through a 1 cm^2^ of membrane area under a pressure difference of 1 cmHg. A related property, membrane permeance, measures the flux of a substance per unit driving force and membrane thickness. It is typically expressed in Gas Permeance Units (GPU), where 1 GPU corresponds to the permeance of a membrane that allows 1 cm^3^ of gas (STP) to pass through 1 cm^2^ under a pressure difference of 1 cmHg. Expressing permeability in GPU is convenient because the definition inherently accounts for membrane thickness, which can be useful when this parameter is unknown or varies between samples.

In membrane science, selectivity is expressed as the ratio of the permeabilities, *P_i_*/*P_j_*, of two species *i* and *j*, as schematically shown in Fig. 4. While this ratio must be high enough to comply with the needs of a given process, at the same time a minimum permeability for the fast-permeating gas (e.g., *P_i_*) should be ensured. Otherwise the overall process is regarded as of discouragingly low performance for industrial applications.

**Figure 4:**
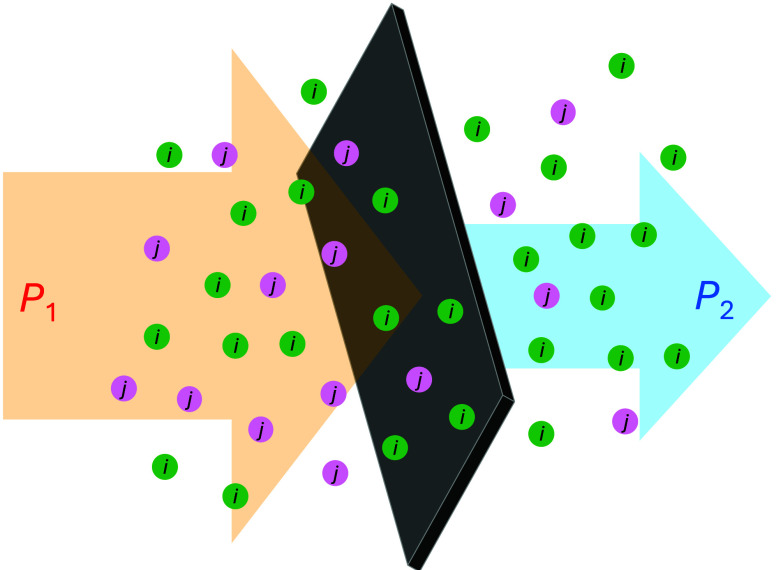
Schematic representation of a membrane separation process, showing the feed side of a mixture *i* and *j* at higher pressure (*P*_1_), permeate at lower pressure (*P*_2_) across the membrane.

Polymeric membranes are extensively used in industrial-scale separations due to ease of fabrication, cost-effectiveness, and scalability. Polymeric membranes are classified into isotropic, which have a uniform composition and pore structure throughout their thickness, and anisotropic, which have varying pore structures and compositions across their cross-section.^135^ Gas separation in polymeric membranes is driven by both solubility and diffusivity selectivity. While the well–known permeability/selectivity trade–off is fundamentally diffusion-dominated,^136^ chemical modifications often improve performance by increasing solubility selectivity. For example, introducing polar or CO_2_ – philic groups can enhance the uptake of condensable gases (e.g., CO_2_, CH_4_, O_2_) and shift materials upward relative to the Robeson limits. For H_2_ – related separations, this has asymmetric consequences. Because H_2_ is a non-condensable gas, and its solubility is difficult to increase, membranes aiming for high H_2_ permeation selectivity (H_2_/CO_2_, H_2_/CH_4_) remain largely controlled by diffusion. In contrast, increasing the solubility of the heavier partner can substantially improve CO_2_/H_2_ or CH_4_/H_2_ selectivity, which explains why many polymer modifications benefit processes focused on capturing CO_2_ or CH_4_ from H_2_ – rich streams but have limited impact on designing highly H_2_ – selective membranes.

This observation creates a significant trade-off between permeability and selectivity, as it is shown in Fig. 5 where experimental results from the literature for separations of H_2_/CH_4_ (Fig. 5(a)) and H_2_/CO_2_ (Fig. 5(b)) mixtures with polymeric membranes are indicated with red color. This limitation hinders the widespread use of polymeric membranes for separations of H_2_ streams, and has spurred research into exploring alternative materials that can overcome these intricacies.

**Figure 5:**
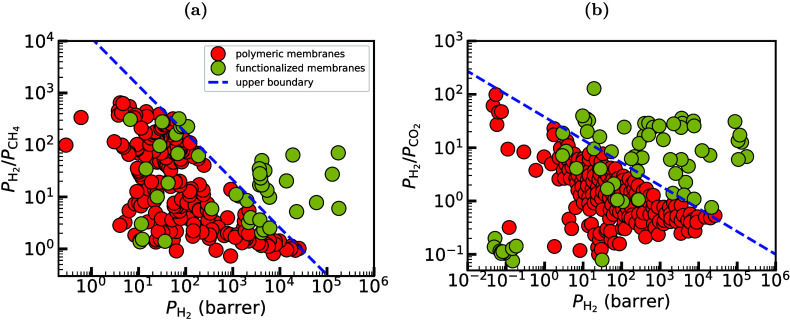
Representative performance assessment in the form of Robeson plots for (a) H_2_/CH_4_ and (b) H_2_/CO_2_, comparing experimental results on polymeric and functionalized nanoporous membranes (e.g., MOFs, COFs, zeolites, carbon-based, mixed matrix membranes). Polymeric membranes literature results taken from the seminal work of Robeson.^136^ The data from literature for the functionalized materials were collected from an extended survey of the authors and can be found in Tables 15 and 16.

Crystalline nanoporous materials have surfaced as a prominent class of candidates for addressing the drawbacks of polymeric membranes. These materials have high permeability and selectivity due to their unique structure. Nano-porous materials achieve separations of mixtures via primarily size and shape exclusion. This is due to the small, well-defined windows or apertures connecting internal cavities or narrow channels within the structures of the materials. The apertures act like molecular sieves, allowing smaller molecules to pass through while blocking larger ones.^137,138^ The rigid structure of crystalline nanoporous materials provides more precise control over which molecules can pass, unlike polymeric membranes that typically rely on diffusion through flexible, less structured pathways.

While zeolites and activated carbons are widely used materials for H_2_ gas separations in the industry,^139–143^ due to the limited tunability of pore sizes of zeolites, attention has been directed towards exploring functionalized materials. The most prominent classes of such materials are MOFs (i.e., materials consisting of metal clusters linked with organic ligands)^144^ and COFs (i.e., materials composed ogranic molecules connected through covalent bonds).^145^ Membranes can be made solely based on MOFs or COFs, or in a hybrid form by using polymers as a matrix and nanoporous materials as fillers, resulting in the so-called mixed-matrix membranes (MMMs).^146^ One of the most fascinating features of MMMs is that they can be modified at the molecular level (by using e.g., different fillers, varying filler compositions, different polymer matrices) to tailor their macroscopic properties.^147^. This versatility opens an almost boundless array of possibilities for achieving desired separation results within the H_2_ value chain.^148^ This also becomes evident by the increased performance of functionalized membranes made of MOFs and COFs, compared to the limited performance of pure polymeric membranes shown in Fig. 5.

Due to the virtually limitless ways of tailoring the separation performance of these materials through functionalization, achieving the desired results solely in the laboratory is a strenuous, time-consuming, and financially demanding process. Therefore, molecular simulation has emerged as a powerful computational tool for exploring modification-performance correlations and aiding the development of highly H_2_-selective membranes.^149^ MC and MD simulations are particularly valuable for computing properties related to performance metrics in membrane separations. Such properties are the solubilities, diffusivities, permeabilities, selectivities, and heats of adsorption of H_2_ and other gases in the membranes. A brief but comprehensive review of the literature regarding these properties for pure H_2_ and different mixtures (with e.g., CO_2_, CH_4_^′^, N_2_, He,) is provided in Section 4.6 for various materials (i.e., MOFs, COFs, MMMs, and zeolites). This discussion also covers properties such as the storage capacities, which are mainly useful for the design and evaluation of H_2_ storage systems as briefly covered in Section 2.3.2. Relevant details on models and methods widely used to perform simulations of nanoporous materials and membranes are provided in Sections 3.1.4, 3.2.5, and 3.3.2.

With the advent of high performance computing, the execution of hundreds or even thousands of simulations is now feasible, allowing the exploration of wide design spaces of functionalized materials.^149^ Assisted by ML techniques, these complex correlations become easier to uncover, as discussed in more detail in Sections 4.6.1 and 4.6.2. As shown in Fig. 6, ML leads to a paradigm shift in the development of separation membranes, enabling the transition from a hypothesis-driven approach, limited to trial-and-error, to a data-driven approach.^150–158^

**Figure 6:**
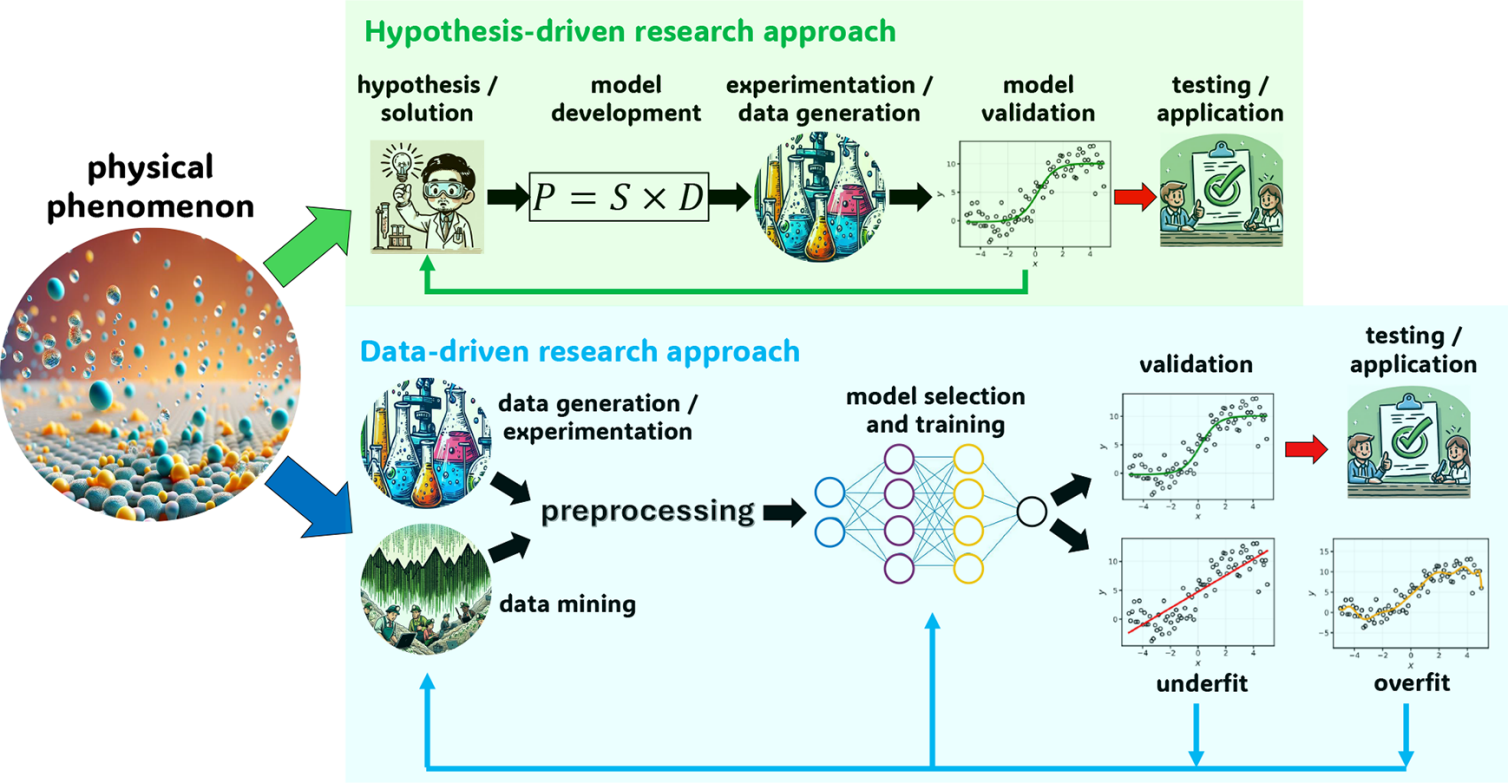
Schematic showing hypothesis (traditional) and data (modern)-driven research approaches. In the traditional route, a hypothesis is usually made in the beginning. Based on this hypothesis models are devised, and experiments are performed to test its validity. In the modern route, data are produced, analyzed, processed, and used to train machine-learning models. The outcomes of these models are validated and used to fine-tune the model. This approach is revolutionizing the study of H_2_ processes involving porous materials. The figure is inspired from Fig. 2 of ref 150.

In addition to membrane-based separations, large-scale H_2_ purification is frequently carried out using adsorption processes such as pressure-swing adsorption (PSA) and temperature-swing adsorption (TSA). Both these processes rely on packed beds of porous adsorbents, often zeolites or activated carbons, and comprise the industrial state-of-the-art for H_2_ recovery from complex mixtures. Although PSA/TSA operate on different principles than selective transport across membranes, both approaches benefit from molecular–scale insights into adsorption equilibria, energetics, and diffusion. Discussion on how high–throughput molecular simulations combined with ML approaches can be an important tool for selecting high–performing adsorbents for PSA and TSA processes is provided in Sections 4.6.1 and 4.6.2. For details relevant to PSA process design and optimization, the reader is referred to the studies of Park et al.^159^ and Ribeiro et al.^160^

#### 2.2.2 Hydrate-Based Separations

Clathrate hydrates, or simply hydrates, are crystalline, non-stoichiometric, inclusion compounds consisting of a hydrogen-bonded network of water molecules that form three dimensional cavities (cages) inside which guest molecules of appropriate size can be enclathrated (i.e., encaged).^161,162^ The water molecules can self-assemble into forming cages under appropriate conditions (i.e., high pressures and/or low temperatures). The cages are further stabilized by weak van der Waals interactions between the water molecules in the lattice and the encaged guest molecules. The most common hydrate structures in nature are the sI, sII, and sH, which result from combinations of different numbers and types (i.e., pentagonal dodecahedron, 5^12^; tetrakaidecahedron, 5^12^6^2^; hexacaidecahedron, 5^12^6^4^; icosahedron, 5^12^6^8^; irregular dodecahedron, 4^3^5^6^6^3^) of cages which form the unit cell of each hydrate structure. Table 2 shows the structural characteristics of the sI, sII, and sH hydrate structures.

**Table 2:** Structure and Properties of the Three Most Common Types of Clathrate Hydrates in Nature^161^

hydrate structure	sI		sII		sH		
crystal system	Primitive cubic		Face-centered cubic		Hexagonal		
space group	*Pm3n*		*Fd3m*		*P6/mmm*		
cavity type (notation)	small (S)	large (L)	small (S)	large (L)	small (S)	medium (M)	large (L)
Description	5^12^	5^12^6^2^	5^12^	5^12^6^4^	5^12^	4^3^5^6^6^3^	5^12^6^8^
cavity radius (Å)	3.95	4.33	3.91	4.73	3.94	4.04	5.79
cavities/unit cell, *n_i_*_,*k*_	2	6	16	8	3	2	1
H_2_O/unit cell, *N_w_*_,*k*_	46		136		34		
Unit cell formula	2*S* · 6*L* · 46 H_2_O		16*S* · 8*L* · 136 H_2_O		3*S* · 2*M* · 1*L* · 34 H_2_O		

Hydrates have attracted significant interest from the industry and academia due to their ability to selectively incorporate particular molecules of appropriate size in their crystal structure. This makes hydrates attractive candidates for a number of industrial applications, including the storage/transport of energy carriers such as H_2_ and CH_4_ ^163–166^ and environmental gases such as CO_2_,^167,168^ gas mixture separations, water purification,^169^ and desalination.^170^ In particular, two industrial applications of hydrates are of significant importance within the H_2_ value chain: (i) the storage (stationary or mobile applications) and transportation of H_2_ inside hydrates^171,172^ (i.e., hydrate slurries or solid hydrates in pellet form), and (ii) hydrate-based separation of the H_2_/CO_2_ binary gas mixture resulting from the so-called integrated coal gassification cycle (ICGC) plants, where the CO_2_ is captured before fuel combustion (i.e., precombustion capture^173^). A hydrate-based separation of a gas mixture is the direct result of the selective incorporation of gas molecules from the mixture of appropriate size in the hydrate crystal structure.^174^ Fig. 7 shows a cartoon representation of a single-stage hydrate-based separation for the general case of a gas mixture consisting of components A and B. The process works as follows: Initially, a gas mixture of A and B is in contact with water. The pressure and temperature conditions are tuned to induce hydrate formation, which is followed by separation of the solid hydrate phase and the subsequent hydrate dissociation. Two gas streams, an A-rich (i.e. the ”unreacted” gas mixture which remained after hydrate formation), and a B-rich (i.e., resulting from hydrate dissociation), are produced, each of which has different compositions from the initial one. After implementing a number of hydrate formation/dissociation stages, gas streams having a certain degree of required purity are obtained. For extensive discussions on the experimental and computational aspects of hydrate-based H_2_-mixture separations, the reader is referred to the review articles by Babu et al.,^173^ Tsimpanogiannis et al.,^175^ and Hassanpouryouzband et al.^176^

**Figure 7:**
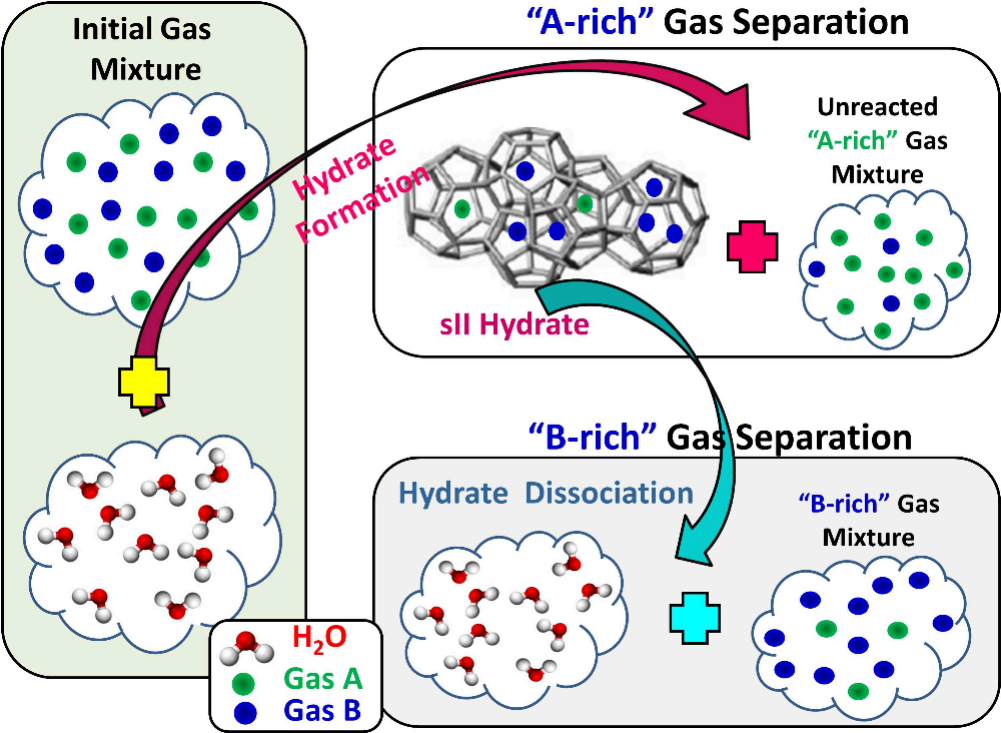
Schematic of a single-stage, hydrate-based, separation of a binary gas mixture ”A + B”, where A and B are the two gas components.

Molecular simulation is an important tool for computing various thermodynamic, transport, kinetic, and structural properties of interest to H_2_ hydrate-related applications. Table 3 shows a number of such properties that can be obtained via MD and MC simulations. Table 3 also guides the reader to the corresponding sections in this review where particular properties and methods are further discussed for the case of pure or mixed H_2_ hydrates. It should be noted that some of these properties can also be obtained by means of first principle calculations (e.g., DFT^177^). For detailed discussions on DFT-based calculations relevant to H_2_ hydrates, the reader is referred elsewhere.^178–182^

**Table 3:** Important Thermophysical, Transport and Structural Properties for Pure or Mixed H_2_ Hydrates^a^

Property	Methodology	Discussion
Phase (*p*, *T*) equilibria	Section 3.2.14	Section 4.8.1
Structure	Section 3.2.15	Section 4.8.3
H_2_ diffusivities	Section 3.2.3	Section 4.8.8
Kinetic rates	Section 3.2.15	Section 4.8.2
Promoter identification	Section 4.8.7	
Storage capacities	Section 3.3.3	Sections 4.8.5 and 4.8.4

a The reader is referred to the sections in this review where the respective methods (methodology) and review (Discussion) of the available literature are located.

Three-phase equilibria is among the most important thermodynamic properties for the design stages of hydrate-based processes. Fig. 8 shows a typical phase diagram of a hydrate-forming system that has a lower (Q_1_) and an upper (Q_2_) quadruple point. The solid lines indicate where the three-phases are at equilibrium, while the two stars indicate the quadruple points, i.e., where four-phase equilibria occurs. Certain hydrate-forming systems, such as CH_4_, N_2_, and Ar, exhibit only Q_1_, while others, e.g., CO_2_, H_2_S, C_2_H_6_, C_3_H_8_, exhibit both Q_1_ and Q_2_. Experimental measurements, continuum scale modeling and molecular simulations can be used for the estimation of hydrate phase equilibria. The vast majority of continuum scale modeling studies has been performed by an approach that couples van der Waals–Platteeuw statistical theory,^183^ used for describing the solid solution, with an EoS that describes the vapor-liquid equilibria. The seminal work of Parrish and Prausnitz,^184^ and the later modifications,^185,186^ introduced to overcome some of the initial limitations of the theory, are capable of providing a satisfactory description of the phase equilibria for the most common hydrate systems. For a detailed review on this, the reader is referred to the study by Medeiros et al.^187^

**Figure 8:**
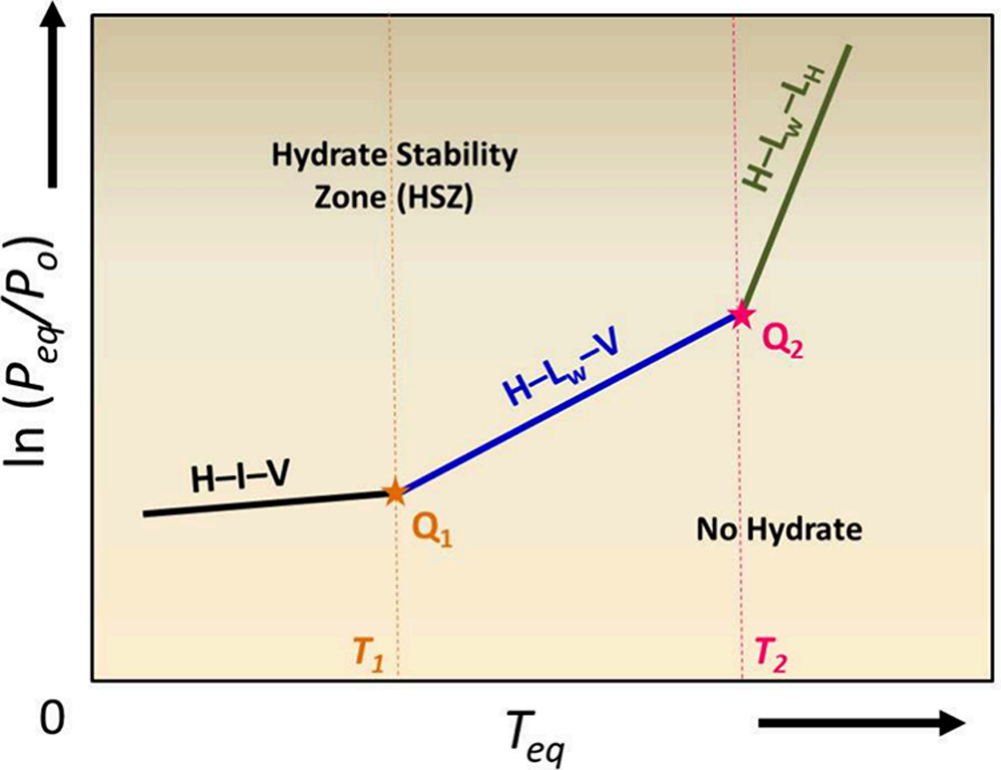
Pressure-equilibrium temperature diagram showing the three-phase equilibria curves for a hydrate-former with lower (Q_1_) and upper (Q_2_) quadruple points. Phase notation: H – hydrate; L_*w*_ – liquid water; I – ice; V – vapor; L_*H*_ – liquid hydrate-former.

The study of hydrate equilibria with MC and MD simulations can provide a deeper understanding of the related microscopic phenomena. Such molecular-scale methodologies can be used in a complementary role to the experimental and continuum-scale approaches to aid the design of new hydrate-related processes. Additional properties of interest reported in literature are the enthalpies of hydrate dissociation (Δ*H* ^dissoc^), the enthalpies of enclathration (Δ*H* ^encl^), isobaric heat capacities (*C_p_*), hydrate lattice expansivities, and separation efficiencies. Interestingly, no molecular simulation data regarding these properties are available for H_2_ containing systems, despite the fact that these properties are essential for the design/optimization of industrial processes involving hydrate-based systems. This clearly shows that ample room is available for future computational studies of H_2_-containing mixtures capable of forming hydrates.

#### 2.2.3 Separations Using Ionic Liquids

As discussed earlier, membranes comprising polymeric and nanoporous materials can be used to efficiently separate H_2_ streams under various conditions and for different applications. High selectivities and adsorption capacities, have also been reported using solvents based on ionic liquids (ILs) and deep eutectic solvents (DES). ILs are salts (comprising only ions) with melting temperatures orders of magnitude lower than common salts, e.g., NaCl. The melting *T* of ILs is below 100 °C, and for some components (the so-called room temperature ILs) it can even be below ambient *T*. The broad liquid range, combined with other properties such as the negligible vapor pressures, thermal stability, and high degree of tunability, makes ILs an interesting class of materials for a wide variety of processes. In addition, in the past 50 years ILs have been considered for use in industrial applications as an environmentally friendlier solvent compared to common organic components.

ILs have found application also in extraction (e.g., liquid-liquid) and separation (e.g., gas) processes, usually in the form of IL-supported membranes. For more details on ILs and their applicability the reader is referred elswhere.^188–191^ To the best of our knowledge, no molecular simulation studies investigating relevant H_2_/DES properties are available in literature. Contrary, studies on sorption, diffusivities, and interfacial properties of H_2_ (and mixtures of H_2_ with other gases) in ILs computed via MC and MD simulations are available. Nevertheless, the number of these studies is limited compared to e.g., CO_2_/ILs or CH_4_/ILs systems. Evidently, the main motivation for performing molecular simulations of H_2_/ILs is the study of CO_2_/H_2_ separation, which is crucial for pre-combustion processes. Predicting properties for H_2_ separations from N_2_ and CH_4_ are also common in the available molecular simulation literature owing to the importance of these mixtures for the design of carbon capture technologies. Since mixed-gas experiments are usually challenging to conduct, molecular simulations offer a compelling and efficient complementary method for predicting and understanding the absorption selectivities of IL/gas systems.

Based on our review of the available molecular simulation literature, H_2_ appears to have very low solubilities in ILs compared to other gases, which increases with temperature (contrary to some experimental findings). CO_2_/H_2_ selectivity in ILs dramatically decreases with temperature, while, computations of H_2_ diffusivities show that it diffuses up to 12 times faster than CO_2_. The full review of this topic is provided in Section 4.5, along with details regarding discrepancies between simulations and experimental data. For relevant methods and models, the reader is referred to Sections 3.1.3, 3.2, and 3.3.1.

### 2.3 Hydrogen Storage

The high gravimetric energy density of H_2_ (ca. 120 MJ/kg), nearly two and a half times higher than conventional fossil-based fuels such as methane, propane, diesel, and gasoline (see Fig. 9), makes it an attractive energy carrier. However, focusing solely on gravimetric energy density does not provide a complete picture regarding the widespread application of H_2_, due to the storage challenges associated with its low volumetric density (ca. 0.010 MJ/L at 20 °C).^22,192^ Typically, commercially compressed H_2_ gas (CHG) is stored at pressures up to 700 bar. CHG at 300 bar has a density of 24 kg/m^3^ at 15 °C, while at 700 bar and the same temperature, the density increases to 40 kg/m^3^ as shown in Fig. 10.

**Figure 9:**
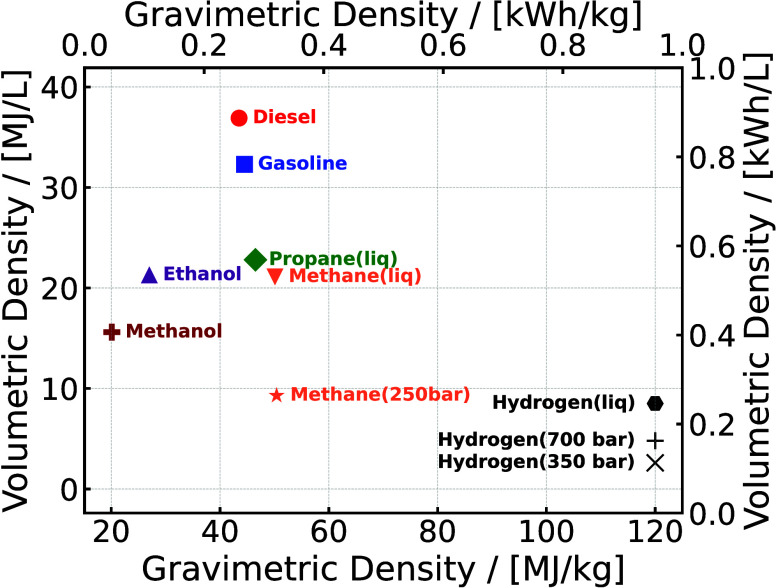
Gravimetric and volumetric energy densities of hydrogen compared to other fuels at ambient temperature. The data are extracted from refs 38 and 193.

**Figure 10:**
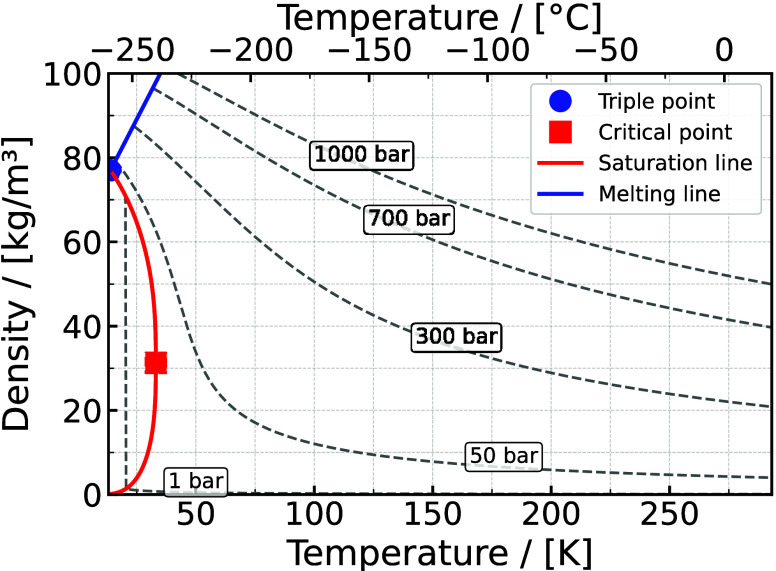
Hydrogen density - temperature diagram for different isobars. Data are taken from NIST.^194^

Liquid H_2_ (LH) at cryogenic temperatures has densities up to 70 kg/m^3^. Subcooled liquid H_2_ (SLH), which achieves a density of 62 kg/m^3^ at -245 °C and 16 bar, is produced by slightly increasing the pressure of LH using state-of-the-art compression systems.^195,196^ SLH storage faces challenges due to boil-off, estimated to last between 10 and 200 hours depending on the state of charge of the system.^195^ Cryo-compressed H_2_ (CCH), combining low temperatures with high pressures, and LH are other storage types, although the technology readiness level (TRL) for CCH remains low.^195^

Cost-effective, high energy density H_2_ storage is a crucial part of the future sustainable energy landscape. The need to discover (at the material level) and design (at the system level) improved media for H_2_ storage is specifically emphasized by the US Department of Energy (DOE).^22,197,198^ Molecular simulations play a central role in addressing the material challenges and in modeling H_2_ storage processes, especially under elevated pressures and temperatures (relevant to *e.g.*, underground storage), providing unique insights into the physical-chemical mechanisms governing the behavior of the systems at hand. Such insights are almost impossible to achieve through experimental methods alone, which are limited by both safety, cost, and resolution constraints. To this end, the prediction of relevant thermophysical properties and insights obtained from molecular simulations can guide the design of more efficient H_2_ storage systems.^77,199–201^ This review focuses solely on classical molecular simulations related to three different types of storage, namely compressed H_2_ for vehicular applications, storage in materials, and underground H_2_ storage. It is important to note that this work does not extensively cover H_2_ storage in materials, since this field is already well-documented in the literature, particularly regarding the vast array of nanoporous and 2D nano-materials. However, several key references to comprehensive reviews are provided on this topic for further reading.

#### 2.3.1 High Pressure Storage of Gaseous Hydrogen in Tanks

The pathway to achieving a less carbon-intensive mobility sector relies on the use of battery electric vehicles (BEVs) and fuel cell electric vehicles (FCEVs). Usually, BEVs are most suitable for short-range commutes, while FCEVs are preferred for medium to long-haul applications.^193,195,202–206^ Currently, CHG at 70 MPa and 35 MPa is used in light-duty vehicles, while an inlet pressure of up to 875 bar is required in on board H_2_ storage tanks to refuel the FCEV within 3 to 4 minutes.^79,201,207–215^

In FCEVs of all types, e.g., trucks, light vehicles, buses, H_2_ is stored at high pressures in different tanks of varying type and size. The main types of tanks are classified as I, II, III, IV, and V.^216,217^ Type I tanks typically operate at a maximum pressure of 200 bar, with a gravimetric storage density of 0.01 to 0.02 kg_H_2__/kg. Type II tanks, with a maximum pressure of 300 bar, reduce weight by 40% compared to type I tanks, but are still less efficient for H_2_ storage due to a 50% increase in manufacturing costs.^216,217^ Both types are unsuitable for mobility applications because of their low storage density. In contrast, type III and IV tanks, which operate between 450 and 700 bar, provide significantly higher H_2_ storage capacity and are commonly used in fuel cell vehicles. Type IV tanks, in particular, are widely used due to their lightweight composite structure, capable of withstanding pressures up to 700 bar or higher.^216,217^ Type V tanks, though still in development, are expected to offer even greater performance.^216,217^

Due to the high pressures in the tanks (e.g., 700 bar), H_2_ gas experiences a strong driving force to escape. To mitigate this thermodynamic phenomenon, it is common that thermoplastic polymers and composite materials are used in the tanks as barrier materials (i.e., inner liners) that provide H_2_ gas containment. To this purpose, developing polymers and composites with low inherent H_2_ permeability is crucial. This requires extensive research involving a wide spectrum of materials tested under high pressures, which is not trivial to do with experiments alone. Molecular simulation can make a substantial contribution towards advancing H_2_ storage systems under high pressures, which are essential for FCEVs.^218^ The insights from molecular simulations allow for the understanding of H_2_ interactions with the liner materials, which affect permeation resistance and gas barrier properties.^218^ By studying these interactions at the nano-scale, molecular simulations help assess the suitability of polymeric and composite materials, thus, providing crucial insights into the performance and safety of H_2_ storage systems. Molecular simulation studies in this field have mainly focused on studying H_2_/polymer systems relevant to IV-type hydrogen tanks. The most common polymers in these tanks are polyamide-6 (PA6), polyethylene (PE) and high density polyethylene (HDPE), and composite materials such as graphene/PA6. Molecular simulation studies of synthetic rubbers, such as ethylene propylene diene monomer (EPDM), used mainly as sealing components (e.g., gaskets, hose liners, O-rings) in H_2_ energy/storage infrastructure, are also available in the literature.

In Section 4.4 we provide a detailed review of the available molecular simulation literature reporting thermophysical properties of H_2_/polymer (and composite) systems. These studies mainly report diffusivities (and diffusion mechanisms), solubilities (and sorption), permeabilities of H_2_ in the solid phases, and polymer-specific properties such as the material fractional free volume (FFV), specific volume, and density. Our analysis of the available literature revealed that there is a large data variability among the simulation studies. For example, the computed H_2_ solubilities and permeabilities span three orders of magnitude across different studies, despite using the same (or similar) force fields and methods. In addition, we show that while molecular simulations capture experimental permeabilities qualitatively, quantitative differences exceed three orders of magnitude clearly indicating that there is ample room for improved predictions. For a description of the relevant molecular models and methods for computing the thermodynamic and transport properties of H_2_/polymer systems, the reader is referred to Sections 3.1.4, 3.2.4, 3.2.5, 3.3.1, and 3.3.2.

#### 2.3.2 Storage in Materials

To avoid working at high pressures (as discussed in Section 2.3.1) and/or at low temperatures (see Section 2.4.3), H_2_ can be stored in solids and liquids.^22^ Materials can store H_2_ via physical (physisorption, i.e., via weak electrostatic or Van der Waals interactions) or chemical (chemisorption, i.e., via formation of chemical bonds) sorption.^23^ Characteristic examples are intermetallic hydrides, where H_2_ is physically absorbed within the interstitial space in the solid, or complex hydrides where covalent/ionic interactions between H_2_ and adsorption cites drive the H_2_ storage process.^193^ Other families of relevant materials are the so-called Liquid Organic Hydrogen Carriers (LOHCs),^219^ 2D materials (e.g., graphene, borophene),^220–222^ and nanoporous materials such as MOFs and COFs (introduced in Section 2.2).

DOE has issued requirements for the design and production of new onboard automotive H_2_ storage systems.^197,199^ The most important requirements comprise H_2_ gravimetric capacities of 6.5 wt.% or higher, volumetric capacities of 0.05 kg H_2_/L or higher, and system costs of 266 *$*/kg H_2_ or cheaper.^197^ The adsorption energy of H_2_ with the material should optimally range from -0.20 to -0.40 eV/H_2_ to avoid under- or over-binding of the H_2_ molecules with the storage medium.^223–226^ A detailed description of the requirements including the minimum durability of the storage unit can be found in ref 197. It is noteworthy that no existing material has yet met all the requirements of DOE for onboard H_2_ storage. This is a major motivation, driving research in both academia and industry to discover and fabricate new materials and catalysts for H_2_ storage systems.^197^

Physisorption on porous materials such as MOFs and zeolites generally results in fast H_2_ release and capture kinetics, but suffers from low gravimetric and volumetric H_2_ capacities.^227,228^ Chemisorption on materials such as metal hydrides, reaches larger H_2_ capacities but the release of H_2_ (i.e., the dehydrogenation reaction) is energy intensive and has slow kinetics.^229,230^ To make H_2_ economy feasible, discovering and using suitable and cost-effective materials with high H_2_ capacities and fast release/capture kinetics are essential. 2D materials and porous structures, such as MOFs, are frequently studied for H_2_ storage and separation (see also Section 2.2.1) due to their high surface-to-volume ratio and chemical tunability, which allows for optimizing adsorption capacities and efficiencies (see Section 2.2.1). The number of possible new materials that can be potentially synthesized and used for H_2_ storage is virtually unlimited.^198^

Even when considering specific classes of nanoporous media, such as MOFs or zeolites, the number of materials that has been theoretically predicted to date reaches the order of mangnitudes of hundreds to millions. This combined with the rapid rate that new families of materials are being discovered, synthesized, and considered for H_2_ storage makes relying solely on experiments prohibitively costly and time-consuming.^231–235^ Also, experimental measurements of H_2_ systems require strict adherence to safety standards which involve protocols to prevent leaks, explosions, and contamination, especially at high-pressures.^197^ To this end, atomistic molecular simulations have emerged as a robust and efficient approach to *in silico* screen extensive families of materials with respect to important properties governing H_2_ storage. These properties include H_2_ adsorption energies, gravimetric and volumetric storage capacities, and diffusion coefficients of H_2_ in the materials.^51,236^ This allows for early detection of unsuitable materials, either due to low storage capacities or material instability, but also for identifying key-performing media which can be further studied experimentally. Material properties such as the Young’s modulus and thermal conductivity can also be predicted using molecular simulations^237,238^ and used to evaluate potential H_2_ storage systems. Considering the extensive body of literature on H_2_ storage in 2D and 3D materials, and given the fact that this topic closely aligns with Section 2.2.1, in this review we present only a condensed discussion of H_2_ sorption and storage in nanoporous media in Section 4.6. Focus is directed in the increasing coupling of AI techniques with classical molecular simulation to screen thousands of different materials Sections 4.6.1 and 4.6.2.

#### 2.3.3 Storage in Hydrates

While gas hydrates have been known for more than 200 years, it was a common belief until recently that pure H_2_ was not capable of stabilizing the hydrate structure due to the small size of the H_2_ molecule. A major change in the dominant belief occurred in 1997, when Kuhs et al.^239^ using neutron diffraction data of sII N_2_ hydrates demonstrated experimentally that multiple occupancy of a hydrate cage was possible. The important study of Kuhs et al. was followed by a number of experimental studies that confirmed the possibility of multiple occupancies for N_2_, O_2_ and Ar (for more details the reader is referred to the relevant experimental studies discussed in the review by Tsimpanogiannis and Economou^240^). A few years later, MC studies confirmed the capability of molecular simulations to capture multiple occupancy phenomena. A detailed review of the relevant MC studies is presented in ref 240.

While experimental evidence of the existence of H_2_ hydrates became available via a number of studies,^241–244^ the interest in H_2_ hydrates increased significantly only after Mao et al.^171^ reported experimental measurements confirming the formation of pure H_2_ hydrates at high pressures (i.e., 300 MPa at 280 K). At ambient pressure, pure H_2_ hydrate was found to be stable at low temperatures (i.e., 150 K). Mao et al. performed energy-dispersive X-ray diffraction (EDXD) and neutron diffraction experiments to identify that H_2_ hydrates are of type sII structure. Mao et al. also suggested that the 5^12^ (S-cages) could accommodate two H_2_ molecules, while the 5^12^6^4^ (L-cages) could accommodate up to four H_2_ molecules. Such a configuration allows for a H_2_ storage capacity equal to 5.0 wt.% H_2_ on hydrates. This value is close to the US DOE target of 6.5 wt% and a volumetric density of 62 kg H_2_/m^3^ for H_2_ storage in solid materials prior to 2001.^206^ Fig. 11 shows a schematic of sII H_2_ hydrate formation. Namely, when water and H_2_ are brought together at appropriate pressure and temperature conditions, sII hydrate forms. This hydrate consists of two different types of cages (i.e., S-, and L-cage) as shown in the top right panel of Fig. 11. The possibility of multiple H_2_ molecules occupying the L-cages (see bottom right panel of Fig. 11) results in a solid material with increased H_2_ storage capacity compared to the case when all types of cages are occupied (at most) by a single H_2_ molecule.

**Figure 11:**
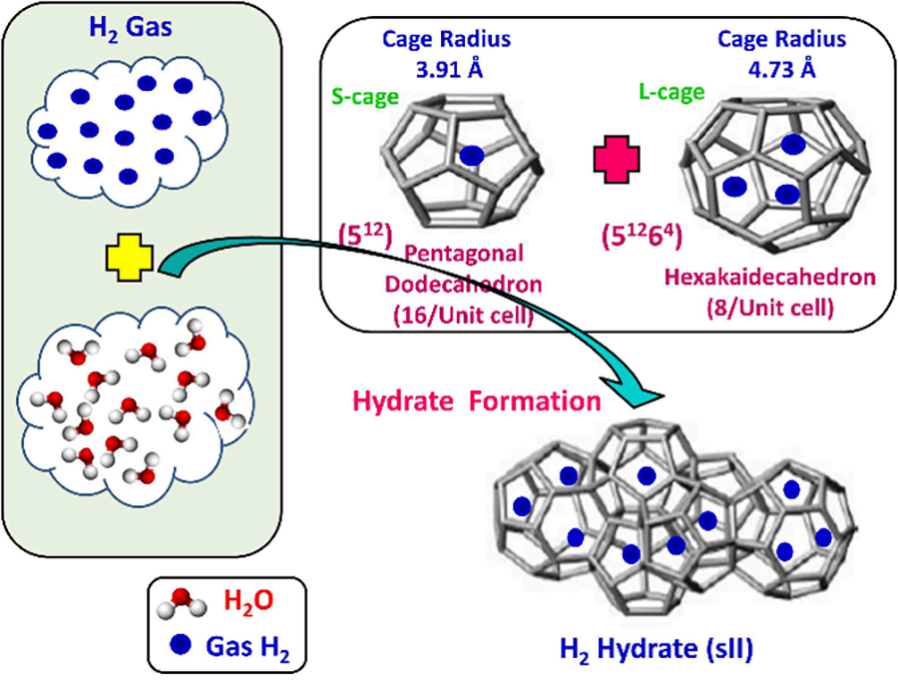
Schematic representation of H_2_ hydrate (sII) formation. Sixteen pentagonal dodecahedron (5^12^; S-cage) cages and eight hexacaidecahedron (5^12^6^4^; L-cage) cages combine in order to form a single unit cell of sII hydrate. The multiple occupancy of L-cages results in increasing the H_2_ storage capacity of the hydrate.

The high pressures (or the low temperatures) required for the formation of pure H_2_ hydrates is a major limitation for the use of hydrates as H_2_ storage materials for e.g., vehicular applications. Florusse et al.^245^ reported experiments demonstrating that the high pressures (≈ 2000 bar) required for the formation of pure H_2_ hydrates can be significantly reduced (e.g., 50 bar at 280 K) by using tetrahydrofuran (THF) as hydrate ”promoter” (i.e., a component that promotes hydrate formation at moderate pressure and temperature conditions compared to the pure case^246^). The use of a promoter, however, results in lower H_2_ storage capacity due to the promoter occupying the L-type cages (see Table 2 for cage notation). Lee et al.^247^ reported the possibility of increasing the H_2_ storage capacity of the mixed H_2_/THF hydrate by ”tuning” the hydrate. Namely, using less than the stoichiometric amount of THF, therefore succeeding in having partial occupancy of the L-cages by H_2_, instead of being occupied completely by THF. However, hydrate tuning has been highly debated in the literature due to the difficulty in experimentally reproducing the process (i.e., see the detailed discussions by Alavi and Ripmeester^177^ and Papadimitriou et al.^248^). Furthermore, an extensive discussion on the experimental and computational aspects of H_2_ storage in hydrates can be found in a series of review studies,^172,177,249–256^ while an excellent discussion on the statistical mechanics theory behind the thermodynamic stability of hydrates is provided by Tanaka and Matsumoto.^257^

From the discussion presented here it becomes evident that we are interested in identifying properties (thermodynamic, transport, and structural) and condition that affect the H_2_ storage capacity of pure or mixed H_2_ hydrates. Such are the following: (i) three-phase equilibrium conditions (i.e., higher pressures result in higher cage occupancies, therefore higher storage capacities, as a result of the fact that hydrates are not stoichiometric materials. Namely, not all cages are required to be occupied for hydrate stability), (ii) H_2_ diffusivity within the hydrate crystal (i.e., H_2_ can escape via diffusion from the hydrate solid structure, reducing the amount of H_2_ stored in the hydrate), and (iii) hydrate lattice constants (i.e., the use of various promoters, having different sizes, can result in increasing the lattice constant, therefore, providing increased storage capacity).

#### 2.3.4 Subsurface Storage

In 2021, the world energy consumption was estimated at 600 EJ.^258,259^ This massive energy requirement highlights the need for extensive H_2_ storage solutions. Given the limitations of surface storage facilities, e.g., holding the large volumes necessary for long-term energy storage, alternative solutions are explored. Geological formations, such as depleted reservoirs and salt caverns, offer storage capacities in the order of hundreds of gigawatt-hours, providing a feasible option for large-scale H_2_ storage.^259–262^

Underground H_2_ storage (UHS)^263,264^ (also referred to as subsurface H_2_ storage) involves injecting H_2_ into geological formations where interactions with the surrounding gases, fluids, and solids occur. A cartoon representation of UHS is shown in Fig. 12. This process can include the use of a so-called cushion gas,^259–262^ occupying up to 30% of the reservoir’s volume, to maintain a minimum pressure and ensure mechanical stability. Brine (usually a mixture of water and sodium chloride) acts as a natural sealant preventing H_2_ leakage into surrounding aquifers, maintaining the integrity and stability of the geological structure. Understanding the physical processes involved in subsurface H_2_ storage is crucial for optimizing storage strategies and ensuring safety and efficiency. In past years, molecular simulation has been proven to be a necessary tool for investigating these processes at the molecular level, providing detailed insights into several key properties and physical mechanisms, while accessing state points and quantities which can be inaccessible without costly and complex experimental setups. For example, H_2_ tends to form mixtures with other gases present in the reservoir, driven by molecular diffusion (see Fig. 12) and hydrodynamic dispersion^265^ caused by cyclic injection and production. The generalized Fick’s law describes this multi-component diffusion, relating the mass flux of each component to the concentration gradients of the others, governed by the mutual diffusion coefficient.^266,267^ These coefficients depend on thermodynamic state variables such as the pressure, temperature, and composition of the mixture. MD simulations can yield both accurate predictions of these coefficients, enhancing our understanding of the gas mixing behavior under various conditions relevant to subsurface H_2_ storage.

**Figure 12:**
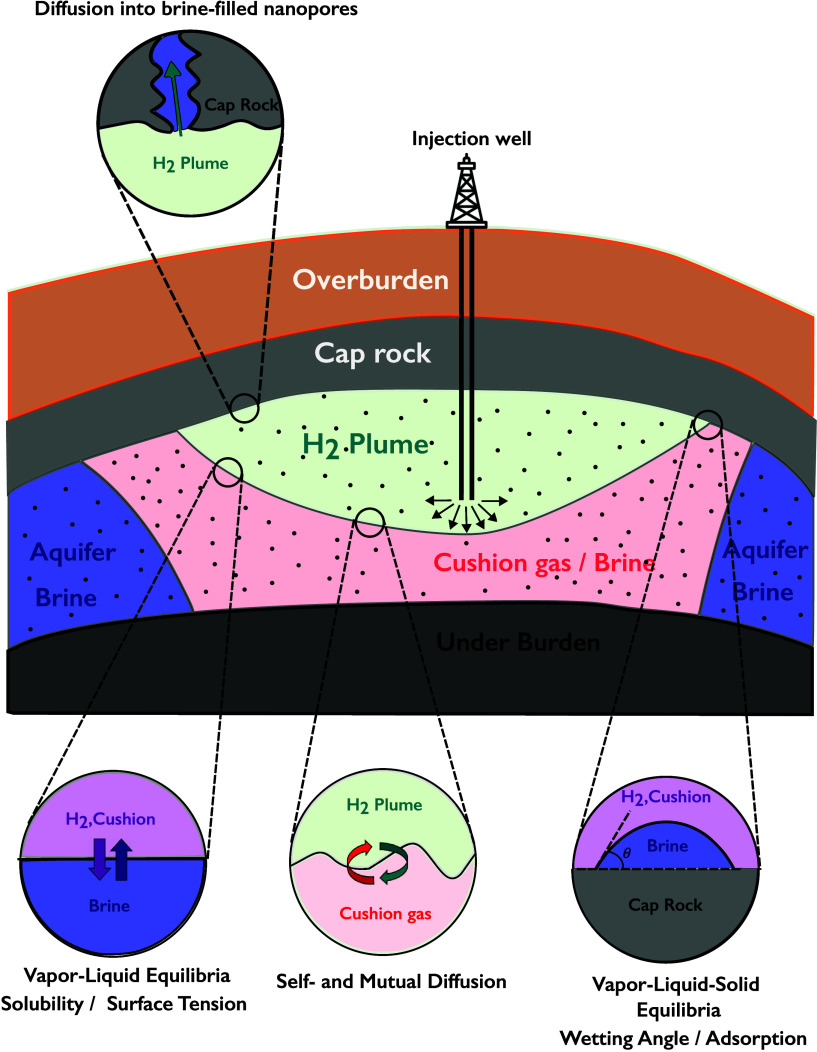
Schematic representation of key physical processes governing subsurface H_2_ storage. We highlight areas where molecular simulations are crucial, e.g., the diffusion of H_2_ into empty or brine-filled nanopores in the caprock, essential for assessing leakage risks, the dissolution of H_2_-cushion gas mixtures into brines under reservoir conditions, where atomistic modeling enhances our understanding, the mixing of H_2_ with cushion gas via molecular diffusion, potentially leading to contamination, and the three-phase contact angle between H_2_-cushion gas mixtures, brine, and geological solids, determining the capillary pressure needed for hydrogen to invade brine.

H_2_ and other gases in the reservoir interact with brine, leading to dissolution and exchange of molecules between the gas and liquid phases. The solubility of H_2_ in the brine, which depends on factors such as pressure, temperature, and salinity, determines the mole fraction of dissolved H_2_ and the moisture content in the gas phase at equilibrium (see Fig. 12). Accurate knowledge of these solubilities is critical for maintaining H_2_ purity, and optimizing storage conditions. Molecular simulations offer a detailed view of these interactions, allowing for precise predictions of solubilities and the overall phase behavior. The interfacial tension between H_2_ and brine significantly affects the stability and integrity of the storage system. The interfacial tension not only suppresses phase mixing, maintaining sharp gas-brine boundaries, and thus, reducing leakage,^259^ but also promotes capillary trapping, in which H_2_ becomes immobilized within brine-saturated pores on the grain scale. Although limiting phase mixing helps preserve the purity of stored H_2_, capillary trapping within brine-filled pores diminishes the recoverable volume and thus hampers the effectiveness of underground hydrogen storage (UHS).^261^ Because direct experimental measurements of such nanoscale forces are challenging, molecular simulations are indispensable for quantifying interfacial tension and elucidating the mechanisms of the H_2_ brine interaction, thus guiding the design of safer and more efficient storage strategies.

Another property, important for UHS, is the contact angle between H_2_, brine, and the solid geological matrix, which influences the wettability of the rock and the overall capillary pressure in the system (see Fig. 12). This, in turn, affects the storage capacity and retrievability of H_2_. Molecular simulations can be performed to predict the wetting behavior and contact angles in these systems, offering insights into how to modify surface properties to improve storage performance. While atomistic simulations provide useful insight into inter- and intra-molecular forces, and capture the fundamental physics of the wetting phenomena, they cannot always provide accurate quantitative predictions. Therefore, coupling molecular simulations with higher scale modeling methods is essential for studying, and ultimately, improve UHS performance. Key transport and displacement processes unfold over pore networks that extend from millimeters to centimeters and feature tortuous throats and irregular geometries, length scales far beyond the reach of molecular dynamics because of the prohibitively large number of molecules needed for the simulations. To describe flow and capillary behavior in such media, one must therefore couple the interfacial properties (e.g., surface tension, contact angle, diffusion coefficients) computed from molecular simulations to mesoscale or continuum models, such as pore-network and lattice-Boltzmann frameworks, volume-of-fluid CFD solvers, or hybrid multiscale schemes. UHS is thus inherently multiscale, which means that only by integrating molecular-level data into coarser-scale simulations one can predict injectivity, storage capacity and H_2_ retrievability relevant for UHS.

Understanding adsorption – the process by which gas molecules adhere to the surfaces of solids – is important for evaluating the feasibility and efficiency of UHS in geological formations. Adsorption can provide information related to the storage capacity, retention time, and potential leakage risks of H_2_ within these formations, essential for ensuring both efficiency and safety.^268^ Additionally, interactions between H_2_ and swelling clays at grain contacts, involving adsorption and desorption, can induce clay swelling.^261^ This swelling may lead to small normal and shear strains, resulting in stresses between individual grains. Over the lifespan of a H_2_ storage complex, such stresses could contribute to mechanical fatigue and permanent deformation of the reservoir, leading to induced seismicity.^261^ Therefore, sorption processes not only influence the retrievability of stored H_2_ but also play a significant role in the long-term stability and safety of the storage site. The complexity of the subsurface environment, with its variations in pressure, temperature, mineral compositions, and complex pore morphologies, makes predicting adsorption behavior of H_2_ an arduous task. Performing experimental measurements to study adsorption under these challenging conditions is fraught with practical challenges and risks, such as the high flammability of H_2_ and the difficulty of replicating complex subsurface conditions in the laboratory. Molecular simulations have emerged as a powerful tool to overcome these challenges. Molecular simulations can accurately model the interactions between H_2_ molecules and the diverse mineral surfaces found in geological formations, such as shale, quartz, and kerogen.^268^ By replicating subsurface conditions, molecular simulations help predict the adsorption capacity and behavior of H_2_, including its interaction with other gases like CH_4_ and CO_2_. The computed adsorption isotherms can then be used as input to reservoir-scale simulators to predict the transport of multi-component H_2_ mixtures, thus, helping in building predictive models for UHS.

From the short discussion above, it becomes apparent that molecular simulations are indispensable for advancing our understanding of subsurface H_2_ storage systems. By providing detailed insights into gas-gas mixing, vapor-liquid equilibrium, interfacial tensions, and wetting behaviors, molecular simulations enable the optimization of storage strategies, ensuring safe, efficient, and long-term H_2_ storage in geological formations. In an effort to build a sustainable energy future, taking full advantage of the power that molecular simulations offer will be crucial in overcoming the challenges associated with large-scale H_2_ storage. To facilitate navigation, Table 4 provides a summary of key properties relevant to UHS, indicating the sections where relevant computational methods are explained and where the discussion of related articles can be found in this review.

**Table 4:** Comprehensive List of Hydrogen Properties Relevant to Underground Storage (See Also Fig. 12), as Computed via MD Simulations^a^

Property	System	Condition
*pVT*	H_2_	323-423 K, 5-50 MPa, 0-1 $x_{H_{2}}$
	H_2_-CO_2_	323-423 K, 5-50 MPa, 0-1 $x_{H_{2}}$
	H_2_-CH_4_	310-470 K, 15–30 MPa, 0-1 $x_{H_{2}}$
	H_2_-CO_2_	310-470 K, 15–30 MPa, 0-1 $x_{H_{2}}$
Self- and mutual-diffusivity	H_2_-CO_2_	323-423 K, 5-50 MPa, 0-1 $x_{H_{2}}$
Thermodynamic factor	H_2_-CO_2_	323-423 K, 5-50 MPa, 0-1 $x_{H_{2}}$
Interfacial tension	H_2_-H_2_O	0.1–160 MPa, 298–523 K, 0–5 mol
	H_2_-NaCl Brine	0.1–60 MPa, 298–523 K, 0–5 mol
	H_2_-CaCl_2_ Brine	1–30 MPa, 298–373 K, 0–5 mol
	H_2_-MgCl_2_ Brine	1–30 MPa, 298–373 K, 0–5 mol
	H_2_-KCl Brine	1–30 MPa, 298–373 K, 0–5 mol
	H_2_-N_2_-H_2_O	1–70 MPa, 300–343 K
	H_2_-CH_4_-H_2_O	10–60 MPa, 323–388 K
	H_2_-CO_2_-NaCl Brine	5–40 MPa, 298–373 K, 0–3 mol
Wetting angle	H_2_-H_2_O-Mica	5 and 20 MPa, 300 and 323 K
	H_2_-H_2_O-Gypsum	5 and 20 MPa, 300 and 323 K
	H_2_-H_2_O-Anhydrite	5 and 20 MPa, 300 and 323 K
	H_2_-H_2_O-Halite	5 and 20 MPa, 300 and 323 K
	H_2_-H_2_O-Silica (Q2 and Q4)	5–60 MPa, 320 K
	H_2_-H_2_O-Silica (Q3/Q4)	1–160 MPa, 298–523 K
	H_2_-NaCl Brine-Quartz	20 MPa, 333 K, 1.5–3.5 mol
	H_2_-NaCl Brine-Silica (Q2)	0.1–150 MPa, 300–423 K, 0–5.4 mol
	H_2_-NaCl Brine-Humic Acid substrate (Q2)	0.1 MPa, 300 K, 0.5 mol
	H_2_-NaCl Brine-Siloxane with defects	10 MPa, 323 K, 20 wt%
	H_2_-NaCl Brine-Halite NaCl	5–25 MPa, 300–400 K, 0–1 mol
	H_2_-NaCl Brine-Quartz	5–25 MPa, 300–400 K, 0–1 mol
	H_2_-NaCl Brine-Calcite	5–25 MPa, 300–400 K, 0–1 mol
	H_2_-NaCl Brine-Montmorillonite	5–25 MPa, 300–400 K, 0–1 mol
	H_2_-KCl Brine-Silica (Q2)	14–150 MPa, 323–423 K, 0–5.4 mol
	H_2_/CO_2_-H_2_O-Calcite	0.1–70 MPa, 298–373 K
	H_2_/CO_2_-H_2_O-Quartz	0.1–70 MPa, 298–373 K
	H_2_/CO_2_-H_2_O-Silica	10–30 MPa, 333–413 K
	H_2_/CO_2_-H_2_O-Silica	10–30 MPa, 333–413 K
	H_2_/CO_2_-H_2_O-Graphene	5–40 MPa, 292–343 K
	H_2_/CH_4_-H_2_O-Calcite	0.1–70 MPa, 293–373 K
	H_2_/CH_4_-H_2_O-Quartz	0.1–70 MPa, 293–373 K
	H_2_/CH_4_-H_2_O-Silica	20 MPa, 373 K
	H_2_/CH_4_-H_2_O-Graphene	5–40 MPa, 292–343 K
	H_2_/N_2_-H_2_O-Silica	20 MPa, 373 K
	H_2_/CO_2_-NaCl Brine-Siloxane (Kaolinite)	5–80 MPa, 303–343 K, 1.7 mol
	H_2_/CO_2_-NaCl Brine-Talc	15 MPa, 333 K, 3.4 mol
	H_2_/CO_2_-KCl Brine-Talc	15 MPa, 333 K, 0.13 mol
	H_2_/CO_2_-NaCl Brine-Gibbsite (Kaolinite)	15 MPa, 333 K, 3.4 mol
	H_2_/CO_2_-KCl Brine-Gibbsite (Kaolinite)	15 MPa, 333 K, 0.13 mol
	H_2_/CH_4_-NaCl Brine-Siloxane (Kaolinite)	5–80 MPa, 303–343 K, 1.7 mol
	H_2_/CH_4_-NaCl Brine-Talc	15 MPa, 333 K, 3.4 mol
	H_2_/CH_4_-KCl Brine-Talc	15 MPa, 333 K, 0.13 mol
	H_2_/CH_4_-NaCl Brine-Gibbsite (Kaolinite)	15 MPa, 333 K, 3.4 mol
	H_2_/CH_4_-KCl Brine-Gibbsite (Kaolinite)	15 MPa, 333 K, 0.13 mol
Adsorption on clay-substrates	CH_4_/H_2_/H_2_O-Na-Montmorillonite	343 K / -
	H_2_-Kaolinite (Gibbsite and Siloxane)	300 K / 1.0–50.7 MPa
	H_2_/CH_4_-Kerogen	338 K / 0–27.6 MPa
	H_2_/CH_4_-Montmorillonite	323 K / up to 11.15 MPa
	H_2_/CH_4_/H_2_O-Montmorillonite	323 K / up to 11.15 MPa
	H_2_/H_2_O-Kaolinite (Gibbsite and Siloxane)	298.15 K / 0.1–10 MPa
	H_2_/CH_4_-Hydroxylated Quartz	323.15–423.15 K / 1–50 MPa
	H_2_/CO_2_-Hydroxylated Quartz	323.15–423.15 K / 1–50 MPa
	H_2_/H_2_O-Hydroxylated Quartz	323.15–423.15 K / 1–50 MPa
	H_2_-Kaolinite	303.15–353.15 K / 0–18 MPa
	H_2_-Illite	303.15–353.15 K / 0–18 MPa
	H_2_-Dolomite	303.15–353.15 K / 0–18 MPa
	H_2_-Quartz	303.15–353.15 K / 0–18 MPa
	H_2_-Calcite	303.15–353.15 K / 0–18 MPa
	H_2_-SiO_2_	300 K / 10–30 MPa
	H_2_/CH_4_-SiO_2_	300 K / 10–30 MPa
	H_2_/H_2_O-SiO_2_	300 K / 10–30 MPa
	H_2_/CH_4_/H_2_O-SiO_2_	300 K / 10–30 MPa
	H_2_/CH_4_/H_2_O-Kaolinite	333.15 K / 30 MPa
	H_2_/CO_2_/H_2_O-Kaolinite	333.15 K / 30 MPa
	H_2_/CH_4_-Montmorillonite	333 K / 15 MPa
	H_2_/CO_2_-Montmorillonite	333 K / 15 MPa
	H_2_/CH_4_-Illite	333 K / 15 MPa
	H_2_/CO_2_-Illite	333 K / 15 MPa
	H_2_/H_2_O-Kaolinite (Gibbsite and Siloxane)	298 K / 10 and 20 MPa
	H_2_-Calcite	298–350 K / 1–10 MPa
	H_2_-Hematite	298–350 K / 1–10 MPa
	H_2_-Quartz	298–350 K / 1–10 MPa
	H_2_/CH_4_-Kaolinite	333.15 K / 30–15–10 MPa
	H_2_/CH_4_-Kerogen	333.15 K / 30–15–10 MPa
	H_2_-Kerogen II-D	333.15 K / 3–18 MPa
	H_2_/CH_4_-Montmorillonite	333.15 K / 3–18 MPa
	H_2_/CO_2_-Montmorillonite	333.15 K / 3–18 MPa
	H_2_-Kaolinite	323–403 K / 1–50 MPa
	H_2_/CH_4_-Kaolinite	323–403 K / 1–50 MPa
	H_2_/CO_2_-Kaolinite	323–403 K / 1–50 MPa
	H_2_/CH_4_-Quartz	339–400 K / 1–50 MPa
	H_2_/CH_4_/H_2_O-Quartz	339–400 K / 1–50 MPa
	H_2_-Kaolinite (Gibbsite and Siloxane)	303–423 K / 1–30 MPa
	H_2_/H_2_O-Kaolinite	318.15 K / 0.1–30 MPa
	CH_4_/H_2_O-Kaolinite	318.15 K / 0.1–30 MPa
	H_2_/CH_4_/H_2_O-Kaolinite	318.15 K / 0.1–30 MPa
	H_2_/H_2_O-Pyrophyllite	300 K / 0.1 MPa
	CO_2_/H_2_O-Pyrophyllite	300 K / 0.1 MPa
	H_2_/H_2_O-Gibbsite	300 K / 0.1 MPa
	CO_2_/H_2_O-Gibbsite	300 K / 0.1 MPa
	H_2_-Kerogen	363.15 K / 0.1–50 MPa
	CH_4_-Kerogen	363.15 K / 0.1–50 MPa
	H_2_/CH_4_-Kerogen	363.15 K / 0.1–50 MPa
	H_2_-Na-Montmorillonite	298–403 K / 1–50 MPa
	H_2_/CO_2_/H_2_O-Na-Montmorillonite	298–403 K / 1–50 MPa
	H_2_/CH_4_/H_2_O-Na-Montmorillonite	298–403 K / 1–50 MPa
	H_2_/CO_2_/Octane-Kaolinite (Gibbsite and Siloxane)	300 K / 5.1–30.4 MPa
	H_2_/OMCTS-MCM-41 Silica	298 K / 0–1 MPa
	CO_2_/OMCTS-MCM-41 Silica	298 K / 0–1 MPa
	H_2_-Montmorillonite	333–413 K / 2–30 MPa
	H_2_/H_2_O-Montmorillonite	333–413 K / 2–30 MPa
	H_2_/H_2_O-Kaolinite	310–410 K / 30 MPa
	H_2_/NaCl Brine-Kaolinite	310–410 K / 30 MPa
	H_2_/MgCl_2_ Brine-Kaolinite	310–410 K / 30 MPa
	H_2_-Calcium Silicate Hydrate	300–370 K / 2.5–40 MPa
	H_2_-Kerogen	360 K / 3–41 MPa
	H_2_/CH_4_/H_2_O-Illite	326 K / 30.4 MPa
	H_2_/CH_4_/CO_2_/H_2_O-Illite	326 K / 30.4 MPa
	H_2_/CH_4_/H_2_O-Kerogen	326 K / 30.4 MPa
	H_2_/CH_4_/CO_2_/H_2_O-Kerogen	326 K / 30.4 MPa
	H_2_/H_2_O-Pyrophyllite	368.15 K / 30 MPa
	H_2_/H_2_O-Montmorillonite	368.15 K / 30 MPa
	H_2_/H_2_O-Beidellite	368.15 K / 30 MPa
	H_2_/H_2_O-Calcite	353 K / 20 MPa

a For each property, the table contains the specific systems investigated and the conditions of the studies. The relevant methods and in-depth discussions per property can be found in this review in the following sections: *pVT*: Sections 3.2 and 3.3 (methods), Section 4.2 (discussion). Diffusivities and thermodynamic factors: Sections 3.2.3, 3.2.4, and 3.2.13 (methods), Section 4.2.5 (discussion). Interfacial tension: Section 3.2.16 (methods) and Section 4.9 (discussion). Wetting: Section 3.2.17 (methods) and Section 4.10 (discussion). Adsorption on clays: Section 3.3.2 (methods) and Section 4.7 (discussion).

### 2.4 Hydrogen Transport

#### 2.4.1 Transport in Polymer Pipelines

H_2_ economy presents new opportunities and challenges for pipeline systems. Currently, H_2_ is mainly transported in compressed pressure vessels, tube trailers, and pipelines. Pipeline transport offers advantages due to high capacity, extensive inter-connectivity, geographic coverage, and relatively low operational costs. While over 5,000 km of H_2_ pipelines exist globally, these are primarily constructed of various steel grades that face significant challenges from hydrogen embrittlement as discussed in more detail in Section 2.4.2. For this reason, polymer pipelines are considered a plausible solution for the transport of gaseous H_2_ due to intrinsic advantages over traditional metallic infrastructures, such as corrosion resistance, flexibility, cost-effectiveness, and the ability to form robust joints through fusion techniques.^269^ Among the numerous available polymeric materials, PE (and HDPE) is the predominant choice for gas distribution networks, comprising 90-95% of new installations in Europe and the United States.^270^ Despite their advantages, polymer pipelines face distinct challenges when transporting H_2_. The most important drawback is the enhanced permeation rate of H_2_ molecules through the polymer matrix, which can be up to five times higher than that of CH_4_. The permeability is also heavily affected by the temperature and pressure conditions. Other disadvantages are the so-called blistering risk (i.e., H_2_ absorbed by the polymer liner at high pressure cannot escape quickly enough during decompression, causing internal pressure buildup that creates blisters on the material), and various other possible material failure mechanisms which are outside the context of this review.^271^ The semi-crystalline structure of PE consists of long polymeric chains with varying lengths and side branches. Pipeline performance is primarily determined by the base resin density, molecular weight of these chains, and their distribution. The viscoelastic properties of PE create complex creep and stress relaxation behaviors that make short-term tests inadequate for predicting long-term performance.

As with all aspects of H_2_ technology discussed thus far, the safe and effective transport of H_2_ through polymer pipelines requires deep understanding of material properties, manufacturing processes, and operational conditions. With the plans to expand H_2_ infrastructure, developing polymer systems with enhanced barrier properties will be crucial for maintaining integrity and safety in transportation networks. Toward this goal, molecular simulation has become a widely used tool for studying H_2_/polymer systems, as also discussed in Sections 2.2.1 and 2.3.1. As expected, the main body of the relevant molecular simulation literature focuses on PE/HDPE, which are the most adequate materials for pipelines. Nevertheless, H_2_ interactions with other polymers such as ethylene-vinyl alcohol copolymer (EVOH) (a promising alternative to HDPE), poly­(dimethylsiloxane) (PDMS), poly­(chloro-p-xylene), polystyrene (PS), rubbery matrices, and polysaccharides have been studied via molecular simulation in a broader context spanning H_2_ applications beyond transport in pipelines. The vast majority of these studies focus on the same properties (i.e., sorption, diffusion, and permeation), make use of similar methods (MC for computing sorption and MD for gas transport coefficients - see Section 3) and force fields (see Section 3.1.4). A representative selection of molecular simulation studies in this field is reviewed in Section 4.4.

#### 2.4.2 Hydrogen Embrittlement of Metallic Pipelines

Numerous efforts for designing metallic pipelines such that they are compatible with H_2_ are made by both the industry and scientific community. This is not a simple engineering task since the presence of atomic hydrogen (H) in metals (e.g., in steels used in existing natural gas pipelines) results in the deterioration of mechanical properties, such as strength and deformability. This phenomenon is known as hydrogen embrittlement (HE)^21,272–277^ and can occur even at extremely low H_2_ concentrations (in the order of 1 ppm). Therefore, HE poses a significant challenge to the safe transportation of gaseous H_2_ via pipelines. The complexity behind understanding the fundamental mechanisms of HE lies in the fact that it requires an accurate description of the atomic H trapping and diffusion in the presence of different kinds of microstructural features. The diffusivity study, in particular, is crucial as the fact that the diffusible H causes embrittlement, is well established.^278,279^ The minute amount of H present in a material, is attracted towards stress fields e.g., front of crack tip where it concentrates causing brittle fracture. Diffusion measurements e.g., permeation tests and Thermal Desorption Analysis (TDA),^280^ have indicated that diffusible H can be trapped in microstructural features e.g., grain boundaries (GBs) and carbides. There is experimental evidence^281^ that a material in service does not suffer from HE if the diffusible H can be controlled. This observation forms the basis of designing alloys with irreversible traps for immobilizing diffusible H.^281–283^ Another effective way to reduce the amount of diffusible H in the material is by the usage of coatings, such as oxides and metallic alloys, which act as barriers against H thereby reducing the H uptake by the material.^283^ The Slow Strain Rate Test (SSRT) performed within recent works^31,284^ has shown that the stress-strain curves of H charged coated sample is similar to that of uncharged bare sample of X70 pipe steel. Most of the experimental techniques employed in the investigation of H in materials operate at the macro-scale and hence lack spatial resolution, thus, making the determination of H trapping sites difficult, especially when H is trapped in nano-sized precipitates. In this regard, Atom Probe Tomography (APT), which allows high spatial resolution, proves to be a reliable tool for nano-scale visualization of H distribution in steels.^285–288^ Another useful experimental technique in this regard is the Small Angle Neutron Scattering (SANS).^289,290^ The individual underlying mechanisms are, however, still hard to elucidate experimentally since the complexity of the steels implies combined effects.

HE in metallic pipelines is a multi-disciplinary phenomenon, involving chemical processes and mechanical deformation across a wide range of spatial and temporal scales.^291^ At the chemical level, HE is initiated with H_2_ adsorption and dissociation on metal surfaces, followed by atomic H diffusion into bulk lattices, vacancies, dislocation cores, and GBs. All involved processes, i.e., adsorption, absorption, desorption, and lattice diffusion, are governed by distinct thermodynamic and kinetic parameters, which strongly depend on the local microstructure. Concurrently, mechanical deformation involving crack propagation, GB decohesion, dislocation nucleation and motion, and vacancy formation interacts continuously with H diffusion, leading to coupled chemo-mechanical degradation.^292^ Capturing these phenomena demands a hierarchical, multi-scale modeling approach: DFT simulations provide atomically accurate energetics and kinetics of H behavior at the sub-nanometer scale; MD simulations, with classical interatomic potentials, enable insights into defect-driven deformation processes at the nano-meter and nano-second scales; and Kinetic Monte Carlo (KMC) methods allow for the study of H diffusion and trapping processes over significantly longer, time-scales (i.e., milliseconds to seconds). Bridging these methodologies offers a powerful and comprehensive framework to unravel the fundamental mechanisms underlying HE, enabling more accurate predictions and effective mitigation strategies for metallic structural components in H-rich environments. Table 5 summarises the key properties investigated in HE, and the metallic systems considered, while referring the reader to the sections where the relevant methods and discussion are presented in this review.

**Table 5:** Thermophysical and Mechanical Properties Investigated in Relation to the Mechanisms Governing Hydrogen Embrittlement of Metals^a^

		Relevant sections	
Properties	Materials	Methods	Discussion
H diffusion	bcc Fe; fcc Ni; bcc MoNbTaW; fcc FeMnCrCoNi	3.2.7, 3.2.9, and 3.3.5	4.11.1, 4.11.6, and 4.11.7
H trapping and H-mediated vacancy behavior	Carbides; oxides; bcc Fe; fcc Cu and Pd; bcc MoNbTaW; fcc CrCoNi, FeMnCrCoNi	3.2.7 and 3.2.8	4.11.2, 4.11.6, and 4.11.7
Dislocation nucleation and mobility	fcc Ag, Ni, Pd; bcc Fe; fcc FeNiCr and FeMnCrCoNi	3.2.10 and 3.3.5	4.11.3, 4.11.6, and 4.11.7
Crack propagation and GB decohesion	fcc Ni; bcc Fe; fcc CrCoNi, CoNiV, CoCrFeNi and FeMnCrCoNi	3.2.7, 3.2.11, and 3.3.5	4.11.4 and 4.11.6
Complex deformation behavior	fcc Ni; bcc Fe; hcp Mg	3.2.7 and 3.3.5	4.11.5

a The sections that detail the methods and the subsequent discussion are indicated.^292^

#### 2.4.3 Liquid Hydrogen

As already discussed earlier in this review, the implementation of green H_2_ is currently seriously hindered by the challenges related to transport and storage. Gaseous H_2_ has an extremely low volumetric energy density, which can be increased if H_2_ is liquefied. However, liquefaction is an expensive process that normally requires very low temperatures (ca. 20 K). The specific liquefaction energy is roughly equal to 15 kWh/kg, which corresponds to 1.5 Euros/kg if a realistic electricity price of 0.1 Euros/kWh is used.^293^ The high energy consumption is a consequence of the low liquefaction temperatures that can only be attained by complex refrigeration cycles, and the unique behavior of H_2_ at very low temperatures (the so-called quantum effects).^294^ The molecules of H_2_ can exist in two forms depending on the spins on the two H nuclei. If both spins are symmetric, i.e., in the same direction, the molecule is called ortho-H_2_ (o-H_2_) and when the spins are anti-symmetric, the molecule is referred to as para-H_2_ (p-H_2_). o-H_2_ molecules have a higher energy state than the p-H_2_ molecules. H_2_ at room temperature contains 75% o-H2 and 25% p-H2, but the para-fraction is increased to almost 100% as the temperature is reduced to the liquefaction temperature of ca. 20 K. Due to the difference in the energy state of the spins, the ortho-to-para conversion is highly exothermic and releases a huge amount of heat during liquefaction. The released heat should be removed, which increases the cooling demand (energy requirement) of the refrigeration cycle. However, the heat release is not linear but increases exponentially with decreasing temperature. An interesting conclusion from this physics exercise is that it would be beneficial if H_2_ could be liquefied at higher temperatures, because that would reduce the cooling demand as most of the energy is consumed at the lower temperatures (due to increased heat release). This sounds almost impossible, because the liquefaction of H_2_ is governed by its pressure-temperature diagram (saturation line), which states that H_2_ can be at the liquid state only in a temperature range of 14 K (triple point) to 33 K (critical point). Interestingly, a molecular simulation study in 2005 by Han et al.^295^ showed that H_2_ can be liquefied at 80 K if the liquefaction is performed under confinement in a carbon nanotube (CNT). This phenomenon was attributed to the strong electrostatic interaction between the CNT and H_2_ molecules, which induce local-ordering of the H_2_ molecules and results in liquefaction. Note that such a finding does not necessarily violate thermodynamics, because the previously mentioned liquefaction temperature range of 14 to 33 K holds for bulk systems, which may be different for systems under confinement. However, the understanding of H_2_ liquefaction under confinement is poorly studied and understood. To the best of our knowledge, no other theoretical or experimental studies to date were reported to verify the results of Han et al.^295^ This showcases the power of molecular simulation not only as a predictive tool, complementary to experiments, but also as a vehicle for exploring new H_2_ technologies. More details on this and a few other studies related to molecular simulation of liquid H_2_ are provided in Section 4.3.

## 3 Molecular Simulation Methods for Computing Thermophysical Properties of Hydrogen Systems

In this section, we provide an overview of the most widely used methods and models for computing thermophysical properties of H_2_ systems. Although a lot of ground related to thermodynamic, transport, interfacial, and structural properties, as well as physical mechanisms and force fields is covered here, a detailed discussion on statistical mechanics fundamentals, derivations of equations, and a full list of possible methods are out of the scope of this review. For such information, the reader is referred to relevant textbooks in the field of molecular simulation.^51,52,296–299^

### 3.1 Force Fields

To a great extent, the accuracy of classical molecular simulations, both MD and MC, relies on semi-empirical models, the so-called force fields, which describe the intra- and inter-molecular interactions between the atoms and molecules in the system. Thus, it is not surprising that rigorous effort has been put during the past five decades by numerous researchers to design “high-performance” force fields with varying complexity for almost all types of chemical species. “High-performance” entails that a force field is able to accurately reproduce as many experimentally measured thermophysical properties of a species as possible. Such a performance is usually tied to the strategy followed during the parameterization of the force field, e.g., the number and type of properties considered, the range of conditions, the functional forms used to approximate inter- and intra-molecular forces.

The main focus of force fields for important components, such as water, hydrocarbons, electrolytes, and bio-molecules, has been the replication of phase behavior, volumetric properties, structure, transport coefficients, and energies.^56,300–307^ It is important to note that due to the fact that force fields are primarily fitted to experimental data, the abundance and accuracy of the available experimental data are crucial elements for accurate molecular simulations. Complementary, the significant attention put in performing MC and MD simulations for the prediction of many H_2_ properties is a motivation for further experimental effort. In this review, we provide a brief discussion on force fields commonly used to perform molecular simulations of H_2_ systems. The main focus is on the H_2_ force fields, pure or in aqueous electrolyte solutions (important for electrochemical processes), systems involving H_2_ and solid surfaces (important for storage), and organic components (important for separation applications).

Since this review paper mainly focuses on classical force fields, it is important to delineate the conditions under which quantum effects become significant and lead to noticeable deviations in the prediction of thermophysical properties using classical molecular simulations. The criteria discussed below serve as indicators for the onset of quantum mechanical contributions that should not be neglected. A commonly used criterion is based on the comparison between the thermal de Broglie wavelength and the characteristic interparticle spacing, approximated by mean nearest neighbor separation, ${\left(V/N\right)}^{\frac{1}{3}}$ .^308^ The de Broglie wavelength is given by

1 $$\Lambda ={\left(\frac{h^{2}}{2\pi mk_{B}T}\right)}^{1/2}$$

where *h* is Planck’s constant, *m* is the particle mass, *k*_B_ is the Boltzmann constant, and *T* is the absolute temperature. Bartolomeu and Franco^308^ report representative values for the ratio $\Lambda /{\left(\frac{V}{N}\right)}^{\frac{1}{3}}$ as 0.36 at 100 K and 1000 bar, and 0.016 at 400 K and 1 bar. For liquid H_2_, this ratio approaches 0.97, indicating that quantum effects are substantial and should be explicitly accounted for. A second criterion involves the comparison between the rotational temperature Θ_rot_ of molecular H_2_ and the simulation temperature. Quantum effects in rotational degrees of freedom become significant when Θ_rot_ is comparable to or greater than the system temperature.^308^ For molecular hydrogen, the rotational temperature is ca. 86 K, and is defined as

2 $$\Theta_{\mathrm{rot}}=\frac{h^{2}}{8\pi^{2}Ik_{B}}$$

where *I* is the moment of inertia of the molecule. If either of these criteria is fulfilled, classical simulations may yield inaccurate results. A way to mitigate this drawback is using the so-called quantum corrections (discussed in Section 3.1.1).

#### 3.1.1 Hydrogen

Many different classical force fields have been developed for H_2_, differing on the experimental/ab-initio parameters they are trained on, the functional form, and the number of interaction sites.^48,309–314^ While the discussion we are presenting here does not aim to be exhaustive, it covers several of the most frequently used H_2_ models in molecular simulation studies. Our goal is to highlight key parameter choices, point out inconsistencies and ambiguities that have emerged in the literature, and identify points of attention for researchers aiming to ensure consistency and reproducibility in the application of H_2_ force fields. It is important to acknowledge the detailed assessment by Barraco et al.^309^, who systematically compared eight Lennard-Jones (LJ)–based models in the gas phase, at conditions relevant to H_2_ storage in tanks. To ensure cohesion and provide a self–contained overview, we also summarize relevant aspects of their findings in this review.

In classical simulations, the most popular functional form describing the pairwise attractive (van der Waals) and repulsive (Pauli’s exclusion) forces is the 12-6 LJ potential (*U*_LJ_)^51^

3 $$U_{\mathrm{LJ}}=4\epsilon_{ij}\left[{\left(\frac{\sigma_{ij}}{r_{ij}}\right)}^{12}-{\left(\frac{\sigma_{ij}}{r_{ij}}\right)}^{6}\right]$$

where *ϵ_ij_* and *σ_ij_* are the so-called energy and size parameters, respectively, and *r_ij_* is the distance between species *i* and *j*. Although most H_2_ force fields are based on the 12-6 LJ potential, the 9-6 LJ form has also been used e.g., in the IFF (9-6) force field^313^ and the COMPASS force field of Yang et al.^315^, Sun^316^. H_2_ force fields using the 9-6 LJ potential have larger *σ_ij_* values than the 12-6 LJ to compensate for the reduced repulsive interactions.^309^ The LJ potential can be considered as a special case of the more general Mie potential:

4 $$U(r)=\frac{n}{n-m}{\left(\frac{n}{m}\right)}^{\frac{m}{n-m}}\times \varepsilon \left[{\left(\frac{\sigma_{ij}}{r_{ij}}\right)}^{n}-{\left(\frac{\sigma_{ij}}{r_{ij}}\right)}^{m}\right]$$

By setting *n* = 12 and *m* = 6, one recovers the familiar 12-6 LJ potential, while *n* = 9 and *m* = 6 yields the softer-repulsion 9-6 LJ variant. Bartolomeu and Franco^308^ proposed a single-site Mie potential, known as the SAFT-*γ* Mie force field, using parameters previously optimized within the SAFT-VR Mie EoS framework.

To account for the electrostatic interactions, the most common approximation in H_2_ force fields is Coulomb’s law^51^

$$U_{\mathrm{Coul}}=\frac{q_{i}q_{j}}{4\pi \epsilon_{0}{r_{ij}}^{2}}$$ 5

where *q_i_* is the charge of species *i*, and *ϵ*_0_ is the electric constant. Force fields that incorporate charges can be classified in two different categories, i.e., non-polarizable and polarizable. In non-polarizable force fields, H_2_ is modelled using fixed point charges. Polarizable force fields account for the change in the charge distribution of a molecule depending on the environment.^314^ Most of the H_2_ models to date are non-polarizable.^309–311,313^ For models with no intramolecular degrees of freedom, the total potential energy of the system follows from the summation of Eq. ([Disp-formula eq3]) and Eq. ([Disp-formula eq5]).^51,52^ The combination of LJ and Coulombic potentials is not always sufficient to describe strong adsorption interactions of H_2_ with materials.^317^ In such cases, instead of the LJ, the Morse potential is used^318^

6 $$U_{\mathrm{Morse}}=D_{ij}\left(1-\exp[-\alpha (r_{ij}-r_{ij}^{*})]\right)$$

where *D_ij_* and *r*_ij_^*^ are the interaction and size parameters (analogous to *ϵ_ij_* and *σ_ij_* for the LJ potential), respectively. Parameter *α* controls the width of the potential well. This extra fitting parameter of the Morse potential allows for better reproducibility of H_2_ adsorption onto materials with exposed metal centers, such as MOFs.^317^ Another functional form of intermolecular potentials of H_2_ is the so-called Exp-6 (Buckingham). Although such models have been already derived more than 70 years ago (see for example the work of Mason and Rice^319^), they are scarcely used in molecular simulations of H_2_ systems, and therefore, we do not discuss them further.

Fig. 13 shows a schematic representation of single-, two-, and three-site H_2_ force fields. In single-site force fields, H_2_ is assumed to have only one effective interaction site (united atom approach), which interacts with the surrounding molecules using only two-body attractive and repulsive forces.^309,320^ The model by Vrabec and co-workers^48^ (also referred to as Köster), with *ϵ*/*k*_B_ = 25.84 K and *σ* = 3.0366 Å, was fitted to reproduce speed of sound and *PVT* data in the 50-250 K range. In contrast, the potential by Buch^312^ focuses on capturing orientational effects of H_2_ at low temperatures (parameters: *ϵ*/*k*_B_ = 34.2 K and *σ* = 2.96 Å). Hirschfelder et al.^321^ proposed multiple sets of LJ parameters based on different datasets and methodologies: fitting to classical viscosity data yields either *ϵ*/*k*_B_ = 33.3 K, *σ* = 2.968 Å or *ϵ*/*k*_B_ = 38.0 K, *σ* = 2.915 Å, while fitting to second virial coefficients produces *ϵ*/*k*_B_ = 37.0 K, *σ* = 2.928 Å (quantum-based) and *ϵ*/*k*_B_ = 29.2 K, *σ* = 2.87 Å (classical). The authors recommend using the viscosity-based fits for transport property calculations, and the virial-based parameters for thermodynamic modeling. In their review, Barraco et al.^309^ refer to the parameter set *ϵ*/*k*_B_ = 37.0 K and *σ* = 2.928 Å, derived from fits to the second virial coefficients. Rzepka et al.^322^ and Ferrando and Ungerer^323^ used the classically derived parameters from Hirschfelder’s viscosity-based model, i.e., *ϵ*/*k*_B_ = 33.3K and *σ* = 2.97 Å. Similarly, Caviedes and Cabria^324^ adopted this parameter set in their study of H_2_ storage capacities in slit-shaped pores, nanotubes, and torusenes. In contrast, Rahbari et al.^110^ used the alternative viscosity-based parameterization by Hirschfelder, namely *ϵ*/*k*_B_ = 38.0 K and *σ* = 2.915 Å. The specific rationale for selecting among the four parameter sets proposed by Hirschfelder is not necessarily explicitly addressed in these studies; however, in most cases, the choice appears to align with the focus and requirements of the respective application.

**Figure 13:**
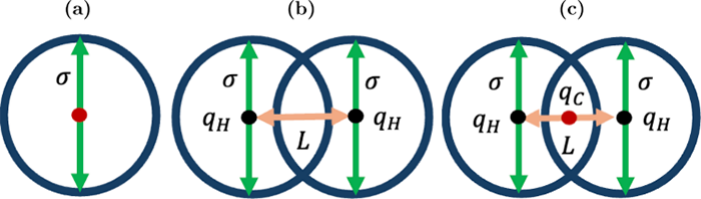
Schematic representations of (a) single-site, (b) two-site, and (c) three-site LJ-based H_2_ force fields. The green arrows represent the *σ* value of the LJ potential, the pink arrows represent the bond distance (*L*) of H_2_, and *q*_H_ and *q*_C_ represent the charges on the H-atom and the center of mass of H_2_, respectively. The force fields developed by Buch,^312^ Köster et al.,^48^ and Hirschfelder et al.^321^ are examples of one-site force fields, in which the LJ site is located at the center of mass of H_2_. The force fields developed by Cracknell^311^ and Wang et al.^313^ are examples of two-site force fields, in which the LJ sites are separated by a bond length *L*. The force fields by Cracknell^311^ and Wang et al.^313^ are non-polarizable and do not use charges (i.e., *q*_H_ = 0). The force field by Marx and Nielaba^310^ is an example of a three-site H_2_ force field, which accounts for the quadruple moment of H_2_. Three-site LJ force fields can have up to three different LJ sites. This figure is based on the work of Barraco et al.^309^

Two-site force fields, such as the IFF force field by Wang et al.^313^, provide a more realistic representation of the structure of H_2_ by including explicit interaction sites for each atom. This also allows for the inclusion of intra-molecular interactions, often represented by harmonic potentials, which capture the H-H bond vibrations. These vibrations are particularly important for accurately predicting spectroscopic properties such as Raman and IR spectra. However, this approach, due to the high-frequency nature of vibrational modes, requires the use of smaller MD integration time steps (typically <1 fs) to maintain numerical stability due to the high-frequency nature of vibrational modes. Despite their importance for vibration-dependent properties, the use of two-sites in the H_2_ force fields has limited impact on the prediction of bulk thermodynamic and transport properties, such as self-diffusivities, *pVT* relations, and physisorption of H_2_, which are dominated by slower translational and rotational motions.^311^ For many practical applications, the additional computational cost of resolving vibrations is unnecessary, and the use of simpler, rigid models (i.e., with a fixed bond length) is sufficient. Nevertheless, several two-site inter-molecular potentials have been developed to describe molecular H_2_ based on different calibration targets. Yang and Zhong^325^ proposed a H_2_ model using parameters *ϵ*/*k*_B_ = 10 K and *σ* = 2.72 Å, with a fixed bond length of 0.74 Å. This model was calibrated using experimental *PVT* data and self-diffusion coefficients of H_2_ over a temperature range of 80–300 K and pressures up to 600 bar. Cracknell^311^ proposed a two-site intermolecular potential for H_2_ with *ϵ*/*k*_B_ = 12.5 K, *σ* = 2.59 Å, and a fixed bond length of 0.74 Å. The LJ parameters were adjusted to reproduce H_2_ adsorption behavior in graphitic slit pores, specifically targeting agreement with isosteric heats and adsorption isotherms obtained from earlier Path Integral Monte Carlo (PIMC) simulations.

The following models extend the two-site description of H_2_ by incorporating electrostatic interactions via embedded quadrupoles, most commonly implemented through point-charge arrangements rather than explicit multipole sites. Most of the molecular simulation software packages do not support point multipole sites,^326^ and thus, point charges are often preferred. Dipole moments can, in principle, be represented by a pair of closely spaced point charges (+*q*, – *q*), yielding a dipole moment *μ_d_* = *qd*. Similarly, three point charges (+*q*, – 2*q*, + *q*) can be arranged to reproduce a quadrupole moment of the form *Q* = 2*qd* ^2^. Either the distance *d* or the charge *q* may be specified, and the choice of parameter is non-trivial.^326^ In this review, most of the surveyed studies use point-charge models. In the case of the force field developed by Marx and Nielaba^310^, some confusion has arisen regarding the specific charges used to reproduce the quadrupole moment, as noted by Barraco et al.^309^ The parameters reported by Marx and Nielaba^310^ are *σ* = 2.958 Å, *ϵ*/*k*_B_ = 36.7 K, and a reduced quadrupole moment of *Q*/(*(πϵ*_0_ *k*_B_)^1/2^=54.2 in units of (KÅ)^1/2^. While this quadrupole can be implemented directly via a point-multipole interaction, it may also be represented using three point charges, if needed.^48^ Köster et al.^48^ refer to this value as *Q* = 0.6369 DÅ. Calculating the corresponding partial charge using the definition of the quadrupole yields *q*/*e* = 0.120746, as also reported by Barraco et al.^309^, which is consistent with the original model.

Darkrim et al.^327^ referenced a charge of *q* = 0.615 × 10^–26^ esu associated with quadrupole interactions between H_2_ molecules. This appears to be a typographical or unit error. Interpreting this instead as *q* = 0.615 × 10^–10^ esu, an equivalent *q*/*e* = 0.1280 is obtained, which is close to the original charge of the force field.^309^ Using the relation *Q* = 2*qd* ^2^ with *d* = 0.741 Å, the resulting quadrupole moment is *Q* = 0.675 DÅ, which is close to the value of 0.6369 DÅ reported by Köster et al.^48^ In sharp contrast, using the originally stated^327^ *q* = 0.615 × 10^–26^ esu underestimates the original quadrupole magnitude by several orders of magnitude. Ferrando and Ungerer^323^ adopted the charge parameter of *q*/*e* = 0.4664 for the H_2_ atom, which seems to deviate from the original force field by Marx, followed up by Darkrim. Similarly, Rahbari et al.^110^ and Bartolomeu and Franco^308^ adopted the LJ parameters from the Marx force field but used partial charges of *q* = +0.468 for H_2_ and −2*q* for the center of mass, which does not reproduce the quadrupole moment reported in Marx and Nielaba^328^. Another example of inconsistency appears in Li et al.^329^ and Ferrando and Ungerer^323^ who adopted the model by Darkrim et al.,^327^ itself based on the Marx force field, and reported charges of *q* = 0.4664 for H_2_ and −2*q* for the center. It is unclear how this value was derived from the *q* = 0.615 × 10^–26^ esu cited in ref 327 as it does not reproduce the quadrupole originally reported by Marx and Nielaba. Bouanich^330^ proposed another two-site H_2_ LJ 12-6 potential with quadrupole having *σ* = 2.68259 Å, *ϵ*/*k*_B_ = 11.2544 K with a fixed bond length of 0.7508 Å. The partial charge supplemented on H_2_ sites is *q*/*e* = 0.1108 with a −2*q* charge in the center of mass. This force field was fitted using the experimental second-virial coefficients in the 98-773 K temperature range. Sun et al.^331^ proposed a rigid three-site (H– M– H) potential for H_2_ with LJ parameters *σ* = 3.03 Å and *ϵ*/*k*_B_ = 8.06 K, and an intramolecular H-H distance of 0.741 Å. Partial charges of *q*_H_ = +0.47 *e* are placed on each H atom and a compensating charge of *q*_M_ = −0.94 *e* on the central M-site. The force field parameters were fitted using Monte Carlo simulations to reproduc experimental VLE data of H_2_ near the critical temperature (ca. 33 K). Although it is not always possible to trace the complete history of how force field parameters have been used, adopted or modified, it is important to emphasize the need for careful comparison with original references and to explicitly report any deviations or assumptions made by subsequent authors.

Three-site force fields add further complexity by introducing an additional interaction site, typically placed at the geometric center of the molecule or representing a pseudo-atom. This approach enables a better representation of the electronic charge distribution within H_2_, capturing dipole-induced interactions and quadrupole effects more accurately. These effects are particularly relevant in systems where electrostatic interactions play a significant role, such as in the study of H_2_ interactions with polar surfaces or within electric fields. Although three-site models improve the accuracy of intermolecular interactions, there is a higher computational cost compared to one-site and two-site models. Belof et al.^332,333^ parameterized both polarizable and non-polarizable potentials for H_2_, derived from first-principles calculations. The non-polarizable LJ 12-6 potential incorporates three LJ interaction sites along with an embedded quadrupole moment. A fixed bond (with length of 0.742 Å) is used at the ends of which the masses are placed along with a charge of *q*/*e* = 0.3732 each. The charge neutrality is conserved by the addition of a counter charge of −2*q* at the center of mass. The LJ interaction site (*ϵ*/*k*_B_ = 8.8516 K and *σ* = 3.2293 Å) is also placed at center of mass. Two other LJ sites (*ϵ*/*k*_B_ = 4.0659 K, *σ* = 2.3406 Å, and *m* = 0) are also placed 0.329 Å from the center of mass (on the same axis with the sites carrying the masses). The validation of this force field was based on second virial coefficient data for temperatures ranging from 50 to 500 K. The pressure density curves were validated for pressures between 0 and 20 MPa and 0 to 200 MPa, at 298.15 K and 77 K, respectively. For a comparative summary of the selected H_2_ force fields discussed above, including parameter values, number of interaction sites, and fitting approaches, see Table 6.

**Table 6:** Overview of Selected Hydrogen Force Fields and Their Parameters^a^

Force Field	No. of Sites	Fitting Basis	*ϵ*/*k*_B_	*σ*/Å	*q_H_/e*	*Q*/DÅ	Temperature/Pressure Range
Vrabec^48^	1	Speed of sound, PVT data, enthalpy of vaporization	25.84	3.0366			50–250 K
Buch^312^	1	Potential energy, para-ortho H_2_ clusters	34.2	2.96			Low T (34 K)
Hirschfelder (a)^321^	1	Viscosity (classical)	33.3	2.968			Not specified
Hirschfelder (b)^321^	1	Viscosity (classical)	38.0	2.915			Not specified
Hirschfelder (c)^321^	1	2nd virial (quantum)	37.0	2.928			Not specified
Hirschfelder (d)^321^	1	2nd virial (classical)	29.2	2.87			Not specified
Mondal^334^	1		9.56	3.14			
Yang and Zhong^325^	2	PVT and self-diffusion	10.0	2.72			80–300 K, up to 600 bar
Cracknell^311^	2	Adsorption energy, *Q*_st_ in pores	12.5	2.59			298 K
Yang	2	Bulk PVT fit					
Sun^331^	3	VLE near-critical region	8.06	3.03	0.47		near critical region
SAFT-*γ*-Mie^308^	2	Heat capacity at constant pressure, speed of sound, PVT data	18.355	3.1586			
Marx and Nielaba^310^	2+Q	Bond length, enthalpy of adsorption, isochoric heat capacity	36.7	2.958		0.6369	Not specified
Bouanich^330^	2+Q	2nd virial coeff. fit	11.2544	2.68259	0.1108		98–773 K
TraPPE^331^	2+Q	VLE near critical	8.06	3.03	0.47		33 K
Belof^332^	3+Q	Ab initio, second virial coefficient, pressure-density relations	8.8516 (COM), 4.0659 (outer)	3.2293 (COM), 2.3406 (outer)	0.3732		50–500 K; 0–2000 atm

a For extended discussion on the paremeters and consistency issues, the reader is referred to Section 3.1.1. *ϵ* and *σ* are the LJ parameters (see Eq. ([Disp-formula eq3])). Two- and three-site LJ force fields with a quadrupole are referred to as 2+Q and 3+Q, respectively.

As for bulk systems, choosing suitable force fields is a crucial step towards obtaining accurate results in classical molecular simulations of H_2_ hydrates. A force field often used in hydrate studies is Silvera–Goldman^335^ (SG). This model has both LJ and electrostatic interactions but uses a rather complex functional form which is not easy to implement in molecular simulation software. An attempt to resolve this drawback is through the approach proposed by Alavi et al.^336^ where SG is replaced by a three-site LJ-based force field developed to reproduce the experimental quadrupole moment of H_2_. In this model, the two H atoms bear negative point charges, while the positive charge is placed on a “dummy” atom located at the center of mass of the molecule (see Fig. 13c). This force field is the most popular in the field of hydrates, having been used in more than 60% of the MC and MD studies in literature. In the studies of Levesque et al.^337^ and Gu et al.^338^, H_2_ was modeled using an one-site LJ model which takes into account the quantum nature of H_2_ via the Feynman–Hibbs (FH) correction^339^

7 $$U_{\mathrm{FH}}=U_{\mathrm{LJ}}+\frac{\beta \hbar }{24\mu }\left(\frac{d^{2}U_{\mathrm{LJ}}}{dr^{2}}+\frac{2}{r}\frac{dU_{\mathrm{LJ}}}{dr}\right)+\cdot \cdot \cdot$$

where *β* = 1/(*k*_B_ *T*) (where *k_B_* is the Boltzmann constant and *T* is temperature), *μ* = *m*/2 (where *m* is the molecular mass of H_2_), and *ℏ* = *h*/(2*π*) (where *h* is the Planck constant). For computational efficiency in molecular simulations of hydrates, instead of using Eq. ([Disp-formula eq7]), the corrected potential is replaced by an equivalent LJ potential with temperature-dependent *σ* and *ϵ* parameters. This dependence is described using third degree polynomials. The use of higher order (e.g., quartic) terms in Eq. ([Disp-formula eq7]) has also been reported in literature.^340^ However, such terms have been mostly ignored in hydrate studies. Figure 14 shows a comparison of different H_2_ force fields used in studies of hydrate.

**Figure 14:**
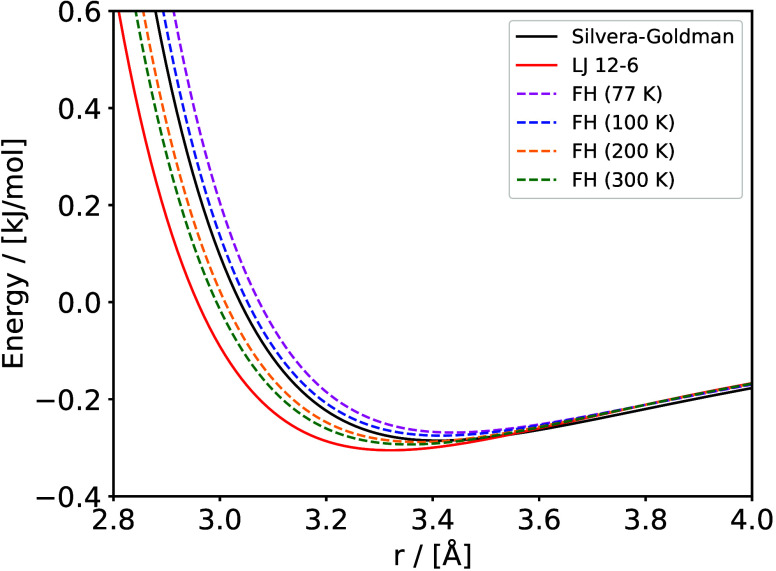
Comparison of different hydrogen potentials: Lennard-Jones (Eq. ([Disp-formula eq3])), Feynman–Hibbs quantum effective potential (Eq. ([Disp-formula eq7])) at different temperatures, and Silvera–Goldman force field. The data are taken from ref 341 (where more temperatures for the FH can be found).

Michalis et al.^342^ in an effort to increase the accuracy of three-phase equilibrium calculations for the system H_2_/H_2_O used a modification factor, *χ*, to correct the Lorentz-Berthelot cross interaction energy parameter according to

8 $$\epsilon_{H(H_{2})-O(H_{2}O)}=\chi \sqrt{\epsilon_{H(H_{2})}\epsilon_{O(H_{2}O)}}$$

where *ϵ*_H(H_2_)_(H_2_) and *ϵ*_O(H_2_O)_(H_2_) are the LJ energy parameters of the H atom in H_2_ and the oxygen atom in H_2_O, respectively. The value of *χ* was obtained by minimizing the error in the calculation of aqueous H_2_ solubility. Finally, a less used force field in hydrate studies is the one by Alavi et al.^343^. This model uses the anisotropic H_2_/D_2_ potential introduced by Wang^344^, which was parametrized to account for quantum mechanical effects of H_2_ motion at low temperatures (of order *ℏ* ^2^ in the second virial coefficient).

#### 3.1.2 Water

Force fields for water are relevant for molecular modeling of processes such as hydrate formation, H_2_ production (e.g., in alkaline electrolyzers), and H_2_ storage (e.g., underground storage, where H_2_ is in contact with aqueous NaCl solutions). Non-polarizable force fields with fixed point-charges are commonly used in molecular simulations of aqueous electrolytes.^345–348^ Non-polarizable force fields of water such as SPC/E^346^ and TIP4P/2005 ^349^ accurately capture thermophysical properties such as densities, radial distribution functions (RDFs), viscosities, and self-diffusivities of water. TIP4P/2005 which outperforms other fixed charged force fields in predicting densities and transport properties,^348^ overestimates the vaporization enthalpy and underestimates the excess chemical potential of water.^110,345,350^ At 323.15 K, the chemical potentials for the TIP4P/2005 water model deviate from the IAPWS empirical EoS^351,352^ by ca. −500 K/*k*_B_. The underestimation of the excess chemical potential of liquid water leads to severe underestimations of the saturated vapor pressure of water compared to experiments, and the solubilities of water in H_2_ compared to experiments.^350^ The TIP3P, TIP4P, TIP4P/*μ* (see the Supporting Information of ref 47 and Section 4.1.2.1), and the SPC water force fields significantly outperform the TIP4P/2005 force field for computing chemical potentials of liquid water.^110,345^ A comparison of the predicting ability of the chemical potential in the liquid phase at *T* = 323 K and pressures ranging between *P* = 10 and *P* = 1000 bar of different water force fields is provided in ref 110. The rigid, five-site TIP5P/Ew^353^ water model has also been used in the literature to study high-pressure H_2_ drying using zeolites.^143^ Polarizable water force fields such as HBP,^354^ BK3,^355^ and AMOEBA^356^ are on average ca. 3-5 times more computationally expensive compared to non-polarizable force fields (the newly developed polarizable force field of Xiong et al.^357^ is an exception). Despite their improved accuracy for various properties of water, polarizable force fields have not gained the widespread popularity of non-polarizable force fields due to their increased computational cost and limited availability in open-source simulation software. For a relevant discussion, the reader is referred to study by Jiang et al.^354^

Already in 1987, Berendsen et al.^346^ discussed that fitting non-polarizable force fields to the free energies of water (i.e., vaporization energies or excess chemical potentials) results in excluding the self-polarization energy of water. The inclusion of self-polarization energy results in accurate predictions for the transport properties of liquid water but at the cost of lower accuracy for the free energies of water. As such, not all thermodynamic and transport properties of aqueous systems can be described simultaneously using a single non-polarizable force field. Different water force fields can be chosen depending on the system or properties of interest. Force fields such as TIP4P/*μ*, which are trained on free energies and chemical potentials are more suitable than TIP4P/2005 for phase coexistence calculations, as accurate predictions for the chemical potentials of water is crucial for improving the prediction of vapor-liquid phase-coexistence compositions of H_2_O – H_2_ systems.^47^ For computing transport properties of H_2_ in liquid water, force fields such as TIP4P/2005 and SPC/E which are trained on liquid phase transport properties are more suitable, as accurate predictions for the viscosity of water ensures that drag forces that influence the transport of H_2_ in water are accurately modeled.

Fig. 15 shows an overview of the relative popularity of the H_2_O force fields used in MC and MD studies of H_2_-hydrate studies. As can be seen, the order of popularity in MC studies (Fig. 15(a)) is TIP4P/Ice,^358^ SPC/E,^346^ TIP5P,^359^ TIP4P/2005,^349^ and TIP4P,^360^ while the respective order for MD studies (Fig. 15(b)) is TIP4P/Ice, SPC/E, TIP4P/2005, TIP5P, and TIP4P. In MD studies, various other force fields have also been used to a lesser extent, e.g., TIP3P,^300^ SPC,^361^ q-TIP4P/F,^362^ TIP4P-FQ/Ice^363^ While the vast majority of H_2_-hydrate studies is performed with non-polarizable H_2_O force fields (which also ignore quantum effects), there are a few exceptions. Rick and Freeman^364^ used the polarizable TIP4P-FQ/Ice H_2_O,^363^ which yields accurate predictions of the density and melting point of ice I_*h*_. TIP4P-FQ/Ice is a reparameterization of the TIP4P-FQ model.^365^ Cendagorta et al.^366^ performed simulations for the quantum free energy profiles and rates of diffusion of H_2_ and D_2_ via the hexagonal face of two neighboring 5^12^6^4^ hydrate cages considering the temperature range 8 K up to 200 K with q-TIP4P/F^362^ H_2_O, which is a modified version of TIP4P/2005. q-TIP4P/F is flexible and implicitly accounts for quantum effects.

**Figure 15:**
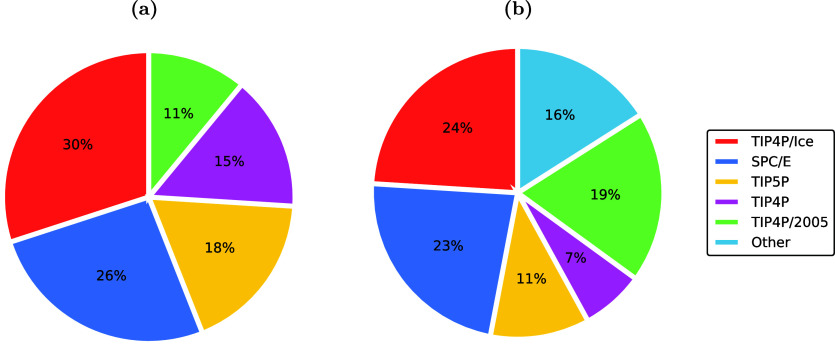
Overview of the relative popularity of the H_2_O force fields used in the H_2_-hydrate literature of (a) MC and (b) MD studies. In panel (b), “Other” includes TIP3P (4%), TIP4P-FQ/Ice (2%), q-TIP4P/F (2%), SPC (2%), and other force fields. The statistics are based on the studies considered in this review.

During recent years, an increase in the use of TIP4P/Ice^358^ H_2_O force field in MD studies of hydrates is observed. TIP4P/Ice is a rigid and non-polarizable model which was designed to accurately reproduce the melting temperature of hexagonal ice (I_*h*_). García Fernández et al.^367^ reported a melting temperature of *T*_m_ = 270 ± 3 K. In a more recent study, Conde et al.^368^ performed a systematic study to increase the accuracy of the direct phase coexistence approach (see Section 3.2.14), and recommended a value of *T*_m_ = 269.8 ± 0.1 K for I_*h*_. These calculations, therefore, show a clear under-prediction of the melting point of TIP4P/Ice by 3.35 K. Conde and Vega^369^ argued that the accurate calculation of the melting temperature of hexagonal ice is essential for the accurate calculation of the three-phase coexistence temperature of hydrate systems using the phase coexistence approach.

#### 3.1.3 Ionic Species

The presence of ions, such as Na^+^, K^+^, Cl^–^, and OH^–^, influences the thermophysical properties of water, which in turn affects the solubility, diffusivity, and phase behavior of H_2_ in the system. Similarly to water, force fields for ions can be either non-polarizable or polarizable. Non-polarizable force fields of ions, such as Madrid-2019 ^370^ and Joung-Cheatam,^371^ assume fixed charges on the ions. Polarizable force fields for ions, such as the ones developed by Kiss and Baranyai^372^ and Dočkal et al.^373^, use more elaborate functional forms e.g., Gaussian charge distributions and the charge-on-spring model. In the “charge-on-spring” model a massless charge is attached to each ion by a harmonic spring. This allows the charge to move in response to the local electric field. As in the respective water force fields, the additional calculations required to determine the positions and forces on these charge-on-spring elements increase the complexity and the time requirements of the simulation (typically by a factor of 3-10).^372^ Recent advances in polarizable force fields aimed at overcoming these limitations, striving to balance accuracy and efficiency.^357,373^

Until 2009, force fields of ions were primarily parameterized based on free hydration energies of salts and RDFs of ions and water (e.g., the location of the first peak and the number of water molecules in the first hydration layer).^371,374–377^ A list of available ion force fields (until 2011) and the target properties, which are used for their validation is provided by Reif and Hünenberger^378^. The non-polarizable force field developed by Joung and Cheatham^371^ for alkali (e.g., Li^+^, Na^+^, and K^+^) and halide (e.g., F^–^, Cl^–^, and Br^–^) ions is particularly popular in literature. The Joung-Cheatham^371^ force field uses nominal charges (i.e., +1 [e] for Na^+^ and −1 [e] for Cl^–^) to model the electrostatic interactions of ions in the solution and can accurately predict the free hydration energies of ions in the aqueous solution along with the lattice energies (and constants) of the alkali halide salts in crystal form. Despite the success of the Joung-Cheatham force field for describing the free energies of ions, the dynamic properties (e.g., viscosities and self-diffusivities of ions and water) in concentrated salt solutions (i.e., beyond a molality of 1 mol salt / kg salt) cannot be accurately modeled with respect to experiments.

Since 2009, Leontyev and Stuchebrukhovhav have published several works using the concept of ”scaled charges” for modeling ions in aqueous solutions.^379–382^ Scaling down the charges of ions by a factor of 0.7-0.9 allows for accounting the dielectric screening of electrostatic charges in the aqueous medium.^370,381,383^ Use of scaled charges has been shown to significantly improve the accuracy of predicting dynamical properties such viscosities, diffusion coefficients of water, and electrical conductivities for aqueous electrolyte solutions^112,113,370,384,385^ at the cost of less accurate predictions for free hydration energies of salts. Several different force fields have been recently developed for modeling ions using scaled charges.^370,384^ Madrid-2019, developed by Zeron et al.^370^ in 2019, was parameterized based on the TIP4P/2005 ^349^ water force field, and is particularly popular in literature of aqueous electrolytes due to its computational efficiency and accurate predictions of densities, RDFs, infinite dilution self-diffusivities of ions, and freezing point depression in aqueous electrolyte solutions. Madrid-2019 uses a charge scaling factor of 0.85 and has parameters for a wide array of ions such as Li^+^, Na^+^, K^+^, Cl^–^, and SO_4_^2–^), which are particularly important for H_2_ applications. Despite its widespread use, Madrid-2019 force field cannot accurately predict all properties (e.g., free hydration energies of salts and the transport properties of concentrated aqueous solutions). Recently, the Madrid-Transport^384^ (with a charge scaling factor of 0.75) has been developed for aqueous NaCl and KCl solutions, which outperform the Madrid-2019 ^347^ in predicting the transport properties (i.e., viscosities, self-diffusivities of ions, and electrical conductivities). However, this force field yields less accurate results for interfacial tensions and activities of water in aqueous electrolyte solutions.

Despite being scarce, molecular simulation data for properties of systems involving H_2_ and more complex ionic species, such as ionic liquids (ILs), have been reported. In these studies, common force fields for ILs are the ones developed by Maginn and co-workers,^386–388^ the General Amber force field (GAFF),^307^ the Optimized Potentials for Liquid Simulations-All Atom (OPLS-AA) for ILs by Sambasivarao and Acevedo^389^, and the model by Koller et al.^390^. Usually, the atomic point charges of the atoms of ILs are derived from quantum mechanical calculations using e.g., the Restrained Electrostatic Potential (RESP) method.^391^ Similarly to the monovalent ions discussed earlier, a common practice in simulations of ionic systems such as ILs and DES is to scale the charges obtained from RESP (or other QM-based methods) to reduce the charge screening, and hence, improve force field accuracy. For more details on this approach, the reader is referred to refs 389 and 392–396 and the discussion in Section 4.1.2.

A wide variety of force fields for other ions, important for modeling H_2_ processes, such as the Delft-Force field (for OH^–^, B­(OH)_4_^–^, and BH_4_^–^ ions),^113,397^ have been developed recently. For a detailed overview of common ion models the reader is referred to the review by Nezbeda et al.^398^ and refs 302, 370, and 385.

#### 3.1.4 Nanoporous Materials, Membranes, and Solid Substrates

In the studies of H_2_ separations or storage in nanoporous materials (e.g., MOFs, COFs, zeolites, CNTs), general force fields are mainly used. Among them, the Universal force field (UFF), developed in 1992 by Rappe et al.,^399^ is designed to be applicable across the entire periodic table. UFF parameters are based on general rules that consider an element’s hybridization and connectivity. Similarly, DREIDING by Mayo et al.^400^ is a widely used generic force field aimed at predicting the structures and dynamics of organic, biological, and main-group inorganic molecules, making it a reasonable choice for polymer simulations. To better account for the wide structural space of specific functional materials families, more sophisticated approaches have been developed; QuickFF^401^ is a force field parametrization protocol for MOFs and other nanoporous materials that produces force field parameters from first-principles calculations. UFF4MOF^402^ is an extension of UFF aiming at improved accuracy for MOFs. IntraZIF-FF^403^ and ZIF-FF^404^ are specialized force fields for ZIFs, capturing their flexibility and dynamic behavior.

For representing both crystalline and amorphous polymeric materials (e.g., in studies of barrier materials in H_2_ tanks, and as membranes for separations and in electrochemical processes), the most common force fields are the condensed-phase optimized molecular potentials for atomistic simulation studios (COMPASS),^315,316,405^ the polymer consistent force field (PCFF),^406^ and OPLS.^360^ These popular polymer force fields are combined with different H_2_ models, such as the ones discussed in detail in Section 3.1.1. It is also common that specially developed energy interaction parameters for the H_2_ ^325,407^ are used to study properties of H_2_/polymer systems. It is noteworthy that despite being one of the most successful force fields for these systems, COMPASS is a proprietary model, part of the Direct Force Field software suite.^408^ Other force fields used to model polymeric systems include the modified Adaptive Intermolecular Reactive Empirical Bond Order potential (AIREBO-M),^409^ modified versions of the DREIDING force field (e.g., Mabuchi and Tokumasu^410^), and the model by Smith et al.^411^. Scarcely, the anisotropic united atom force field (AUA4)^412^ is used to model polymeric materials.^413^ It is important to note that many of these models, have also been used for zeolites and other nanoporous materials.

When computing the adsorption and storage capacities of H_2_ in functionalized nanoporous materials, intramolecular interactions are often neglected.^198,317,414^ Although MOFs are assumed to be rigid in most H_2_ adsorption studies, it is important to note that the presence of gases, such as CO_2_, in MOFs can induce swelling and phase transitions, which can result in changes to pore-sizes and adsorption properties.^415^ To study these phenomena, flexible force fields are required. A review of flexible force fields for MOFs is provided by Heinen and Dubbeldam.^415^ There is no general consensus as to which H_2_ force field is most suitable for adsorption in MOFs.^414^ For strong H_2_ adsorption energies (especially for open metal and H_2_ interactions), the Morse potential can be used to model the interatomic interactions between H_2_ and the MOFs. The Morse potential has been shown to perform better than LJ in modeling stronger interatomic interactions.^317^ Many studies in the literature underline the importance of quantum-corrections to classical potentials of H_2_, with the most popular being the quasi-classical Feynmen-Hibbs correction^317^ (see also Eq. ([Disp-formula eq7]) and Fig. 14). Without quantum corrections, the low temperature (below 70 K) adsorption capacity of H_2_ can be over-predicted by ca. 20% as shown by Bobbitt and Snurr.^414^ Although most H_2_ force fields neglect the polarization of H_2_ (primarily due to its small size), polarization can play an important role in H_2_ adsorption especially when dealing with open metal adsorption sites. Polarization of H_2_ significantly enhances the binding between H_2_ and the adsorbent.^414^ Thus, neglecting polarization by using non-polarizable H_2_ force fields,^312^ can result in inaccurate predictions of binding sites and capacities as shown by Franz et al.^416^ In such cases, it is necessary to use polarizable force fields of H_2_ or ab-initio simulations.^414,416^ Getman et al.^317^ and Bobbitt and Snurr^414^ discuss in detail molecular simulations of H_2_ adsorption on MOFs, and the use of appropriate force fields. In practice, researchers often need to make a compromise when choosing force fields, since the most accurate representation of adsorbate–organic group and adsorbate–metal interactions may not be equally well captured within a given model. While many studies adopt transferable force fields, others have introduced custom parameterizations in an effort to better capture the structural behavior of the frameworks of the porous media, particularly when new metals are present (see for example refs 417 and 418). Such refinements do not guarantee a universally improved adsorbate–framework description, but can enhance e.g., the accuracy of framework flexibility.

For guest gas molecules other than H_2_ (e.g., CO_2_, hydrocarbons), widely used force fields include the Transferable Potentials for Phase Equilibria (TraPPE), designed by Martin and Siepmann^301^ to model phase equilibria of gases and hydrocarbons, emphasizing transferability and vapor-liquid coexistence properties, the Elementary Physical Model 2 (EPM2) by Harris and Yung^419^ developed to accurately describe the phase behavior and thermodynamic properties of CO_2_, making it suitable for gas adsorption studies, and OPLS.^360,420,421^ In general, OPLS and GAFF^307^ force fields are the most widely used force field for modeling hydrocarbons and guest molecules in porous materials.

MD studies related to H_2_ storage in subsurface geological formations typically use classical force fields tailored to represent the structural and interaction properties of solids. Among these, CLAYFF (and its variations)^422–424^ is the most prevalent, designed explicitly for layered minerals such as montmorillonite, kaolinite, pyrophyllite, beidellite, quartz, hematite, and calcium silicate hydrates. CLAYFF predominantly treats interactions through non-bonded potentials, capturing the essential physics of clays and related nanoporous materials with particular emphasis on hydration and surface interactions. UFF^399^ is also frequently chosen to model inorganic solids such as kaolinite, illite, dolomite, quartz, and calcite, thanks to its wide applicability across the periodic table, and parametrization approach based on atomic hybridization and connectivity. The INTERFACE force field^425^ extends popular biomolecular and organic force fields to accurately simulate inorganic oxide interfaces, as exemplified by its application to kaolinite. Other specialized force fields used in molecular simulations of H_2_/substrate systems are the CVFF^426^ and PCFF++^427^ for modeling kerogen, OPLS^428^ for graphene, and specialized models for silica-based meso-porous materials e.g., MCM-41.^429^ Furthermore, force fields such as PCFF+ ^316,406,430^ for SiO_2_, and those specifically optimized for calcite (e.g., by Xiao et al.^431^ and Raiteri et al.^432^), highlight the importance of targeted development of force fields to accurately capture specific structural, mechanical, or thermodynamic properties relevant to UHS.

#### 3.1.5 Metal-Hydrogen Systems

Several classes of interatomic potentials have been developed to model metal–H interactions, including classical many–body methods such as the Embedded–Atom Method (EAM), its modified form (MEAM), Angular–Dependent Potentials (ADP), and Bond–Order Potentials (BOP), as well as modern machine–learning interatomic potentials (MLIPs). These methods represent a hierarchy of increasing complexity and accuracy in describing metal–H systems.

The EAM potential is a many–body framework that models metals by combining pairwise interactions with an embedding function representing the host electron density. Developed by Daw and Baskes,^433^ EAM was groundbreaking in capturing metallic cohesion more accurately than simple pair potentials. Notably, the original work already addressed H in metals, employing EAM to study H interstitials, surface adsorption, and even the effect of H on Ni and Pd fracture.^434^ This demonstrated the unique capability of EAM to model HE problems that pair potentials could not resolve. EAM potentials have been extensively parameterized for various metal–H systems, enabling detailed simulations of H trapping at defects and its weakening effect on metal bonds. Currently available EAM potentials for metal–H systems include Pd–Ag–H,^435^ Al–Ni–H,^436^ Fe–H,^437–439^ Pd–H–He,^440^ W–H–He,^441^ Ni–H,^442^ Pd–H,^440^ W–H,^443^ and Fe–Cr–Ni–H.^444^

The MEAM potential extends the EAM framework by incorporating directional bonding effects, addressing a key limitation of traditional EAM potentials. Developed by Baskes^445^, MEAM was the first semi–empirical scheme able to treat fcc, bcc, hcp and diamond structures within one formalism, thereby capturing both metallic and covalent bonding. It has since been extended to second–nearest–neighbour (2NN–MEAM)^446^ and multi-component versions and is now widely used for metal–H systems. Compared to EAM, the inclusion of angular terms in MEAM, provides enhanced accuracy in modeling HE phenomena across diverse metals, though at the cost of increased computational and parametric complexity. To date, MEAM potentials have been developed for various metal–H systems, including Al–H,^447^ Al–V–H,^448^ Fe–H,^449^ Ni–H,^447^ Ni–V–H,^448^ V–H,^450^ and Zr–H.^451^

ADPs offer a streamlined way to include directional bonding effects in metals, extending the EAM as a more tractable successor to MEAM. Introduced by Mishin et al.,^452^ ADPs supplement the usual EAM energy with explicit dipole and quadrupole terms that penalize deviations from high-symmetry environments, capturing bond-angle dependence via analytic functions rather than the many–body screening routines of MEAM. These additional angular contributions span multiple coordination shells and employ smooth cutoffs for efficiency. Although initially parameterized for transition metals like bcc Fe and fcc Ni,^452^ the same formalism has since been adapted successfully to a variety of alloys and metal–H systems. Currently available ADP parameterizations include Al–H,^453^ Cr–Fe–H,^454^ and Mg–H^455^ systems.

BOPs derive from quantum mechanical principles, where bond strength depends explicitly on the local coordination environment. Unlike simpler approaches, BOPs incorporate multi–atom interactions (three– and four–body terms) that dynamically adjust bond energies based on atomic geometry.^456^ This formulation provides superior description of directional bonding and bond-breaking/forming processes compared to ADP or MEAM, though at the expense of greater mathematical complexity. Developed initially for alloys and semiconductors,^457^ BOP frameworks have been successfully extended to metal-H systems. Currently available BOP parameterizations for metal-H systems include Al-Cu-H^458^ and Cu-H.^459^

MLIPs have emerged in the past decade as a transformative approach, leveraging data-driven models to achieve accuracy approaching that of DFT. The field was pioneered by Behler and Parrinello’s neural network potential,^460^ which demonstrated that high-dimensional neural networks could accurately reproduce DFT potential energy surfaces. Subsequent advances, including Gaussian approximation potential (GAP)^461^ and moment tensor potential (MTP),^462^ have broadened this methodology, enabling the modeling of complex multi-component systems through flexible functional forms. Unlike traditional empirical potentials, MLIPs are not constrained by predefined functional forms tied to specific theoretical frameworks (e.g., electron density or bond order). This allows MLIPs to achieve accuracies comparable to quantum-mechanics across diverse atomic environments. However, this comes at the cost of requiring extensive training data and reduced physical interpretability compared to conventional potentials.^463^ For metal–H systems, MLIPs represent a significant breakthrough as they can simultaneously describe metallic, covalent, and even molecular interactions relevant to HE. Importantly, MLIPs serve as a unifying framework that can, in principle, replicate the behavior of EAM, MEAM, BOP, and other conventional potentials when trained on sufficient data, thereby bridging gaps between these established methodologies. With advancing computational power and algorithms, MLIPs are becoming indispensable tools for investigating HE across various metal systems with unprecedented detail. Successful applications include Pd-H,^464^ Nb-H, W-H,^465^ Ti-H,^466^ Fe-H,^467^ and multi-component systems like Mo-Nb-Ta-W-H,^468^ and Fe-Co-Ni-Cr-Mn-H.^469^

The landscape of interatomic potentials for metal-H systems reveals two fundamentally distinct modeling philosophies. Traditional approaches (EAM, MEAM, ADP, and BOP) employ compact functional forms grounded in physical principles, with parameters carefully optimized against experiments or DFT reference data. These models incorporate essential physics through distinct mechanisms: electron-density embedding in EAM, angular corrections in MEAM and ADP, and bond-order dependence in BOP. These fundamental physical principles serve as a safeguard, ensuring that even beyond their fitted regimes, the potentials typically maintain reasonable metallic or covalent behavior. In contrast, MLIPs forgo explicit physical constraints in favor of complex, high-dimensional functions with thousands of parameters trained on extensive DFT datasets.^463^ While this flexibility enables remarkable accuracy across diverse structural and chemical environments when training data is comprehensive, it carries an inherent risk: configurations outside the training domain may produce unphysical predictions that empirical potentials would naturally avoid. Recent efforts to develop hybrid approaches, such as physics-informed neural network potentials, aim to bridge this divide by embedding physical constraints within ML architectures.^470^ However, these methods currently remain limited in scope (e.g., to pure Al^471^ and Ta^472^ systems), and have not yet been successfully extended to metal-H applications.

When selecting interatomic potentials for HE studies, users must carefully balance two critical factors: (1) the accuracy for target phenomena, and (2) computational efficiency. For physics-based potentials (EAM, MEAM, ADP, BOP), accuracy depends on proper parameterization for specific applications, as demonstrated by Song and Curtin,^276^ who refined Fe-H and Ni-H EAM potentials to better capture H-defect interactions, highlighting how even established models may require reparameterization for certain HE mechanisms. For MLIPs, accuracy is intrinsically linked to training data coverage; for instance, an MLIP trained only on bulk H diffusion^465^ cannot reliably predict H-defect interactions^469^ absent from its training set. Computational cost varies significantly across methods as shown in Table 7: relative to EAM, ADP and MEAM increase costs by ca. 3× and 7×, respectively, while BOP reaches ca. 13×.^473^ Modern MLIPs (e.g., ACE, MTP16) further escalate costs to 20–66×,^474^ though they offer near-DFT accuracy. Thus, the choice hinges on the priorities of the study: empirical potentials provide transferable, computationally efficient frameworks with inherent physical safeguards, whereas MLIPs deliver higher fidelity at greater computational expense, provided their training data adequately represents the relevant atomic configurations for HE.

**Table 7:** Comparison of Computational Costs for Different Interatomic Potentials, Normalized Relative to EAM^a^

Force field	CPU cost relative to EAM (times)
EAM	1
ADP	3
MEAM	7
BOP	13
ACE	20
MTP16	66

a Performance data for EAM, ADP, MEAM, and BOP potentials are obtained from LAMMPS benchmark tests,^473^ while results for ACE and MTP16 (where 16 denotes the MTP level) are sourced from Shuang et al.^474^

### 3.2 Computation of Hydrogen Properties from Molecular Dynamics Simulations

The past two decades, MD simulations have been proven to be an invaluable tool for studying properties of H_2_ systems. In brief, MD performs numerical integration of Newton’s equations of motion to yield the positions of particles over time. Unlike MC simulations, which are limited to computing thermodynamic ensemble averages, MD simulations provide insight into both equilibrium and transport properties of a system. The interactions between atoms are determined based on the chosen force field (discussed in detail in Section 3.1). Typically, an MD simulation starts by initializing the positions and velocities of all particles in the simulation box, followed by an equilibration period to bring the system at thermodynamic equilibrium. Then, a production run, usually in the order of ns, is performed to sample the relevant properties. For all details regarding MD simulations the reader is referred to the relevant textbooks.^51,52,297^

The most common open source MD simulation packages for modeling H_2_ systems are the Large-scale Atomic/Molecular Massively Parallel Simulator (LAMMPS)^475^ and Groningen Machine for Chemical Simulations (GROMACS).^476,477^ The modular structure of these simulation tools (and especially of LAMMPS), allows for extensions to the main code (e.g., via the creation of plugins) to accommodate new or advanced computations which are not part of the package (for example the information in ref 478). Nevertheless, other open-source MD software such as DLPOLY^479^ and RASPA^480^ is also used for modeling H_2_ systems. Aside from the simulation packages, visualization software, such as VMD,^481^ AVOGADRO,^482^ iRaspa,^483^ and OVITO,^484^ is an indispensable tool for not only visualizing simulations but also computing properties and creating initial configurations. These tools are used to visualize both MD and MC simulations.

#### 3.2.1 Transport Properties: A Brief Introduction

Both equilibrium (EMD)^51,52^ and non-equilibrium molecular dynamics (NEMD)^52,485^ simulations are naturally suited, and thus, widely used for computing transport coefficients. In NEMD, transport coefficients are calculated using^485^

9 $$\gamma =\underset{X\to 0}{\lim}\underset{t\to \infty }{\lim}\frac{J}{X}$$

where *γ* is the transport coefficient (e.g., self-diffusion coefficient), *X* is (usually) a fictitious field that drives a conjugate flux, *J* (i.e., mass flux in case of self-diffusivity or momentum flux for the case of dynamic viscosity), and *t* is the simulation time.^485^ In EMD, transport coefficients can be computed either by using the Green Kubo (GK) or the Einstein relations.^51,485,486^ Following the GK approach, transport coefficients are computed from integrals of time-correlation functions of dynamical variables, according to^52,299,487^

$$\gamma =\int_{0}^{\infty }\left\langle Ȧ(t^{\prime })Ȧ(0)\right\rangle dt^{\prime }$$ 10

where *Ȧ* is the corresponding dynamic variable and the angle brackets ⟨···⟩ denote an ensemble average. The Einstein method follows^52^

11 $$\gamma =\left\langle {\left(A\left(t\right)-A\left(0\right)\right)}^{2}\right\rangle /2t$$

which is a linear relation between time (*t*) and the mean-squared displacement (MSD) of the dynamical variable. Eq. ([Disp-formula eq11]) is valid at time-scales where the slope of MSD­(*t*) equals 1 in the logarithmic scale.^51,52^ Using this as a criterion, one can specify the minimum simulation duration for computing transport coefficients using the Einstein method. Such a criterion is not present in the GK approach, where the tail of the time-correlation functions converges to zero regardless of how long the simulation is.^488,489^ Thus, from a purely practical standpoint, the Einstein method is advantageous for computing transport properties. Nevertheless, both GK and Einstein methods should yield identical results.^490^

An advantage of using EMD over NEMD simulations is that a single simulation can be used to sample multiple different transport properties.^485,486^ NEMD simulations often require multiple simulations at different field strengths (to extrapolate to 0 field) for each transport property.^94^ An advantage of NEMD simulations is that, unlike EMD simulations, it can be used to simulate non-linear response behaviors such as the rheology of non-Newtonian fluids.^485^ While EMD is well suited for studying spontaneous fluctuations in equilibrium, NEMD is particularly effective for investigating transport processes driven by external forces, such as electro-osmotic drag in polymer membranes. Since most of the systems reviewed in this work deal with aqueous Newtonian systems (such as alkaline electrolyte solutions), primarily EMD simulations are discussed in this work.

To compute transport properties using EMD in LAMMPS,^475^ the OCTP plugin^491^ or the PyLAT^492^ post-processing tools can be used. In the OCTP plugin, Einstein relations are used in combination with the order-*n* algorithm^51^ as implemented by Dubbeldam et al.^493^ PyLAT^492^ uses a combination of GK (for dynamic viscosity and ionic conductivity), and Einstein relation (for self-diffusivity) expressions.

#### 3.2.2 Viscosity

Computing viscosity, *η*, via EMD is generally preferred over NEMD because it uses spontaneous fluctuations in pressure of an unperturbed system avoiding introducing external fields or shear flows, which can potentially induce non-linear effects.^51,52,490^ As mentioned earlier for all transport properties, *η* in EMD can be computed by using either the GK approach or the Einstein relation.^490^ The Einstein expression for *η* is as follows

$$\eta =\underset{t\to \infty }{\lim}\frac{d}{dt}\left[\frac{1}{20}\frac{V}{k_{B}T}\left\langle \underset{\alpha \beta }{\sum }{\left(\int_{0}^{t}dt^{\prime }p_{\alpha \beta }^{\mathrm{Tr}}(t^{\prime })\right)}^{2}\right\rangle \right]$$ 12

where *t* is time, *V* is the volume of the simulation box, *k_B_* is the Boltzmann constant, *T* is the temperature, and ⟨···⟩ indicates an ensemble average. *p*_*αβ*_^Tr^ are the components of the traceless pressure tensor calculated using

13 $$p_{\alpha \beta }^{\mathrm{Tr}}=\frac{p_{\alpha \beta }+p_{\beta \alpha }}{2}-\delta_{\alpha \beta }\left(\frac{1}{3}\sum_{k}p_{kk}\right)$$

where *δ_αβ_* is the Kronecker delta. Eq. ([Disp-formula eq12]), allows viscosities to be computed directly from the slope of the MSD (term in the bracket) at long times.^490^ This approach yields results identical with those obtained from the GK method, but avoids the complexity of fitting correlation functions, as discussed by Maginn et al.^490^

#### 3.2.3 Self-Diffusivity

Self-diffusivity is the mass transport mechanism of a species driven by Brownian motion, occurring in the absence of chemical potential, temperature, or pressure gradients.^494^ This review mainly deals with self-diffusivities in binary and multi-component gas/liquid mixtures containing H_2_. In a multi-component mixture, the self-diffusivity can be calculated as follows

14 $$D_{i}^{\mathrm{self},\mathrm{MD}}=\underset{t\to \infty }{\lim}\frac{d}{\mathrm{dt}}\left[\frac{1}{2dN_{i}}\left\langle \sum_{j=1}^{N_{i}}{\left(r_{j,i}(t)-r_{j,i}(0)\right)}^{2}\right\rangle \right]$$

where **r**_*j*,*i*_(*t*) is the position of the *j* ^th^ molecule of species *i* at time *t*, ⟨···⟩ denotes an ensemble average, and *d* is the dimensionality of the system. Better statistical accuracy in the computation of *D*_*i*_^self,MD^ is achieved by considering the MSDs of all *N_i_* molecules in Eq. 14. At early times (*t* → 0), a molecule exhibits ballistic motion with MSD scaling as *t* ^2^, while at long times (*t* → *∞*), the MSD scales linearly with *t*, indicating diffusive behavior, during which self-diffusivities are computed. It is important to note that although the slope of unity in the log-log plot of MSD as a function of time is a necessary condition for computing self-diffusivities (and other transport coefficients), it is not always sufficient. According to Maginn et al.^490^, the square root of the MSD should be comparable or larger than the simulation box size length. This is a common practice ensuring that the diffusing molecule has explored a substantial part of the configurational space. A similar rule of thumb has been recently proposed by Kärger et al.^495^ for diffusivity in nanoporous materials, where the square root of the MSD should be at least equal to one unit cell. This criterion ensures that the molecule is diffusing and not simply vibrating around its position.

Self-diffusivities computed in MD simulations suffer from finite-size effects which arise from spurious hydrodynamic interactions between the particles in the simulation box with their periodic images. These effects can be substantial, depending on the system size and type, and particularly prominent in dense systems, i.e., liquids or compressed gasses.^496^ To mitigate finite-size effects, one should either perform MD simulations with large system sizes or use analytical corrections.^497–500^ The most widely used correction term, the so-called Yeh-Hummer correction (*D* ^YH^), was derived in the pioneering studies of Dünweg and Kremer^497^ and Yeh and Hummer.^498^ The self-diffusivity at the thermodynamic limit can be obtained via the following expression

15 $$D_{i}^{\mathrm{self}}=D_{i}^{\mathrm{self},\mathrm{MD}}+D^{\mathrm{YH}}(T,\eta ,L)=D_{i}^{\mathrm{self},\mathrm{MD}}+\frac{k_{B}T\xi }{6\pi \eta L}$$

where *D*_*i*_^self^ is the finite-size corrected self diffusivity of the *i* ^th^ species, *ξ* is a dimensionless constant equal to 2.837298, and *L* is the box length of a cubic simulation box. *η* is the shear viscosity computed from MD simulations, which is shown to be independent of the size of the system.^496,498,501–503^

#### 3.2.4 Maxwell-Stefan and Fick (Mutual) Diffusivities

Mutual diffusivities describe the collective diffusion of species which is commonly modeled using the MS (Maxwell-Stefan) and Fick frameworks. This review mainly focuses on mass transport phenomena in binary and multi-component gas/liquid mixtures containing H_2_. The MS framework expresses diffusion in terms of chemical potential gradients, where the flux is driven by differences in the chemical potential, *μ_i_*, of each species and characterized by the MS diffusivity, Đ, an inverse-friction coefficient.^266,321^ On the other hand, the Fickian approach uses concentration gradients, with flux governed by the Fick diffusivity, *D* ^Fick^.^266,504^ Despite the different gradients considered, MS and Fick frameworks describe the same physical process, and are mathematically connected by the so-called thermodynamic factor, Γ, for diffusion, according to^505–509^

16 $$D^{\mathrm{Fick}}=\Gamma {Đ}^{\mathrm{MS}}$$

As discussed in more detail in Section 3.2.13, Γ accounts for deviations from ideal behavior, and depends on the mixture’s composition, pressure, and temperature.^510^ While MS diffusivities are linked to chemical potentials, which are difficult to measure experimentally,^511^ Fick diffusivities are more commonly reported due to their dependence on measurable concentration gradients.^266^ Direct calculation of *D* ^Fick^ using non-equilibrium molecular dynamics simulations is possible, but impractical as it requires setting up systems with large concentration gradients, yielding inaccurate predictions of *D* ^Fick^ as discussed in the studies by Liu et al.,^506^ Maginn et al.,^512^ Tsige and Grest.^513,514^

In an *N* – component mixture, there are *N*(*N* – 1)/2 unique MS diffusivities which can be represented as a symmetric matrix. By performing EMD, the MS diffusivity, Đ, in binary mixtures are computed by first obtaining the Onsager coefficients Λ_*ik*_ at zero total linear momentum.^505–507^ Λ_*ik*_ is computed from the cross-correlations of the molecular displacements of species *i* and *k* ^505–507,515^

17 $$\Lambda_{ik}=\underset{t\to \infty }{\lim}\frac{d}{dt}\left[\frac{1}{6N_{\mathrm{tot}}}\left\langle \left(\sum_{l=1}^{N_{i}}(r_{l,i}(t)-r_{l,i}(0))\right)\times \left(\sum_{m=1}^{N_{k}}(r_{m,k}(t)-r_{m,k}(0))\right)\right\rangle \right]$$

where *N_i_*, *N_k_N*_tot_ are the number of molecules of type *i* and *k*, and the total number of molecules in the system, respectively. *Đ* ^MS,MD^ for a binary mixture is then expressed as a linear combination of the Onsager coefficients,^505–507,515^

18 $${Đ}^{\mathrm{MS},\mathrm{MD}}=\frac{x_{2}}{x_{1}}\Lambda_{11}+\frac{x_{1}}{x_{2}}\Lambda_{22}-2\Lambda_{12}$$

where *x*_1_ and *x*_2_ are the mole fractions of the components in the binary mixture.

As in the computation of self-diffusivities with EMD, finite-size effects are present also in MS diffusivities.^499,503^ In a binary mixture, MS diffusivities can be corrected for finite-size effects using the term derived by Jamali et al.:^503^

19 $${Đ}^{\mathrm{MS}}-{Đ}^{\mathrm{MS},\mathrm{MD}}=\frac{1}{\Gamma }\frac{k_{B}T\xi }{6\pi \eta L}=\frac{1}{\Gamma }D^{\mathrm{YH}}$$

Fick diffusivities, *D* ^Fick,MD^, can be computed using Eq. ([Disp-formula eq16]). As shown by Jamali et al.,^516^ extrapolation of *D* ^Fick,MD^ to its value at the thermodynamic limit follows from:

20 $$D^{\mathrm{Fick}}-D^{\mathrm{Fick},\mathrm{MD}}=D^{\mathrm{YH}}$$

Eq. ([Disp-formula eq20]) shows that the finite-size correction to Fick diffusivities equals the Yeh-Hummer correction term for self-diffusivities (Eq. ([Disp-formula eq15])). Corrections for an *N*-component system, were derived by Jamali et al.^500^. In an *N* component mixture, unlike *Đ* ^MS^, there are (*N* – 1)^2^ distinct Fick diffusivities where label order matters, i.e *D*_*ij*_^Fick^ ≠ *D*_*ij*_^Fick^. For a binary mixture, however, *D*_12_^Fick^ = *D*_21_^Fick^ = *D* ^Fick^.

The discussion above focuses on mass fluxes in a molar reference frame, where velocities are mole-fraction-weighted. Mass fluxes can also be evaluated using mass- or volume-averaged velocities, yielding different Fick diffusivities.^266,517^ The benefits of each reference frame are discussed in detailed in Taylor and Krishna^266^ (Section 3.1), Bird et al.^517^ (Section 17.7), and in refs 508, 510, 518. Notably, for binary mixtures, Fick diffusivities remain identical across reference frames.^266,517^ The discussion provided here is not exhaustive, but is meant to showcase the main approach for computing mutual diffusivities of H_2_ mixtures using EMD. For detailed discussions on the MS and Fick frameworks in multi-component mixtures, the reader is referred to refs 266, 267, 500, and 519–526.

#### 3.2.5 Diffusivity in Nanoporous Materials

Diffusion inside nanoporous solids is studied extensively using experimental techniques such as frequency response methods, pulsed-field gradient NMR, and quasi-elastic neutron scattering.^527^ These approaches define different, but closely related to each other types of diffusivities. Among these types, self-diffusivities in sorbents are convenient to compute via EMD using the methodology discussed in detail in Section 3.2.3. Transport diffusivity (*D*_t_(*c*), where *c* is the concentration of the species in the material) is one of the most widely computed quantities to quantify the propagation of diffusing species in sorbents. *D*_t_(*c*) can be obtained using the following expression:

21 $$D_{t}(c)=D_{0}{\left(\frac{\partial \ln f}{\partial \ln c}\right)}_{T}$$

The second term on the right-hand side in Eq. ([Disp-formula eq21]) is the thermodynamic factor, i.e., the derivative of the logarithm of the fugacity of the bulk phase (*f*) with respect to the logarithm of the concentration of the adsorbed phase.^528^ Fugacities can be calculated via MC simulations (see Section 3.3). *T* is temperature and *D*_0_ is the so-called corrected diffusivity, which describes the displacement of the center of mass of the molecules propagating in the pores of the solid. Note that *D*_0_ depends on *c*. Both self- and transport diffusivities, depend on the concentration or the guest loading. For a useful discussion on the subtle but critical differences between the measured and computed diffusivities, the approximations made in relating macroscopic and microscopic diffusion measurements, and the assumptions used in permeation models, the reader is referred to the work of Skoulidas and Sholl.^528^

Conventional MD faces challenges in accurately estimating diffusivity for orders of magnitude below 10^–12^ m^2^/s^154,529^ due to time-scale limitations. For this reason, other, non-conventional approaches are followed, that mostly fall under the category of enhanced sampling methods, addressing the problem as an activated process.^530^ The most popular approach in the field is Transition State Theory (TST)^531^ which can be coupled with umbrella sampling^532,533^ to identify transition states of the propagation of H_2_ gas molecules in the cages and apertures of the nanoporous material. Dynamically corrected TST (dcTST)^531,533,534^ refines classical TST by applying a transmission coefficient (*κ*) to account for the fact that overcoming the energy barrier of the aperture does not always result in a successful cage–to–cage crossing, i.e., some jumps fail to thermalize in the destination cage and re–cross the aperture. *κ* (especially in the cases when it has a low value) can be efficiently computed following the approache of Ruiz-Montero et al.^530^ Such effects can be even more pronounced when moving from rigid to flexible simulations (although *κ* is also needed in simulations of rigid lattices^531,535^ ), since dynamic motions of groups located near the exit side of the aperture (e.g., rotating methyl groups in ZIFs or flexible linker tails) may intermittently obstruct or deflect the trajectory of the penetrating molecule. In dcTST, the diffusivity of H_2_ is approached by tracking “hops” of H_2_ molecules from cage to cage via crossing the framework’s apertures. After many attempts, a successful aperture crossing will be achieved. The success rate is then translated to the diffusivity via the following expression

22 $$D_{0}=\frac{1}{2n}k_{\mathrm{EXIT}}l^{2}$$

where *n* is the dimensionality of diffusion (e.g., for 3D materials in which H_2_ can diffuse in *x*, *y*, and *z* dimensions, *n* equals 3), *k*_EXIT_ is the total exiting rate of a molecule from a cage to any of the adjacent ones, and *l* is the distance between energy minima, which are the centers of cages. *k*_EXIT_ is estimated by

23 $$k_{\mathrm{EXIT}}=n_{\mathrm{apert}}k_{\mathrm{cross}}$$

where *n*_apert_ is the number of available exiting apertures in a cage and *k*_cross_ is the rate of successful crossings of a molecule (e.g., H_2_) through a given aperture, given by^536^

$$k_{\mathrm{crossing}}=\frac{1}{\sqrt{2\pi m}}P(\lambda^{*})$$ 24

where *m* is the mass of the molecule, *λ* is the reaction coordinate which can be regarded as a function of the Cartesian coordinates.^536^ *P*(λ*) is the probability of finding the molecule in the dividing surface, which is an orthogonal plane at *λ* = λ* vertical to the reaction axis, close to the energy barrier (or at the point where free energy is maximized). For the cases discussed in this review the energy barrier corresponds to the aperture’s center. *P*(λ*) depends on the free energy barrier and can be computed from

$$P(\lambda^{*})=\sqrt{\frac{k_{B}T}{2\pi m}}\frac{\exp^{-\beta F(\lambda^{*})}}{\int_{-\infty }^{\lambda^{*}}\exp^{-\beta F(\lambda^{*})}}$$ 25

where *F* is the free energy, which can be computed by umbrella sampling.^51,52,532^ *k*_crossing_ (Eq. ([Disp-formula eq24])) does not account for some seemingly successful jumps to a new cage, which end up again back to origin cage.^531^ To account for this, a dynamic correction factor must be calculated, called correction factor (*κ*). Thus, the actual, crossing rate is

26 $$k_{\mathrm{cross}}^{\mathrm{dc}}=\kappa k_{\mathrm{cross}}$$

Details on the umbrella sampling technique and the estimation of the correction factor *κ* can be found in the work by Krokidas et al.^418^

#### 3.2.6 Thermal Conductivity Using the WAVE Method

Cheng and Frenkel^537^ introduced the so-called WAVE method to calculate thermal conductivity of liquid H_2_ (and other systems) by analyzing density fluctuations rather than using the traditional GK method. According to the authors, this approach solves a significant limitation of GK methods when dealing with systems that have non-pairwise additive interactions (e.g., ML and DFT-derived potentials). The WAVE method uses Fourier components of density fields in the simulation cell, applies hydrodynamic theory that describes density fluctuations responsible for Rayleigh-Brillouin scattering, and uses the autocorrelation function of density waves to extract thermal conductivity. The width of the central Rayleigh peak in the power spectrum is proportional to thermal diffusivity (and thus thermal conductivity). In short, the scheme for computing thermal conductivity using the WAVE method involves the following elements: (i) Decomposing the density field into discrete Fourier components, (ii) analyzing the autocorrelation function or power spectrum of these components, (iii) fitting to hydrodynamic equations to extract conductivity *λ*(*k*), and (iv) extrapolating *λ*(*k*) to *k* = 0 to obtain the macroscopic thermal conductivity. Note that as stated by Cheng and Frenkel,^537^ electronic effects are not accounted for since heat transfer in insulators occurs mainly due to the motion of nuclei (electrons are in the ground state and adiabatically follow the nuclei). The authors showed that the electronic contribution to the thermal conductivity of H_2_ for the state points studied is negligible, i.e., ca. 0.002% of the computed value.

The method was validated with GK using a generalized LJ fluid over a wide range of temperature and density conditions (i.e., >20 different state points). The WAVE method only requires positional trajectories from MD simulations, making it usable as a post-processing step, there is no need to modify existing MD codes (classical or ab initio), while it works with any interaction potential, including many-body force fields, ML potentials, and DFT. According to the authors, the WAVE method eliminates the ambiguity in defining heat flux and atomic energy partitioning, and avoids potential issues with slowly decaying correlation functions in GK. Limitations of the WAVE method are the requirement of the system to be in the hydrodynamic regime (wavelengths larger than atomic dimensions), the possibility of large systematic errors due to extrapolations (although the statistical errors reported in ref 537) are small, and some discrepancies observed between GK and WAVE for bulk viscosity computations.

#### 3.2.7 Identification of Hydrogen Segregation Sites at Defects

The first task in studying H trapping and its interaction with defects is to identify the stable H segregation sites and the diffusion pathways that connect them. In perfect crystals, H occupies the classical tetrahedral (T) and octahedral (O) interstitials in bcc and fcc lattices respectively, and migrates along the minimum-energy paths between these sites as determined by Nudged Elastic Band (NEB) calculations. However, in highly distorted regions such as GBs or dislocation cores, these ideal sites no longer correspond directly to the actual trapping sites, making their identification non-trivial.

A broadly applicable strategy is the pure Voronoi-vertex sweep, i.e., one computes a full 3D Voronoi tessellation of the atomic network and treats every Voronoi vertex as a prospective H site. In fcc Ni, this automatically recovers all the T and O bulk interstices plus any irregular GB-specific voids;^538^ in bcc Fe, the same vertex list captures all T sites but must be augmented by the midpoints of each Fe-Fe next-nearest-neighbor bond to include the classical O sites before energy screening.^539^

By contrast, the polyhedral-centroid method first groups the GB region into its constituent convex packing units (e.g., tetrahedra, octahedra, pentagonal bipyramids, capped prisms) via a deltahedron-based tessellation, then places exactly one H candidate at the geometric center of each cell. This extra grouping step collapses thousands of raw vertices into a parsimonious set of physically meaningful “cages,” each labeled by its polyhedral type and directly linked to local volume dilation, greatly enhancing interpretability and computational efficiency.^540,541^

Wang et al.^542^ resort to a straightforward grid-scan of the GB region, placing H at regularly spaced points along and normal to the boundary and evaluating each binding energy. Although easy to implement, this brute-force line-grid approach is computationally intensive and its fixed spacing can overlook narrow or irregular traps, making it far less efficient and precise than geometry-driven methods like pure Voronoi sweeps or polyhedral-centroid tessellations.^542^

Having identified these distinct segregation sites, one can assemble the corresponding H-segregation energy spectrum for complex defect configurations, delineate the associated migration pathways, predict the equilibrium H distribution, and ultimately employ KMC simulations to model H diffusion.

#### 3.2.8 Hydrogen Trapping and Interactions with Defects

The energetics of H incorporation and trapping in a metal can be quantified in terms of two key quantities: (1) the solution energy and (2) the segregation (or trapping) energy. The energy to dissolve one H atom in the host crystal (relative to H_2_ gas) is

27 $$E_{\mathrm{sol}}=E_{\mathrm{host}+H}-E_{\mathrm{host}}-\frac{1}{2}E_{H_{2}}$$

where *E*_host+H_ is the total energy of the supercell containing a single interstitial H atom, *E*_host_ is the total energy of the pristine (defect-free) supercell, and *E*_H_2__ is the total energy of an isolated H_2_ molecule in vacuum. To assess the preference of H for a defect site versus a regular bulk interstitial, one evaluates

28 $$E_{\mathrm{seg}}=\left(E_{\mathrm{defect}+H}-E_{\mathrm{defect}}\right)-\left(E_{\mathrm{bulk}+H}-E_{\mathrm{bulk}}\right)$$

A negative value of *E*_seg_ indicates that H is energetically more stable at the defect site than in a bulk interstitial position.

Because the light mass of H gives rise to significant zero-point vibrational energy, one typically adds zero point energy (ZPE) corrections to the energies in the equations above. In practice, since the host and defect lattices contribute negligibly, the ZPE-corrected quantities reduce to^543,544^

29 $$E_{\mathrm{sol}}^{\mathrm{ZPE}}=E_{\mathrm{sol}}+E_{\mathrm{host}+H}^{\mathrm{ZPE}}-\frac{1}{2}E_{H_{2}}^{\mathrm{ZPE}}$$

and

30 $$E_{\mathrm{seg}}^{\mathrm{ZPE}}=E_{\mathrm{seg}}+E_{\mathrm{defect}+H}^{\mathrm{ZPE}}-E_{\mathrm{host}+H}^{\mathrm{ZPE}}$$

where each *E* ^ZPE^ is the sum of $\frac{1}{2}\hbar \omega_{i}$ over the three H vibrational modes. All of these energies can be obtained from zero-temperature (static) calculations by first relaxing the relevant structures and then performing vibrational analyses on the H atoms. Energy minimization is typically carried out with conjugate-gradient (CG) or fast inertial relaxation engine (FIRE) algorithms, either at the DFT level or using EAM or MLIP (see Section 3.1.5).

#### 3.2.9 Hydrogen Diffusion in Metals

MD simulations provide a powerful approach for studying how H migrates through lattices in metals under realistic thermo-mechanical conditions. In a typical HE MD study, one integrates Newton’s equations of motion for a large ensemble (10^3^-10^6^ atoms) using interatomic potentials parameterized to capture metal-H interactions, which range from EAM and MEAM to MLIP (see Section 3.1.5). H diffusion coefficients are then extracted from the MSD of H atoms via the Einstein relation (see Section 3.2.3). By performing simulations over a temperature range, one can obtain Arrhenius parameters (pre-exponential factor and activation energy). Periodic boundary conditions, a careful choice of time step, and sufficiently large simulation cells are crucial to minimize finite-size and sampling artifacts. Beyond pure lattice diffusion, MD naturally captures H trapping at microstructural features such as vacancies, dislocations, GBs, or free surfaces, enabling the observation of how these defects alter local diffusivity and cause transient trapping or “hopping” behavior.^538,545^

Path integral MD (PIMD), centroid MD (CMD) and ring-polymer MD (RPMD) represent three conceptually distinct ways to include nuclear quantum effects in atomistic simulations of H diffusion. In PIMD each H nucleus is replaced by a closed ring polymer of beads whose spring-bead system is thermostatted so as to sample the exact quantum canonical ensemble; because all beads move collectively, PIMD yields thermodynamic properties but does not correspond to real-time quantum dynamics. CMD and RPMD both build on the same ring-polymer framework but are tailored to approximate quantum dynamical observables: CMD propagates only the centroid (mean) position of each polymer under an adiabatic separation of internal modes, whereas RPMD evolves the full bead coordinates under Hamiltonian dynamics (with no thermostat on the real-time trajectories). Although the only formal differences among the three methods lie in the choice of fictitious bead masses and the thermostatting protocol, CMD and RPMD are based on different interpretations of how to extract time-correlation functions from the path-integral isomorphism. Both CMD and RPMD recover ordinary MD in the high-temperature limit, when the ring polymer collapses to a single point and quantum fluctuations become negligible.^465,546^

#### 3.2.10 Hydrogen-Mediated Dislocation Nucleation and Mobility

Atomistic simulations, MD and transition-state calculations via the NEB method, offer powerful insight into how H modifies both the nucleation of dislocations at crack tips or free surfaces and the mobility of existing dislocations. H atoms must first be introduced at concentrations and lattice sites that mirror the intended service or experimental conditions. Equally critical is constructing a simulation cell that faithfully reproduces the target geometry, stress distribution and boundary conditions. For example, nanoindentation studies use MD to drive an indenter into a crystalline surface while monitoring the characteristic “pop-in” event in the load-displacement curve, which marks the onset of dislocation nucleation beneath the indenter^547^ In a similar vein, constant-load compression of nanopillars with subsequent extended dynamic relaxation enables direct observation of dislocation emission from surfaces or edges under realistic loading rates and avoids artifacts associated with excessively high strain rates.^548,549^ To probe H’s effect on dislocation motion, MD simulations can measure the critical resolved shear stress required to mobilize pre-existing dislocations in H-charged lattices.^550^ In all cases it is essential to choose a sufficiently large simulation cell to eliminate spurious image interactions, apply thermostats and loading protocols that isolate the active deformation region, and select boundary constraints (fixed, free or periodic) that replicate experimental stress gradients. When these MD results are combined with climbing-image nudged elastic band (CI-NEB) calculations of the minimum-energy pathways for dislocation loop or half-loop formation, the integrated approach yields both activation energies and critical stress thresholds. Such insights are essential for quantifying how H alters the atomic mechanisms underlying fracture toughness and surface-driven plasticity.

#### 3.2.11 Hydrogen-Mediated Crack Propagation and Grain Boundary Decohesion

One of the most critical aspects in the study of HE mechanisms is the coupled study of crack propagation and GB decohesion in the presence of H. In this process, H diffusion and mechanical deformation interact continuously, i.e., stress concentrations at a crack tip or along a GB attract H, which in turn weakens interatomic bonds and alters the local stress field, accelerating crack advance and promoting intergranular fracture.^551^ Capturing this feedback loop requires chemo-mechanical models that combine H transport with fracture mechanics. Direct atomistic simulation of this coupling is particularly challenging because H diffusion is manifested on timescales (microseconds to seconds) far longer than typical MD loading rates (nanoseconds). As a result, most studies decouple diffusion and deformation by first establishing an equilibrium H distribution under a given stress field and then applying mechanical loading.

Two widely used strategies accomplish this without explicit H charging. In the Grand Canonical Monte Carlo (GCMC) method (see Section 3.3.2), H atoms are added or removed under a prescribed chemical potential (or gas pressure), allowing the system to reach equilibrium coverage at surfaces, GBs and crack-tip regions before loading begins.^552^ Alternatively, insertion-relaxation protocols emulate H arrival by placing individual H atoms at interstitial sites dictated by the local elastic field of the crack tip, then relaxing the entire system via MD to permit real-time diffusion into favorable sites. Song and Curtin^276^ used a self-consistent stochastic insertion algorithm which can be summarized as follows: At each step, the energy cost to add one more H at every empty interstitial is computed and translated into an equilibrium occupancy probability. Then randomly populate sites until no further H can be inserted at that stress intensity. Both approaches generate near-equilibrium H distributions around evolving crack tips without ever simulating H_2_ molecules, enabling subsequent MD or CI-NEB studies of crack advance and GB decohesion under realistic H coverages.

By choosing an appropriate method for generating H distributions under varying loads, one can study HE under more realistic, coupled conditions. GCMC is typically performed before any mechanical loading to establish equilibrium H coverages at surfaces, GBs and crack tips. Once deformation begins, whether via dislocation emission, cleavage or crack advance, it proceeds at MD strain rates many orders of magnitude higher than H diffusion rates, so further redistribution of H is negligible on the simulation timescale.^553^ The downside is that GCMC is computationally expensive, and therefore, impractical to run different simulations for each small loading increment. As a result, it cannot capture any load-induced changes in H segregation. In contrast, self-consistent stochastic insertion algorithms rebuild the H distribution after each incremental increase in stress intensity. Although this still does not track H diffusion in real time, it captures the instantaneous interplay between evolving stress fields and H trapping at every load step, providing a practical compromise between physical fidelity and computational cost.^276^

#### 3.2.12 Electroosmotic Drag Coefficient

Electro-osmotic drag (EOD) signifies the transport ratio of water to protons (H^+^) in a membrane.^92^ In hydrated membranes, H^+^ are conveyed across the membrane in response to an electric field. H^+^ do not migrate independently across the membrane; instead, they are stabilized as H_3_O^+^ or larger protonated clusters. Proton transport, therefore, occurs via the so-called proton hopping (Grotthuss mechanism)^554,555^ and vehicular mechanism.^556^ According to the vehicular mechanism, H^+^ move between water molecules involving coordinated movement of H_3_O^+^ ions and water molecules. This differs from the Grotthuss mechanism, where protons hop through a chain of water molecules by breaking and forming hydrogen bonds. The combined effect of these two mechanisms results in the manifestation of ionic conductivity in hydrated membranes. EOD is influenced by the level of hydration (*λ*) which indicates how many water molecules are associated with each ion exchange site in the membrane. For a Nafion membrane, hydration is defined as

31 $$\lambda =\frac{N_{H_{2}O}}{N_{{{\mathrm{SO}}_{3}}^{-}}}=\frac{N_{H_{2}O}}{N_{H_{3}O^{+}}}$$

where *N*_H_2_O_, and $N_{{\mathrm{SO}}_{3}^{-}}$ are the numbers of water molecules and sulfonic acid groups in the membrane, respectively.

The EOD coefficient, *ξ*_D_ is a measure of the number of water molecules transported alongside each proton within its solvation shell, and is defined as

32 $$\xi_{D}=\frac{j_{H_{2}O}}{j_{H^{+}}}$$

where *j*_H_2_O_ and *j*_H^+^_ are the fluxes of water molecules and protons, respectively. Note that *ξ*_D_ is defined in the absence of concentration and/or pressure gradients, and at vanishing chemical potential gradients of water.^92,100,557,558^ EOD coefficients are computed using NEMD, in which the driving force is imposed by an external electric field, **E**. The necessity for NEMD arises because the transport of water and ions are governed by both diffusion and the coupling of electrical and hydrodynamic forces. NEMD framework is therefore necessary, as the introduction of an electric field induces directional ion motion, which in turn influences water transport. In practice, the EOD coefficient is computed by applying external electric fields of varying magnitudes across one axis of the simulation box at a time and observing that ion velocities scale linearly with the field.^94^ By analyzing the system’s response to these external perturbations, the EOD can be extracted from the NEMD simulation in agreement with Eq. ([Disp-formula eq32]), from

33 $$\xi_{D}=\underset{E\to 0}{\lim}\frac{j_{H_{2}O}}{j_{H^{+}}}$$

where each flux is obtained at vanishing **E**, ensuring the sampling occurs in the linear response regime. This definition is in alignment with the broader framework introduced in Eq. ([Disp-formula eq9]).

In a classical MD simulation, where proton hopping is absent, the flux of H_3_O^+^ perpendicular to one of the faces of the simulation box along the x, y, or z axis can be expressed as the product of the number of H_3_O^+^ and their average velocity along the respective axis, sampled during the production runs. Similarly, the flux of water molecules can be expressed as the product of the number of water molecules and their average velocity.^94^ Consequently, Eq. ([Disp-formula eq33]) can be rewritten in terms of number density and velocity for the classical case where proton hopping does not occur

34 $$j_{H_{3}O^{+}}=N_{H_{3}O^{+}}\langle v_{H_{3}O^{+}}\rangle$$

where *j*_H_3_O^+^_ is the flux of hydronium ions across the membrane. A similar expression applies to the molecular flux of water. Using the definition of *λ* in Eq. ([Disp-formula eq31]), one can substitute *N*_H_2_O_=*λN*_H_3_O^+^_ into Eq. ([Disp-formula eq33]), leading to

35 $$\xi_{D}=\underset{E\to 0}{\lim}\frac{j_{H_{2}O}}{j_{H_{3}O^{+}}}=\lambda \times \underset{E\to 0}{\lim}\frac{\left\langle v_{H_{2}O}\right\rangle }{\left\langle v_{H_{3}O^{+}}\right\rangle }$$

Eq. ([Disp-formula eq35]) enables the direct computation of the EOD coefficient from MD simulations by tracking the velocities of water and H_3_O^+^ under an applied electric field. The EOD coefficient is determined by interpolating the average velocities as **E** approaches zero.^94^ It is evident that Eq. ([Disp-formula eq35]) represents a specific case of the more general formulation given in Eq. ([Disp-formula eq33]).

It is important to note that *ξ*_D_ can be computed using both classical simulations^94,127,559^ and first principles MD of small systems.^129^ The advantage of a first-principles simulation is that it captures the Grotthuss mechanism, in contrast to MD. Due to the fact that this mechanism becomes more dominant at higher water uptakes in the membrane, it is not a surprise that *ξ*_D_ of water computed via classical MD is in good agreement with experimental data^560^ at lower water uptakes^94^

#### 3.2.13 Thermodynamic Factor for Diffusion

The thermodynamic factor Γ for diffusion links MS and Fick diffusivities via Eq. ([Disp-formula eq16]). For an *N*-component mixture, the thermodynamic factor matrix **Γ** ^266,510^ is defined as

36 $$\Gamma_{ij}=\delta_{ij}+x_{i}{\left(\frac{\partial \ln\gamma_{i}}{\partial x_{j}}\right)}_{T,P,\sum }$$

where *x_i_* is the mole fraction, *γ_i_* the activity coefficient, *T* the temperature, *P* the pressure, and *δ_ij_* the Kronecker delta. The constraint *∑* enforces that during the differentiation, {*x_i_*} of all species remain constant, except for the *n* ^th^ component, so that Σ_*i*=1_^*n*^ *x_i_* = 1 ^266,507,510^ . The activity coefficient *γ_i_* is related to the chemical potential as follows^266,510^

37 $$\ln\gamma_{i}=\frac{\mu_{i}-\mu_{i}^{o}}{k_{B}T}-\ln x_{i}$$

where *μ*_*i*_^*o*^ is the chemical potential of pure component *i*. For a binary mixture, Eq. ([Disp-formula eq36]) reduces to a single thermodynamic factor Γ. Γ = 1 denotes an ideal mixture, while negative values indicate phase separation. In general, **Γ** for an *N*-component mixture is not symmetric,^267,510^ and for ideal mixtures (*γ_i_* = 1), Γ_*ij*_ = *δ_ij_*. **Γ** can be computed via molecular simulation in various ways; via Kirkwood-Buff integrals (KBIs),^561–565,565–570^ simulations at the grand canonical (GC) ensemble,^571^ and by using the Permuted Widom Test Particle Insertion (PWTPI) method.^572,573^ Each approach has advantages and drawbacks. For example, KBIs yield thermodynamic factors without relying on activity coefficient models but require large system sizes to obtain accurate RDFs, especially for *n* > 2 components.^506,574^ In contrast, PWTPI directly yields composition derivatives from a single simulation at a computational cost similar to WTPI,^51,575^ but like WTPI and GC methods, it is inefficient at liquid-like densities due to the low probability of successful single-step particle insertions.^51^ Recently, the CFCPWTPI method developed by Hulikal Chakrapani et al.^576^ was used to compute thermodynamic factors for dense CO_2_-H_2_ mixtures using small systems (i.e., containing a few hundred molecules). Such innovations improve the feasibility and reliability of using molecular simulations to directly realize the theoretical definitions of thermodynamic factors.

#### 3.2.14 Hydrate Phase Equilibria from the “Direct Phase Coexistence” Method

The “direct phase coexistence” method^577^ has extensively been used to calculate the phase equilibria of systems such as ice^367,368^ and hydrates.^342,369,578–581^ The different phases that are at equilibrium (e.g., solid hydrate (H)/ice (I), liquid water (W), gas (G)/vapor (V)) are brought in contact, and the evolution of the potential energy of the system is sampled via MD simulations at the *NPT* ensemble. By performing a temperature scan (i.e., recording the potential of the system for each temperature considered) for any given, fixed pressure, the three-phase equilibrium temperature can be identified, as the average between the lowest value that hydrate dissociation occurs and the highest value that hydrate formation occurs. To increase the accuracy of the calculation, the equilibrium temperature should be the result of averaging multiple MD runs (replicas).

Fig. 16 is a schematic representation of the various steps of the “direct phase coexistence” approach. Typical configurations for the simulations, encountered in the literature, are the three-slab (WHG) or four-slab (WHWG) arrangement. A hydrate supercell (*K* × *M* × *N*) of the hydrate structure of interest is constructed by using *K*, *M*, and *N* unit cells in the *x* −, *y* −, and *z* – directions, respectively. The positions of the oxygen atoms within the unit cell of the sII hydrate can be obtained from Mak and McMullan,^582^ while the corresponding positions for sI hydrates, from McMullan and Jeffrey.^583^ The positions of the hydrogen atoms can be found by minimizing the energy of the system while fixing the oxygen atoms, resulting in a structure that respects the Bernal and Fowler^584^ rules. Such an approach has been suggested^585^ as an alternative to finding a configuration that has the minimum dipole moment. Takeuchi et al.^586^ provided a detailed discussion for sI, sII, and sH hydrates. The authors used the TIP4P water model, and reported the coordinates of water molecules in the unit cell of hydrates that have nearly zero net dipole moment. The reported coordinates have been used in many molecular simulation studies, and have been further adapted for use with different water force fields.

**Figure 16:**
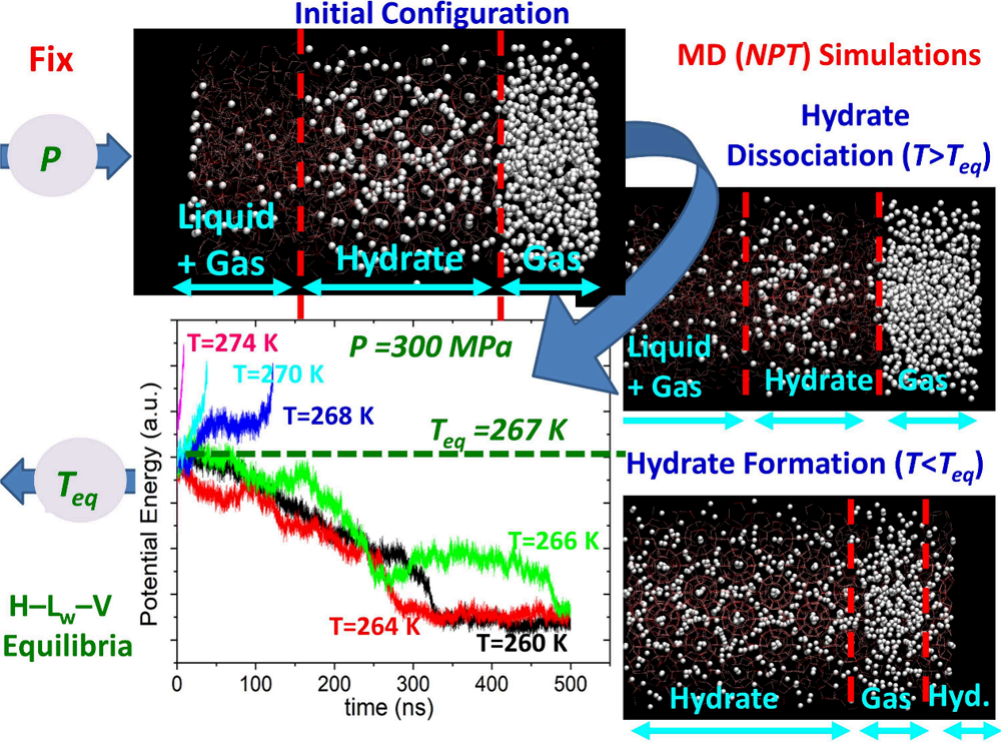
A schematic representation of the “direct phase coexistence” method. For a fixed *P*, an initial configuration (shown in the top left panel) consisting of three distinct slabs (i.e., (i) Liquid H_2_O + dissolved H_2_, (ii) hydrate crystal, and (iii) gaseous H_2_) is constructed to perform MD simulations (*NPT* ensemble). The potential energy of the total system is recorded for various temperatures (shown in bottom left panel). For *T* > *T*_eq_ (where *T*_eq_ is the equilibrium temperature) the potential energy increases, and the hydrate dissociates (top right panel), while for *T* < *T*_eq_ the potential energy decreases, and the hydrate phase grows (bottom right panel). For the shown *P*, *T*_eq_ = 267 K is identified as the average between the lowest value (268 K) at which hydrate dissociation occurs and the highest value (266 K) at which hydrate formation occurs.

All the centers of the cages can be filled with a single guest molecule (i.e., 100% (full) cage occupancy). Alternatively, experimental measurements or MC simulations can be used for cases of examining partial (i.e., lower than 100%) or multiple (i.e., higher than 100%) cage occupancy. A typical procedure followed during these simulations involves the following steps: Initially, each slab is equilibrated separately at each different pressure. Next, all different slabs are connected with a buffer distance, followed by energy minimization to avoid overlaps, while keeping the oxygen positions frozen. The connection of the slabs is achieved with their interfaces normal to the *x*-direction. During the equilibration of the water and H_2_ slabs, their *y* and *z* dimensions are kept equal to the equilibrated *y* and *z* dimensions of the hydrate slab.

#### 3.2.15 Hydrate Structure and Kinetics

A number of methods have been reported for the calculation of the growth rate of hydrate crystals based on MD simulations. Such methods include: (i) the counting of the water molecules added to the hydrate phase,^587^ (ii) plotting the potential energy as a function of time (until it reaches constant value) in order to estimate the growth time *τ*. The growth rate, *u*, is then simply obtained by dividing the length of the grown hydrate, Δ*L*, by 2*τ* due to the fact that there are two interfaces growing under periodic boundary conditions,^588^ and (iii) tracking of the position of the hydrate-liquid interface.^589,590^ To identify the type of structure each water molecule belongs to, order parameters have been defined. The order parameters can describe the hydrogen bond network within the water. In particular, *F*_3_ expresses deviations from the tetrahedral angle within the hydrogen bond network.^591^ An angle *θ* can be defined between the oxygen atoms in a *B_i_* – *A* – *B_j_* triad and then used to calculate *F*_3_ through the equation

38 $$F_{3}=\left\langle {\left(\cos\theta |\cos\theta |+\cos^{2}\theta_{t}\right)}^{2}\right\rangle$$

where *θ_t_* is the tetrahedral angle (109.47^*o*^). The angular brackets denote an average over all unique pairs *B_i_*, *B_j_* and over all the designated central atoms *A*. Essentially, the order parameter probes oxygen atoms within the first solvation shell of the central oxygen atom (i.e., less than 0.35 nm from oxygen A). *F*_3_ will be close to zero for tetrahedrally coordinated water and larger than zero otherwise.^592^

For the analysis of hydrate crystals Rodger et al.^593^ concluded that it would be useful to have an order parameter that can distinguish between the different tetrahedral networks adopted by water, and introduced a new, four-body order parameter (*F*_4*ϕ*_) based on the H-O–O-H torsion angle, *ϕ* for two adjacent water molecules; for these purposes the hydrogen atoms have been defined as the outer-most H in the water dimer. (*F*_4*ϕ*_) is defined as

39 $$F_{4\phi }=\left\langle \cos3\phi \right\rangle$$

Therefore, by comparing the set of instantaneous order parameters for any specific water molecule with the distribution of parameters found within various stable phases (see Table 8), it is possible to identify the local phase to each water molecule, and thereby to determine both the hydrate content of a system and the extent to which the water molecules with hydrate local phase are clustered.

**Table 8:** Order Parameters for Water Molecules in Various Phases

Phase	*F*_3_	*F*_4*ϕ*_
Liquid	0.1	–0.04
Hydrate	0.01	0.7
Ice	0.01	–0.4

Alternatively, some studies considered the time evolution of the number of 5^12^ or 5^12^6^4^ cages to track the hydrate growth rate. In particular, in two H_2_-hydrate-related studies (i.e., H_2_ + CH_4_;^594^ H_2_ + THF^595^), the hydrate growth process was monitored with the face-saturated incomplete cage analysis method (FSICA).^596^ By performing an analysis of the network topology formed by the hydrogen bonds of water molecules, FSICA can identify all of the face-saturated hydrate cages in the system, where face-saturation means that the edge formed between two water molecules is shared by at least two faces (water rings).^596^ Zhang et al.^594^ showed that the calculation of the growth rate, either using the evolution of the number of 5^12^ cages, or using the evolution of the number of 5^12^6^4^ cages produce identical results for the hydrate crystal growth rate. The hydrate growth rate in these studies was calculated using the following equation:

40 $$R=\frac{N_{500\mathrm{ns}}-N_{100\mathrm{ns}}}{L_{x}L_{y}400}$$

where *N*_100ns_ and *N*_500ns_ represent the number of 5^12^ cages in the system at 100 and 500 ns of simulation duration, respectively. *L_x_* and *L_y_* represent the *x* – and *y* – direction lengths of the system box, respectively. Δ*t* = 400 is the time span between the two measurements.

#### 3.2.16 Interfacial Tension

Let us consider a planar slab of fluid with a planar area $A={L_{∥}}^{2}$ , in contact with a gas inside a simulation box with dimensions *L*_∥_, *L*_∥_, *L*_⊥_ in the *x*, *y*, *z* directions (box volume: *V* = *L*_∥_^2^×*L*_⊥_), respectively. The thermodynamical definition of the interfacial tension of gas-liquid (or a liquid-liquid) interface in the canonical (*NVT*) ensemble is the change in the Helmholtz free-energy (*F*) of the system due to the change in the surface area of the interface^51,52,597^

41 $$\gamma ={\left(\frac{\partial F(N,V,T)}{\partial A}\right)}_{N,V,T}$$

where the composition, volume, and temperature of the system are fixed. Interfacial tension in a system with fixed pressure, temperature, and composition is given by^51,52,597^

42 $$\gamma ={\left(\frac{\partial G(N,p,T)}{\partial A}\right)}_{N,p,T}$$

where *G*(*N*, *p*, *T*) is the Gibbs free energy of the system.

The mechanical definition of the surface tension using the microscopic pressure tensor for a planar fluid slab coexisting with a gas as proposed by Irving and Kirkwood is^597–599^

43 $$\gamma =\int_{-\infty }^{\infty }dz\left[p_{⊥}(z)-p_{∥}(z)\right]$$

where *p*_⊥_ and *p*_∥_ follow from

44 $$p_{⊥}=p_{zz}$$

and

45 $$p_{∥}=\frac{1}{2}(p_{xx}+p_{yy})$$

The integrand of Eq. 43 is non-zero only in the vicinity of interface separating the two phases and zero otherwise.^51,52,597^ As stated in the review by Ghoufi et al.^597^, the definition of *γ* from the thermodynamic route (Eq. ([Disp-formula eq41])) and the mechanical route (Eq. ([Disp-formula eq43])) are formally equivalent. It is important to note that Eq. ([Disp-formula eq43]) is valid for continuous pairwise-additive potentials.^51,52,597^ It is readily shown that,

46 $$\int dzp_{\alpha \beta }=L_{⊥}\langle p_{\alpha \beta }\rangle$$

where

47 $$p_{\alpha \beta }=\rho k_{B}T{\left(I\right)}_{\alpha \beta }+\frac{1}{V}\langle \sum_{i=1}^{N-1}\sum_{j>i}^{N}{\left(r_{ij}\right)}_{\alpha }{\left(f_{ij}\right)}_{\beta }\rangle$$

where *ρ* = *N*/*V* is density, **I** is the identity matrix, **r**_*ij*_ and **f**_*ij*_ are the separation vector and the intermolecular force between molecule *i* and *j*, *α*, *β* = *x*, *y*, *z* and ⟨··· ⟩ is the canonical (Boltzmann) average over the simulation run. Using Eq. ([Disp-formula eq46]) the interfacial tension in Eq. ([Disp-formula eq43]) can be rewritten as follows

48 $$\gamma =\frac{L_{⊥}}{2}\langle p_{⊥}-p_{∥}\rangle$$

where the factor 2 in the denominator signifies the presence of two interfaces. Equation 48 is also called the Kirkwood-Buff definition.^597,600^

#### 3.2.17 Solid Gas/Liquid Contact Angle

Contact angles provide a quantitative measure of how a liquid, solid, and gas interact at their three-phase contact line.^52^ As shown in Fig. 17, the contact angle is defined as the angle formed between the tangent to the liquid-gas interface and the solid surface at the point where they meet. Contact angle characterizes the wettability of the surface, i.e., small contact angles indicate a surface that is easily wetted, while large ones suggest poor wettability. By measuring contact angles, one can gain insights into surface chemistry, material properties, and fluid-solid interactions. Below, we describe how to compute contact angles for gas bubbles in MD simulations.

**Figure 17:**
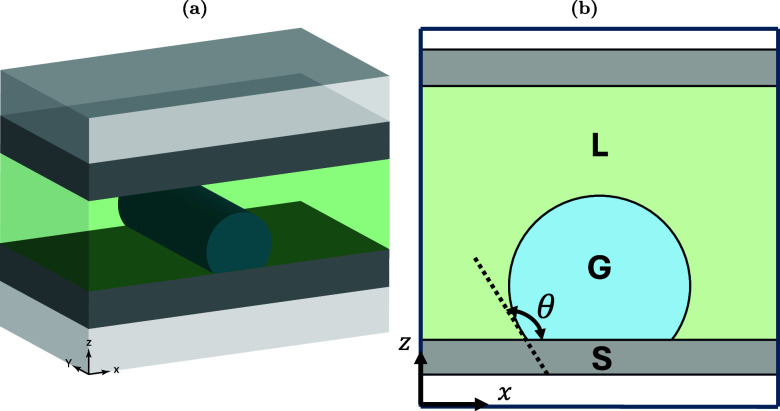
(a) A typical MD simulation setup for computing contact angles: A hydrogen nano-bubble (dark green) immersed in water/brine (transparent green), shaped cylindrically due to symmetry along the *y*-direction, is enclosed between solid surfaces (dark gray) at the top and bottom. The top wall serves as a piston (or barostat) to control gas-liquid pressure.^601,602^ To prevent wall interactions across periodic boundaries, the simulation box length in the *z*-direction is (typically) several times longer than lateral dimensions.^602^ The regions (in light gray) above the top wall and below the bottom wall are vacuum. (b) A cross section of the simulation box in the *x* – *z* plane. The intersection between the solid surface (S), surface of the nanobubble (G) and the liquid medium (L) co-determines the contact angle *θ*.

To compute contact angles, MD simulations are performed in the *NVT* ensemble adopting the setup shown in Fig. 17(a).^602–609^ A cylindrical slab composed of H_2_ gas spanning the *y* – direction is surrounded by water while directly contacting a “solid” slab beneath it. Typically, a gas nanobubble spanning the *y* – direction is chosen to avoid the effects of line tension on the contact angle. By eliminating effects of line tension on the contact angle, a comparison with experiments is possible. Introducing another solid slab positioned at a distance *w* from the bottom slab confines the gas-liquid medium. Adjusting the distance *w* allows for variation in the confining pressure, effectively serving as a barostat, as depicted in Fig. 17(a). The volumes between the lower surface of the lower solid slab and the upper surface of the upper solid slab are vacuum. To ensure that the slabs do not interact via the periodic boundary in the *z*-direction the top and bottom wall are positioned far from each other. Finally, the gas nanobubble is equilibrated for a duration of several ns, after which the contact angle can be computed.

To determine the contact angle, 2D *xz* density maps (see Fig. 17(b)) are obtained by a binning procedure wherein the center-of-mass of the gas nanobubble in the simulation box are segregated into bins of a (typical) size of 0.5 × 0.5 Å^2^, based on their position in the *xz* plane. Density maps are averaged over several ns to obtain a smooth density profile. A non-linear regression is performed to fit the iso-density curve of the gas nanobubble to a circle, and its intersection with the position of the wall in the *z* direction determines the contact angle, as shown in Fig. 17(b). For instance, the position of the wall in the *z*-direction is determined by averaging the positions of the outermost silica atoms if the wall is composed of silica as in the study by Chen and Xia.^605^ The contact angle of a water droplet is the supplementary angle corresponding to the contact angle of the gas nanobubble, i.e., *θ*_H_2_O_=180-*θ* in Fig. 17(b). A note of caution to the reader: in the absence of any pinning sites (or heterogeneities) the gas nanobubble tends to translate parallel to the wall, and its center of mass may vary with time. Thus, when generating density maps, it is important to reposition the the gas nanobubble about its center of mass.

### 3.3 Computation of Hydrogen Properties Using Monte Carlo Simulations

The past decades, MC simulations have been evolved into a powerful tool for computing thermodynamic properties of reactive and non-reactive H_2_ systems. Representative examples of such properties are phase coexistence, chemical reactions, solubilities (in gas, liquid, and solid phases), densities, and chemical potentials. As in MD simulations, the interactions between atoms and molecules are determined based on the chosen force fields. Unlike MD simulations, MC simulations use a stochastic algorithm. The concept of ”trial-moves” is used in MC simulations to sample the phase-space. Examples of trial-moves are displacements and rotations of molecules, particle insertions (or deletions), volume changes, and changes in bond lengths.^51,52^ Trials moves can be accepted (or rejected) based on a probability (*P_i_*), which is often proportional to the probability of occurrence of the given micro-state (i.e., metropolis algorithm). Trial moves can also be more complex, such as identity swaps, where one particle type is replaced by another. The flexibility of MC methods is a significant advantage compared to MD. MC simulations typically involve thousands to millions of cycles, where each cycle consists of *N* trial moves. The number of cycles needed depends on the property being studied, the size of the system, and the complexity of the force field. The properties listed in this section such as excess chemical potentials and solubilities can also be computed using MD. However, they are most commonly computed using MC simulations. For all details regarding MC simulations and statistical mechanics the reader is referred to the relevant textbooks.^51,52,296,298^

As for MD simulations, several high-quality open-source software packages have been developed through the years for performing MC simulations of H_2_ (and other) systems. Prime examples are Towhee,^610^ Cassandra, RASPA,^480^ and BRICK-CFCMC,^611,612^ however, the list of available codes is long. For a relevant discussion the reader is referred to ref 613. Visualization of MC simulations is an important feature allowing for both understanding of mechanisms and tracing of possible simulation errors. Examples of state-of-the-art visualization software for molecular simulations is discussed in Section 3.2.

Kinetic Monte Carlo (KMC) is a simulation technique that is used to model the time evolution of physical systems through stochastic processes, focusing on the dynamics and rates of transitions between states.^614,615^ Unlike classical MC (described above), which primarily samples equilibrium configurations using acceptance probabilities based on energy differences, KMC explicitly tracks the temporal evolution of the system with transition rates, and associates time with each simulation step. KMC works by selecting transitions proportionally to their rates rather than attempting moves with uniform probability followed by accept/reject decisions, making it particularly well-suited for studying non-equilibrium processes and kinetic phenomena while classical MC excels at determining equilibrium thermodynamic properties. While MD simulations (see Section 3.2) are well-suited for studying fast processes such as the dislocation motion, they are limited in their ability to capture the long-timescale diffusion and segregation of H_2_ in complex micro-structures. KMC methods complement MD by enabling the simulation of H_2_ transport over experimentally relevant timescales (up to seconds or longer) while maintaining atomic-level resolution. In this review, we briefly describe properties computed with KMC related to HE.

#### 3.3.1 Phase Equilibria of Hydrogen Systems

Based on vapor-liquid equilibrium (VLE), the solubilities of H_2_ in different liquid mixtures, densities, and equilibrium gas compositions can be determined. Equilibrium is established when the chemical potentials of both phases are equal. At this point, the densities of each phase become stable, fluctuating around well-defined values. To compute VLEs of H_2_ mixtures from molecular simulations, different methods can be used.

##### 3.3.1.1 Gibbs Ensemble

A common approach for computing the VLE of a system with MC simulations is the Gibbs Ensemble (GE) method introduced by Panagiotopoulos^616^ in 1987. Unlike most MC and MD methods, GE considers two distinct simulation boxes (without any interface), each containing the gas or the liquid phase. GE enables the determination of phase coexistence properties of multi-component systems from a single simulation. To achieve equilibrium, a sufficient number of MC trial moves are performed to generate random configurations within each phase, along with the random transfer of molecules between them.^51^ To assess whether the chemical potentials of the two phases have equilibrated, test particle insertion methods^617^ are often employed. When one of the phases has a high density (e.g., liquid water at room temperature), VLE computations can become inefficient. This is primarily due to the frequent rejection of molecular exchanges between the simulation boxes in conventional single-molecule trial insertion and removal moves. In such cases, the Continuous Fractional Component Gibbs Ensemble (CFCGE) approach (GE expanded with fractional molecules) can be used to facilitate the equilibration of phase compositions (i.e., sampling densities at equilibrium) while also enabling direct sampling of chemical potentials.^618^ This approach will be discussed further in Section 3.3.1.4.

##### 3.3.1.2 Vapor–Liquid Equilibrium from Chemical Potentials

Although GE and CFCGE methods provide a straightforward approach to compute the VLE of H_2_ systems, the chemical potential of each phase (or species) can also be independently sampled in separate simulations to determine VLE compositions. Rahbari et al.^110^ performed CFCGE simulations to compute the VLE of H_2_O – H_2_ systems at high pressures. At low H_2_ concentrations, either a simulation box with a large number of molecules is required or extended simulation cycles to adequately sample the equilibrium composition in each phase. In such cases, separate simulations for each phase are recommended to determine the chemical potential for phase equilibrium.

Accurately computing chemical potentials is crucial for obtaining solubilities and VLEs of H_2_ systems using molecular simulations. The chemical potential of H_2_ in each phase can be expressed as

49 $$\mu_{H_{2}}=\underset{\text{ideal gas part}:\mu_{H_{2}}^{\mathrm{id}}}{\underset{︸}{\mu_{H_{2}}^{0}(T)+k_{B}T\ln\left(\frac{\left\langle \rho_{H_{2}}\right\rangle }{\rho_{0}}\right)}}+\mu_{H_{2}}^{\mathrm{ex}}$$

where *μ*_H_2__^0^(*T*) represents the reference chemical potential, incorporating intramolecular contributions such as rotation, vibration, translation, and bond dissociation energy. The number density, *ρ*_H_2__, is defined as the number of molecules per unit volume, with *ρ*_0_ = 1 Å^–3^ serving as a reference to ensure a dimensionless logarithm. *μ*_H_2__^0^(*T*) is relevant when chemical reactions (bond breaking/formation) are considered in the simulations. The first two terms in Eq. ([Disp-formula eq49]) correspond to the ideal gas contribution (*μ*_H_2__^id^), while the final term represents the excess chemical potential (*μ*_H_2__^ex^), which quantifies the difference in the Gibbs free energy associated with the addition of a molecule or species of type *i* into an interacting molecular system compared to an ideal gas at the same temperature, pressure, and composition.^619^ Different methods can be used to compute *μ*_H_2__^ex^, and therefore, VLE and solubilities, in molecular simulations. Brief overviews of these methods are provided below.

##### 3.3.1.3 Widom Test Particle Insertion Method

The Widom test particle insertion (WTPI) method, along with its adaptations, is widely used for computing *μ*_H_2__^ex^ in both MC and MD simulations.^51,572,573,576,617,618,620–623^ *μ*_H_2__^ex^ is sampled using the WTPI method as

50 $$\mu_{H_{2}}^{\mathrm{ex}}=-k_{B}T\ln{\left\langle \exp\left[-\beta \Delta {U_{H_{2}}}^{+}\right]\right\rangle }_{NVT}$$

where Δ*U*_H_2__^+^ is the energy change of the system when a H_2_ probe molecule (or any other species) is randomly inserted in the system. One should be aware that Eq. ([Disp-formula eq50]) faces challenges when simulating high-density systems or systems with a strong dependence on molecular orientation.^54,624,625^ Variations of WTPI, in which the test particle is gradually inserted, are commonly used in expanded ensemble methods (in both MC and MD) to avoid the drawbacks of the single-step insertions.

##### 3.3.1.4 Continuous Fractional Component Monte Carlo

The CFCMC^626,627^ method is an adaptation of the expanded ensembles concept in MC simulations. A thorough review of this method is provided in.^618^ In CFCMC, conventional ensembles such as *NPT*, *NVT*, GE or the reaction ensemble (RxMC), are expanded with a fractional molecule or a group of fractional molecules with scaled interactions. This leads to improving efficiency in cases where one phase has a high density or strong electrostatic interactions. Simultaneously, the excess chemical potentials of species in both phases are obtained. The interactions of the fractional molecules are scaled with a coupling parameter *λ* ∈ [0, 1].^627–629^ At *λ* = 0, the fractional molecule does not interact with the rest of the molecules in the system, while at *λ* = 1, the fractional molecule has fully scaled interactions. Staged insertions are performed during the simulation using random trial moves in *λ* space by increasing or decreasing the value of *λ*. To improve sampling in *λ* space (especially in dense systems, such as aqueous solutions), a biasing weight function is often applied^112,630^ using the Wang-Landau algorithm.^631^ Provided that sufficient sampling is performed at *λ* = 0 and *λ* = 1, *μ*_H_2__^ex^ in the *NVT* or *NPT* ensemble follows from^627,632^

51 $$\mu_{H_{2}}^{\mathrm{ex}}=-k_{B}T\ln\left(\frac{p(\lambda_{H_{2}}=1)}{p(\lambda_{H_{2}}=0)}\right)$$

where *k*_B_ is the Boltzmann constant, and *T* is the temperature in K. *p*(*λ*_H_2__ = 1) and *p*(*λ*_H_2__ = 0) are the probabilities of the coupling parameter *λ* of H_2_ fractional molecules being equal to 1 and 0, respectively.

Thermodynamic integration is an alternative method for computing excess chemical potentials in CFCMC simulations or in similar adaptations of expanded ensembles:^618^

52 $$\mu_{H_{2}}^{\mathrm{ex}}=\int_{0}^{1}\left\langle \frac{\partial U(\lambda_{H_{2}})}{\partial \lambda_{H_{2}}}\right\rangle d\lambda_{H_{2}}$$

In dense electrolyte solutions, performing random walks in the *λ* space can become difficult because of the strong electrostatic interactions in the mixture.^612^ In such cases, it is difficult to sufficiently sample the *λ* space even using biasing weight functions constructed by the Wang-Landau algorithm. This is especially the case when computing *μ* ^ex^ of ions, such as Na^+^.^612^ Thermodynamic integration can therefore be used in multiple simulations for fixed values of *λ* ∈ [0, 1] to compute the excess chemical potentials.^612^

##### 3.3.1.5 Solubility of Hydrogen in the Liquid Phase

The solubility of H_2_ in the liquid phase can be computed based on the excess chemical potential of H_2_ in the liquid phase. The ideal gas chemical potential of H_2_ (and therefore the number density) in the liquid phase is obtained by imposing equal chemical potentials of H_2_ in the gas phase and the liquid phase $\left(\mu_{{H_{2}}_{\left(g\right)}}=\mu_{{H_{2}}_{\left(l\right)}}\right)$ . The solubility of H_2_ in the liquid phase follows from^47,110^

53 $$\frac{\left\langle \rho_{{H_{2}}_{\left(l\right)}}\right\rangle }{\rho_{0}}=\exp\left[\frac{\mu_{{H_{2}}_{\left(l\right)}}^{\mathrm{id}}-\mu_{H_{2}}^{0}}{k_{B}T}\right]=\exp\left[\frac{\mu_{{H_{2}}_{\left(g\right)}}-\mu_{{H_{2}}_{\left(l\right)}}^{\mathrm{ex}}-\mu_{H_{2}}^{0}}{k_{B}T}\right]$$

At low fugacities of H_2_, the solubility of H_2_ can also be computed using Henry coefficients (*H*):

54 $$\frac{\left\langle \rho_{{H_{2}}_{\left(l\right)}}\right\rangle }{\rho_{0}}=\underset{f_{H_{2}}\to 0}{\lim}\frac{f_{H_{2}}}{H}$$

The Henry coefficient of H_2_ can be computed directly based on $\mu_{{H_{2}}_{\left(l\right)}}^{\mathrm{ex}}$ :

55 $$H=\rho_{0}k_{B}T\exp\left[\frac{\mu_{{H_{2}}_{\left(l\right)}}^{\mathrm{ex}}}{k_{B}T}\right]$$

At sufficiently low H_2_ densities, the ideal gas law can also be used (i.e., *f*_H_2__=*P*_H_2__=*ρ*_H_2__ *k*_B_ *T*). This significantly simplifies the computation of H_2_ solubilities compared to Eq. ([Disp-formula eq53]), as no simulations are needed for the gas phase.

##### 3.3.1.6 Grand Equilibrium Method

The grand equilibrium method computes VLE for multi-component systems by equilibrating the liquid phase in the *NPT* ensemble, where the chemical potential is sampled using Widom’s test particle insertion method (explained in Section 3.3.1.3).^48^ From the chemical potential, the Henry constant is calculated at infinite dilution. The chemical potential is then fitted as a function of pressure (*μ*(*P*)) at constant temperature and mixture composition and approximated using a Taylor series expansion. This function is subsequently used in a pseudo-*μVT* ensemble simulation of the vapor phase to sample the vapor pressure. Köster et al.^48^ applied this method to compute VLE for H_2_ multi-component systems.

##### 3.3.1.7 Fugacity Coefficient

The fugacity coefficient of H_2_ can be computed from the *μ*_H_2__^ex^ or probability distribution of *λ* (see Eq. ([Disp-formula eq51])) in a mixture using

56 $$\phi_{H_{2}}=\frac{\exp\left[\frac{\mu_{H_{2}}^{\mathrm{ex}}}{k_{B}T}\right]}{Z_{\mathrm{mix}}}=\frac{1}{Z_{\mathrm{mix}}}\times \frac{p\left(\lambda_{H_{2}}=0\right)}{p\left(\lambda_{H_{2}}=1\right)}$$

where $Z_{\mathrm{mix}}=\frac{P\langle V\rangle }{N_{t}RT}$ is the compressibility factor of the mixture, and *N*_t_ is the total number of moles in the mixture.

#### 3.3.2 Hydrogen Adsorption onto Nanoporous Materials

The Grand Canonical Monte Carlo (GCMC) method (system at constant *μ*,*V*, *T*) is widely used to study gas adsorption in nanoporous media such as polymers, MOFs, COFs, zeolites, and CNTs.^51,52,317^ In GCMC, a simulation box with constant volume containing the adsorbent (e.g., solid) is used,^51,52^ assuming contact with an infinite reservoir (not explicitly considered) that holds the adsorbate (e.g., H_2_ or gas mixture) at a specified temperature, pressure, and composition. Gas molecules are randomly inserted and deleted (adsorbed/absorbed and desorbed) onto the material surface or in the pores. The chemical potential of each component in the reservoir (and therefore the solubility of the adsorbate), *μ_i_* (*p*, *T*, *x*), can be computed using an EoS or WTPI method (see Section 3.3.1.3) in the *NPT* ensemble. This ensemble can also be extended with fractional H_2_ molecules to compute solubility, phase coexistence, and equilibrium properties more efficiently, following the principles outlined in Section 3.3.1.4

An alternative way to compute the solubility coefficient is to use the WTPI method in MD trajectories of only the adsorbent (e.g., MOF, ZIF). The MD simulation generates representative host configurations (usually in the order of 10^3^ simulation snapshots). In each of these frames, a sole molecule of the gas under investigation is randomly inserted at various positions, to get adequate statistics on the conformations of the guest-host system, and at each insertion the energy of the system is calculated. An average over these different conformations yields *μ*_*i*_^ex^ of the inserted species according to Eq. ([Disp-formula eq50]). The solubility, *S_i_*, of component *i* (e.g., H_2_), can be then obtained from

57 $$S_{i}=\frac{22400{\mathrm{cm}}^{3}\mathrm{STP}}{\mathrm{mol}}\frac{1}{RT}\exp\left(-\frac{\mu_{i}^{\mathrm{ex}}}{RT}\right)$$

As already shown in Eq. ([Disp-formula eq56]) in Section 3.3.1.7, the excess chemical potential can lead to fugacity. This facilitates the production of adsorption isotherms, by linking the fugacity to the number of existing sorbed molecules.

#### 3.3.3 Hydrogen Adsorption and Separation of Gas Mixtures in Hydrates

As discussed in the previous section, GCMC simulations are widely used to compute gas adsorption in solid nanoporous materials. Considering that gas hydrates have a fixed and well defined geometry, enables simulating their formation as a process of adsorption of H_2_ gas inside a porous solid material (i.e., the different cages are considered as effective pores). Namely, the hydrate lattice is treated as a rigid porous solid substrate (i.e., H_2_O molecules are fixed in their positions, not allowed to move) where an adsorption site can be positioned anywhere within a hydrate cage belonging to the hydrate lattice. To this end, MC simulations have been performed with either in-house or open-source software (e.g., MCCCS Towhee^610^). While studies using GCMC simulations consider H_2_O molecules fixed in space, Brumby et al.^633^ reported MC simulations in the GE (Section 3.3.1.1), allowing isotropic volume change moves (i.e., H_2_O movement) in addition to the regular MC moves, and reported good agreement with the counterpart GCMC studies.^248^

To determine the average occupancy *θ* (i.e., average number of H_2_ molecules per cavity) for every cavity type, each H_2_ molecule is assigned to the cavity center whose center is the closest. For a hydrate structure that contains *k* types of cavities with *n_i_*_,*k*_ cavities of type *i* together with *N*_w_ water molecules in a single unit cell, the gas (H_2_) content, expressed as weight percentage (wt.%), is given by

58 $$(\mathrm{wt}.\%)=\frac{MW_{g}\cdot \sum_{i=1}^{k}n_{i,k}\cdot \theta_{i}}{MW_{g}\cdot \sum_{i=1}^{k}n_{i}\cdot \theta_{i}+N_{w}\cdot MW_{w}}\times 100$$

where *MW*_g_ and *MW*_w_ are the molecular weights of the guest gas (g) and water (w), respectively. In the more general case where a promoter (i.e., guest that contributes in hydrate formation under more favorable *P* and *T* conditions) is also present in the hydrate structure, the term *n_p_* ·*θ_p_* · *MW*_p_ needs to be added to the denominator of Eq. ([Disp-formula eq58]), where it is assumed that the promoter has a molecular weight *MW_p_* and occupies the cavities of type *p*, with an average occupancy *θ_p_*. Usually it is assumed that all of the promoter-designated cavities are fully occupied by a single promoter molecule, therefore, *θ_p_* = 1.

The basic input for a GCMC run is the chemical potential of the adsorbate, which can be calculated via different methods. During the earlier hydrate-related MC studies, a simpler approach was utilized that was based either on (i) the use of general-purpose, cubic EoS,^634–637^ which largely failed at computing the chemical potentials of H_2_ at the conditions of interest for storage in hydrates since they were not developed using H_2_ data, or (ii) on component-specific^638,639^ EoS, which partially solved this problem. Later MC studies^248,341,640,641^ used a more refined approach for the calculation of the chemical potential and the correlation of chemical potential to the pressure of the system. In particular, a series of MC simulations of bulk H_2_ in the *NVT* ensemble is performed from which the virial pressure is calculated. In a subsequent step, the excess chemical potential is calculated with WTPI^617^ as explained in Section 3.3.1.3. The reference value of chemical potential, *μ*_0_, is calculated from an additional *NVT* run where all interactions are set to zero. The chemical potential is, thus, calculated as a function of pressure, at a constant temperature. This approach is more reliable and self-consistent than using EoS, however, it requires additional computational resources. In this way, each run corresponds to a certain H_2_ density and for this density the pressure and chemical potential are calculated from the appropriate equations. This procedure allows for the determination of the relation between pressure and chemical potential, while simultaneously, provides the EoS for H_2_. Additional details regarding the methodology can be found in refs 248 and 641.

The separation of gas mixtures (e.g., H_2_/CH_4_ and H_2_/CO_2_) via hydrate formation is based on the principle that the different components of the gas mixture have a different distribution within the hydrate crystal compared to the original mixture composition. Therefore, a series of GCMC simulations can be performed at different *P* and *T* conditions to calculate the separation efficiency of the hydrate-based process. The simulation protocol is identical to the one described for the calculation of the hydrate gas storage capacities, applied however for the gas mixture, instead of the pure H_2_ gas.

#### 3.3.4 Heat Capacities, Thermal Expansivity, Joule–Thomson Coefficient, and Speed of Sound from Ensemble Fluctuations

Thermal expansivity (*α_P_*), isothermal compressibility (*β*_T_), isobaric heat capacity (*C_P_*), isochoric heat capacity (*C_V_*), and the Joule-Thomson coefficient *μ*_JT_ are derivative properties defined in classical thermodynamics as pressure or temperature derivatives.^571,642,643^ These thermodynamic properties of H_2_ gas or for multi-component H_2_ systems at high pressures are computed using the so-called ensemble fluctuations in molecular simulations.^47,51^ For the computation of heat capacity, ones needs to consider both the ideal and the residual contributions^644,645^

59 $$C_{P}(T,P)={\left(\frac{\partial \left\langle H\right\rangle }{\partial T}\right)}_{P}=C_{P}^{\mathrm{id}}(T)+C_{P}^{\mathrm{res}}(T,P)$$

where

60 $$C_{P}^{\mathrm{res}}(T,P)=\frac{1}{k_{B}T^{2}}\left[\left\langle U^{\mathrm{ext}}Ĥ\right\rangle -\left\langle U^{\mathrm{ext}}\right\rangle \left\langle Ĥ\right\rangle +P\left(\left\langle VĤ\right\rangle -\left\langle V\right\rangle \left\langle Ĥ\right\rangle \right)\right]-Nk_{B}$$

where *N* is the number of molecules in the system. Eq. ([Disp-formula eq60]) is derived by using the enthalpy, *H*,^645^

61 $$\begin{array}{l} H=H^{\mathrm{id}}+H^{\mathrm{res}}\Rightarrow \\ H=U^{\mathrm{int}}+K+Nk_{B}T+U^{\mathrm{ext}}+PV-Nk_{B}T\Rightarrow \\ H=Ĥ+K \end{array}$$

where *H* ^id^ and *H* ^res^ are the ideal and residual parts of *H*, and *K* is the kinetic energy. *C*_*P*_^id^ can be obtained from thermodynamic reference tables^643,646^ or quantum mechanical calculations.^53,647^

Thermal expansivity (*α_P_*) and isothermal compressibility (*β_T_*) from ensemble fluctuations follow from

62 $$\alpha_{P}=\frac{1}{\left\langle V\right\rangle }{\left(\frac{\partial \left\langle V\right\rangle }{\partial T}\right)}_{P}=\frac{1}{k_{B}T^{2}\left\langle V\right\rangle }\left(\left\langle VĤ\right\rangle -\left\langle V\right\rangle \left\langle Ĥ\right\rangle \right)$$

63 $$\beta_{T}=-\frac{1}{\left\langle V\right\rangle }{\left(\frac{\partial V}{\partial P}\right)}_{T}=-\frac{1}{k_{B}T\left\langle V\right\rangle }\left(\left\langle V^{2}\right\rangle -{\left\langle V\right\rangle }^{2}\right)$$

Having obtained *α_P_* and *β_T_*, one can also calculate the isochoric heat capacity from

64 $$C_{V}=C_{P}-\frac{T\langle V\rangle {\alpha_{P}}^{2}}{\beta_{T}}$$

The Joule–Thomson coefficient (*μ*_JT_) and speed of sound (*c*_sound_) follow from

65 $$\mu_{\mathrm{JT}}={\left(\frac{\partial T}{\partial P}\right)}_{H}=-{\left(\frac{\partial H}{\partial P}\right)}_{T}\frac{1}{C_{P}}=\frac{1}{c_{P}}\left[T{\left(\frac{\partial \upsilon }{\partial T}\right)}_{P}-\upsilon \right]=\frac{\upsilon }{c_{P}}\left[T\alpha_{P}-1\right]$$

and

66 $$c_{\mathrm{sound}}=\sqrt{\frac{c_{p}}{c_{V}\times \beta_{P}\times \rho }}$$

where *c_P_*, *c_V_* and *υ* are the specific heat capacities and the specific volume, respectively. In principle, the same fluctuation-based formulas and methods used to compute thermodynamic properties in MC simulations also apply to MD simulations performed in the same ensemble (e.g., see ref 308). Another example of using ensemble fluctuations in MD simulations is the study by Rahbari et al.^94^ where partial molar volumes and partial molar enthalpies of water in Nafion membranes were computed at different water uptakes.

It is noteworthy that for multi-component systems, the analytical expressions for thermodynamic derivatives based on fluctuations become increasingly complex. In such cases, using alternative methods such as the multiple linear regression are more practical and conceptually straightforward as shown by Rahbari et al.^648^.

#### 3.3.5 Hydrogen Diffusivity in Metals via Kinetic Monte Carlo

KMC provides a framework for simulating H diffusion across experimentally relevant time scales (nanoseconds to seconds) while retaining atomistic resolution of individual hopping events.^649,650^ The method is transferable across crystal structures. Here we illustrate with bcc and fcc lattices (such as Fe and Ni), though only the site topology and hop catalog require modification for other lattices. The simulation protocol begins by constructing a periodic supercell containing all symmetry-inequivalent interstitial sites (tetrahedral, octahedral) and defect traps (vacancies, dislocations, GBs). Each site *i* is assigned a solution energy *E*_*i*_^sol^ from DFT or validated potentials. For each allowed transition *i* → *j*, the CI-NEB calculations^651^ are used to determine the saddle-point energy *E*_*i*→*j*_^†^, while harmonic transition-state theory yields the prefactor *ν_i_*_→*j*_ (or a representative *ν* ≈ 10^13^ s^–1^ when phonon calculations are unavailable).^652^ Symmetry operations reduce equivalent hops to a minimal set stored for reuse. The hop rate follows from

67 $$k_{i\to j}=\nu_{i\to j}\exp\left[-\frac{E_{i\to j}^{†}-E_{i}}{k_{B}T}\right]$$

where *E_i_* =*E*_*i*_^sol^+*E*_*i*_^int^+*E*_*i*_^*σ*^ combines solution, interaction (H-H or H-defect), and optional elastic terms. The residence-time algorithm proceeds by: (1) selecting events proportionally to their rates using *u*_1_ *K* (*u*_1_ ∈ (0, 1], *K* = *∑_m_k_m_*), (2) advancing time by Δ*t* = – ln *u*_2_/*K* (*u*_2_ ∈ (0, 1]), (3) updating affected rates and heap structure, and (4) recording observables. The diffusivity is computed using the method described in Section 3.2.3, while trap statistics emerge from waiting-time distributions at defect sites.

A fundamental limitation of conventional KMC is its dependence on a predefined event catalog, in which all possible hops and their associated barriers must be known in advance. This requirement is tractable in pristine bcc or fcc lattices or for simple defects such as isolated vacancies, where diffusion pathways can be exhaustively enumerated. However, it becomes prohibitive in materials exhibiting a rich variety of local environments, such as H at complex GBs or dislocation cores, and is further exacerbated in chemically disordered systems like high-entropy alloys (see Section 4.11.6), where the combinatorial explosion of distinct atomic configurations yields effectively infinite diffusion paths and barrier values. Two strategies promise to remove this bottleneck. (i) On-the-fly KMC (for example using the kinetic Activation-Relaxation Technique) triggers a local saddle search whenever the system enters a new atomic environment, stores the resulting barrier under a topological fingerprint, and reuses it on subsequent visits.^653^ (ii) Machine-learning-assisted KMC trains a surrogate model (neural network) on a modest set of barriers from DFT or highly accurate interatomic potentials, predicts activation energies from local descriptors during the run.^468,654^ These adaptive workflows dispense with a static catalogue and make KMC practical for H diffusion in chemically and structurally heterogeneous micro-structures.

GCMC (see Section 3.3.2), on the other hand, can be an efficient pre-equilibration method for multi-scale diffusion studies. GCMC ”teleports” H atoms directly into deep traps such as vacancies, dislocation cores and GB sites, thereby establishing the equilibrium occupancy that ordinary diffusion would approach only after long KMC or MD times.^552^ To generate KMC-ready H distributions one first fixes the external chemical potential *μ*_H_ (or the equivalent gas pressure) and builds a simulation cell, starting with a defect-free lattice to calibrate bulk solubility and later expanding it to include vacancies, dislocations, GBS or precipitates. A cavity-biased grand-canonical Monte Carlo (CB-GCMC) pre-equilibration then accelerates sampling: the lattice is scanned once to catalogue genuine interstitial cavities, after which each MC sweep attempts three move types, namely (i) small displacements of existing H atoms, accepted according to the Metropolis criterion; (ii) insertions of H atoms restricted to the pre-identified cavities; and (iii) deletions of cavity-hosted H atoms, with both insertion and deletion accepted or rejected by the grand-canonical rule. The thermodynamically consistent, trap-filled configurations produced by CB-GCMC can be used for subsequent MD simulations that target H-affected deformation such as crack propagation^553^ and dislocation motion,^550^ allowing those simulations to focus on the relevant kinetics rather than spending computational time on equilibration.

## 4 Review of Thermophysical Properties of Hydrogen Systems Computed with Molecular Simulation

As discussed in Section 2, reliable thermophysical property predictions are essential for the design and optimization of all major H_2_ processes, i.e., water electrolysis, compression, storage, transportation, liquefaction, and underground sequestration. The relevance of data derived from molecular simulations lies both in the ability to extract physical insight from molecular structures and interactions, and in the reduction of required experimental measurements under hazardous or challenging conditions, e.g., high pressures or toxic environments. To this extent, the use of molecular simulation in the process design of H_2_ systems can greatly strengthen engineering workflows by providing reliable data on-demand. Here, we compile an extensive review of the available literature on classical molecular simulation of both pure H_2_ and of H_2_ systems involving other gases, liquids, and solids. Our discussion spans VLE of aqueous and non-aqueous H_2_ solutions, electrolyte systems, thermophysical properties, transport coefficients, and interfacial phenomena. Due to the extended body of literature reporting properties of H_2_ combined with porous and 2D materials (studied via both classical and quantum simulation methods), we limit the respective part of our review to mostly materials useful in UHS, hydrates, polymeric/composites, and metals. Nevertheless, a short overview of H_2_/membrane/nanoporous systems is provided, espcecially in light of the new advancements in the field exploiting ML and AI approaches.

### 4.1 Thermodynamic and Transport Properties of Aqueous Hydrogen Solutions

#### 4.1.1 Vapor–Liquid Equilibrium, Fugacity, and Henry Coefficients of Aqueous Hydrogen Solutions

The knowledge of VLE of aqueous H_2_ solutions is essential for the design and optimization of electrochemical systems and storage processes. In water electrolysis, the gas stream exiting the electrolyzer or separator tank typically consists of H_2_ saturated with water vapor. Accurate determination of the partial pressure of water in H_2_ is therefore critical for the design of downstream units such as knockout drums or dryers. Conversely, the solubility of H_2_ in water or aqueous electrolyte solutions is necessary for modeling concentration gradients within the electrolyte phase, for instance when calculating gas crossover. An overview of representative molecular simulation studies reporting phase equilibria data of H_2_ systems is provided in Table 9. The table includes both aqueous and non-aqueous systems (e.g., H_2_-hydrocarbon systems) to provide the reader a view of the available literature.

**Table 9:** Representative List of Phase Equilibrium Calculations of H_2_-Containing Systems Using Molecular Simulations, Along with Relevant Details on Systems, Force Fields, Conditions, and Methods^a^

		System Details		Thermodynamic Conditions		Simulation Method
Year	Study	System	Force Fields	Pressure	Temperature	
1993	Buch and Devlin^661^	H_2_O(s), H_2_(g)	Buch^661^		12 K	Diffusion MC, Path Integral MC
1994	Buch^312^	(para-D_2_)_3_(ortho-D_2_)_10_ and (para-D_2_)_13_ clusters	Buch^312^		1-3 K	Path Integral MC
1998	Rzepka et al.^322^	H_2_(g), Carbon Slitpores, Carbon Nanotubes(s)	Rzepka (H_2_)	10-30 MPa	50-600 K	GCMC
1999	Darkrim et al.^327^	N_2_(g), H_2_(g), Graphite(s)	Darkrim^327^	0-600 MPa (N_2_: 0-500 MPa, H_2_: 0-60 MPa)	77 and 293 K	GCMC
2001	Cracknell^311^	H_2_(g), Graphitic Nanofibres (GNFs)(s)	Buch,^312^ Cracknell^311^	1-112 bar	298 K	GCMC
2005	Urukova et al.^662^	CO_2_(g), CO(g), H_2_(g), [bmim][PF_6_](l)	Cracknell^311^	Up to 9 MPa	293 - 393 K	GE
2007	Ferrando et al.^323^	H_2_(g), n-Alkanes(g), Iso-Alkanes(g), Alkenes(g), Cycloalkanes(g), Aromatics(g), Polyaromatics(g)	Hirschfelder,^321^ Corresponding State, Darkrim,^327^ Cracknell	20.1-1200 bar	160-664.1 K	GE
2018	Köster et al.^48^	H_2_, N_2_, H_2_O, Ar, O_2_	Vrabec, Marx^310^	Up to 304.5 MPa	83.15-625 K	NPT, *μ*-VT
2019	Rahbari et al.^110^	H_2_(g), H_2_O(l)	Hirschfelder,^321^ Buch,^312^ Vrabec,^48^ Cracknell,^311^ Marx^310^	10-1000 bar	283-423 K	CFCMC
2020	Salehi et al.^663^	CO_2_(g), H_2_S(g), CH_4_(g), CO(g), H_2_(g), N_2_(g), ChClU(l), ChClEg(l)	H_2_ (Cracknell^311^), GAFF (ChClEg), OPLS (ChCIEg)	1 bar	328 K	CFCMC
2021	Rahbari et al.^47^	H_2_(g), H_2_O(l), Ice Ih(s)	TIP3P, Modified TIP4P (TIP4P*μ*); Marx,^310^ Vrabec,^48^ Cracknell,^311^ Buch,^312^ Hirschfelder (H_2_)	50-1000 bar (VLE: 100-1000 bar)	264.21-423.15 K	CFCMC
2021	Liu et al.^664^	CO_2_(g), SO_2_(g), N_2_(g), CH_4_(g), H_2_(g), Ionic Liquid(l)	Cracknell,^311^ OPLS-AA (ILs)	1 bar	300 K	NPT(MD)-GCMC
2022	Caviedes et al.^324^	H_2_(g), Carbon nano-pores(s)	Rzepka (H_2_(g)), Steele,^665^ Tjatjopoulos^666^ and Siderius-Gelb^667^ potentials for pore interactions	0.1-35 MPa	298.15 K	GCMC

a The data also contain non-aqueous systems for completeness.

Rahbari et al.^110^ computed densities and fugacities of pure H_2_, the VLE of H_2_-water systems covering temperatures of 310, 323, 366, and 423 K, and pressures ranging from 10 to 1000 bar, and VLE at low temperatures along the melting line of ice I_h_, corresponding to a temperature range of 264.21-272.4 K.^47^ This low-temperature regime is particularly relevant for evaluating the thermodynamic behavior of H_2_-water mixtures in refueling stations utilizing electrochemical compression. Several rigid, non-polarizable force fields were used in the CFCGE and CFC*NPT* ensembles (see Section 3.3.1.4). In the study by Köster et al.,^48^ the Grand Equilibrium method (see methodology in Section 3.3.1.6) was used to calculate the VLE of binary H_2_-water at 423 and 477.59 K for pressures in the range of 57 to 125 bar, as well as ternary mixtures of N_2_-Ar-H_2_ (which are discussed in Section 4.2). The solubilities of H_2_-water systems at high pressures reported in refs 47, 48, and 110 are compiled in Fig. 18.

**Figure 18:**
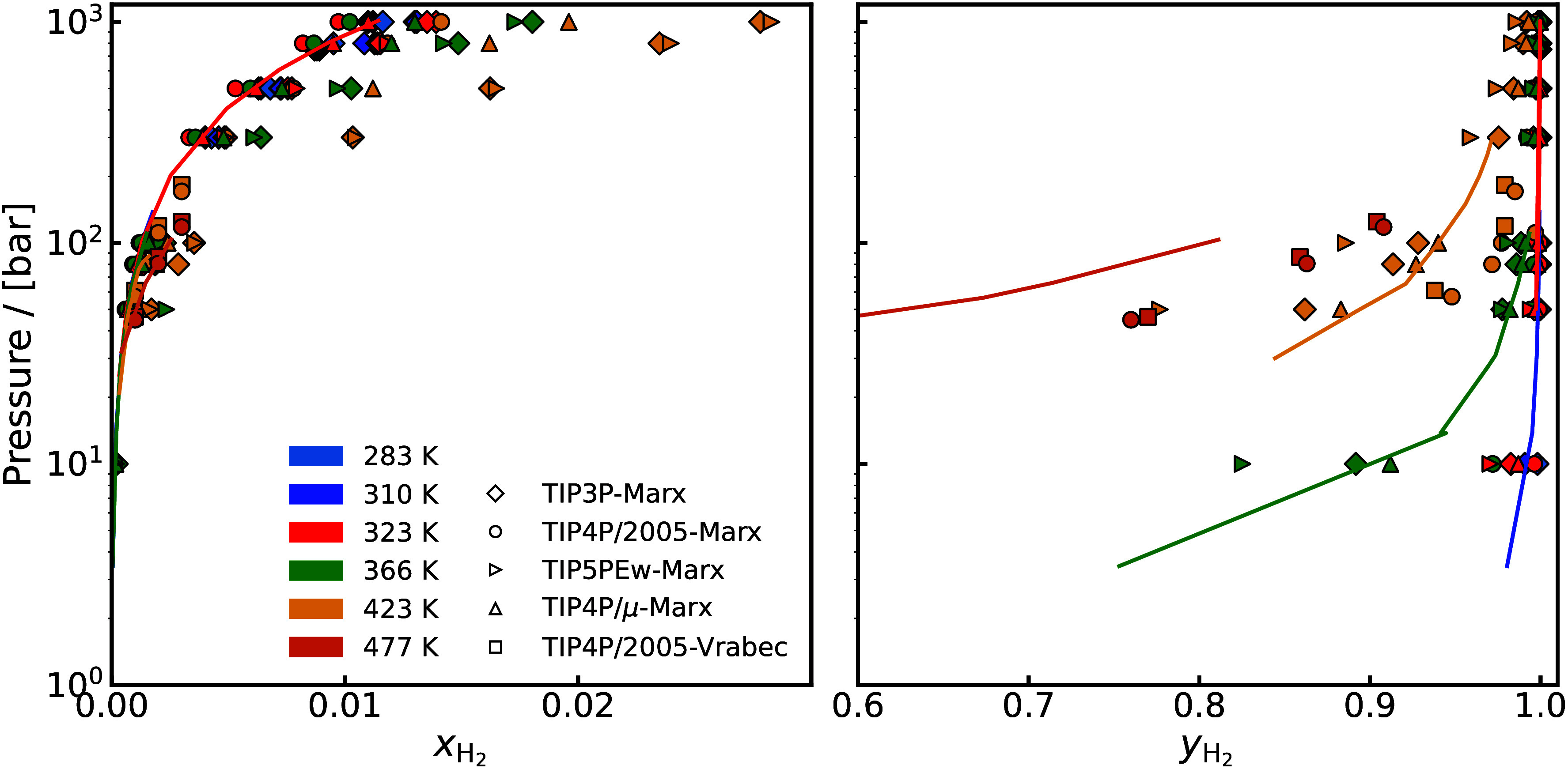
Isothermal phase diagram of the water-hydrogen system computed from molecular simulations, as reported in refs 47, 48, and 110. The solid lines represent experimental data. Simulation parameters and force field details are provided in refs 47, 48, and 110.

It is evident that experimental solubility data at elevated pressures remain limited. Available high-pressure solubility and VLE data have been compiled and reported in ref 110. For the gas phase, solubility measurements used for validation are in the following pressure ranges: 50-300 bar at 423 K, 3.45-110 bar at 366 K, 50-1013 bar at 323 K (the widest pressure range), and 3.44-138 bar at 310 K. For the liquid phase, experimental solubility data span 20-87 bar at 423 K, 3-110 bar at 366 K, up to 1013 bar at 323 K, and 3.45-138 bar at 310 K. These data, as visualized in Fig. 18, serve as a benchmark for evaluating the performance of various force field combinations in reproducing VLE behavior across different thermodynamic conditions. The raw solubility data from molecular simulations, used in Fig. 18, are provided in the Supporting Information of refs 47, 48, and 110 for several H_2_ models, including Hirschfelder,^321^ Vrabec,^48^ Buch,^312^ the two-site model of Cracknell,^311^ and the multi-site model of Marx.^310^ Fig. 18 shows that the TIP3P and TIP4P/*μ* (see Section 4.1.2.1) water models, when combined with the Marx force field for H_2_, yield the best agreement with experimental gas-phase solubility data. For the liquid phase, solubilities are most accurately captured using the TIP4P/*μ* combined with the Marx force field;^47^ however, this comes at the cost of reduced accuracy in predicting liquid water densities compared to the TIP4P/2005 model. These findings underscore that while specific force field combinations can be tuned to yield accurate phase composition predictions, this often involves trade-offs with other thermophysical properties.

In ref 47, the TIP3P and TIP4P/2005 force fields were used to compute the VLE of the H_2_-water system along the ice melting line and at elevated pressures. The results are presented in Fig. 19. TIP3P was selected in that study due to its superior performance in reproducing gas-phase compositions, as previously demonstrated by Rahbari et al.^110^ Given the low solubility of water in H_2_ at low temperatures, the mole fractions of water are plotted in Fig. 19 (right panel), rather than showing the H_2_ mole fraction, which approaches unity under these conditions. This representation is particularly relevant for applications involving deep-freezing of H_2_, such as at refueling stations, where accurate knowledge or prediction of water content at low temperatures is essential. No experimental data were found in published literature for the H_2_-water system at such high pressures, highlighting the value of molecular simulation as a powerful predictive tool to estimate VLE behavior where experimental data are lacking. For this, appropriately validated force fields is a prerequisite.

**Figure 19:**
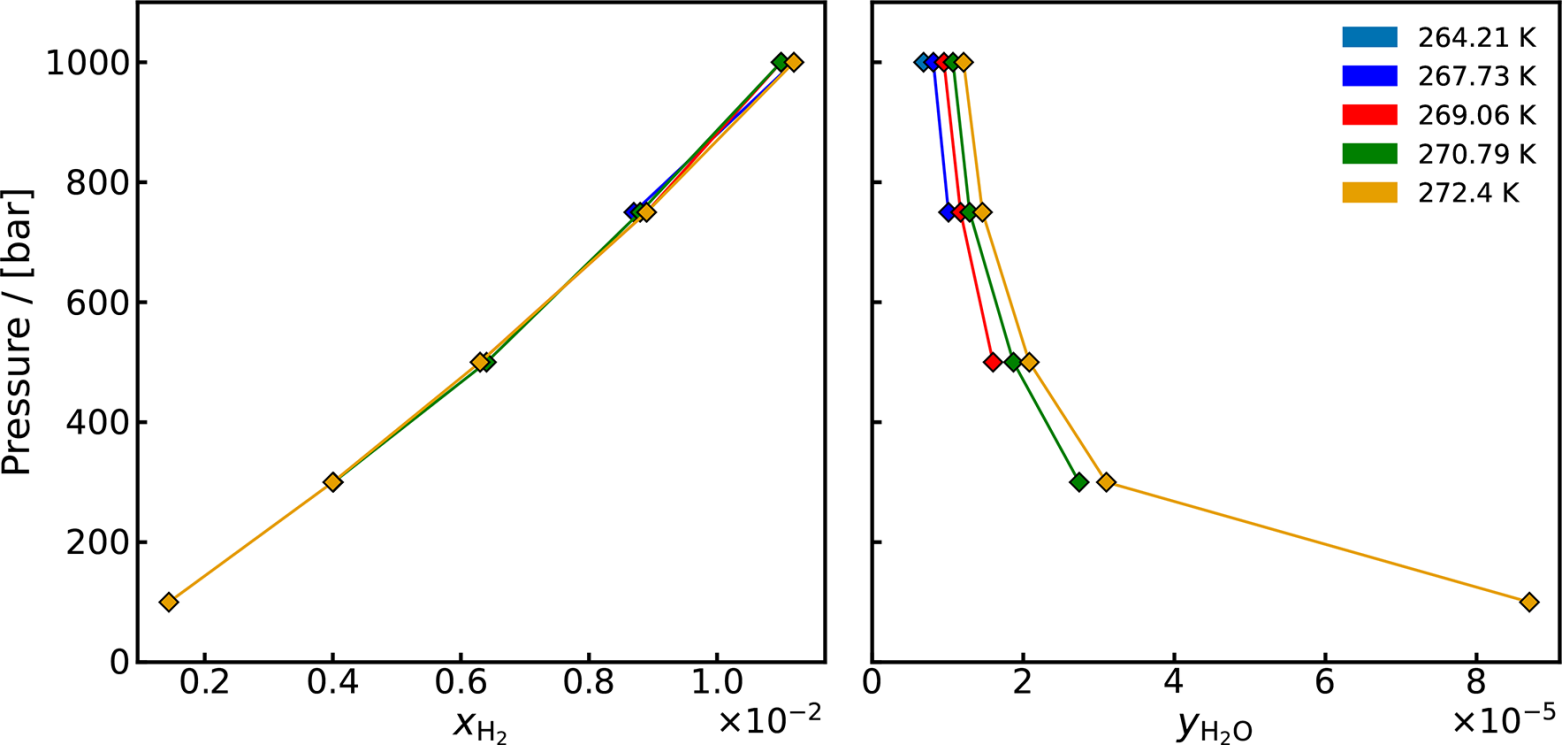
VLE of water hydrogen along the melting line of ice and higher pressures than the corresponding equilibrium pressure. The force field combination shown is TIP3P-Marx. The lines connecting the markers are drawn to guide the eye.

A notable advantage of the CFCMC method for VLE calculations is that in addition to determining phase coexistence, it enables direct sampling of the excess chemical potentials of each component in both phases through random walks in *λ* space. This facilitates the calculation of fugacity coefficients and Henry’s law constants within the same simulation framework. The fugacity coefficients of pure H_2_-calculated using the CFC*NPT* ensemble-and fugacity coefficients of H_2_ in the gas phase from VLE simulations using the CFCGE or CFC*NPT* ensembles are presented in Fig. 20. As discussed earlier, fugacity coefficients and Henry’s law constants are obtained inherently in these methods by introducing a fractional molecule and performing random walks in *λ* space.

**Figure 20:**
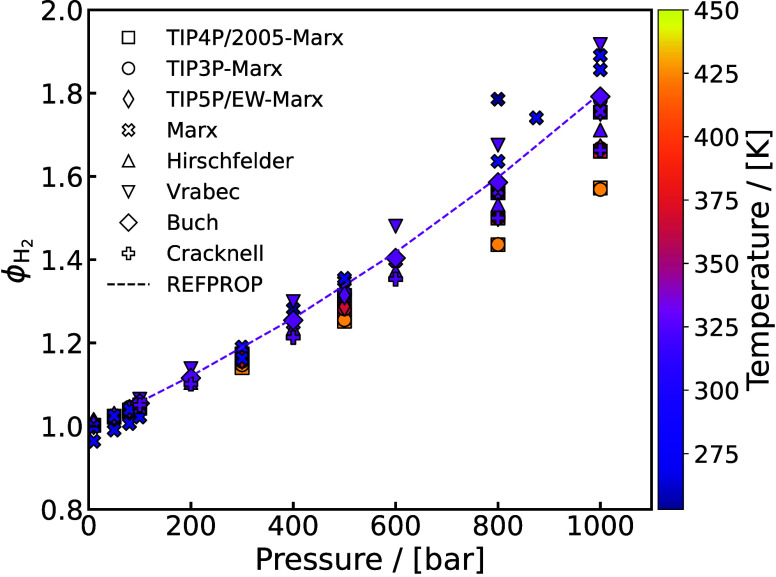
Fugacity coefficients of hydrogen computed for different force fields as a function of pressure. The data are obtained from legacy raw simulation data in refs 47 and 110 which have not been previously published, except for pure hydrogen at 323 K. As a reference, the fugacity coefficient of hydrogen at 323 K from REFPROP^352^ is shown.

H_2_ solubilities in the liquid phase obtained from the excess chemical potential, can be reported in form of Henry coefficient. The Henry coefficient (in units of bar) in ref 48 was computed using the following thermodynamic relation:

68 $$H=\rho k_{B}T\exp\left(\mu^{\inf}/(k_{B}T)\right)$$

where *μ* ^inf^ is the excess chemical potential at infinite dilution and *ρ* is the saturated liquid density of the solvent. In the MD simulation study by Zhang et al.^655^, the Henry constant is reported in units of bar^–1^ and is calculated using

69 $$H_{\mathrm{MD}}=\frac{\exp\left(-\mu^{\inf}/(k_{B}T)\right)}{\rho k_{B}T}.$$

Henry’s law coefficients for H_2_ in water reported by Köster et al.^48^ were obtained over a broad temperature range of 280-625 K. These results exhibit the characteristic non-monotonic temperature dependence of H_2_ solubility, with a clear maximum in the Henry’s constant between 330 and 370 K, indicative of the low solubility of H_2_ in water at intermediate temperatures. Zhang et al.^655^ also calcualted the Henry coefficient of H_2_ for different force fields of water and H_2_ using MD simulations combined with the WTPI method (see Section 3.3.1.3).

Henry coefficients reported by Köster et al. and Zhang et al. are presented in Fig. 21. To ensure consistent units in the figure, the reported Henry coefficients by Zhang et al.^655^ were converted to units of GPa. For completeness, the original correlation by the authors, valid at 273-433 K and 1 bar, is provided here as well:

70 $$H\left[\frac{x_{H_{2}}}{\mathrm{bar}}\right]=2.0369\times 10^{-4}-1.4628\times 10^{-6}T+3.6181\times 10^{-9}T^{2}-2.8405\times 10^{-12}T^{3}$$

**Figure 21:**
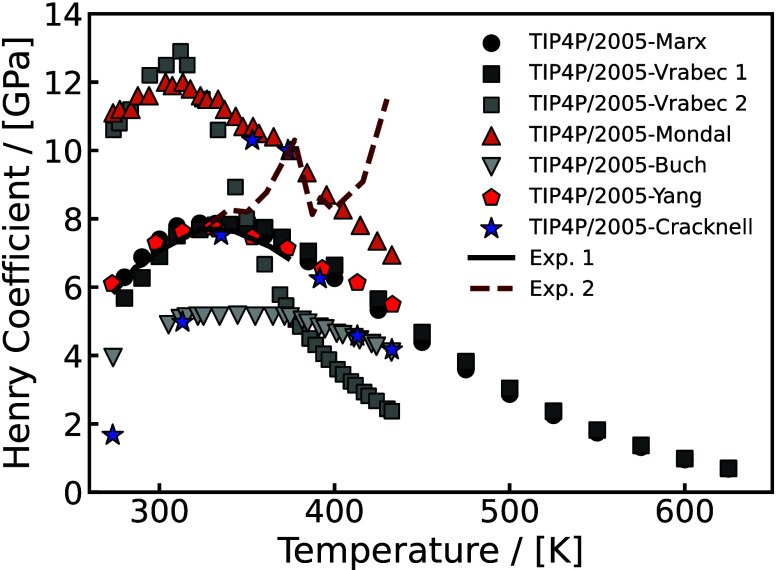
Henry coefficient of hydrogen in water as a function of temperature and pressure were compiled from refs 48 and 655. In ref 48, the Henry coefficients were calculated over a temperature range of 280-625 K. Zhang et al.^655^ employed the TIP4P/2005 water model in combination with various hydrogen force fields, including those by Buch, Mondal, Yang, Cracknell, and Vrabec. To differentiate between the datasets corresponding to the TIP4P/2005-Vrabec force field combination, the results from Köster et al. are referred to as the first set, while those reported by Zhang et al. are designated as the second set. Exp. 1 are experimental data from refs 655–657 and Exp. 2 from refs 655 and 658.

Experimental data compiled from refs 656 and 657 as cited by Zhang et al., are labeled as Exp. 1 in Fig. 21, while additional Henry coefficients reported by Zhang et al.^655^, based on data from Alvarez,^658^ are labeled as Exp. 2. The Exp. 1 dataset demonstrates internal consistency and excellent agreement with molecular simulation results. In contrast, the data from Alvarez significantly deviate from the other two sources, and are thus, distinguished as a separate experimental set, i.e., Exp. 2. Zhang et al. reported that the combination of TIP4P/2005 for water and the hydrogen model by Yang^325^ yields excellent agreement with the Exp. 1 dataset. However, their reported Henry coefficients based on the TIP4P/2005-Vrabec force field combination show inconsistencies when compared to those by Köster et al., whose results align well with the experimental data. The H_2_ force fields developed by Buch and Mondal reproduce the temperature dependence observed in the experimental data but systematically overpredict solubility across the entire temperature range. Nevertheless, both models are able to quantitatively capture the peak in the Henry coefficient between 300 and 350 K. Thermodynamic and structural properties of H_2_ gas in bulk water computed via MC simulations have also been reported by Sabo et al.^659,660^

#### 4.1.2 Vapor–Liquid Equilibrium of Aqueous Hydrogen Electrolyte Solutions

Obtaining accurate thermodynamic properties of aqueous electrolyte solutions is considered one of the most challenging modeling problems in applied thermodynamics due to the complex interactions between the dissolved ions and the solvent.^668–671^ Nevertheless, this is highly relevant for developing H_2_ technologies, which often involve electrolytes, e.g., alkaline solutions in electrolysis cells. Typical salts encountered in H_2_ applications are NaCl, NaOH, and KOH. For example, the solubility of H_2_ in aqueous NaCl solutions is relevant for underground storage of H_2_, and therefore, the prediction of accurate data at high temperatures (up to 500 K) and pressures (up to 1000 bar) is central.^630,672^

The reduction in the solubility of non-polar gasses in water in the presence of salts (ofter referred to as the salting-out effect) is a well-established phenomenon.^656,673,674^ The salting-out effect of H_2_ in aqueous solutions has also been studied using molecular simulations.^112,113,630,672,675^ van Rooijen et al.,^630^ Zhang et al.,^676^ Kerkache et al.,^672^ and Lopez-Lazaro et al.^675^ computed the solubility of H_2_ in concentrated NaCl solutions using MC simulations. Lopez-Lazaro et al.^675^ and Zhang et al.^676^ used the WTPI method to estimate the excess chemical potential of H_2_ in aqueous NaCl solutions (from which H_2_ solubilities can be computed as discussed in detail in Section 3.3.1). van Rooijen et al.^630^ and Kerkache et al.^672^ both performed CFCMC simulations to compute excess chemical potentials. As shown in Fig. 22(a), which is a compilation of solubility data from different studies, the H_2_ solubilities computed using the WTPI^675^ have error bars ca. 5 times larger than the solubilities computed using CFCMC calculations.^630,672^ This showcases that the gradual insertion using the CFCMC method allows for much better sampling of the excess chemical potentials compared to the WTPI (particularly in the liquid phase). The marginal differences between the solubilities computed in the different studies can be almost solely attributed to the choice of force fields, as discussed thoroughly later on.

**Figure 22:**
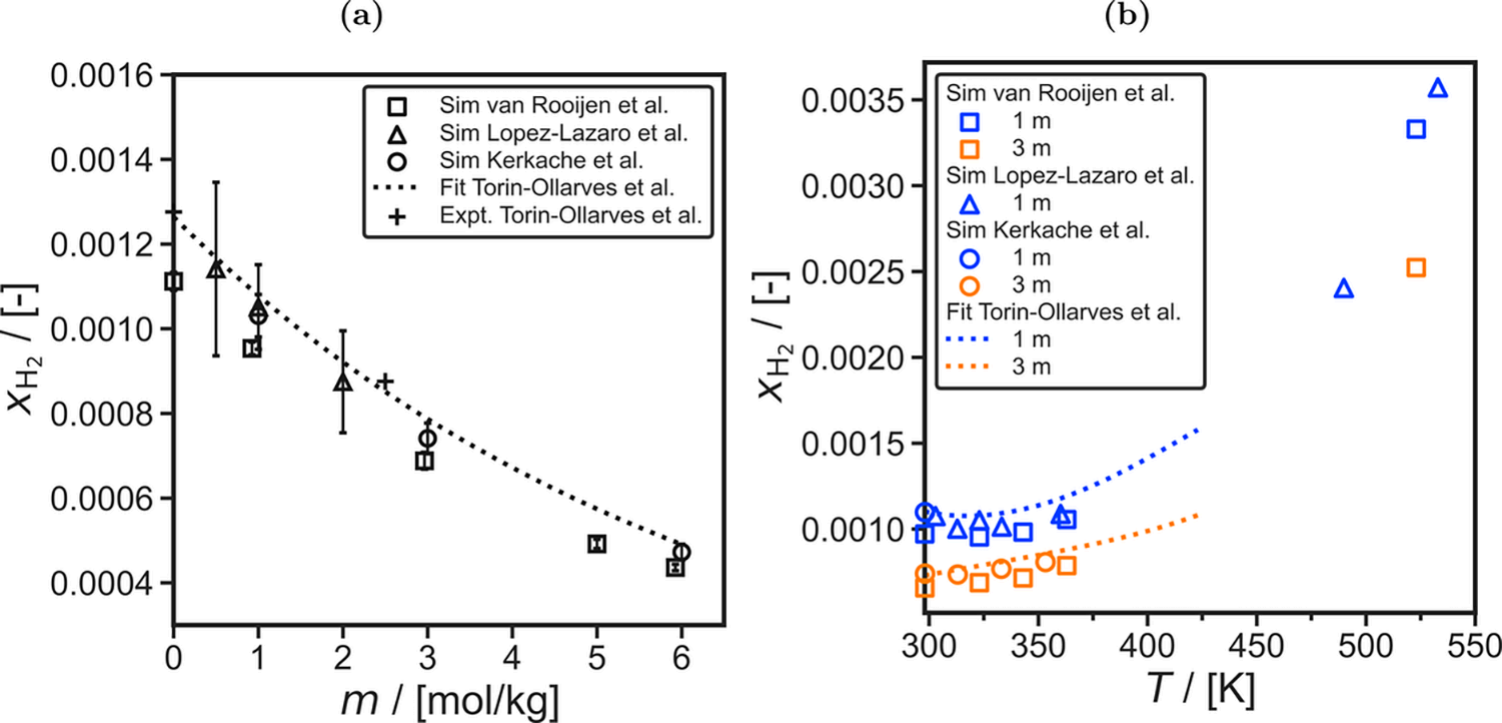
Solubilities of H_2_ (*x*_H_2__) in aqueous NaCl solutions at a H_2_ fugacity of 100 bar at (a) 323 K as functions of salt molality *m* (in units of mol salt / kg water), and (b) as functions of temperature *T*. The MC simulation data of van Rooijen et al.^630^ and Lopez-Lazaro et al.^675^ are shown. The MC data of Kerkache et al.^672^ for solubilities of Marx H_2_ in TIP4P/2005 water and Madrid-2019 ^347^ NaCl with adjusted interaction parameters (*k_ij_* for H_2_-H_2_O) is also plotted. The experimental fit of Torín-Ollarves and Trusler^674^ is shown for comparison.

It has been shown that scaling down the partial charges of ions can improve the accuracy in predicting thermodynamic and transport properties in systems involving electrolytes.^347,394,395^ This holds true also for aqueous H_2_ electrolyte solutions, where the choice of scaled or un-scaled force fields plays a dominant role. van Rooijen et al.^630^ combined Marx (for H_2_), TIP4P/2005 (for water), and the Madrid-2019 NaCl force fields. Kerkache et al.^672^ tested several different model combinations for H_2_O (i.e., TIP4P/2005, SPC/E, and TIP4P/EW), H_2_ (i.e., Marx, Alavi, and Yang and Zhong), and NaCl (i.e., Madrid-2019, KBF, and OPLS). Madrid-2019 NaCl has scaled charges of +0.85 / -0.85 [e], while KBF and OPLS have charges of +1 / -1 [e]. Kerkache et al.^672^ showed that NaCl force fields with un-scaled charges under-predict the solubilities of H_2_ (thereby over-predicting the salting out effect), especially at higher salt molalities (≥ 4 mol salt / kg water). Lopez-Lazaro et al.^675^ and Zhang et al.^676^ also showed that the combination of Darkrim H_2_ force field with TIP4P/2005 water, and OPLS, Joung-Cheatam, Smith, and Dang NaCl force fields (all having un-scaled charges) under-predict the solubility of H_2_ in aqueous NaCl solutions by ca. 25%. Despite this, behavior of H_2_ solubilities as a function of temperature is correctly predicted in refs 675 and 676. To accurately model the salting out of H_2_ in aqueous NaCl solutions, Lopez-Lazaro et al.^675^ adjusted the LB mixing rules for Na^+^-H_2_ and Cl^–^-H_2_. Madrid-2019 NaCl force field with scaled charges of +0.85 / -0.85 [e] was shown to perform accurately in predicting the salting out of H_2_ in aqueous NaCl solutions without any further fitting of mixing rules as shown in Fig. 22 and discussed in refs 630 and 672.

Habibi et al.^112^ computed the solubilities of H_2_ and O_2_ in aqueous KOH and NaOH solutions using the scaled charge force fields Madrid-Transport (K^+^ and Na^+^) and the DFF/OH^–^ (developed in the same study). The authors performed CFCMC simulations at a temperature and pressure range of 298-353 K and 1-100 bar, respectively. The authors showed that thew newly developed DFF/OH^–^ model accurately predicts densities and viscosities (5 to 20% deviations) of the aqueous NaOH and KOH solutions up to molalities of 8 mol/kg. The expected salting-out effect with increasing electrolyte concentration was also captured. Engineering equations were fitted using the simulation data for both solubilities and diffusivities of the gases in the aqueous solutions. It is important to note that the solubilities of H_2_ in water are ca. 1 order of magnitude smaller at a concentration of 8 mol KOH / L solution compared to the solubilities of H_2_ in pure water. Therefore, the choice of the salt force field is crucial when computing the solubility of H_2_ in the aqueous phase. This is also the main motivation for heavily discussing the performance of force fields in this section.

Hulikal Chakrapani et al.^677^ performed molecular simulations to compute the solubilities of H_2_-CO_2_ mixtures in aqueous NaCl solutions over a wide range of pressures (5-50 MPa), temperatures (323.15-423.15 K), and salinities (0-2 molal NaCl). TIP4P/*μ*,^110^ all-atom TraPPE,^678^ and Marx^328^ force fields were used for water, CO_2_, and H_2_, respectively. Using the CFCGE (see Section 3.3.1.1) method, solubilities of H_2_, CO_2_, and H_2_O were computed by equilibrating chemical potentials between the gas and liquid phases, with a fixed initial 1:1 H_2_:CO_2_ gas phase ratio. CO_2_ solubility in aqueous NaCl solutions increased with pressure and temperature, but decreased with salinity due to the salting-out effect (well captured by the Setschenow equation^679^). The decline in solubility followed a consistent slope of approximately −0.21, independently of pressure and temperature. At low pressures (<10 MPa), CO_2_ solubility rose linearly, transitioning to a non-linear behavior at higher pressures. Simulations matched experiments within 5-10% at 423.15 K. Larger deviations were observed at lower temperatures.

In the study of Hulikal Chakrapani et al.^677^ H_2_, solubility increased with temperature across the range 323.15–423.15 K, at all pressures and salt concentrations. However, at a fixed pressure and temperature, the solubility of H_2_ decreased with increasing salt concentration, exhibiting a Setschenow-type^679^ slope of −0.11 to −0.19, corresponding to ca. 15% decrease in solubility per mole NaCl. The simulation results showed good agreement (within 5–10%) with experimental data at 323.15 K, though higher temperatures and pressures led to slight overestimations. In CO_2_ – H_2_ – H_2_O – NaCl systems, CO_2_ solubility was shown to be lower than in CO_2_ – H_2_O – NaCl (see Fig. 23a), whereas H_2_ solubility increased (see Fig. 23b), suggesting a cooperative mechanism where H_2_ promotes its own solubility while suppressing that of CO_2_. Water content in the gas phase was computed to be between the values observed in the CO_2_ and H_2_ aqueous electrolyte systems (see Fig. 23c). At 423.15 K, water content decreased with pressure but showed little dependence on salt concentration. At 323.15 K, the water content remained nearly constant, while at 423.15 K it was found to be 20–40 times greater than the value at 323.15 K, when considered across all pressures.

**Figure 23:**
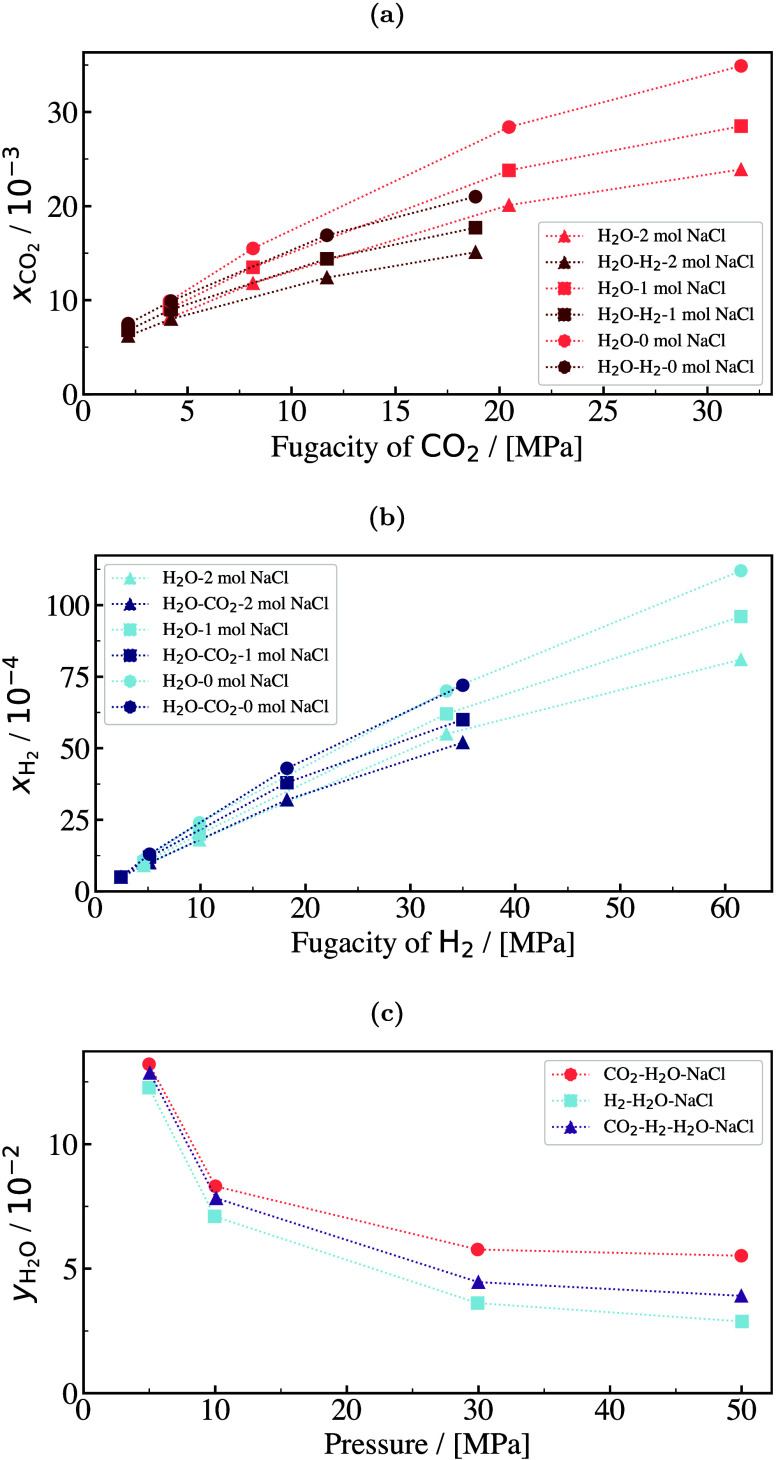
(a) The solubility of CO_2_ in different aqueous NaCl solutions as a function of CO_2_ fugacity. (b) The solubility of H_2_ in different aqueous NaCl solutions as a function of H_2_ fugacity. (c) The mole fraction of H_2_O in the gas phase as a function of pressure for different aqueous NaCl-H_2_ mixtures. The lines connecting the markers are drawn to guide the eye.

The solubilities of H_2_ in aqueous NaB­(OH)_4_ solutions were recently computed using CFCMC simulations at 298–363 K and 1 bar.^113^ This electrolyte is relevant for applications related to hydrolysis of NaBH_4_ solutions for H_2_ storage. The presence of 5 mol NaB­(OH)_4_ / kg water is shown to significantly reduce both H_2_ solubilities (by a factor of ca. 0.4) and the activities of water (by a factor 0.75). In a follow up work also focusing on NaBH_4_ as a H_2_ energy carrier, Postma et al.^680^ developed a new force field (i.e., the DFF/BH_4_^–^, extending the DFF family) performed MD simulations to compute densities and viscosities of NaBH_4_ (in the range 0 to 5 mol salt / kg water), NaB­(OH)_4_ (0 to 3 m), and 1 m NaOH aqueous solutions at 295 K. The predicted properties were within ca. 2% and 10% from the respective experimental data. The authors used the MD data to devise engineering correlations for densities, viscosities, and self-diffusivities for these systems which can be used as a fast model when data are required for e.g., NaBH_4_ hydrolysis reactor design.

##### 4.1.2.1 Force Field Performance and the Effective Charge Surface Approach

It is observed in the pervious section that obtaining accurate VLE data reequires careful selection of force field combinations for hydrogen and water. The results show that there is not a single combination of classical force fields that can predict all thermodynamic and transport properties accurately. An illustrative example is provided in Fig. 24, aiming to show the differences in performance as a result of different force field parameterization. Figure 24 shows the solubilities of H_2_ in liquid water and the solubilities of water in gaseous H_2_ computed using MC simulations at 323 K and pressures from 10 to 1000 bar.

**Figure 24:**
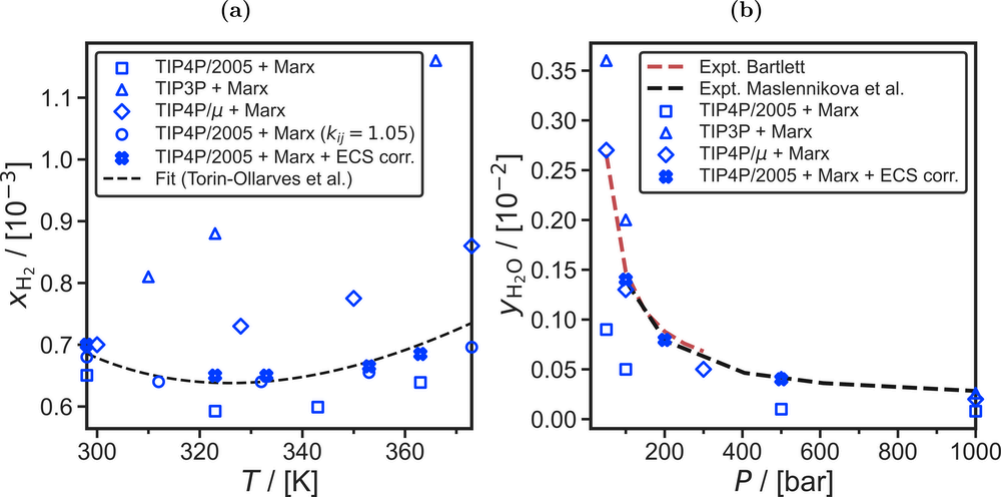
(a) Solubilities of H_2_ (in units of mole fraction *x*_H_2__) in liquid water at a H_2_ fugacity of 50 bar as functions of temperature. (b) Solubilities of H_2_O (in units of mole fraction *y*_H_2_O_) in gaseous H_2_ at 323 K as functions of total pressure. The MC data for *x*_H_2__ and *y*_H_2_O_ for the combination of Marx^310^ H_2_ force field and TIP4P/2005 and TIP3P water is obtained from Rahbari et al.^46^ The MC data for *x*_H_2__ and *y*_H_2_O_ of Marx^310^ H_2_ and modified TIP4P (herein referred to as TIP4P/*μ*) is obtained from refs 111 and 47, respectively. The MC data of Kerkache et al.^672^ for solubilities of Marx H_2_ in TIP4P/2005 with adjusted interaction parameters (*k_ij_* for H_2_-H_2_O) is also shown in (a). The MC data for Marx^310^ and TIP4P/2005 water force field including the ECS free energy correction as introduced by Habibi et al.^350^ is obtained from ref 111. The experimental fit of Torín-Ollarves and Trusler^674^ for *x*_H_2__ and the experimental data of Maslennikova et al.^681^ and Bartlett^682^ for *y*_H_2_O_ are shown for comparison.

In ref 110 it is shown that the TIP3P water force field^300^ combined with the Marx^310^ force field results in accurate predictions for the equilibrium water content in the gas phase but cannot accurately predict the solubilities of H_2_ in the liquid phase. The TIP4P/2005 water force field and the Marx^310^ force field result in accurate predictions for the solubility of H_2_ in water (and the variations with respect to temperature), but the water content in H_2_ is under-predicted with respect to experiments (e.g., by a factor of 3 at 323 K).^110^ The TIP3P water force field paramters are fitted on the vaporization energy of water, and can thereby correctly predict the experimental vapor pressure of water and the water content in the gas phase.^110,350^ The TIP4P/2005 ^349^ water force field parameters are trained on the liquid phase properties such as densities, temperature of maximum density, and transport properties (e.g., shear viscosities of water), and can thereby predict the solubilities of H_2_ in water (and their temperature dependence) accurately with respect to experiments.^110^

In ref 47, a modified version of TIP4P/2005 (i.e., using the same geometry) was employed by scaling the atomic charges by a factor of 0.955, resulting in a dipole moment of 2.2 D. Subsequently, the Lennard-Jones energy parameter *ϵ* for oxygen was adjusted to 79.86 K to better match the chemical potential of liquid water. The TIP4P/*μ* accurately models the excess chemical potential of water (i.e., with respect to the ideal gas reference state), and results in accurate modeling of the water content in H_2_. The solubilities of the Marx H_2_ force field^310^ in water at 298 K are also accurately modeled. However, the temperature dependence of H_2_ solubilities in water are not accurately captured (no minimum is observed in H_2_ solubilities at ca. 323 K similar to experiments^683^).

The reason why it is difficult to model the VLE of H_2_ and water systems using non-polarizable water force fields can be traced back to the paper of Berendsen et al.^346^ in 1987. Berendsen et al.^346^ discussed that non-polarizable water force fields cannot capture both the experimental vaporization energy of water (required for modeling the vapor pressures of water) and the effective interactions that result in accurate predictions of densities (required for modeling the solubility of H_2_ in water) and transport properties of water, due to “the missing term in effective pair potentials” (i.e., the title of the paper by Berendsen et al.^346^). Recently, Habibi et al.^350^ developed a new method in which the TIP4P/2005 water force field is used to model the effective interactions in the liquid phase and an additional effective charge surface (ECS) is used to compute the excess chemical potential of water. The excess chemcial potential of water obtained using the ECS is used to compute a free energy correction for the free energies of TIP4P/2005 at different temperatures. Applying a free energy correction to the partition function of the isolated water molecules allows for accurately computing the excess chemical potentials of water, without compromising the liquid phase properties. As shown in Figure 24 and ref 111 this energy correction can be used to accurately predict both the equilibrium water content in the gas phase and the solubilities of H_2_ in the liquid phase with respect to experiments. The simulations are performed at 298-423 K, 10-500 bar, 0-6 mol NaCl/kg water, and 0-8 mol KOH/kg water. As discussed in ref 350 it is important not to model water vapor using non-polarizable water force fields trained on the liquid phase (e.g., TIP4P/2005 and TIP3P), as the second virial coefficients of water vapor (and thereby the excess chemical potentials of water in the gas phase) are not accurately modeled with respect to experiments.^684^ It is advised to combine MC simulations with EoS, such that the MC simulations can be used to obtain the liquid phase chemical potentials and free energies and the EoS (such as the GERG-2008 ^685^) can be used to model H_2_O-H_2_ gaseous mixtures.

#### 4.1.3 Pressure–Volume–Temperature Computations

Yang et al.^686^ performed molecular simulations of aqueous H_2_/CO_2_/H_2_O systems to compute *pVT* data and densities at conditions close to the supercritical regime, for which no prior experimental data were available. The motivation for this molecular simulation study is the application of supercritical water gasification of coal for energy production, a process in which *pVT* properties are difficult to measure. The authors state that no experimental, theoretical, or simulation studies exist regarding the *pVT* properties of H_2_O-H_2_-CO_2_ mixtures in the near-critical and supercritical regions of water. They tested both the Cracknell and Buch force fields for hydrogen, but the results presented in the paper are based on the Cracknell force field. In their H_2_O-CO_2_ binary-mixture validation, the authors report that the absolute average error of the PR EoS and MD simulation is 15.09% and 0.66%, respectively. Thus, the adopted MD simulation model appears to have significantly better predictive accuracy than the PR EoS in near-critical and supercritical regions of water. However, for the H_2_O-H_2_ binary system, they note that no experimental research has investigated the *pVT* properties of H_2_O-H_2_ mixtures under those conditions. Therefore, their MD results are only compared against Peng-Robinson calculations, with no direct experimental benchmark for H_2_O-H_2_ in the near-critical and supercritical regimes. The pressure calculations obtained for binary H_2_O-H_2_ and ternary H_2_O-H_2_-CO_2_ mixtures are shown in Fig. 25. Temperatures vary from 673 to 973 K, and the mole fractions of hydrogen range from 0.05 to 0.20. The results obtained from MD simulations are potentially more accurate and can provide more insight than the PR EoS.

**Figure 25:**
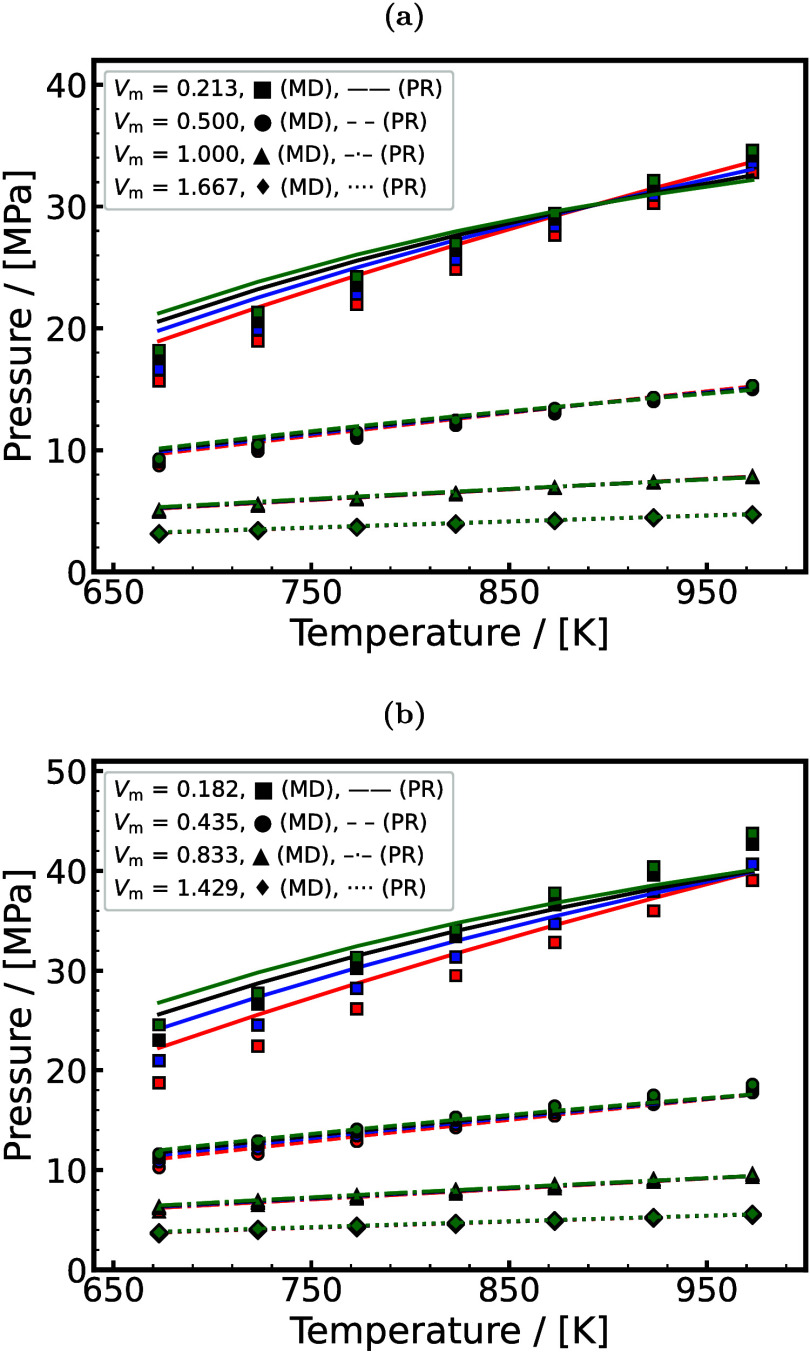
Pressure-temperature relation of (a) binary H_2_O-H_2_ mixtures and (b) ternary H_2_O-H_2_-CO_2_ mixtures at different hydrogen mole fractions and molar volumes. MD simulation results are compared with predictions from the Peng-Robinson EoS. The different colors of the lines and markers correspond to different mole fractions of hydrogen (red: *x*_H_2__=5%, blue: *x*_H_2__=10%, black: *x*_H_2__=15%, and green: *x*_H_2__=20%).

#### 4.1.4 Thermal Expansivity, Heat Capacity, Joule-Thomson Coefficient, and Speed of Sound of Aqueous Hydrogen Mixtures

As discussed in Section 3.3.4, isothermal compressibility, thermal expansivity, heat capacity, speed of sound, and the Joule-Thomson coefficient are thermodynamic properties which can be computed from ensemble fluctuations in molecular simulations.^571,642,643^ Alternatively, these properties can be calculated using the multiple linear regression method which have been shown to be mathematically equivalent.^648^

The study by Rahbari et al.^47^, mainly motivated from electrochemical compression systems, focused on the effect of trace amounts of water on the thermodynamic properties of compressed H_2_ using MD simulations. The authors computed thermal expansivity, heat capacity, Joule-Thomson coefficients, partial molar volumes and enthalpies and the VLE of pure H_2_ and aqueous H_2_ mixtures. The H_2_-water data were obtained from a prior study by the authors^110^ where equilibrium compositions were sampled at 366 - 423 K, and pressures up to 1000 bar. Importantly, the isothermal compressibility data were retrieved from the legacy simulation data in ref 47 and are presented in this review for the first time (Fig. 26 upper). TIP3P model was used for water, while a new force field, i.e. TIP4P/*μ* was developed to yield improved solubilities (for more details the reader is referred to the Supporting Information of ref 47 and Section 3.1.2). H_2_ was modelled with different force fields, with Marx showing the best overall performance. The molecular simulation results clearly indicated that at low pressures, the water content has significant influence on the thermodynamic properties of the H_2_/H_2_O mixtures (i.e., a positive Joule-Thomson coefficient for H_2_O – H_2_ mixtures and a negative value for pure H_2_ mixtures at 423 K and 50 bar).^47^ The effects become less pronounced as pressure increases, and become negligible above 700 bar. All computed properties (for different force field combinations) are shown in Fig. 26 along with available data from NIST (REFPROP92 ^352^) for pure H_2_. These results underline the importance of accounting for trace water content when modeling hydrogen systems for storage and energy applications.

**Figure 26:**
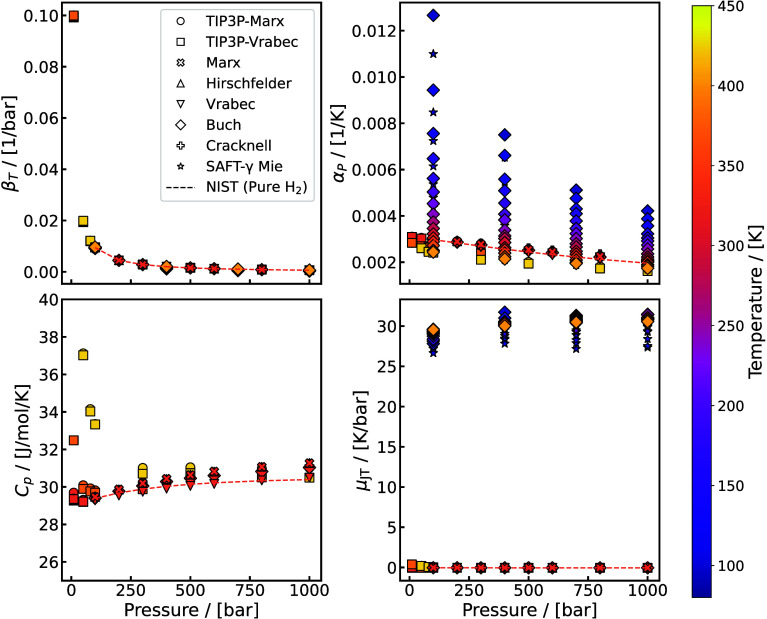
Comparison of different force fields in predicting the isothermal compressibility (top left), thermal expansivity (top right), isobaric heat capacity (bottom left), and Joule-Thomson coefficient (bottom right) of pure hydrogen in the gas phase over a pressure range of 10 to 1000 bar. Experimental data from NIST (REFPROP92 ^352^) are shown as lines. Raw simulation data are provided in the Supporting Information of ref 47. The error bars are smaller than the symbol size. For force field combinations with TIP3P, representing a gas phase saturated with water, the compositions at each temperature are directly taken from VLE simulations in ref 110 where the TIP3P force field was used for water and the Marx force field for hydrogen. The mole fraction of water in saturated hydrogen–water mixtures in the gas phase ranges from 0.004 to 0.138. Raw data from ref 308 were provided by the authors.

Bartolomeu and Franco^308^ developed a single-site force field for H_2_ using the SAFT-*γ* Mie potential, and validated it against existing LJ-type models (both 1-site and 2-site) for predicting thermophysical properties of supercritical H_2_. Similarly to the study by Rahbari et al.,^47^ the motivation of this study was high-pressure EHC applications. The authors reported both thermodynamic (i.e., densities, isochoric and isobaric heat capacities, thermal expansion coefficients, isothermal compressibilities, joule-Thomson coefficients, and speed of sound) and transport properties (i.e., diffusivities, viscosities, and thermal conductivities). The data from Bartolomeu and Franco^308^ (provided to us by the authors) for the thermodynamic properties are shown in Fig. 26 and Fig. 27. This study emphasized that quantum effects are non-negligible at low temperatures, particularly when the de Broglie thermal wavelength approaches the inter-molecular distance or when the simulation temperature is below the rotational temperature of hydrogen (86 K). The authors observed that while a classical treatment may be acceptable for temperatures above 200 K, below this threshold, classical models increasingly deviate from experimental reference data. Among the tested models, the SAFT-*γ* Mie (which is a force field developed in the same study^308^) yielded the lowest average deviations, attributed to its softer repulsive potential and EoS-consistent parameterization, despite the absence of explicit quantum corrections.

**Figure 27:**
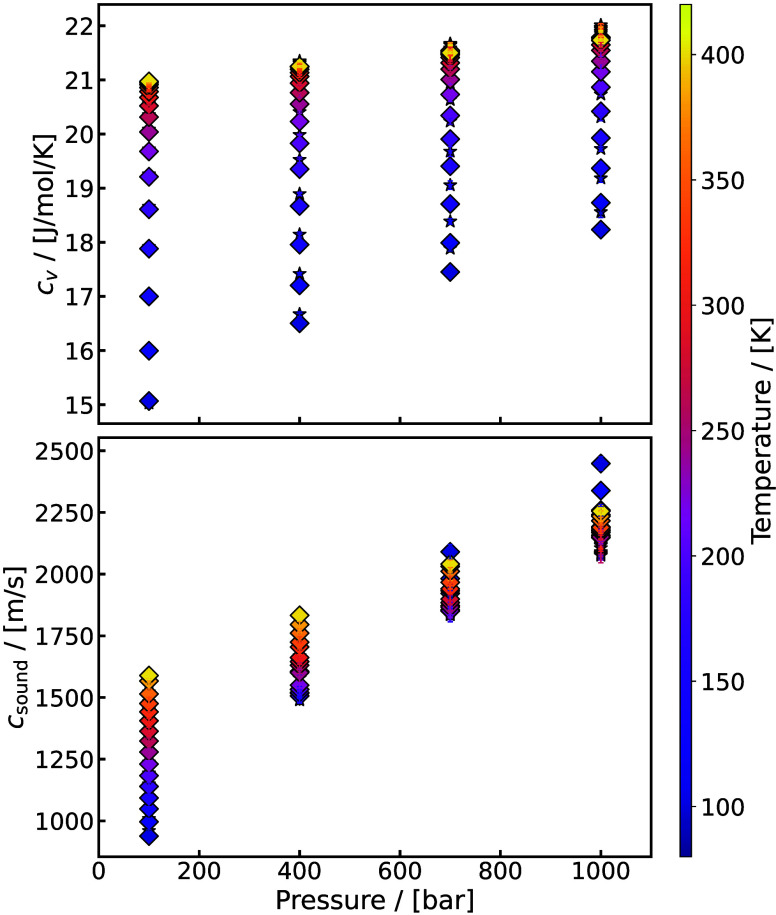
Isochoric heat capacity (top) and speed of sound (bottom) of supercritical hydrogen from ensemble fluctuations from MD simulations. Raw data from ref 308 was provided by the authors.

#### 4.1.5 Self-Diffusivity of Hydrogen in Aqueous Electrolyte Solutions

As discussed in detail in Section 2.1, reliable data of H_2_ diffusivities in the aqueous phase are essential for the design and optimization of H_2_ electrochemical systems such as water electrolyzers and fuel cells.^71^ The diffusion of H_2_ in the aqueous phase influences the bubble formation and growth rates, which in turn influence the ohmic resistance of the aqueous electrolyte and reduces the active electrode area.^71^ The self-diffusivity of H_2_ is also a relevant property in supercritical water gasification for conversion of biomass to fuel gasses.^687^ Obtaining self-diffusivities of H_2_ (and other gasses) at high pressures and temperatures (e.g., supercritical conditions) requires cumbersome and expensive experiments. Thus, molecular simulations have been widely used for obtaining these data over a large temperature and pressure range.^688,689^ The relevant methods are described in Section 3.2.

Fig. 28 shows the temperature and pressure conditions for which data (MD and experimental) of H_2_ self-diffusion coefficients in water are available.^687,688,690^ The experimental dataset of Wang et al.^690^ spans the most state points compared to the rest of the available experiments.^690^ Additional experimental data at low pressures (0.1 MPa) have been collected and reported in ref 688. To the best of our knowledge, no experimentally measured self-diffusivities of H_2_ in supercritical water are reported in open literature. Zhao and Jin^687^ computed the diffusivity of H_2_ in supercritical water while Tsimpanogiannis et al.^688^ performed MD simulations to compute the diffusivities of H_2_ and O_2_ in water up to pressures of 2000 bar and 975 K. Kallikragas et al.^691^ computed the diffusivity of H_2_, O_2_, OH radical, and H_2_O in liquid water at a temperature range of 298 - 973 K and water densities spanning 0.1 g cm^–3^ to 1 g cm^–3^. Since small gas molecules such as H_2_ are sparsely soluble in liquid water (i.e., mole fractions of ca. 10^–5^ at 298 K and a H_2_ pressure of 1 bar^112^), simulations of H_2_ are often performed at near infinite dilution.^687,688,691^ Therefore, to a great extent, the behavior of the solvent (e.g., density, viscosity) is the one dictating the diffusivity of the gas. This means that the choice of the water force field is crucial in these studies because the properties of the solvent need to be accurately modeled over a wide temperature and pressure range. Tsimpanogiannis et al.^688^ and Zhao and Jin^687^ used TIP4P/2005,^692^ while Kallikragas et al.^691^ used SPC/E. Both these water force fields perform well in modeling the density, viscosity, and self-diffusivity of liquid water.^345,348,692–694^ As discussed in Section 3.1.2, TIP4P/2005 and SPC/E are rigid, non-polarizable force fields, ca. 3–10 times more computationally efficient than polarizable force fields.^354,355,695^ The computational efficiency of non-polarizable force fields is a significant advantage compared to polarizable ones or quantum mechanical simulations (e.g., Born-Oppenheimer MD) for generating large datasets for transport properties of H_2_ in aqueous mixtures. Polarizable force fields have been used by Śmiechowski^314^ (i.e., AMOEBA combined the H_2_ force by Śmiechowski^314^) to compute the diffusion of H_2_ and H_2_O in liquid water. However, these computations were carried out only at 298 K, and were not the main focus of the work by Śmiechowski^314^ (i.e., calculation of Raman spectra for the hydrated H_2_).^314^

**Figure 28:**
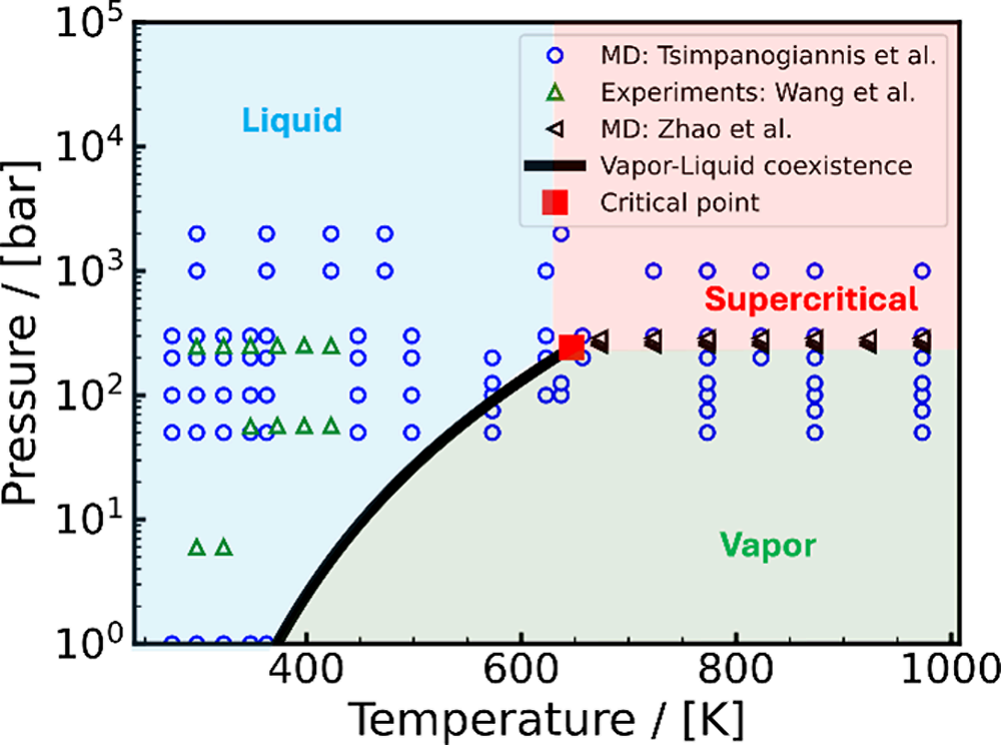
State points for which self-diffusivities of H_2_ in water are available in literature. Experimental data ranges are from Wang et al.^690^ which is the largest experimental dataset available. MD simulation data ranges are from the studies of Tsimpanogiannis et al.^688^ and Zhao and Jin.^687^

To model H_2_, Tsimpanogiannis et al.^688^, used six different force fields for (i.e., Buch,^312^ Marx,^310^ Vrabec,^48^ Cracknell,^311^ Silvera-Goldman,^335^ and Hirschfelder^321^ H_2_ force fields - see relevant discussion in Section 3.1.1). Depending on the H_2_ model, the self-diffusivities varied by ca. 20–30%. Though this is a notable variation, it is comparable to the error of experimentally measured H_2_ diffusivities found in literature.^348^ The authors concluded that the Buch–TIP4P/2005 force field combination, performs the best, and proceeded in producing a wide range of data for H_2_ and O_2_ self-diffusivities in H_2_O as shown in the Arrhenius-type plot of Fig. 29. Based on these data, Tsimpanogiannis et al.^688^ developed engineering correlations for the regions shown in Fig. 29 corresponding to vapor, liquid, and supercritical conditions. In a follow-up study, Moultos and Tsimpanogiannis^696^ revised the proposed correlation of the vapor region to increase the accuracy of predictions for the lower pressure and higher temperature regions.

**Figure 29:**
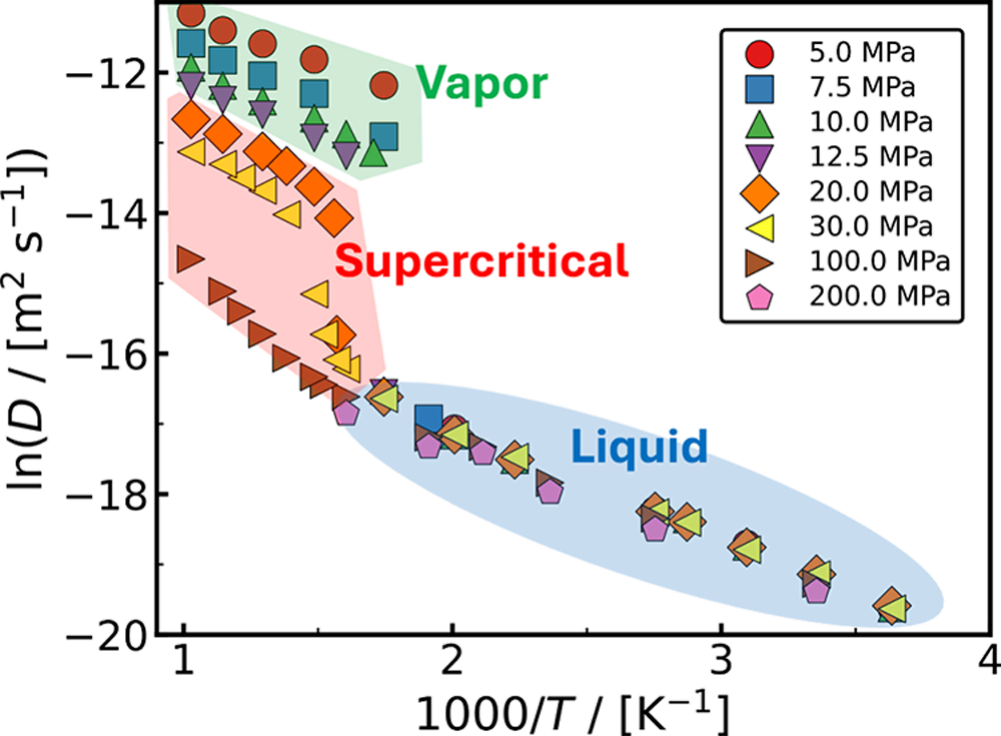
Arrhenius plot of the self-diffusion coefficients of H_2_ in H_2_O computed by Tsimpanogiannis et al.^688^ using the Buch^312^–TIP4P/2005 ^305^ force fields. The computed diffusion coefficients are corrected for system-size effects.^498–500^ The identified self-diffusivity patterns of the H_2_ in H_2_O, in the vapor, liquid, and supercritical regions are highlighted. Data were collected from ref 688.

Available data from experiments and MD simulations for the self-diffusivities of H_2_ in liquid H_2_O have been considered to examine the validity of the Stokes-Einstein relation. Following the steps of Tsimpanogiannis et al.,^697^ the analysis performed in ref 698 clearly indicates that the Stokes-Einstein relation is violated for both the experimental measurements and the MD simulation data. It is important to note that H_2_ self-diffusivities computed from MD simulations are subject to finite-size effects^688^ (see the relevant discussion in Section 3.2.3). The magnitude of these effects in the liquid phase depends on the temperature, number of molecules, and viscosity, as shown in Eq. ([Disp-formula eq15]).^498–500^ The finite-size effects for diffusivities of H_2_ in the aqueous phase are in the range 3-15% based on the studied by Tsimpanogiannis et al.,^688^ Habibi et al.,^112^ and Śmiechowski.^314^ Accounting for these effects is necessary to ensure the reproducibility of the MD data.

In a recent study Kerkache et al.,^699^ investigated H_2_ diffusivity in water and NaCl brine over a wide range of thermodynamic conditions, including temperatures from 298 to 473 K, pressures from 1 to 1000 bar, and salt molalities up to 6 mol salt/kg solvent, focusing on self-diffusion coefficients relevant to infinitely diluted H_2_ in aqueous solutions; the study primarily addressed H_2_-H_2_O and H_2_-H_2_O-NaCl systems. The results indicated that H_2_ self-diffusion coefficients decrease with increasing salinity, for example from approximately 6.0 × 10^–9^ m^2^/s in pure water to ca. 4.2 × 10^–9^ m^2^/s at 6 mol/kg NaCl at 323 K and 200 bar, and increase with temperature and slightly decrease with pressure. No data on mutual diffusivities for H_2_-CH_4_ or H_2_-CO_2_ mixtures were provided. The physical mechanism underlying these findings is linked to the increasing structuring of the solvent and ions around the H_2_ molecule, reflected by more negative two-body excess entropy values with higher salinity and pressure, leading to higher viscosity and thus lower diffusivity, while temperature weakens solvent structuring and enhances H_2_ mobility. The solvent viscosity, accurately captured by the TIP4P/2005 water model combined with the Madrid-Transport salt force field, was found to be the dominant factor influencing H_2_ diffusivity, consistent with the fractional Stokes-Einstein relation, having an exponent of ca. 0.83. The computed diffusion coefficients were compared qualitatively to limited experimental data available for H_2_ diffusion in pure water and low salinity brine, showing good agreement in trends though experimental data remain sparse and uncertain at higher salinities and pressures. Finally, the study proposed both a fractional Stokes-Einstein model and an Arrhenius-type empirical correlation for H_2_ self-diffusivity as functions of temperature, pressure, and NaCl molality, providing a reliable predictive framework for underground H_2_ storage applications.

Molecular simulations have also been used to compute the diffusivity of H_2_ in concentrated aqueous NaCl, NaOH, KOH, and NaB­(OH)_4_ solutions.^112,113,630,680^ The data collected from literature are shown in Fig 30. These electrolyte solutions are primarily relevant for water electrolysis and H_2_ storage (see also Section 2.1). As in the simulations of H_2_/water systems discussed earlier, it is important to choose salt/water force field combinations that can accurately model the densities and transport properties of the aqueous electrolyte solvents. For example, salt force fields that cannot capture the change in viscosity as a function of salt molality, it is expected that they also fail in modeling the diffusivity (and other properties) of H_2_, ions, or water at different salt loadings.^630^ Blazquez et al.^700^ has shown that non-polarizable force fields of NaCl and KCl that correctly predict the density and viscosity of the aqueous solution can accurately describe the diffusion coefficients of Na^+^, K^+^, Cl^–^, and water. Similarly, Avula et al.^701^ has shown that for predicting the anomalous increase in the water diffusivities in aqueous CsI solutions, the decrease in solution viscosities needs to be accurately captured. The data reported in the Supporting Information of van Rooijen et al.^630^ show that the Madrid-2019 NaCl^370^ force field overestimates the solution viscosities by ca. 20% at the solubility limit compared to Madrid-Transport,^384^ and therefore, underestimates the diffusivities of H_2_ in the aqueous solution.

**Figure 30:**
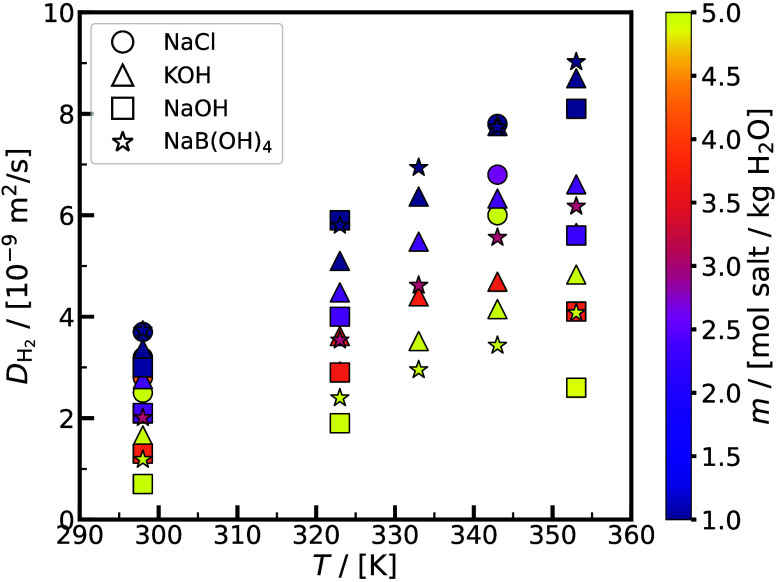
Self-diffusivities of H_2_ in aqueous NaCl, KOH, NaOH, and NaB­(OH)_4_ solutions at different temperatures and salt molalities at 1 bar. The data are collected from refs 112, 113, and 630.

#### 4.1.6 Electroosmotic Drag Coefficient

Din and Michaelides^127^ computed EOD within a cylindrical pore examining both a flow of water molecules or ions, and the case of no macroscopic flow in the channel. The interactions between water molecules and wall atoms, and the interactions between hydrogen atoms (protons) and wall atoms were determined based on the work of Zhu and Robinson.^702^ Din et al. established a linear connection between the EOD coefficient and water uptake. For *λ* ∈ [7.65, 10.9, 12.2, 20.9], the EOD computed equals [7.34, 10.5, 12.0, 19.6], respectively. These results are shown in Fig. 31. The overestimation of the EOD possibly arises from the simplified pore model used in the simulations, which assumes uniform wall charge, straight cylindrical pores, and neglects structural complexity of the membrane. As a result, the simulations allowed for more water to be dragged by protons than what is observed in experiments with membranes. In addition, the proton hopping mechanism is missing from the model of the authors.

**Figure 31:**
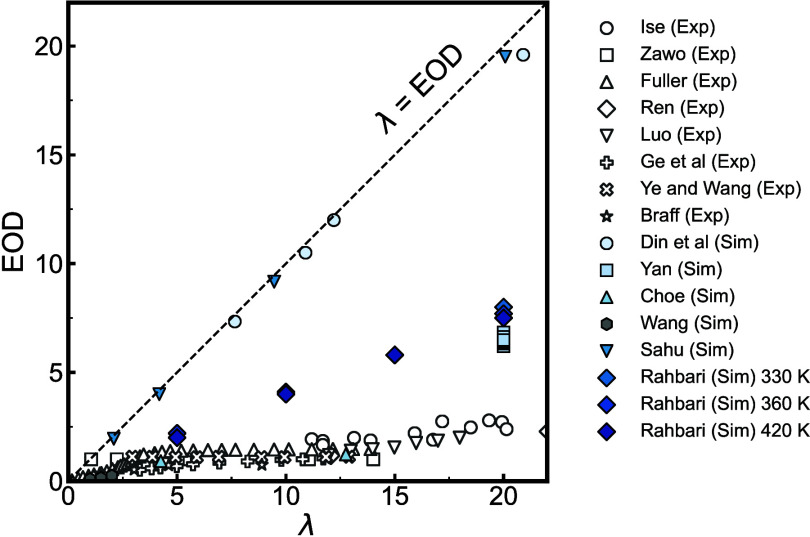
Electro-osmotic drag coefficients from MD simulations and experiments as a function of the hydration level, *λ* (defined in Eq. ([Disp-formula eq31])). The dashed line denotes the ideal case where EOD equals *λ*, serving as a reference. Data are compiled from refs 92, 94, 98, and 127–129.

Yan et al.^128^ performed atomistic MD simulations to compute the EOD coefficient with electric fields (up to 2 V/*μ*m) for *λ* = 20. All-atom force-fields were used to model the hydrated PFSA oligomers, water molecules, and H_3_O^+^.^128,703^ Every backbone comprised four side chains terminated with sulfonic acid groups. The EOD coefficient was computed using two approaches: one based on average transport velocities, and the other on peak shifting velocities. Both methods yielded comparable results.^128^ The EOD was computed over a temperature range of 287.15 K to 364.65 K, showing no significant temperature dependence. An average value of 6.41 was obtained. These values are notably higher than most experimental values as shown in Fig. 31. The authors attributed this overestimation to the absence of proton hopping in their model, which omits the dissociation of O-H bonds, and therefore, excluding a major transport mechanism. While the authors suggested a correction, the discussion was and not further supported by additional simulations or quantitative analysis. The proposed correction was, thus, a qualitative estimate rather than a rigorously derived result, limiting the strength of the comparison with experimental values.

Choe et al.^129^ computed the EOD coefficient of water at *T* = 363 K for *λ* values ranging from 4.25 to 12.75. Under a uniform electric field, the EOD coefficients were extracted from ab initio MD trajectories, yielding values of 0.92 and 1.23 for *λ* = 4.25 and *λ* = 12.75, respectively. These results are consistent with experimental measurements, as shown in Fig. 31. The model system comprised two Nafion monomers arranged around a central water cluster in a sandwich-like structure, with hydrophilic tails facing the water. The proton from the sulfonic acid end group was modeled as a hydronium ion. The Dreiding force field was used for intra-molecular interactions within Nafion, and UFF was applied for nonbonded interactions with water and hydronium (for more information on these force fields the reader is referred to Section 3.1.4). Classical MD simulations were first used to generate equilibrated configurations, which then served as initial states for AIMD. The authors attributed the nearly constant EOD across hydration levels to two opposing effects, i.e., the increased proton hopping at higher hydration, which tends to lower the drag, and the enhanced water mobility beyond the hydration shell, which tends to raise it. These effects largely offset each other, resulting in the observed invariance of the EOD with respect to water content. The study by Choe et al.^129^ clearly demonstrates that combining ab initio calculations with classical MD enables a more accurate representation of the underlying transport physics (as also recently shown in ref 105). Although this review does not elaborate on QM methods, it is worth noting that such approaches are necessary for accurately predicting H_2_ properties in electrochemical systems.

Rahbari et al.^94^ computed the EOD coefficient for hydrated Nafion membranes at water contents *λ* ∈ [5, 10, 15, 20] and temperatures between 330 and 420 K. The data reported in this study are shown in Fig. 31. The electric fields applied were in the range 0.05 to 0.10 V/Å while the velocity ratio of water to H_3_O^+^ ions was determined across different field strengths. PCFF and COMPASS force fields were used to model all interactions. This study showed that the average velocities of both species scaled linearly with the electric field, resulting in a constant velocity ratio and a linear dependence of EOD on hydration level. The EOD increased with *λ*, reaching up to 7.5 at *λ* = 20 and 420 K, while showing a slight decrease with temperature. As in the prior studies using classical force fields, proton hopping was not modeled, thus, limiting the accuracy of the computed EOD at high hydration. Nevertheless, the results at lower *λ* aligned better with experiments as can be observed in Fig. 31.

Wang et al.^98^ conducted classical MD simulations to compute the EOD in Nafion membranes with low hydration levels *λ* = 1, 1.5, and 2 at *T* = 350 K. The computed EOD coefficients were 0.098, 0.149, and 0.255, respectively. Electric fields ranging from 0.001 to 0.005 V/nm were applied, and EOD coefficients were determined from the average velocities of water and H_3_O^+^. The results are in excellent agreement with the linear model $\mathrm{EOD}=\frac{2.5}{22}\lambda$ , confirming the assumption made by Springer et al.^704^ that EOD varies linearly with water content at low hydration. This finding is also in line with most experimental and simulation data at low water contents as shown in Fig. 31. Similarly to the prior classical studies, ss the hopping mechanism was not included, the calculated EOD reflects only vehicular proton transport.^94,556^ The authors showed that EOD approaches zero as water content vanishes.

The linear increase of the EOD coefficient with hydration level, as predicted by classical MD simulation studies,^94,127^ was also confirmed by Sahu and Ali.^705^ Consistently with the prior studies, the approach did not include the Grotthuss mechanism (proton hoping). As a result, the computed EOD values were shown to overestimate experimental data, particularly in well-hydrated systems. This overestimation is further amplified by the use of high electric field strengths. The EOD values reported by Sahu et al. are shown in Fig. 31.

In summary, several studies have investigated the EOD in hydrated membranes using molecular simulations (see Table 10), providing valuable insights into the underlying transport mechanisms and associated limitations. Collectively, these studies show that while classical force fields can qualitatively reproduce trends in EOD, quantitatively accurate predictions, particularly at high hydration, require explicit modeling of proton hopping and a more realistic description of membrane nanostructure. It is also important to note that there remains considerable variability among experimental EOD measurements; however, a detailed discussion of experimental inconsistencies is beyond the scope of this review. Nevertheless, the major differences and systematic trends between simulation and experimental data can still be identified.

**Table 10:** Summary of Studies Calculating the EOD from Molecular Dynamics Simulations

		System Details		Thermodynamic Conditions		
Year	Study	Phases	Force Fields	Electric Field V/Å	Temperature	Method
1998	Din et al^127^	Nafion(s), H_2_O(l), H^+^(aq)	SPC/E, H^+^,^127^ Wall atoms (Nafion)	0.0592 to 0.3809	298	NEMD
2008	Choe et al.^129^	Nafion(s), H_2_O(l), H_3_O^+^(aq)	SPC/E, Nafion (Dreiding, UFF)	0.05	363	NEMD/Ab initio
2008	Yan et al.^128^	Nafion(s), H_2_O(l), H_3_O^+^(aq)	TIP3P, H^+^,^127^ Nafion	0.02 to 0.05	287.15-364.65	NEMD
2022	Wang et al.^98^	Nafion(s), H_2_O(l), H^+^(aq)	SPC/E, Nafion (AMBER/GAFF)	0.0001 to 0.0005	350	NEMD
2022	Rahbari et al.^94^	Nafion(s), H_2_O(l), H_3_O^+^(aq)	PCFF (Class II), COMPASS (partial charges)	0.02, 0.05, 0.075, 0.100	330, 360, 420	NEMD
2025	Sahu et al.	Nafion(s), H_2_O(l), H_3_O^+^(aq), Cu^+^, Cu^2+^, Cl^–^	TIP4P/2005 (H_2_O), DFT-derived charges (Nafion), LJ + Coulomb (ions), Nafion model from TURBOMOLE	0.00, 0.02, 0.05, 0.075, 0.10	298	NEMD (classical MD)

#### 4.1.7 Reactive Systems and Membrane Properties Related to Hydrogen Research

Despite the fact that in this review we primarily focus on the computation of thermophysical propertiers using classical molecular simulations, in many H_2_ processes chemical, reactions cannot be ignored to obtain reliable predictions.^706^ To this purpose, reactive MD and MC simulations allow for the study of chemical reactivity by explicitly modeling bond formation and breaking, providing a pathway towards understanding complex phenomena in H_2_ (and many other) systems. These methods are particularly useful for studying the VLE of reactive systems, the internal workings of MEAs in fuel cells and electrolyzer cells, or any H_2_ system where chemical reactions play a crucial role.^707–710^ An important example in the context of H_2_ systems is the application of reactive MD in modeling ion-exchange membranes, where proton hopping mechanisms, the lifetime of H_3_O^+^, and the dynamic behavior of reactive species in electrolyte environments are central mechanisms.^118^ Unlike classical MD, which relies on predefined force fields (see Section 3.1), reactive MD uses techniques that allow for changes in molecular topology during the simulation. Reactive MD can be implemented within different modeling frameworks, including hybrid quantum-classical models, fully classical approaches, and quantum-based reactive force fields.^707,711^

The prediction of thermodynamic and transport properties of H_2_, H_3_O^+^, and protons in MEAs (see Section 2.1) also requires the use of combined classical/reactive molecular modeling approaches. Relevant properties in such systems include ion diffusion, electrical conductivity, membrane structure, EOD, and thermal conductivity. As discussed in the previous section, accurate modeling of the membrane, serving as the medium for H_2_ transport, is essential for understanding and optimizing system performance. Although a detailed analysis of MEAs lies outside the main scope of this review, selected membrane properties relevant to H_2_ applications are discussed here to illustrate the depth of insight that can be obtained via molecular simulations.

The review by Arntsen et al.^93^ explores the application of reactive MD simulations to study proton transport in polymers commonly used in fuel cells (Nafion 3M and Hyflon). The authors particularly highlight the Multistate Empirical Valence Bond (MS-EVB) method, which can capture the Grotthuss mechanism that classical non-reactive MD simulations fail to model. The application of MS-EVB simulations revealed an anti-correlation between vehicular and Grotthuss transport mechanisms. This anti-correlation was already hinted in Section 4.1.6 and Fig. 31 where our analysis of the collected simulation results revealed that excluding Grotthuss mechanism leads to overpredicting the EOD. The review by Arntsen et al.^93^ demonstrates how H^+^ conductivity computed from MS-EVB simulations aligns more closely with experimental data,^93^ showcasing the accuracy of the MS-EVB method. This study not only captured proton hopping, but also proposed a transport mechanism in which H^+^ primarily pass between sulfonate groups, rather than diffusing through the bulk water region.

Awulachew and Nigussa^115^ conducted MD simulations of MEAs encountered in fuel cells considering Pd_3_Ag electrodes as alternatives to Pt electrodes. Self-diffusion coefficients were computed using the Einstein approach (see Section 3.2.3) to determine H^+^ conductivity, while coordination numbers were obtained from radial distribution functions. The conductivity values obtained were comparable to, but slightly higher than, those reported in the literature. A benchmark comparison was performed, and it was concluded that the combination of a Nafion membrane with a Pd_3_Ag electrode offers an efficient and cost-effective solution for MEAs. The authors discussed the influence of hydrogen bonding and temperature on H^+^ mobility. The authors proposed that including interaction effects between H^+^, the electrode, and the electrolyte improves the computation accuracy of H^+^ conductivity. Cui et al.^116^ performed classical MD simulations of hydrated Nafion membranes at hydration levels corresponding to *λ* = 3.44, 5.42, 8.63, and 11.83. Water clustering and H_3_O^+^ diffusion were analyzed as functions of hydration. This study revealed that increasing water content promotes the formation of a connected aqueous network composed of interlinking water channels. Based on the molecular structure, the formation of Eigen-type ions is proposed as hydration increases. The authors attribute the low H^+^ conductivity at low water contents to the disconnected morphology of the water network.

Venkatnathan et al.^117^ performed MD simualtions to compute the density and diffusion coefficient of H_3_O^+^ in a nafion membrane at hydration levels *λ* = 3.5, 6, 11, 16, and temperatures of 300 and 350 K. Consistently with the prior studies, diffusion coefficients were underestimated due to the lack of proton hoping in classical MD framework. The effects of hydration on the structure and vehicular transport of ions and the mobility of water molecules in Nafion membrane were discussed. The authors proposed that H_3_O^+^ ions do not merely act as passive charge carriers but actively shape the local structure of the hydrated Nafion membrane. At low hydration levels, participation of H_3_O^+^ in forming hydrogen bonds constrains the mobility of sulfonate, effectively affecting the morphology of the membrane. As hydration increases, these constraints are relaxed due to enhanced solvation, allowing sulfonate groups and H_3_O^+^ to become more spatially separated.

Hofmann et al.^118^ performed reactive MD simulations to compute the diffusion and conductivity of oxygen and hydrogen ions in hydrated Nafion membranes at *λ* = 6 and *λ* = 12. The simulations indicated shorter H_3_O^+^ lifetimes at higher hydration levels. The authors proposed that the time–determining step for proton transfer is the formation of a precursor complex (Zundel-type) prior to rapid proton hopping. This study also showed that the conductivity significantly exceeds the value expected from self-diffusion alone, highlighting the role of collective proton motion. This is reflected in a reduced Haven ratio at higher hydration, indicating enhanced correlated transport and increased conductivity.

Brunello et al.^120^ performed fully atomistic MD simulations to study the structure, sulfonate solvation, density, and water transport in hydrated sulfonated poly­(ether ether ketone) (S-PEEK) at temperatures of 298 to 353 K, and *λ* = 4.9 and 11.1. The simulations revealed that temperature has limited impact on sulfonate solvation and morphology, while water content significantly enhances nano-phase segregation and the development of water channels. Water diffusion increased with temperature and hydration, though activation energies remained similar, indicating a consistent diffusion mechanism. While explicit experimental validation is not provided, the authors point to qualitative consistencies with previous findings on membranes such as Nafion and Dendrion, suggesting physically plausible trends in structure and transport behavior.

Lins et al.^122^ performed MD simulations of fully sulfonated Ph-SPEEKK to evaluate the influence of hydration level (*λ* = 3.5, 6, 11, 16, 25, 40) on the internal structure of the membrane and H^+^ transport. The authors computed diffusion coefficients for water and H_3_O^+^, as well as hydrogen bond statistics. In the absence of direct experimental data for Ph-SPEEKK, the pair correlation functions was used to assess sulfonate clustering, which revealed that higher hydration leads to greater sulfonate mobility and separation. While explicit validation was not deemed possible at the time of the publication, qualitative agreement with prior findings on SPEEK and Nafion was noted, particularly in hydration–dependent structural trends and the enhancement of diffusion at higher *λ*.

Ozmaian and Naghdabadi^101^ computed the diffusion coefficient of water in hydrated Nafion with *λ* = 14 at temperatures ranging from 100 to 600 K. The authors investigated the glass transition temperature (*T*_g_) of hydrated Nafion to better understand its thermomechanical stability and performance limits, particularly for applications such as fuel cells and shape–memory devices. This study showed taht increasing hydration significantly lowers *T*_g_; for example, at *λ* = 20, *T*_g_ = 230 K, while for *λ* = 2, no clear transition is observed below 500 K. Overall, the simulations results show good consistency with experimental *T*_g_ values (340 - 350 K). An important insight finding in this study was that membranes with hydration gradients may exhibit heterogeneous thermal and degradation behavior across their thickness, which is relevant for their operational durability.

Cha^124^ performed MD simulations to investigate how the morphology of perfluorosulfonic acid (PFSA) membranes, specifically, the length of side chains, affects H_3_O^+^ conductivity in polymer electrolyte membranes (PEMs), which constitutes a key performance factor in fuel cells. The study was motivated by discrepancies between prior simulation and experimental studies, which reported conflicting results on whether shorter side-chains improve conductivity. To address this, Cha^124^ developed tracked H_3_O^+^ pathways throughout the simulation. The findings showed that shorter side-chains increase the fraction of conductive H_3_O^+^, and enhance inter-chain ion movement, thus, improving ionic conductivity, contrary to some earlier experimental observations. Despite the motivation of this study, the discrepancies between the simulation and experimental results were not resolved, and follow-up research is proposed.

Flottat et al.^125^ conducted MD simulations to elucidate why Aquivion (850 g/equiv) exhibits higher water diffusion than Nafion (850 g/equiv). Motivated by the fact that H^+^ conductivity depends on the morphology of the hydrophilic water network (which itself is strongly influenced by hydration), the authors investigated how nanoscale structure governs transport properties. The simulations were shown to reproduce experimental trends for membrane density and water diffusion, while a linear increase in water domain size with hydration was obtained, in qualitative agreement with experimental measurements. A key finding was that the transition from isolated to percolated water networks occurs at a lower hydration level in Aquivion than in Nafion, potentially explaining improved diffusion/conductivity under dry conditions. The study also showed that structural and dynamic properties scale with water volume fraction, identifying it as a more fundamental parameter than hydration number.

Zhang et al.^95^ investigated the effects of Na^+^ and Ca^2+^ ions on the diffusion of H_3_O^+^ and water in Nafion 117 membranes using MD simulations. The study revealed that both cations significantly reduce H_3_O^+^ and water diffusivities by associating with sulfonic acid groups and disrupting water cluster connectivity, especially at *λ* = 16 and temperatures of 298 and 353 K. Ca^2+^ appeared to have a stronger effect than Na^+^ due to the higher charge and higher hydration degree, leading to stronger binding with sulfonic groups and greater disruption of water channels. Diffusion coefficients reported for all species confirm that Na^+^ diffuses more rapidly than Ca^2+^. The decline in H_3_O^+^ and H_2_O transport aligned with previous experimental and simulation findings. The computed diffusion of Na^+^ was also shown to be in line with with the respective experiments.

This section highlighted how molecular simulations can be used to obtain deep mechanistic insights into the transport and thermodynamic behavior of H_2_-related species in hydrated polymer membranes. By considering water content, temperature, ionomer morphology, and contaminant effects, molecular simulations not only provide a means of computation of diffusion coefficients and conductivities but also reveal nano-scale structural phenomena, such as water channel formation, sulfonate-cation interactions, and the role of hydration in enabling or restricting ionic transport. The representative studies reviewed above demonstrate the power of molecular simulations in studying experimentally inaccessible systems/conditions, provided that accurate force fields and methods are used.

Table 11 provides a succinct summary of the key insights obtained from the relevant literature. While our focus here was mainly on transport properties which are relevant to H_2_ applications, molecular simulations can also be used to study other membrane features such as thermal conductivity^126^ or the impact of contamination on oxygen transport.^96^ These topics lie outside the scope of this review but further illustrate the wide applicability of molecular simulation in membrane research.

**Table 11:** Brief Overview of Mechanistic Insights Obtained from Molecular Simulation Studies of Hydrated Membranes Relevant to Hydrogen Systems^a^

Reference	Property Studied	Key Insights
Flottat et al.^125^	Water diffusion in Nafion vs. Aquivion	Linear growth of water domains with hydration; earlier percolation in Aquivion; water volume fraction governs structure and dynamics
Awulachew and Nigussa^115^	Proton conductivity in MEAs with Pd_3_Ag electrodes	H-bonding enhances proton mobility; electrode-electrolyte interaction boosts conductivity; Pd_3_Ag-Nafion outperforms Pt; simulation exceeds literature values
Cha^124^	Side chain length effect on H_3_O^+^ transport	Shorter side chains improve inter-chain mobility and ion conductivity; contradicts some experimental trends
Zhang et al.^95^	H_3_O^+^ and H_2_O diffusion in Nafion	Na^+^, Ca^2+^ disrupt water clusters; Ca^2+^ has stronger binding and impact; reduced H_3_O^+^, H_2_O mobility
Arntsen et al.^93^	Review of proton transport in fuel cell membranes	MS-EVB captures Grotthuss mechanism; anti-correlation with vehicular transport; hopping occurs between sulfonates; matches experiments
Ozmaian and Naghdabadi^101^	Glass transition temperature of Nafion	Hydration lowers *T_g_* significantly; hydration gradients lead to heterogeneous degradation behavior
Brunello et al.^120^	Water transport in S-PEEK	Water content enhances phase segregation and water channel development; consistent with Nafion trends
Lins et al.^122^	Structure and transport in Ph-SPEEKK	Hydration increases sulfonate mobility and spacing; qualitative match with Nafion/SPEEK behavior
Hofmann et al.^118^	Proton transfer mechanisms	High *λ* shortens H_3_O^+^ lifetime; precursor (Zundel-type) forms before hopping; collective effects increase conductivity
Venkatnathan et al.^117^	Density and diffusion in Nafion	H_3_O^+^ shapes local structure; low *λ* restricts mobility via H-bonding with sulfonates
Cui et al.^116^	Water clustering and hydronium diffusion	Hydration enables channel formation; Eigen-ion structures proposed; disconnected morphology limits conductivity at low *λ*

a The table summarizes key findings on structure-transport relationships, ion coordination, and hydration-dependent phenomena in membrane electrode assemblies.

### 4.2 Thermodynamic and Transport Properties of Nonaqueous Hydrogen Mixtures

#### 4.2.1 Vapor-Liquid Equilibrium of Pure Hydrogen and Binary and Ternary Mixtures of Hydrogen with Argon, Nitrogen, and Carbon Dioxide

Köster et al.^48^ studied various thermophysical properties (including VLE, vapor and liquid densities, and residual enthalpy of vaporization) of binary H_2_-N_2_ mixtures using the grand equilibrium method (see Section 3.3.1.6). The simulations were conducted at *T* = [83.15, 100, 110.3] K, with corresponding pressure ranges of [(0.196–14.1), (0.794–10.20), (1.51–8)] MPa. To model H_2_, the Vrabec^48^ and Marx^310^ force fields were used. The results, shown in Fig. 32a, demonstrate that the Vrabec model, which was fitted to *pVT* and speed of sound data, is in good agreement with experimentally measured VLE data across the whole temperature range considered, outperforming Marx. Deviations are observed as pressure increases. Vapor pressures and saturated liquid densities are accurately captured, particularly at lower temperatures. The Vrabec force field is also shown to yield improved predictions of relative volatilities and residual enthalpies of vaporization compared to Peng–Robinson, SRK, and PC-SAFT EoS (for the PC-SAFT predictions the reader is referred to ref 48), which exhibit significant deviations from reference data in the critical and dense liquid regions. This force field also performs well in predicting homogeneous phase densities of equimolar H_2_-N_2_ mixtures under cryogenic and near-critical conditions, with mean absolute percentage errors below 1% in some cases.^48^

**Figure 32:**
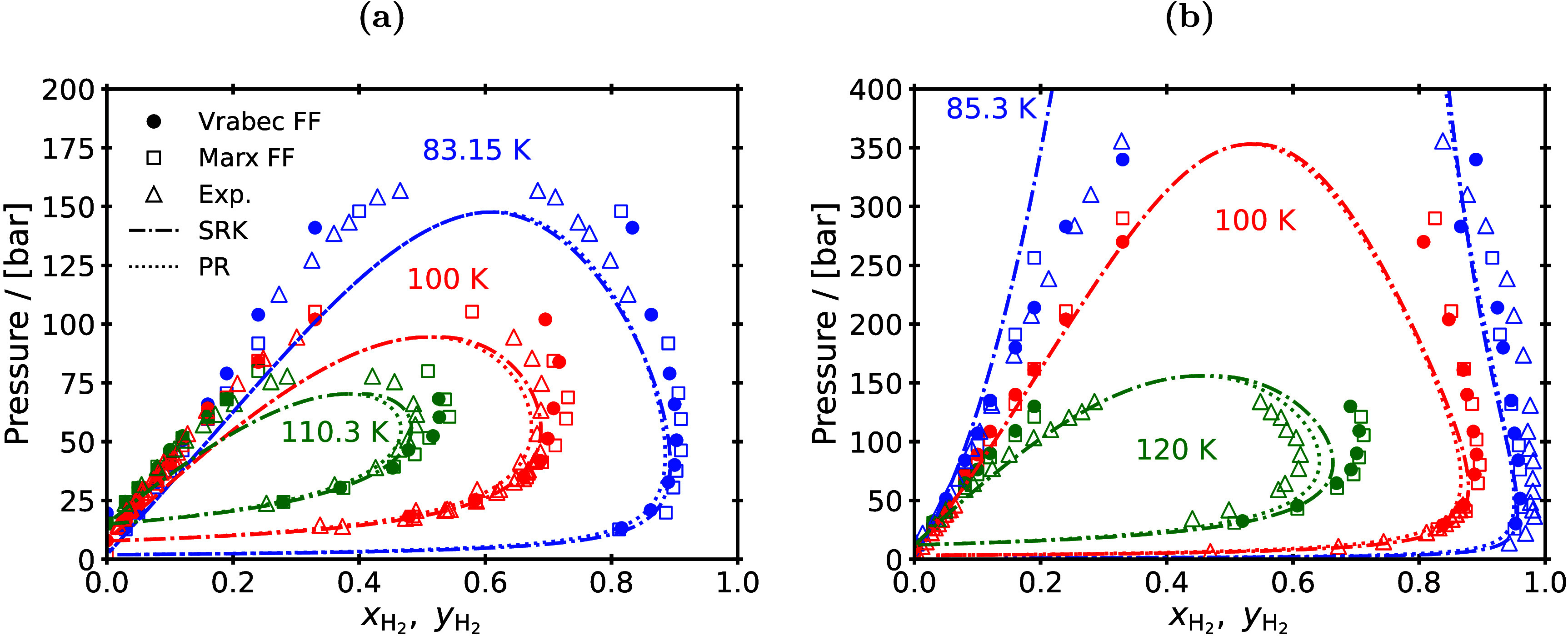
Comparison of molecular simulation results with experimental VLE data for the (a) H_2_-N_2_ and (b) H_2_-Ar binary mixtures using the Vrabec and Marx hydrogen force fields, adapted from Köster et al.^48^ The experimental data^712–714^ are also presented in Köster et al.^48^ The PR and SRK predictions are computed with the Open-Source Fluid Thermodynamics Toolkit Clapeyron.^715^ The simulation results of H_2_-Ar at 120 seem to deviate significantly from the experimental results, however, these measurements were performed at 122.73 K instead of 120 K.^714^

In the same study, Köster et al.^48^ performed simulations also for H_2_-Ar binary mixtures to compute the same properties (VLE, densities, and residual enthalpy of vaporization). For this binary mixture, the authors considered *T* = [85.8, 100, 120] K, with corresponding pressure ranges of [(0.090–34), (0.331–27), (1.220–15.6)] MPa for the Vrabec force field, and [(0.088–25.644), (0.331–29), (1.220–14.3)] MPa for the Marx force field. As shown in Fig. 32b, molecular simulations using the Vrabec^48^ force field, demonstrated good agreement with the available experimental VLE data. In particular, the saturated liquid compositions and vapor pressures at cryogenic conditions (90–120 K) are well captured. Although minor deviations were noted in the saturated vapor compositions at lower temperatures, the predictions from the molecular simulations remained within the scatter of the experimental data. Furthermore, in the homogeneous supercritical region, the computed compressibility factors matched experimental values with high accuracy, achieving mean absolute percentage errors below 1% for pressures up to 9 MPa. These findings highlight the reliability of Vrabec force field in describing both phase behavior and volumetric properties of H_2_-N_2_ and H_2_-Ar mixtures under sub- and super-critical conditions.

Raju et al.^716^ computed the VLE of the H_2_-CO_2_ binary mixture using molecular simulations in the CFCGE ensemble (see Section 3.3.1.1). The authors compared the simulation results with data from the GERG-2008 ^685^ EoS and available experiments. The results are illustrated in Fig. 33. Notably, the authors faced convergence issues with GERG-2008 at pressures exceeding 170 bar, as solid lines in Fig. 33 indicate. Moreover, the computed bubble points from GERG-2008 showed significant discrepancies from the experimental measurements. In contrast, the mole fractions obtained from molecular simulations exhibited good agreement with the experimental data by Tsang and Street,^717^ with relative deviations remaining below 5% even at elevated pressures.

**Figure 33:**
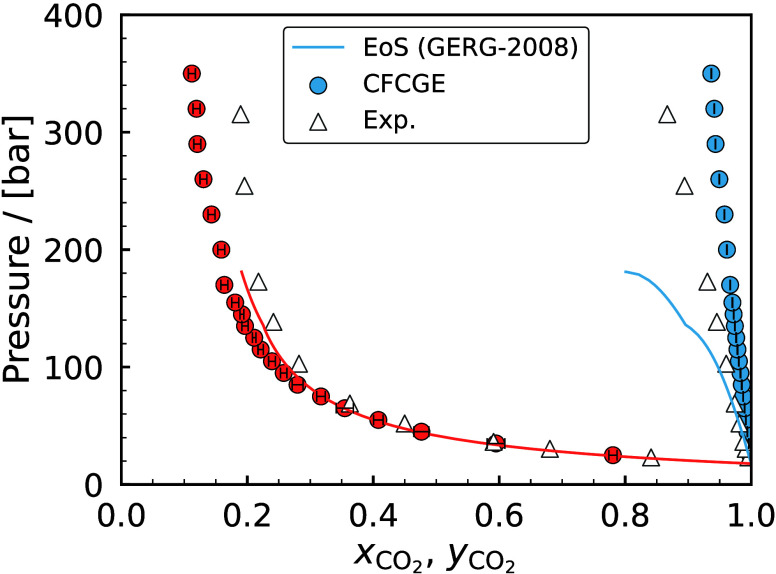
Vapor-liquid equilibrium data for the CO_2_-H_2_ binary mixture from molecular simulations^716^ compared to GERG-2008 ^685^ EoS predictions and experimental measurements.^717^

The VLE of the ternary H_2_/Ar/N_2_ system at 100 K was studied by Köster et al.^48^ using the force field fitted in the same study. As shown in Fig. 34, the simulations accurately captured the vapor and liquid compositions for both pressures of 20 and 30 bar. A particularly large two-phase region is observed due to the presence of H_2_. The simulation results show excellent agreement with experimental data, while cubic EoS (SRK and PR) were shown to underestimate the extent of phase separation, and predicted narrower two-phase envelopes. Consistent with the accurate predictions for the binaries discussed earlier, Vrabec force field was also shown to perform well in predicting also the ternary phase behavior of these light gas mixtures.

**Figure 34:**
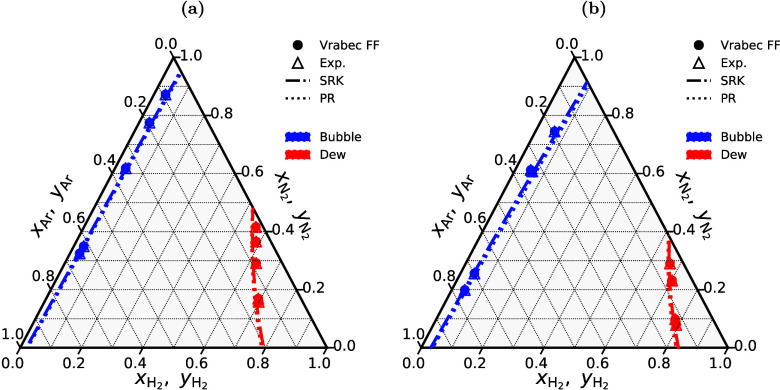
Ternary phase diagrams for the H_2_/Ar/N_2_ system at 100 K and (a) 20 bar or (b) 30 bar. The simulation data are collected from Köster et al.,^48^ the experimental measurements from Xiao et al.,^712^ and the SRK and PR EoS datapoints are computed with Clapeyron.^715^

#### 4.2.2 Pressure–Volume–Temperature and Thermodynamic Factor Computations for Hydrogen, Methane, and Carbon Dioxide Mixtures

Zhang et al.^718^ performed MD simulations to compute the pressure of pure H_2_, CO_2_, CH_4_, and their binary mixtures at various temperatures and molar volumes. The results, corresponding to a molar volume of *V*_m_ = 0.143 L mol^–1^, are shown in Fig. 35. For pure-component simulations, the authors reported that the data from MD are in excellent agreement with predictions from GERG-2008 EoS and NIST. However, the reference provided by Zhang et al.^718^ (ref 54) for the NIST database does not directly lead to the respective data. The molecular simulation data were subsequently used to parameterize new, computationally efficient analytical equations describing the *pVT* behavior of H_2_/CO_2_/CH_4_ mixtures at pressures up to 1720 bar and temperatures in the range of 310.9 to 470 K.

**Figure 35:**
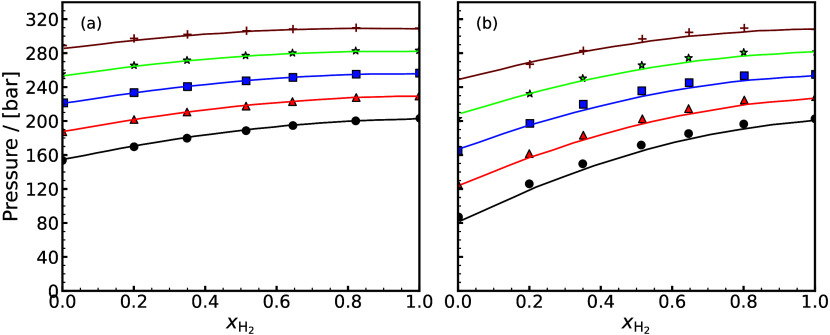
Pressure as a function of mole fraction of H_2_ at different temperatures for (a) H_2_-CH_4_ and (b) H_2_-CO_2_ binary mixtures. EoS data are shown as lines (black: 310.9 K, red: 350 K, blue: 390 K, green: 430 K, brown: 470 K). Molecular simulation data are shown as symbols (black circles: 310.9 K, red triangles: 350 K, blue squares: 390 K, green stars: 430 K, brown crosses: 470 K).

Zhang et al.^718^ reported excellent agreement with available experimental data^719^ at temperatures between 300 and 350 K, for 5 to 50% H_2_ in CH_4_ (see Fig. 36). Also, the *PρT* MD data at 673.1 K are in excellent agreement with the available experiments^720^ for the mixture 0.6992 H_2_ + 0.3008 CO_2_ as shown in Fig. 36. The authors proposed further developing analytical EoS for other binary or multicomponent gas mixtures using MD simulations, potentially augmented with machine learning.

**Figure 36:**
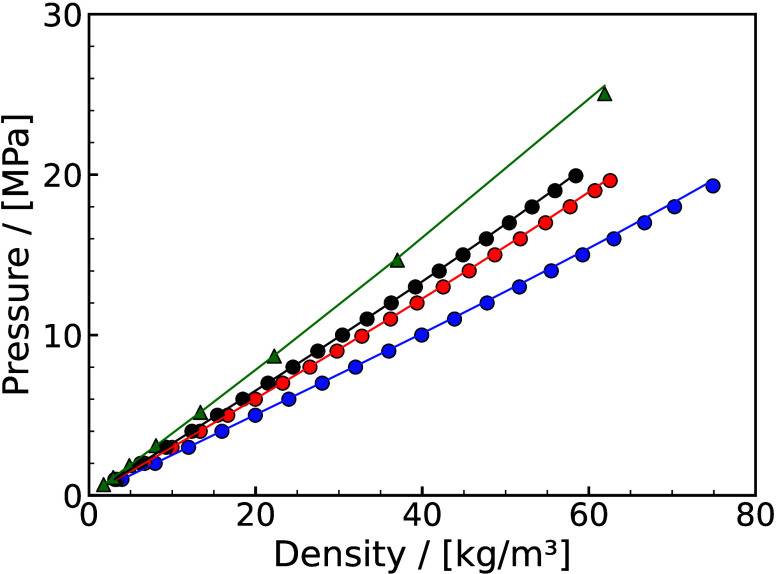
Pressure - density diagrams of an equimolar mixture of H_2_ and CH_4_ (black, red, and blue circles are molecular simulation data from ref 718. Black, red, and blue lines represent experimental data from ref 719). Additional comparisons for mixtures with H_2_ mole fractions of 0.05 and 0.10 are provided in the Supporting Information of ref 718. Here, a H_2_-CH_4_ mixture with H_2_ mole fraction of 0.6992 H_2_ at 673.1 K is also shown (simulation data, represented as green triangles are from ref 718 and experimental data, shown as green line, are from ref 720).

In the recent studies by Hulikal Chakrapani et al.,^576,677^ the Marx^310^ force field was combined with TraPPE^721^ to model the densities, Gibbs excess energies, and thermodynamic factors of H_2_/CO_2_ mixtures. For an extended collection of raw simulation data for these properties, but also viscosities, compressibility factors, and diffusivities, the reader is referred to the Supporting Information of refs 576 and 677. The authors presented a wide and reliable collection of simulation data spanning gaseous densities of H_2_/CO_2_ mixtures in the range of 298-423 K, 0-1 mole fraction of H_2_, and for pressures up to 500 bar.^677^ The thermodynamic factors of H_2_/CO_2_ systems were computed at 323 K (using CFCMC simulation - see Section 3.3.1.4), and were shown to have excellent agreement (within 3%) compared to values obtained using REFPROP.^352^ Hulikal Chakrapani et al.^576^ also showed that thermodynamic factors can be computed from simulations of small system sizes (of ca. 100 molecules) even for densely packed systems using the proposed method.

Barraco et al.^309^ systematically compared eight classical LJ-based rigid molecular models of pure H_2_ gas under thermodynamic conditions relevant to on–board H_2_ storage in tanks. The selected temperatures (-50 °C to 90 °C) and pressures (50 to 2000 bar) reflect specifications provided by H_2_ tank manufacturers, ensuring that the models are evaluated under realistic, high pressure, operating scenarios. A total of 600 MD simulations were conducted across twenty–five experimentally derived H_2_ gas densities per temperature (-50 °C, 20 °C, and 90 °C). For each model, the study presented data for the *pVT* and phase behavior, and self-diffusivities. The raw simulation data are available in the original work.^309^ While all models exhibited nearly identical diffusion behavior, clear differences emerged in reproducing phase behavior and solubility, especially at high densities. Among the force fields tested, the two-site model by Yang and Zhong^325^ and the single-site model derived from Buch^312^ performed most consistently across all conditions. For a detailed discussion on H_2_ force fields see Section 3.1.1.

#### 4.2.3 Density, Thermal Expansivity, Heat Capacity, Joule-Thomson Coefficient, and Isothermal Compressibility of the Hydrogen-Light Gas Mixtures

Raju et al.^716^ performed MC and MD simulations to compute various thermodynamic properties of CO_2_-rich mixtures with different gases (N_2_, Ar, CH_4_, and H_2_) at mole fractions ranging from 0.01 to 0.10 (impurity level), exploring a broad spectrum of state points. The authors used well-known force fields previously validated for the pure components. For an extensive collection of raw simulation data and respective figures, the reader is referred to the main manuscript and Supporting Information of ref 716. To illustrate key trends without redundancy, here, we focus on the binary CO_2_-H_2_ system at a H_2_ mole fraction of 0.05, comparing the data from molecular simulation with predictions from the GERG-2008 EoS obtained from REFPROP. Density predictions closely follow GERG-2008 EoS values as shown in Fig. 37. Under these conditions, the maximum relative deviation is approximately 4.4% in the MC simulation at 313 K and 60 bar, 4.3% in the MD simulation at 253 K and 80 bar, 2.1% in MC at 253 K and 40 bar, and 6.6% in MD at 313 K and 60 bar. Notably, in Fig. 37 the GERG-2008 EoS data are absent for 253 K (near the critical region), reflecting the known limitations of the EoS in that regime.

**Figure 37:**
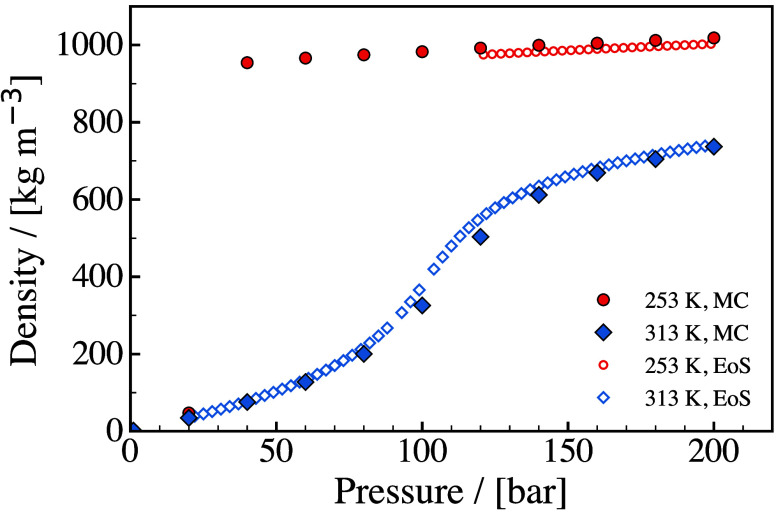
Density of CO_2_-H_2_ mixture as a function of pressure at a fixed CO_2_ mole fraction of 0.05 for two temperatures. The EoS data are from GERG-2008 via REFPROP. Raw simulation data are provided in ref 716.

Raju et al.^716^ also showed that CO_2_-rich mixtures with H_2_ gas as impurity, exhibit the largest reductions in thermal-expansion coefficients, isothermal compressibilities, isobaric heat capacities, and Joule-Thomson coefficients in the gas phase, followed, in decreasing order of effect, by N_2_, Ar, and CH_4_. Conversely, in the liquid and supercritical regions, H_2_ impurities cause the greatest increases in these properties, again with N_2_, Ar, and CH_4_ having progressively smaller impacts. In contrast to these findings, the addition of H_2_ raises gas phase speed of sound, but lowers this property in the liquid and supercritical phases. The respective data are shown in Fig. 38. As can be seen in Fig. 38(a), both the MC simulations and GERG-2008 EoS capture the pronounced maximum in the thermal-expansion coefficient of the CO_2_-H_2_ mixture at 313 K, as the pressure approaches saturation. At 253 K, the EoS fails to converge in the vicinity of the critical point (hence the missing data), whereas the MC simulations produce valid fluctuations, consistent with the absence of any phase split. The highest discrepancy between simulation and EoS occurs at approximately 120 bar and 313 K (very close to the saturation line of the mixture). Similar peaks are observed for the heat capacity (Fig. 38(b)) and the compressibility factor (Fig. 38(c)). For heat capacities near the saturation pressure, a sharp increase in both the EoS and MC results can be clearly observed. Due to the proximity to saturation, and the resulting spontaneous density fluctuations, sampling becomes more computationally expensive, as reflected by the larger error bars. Therefore, the exact peak value cannot be predicted by MC simulations, unless prohibitively large system sizes are used. In ref 716, it is further concluded that the Joule-Thomson coefficient of CO_2_ decreases in the presence of impurities. The JT coefficients for the CO_2_-H_2_ mixture are shown in Fig. 38(d).

**Figure 38:**
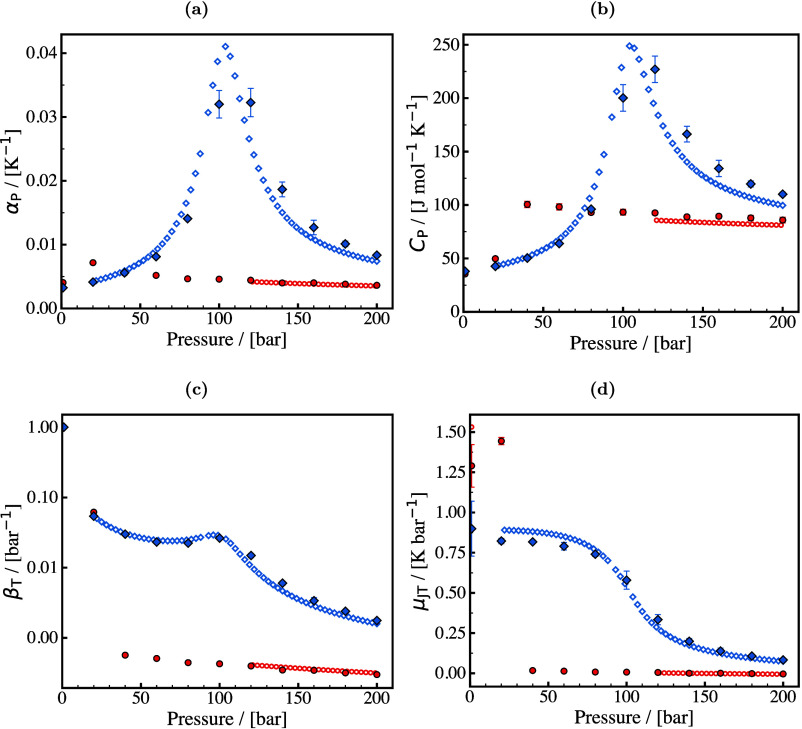
Thermodynamic properties of the CO_2_-H_2_ mixture at fixed H_2_ mole fraction of 0.05: (a) Thermal-expansion coefficient, showing peaks near the saturation pressure at 313 K. (b) Isobaric heat capacity, which surges near saturation, showing increased sampling uncertainty. (c) Isothermal compressibility, also showing maxima at saturation. (d) Joule-Thomson coefficient. In all panels, MC simulation (ensemble-fluctuations) results are plotted with filled colored markers, and GERG-2008 EoS with white-filled markers. Red and blue colors represent 253 and 313 K, respectively.

#### 4.2.4 Self-Diffusivity of Hydrogen in Hydrocarbons

The diffusivity of H_2_ in hydrocarbons is a crucial property for industrial processes such as Fischer-Tropsch, coal liquefaction, hydrotreating, hydrogenation, liquid organic hydrogen carriers, and separation of gas mixtures. The diffusivities of H_2_ in heavy alkanes can be used to evaluate the mass transfer limitations in chemical reactors, which operate in conditions ranging from 450–550 K and 20–80 bar.^722^ As for the diffusivities of H_2_ in aqueous solutions (see Section 4.1.5), experimental data of H_2_/hydrocarbon systems at a wide range of relevant conditions are scarce. Therefore, analytical models, such as modified Stokes-Einstein relations, are used to estimate these diffusivities. To this end, MD simulations are used to predict both self- and mutual diffusion coefficients for different H_2_/hydrocarbon mixtures.

Makrodimitri et al.^722^ investigated the self-diffusivities of H_2_, CO, and H_2_O in alkane mixtures with different chain lengths, ranging from *n*-C_12_ to *n*-C_96_. The TraPPE^301^ and Hirschfelder^321^ force fields were used to model the alkanes and H_2_, respectively (see more details on these force fields in Section 3.1). The choice of force fields was based on the fact that TraPPE can accurately model the critical point, densities, and melting temperatures for pure solutions and mixtures of alkanes over a wide temperature range, while the Hirschfelder model is simple (only LJ interactions) and accurate for pure H_2_ gas properties. While TraPPE force field explicitly accounts for intramolecular interactions and the bond/angle/dihedral changes (modeled using harmonic potentials) for alkanes, for smaller molecules such as H_2_, CO, and H_2_O intramolecular interactions and bond/angle changes are usually not considered as the vibrational and rotational relaxation times are significantly smaller than the time scales relevant for diffusion. Makrodimitri et al.^722^ validated the diffusivities of H_2_ and CO in pure C_12_, C_16_, and C_28_ alkanes and obtained an agreement of ca. 10% compared to the respective experiments. MD simulations were then used to generate data for self-, Maxwell-Stefan, and Fickian diffusivities of H_2_ alkane solutions. The self-diffusivities of H_2_ in alkane solutions at constant temperatures and pressures were shown to decrease with the increase in the alkyl chain length. This is expected since large chain alkanes have high viscosities. The authors used SAFT EoS to compute thermodynamic factors and, thereby, the Fick diffusivities of H_2_ and CO in C_12_ and C_28_ solutions at 473 K (see the method in Section 3.2.4).

Giraudet et al.^723^ performed dynamic light scattering (DLS) experiments and MD simulations to measure/compute self- and mutual diffusivities of binary gas/*n*-alkane mixtures. The authors reported H_2_ self-diffusivities in C_6_ and C_10_ for temperatures spanning 303–423 K (at pressures of ca. 40 and 10 bar for C_6_ and C_10_, respectively). The H_2_ mole fractions in all mixtures were approaching infinite dilution. The Darkrim^327^ and Hirschfelder^321^ force fields were used to model H_2_, while for the alkanes the L-OPLS^420^ force field was used. As shown in Fig. 39, the agreement between computed and experimentally measured diffusivities of H_2_ in light alkanes is relatively high. Among the gases studied by Giraudet et al.,^723^ H_2_ showed distinctly higher diffusivities (e.g., compared to mixtures containing N_2_ or CO), which follow an Arrhenius-like behavior. Consistent with other studies in literature, diffusivity decreased with increasing carbon chain length of the *n*-alkanes. The authors used the experimental and simulation data to develop semi-empirical correlations (based on the model of Wilke and Chang^724^) with parameters being the temperature, solute and solvent molar masses, and the viscosity of the solvent.

**Figure 39:**
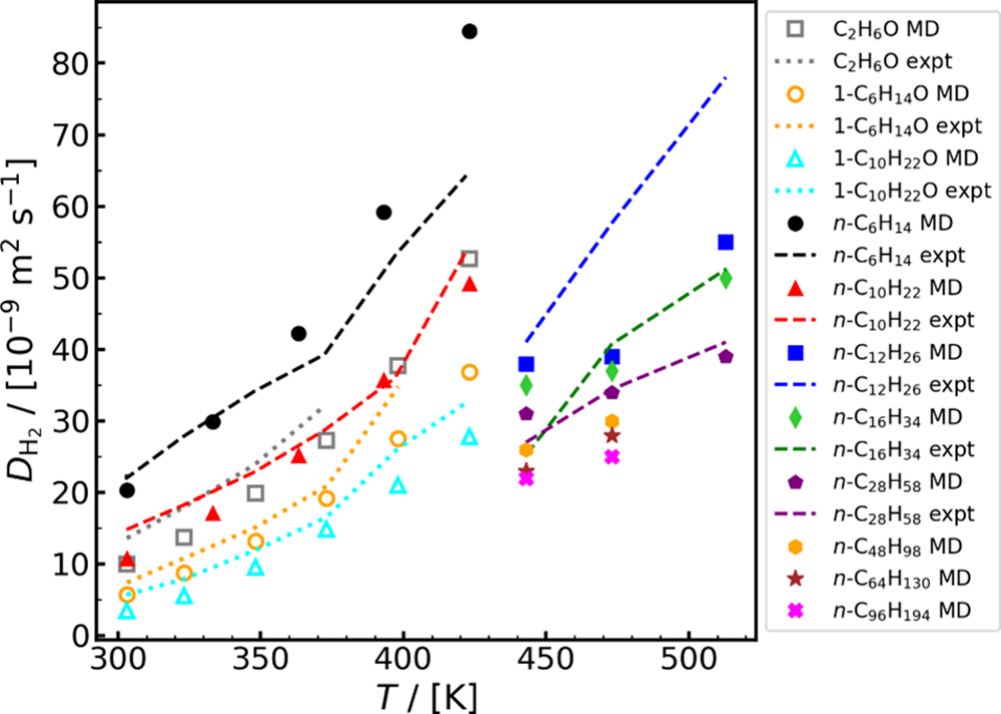
Diffusion coefficients of H_2_ in different *n*-alkanes and 1-alcohols. Data sources: *n*-C_6_H_14_ MD and experimental data (expt),^723^ *n*-C_10_H_22_ MD and expt,^723^ alcohols,^725^ MD data for *n*-C_12_H_26_ up to *n*-C_96_H_194_,^722^ expt for these systems.^726,727^ Details about the force fields are given in Section 4.2.4.

In a consecutive study by the same research group following the same experimental technique (DLS) and simulation approach (methods and force fields), Wu et al.^725^ reported H_2_ self-diffusivities in 1-alcohols (i.e., C_2_H_6_O, 1-C_6_H_14_O, and 1-C_10_H_22_O) in the temperature range 303 - 423 K. As in their previous study,^723^ the concentration of H_2_ was close to the infinite dilution limit. The computed diffusivities are shown in Fig. 39 along with the respective experimental data. The temperature and alkyl chain dependence of H_2_ diffusivity was the same as in the previous study, however, the value was lower compared to He diffusivity in the same solvents, despite the latter having a lower molar mass. The study showed that the fraction of hydrogen-bonded alcohol molecules was another factor affecting diffusivity. The fractions ranged from 0.99 at low temperatures to 0.68 at high temperatures for the different alcohol solvents, decreasing with increasing temperature and increasing alkyl chain length. The authors used the data to construct a semi-empirical model for fast estimations of the gas diffusivities in these alcohols. More MD and experimental data of transport and structural properties of H_2_-hydrocarbon systems have been reported by Fröba and co-workers.^728–730^

By observing the different datasets collectively shown in Fig. 39, one can conclude that despite the different force fields and simulation protocols used in the different studies, the computed diffusivities show consistent trends, mainly dictated by the molecular weight of the hydrocarbon. Fig. 39 also reveals a lack of MD data for low/near-ambient temperature regimes for long *n*-alkanes, which can possibly be explained by the increased computational effort required to equilibrate and sample properties from these systems.

#### 4.2.5 Mutual Diffusivities of Hydrogen Mixtures

Accurately computing the mutual diffusivities in H_2_ mixtures is crucial for various applications including UHS and synthetic fuel production. Mutual diffusivities govern the rate of molecular mixing and mass transport in gas mixtures such as H_2_/CO_2_, impacting H_2_ purity and storage efficiency in subsurface reservoirs. Additionally, H_2_ diffusivity in aqueous environments (e.g., brines) affects H_2_ losses due to dissolution and potential chemical reactions during UHS as discussed in Section 2.3.4. Since experimental data under relevant high-pressure and temperature conditions are not available or challenging to obtain, molecular simulations provide vital insights and reliable predictions of diffusion behavior, enabling better design and management of H_2_ storage systems and related processes. Here, we review articles that report mutual- and self-diffusivities of H_2_ binary mixtures with small gases using MD simulations. Contrary to self-diffusivities of aqueous systems, the data on mutual-diffusivities of H_2_ mixtures are limited, practically corresponding to two studies, which are listed in Table 12.

**Table 12:** List of the MD Simulation Studies Investigating the Mutual Diffusivities of H_2_-Gas Mixtures along with Information on the Force Fields and Thermodynamic Conditions

	System details		Thermodynamic conditions	
Study	Components	Force field	*p*/[MPa]	*T*/[K]
Hulikal Chakrapani et al.^677^	H_2_	Marx^328^	5–50	323.15–423.15
	CO_2_	TraPPE^678^		
Saric et al.^731^	H_2_	Vrabec^48^	9	290–350
	CO_2_	Merker et al.^732^		

Hulikal Chakrapani et al.^677^ computed mutual (i.e., MS and Fick) diffusivities of binary gas mixtures of H_2_ and CO_2_, as well as their solubilities in NaCl brine, over pressures ranging from 5 to 50 MPa, temperatures from 323.15 to 423.15 K, H_2_ mole fractions from 0 to 1, and salt concentrations up to 2 mol salt/kg of H_2_O. MD simulations were used to compute transport properties, i.e., self-, MS, and Fick diffusivities (methods discussed in Sections 3.2.3 and 3.2.4), while GEMC simulations with the CFC method were conducted to obtain VLE and solubilities. MS diffusivities from MD exhibit significant deviations from the Darken relation,^509^ which assumes ideal diffusing mixtures. For a H_2_ mole fraction of ca. 0.7, Darken underestimates MS diffusivities by up to 30%, whereas deviations drop to about 5% at low or high H_2_ concentrations. This underscores the non-ideal behavior of H_2_/CO_2_ mixtures, as also reflected in the thermodynamic factors, Γ, which quantify the non-ideality of mixtures (see Section 3.2.13). Γ decreases with increasing pressure, and increases with temperature, spanning roughly 0.25 to 1 across the studied conditions. The deviation of Γ from unity in the study by Hulikal Chakrapani et al.,^677^ confirms strong non-ideal interactions affecting the MS-Fick relationship. The order of magnitude of Fick diffusivities, which are obtained by multiplying MS diffusivities with Γ, range from approximately 10^–8^ to 10^–6^ m^2^/s, with higher values corresponding to lower pressures and elevated temperatures. At 5 MPa and 423.15 K, where mixtures approach ideal behavior, kinetic theory (using the Chapman-Enskog expression) predicts Fick diffusivities within 5% accuracy. However, at lower temperatures and higher pressures, especially in CO_2_-rich mixtures, kinetic theory deviates by up to 75%. In contrast, H_2_-rich mixtures retain agreement within about 15% even at 50 MPa, owing to their more ideal-gas-like behavior. To remedy kinetic theory’s shortcomings at high pressures and low temperatures, the empirical Moggridge correlation is applied. This captures the liquid-like behavior of CO_2_-rich mixtures and predicts MS, and thus Fick, diffusivities within 5 to 10% of computed values under such conditions. Yet, for H_2_-rich mixtures and low pressures, Moggridge’s correlation can err by up to 35%, making it less suitable in those regimes. The observed transport behavior stems from the transition between gas-like and dense-gas-like regimes, with self-diffusivities inversely proportional to mixture density. Molecular size differences also play a key role i.e., H_2_ diffuses 2.5 to 6 times faster than CO_2_, a discrepancy partially explained by the Stokes-Einstein relation. Comparisons with experimental data at near-atmospheric pressures (indirectly obtained by extrapolating the simulation data via the principle of corresponding states) show agreement within approximately 15%, thereby validating the molecular simulation approach. Although no single general fit function is proposed, established correlations (i.e., Chapman-Enskog kinetic theory, Fuller correlation, and Moggridge correlation) are assessed and recommended according to the relevant pressure, temperature, and composition regimes.

Saric et al.^731^ performed MD simulations and EoS modeling to examine thermodynamic, transport, and structural properties of supercritical carbon dioxide (scCO_2_) mixtures containing solutes such as H_2_, CH_4_, and various hydrocarbons, over temperatures from approximately 290 to 350 K, and pressures in the range 9 - 12 MPa. The study focused on dilute compositions near the infinite-dilution limit (0.3-1.5 mol%). Here, the Widom region refers to the temperature span near the critical point where the fluid undergoes a continuous crossover from liquid-like to gas-like supercritical behavior. It is marked by pronounced maxima in thermodynamic response functions such as *c_p_*, *β_T_*, and *α*, and corresponds to enhanced density fluctuations and structural changes. In this context, the temperature dependence of self-diffusion coefficients (also termed intra-diffusion coefficients) in H_2_/scCO_2_ mixtures was analyzed. These coefficients, representing species-specific Fickian self-diffusion, were derived from velocity autocorrelation functions in MD simulations, distinguishing them from MS diffusivities. Across the Widom region, self-diffusion coefficients exhibit a sigmoidal increase with temperature, rising by at least an order of magnitude between roughly 305 and 320 K at 9 MPa. Lighter molecules, such as H_2_, diffuse significantly faster than e.g., hydrocarbons, with diffusivities increasing more steeply during the liquid-like to gas-like supercritical crossover. This sigmoidal behavior reflects the transition from a dense, liquid-like regime, dominated by strong intermolecular interactions and low diffusivity, to a low-density, gas-like regime where kinetic effects prevail and diffusivity is high. The crossover temperature aligns with maxima in *c_p_*, *β_T_*, and *α*, and coincides with microscopic structural changes: a reduction in average coordination number and clustering effects quantified by Kirkwood-Buff integrals. Moreover, near the Widom line, the kinetic contribution to shear viscosity surpasses the configurational component, indicating a shift in molecular transport mechanisms. In the Widom region, simulation data exhibit increased scatter, reflecting the strong density fluctuations characteristic of critical phenomena. Finally, Saric et al.^731^ proposed an empirical correlation for predicting the Widom line temperature as a function of pressure, solute mole fraction, and the critical properties of mixture components. Although this correlation yields only the thermodynamic crossover temperature, it enables indirect estimation of mutual diffusivity trends by pinpointing the temperature region where transport properties such as diffusivity change dramatically due to the liquid-to-gas transition in the supercritical phase. Consequently, knowledge of the Widom line location aids in anticipating the temperature range over which molecular transport properties vary strongly.

### 4.3 Hydrogen Liquefaction and Properties of Liquid Hydrogen Systems

Han et al.^295^ investigated H_2_ liquefaction on CNT surfaces by performing MD simulations using the reactive force field ReaxFF^707^ to model H_2_-CNT interactions. Although in this review we do not systematically discuss reactive systems, we cover this study for two reasons: (i) to the best of our knowledge, molecular simulations of H_2_ liquefaction are extremely scarce, and (ii) the claim of the authors that the liquefaction of H_2_ could be increased by introducing CNTs is both unique and could lead to significant advancements in H_2_ transport and storage. The simulations compared H_2_ interactions with different CNT systems (bundles, isolated CNTs, single- and multi-walled). The authors report that H_2_ liquefaction occurred only on the CNT bundle (not on single CNTs) at 80 K and 100 bar. This phenomenon involves various mechanisms; deformation of the CNT bundle into oval shapes under pressure, charge transfer from low-curvature to high-curvature regions of the deformed CNTs, development of charge polarization in the bundle, long-range electrostatic interactions between the polarized charges and H_2_ molecules, and high local-ordering of H_2_ gas leading to liquefaction. Single CNTs did not induce liquefaction because they deformed symmetrically under pressure, preventing charge polarization from developing. No chemisorption of H_2_ was observed, only physisorption and liquefaction. Han et al.^295^ suggest that H_2_ gas could potentially be liquefied at temperatures higher than 80 K on more strongly polarized CNT bundles. This study provides insight into the previously unexplained linear increase in H_2_ uptake with pressure observed by Ye et al.^733^ and explains the discrepancy between the high storage capacity of CNT bundles versus the limited capacity of single CNTs.

Cheng and Frenkel^537^ introduced the WAVE method and applied it to calculate the thermal conductivity of liquid H_2_ and supercritical water (see the details of the method in Section 3.2.6). The H_2_ system studied was highly compressed (33 GPa) at 2000 K. The authors used an ML-derived force field, for which they obtained a thermal conductivity of 5 W/mK. This value is on the same order of magnitude with the value for compressed gaseous H_2_ at 1000 bar, obtained from the extrapolation of measurements. The authors note that nuclear quantum effects should probably be considered for more accurate results. For more details on these effects the reader is referred elsewhere.^734,735^

A series of studies, such as the ones by Lenosky et al.,^736^ Collins et al.,^737^ Holst et al.,^738^ Tian et al.,^739^ Redmer et al.^740^ focused on computing properties of liquid H_2_ mainly using quantum mechanical methods (e.g., ab initio MD, tight-binding electronic structure techniques, DFT). These studies report electrical conductivities, *pVT* relations, hopping mechanisms and electronic transport coefficients, atomic/molecular H_2_ properties, liquid-liquid phase transitions, shock-compressed H_2_. Despite the wealth of physical insights and the useful data provided, a detailed analysis of these papers is not in the scope of this review, which focuses on classical molecular simulation of H_2_ systems.

Liu et al.^741^ performed MD simulations to study liquid H_2_ nucleation and boiling on a flat aluminum heat conducting substrate. The authors used a simple LJ potential for H_2_ molecules and an EAM potential for the wall. Bubble nucleation was studied in the temperature range of 100 - 200 K, while liquid H_2_ was obtained at 20 K. The authors mention that their MD simulations revealed a complete bubble nucleation, growth, and merging process, while for the H_2_ boiling process, nucleate, transition, and film boiling stages were obtained. Higher wall temperatures accelerated bubble growth rate and affected gas film growth rate more than bubble growth rate. Wall temperature was shown to have minimal effect on liquid layer thickness during nucleation. The contact angle computed was ca. 4.9 degrees. While the study by Liu et al.^741^ provides some insight into interfacial H_2_ properties, the methods used could raise concerns regarding the accuracy of the reported data and conclusions. The LJ potential used for H_2_-H_2_ interactions may be adequate for gaseous systems, however, since H_2_ is a quantum fluid at low temperatures (such as the cryogenic temperature of 20 K used in the study), is highly unlikely to be accurate (techniques such as path-integral MD would be more appropriate). The authors mentioned that the potential validation was performed for density at 20 K and 1 bar, for which the result deviated only 1.37% from the NIST data (ca. 70 kg m^–3^). While this seems reasonable, density alone is insufficient to validate a model for complex phenomena such as boiling. The authors neither mention the use of quantum corrections nor of a more sophisticated force field which accounts for quantum effects. In addition, the cutoff distance for the interactions is not mentioned. The NEMD simulation performed to study the bubble nucleation was only 26 fs, which is extremely short for observing complete boiling phenomena.

### 4.4 Hydrogen Solubility, Diffusivity, and Permeability in Polymeric and Composite Materials

The role of molecular simulations the past 40 years in understanding gas sorption and permeation in polymers has been significant. Unlike inorganic materials, polymers experience structural alterations during these processes. Many studies successfully modeled barrier properties in various polymers (e.g., PET, PS, polyimides) and accurately predicted sorption mechanisms, temperature effects, and permeation values. Key qualitative findings include the sorption mechanisms below glass transition temperatures, accessible volume pockets influenced by polymer chemistry, and structural changes during sorption. Research also has been conducted to study mixed-gas penetrants and composite materials (such as MOF-polymers, zeolite-polymers), demonstrating that molecular simulations can reproduce experimental trends, and sometimes, even quantitative predictions of permeation properties.^742,743^

As discussed in Sections 2.2.1, 2.3.1 and 2.4.1, important H_2_ technologies, such as storage in tanks, membrane separations, and gas transport via pipelines, may involve polymeric materials. To this purpose, MD and MC studies focusing on H_2_ solubilities, diffusivities, and permeabilities in these materials are abundant in the literature. The study by Voyiatzis and Stroeks^407^ presents a comprehensive investigation into the solubilities of H_2_ and O_2_ in two industrially significant polymers (e.g., as liner materials in high-pressure H_2_ storage tanks): PA6 and HDPE. The authors used the free energy perturbation and fast-growth thermodynamic insertion methods, to predict H_2_ solubilities in both the crystalline and amorphous phases of the polymers. These simulations were conducted in a temperature range of 300 - 360 K and at 700 bar pressure to study H_2_ transport behavior under conditions relevant to storage in tanks. The crystalline phases of the PA6 and HDPE polymers consisted of 64 and 40 chains of infinite length, respectively. The crystal structure of PA6 in the simulation box is shown in Fig. 42(a). The amorphous phases comprised 10 PA6 molecules of 160 monomers, and 30 HDPE chains of 110 monomers. The study revealed that the crystalline phases of both PA6 and HDPE are completely impermeable barriers to both H_2_ and O_2_, despite the small size of these gases. The molecular simulations achieved semi-quantitative agreement with experimental solubility measurements. Discrepancies between predicted and experimental values were attributed primarily to the simulations’ inability to fully account for the constraining effects that crystalline regions exert on adjacent amorphous polymer domains. The study also showed that despite H_2_ being significantly smaller than O_2_, it consistently exhibits lower solubility in both PA6 and HDPE. This is a rather counterintuitive finding, also shown in prior simulation and experimental studies, that can be explained based on the more favorable interactions of the O_2_ with both polymer matrices compared to H_2_. The findings by Voyiatzis and Stroeks^407^ that H_2_ migration occurs predominantly through the amorphous regions, with crystalline domains acting as impermeable obstacles, and that intermolecular interactions between the gases and the polymer chains dominate solubility behavior over considerations of accessible volume distribution within the polymer, can be taken into consideration when designing gas barrier materials. The computed solubilities are shown in Fig. 40 and listed in Table 13.

**Figure 40:**
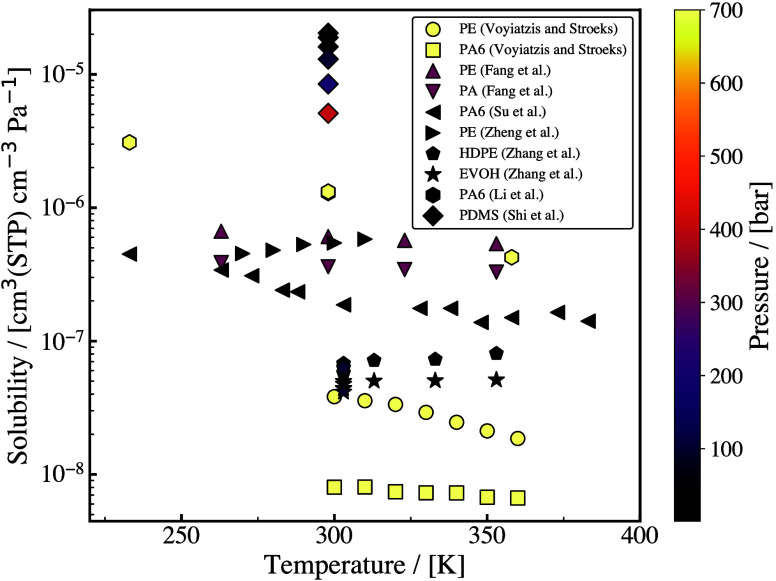
Solubilities of H_2_ in different polymers computed via molecular simulations performed in different studies. The raw data and references are listed in Table 13 except for the solubility of H_2_ in PDMS.^749^

**Table 13:** Solubilities (*S*) of H_2_ in PE/HDPE, PA6/PA, and EVOH Polymeric Matrices Computed in Different Studies^a^

Source	*T*	*p*	PE/HDPE	PA6/PA	EVOH
Voyiatzis and Stroeks^407^	300	700	3.83 × 10^–8^	8.01 × 10^–9^	
	310	700	3.57 × 10^–8^	8.05 × 10^–9^	
	320	700	3.35 × 10^–8^	7.42 × 10^–9^	
	330	700	2.92 × 10^–8^	7.28 × 10^–9^	
	340	700	2.46 × 10^–8^	7.27 × 10^–9^	
	350	700	2.12 × 10^–8^	6.74 × 10^–9^	
	360	700	1.86 × 10^–8^	6.66 × 10^–9^	
Fang and Ji^218^	263	300	6.66 × 10^–7^	3.87 × 10^–7^	
	298	300	6.07 × 10^–7^	3.61 × 10^–7^	
	323	300	5.70 × 10^–7^	3.44 × 10^–7^	
	353	300	5.37 × 10^–7^	3.29 × 10^–7^	
Su et al.^746^	233	1		4.49 × 10^–7^	
	263	1		3.41 × 10^–7^	
	273	1		3.09 × 10^–7^	
	283	1		2.41 × 10^–7^	
	288	1		2.34 × 10^–7^	
	303	1		1.87 × 10^–7^	
	328	1		1.76 × 10^–7^	
	338	1		1.76 × 10^–7^	
	348	1		1.38 × 10^–7^	
	358	1		1.50 × 10^–7^	
	373	1		1.64 × 10^–7^	
	383	1		1.41 × 10^–7^	
Li et al.^745^	298	1		1.32 × 10^–6^	
	298	350		1.31 × 10^–6^	
	298	525		1.29 × 10^–6^	
	298	700		1.32 × 10^–6^	
	233	700		3.09 × 10^–6^	
	358	700		4.25 × 10^–7^	
Zheng et al.^748^	270	1	4.53 × 10^–7^		
	280	1	4.80 × 10^–7^		
	290	1	5.30 × 10^–7^		
	300	1	5.44 × 10^–7^		
	310	1	5.81 × 10^–7^		
Zhang et al.^750^	303	25	6.79 × 10^–8^		4.40 × 10^–8^
	313	25	7.16 × 10^–8^		5.02 × 10^–8^
	333	25	7.30 × 10^–8^		5.05 × 10^–8^
	353	25	8.07 × 10^–8^		5.12 × 10^–8^
	303	25	6.79 × 10^–8^		4.43 × 10^–8^
	303	40	6.36 × 10^–8^		4.755 × 10^–8^
	303	60	5.78 × 10^–8^		4.995 × 10^–8^
	303	100	6.29 × 10^–8^		4.148 × 10^–8^

a *T* is in units of K, *p* in bar, and *S* in cm^3^(STP) cm^–3^ Pa^–1^.

Zhao et al.^744^ performed all-atom MD simulations to study the mass of H_2_ in PE, a liner material used in type IV storage tanks (see also Section 2.3.1). The study was performed at a wide pressure (up to 700 bar) and temperature (up to 400 K) range, and focused on different properties including tensile behavior, glass transition temperature, H_2_ diffusion in various PE structures, and bubble formation during rapid depressurization. Several systems were considered, i.e., varied PE chain length and structure (branched and unbranched), reinforcement with graphene, the incorporation of small molecules as additives (C_20_H_42_), and various H_2_ concentrations. The number of polymer chains in the systems studied varied from 1 to 16, having degrees of polymerization in the range of 500 to 2000. The authors showed that the presence of H_2_ in PE matrices deteriorated tensile performance and decreased the glass transition temperature, compromising the structural integrity of the materials. Several structural factors affected H_2_ diffusion in amorphous PE regions. In short, branches and side chains increased H_2_ diffusivity by introducing more free volume below glass transition temperature, the chain length variation had minimal effect if chains were sufficiently long, polymer orientation improves barrier properties when aligned against the pressure drop direction, small molecules/additives (acting as plasticizers) increased diffusion rates, while graphene reinforcement has the opposite effect. During rapid depressurization, H_2_ molecules aggregated into nano-sized bubbles within the polymer matrix. These bubbles formed in both exclusive and free volume regions, appearing and disappearing during pressure changes before stabilizing at low pressures. To study these bubbling effects, the authors introduced to the systems large number of H_2_ molecules. A simulation snapshot showing a H_2_ bubble comprising 1000 gas molecules in the polymer is shown in Fig. 42(b). Similarly, for computing diffusivity, 100 H_2_ molecules were used. It is important to comment here that such system sizes, which lead to phase separation (gas and polymer), make the control of the pressure of the system an arduous task for the barostat. Additionally, the introduction of rapid changes in pressures in MD simulations, bring the systems in a non-equilibrium state, during which equilibrium properties cannot be accurately sampled. Based on their findings, Zhao et al.^744^ indicate that HDPE without branches and without small molecules as additives could deliver superior performance as a H_2_ barrier, while graphene reinforcement should be oriented against the pressure drop direction. The computed solubilities are shown in Fig. 40 and listed in Table 13.

The permeation behavior of H_2_ in PE and PA was also investigated by Fang and Ji^218^. Since the motivation of this study was also the high-pressure H_2_ storage in IV-type tanks, the simulations were performed at representative for the application conditions, i.e., high pressure (300 bar) and at temperatures ranging from ca. 260 to 350 K. Contrary to Voyiatzis and Stroeks,^407^ only amorphous phases were considered in this study. The authors state that the molecular weight of the PE cell was 3630, while the respective for PA was 3440. The respective polymerization degrees were 40 and 18. This study focused on several properties affecting H_2_ permeation: FFV, and solubility, diffusion, and permeability coefficients. Qualitatively consistent with prior experiments and molecular simulation studies, Fang and Ji^218^ showed that increasing temperature causes a decrease in solubilities and increase in diffusivities of H_2_ in both polymers. FFV correlated positively with temperature, with PE showcasing an increase of FFV of ca. 50% (in the range 263-353 K) compared to ca. 33% of PA. A schematic representation of the FFV distribution in the PA matrix at 353 K is shown in Fig. 42(c). Permeability coefficients increased with increasing temperature, showing the dominant role of the diffusivity in the computation (see also Section 2.2.1). PE consistently exhibited higher solubilities and diffusivities than PA for all conditions considered. As a consequence, PA is stated to have superior H_2_ permeability resistance, performing three to four times better than polyethylene in preventing H_2_ permeation. The computed solubilities and permeabilities are shown in Fig. 40 and Fig. 41, and listed in Table 13 and Table 14, respectively. Despite simulations following the general trends of experimental data, quantitatively the simulation data overshoot the experiments by 1 to 2 orders of magnitude, clearly showing that either the simulation scheme and/or the force fields chosen have limited predictive ability. In a similar study, Li et al.^745^ studied the barrier performance (via computing solubilities, diffusivities, and permeabilities) of graphene/PA6 composites, having the filler (graphene) composition as a parameter ranging from 3 to 7 wt%. Depending on the graphene content, the number of PA6 monomers varied from 8 to 60. The exact number of polymer chains used is not clearly mentioned, however, the unit cell dimensions for the different systems are provided. A configuration of a model graphene/PA6 system as obtained by the commercial software used by the authors is shown in Fig. 42(d). Similarly to the previous study, conditions relevant to H_2_ storage at IV-type tanks were considered, i.e., 233 - 358 K and 1 - 700 bar. In this study, the properties were correlated to different graphene contents except from the different temperatures and pressures. Several insights, relevant to H_2_ storage vessel design, were reported. Particularly, it was shown that the 5 wt% graphene/PA6 composite has the optimal barrier performance, achieving a 54.6% reduction in permeability coefficient compared to pure PA6 at ambient conditions. This substantial improvement indicates the effectiveness of graphene as a nanofiller for H_2_ barrier applications. Li et al.^745^ showed that temperature has a pronounced effect on H_2_ diffusion, with the diffusion coefficient of 5 wt% graphene/PA6 increasing by 138% in the range 298 - 358 K at 70 MPa. The authors state that this finding highlights the importance of thermal management in storage vessel design. As in prior studies, H_2_ diffusivity was shown to have a linear and inverse relationship with pressure for all materials. Pure PA6 consistently showed the highest diffusion coefficient, while the 4 wt% graphene/PA6 composite displayed the lowest. The addition of graphene was found to restrict polymer chain movement, and disrupt pore continuity within the polymer matrix, creating a more tortuous path for H_2_ molecules. FFV directly correlated with diffusion coefficients, providing a mechanistic explanation for the observed barrier improvements. The computed solubilities and permeabilities are shown in Fig. 40 and Fig. 41, and listed in Table 13 and Table 14, respectively.

**Figure 41:**
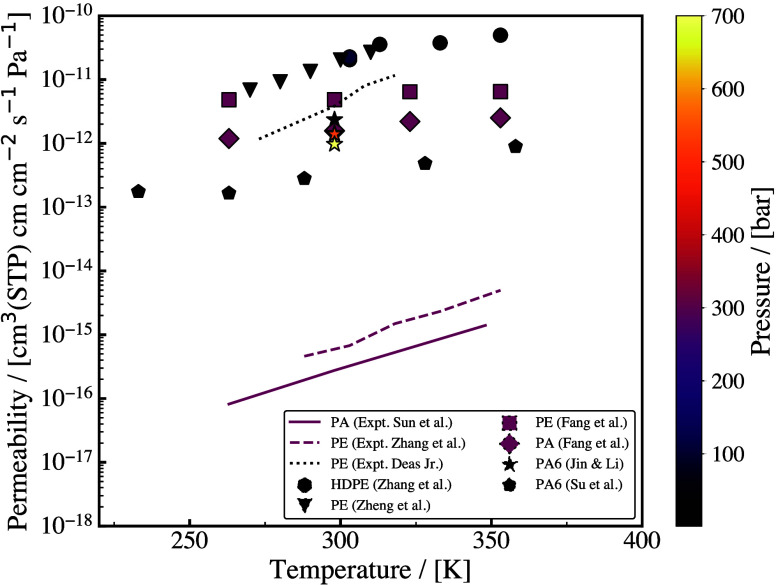
Permeabilities of H_2_ in different polymers computed via molecular simulations performed in different studies. Three experimental datasets are shown for comparison (lines). The raw data and references are listed in Table 14.

**Table 14:** Permeabilities (*P*) of H_2_ in PE/HDPE and PA6/PA Computed in Different Studies^a^

Source	*T*	*p*	PA/PA6	PE/HDPE
Fang and Ji^218^	263	300	1.19 × 10^–12^	4.82 × 10^–12^
	298	300	1.57 × 10^–12^	4.84 × 10^–12^
	323	300	2.20 × 10^–12^	6.43 × 10^–12^
	353	300	2.51 × 10^–12^	6.46 × 10^–12^
Li et al.^745^	298	1	2.35 × 10^–12^	
	298	350	1.44 × 10^–12^	
	298	525	1.39 × 10^–12^	
	298	700	9.70 × 10^–13^	
Su et al.^746^	233	1	1.75 × 10^–13^	
	263	1	1.65 × 10^–13^	
	288	1	2.81 × 10^–13^	
	328	1	4.83 × 10^–13^	
	358	1	8.91 × 10^–13^	
Zheng et al.^748^	270	1		6.84 × 10^–12^
	280	1		9.24 × 10^–12^
	290	1		1.34 × 10^–11^
	300	1		2.03 × 10^–11^
	310	1		2.67 × 10^–11^
Zhang et al.^750^	303	25		2.16 × 10^–11^
	313	25		3.56 × 10^–11^
	333	25		3.76 × 10^–11^
	353	25		4.96 × 10^–11^
	303	40		2.26 × 10^–11^
	303	60		2.05 × 10^–11^
	303	100		2.24 × 10^–11^
Experimental data	273	1		1.17 × 10^–12^
by Deas-Jr et al.^751^	298	1		3.84 × 10^–12^
	308	1		8.04 × 10^–12^
	318	1		1.16 × 10^–11^
Experimental data	288	300		4.60 × 10^–16^
by Na Zhang et al.^752^	303	300		6.75 × 10^–16^
	318	300		1.49 × 10^–15^
	333	300		2.32 × 10^–15^
	353	300		4.97 × 10^–15^
Experimental data	263	300	8.18 × 10^–17^	
by Sun et al.^753^	298	300	2.76 × 10^–16^	
	348	300	1.40 × 10^–15^	

a Three experimental datasets are provided for comparison. *T* is in units of K, *p* in bar, and *P* in cm^3^(STP) cm cm^–2^ s^–1^ Pa^–1^.

**Figure 42:**
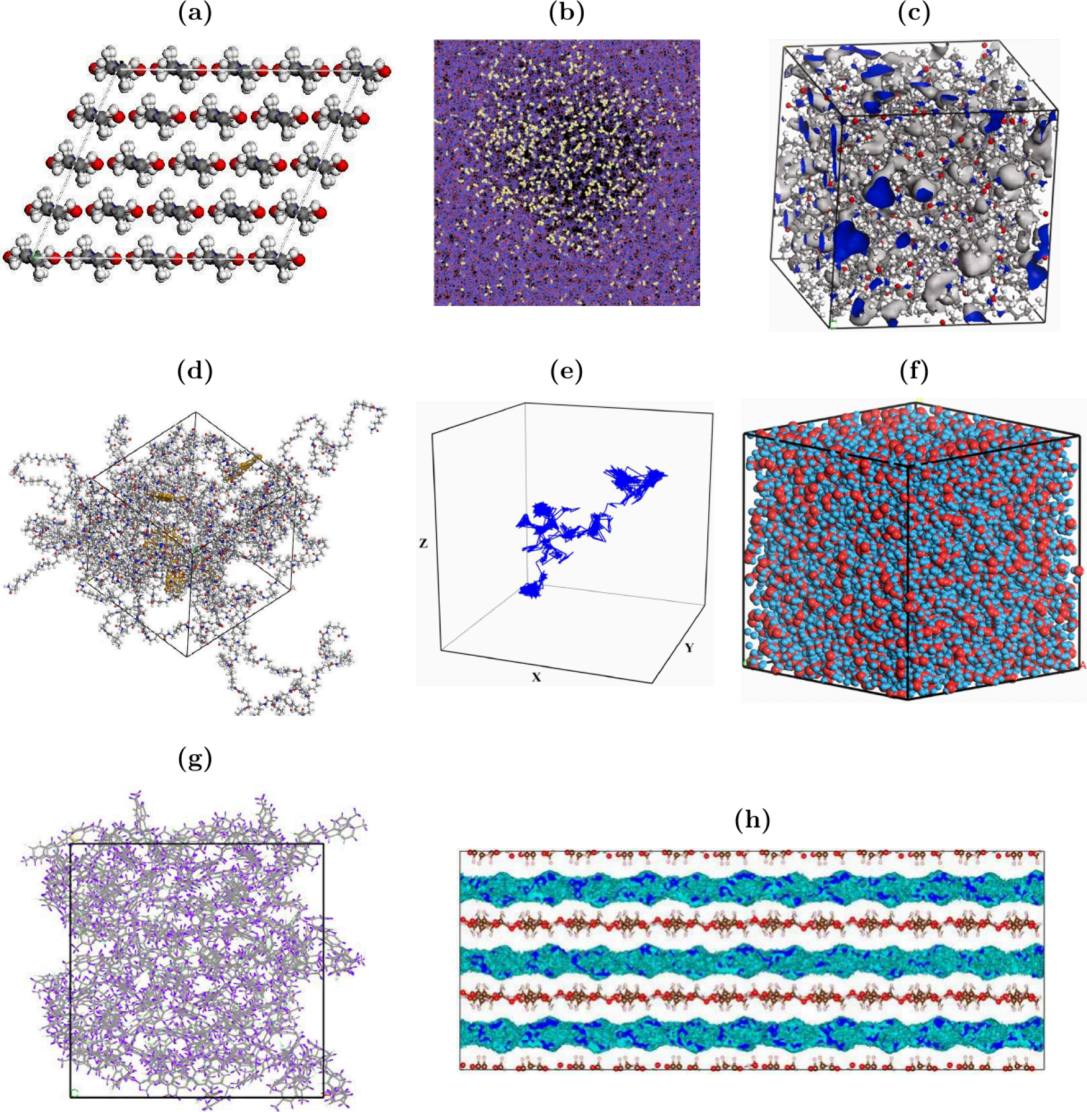
Simulation snapshots and molecular representations collected from different studies of H_2_/polymer systems: (a) PA6 crystal structure shown in the x-y plane, (b) H_2_ gas bubble in PE. H_2_ molecules are shown in yellow; PE is shown in blue (C atoms) and the (H atoms), (c) free volume distribution in PA at 353 K, (d) a model graphene-modified PA6 system. Graphene atoms are shown in yellow, (e) the trajectory of a H_2_ molecule in amorphous PA6 328 K and 0.1 MPa, (f) amorphous PE matrix. Carbon atoms are shown in blue and hydrogen atoms in red, (g) amorphous poly­(chloro-p-xylylene) matrix, (h) H_2_ sorption sites in a cellulose slit pore. [Panel (a) is reprinted with permission from Voyiatzis and Stroeks, Phys. Chem. B 2022, 126, 32, 6102-6111. Copyright 2022, American Chemical Society. Panel (b) is reprinted from International Journal of Hydrogen Energy, Vol 47, Jiawei Zhao et al., Molecular dynamics simulation of H_2_ in amorphous polyethylene system: H_2_ diffusion in various PE matrices and bubbling during rapid depressurization, 39572–39585, Copyright (2022), with permission from Elsevier. Panel (c) is reprinted from Materials Today Communications, Vol 35, Qing Fang, Dongmei Ji, Molecular simulation of hydrogen permeation behavior in liner polymer materials of Type IV hydrogen storage vessels, 106302, Copyright (2023), with permission from Elsevier. Panel (d) is reprinted from Li et al.,^745^ open access article distributed under the terms and conditions of the Creative Commons Attribution (CC BY 4.0) license. Panel (e) is reprinted from International Journal of Hydrogen Energy, Vol 50, Part D, Ying Su et al., Hydrogen permeability of polyamide 6 as the liner material of Type IV hydrogen storage tanks: A molecular dynamics investigation, 1598-1606, Copyright (2024), with permission from Elsevier. Panel (f) is reprinted from Journal of Molecular Liquids, Vol 368, Part B, Dukui Zheng et al., Molecular dynamics investigations into the hydrogen permeation mechanism of polyethylene pipeline material, 120773, Copyright (2022), with permission from Elsevier. Panel (g) is reprinted from Computational Materials Science, Vol 49, Chunhai Lu et al., A molecular modeling study on small molecule gas transportation in poly (chloro-p-xylylene), S65-S69, Copyright (2010), with permission from Elsevier. Panel (f) is from Molecular simulation of hydrogen storage and transport in cellulose Molecular simulation of hydrogen storage and transport in cellulose, Stalker et al., Molecular Simulation, Vol 47, 170–179, Copyright (2010), reprinted by permission of the publisher (Taylor & Francis Ltd, http://www.tandfonline.com).]

**Table 15:** Experimentally Measured H_2_/CH_4_ Permeabilities Gathered from Different Literature Sources^a^

Membrane	*P*_H_2__ (Barrer)	*P*_H_2__/*P*_CH_4__
ZIF-8 ^812^	1.38 × 10^4^	20.3
ZIF-8 ^813^	1.25 × 10^4^	16.2
ZIF-22 ^814^	2.27 × 10^4^	5.2
LTA^815^	3.76 × 10^3^	3.6
HKUST-1 ^816^	1.79 × 10^5^	6
ZIF-7 ^817^	358	5.9
P84/ND^818^	6.7	310
Matrimid/ZIF-11 (10%)^819^	28.11	97
6FDA-TTM/Si-H (5%)^820^	62.6	160.5
MWCNT@GONRs (2%)^821^	42.5	18.5
PLA^822^	25	10
Hyflon AD60X^823^	187	62
ZIF-8/6FDA-durene^824^	4.2 × 10^3^	17
Matrimid-ZIF-8 ^825^	64	137
MMM-ZIF-8 ^826^	27.1	123.2
MMM- UiO-66 ^826^	64.4	153.3
JUC-160 ^827^	2 × 10^3^	8.5
MEFMEQ mixture^828^	3.7 × 10^3^	7
MEFMEQ single components^828^	4.8 × 10^3^	21
ZIF-8 mixture^828^	6 × 10^3^	9.8
ZIF-8 single components^828^	5.3 × 10^3^	13.4
MOF-5 mixture^828^	4.1 × 10^3^	2
MOF-5 single components^828^	4.6 × 10^3^	2.3
Cu-BTC mixture^828^	2.2 × 10^3^	3.2
Cu-BTC single components^828^	2.4 × 10^3^	4
ZIF-7 ^829^	15.4	35.8
ZIF-62 glass^830^	4.2 × 10^3^	50
NH2 – MIL–53 ^831^	1.3 × 10^5^	27.3
ZIF-90 ^832^	1.7 × 10^5^	70.5
Ni_2_(L-asp)_2_(bipy)^833^	6 × 10^4^	7.77
ZIF-7 ^829^	14.4	34.6
MOF-5 ^834^	1.89 × 10^5^	64
SIM-1 ^835^	6.1 × 10^3^	2.52
Sod-ZMOF-1 ^836^	36.5	1.4
PSM-MOF^837^	1.18 × 10^3^	11.1
Pebax ZIF-67 NP^838^	10.7	1.444
Pebax ZIF-67 MP^838^	10.8	1.367
Pebax Zif-67 NS^838^	12.1	1.528
Pebax 1675/CuNi^839^	11.5	3.026
PEEK-WC/CuNi^839^	68.01	68.01
6FDA-BI^840^	33.4	278.2
P/Zn^813,840^	31.8	278
6FDA-BI/20%ZIF-8 ^840^	78.5	223.9
6FDA-BI/20%ZIF-8 0.004 Zn^813,840^	88.2	233.4
6FDA-BI/20%ZIF-8 0.007 Zn^813,840^	110.1	225.2
6FDA-BI/20%ZIF-8 0.01 Zn^813,840^	72.3	318.3
ZIF-67-in-TpPA-1 ^841^	3.67 × 10^3^	34.9
ZIF-8-in-TpPA-1 ^841^	3.92 × 10^3^	23.1
ZIF-67-in-TpBD^841^	3.77 × 10^3^	27.9

a The data are used in Fig. 5(a) and Fig. 48(a).

**Table 16:** Experimentally Measured H_2_/CO_2_ Permeabilities Gathered from Different Literature Sources^a^

Membrane	*P*_H_2__ (Barrer)	*P*_H_2__/*P*_CO_2__
UiO-66-NH2 ^842^	213.2	6.8
PEI/PVP-UiO-66-NH2 ^843^	31	0.0787
Matrimid/EDA/ZIF-90 ^844^	19	9.5
PPO/Silica^845^	257.23	3.6
PI/NH2-MIL-53 ^846^	384.1	16.7
PI/ZIF-8/EDA^847^	283.5	12
PIL/IL/MIL-53 ^848^	0.14778	0.07519
PIL/IL/ZIF-8 ^848^	0.0514	0.2
PEBAX-0H^849^	0.04879	0.13769
PEBAX-2H^849^	0.06889	0.11563
PEBAX-4H^849^	0.07507	0.11055
PEBAX-8H^849^	0.08876	0.10430
PEBAX-12H^849^	0.0808	0.10892
PEBAX-24H^849^	0.07652	0.10732
Glass ZIF-62 ^830^	4597	2.25
P84/ND^818^	6.7	4.1
Matrimid/ZIF-1 (10%)^819^	28.11	4
6FDA-TTM/Si-H (5%)^820^	62.6	2.1
PI/MWCNT@GONRs (2%)^821^	42.5	1.7
6FDA-DAM-ZIF-11 (10%)^819^	106.7	0.97
6FDA-DAM-ZIF-11 (20%)^819^	272.45	1.06
6FDA-DAM-ZIF-11 (30%)^819^	76.76	1.05
P100G0 ^850^	0.07465	0.10417
P100G2 ^850^	0.08241	0.11236
P100G5 ^850^	0.14525	0.12821
P100G8 ^850^	0.18277	0.14286
ZIF-8 ^851^	5411	4.5
CL 6FDA-durene^824^	116.7	24.3
33 MMM^824^	2137	1.4
50 MMM^824^	4157	1.3
CL 33 MMM^824^	394.5	28.6
CL 50 MMM^824^	501	28.8
CL skin alone on 33 MMM^824^	12.5	34.6
CL skin alone on 50 MMM^824^	13.9	32.4
CL skin alone on 6FDA-durene^824^	11.1	31.7
ZIF-67-in-TpPA-1 ^841^	3618.75	35.71
ZIF-8-in-TpPA-1 ^841^	3772.04	28.73
ZIF-67-in-TpBD-1 ^841^	3490.05	23.85
HKUST-1 ^816^	176844	6.8
ZIF-7 ^852^	54.2	8.4
ZIF-7 ^814^	340.8	6.48
ZIF-22 ^853^	19572	7.2
ZIF-8 ^854^	3536.4	6
NH2-MIL-53 (Al)^855^	87750	30.9
ZIF-8 ^856^	2534.8	3.28
Amine-Mg-MOF-74 ^857^	2240	28
MIL-96 (Al)^858^	12260.8	8.8
Zn_2_(Bim)3 ^859^	19.187	128.4
PIM-EA-TB^860^	7620.1	1.09
PIM-SBI-TB^860^	21697.4	0.76
mPBO^861^	178.5	6.2
PHBOA (8:2)^862^	0	8
PBI^863,864^	12.8	20
PBI^865^	3.6	8.6
PBI^866^	2.9	7.1
PBI^867^	27	16
BILP-101x (400 nm)^868^	9.68	39.5
NUS-2@PBI^869^	2.95	18.78
[COF-300]- [Zn2(bdc)2(dabco)][Bibr ref870]	130368	12.6
[COF-300]- [ZIF-8][Bibr ref870]	105520	13.5
COF-300 ^870^	107520	6
COF-LZU1 ^871^	1842.1	6
ACOF-1 ^871^	1211.34	14.1
COF-LZU1-ACOF-1 ^871^	660.2	24.2
CTF-1 ^872^	501	17.4
TpPa-1-30/GO-10 ^873^	943.47	25.57
[COF-300]- [UiO-66][Bibr ref874]	117310	17.2
COF-LZU1 ^875^	7309.6	31.6
TFB-BD^875^	7604.4	25.6

a The data are used in Fig. 5(b) and Fig. 48(b).

H_2_ permeability in PA6 was also studied via MD and MC simulations performed by Su et al.^746^ Similarly to the studies discussed earlier, the authors highlight that H_2_ permeation through these polymeric liners is almost inevitable, thus, understanding the behavior of H_2_/PA systems under extreme conditions (233-358 K and 1-875 bar) is critical for evaluating long-term performance and safety of storage tanks. The authors claim that their work is the first comprehensive molecular-level investigation of H_2_ permeation in PA6 under realistic service conditions. Given that Su et al.^746^ performed simulations up to 875 bar, and that correction factors are applied to the results, their claim may be partially justified. However, it is important to comment here that this study uses conventional methods and tools to compute properties that have also been presented earlier for both PA and other polymeric matrices. In more detail, the authors primarily perform GCMC simulations to compute H_2_ solubilities, MD simulations to calculate diffusion coefficients, while they apply corrections to account for polymer crystallinity (30%). The corrections used for the solubilities and diffusivites are functions of the volume fractions of the amorphous and crystalline regions, which have been used also in earlier studies.^413,747^ In this study, 4 polymer chains comprising 200 monomers, were used. Su et al.^746^ discuss the dependence of solubitlies, FFVs, and diffusivities on temperature, which exhibit complex patterns with multiple Arrhenius regions, particularly at ca. PA6’s glass transition temperature. The computed solubilities and permeabilities are shown in Fig. 40 and Fig. 41, and listed in Table 13 and Table 14, respectively. The study identified a ”hopping” mechanism for H_2_ diffusion in PA6, according to which H_2_ molecules vibrate within the free volume pores for extended periods with a sudden hop happens to adjacent pores when the thermal motion of the polymer chains creates temporary channels. The simulation trajectory of a H_2_ molecule in PA6, indicating the hopping transport mechanism, is shown in Fig. 42(e). Please note that this H_2_ hopping mechanism should not be confused with the proton hopping mechanism discussed in e.g., Sections 3.2.12, and 4.1.6. As a conclusive remark, this study showcases that molecular simulations play a crucial role for understanding complex H_2_ phenomena at elevated conditions. For example, the identification of multiple Arrhenius regions in the behavior of sorption and diffusivity can lead to improved predictive models for permeation behavior.

Motivated by the use of polymers as building or supporting materials in pipelines, Memari et al.^413^ performed experiments and molecular simulations to study the solubility of CH_4_+CO_2_ and CH_4_+H_2_ mixtures in semicrystalline PE below its melting temperature. MC simulations in the osmotic ensmeble were performed using PE chains of 70 carbon atoms each. To the best of our knowledge, this is the only H_2_/PE study which uses an anisotropic potential to model the hydrocarbon (AUA4 ^412^). Interestingly, the force field used for H_2_ was developed by Darkrim et al.^327^ to study the physisorption of H_2_ and N_2_ on graphite basal planes. Memari et al.^413^ performed the MC simulations at 313 K and two pressures (6 and 21 bar), while a high-pressure (700±100 bar) was also examined to mimic the effect of crystallinity of the permeable zones due to the increase in the density of the polymer matrix as stated by the authors. The simulation results for the concentration of H_2_ in PE were shown to be a factor of 2 higher than the respective experimental data. The agreement with experiments for both gas mixtures is significantly better at the high-pressure, while the solubility selectivity *S*_CH_4_/SH_2__=54 is in agreement with predictive correlations.

Zheng et al.,^748^ also motivated by the application of PE as pipeline material, conducted MD and GCMC simulations to predict solubilities, diffusivities, and permeabilities of H_2_ in this polymer. The temperatures and pressures considered were in the range 270 - 310 K and 1 - 70 bar, respectively, in an effort to match common operating conditions for urban PE pipelines. The authors used five PE chains each one with a degree of polymerization of 500 (corresponding to C_1000_H_2002_) to build an amorphous polymer phase. A molecular representation of the PE in the simulation box is shown in Fig. 42(f). In line with the similar studies reviewed earlier, solubilities, diffusivities, and permeabilities increased with temperature, while pressure was shown to have a negligible effect. The computed solubilities and permeabilities are shown in Fig. 40 and Fig. 41, and listed in Table 13 and Table 14, respectively. As in the study by Su et al.,^746^ the gas is shown to obey the hopping transport mechanism. The free volume increases with temperature, therefore, enhancing H_2_ diffusivity. The computed permeabilities were shown to systematically over-predict the experimental data by 5 to 6 times in the whole temperature range considered, clearly indicating that there is room for improved predictions using molecular simulations. This can be achieved by either using more accurate combination of force fields and/or by fine-tuning the polymer chain characteristics (e.g., molecular weight, number of chains, degree of crystallinity).

Recently, H_2_ permeation through HDPE and EVOH was studied by Zhang et al.^750^ at conditions relevant to gas transport in polymer pipelines, i.e., 30 °C to 80 °C (303.15 - 353.15 K) and 25 - 100 bar. The authors constructed systems containing 12 HDPE (or EVOH) chains but the degree of polymerization or the molecular weight of the polymer chains is not mentioned. The representativeness of the model was validated by comparing the density of the simulation box containing the polymer matrix with experimental values. Similarly to all the previous studies discussed in this section, temperature was shown to have a strong effect on H_2_ properties. In particular, as temperature increased from 30 °C to 80 °C, the solubility, diffusion, and permeability coefficients of H_2_ in HDPE increased by 18.7%, 92.9%, and 129.0%, respectively. In EVOH, these coefficients increased by 15.9%, 81.6%, and 112.7%. EVOH exhibited superior H_2_ barrier properties compared to HDPE due to the hydroxyl groups in EVOH forming strong hydrogen bonds that impede H_2_ movement. The computed solubilities and permeabilities are shown in Fig. 40 and Fig. 41, and listed in Table 13 and Table 14, respectively. Zhang et al.^750^ showed that the H_2_ permeation mechanism involves two processes: adsorption, where the gas molecules aggregate in low-potential-energy regions; and diffusion, where H_2_ molecules vibrate within confined spaces before occasionally transitioning between holes. Comparisons of the simulation data with experimental measurements of H_2_ permeability in HDPE are provided, showing an agreement within 7.24% difference.

Except from the study of common thermoplastics such as PE and PA used mainly as liner materials in tanks and pipelines, the sorption and diffusivity of H_2_ in poly­(chloro-p-xylene)^754^ (the simulation box containing the polymer is shown in Fig. 42(g)), PDMS Shi et al.,^749^ and in rubbery polymers^755–757^ have been reported in the literature. Stalker et al.^758^ performed simulations of different cellulose configurations, i.e., purely crystalline (which showed no H_2_ sorption), slit pore structures with varying inter-layer spacings (4.67-7.67 Å), amorphous phase, and ”pinned” structures with fixed glycosidic oxygens. A molecular simulation snapshot of the slit pore configuration is shown in Fig. 42(h). H_2_ molecules within the narrow slit pores were shown to occupy discreet positions, being trapped in the empty spaces between the cellulose layers. The authors showed that in the case of wide slit pores, this behavior is not obeyed, and H_2_ has more freedom to move in between the polymer layers. While pure crystalline cellulose was found to be not suitable for H_2_ storage, the modified structures showed potential at experimentally accessible pressures (<100 bar), suggesting that engineered cellulose-based materials could be a viable option. Yi et al.^759^ performed MD simulations investigating the diffusion of H_2_ and its isotopes; deuterium (^2^H) and tritium (^3^H) in PS under normal temperature (298 K) and pressure (1 atm). The research is motivated by the use of PS as a target material in Inertial Confinement Fusion (ICF), where understanding gas diffusion is critical for maintaining ^2^H-^3^H mixtures within the targets. The authors studied PS models with varying degrees of polymerization (i.e., 60, 120, 180, and 240). Interestingly, the PCFF^406^ was used for all species in this study, including H_2_ and the isotopes. Diffusion coefficients were shown to decrease with increasing isotope mass (H_2_ > ^2^H > ^3^H), and decrease with increasing polymer molecular weight. FFV correlates with diffusion behavior, with higher free volume leading to higher diffusion rates. The novel aspect of this study is the investigation of the isotopes diffusion coefficients in PS, which is particularly valuable for ICF research. These gases had not been previously studied in this context despite their importance in fusion applications. The work also establishes relationships between molecular weight, free volume, cohesive energy density, and diffusion behavior that could guide the development of improved target materials with reduced gas permeability. To the best of our knowledge, studies of H_2_ permeation in polymers using coarse-grained approaches are limited (e.g., see Zhao et al.^760^) and out of this review.

Synthetic thermoset polymers which are primarily used as components in auxiliary equipment (e.g., gaskets, sealing rings) in H_2_ storage infrastructure, with most notable the EPDM rubber, have been studied via molecular simulation. Wilson and Frischknecht^761^ used an MC–based method to create a model of cross–linked EPDM. Subsequently, MD simulations using LAMMPS^762^ were performed to study the rubber’s behavior when filled with high H_2_ at near–ambient temperature. The OPLS-AA^428^ force field was used for all spcecies. The authors computed H_2_ solubilities (as a function of cross–link density) and diffusivities (as a function of the gas concentration). The reported data are in–line with previous simulations and experiments. The authors showed that rapid H_2_ depressurization caused system volume to increase, and H_2_ – accessible free volume to expand (with effects being more pronounced at high H_2_ concentrations). Higher cross-link density increased H_2_ – accessible free volume but reduced volume changes during decompression, and produced fewer large pores post–decompression. These findings indicate that higher cross–linking densities might help prevent cavitation in EPDM, though the additional free volume from cross–links could create potential cavitation initiation sites. The authors recommend experimental investigation of crosslinking density effects on high–pressure H_2_ – induced EPDM failure as a potential future research direction. In a complementary study by the same research group, Brownell et al.^763^ performed all–atom classical MD simulations to examine how compositional changes affect gas diffusion in EPDM, in an effort to guide material design to rubbers less prone to H_2_ – induced failure. The simulations revealed anomalous, sub-diffusive H_2_ transport. Two distinct gas groups were identified: high–mobility gas and low–mobility gas constrained by polymer interactions. Lower temperatures increased gas localization in the low–mobility group, creating necessary conditions for cavitation damage. At reduced temperatures, higher cross–link density enhanced H_2_ mobility and decreased trapped H_2_ fraction, suggesting increased cross–linking may minimize cavitation precursors. The authors applied a two–state kinetic model to determine energy requirements for transitions between mobility states.

Recently, Wilson et al.^764^ expanded prior studies of EPDM by studying a composite material which incorporates silica into the rubber. The motivation is that elastomers with nano-sized fillers such as silica or carbon black achieve improved mechanical properties. Filler dispersion and filler-polymer interfacial strength are crucial factors in this enhancement. Interfacial strength is vital for component durability in pressurized gas sealing applications e.g., O-rings, where poor filler-polymer binding can create internal void formations. To understand fundamental mechanisms behind pressurized H_2_-induced elastomer failure, the authors performed MD simulations to examine H_2_ over-saturation effects on filler-polymer interaction strength. Consistently with their prior studies, the OPLS-AA^428^ force field was used to model the polymer, while the parameters by^765^ were for the silica surface. The authors systematically investigated the interface between EPDM and silica across varying H_2_ gas concentrations, cross-link densities, and surface chemistries. The simulations showed that high H_2_ concentrations (1000 H_2_ molecules) caused significant interfacial failures, including hole formation and complete debonding, while lower concentrations (up to 750 H_2_ molecules) did not cause debonding. H_2_ preferentially accumulated at silica-EPDM interfaces, creating concentration gradients that persisted after decompression. Higher H_2_ concentrations reduced adhesive strength between silica and EPDM, with surface hydroxylation and crosslink density influencing the debonding behavior. The results suggest that dissolved high-pressure H_2_ can compromise silica-EPDM interfaces, potentially leading to bubble formation and cavitation-induced failure in H_2_ environments.

As shown in Figs. 40 and 41, and the Tables 13 and 14, the simulation data reported in literature regarding H_2_ solubilities and permeabilities in different polymeric matrices significantly vary. The computed solubilities and permeabilities collected from different authors span three orders of magnitude. While this is expected up to a degree due to the different pressures and temperatures considered, the fact that most of the studies use the same force fields (primarily COMPASS^316^ and PCFF^406^) and similar methods, makes the discrepancies shown in Figs. 40 and 41 interesting. Despite the common elements in these studies, different H_2_ force fields, system sizes, polymer chain lengths, and simulation schemes (e.g., for equilibration and production runs) are used, leading to diverging results. Another striking outcome after reviewing these datasets is that while all simulations seem to be able to capture the experimental permeability trend (i.e., qualitative agreement), the quantititative difference is more than three orders of magnitutes as shown in Fig. 41. This finding alone is a strong indication that there is still ground to cover for producing accurate predictions for H_2_/polymer systems using molecular simulation. It is also important to mention here that caution should be exercised when interpreting computed results for solubilities and permeabilities due to the inherent difficulties in these simulations having to do with slow dynamics, generic force fields, and computationally demanding systems. Another interesting point is that although we do not show here the collected data on computed diffusivities (which are used to compute permeabilities), none of the studies apply corrections to account for finite size effects.^498–500^ It is not clear how big such effects are for the H_2_/polymer systems, however, no investigation has been performed to shed light on this.

### 4.5 Hydrogen Solubility, Diffusivity, Permeability, and Interfacial Properties in Ionic Liquids

Separation of gases using ILs has been an active field in the molecular simulation community the past 20 years. Due to the ever increasing interest in developing carbon capture technologies for environmental and industrial purposes,^766^ the vast majority of the ILs simulation studies have focused on both pure and mixtures of CO_2_, with a lot of effort put on scrutinizing the diverse families of ILs. Following the same route, various thermodynamic and transport properties of H_2_/ILs computed via MC and MD simulations have been reported to date. Shi et al.,^767^ motivated by the carbon capture potential of the so-called supported IL membranes, performed molecular simulations to study the solubility, diffusivity, permeability, and partial molar properties of pure H_2_, CO_2_, and Ar, and their mixtures in 1-*n*-hexyl-3-methylimidazolium bis­(trifluoromethylsulfonyl)­amide ([hmim]­[Tf_2_N]). Molecular representations of the ions of these ILs are shown in Fig. 43. Two different H_2_ force fields were compared: Buch^312^ and Cracknell^311^ (see Section 3.1.1). Notably, while the original Cracknell model is rigid, the authors implemented a flexible version to make it compatible with their hybrid MC method which involves a time reversible integrator (the authors explicitly note that traditional constraint dynamics methods for rigid molecules are time reversible, and thus, the original rigid H_2_ could not be used in their scheme). Also, the effect of polarizability of H_2_ on solubility was investigated, but it was shown to be negligible. The simulations were performed in a temperature range of 313–573 K and for pressures 1–500 bar for pure H_2_, and 50–300 bar for mixed gas systems. The simulations showed that H_2_ solubility in the IL increased with temperature, contradicting some prior experimental studies. Both force fields gave similar results for thermodynamic properties, though the Cracknell model showed better agreement with experimental data, particularly at high pressures. The molar volume of the ionic liquid was found to be the primary determinant of H_2_ solubility, with a clear linear relationship between the Henry coefficient and molar volume. Based on an energy analysis, the interactions between H_2_ and IL were shown to be ca. three to six times weaker than the interactions of the other gases with the IL. The selectivity of CO_2_ over H_2_ decreased from ca. 30 at 313 K to ca. 3 at 573 K. The authors showed that H_2_ diffuses 5–12 times faster than CO_2_, while the permeability of H_2_ was 2.3–3.5 times larger than experiments. Shi et al.^767^ highlight substantial differences in experimentally measured H_2_ solubilities between research groups. These discrepancies (some showing solubility increasing with temperature, others showing the opposite) indicate difficulties in measuring solubility for such poorly soluble gases. The paper reports negative volume expansion when H_2_ dissolves in the ionic liquid at high pressures, suggesting a compression effect. This is somewhat counterintuitive but can be explained by the high pressures required for appreciable H_2_ solubility. The authors conclude that good IL candidates for CO_2_/H_2_ separation should have low molar volumes (to reduce H_2_ solubility) while maintaining high CO_2_ solubility through strong interactions. This somewhat contradicts the observation that molar volume is positively correlated with gas solubility in general.

**Figure 43:**
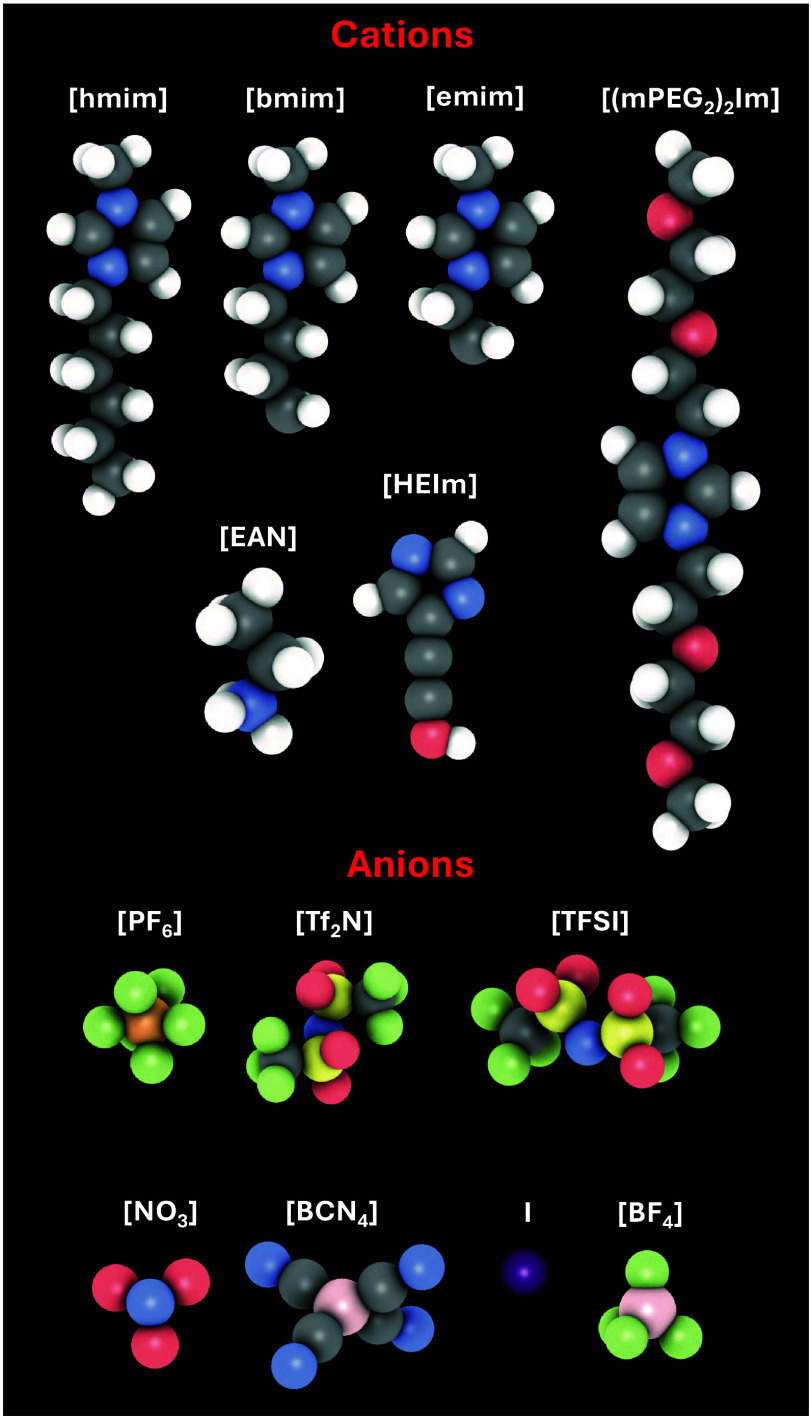
Molecular representations of the cations and anions of the ionic liquids studied in the papers reviewed here (iRaspa^483^ visualization software was used). Color code for the atoms: gray: carbon, white: hydrogen, blue: nitrogen, red: oxygen, green: fluorine, dark yellow: phosphorous, yellow: sulfur, pink: boron, purple: iodine.

In a follow-up study, Shi and Sorescu^768^ investigated how confining [hmim]­[Tf2N] IL in (20,20 and 9,9) CNTs (composite material) affects gas sorption properties. The authors used the same flexible version of Cracknell model.^767^ The simulations were performed at temperatures up to 573 K and pressures up to 400 bar. It was shown that H_2_ diffuses ca. 1.5 times faster than CO_2_ in the composite material. In contrast, H_2_ diffuses almost 10 times faster than CO_2_ in both CNT and bulk IL. At 313 K, H_2_ in the (20,20) CNT was shown to diffuse ca. 4900 times faster than in the composite material. CNT showed the highest H_2_ sorption, followed by composite material and the bulk IL. For increasing temperature H_2_ sorption: (i) increased in [hmim]­[Tf2N], (iii) decreased in CNT, and (iii) and remained almost unaffected in the composite material, which exhibited higher CO_2_/H_2_ sorption selectivity than both IL and CNT ( $S_{{\mathrm{CO}}_{2}/H_{2}}=15.0\pm 1.0,39\pm 2$ , and 79 ± 2 for CNT, IL, and the composite, respectively). The authors suggest that the composite material would be superior to both IL and CNT for the CO_2_/H_2_ separation.

Singh et al.^391^ presented a MC simulation study of the solubility of CO_2_, H_2_, and their mixtures in ILs 1-alkyl-3-methylimidazolium bis­(trifluoromethanesulfonyl)­amide ([C_*n*_mim] [Tf2N], *n* = 4, 6). Molecular representations of the ions of these ILs are shown in Fig. 43. The motivation was the CO_2_/H_2_ separation in pre-combustion processes, for which understanding *S*_CO_2_/H_2__ in high-pressure/temperature gas mixtures is crucial. The authors have implemented advanced sampling algorithms (e.g., cavity-biased, configurational-biased, and “slow-growth” - for more details on these techniques, the reader is referred to the refs within Singh et al.^391^) to overcome the inherent difficulties in MC simulations of dense and complex systems, such as ILs. Simulations were performed at 333, 413, and 573 K, and pressures up to 300 and 80 bar for pure H_2_ and mixtures, respectively. It was shown that H_2_ solubility in ILs slightly increases with temperature (positive enthalpy of absorption), while the volume change upon H_2_ absorption were negligible (e.g., <1% at 300 bar). *S*_CO_2_/H_2__ was shown to decrease significantly with temperature (from 40-60 at 333 K to 3-4 at 573 K). Interestingly, this study contradicts previous experimental findings by that suggested that CO_2_ enhances H_2_ solubility at high pressures. The work concludes that CO_2_ and H_2_ are absorbed almost ideally in these ILs, with no significant interactions between the two gases in the liquid phase at the studied conditions.

Ramdin et al.^769^ performed MC simulations to predict the solubility of various pre-combustion gases including H_2_ in 1-butyl-3-methylimidazolium bis­(trifluoromethylsulfonyl)­imide [bmim]­[Tf2N] (note that [bmim] is a different notation for the [C_4_mim] cation, where b stands for butyl). Cracknell^311^ force field was used to model H_2_ and the model by Liu and Maginn^770^ for the IL. The simulations were performed at 333.15 K and pressures up to 150 bar. The authors showed that H_2_ has the lowest solubility among all studied gases (H_2_S > CO_2_ > CH_4_ > CO > N_2_ > H_2_). Simulations under-predicted the H_2_ solubility compared to experiments with the difference being 23-33.0%. The computed *S*_CO_2_/H_2__ was reported to be 38.3, while the experimental is 30.4. This value is close to the commercial solvent Selexol which as a respective selectivity of 25. This may indicate that the IL might not offer significant advantages despite its higher cost. This study showcases that despite the quantitative discrepancies between MC simulation and experiment, one should also keep in mind that solubilities of H_2_ in ILs are difficult to measure experimentally with uncertainties being as high as 50%.

In their MC study, Wittich and Deiters^771^ examined how the choice of simulation box geometry (cubic and orthorhombic) affects H_2_ solubilities in ILs computed. The IL studied was 1-n-butyl-3-methyl-imidazolium hexafluorophosphate ([bmim]­[PF_6_]) for temperatures ranging from 313 to 373 K at atmospheric pressure. Molecular representations of the ions of this IL are shown in Fig. 43. Similarly to the IL studies discussed earlier, the Cracknell^311^ force field was used to model H_2_, while the united-atom model by Shah and Maginn^772^ was used for [bmim]­[PF_6_]. The cubic simulation box was shown to yield Henry’s constant values closer to experimental results for H_2_, but still overestimated the experimental data by 14-57%. The authors observed that simulation box geometry significantly affects diffusivity, with the orthorhombic box showing faster diffusion rates. The authors mention that system size effects are present but could not fully investigate them due to computational constraints. Despite this study suggested that for some properties, such as Henry’s constants, the orthorhombic box may better represent the system by allowing more configurational freedom, this did not hold true for the case of H_2_.

Klein et al.^773^ performed DLS experiments and MD simulations to measure/compute the diffusivities (thermal and fick) of binary mixtures of [emim] / [hmim] / [C_1_0mim] [NTf_2_] with H_2_, He, N_2_, CO_2_, and Kr. This work is part of a large number of studies by the research group focusing on thermodynamic and transport properties of gas/liquid mixtures spanning alkanes, alcohols, and ILs. The temperature and pressure ranges covered were 298 - 423 K and 1 - 65 bar, respectively. The authors used various combinations of force fields (details are provided within ref 773) to identify suitable models for predicting densities, viscosities, and diffusivities. Klein et al.^773^ reported self- (mentioned as thermal) and fick (component *D*_11_) diffusion coefficients. For the whole temperature and pressure ranges, the computed diffusivities were in relatively close agreement (within 12%) with the experiments. The self-diffusivities of H_2_ in [emim]­[NTf_2_] ranged from 2.54 to 6.45 × 10^–9^ m s^–^1 for temperatures 298 to 348 K. For the same temperature range, the respective values for self-diffusivity of H_2_ in [hmim]­[NTf_2_] and [C_1_0mim]­[NTf_2_] were similar (deviations of lower than 10% between the different solvents). Unlike in *n*-alkanes, the diffusivities of the gasses in the ILs were not significantly affected by the viscosity of the solvents.

Recently, Zhai et al.^774^ investigated how H_2_ affects both bulk (viscosity) and interfacial properties (surface tension) of three imidazolium-based ILs using both experiments and MD simulations. The ILs studied were the hydrophobic 1-ethyl-3-methylimidazolium bis­(trifluoromethanesulfonyl) imide ([C_2_C_1_Im]­[NTf_2_], also commonly abbreviated to [emim] [NTf_2_] since e refers to the ethyl group) and 1-methyl-3-octylimidazolium hexafluorophosphate ([C_8_C_1_Im]­[PF_6_]) as well as the hydrophilic 1,3-bis­(2-(2-ethoxyethoxy)­ethyl) imidazolium iodide ([(mPEG_2_)_2_Im]­I). Molecular representations of the ions of these ILs are shown in Fig. 43. To model the ILs, the authors used the force fields by Kelkar and Maginn^388^ and Seidl et al.^775^ with optimized partial charges. For H_2_ the IFF^313^ model was used. The conditions examined where 303-393 K and 1 - 310 bar. The authors showed that H_2_ has low solubility in all three ILs, with maximum values (in mole fraction units) of *χ*_H2_ = 0.092 for [C_2_C_1_Im]­[NTf_2_] at 270 bar and 353 K and *χ*_H2_ = 0.041 for [(mPEG_2_)_2_Im]I at 310 bar and 393 K. It was shown that H_2_ solubility is ca. 3.5 times higher in the hydrophobic [C_2_C_1_Im]­[NTf_2_] than in the hydrophilic [(mPEG_2_)_2_Im]I at 353 K. Notably, H_2_ appeared to only occupy existing voids in the IL rather than creating additional volume It was also shown that H_2_ has no significant effect on the viscosities of ILs for pressures up to 80 bar. Nevertheless, the IL force fields overestimate the viscosity by a factor of 2-2.5 compared to experiments, though they capture the qualitative behavior. MD simulations revealed two counterbalancing effects, i.e., increased pressure compresses the liquid (increasing viscosity) while dissolved H_2_ has a plasticizing effect (decreasing viscosity). Pressures had a small effect on the gas/liquid surface tension (ca. 6% decrease at 80 bar) which is due to the weak enrichment of the IL-gas interface with H_2_. Overall, the effect of H_2_ on the IL properties investigated is similar to what has been observed for non-electrolytic organic solvents.

Sharing the motivation with the studies discussed earlier, Rivera-Pousa et al.^776^ studied H_2_ absorption in the following protic and aprotic ILs: [EAN]­[NO_3_], [HEIm]­[NO_3_], [HEIm] [TFSI], [EMIm] [B­(CN)_4_], [EMIm]­[TFSI], [EMIm]­[NO_3_], [EMIm] [BF_4_], and a series of [C_*n*_MIm]­[BF_4_] with varying alkyl chain lengths (*n* = 2, 4, 6, 8, 10, 12). Molecular representations of the ions of these ILs are shown in Fig. 43. The authors focused on high temperatures (550 K) and pressures (50 bar) because these conditions are relevant for processes such as Fischer-Tropsch synthesis. It was shown that H_2_ solubility increased with alkyl chain length in cations. Also, H_2_ molecules showed weak interactions with the ILs, while they preferentially occupied the apolar domains of the ILs. However, in ILs with nitrate anions, H_2_ also occupied polar regions. Another finding was that the structure of ILs was almost insensitive to the presence of H_2_, while free volume and cavity formation appear to be the most important factors affecting solubility. Interestingly, in this study unscaled charges for ions were used instead of scaled charges that are typically used to prevent sluggish dynamics (see also Section 3.1.3). The study revealed that even though nitrate-based ILs show the most favorable solvation enthalpies, they have the lowest H_2_ solubility due to limited available void space.

### 4.6 Hydrogen Sorption and Diffusivity in Metal- and Covalent-Organic Frameworks and Zeolites

Molecular simulations play a central role in the pursuit of understanding the mechanisms underlying sorption, diffusion, and permeability of H_2_, and the selectivity of H_2_ mixtures in nanoporous media such as MOFs and COFs. For this reason, an extensive literature body is available, making the detailed review of this area here not feasible. Nevertheless, we provide a short overview of important, relevant, and relatively recent studies to showcase the range of applicability of MC and MD simulations in studying H_2_ sorption phenomena, especially in light of AI and ML approaches.

As discussed in detail in Section 2.2, accurate modeling of separations of H_2_/gas mixtures requires accounting for both sorption and diffusion of the species involved, as membrane permeability is determined by both these phenomena. Early simulation studies focused on such properties in MOFs,^777,778^ particularly focusing on separations of H_2_ from CO_2_ and CH_4_. Keskin and Sholl^130^ performed GCMC and MD simulations to compute both sorption and diffusion of H_2_, CO_2_, CH_4_, and N_2_ in MOF-5. Wu et al.^779^ developed a modified force-field for ZIF-8, enabling simulations of H_2_ sorption and diffusion alongside other gases. In 2012, Keskin^780^ reported simulation results for sorption, diffusion, gas permeability, and membrane selectivity of H_2_ over CH_4_, focusing on COFs. The authors demonstrated the superior performance of COF-6 compared to zeolites and MOFs. Krokidas et al.^418^ explored the potential of functionalization of MOFs to design novel structures for membranes, offering unprecedented H_2_/CH_4_ separation efficiency.

Song and No^781^ used GCMC calculations to study the influence of available volume, surface area, cation types (e.g., Mg, Mn, and Ca), and pre-adsorbed benzene on the H_2_ capacity of zeolites. Song and No^781^ showed that the available volume and surface area per mass of zeolite are the two parameters having the strongest correlation with H_2_ storage capacities, and that pre-adsorbed benzene can significantly decrease H_2_ storage capacity by lowering the available volume in the zeolite structure. Deeg et al.^782^ investigated H_2_ adsorption in two all-silica zeolites (i.e., ITQ-29 and MFI) at cryogenic temperatures using molecular simulations. This study revealed that a single-site H_2_ force field with an uncharged LJ center can best reproduce experimental adsorption isotherms, while small adjustments to LJ parameters can significantly improved accuracy. It was also shown that in ITQ-29, H_2_ preferentially adsorbs onto sodalite cages at low temperatures, creating a unique temperature-dependent pattern in heats of adsorption. In MFI, adsorption occured in sinusoidal channels at low temperatures.^782^ The authors also incorporated quantum effects via the Feynman-Hibbs potential (see Section 3.1.1), demonstrating the importance of considering the quantum nature of H_2_ to accurately model adsorption, especially at very low temperatures (i.e., 25 K). Extensive reviews of molecular simulations of H_2_ adsorption on functionalized nanoporous materials are provided by Getman et al.^317^ and Bobbitt and Snurr.^414^

Simulations have also played a crucial role in advancing the study of MMMs, effectively combining the cost-effective and scalable attributes of polymers with the high-performance capabilities of MOFs/COFs as fillers. Yilmaz and Keskin^783^ explored diverse combinations of MOFs and polymers for H_2_/CH_4_ and H_2_/CO_2_ separations, towards the development of high-performing MMMs. The computed gas selectivity and permeability were compared with experimental data, showing good agreement. The authors also proposed new MOF-based MMMs exhibiting high performance for specific gas separations.

#### 4.6.1 High-Throughput Screening Simulations

So far in this review, insights into H_2_ separation mechanisms obtained solely from molecular simulations have been discussed. Nevertheless, the growing availability of structural databases and computational resources enabled a shift towards large-scale, high-throughput screening studies. This approach, comprising massive numbers of simulations, allows for the systematic evaluation of thousands of candidate materials, offering a data-driven perspective on separation performance. Early efforts focused on extracting simple structure-property relations from these datasets, often using visual/manual inspection of simulation results or heuristic approaches based on intuitive structural descriptors such as pore size or surface area.^784^ In recent years, however, the adoption of ML and AI has allowed for a more powerful and automated analysis of such relationships, enabling faster and more accurate prediction of material performance across vast chemical spaces. Such data-intensive approaches are efficient since the different structures comprising each family of nanoporous materials are in the order of hundreds of thousands. For example, ca. 100000 different MOF structures have been reported in literature, and more than half a million other MOF structures have been predicted to date.^785^

In the past decade, the significant advancements in high-performance computing have enabled researchers to conduct large-scale screening simulation studies, allowing for the evaluation of numerous material structures in terms of separation performance.^786^ For instance, Avci et al.^787^ performed high-throughput computational screening on a comprehensive subset of MOFs of the CSD database,^788^ exploring the CO_2_ capture and H_2_ purification potential of 10221 MOFs. These simulations revealed the best-performing materials for PSA, vacuum swing adsorption (VSA), and TSA processes, outperforming commercial zeolites and previously studied MOFs in terms of CO_2_/H_2_ selectivity (see Fig. 44a).

**Figure 44:**
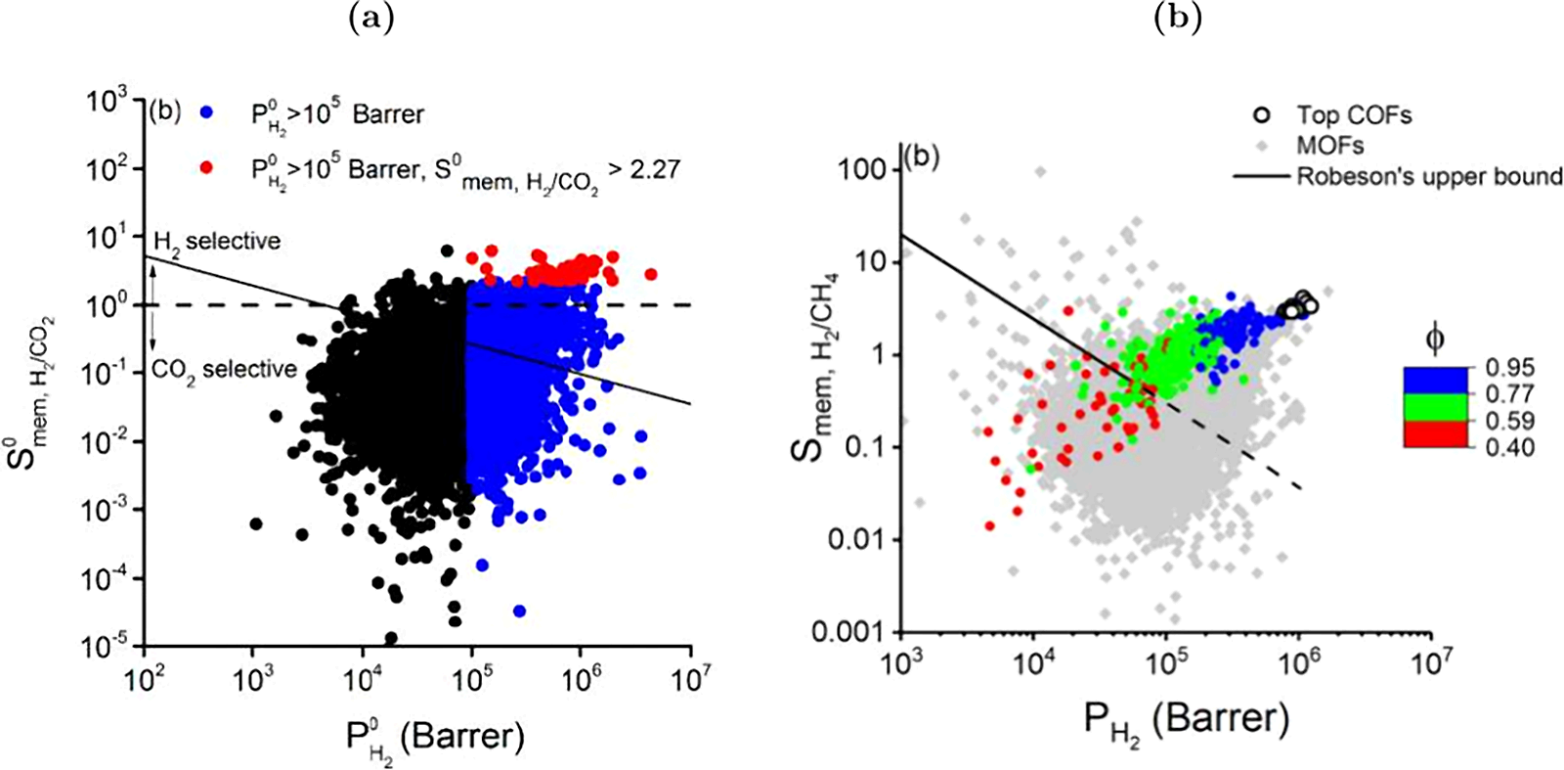
Robeson plots of (a) H_2_/CO_2_ membrane selectivities as a function of H_2_ permeability for the MOFs studied by Avci et al.,^787^ and (b) the high-performing CH_4_/H_2_ COFs, highlighting the importance of porosity, *ϕ*, as a driving characteristic of separation performance.^789^ [Panel (a) is reprinted from Avci et al.,^787^ ACS Applied Materials & Interfaces, Vol 12, 41567–41579. Copyright 2020, American Chemical Society. Panel (b) is reprinted from ref 789. Industrial & Engineering Chemistry Research, Vol 60, 12999–13012. Copyright 2020, American Chemical Society (this publication is licensed under CC-BY-NC-ND 4.0)].

Similarly, Altintas et al.^790^ focused on the comparison of two widely used computation-ready MOF databases, i.e., CoRE^791^ and CSDSS.^792^ The authors id entified 3490 common MOFs in these databases and conducted simulations to compute their CH_4_ and H_2_ uptakes. The study also highlighted ’problematic’ MOFs exhibiting different gas uptakes depending on the database used, leading to significant variations in ranking and material selection. Aksu et al.^793^ focused on COFs for CO_2_/H_2_ separation using a high-throughput computational screening approach on the CoRE COF.^794^ This study covered 288 experimentally synthesized COFs, providing valuable insight into the performance of COFs as adsorbents and membranes for pre-combustion CO_2_ capture. Notably, many COFs outperformed traditional zeolites in terms of CO_2_ selectivity and working capacities. The structural analysis revealed that COFs with specific pore sizes and porosities are potential adsorbents for selective CO_2_/H_2_ separation. Aksu et al.^149^ extended the screening to a database of hypothetical COFs (hypoCOF). Through an extensive series of molecular simulations, the authors identified top-performing hypoCOFs as adsorbents and membranes for CO_2_ capture and H_2_ purification. The findings of this study indicated higher CO_2_ selectivities and working capacities compared to experimentally synthesized COFs under specific conditions. The study also emphasized the importance of hydrogen bonding between CO_2_ and functional groups of linkers for CO_2_ selectivity in hypoCOFs. Altundal et al.^789^ explored the separation potential of COFs for CH_4_ purification from gases such as H_2_, N_2_, and C_2_H_6_. By combining GCMC and MD simulations, they assessed adsorption and membrane-based separation performances of 572 COFs. The results demonstrated that COFs have the potential to outperform conventional adsorbents in H_2_/CH_4_ separations. Additionally, this study identified structural features related to high selectivities and permeabilities, offering a basis for designing COFs with exceptional CH_4_ separation performance. For example, Fig. 44b shows that higher porosity values, *ϕ*, result to higher separation performance. Avci et al.^795^ investigated the influence of metal exchange in MOFs on CO_2_/H_2_ and CO_2_/CH_4_ separation performances. By exchanging Zn nodes with different metals, they obtained 32 metal-exchanged MOFs (M-MOFs), and showed that the exchange of Zn with V and Cr significantly improved CO_2_ uptakes and adsorption selectivities. These molecular-level insights can provide a valuable guide for designing MOFs with enhanced CO_2_ separation efficiency.

Zeolites, are another class of porous media similar to MOFs and COFs in many aspects. Zeolites are crystalline aluminosilicate materials characterized by a well-defined, micro-porous structure composed of interconnected channels and cages. These materials have long been studied for applications in gas storage and separation because of their inherent thermal and chemical stability. Despite their attractive structural features, the storage capacities of H_2_ in unmodified zeolites (e.g., without structural modifications and doping with metals or other additives) are unsatisfactory (i.e., ca. 0.2-1.1 wt% H_2_).^796^ Molecular simulations are used in the literature to identify critical features that can enhance H_2_ storage capacities and to design new zeolite structures suitable for H_2_ storage applications.^781,782,797,798^ Manda et al.^797^ explored the H_2_ storage capacities of 233 zeolites using GCMC simulations combined with ML. An impressive H_2_ storage capacity of 4.8 wt% H_2_ was reported in the Linde Type A structure, attributed to the low mass of the zeolite and the large gravimetric and volumetric surface area.^797^ For a more detailed discussion of H_2_ storage in zeolites the reader is referred elsewhere.^796,797,799^

#### 4.6.2 Artificial Intelligence and Machine Learning to Accelerate Material Screening

Building on the advancements of high-throughput screening and the increasing availability of structure databases, such as the aforementioned CoreMOF,^800^ hypoCOF,^801^ CoRE COF,^802^ as well as, the Inorganic Crystal Structure Database (ICSC),^803^ Crystallographic Open Database (COD),^804^ and the Cambridge Structural Database (CSD),^805^ the scientific community has witnessed a flourishing interest in the utilization of AI and ML as a powerful approach for screening and designing H_2_-related processes involving MOFs and COFs. Particularly in the context of functionalized, nanoporous solids, these techniques offer a remarkable opportunity to explore and comprehend intricate structure-property correlations.^806^ Ahmed et al.^198^ evaluated the H_2_ storage capacities of ca. 20000 different MOF structures using MC simulations (results are shown in Fig. 45). The authors, then, used the data to train a ML model, which was used to screen nearly half a million different MOF structures.

**Figure 45:**
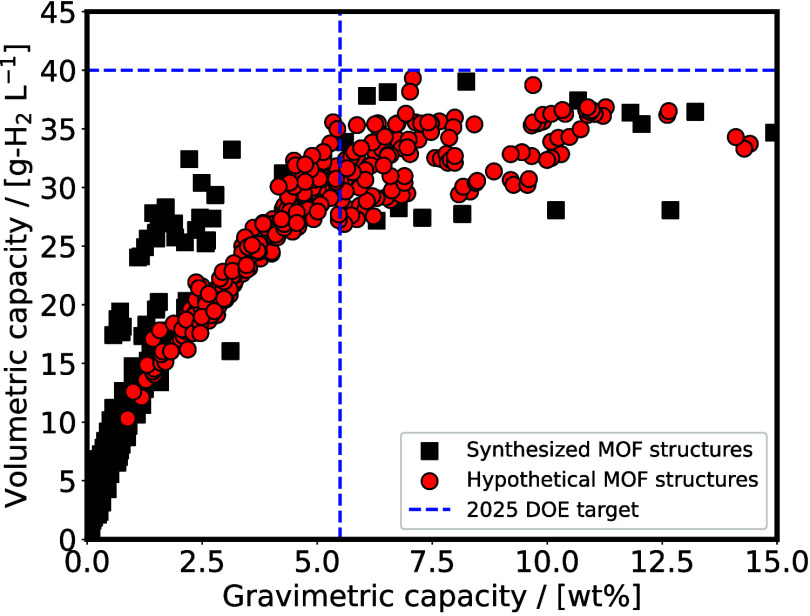
Volumetric and gravimetric capacities of H_2_ for different MOFs computed with using GCMC simulations.^198^ The volumetric and gravimetric capacities are computed at 77 K for a pressure swing of 100 bar to 5 bar. Various MOF structure databases were utilized for the GCMC simulations conducted by Ahmed et al.,^198^ including databases containing synthesized MOF structures as well as databases of hypothetical MOF structures. The data for this plot is obtained from the work of Ahmed et al.^198^

Recently, Chen et al.^151^ presented empirical-Morse force fields based on ab-initio calculations for interactions of H_2_ with Mg-alkoxides. The empirical force fields are then used to perform high-throughput GCMC simulations of ca. 3000 metal-alkoxide functionalized COF structures, and to develop ML models to predict H_2_ capacities.^151^ Bucior et al.^807^ developed a data-driven approach using sparse regression models to predict gas adsorption in MOFs, enabling rapid exploration of large databases and identification of top-performing candidates like MFU-4l. Similarly, Dureckova et al.^808^ used ML to construct quantitative structurerelationship (QSPR) models for CO_2_ working capacities and CO_2_/H_2_ selectivities in MOFs. Their work demonstrated that ML can accurately predict the performance of MOFs, even in topologically diverse databases, while significantly accelerating the screening process. Yang et al.^809^ utilized ML algorithms, such as decision trees and random forests, to predict the separation performance of computational-ready MOF membranes for H_2_-related separations, such as H_2_/CO_2_, H_2_/CH_4_,H_2_/N_2_ and He/H_2_. By analyzing structural feature descriptors and principal components, the authors successfully identified the best-performing MOFs for each binary gas mixture.

Shi et al.^810^ summarized recent progress in ML-assisted high-throughput computational screening of MOFs, including applications in CH_4_ storage, H_2_ storage, and CO_2_ separations. The authors showed that the use of ML models improved the screening speed by several orders of magnitude and facilitated the identification of high-performance materials. Bai et al.^811^ explored the application of high-throughput computational screening and ML for evaluating the H_2_ separation performances of COF membranes. Their models efficiently predicted permeability and selectivity, enabling the identification of top-performing COFs for H_2_ separation. Fig. 46 shows the performance of Gaussian Processes (GP) and Random Forest (RF) regression models in predicting the permeability of TMSP of H_2_, in the form of parity plots (i.e., predictions vs simulation results). Aksu and Keskin^153^ introduced a high-throughput computational screening approach combined with ML for assessing synthesized and hypothetical COFs for CH_4_/H_2_ separation. By developing ML models based on simulation results on 7700 COFs of the 69800 in total in their dataset, these authors accurately predicted the adsorption properties of hypothetical COFs and identified the top-10 performing COFs in VSA and PSA processes as shown in Fig. 47.

**Figure 46:**
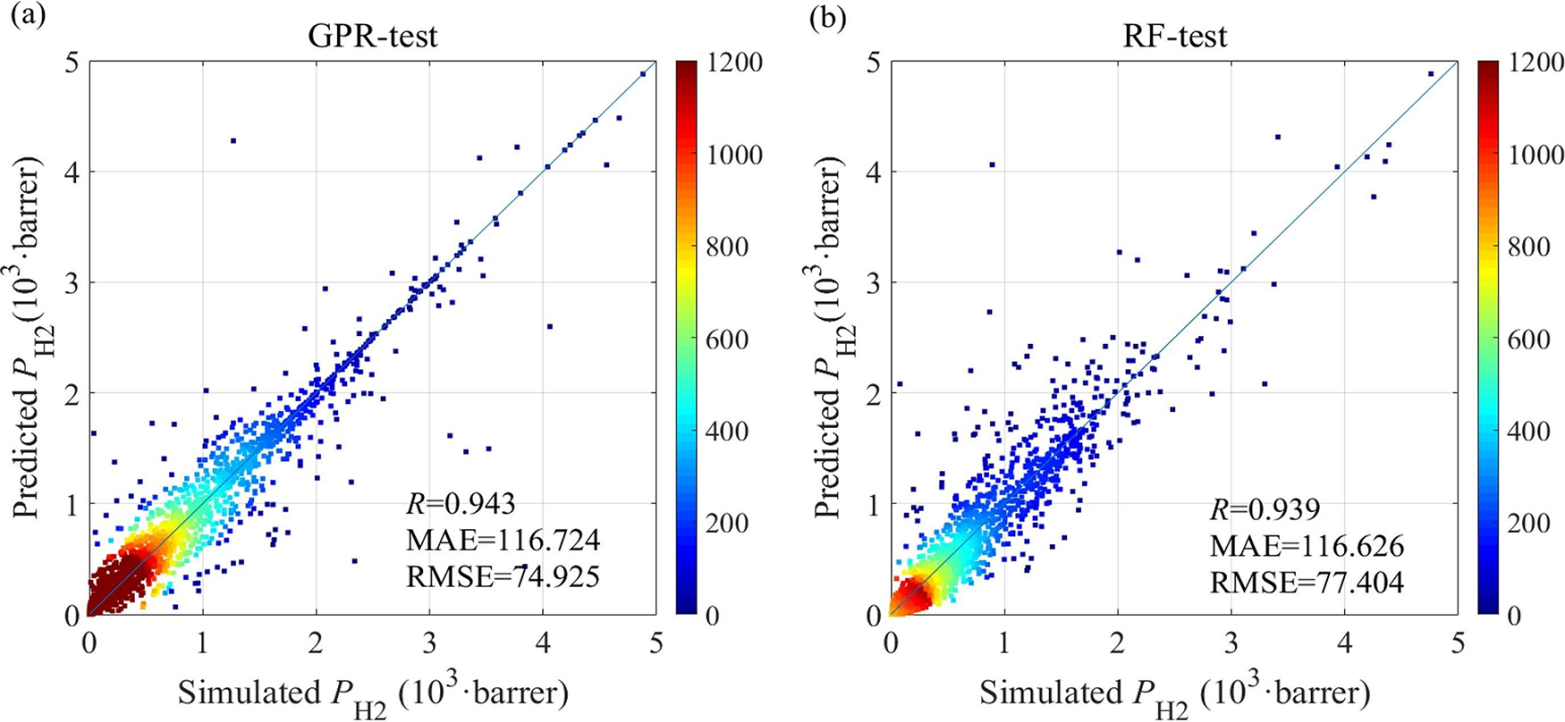
Predictions compared with the data of the test set (simulations) for *P*_H_2__ made with (a) GP and (b) with RF, from the work of Bai et al.^811^ [The figure is reprinted from Chemical Engineering Journal, Vol 446, Xiangning Bai et al., Machine–Learning–Assisted High–Throughput computational screening of Metal–Organic framework membranes for hydrogen separation, Copyright (2022), with permission from Elsevier].

**Figure 47:**
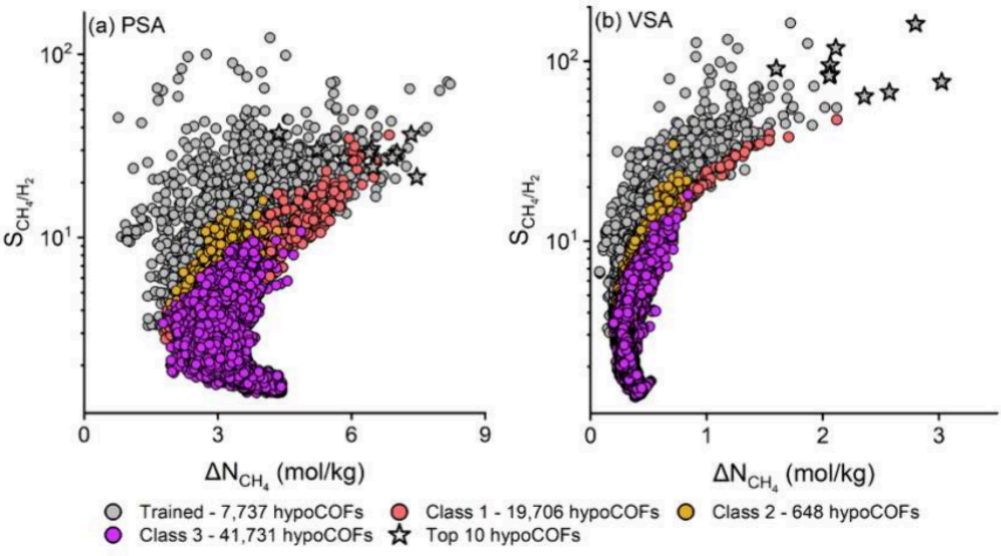
ML predictions of CH_4_/H_2_ performance for (a) PSA and (b) VSA processes. [The figure is reprinted from Journal of Materials Chemistry A, Aksu and Keskin,^153^ Advancing CH_4_/H_2_ separation with covalent organic frameworks by combining molecular simulations and machine learning (This article is licensed under a Creative Commons Attribution-NonCommercial 3.0 Unported Licence - CC BY-NC 3.0)].

As a concluding remark, it is important to highlight that the design of materials for H_2_-gas separations, with molecular simulation as the enabling tool, can pave the way for membranes with unprecedented performance. Fig. 48 illustrates this potential by comparing predicted materials from simulations with state-of-the-art synthesized membranes. These Robeson plots show that *in silico* designed nanoporous materials, such as MOFs and COFs, not only outperform conventional polymer membranes but also exceed the performance of previously studied functionalized porous materials. Notably, many of the predicted structures lie beyond the traditional upper bounds for both H_2_/CH_4_ and H_2_/CO_2_ separations, demonstrating that simulation-guided design can expand the accessible membrane performance space. Table 17 provides a list of the available simulation data from high-throughput screenings and ML studies in literature. We provide data for the diffusivity, permeability, and sorption of H_2_ mixtures on various functionalized nanoporous materials. It is important to note that most computational studies of MOF/COF membranes assume defect-free, perfectly crystalline, and rigid structures. While this practice is useful for identifying intrinsic transport limits, it represents a highly idealized model which may differ significantly from real, defect-containing, and flexible membranes.

**Figure 48:**
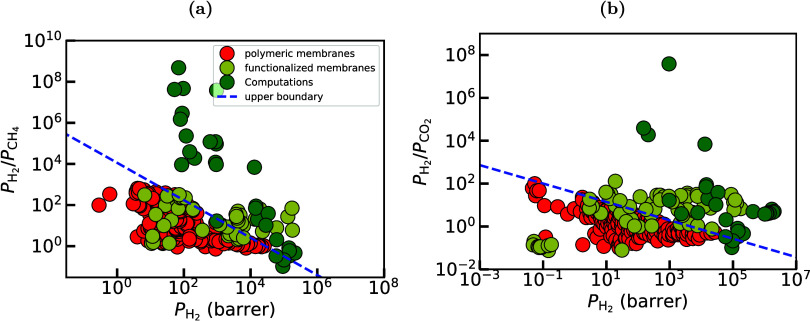
Robeson plots of (a) H_2_/CH_4_ and (b) H_2_/CO_2_ separation depict the impact of computational-empowered design. Functionalized nanoporous materials engineered through simulations surpass the current, experimentally measured polymers and functionalized nanoporous materials, showcasing unprecedented gas separation performance. The literature results on polymeric membranes are taken from the seminal work of Robeson.^136^ The experimental and computational data for functionalized materials are collected from an extended survey of the authors, and are listed in Tables 15, 16, and 17.

**Table 17:** Sorption, Diffusivity, and Permeability Data Collected from Molecular Simulation Studies of Pure H_2_ and H_2_ Mixtures in Different Functionalized Nanoporous Materials

		Sorption Selectivity		Diffusion Selectivity			Permeability Selectivity		
Study	Material	CH_4_/H_2_	CO_2_/H_2_	H_2_/CH_4_	H_2_/CO_2_	*D*_H_2__(10^–10^m^2^/s)	H_2_/CH_4_	H_2_/CO_2_	*P*_H_2__ (barrer)
Yang et al.^876^	MOF-5	5							
Liu et al.^778^	Cu-BTC	10							
	IRMOF-9	13.51							
	IRMOF-10	3.48							
	IRMOF-11	18.53							
	IRMOF-12	6.31							
	IRMOF-13	17.15							
	IRMOF-14	5.3							
Wu et al.^779^	ZIF-8			571.43	41.67	2.28			
Keskin^780^	COF-5	9.894		6.06			0.612		
	COF-6	17.223		4.785			0.272		
	COF-10	4.741		7.09			1.484		
Krokidas et al.^418^	ZIF-8	14.1	21.8	277.8	33.3	1.6 × 10^–8^	20	1.49	1.62 × 10^4^
	ZIF-67	14.1	24.1	2 × 10^–3^	9.1	8.7 × 10^–9^	16.67	0.09	9.66 × 10^2^
	BeIF-1	28.9	49.1	1.1 × 10^9^	45.5	9.4 × 10^–10^	3.85 × 10^7^	0.62	9.55 × 10^2^
	ZIF-7-8	23.7	47.4	4.81 × 10^5^	238.1	2.4 × 10^–10^	1.9 × 10^4^	3.15	2.1 × 10^2^
	Co-ZIF-7-8	25.1	49.1	9.43 × 10^5^	90.9	1.7 × 10^–10^	3.9 × 10^4^	3.49	1.5 × 10^2^
Yilmaz et al.^783^	ZIF-2			2.48	11.7	6.20 × 10^–8^	0.104	0.175	9.21 × 10^4^
	ZIF-3			7.33	73.3	1.10 × 10^–7^	0.343	0.987	1.49 × 10^5^
	ZIF-10			5.71	13.3	1.60 × 10^–7^	0.465	0.409	2.13 × 10^5^
	ZIF-11			9.23	17.1	1.20 × 10^–7^	0.812	0.598	1.41 × 10^5^
	ZIF-12			1.86 × 10^3^	15.3	1.10 × 10^–8^	88.3	0.251	1.45 × 10^4^
	ZIF-60			1.45 × 10^11^	2.16 × 10^9^	1.1	70.4	0.460	1.41 × 10^4^
	ZIF-65			6.32	23.1	1.20 × 10^–7^	0.593	0.658	1.45 × 10^5^
	ZIF-67			5.32 × 10^2^	8.25 × 10^2^	3.30 × 10^–8^	38.7	11.6	3.37 × 10^4^
	ZIF-69			1.29 × 10^5^	22.9	1.10 × 10^–8^	6.9 × 10^3^	0.409	1.30 × 10^4^
	ZIF-78			54.5	1.13 × 10^2^	5.40 × 10^–8^	2.05	1.67	6.02 × 10^4^
	ZIF-79			2.89 × 10^2^	2.00 × 10^2^	2.80 × 10^–8^	10.4	2.02	2.88 × 10^4^
	ZIF-81			7.00	91.3	8.40 × 10^–8^	0.214	1.34	9.93 × 10^4^
	ZIF-90			9.00	2.84 × 10^2^	5.40 × 10^–8^	0.333	4.03	5.80 × 10^4^
Avci et al.^787^	FOTNIN		12		76.05	1.35 × 10^–6^		6.34	1.67 × 10^6^
	BAZGAM		3.47		17.42	1.51 × 10^–6^		5.02	1.97 × 10^6^
	PESSUE		3.93		19.42	3.86 × 10^–7^		4.94	4.26 × 10^5^
	XANLIJ		2.95		13.55	8.39 × 10^–7^		4.59	1.83 × 10^6^
	AVAJUE02		4.1		18.06	1.01 × 10^–6^		4.4	1.33 × 10^6^
	XAHQAA		4.52		18.84	1.06 × 10^–6^		4.17	1.40 × 10^6^
	KAZSIQ		7.25		29.82	9.08 × 10^–7^		4.11	1.21 × 10^6^
	CEFNOT		5.25		20.94	8.67 × 10^–7^		3.99	1.15 × 10^6^
	XAHPUT		4.72		18.47	8.83 × 10^–7^		3.91	1.16 × 10^6^
	NIBJAK		4.78		18.17	7.65 × 10^–7^		3.8	1.02 × 10^6^
Aksu et al.^793^	PE-COF-I		368.2						
	(for PSA)								
	NPN-2 (for PTSA)		2196.1						
	NPN-2 (VSA)		566.3						
	NPN-1 (TSA)		856.8						
	NPN-1 (VTSA)		936						
Aksu et al.^149^	linker99_C_linker100		229						
	_C_pts (PSA)								
	linker92_C_linker92		336						
	_C_bpi (VSA)								
	linker103_CH_linker89		0.49		12.5			6.14	
	_N_unh (membrane)								
	linker91_CH		0.67		9			6.04	
	_linker95_N_bod								
Altundal et al.^789^	CTF-FUM (VSA)	102.7							
	COF-303	21.84							
Yang et al.^809^	TUMGOX						1.2 × 10^4^	4.39	8.9 × 10^2^
									(H_2_/CH_4_)
									7.75 × 10^3^
									(H_2_/CO_2_)
	HEDCEA						9.4 × 10^3^	3.95	9.35 × 10^2^
									(H_2_/CH_4_)
									2.35 × 10^3^
									(H_2_/CO_2_)
Bai et al.^811^	IDAZEU						9.4 × 10^4^		935.42
	WENSIS						1.2 × 10^5^		890.06
	YAFGAP						1.2 × 10^5^		614.29
	BEVQUP						4.7 × 10^7^		95.68
	BEVQID						2.3 × 10^5^		117.45
	JASNEX						2.9 × 10^6^		88.9
	NIRDEX						1.5 × 10^6^		78.75
	HIWXER						4.2 × 10^7^		52.27
	NAXKOO01						9.2 × 10^3^		85.78
	EMANAH						4.8 × 10^8^		69.68

### 4.7 Adsorption and Diffusivity of Hydrogen in Clay-Rich Reservoirs

Here, we summarize the findings from MD and GCMC studies (also see Table 18) to clarify how the insights presented for mechanisms such as adsorption, solubility, diffusivity, and intercalation processes influence the efficiency of UHS. We first discuss competitive adsorption on rock surfaces, then discuss H_2_ solubility in confined water, pore-scale transport dynamics, and intercalation within layered geological structures. Researchers frequently use substrates such as kaolinite and graphite in H_2_ storage studies due to their distinct yet complementary properties, enabling systematic exploration of adsorption and diffusion at the molecular scale. Kaolinite, an abundant clay mineral characterized by a 1:1 layered structure, exhibits two distinct surfaces (hydroxylated (gibbsite) and siloxane) resulting in varying H_2_–solid interactions. These properties make kaolinite ideal for modeling the heterogeneous pore surfaces found in subsurface reservoirs.^877^ Conversely, graphite and its derivative graphene offer high chemical stability, low mass, and a well-defined 2D structure, serving as proxies for organic-rich fractions commonly found in shale formations.^878,879^ The utilization of these representative substrates enables the systematic evaluation of how pore size, temperature, and pressure influence adsorption and diffusion, which are key properties for assessing UHS viability. Additionally, the well-characterized nature of materials such as kaolinite and graphite facilitates the validation of molecular simulations against experimental data, thereby refining theoretical models. The distribution of substrates adopted in the reviewed studies is summarized in Fig. 49.

**Figure 49:**
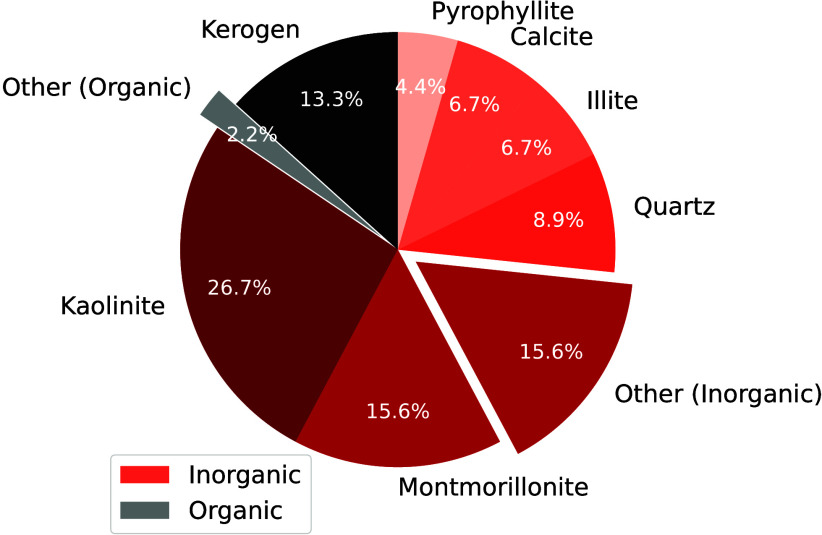
The substrates considered in the molecular simulation studies reviewed in this section. The sectors in shades of red and black represent inorganic and organic materials, respectively.

**Table 18:** Overview of the Literature on H_2_ Adsorption in Geological Substrates^a^

Author	Materials/Force Fields	Method	*T* [K]/*P* [MPa]	Pore Size [nm]	Mixture(s)
Wang *et al.* ^905^	Na-Montmorillonite (CLAYFF)^422,423^	EMD and NEMD	343 K /Not mentioned	3.7–8.2	CH_4_, H_2_, H_2_O
	H_2_ (Cracknell Two-site)^311^				
	CH_4_ (TraPPE-UA)^301^				
	H_2_O (SPC/E)^346^				
Oliver *et al.* ^879^	Kaolinite - Gib.^1^ and Sil.^2^ (CLAYFF)^422,423^	EMD	300 K / 1.0-50.7 MPa	1–20	H_2_
	Graphene (OPLS)^428^				
	H_2_ (Michels Single site)^924^				
Ho *et al.* ^880^	Kerogen (CVFF)^426^	GCMC	338 K / 0–27.6 MPa	Disordered	H_2_ and CH_4_
	H_2_ (Buch Single site)^312^				
	CH_4_ (TraPPE-UA)^301^				
Ho *et al.* ^921^	Montmorillonite (CLAYFF)^422,423^	GCMC	323 K / upto 11.15 MPa	n/a	H_2_ and CH_4_
	H_2_ (Darkrim Three-site)^327^				H_2_, CH_4_ and H_2_O
	H_2_O (SPC/E)^346^				
Zhang *et al.* ^889^	Kaolinite – Gib. and Sil. (CLAYFF)^422,423^	GCMC	298.15 K / 0.1–10 MPa	5–200	H_2_ and H_2_O
	H_2_ (Cracknell Two-site)^311^				
	CH_4_ (TraPPE)^925^				H_2_ and CH_4_
	H_2_O (SPC/E)^346^				
Muther *et al.* ^884^	Hydroxylated Quartz (CLAYFF)^422,423^	GCMC	323.15–423.15 K / 1–50 MPa	3	H_2_ and CH_4_
	H_2_ (Darkrim Three-site)^327^				H_2_ and CO_2_
	CH_TraPPE_ ^301^				H_2_ and H_2_O
	CO_2_ (EPM2)^419^				
Zhao *et al.* ^886^	Kaolinite (UFF)^399^	GCMC and MD	303.15–353.15 K / 0–18 MPa	0.3–0.4	H_2_
	Illite (UFF)^399^				
	Dolomite (UFF)^399^				
	Quartz (UFF)^399^				
	Calcite (UFF)^399^				
	H_2_ (UFF Two-site)^399^				
Liu *et al.* ^923^	SiO_2_ (PCFF+)^316,406,430^	MD and MD–MC	300 K / 10–30 MPa	0.5–1.4	H_2_
	H_2_ (PCFF+)^316,406,430^				H_2_ and CH_4_
	CH_4_ (PCFF+)^316,406,430^				H_2_ and H_2_O
	H_2_O (PCFF+)^316,406,430^				H_2_, CH_4_ and H_2_O
Li *et al.* ^329^	Kaolinite^422,423^	MD	333.15 K / 30 MPa	0.6	H_2_, CH_4_, and H_2_O
	H_2_ (Three-site Darkrim)^327^				H_2_, CO_2_, and H_2_O
	CH_4_ (TraPPE)^925^				
	CO_2_ (TraPPE)^678^				
Kahzadvand *et al.* ^882^	Montmorillonite and Illite (CLAYFF)^422,423^	MD	333 K / 15 MPa	0.4–7	H_2_ and CH_4_
	H_2_ (Three-site Marx)^328^				H_2_ and CO_2_
	H_2_O (SPC/E)^346^				
Bui *et al.* ^895^	Kaolinite – Gib. and Siloxane (CLAYFF)^422,423^	MD	298 K / 10 and 20 MPa	1 and 2	H_2_ and H_2_O
	H_2_ (Single-site Buch)^312^				
	H_2_O (SPC/E)^346^				
Zheng *et al.* ^878^	Calcite (Xiao)^431^	MD	298–350 K / 1–10 MPa	2 and 20	H_2_
	Hematite (modified CLAYFF)^424^				
	Quartz (CLAYFF)^422^				
	H_2_ (Three-site Alavi)^336^				
Xie *et al.* ^887^	Kaolinite^423^ and Kerogen (CVFF)^426^	MD	333.15 K / 30–15–10 MPa	2 and 5	H_2_ and CH_4_
	H_2_ ^313^				
	CH_4_ (TraPPE)^301^				
Zhang *et al.* ^881^	Kerogen II-D (CVFF)^426^	GCMC	333.15 K / 3–18 MPa	2 and 5	H_2_
	Montmorillonite (CLAYFF)^422,423^				H_2_ and CH_4_
	H_2_ (Single-site Buch)^312^				
	CH_4_ (TraPPE)^301^				H_2_ and CO_2_
	CO_2_ (Fully Flexible)^926^				
Muther and Dahagi^883^	Kaolinite (CLAYFF)^422,423^	GCMC	323–403 K / 1–50 MPa	3	H_2_
	H_2_ (Three-site Darkrim)^327^				H_2_ and CH_4_
	CH_4_ (TraPPE)^301^				H_2_ and CO_2_
	CO_2_ (EPM2)^419^				
Chen et al.^885^	Quartz (CLAYFF)^422,423^	GCMC and MD	339–400 K / 1–50 MPa	2, 4, and 10	H_2_ and CH_4_
	H_2_ (Single-site Michels)^924^				H_2_, CH_4_, and H_2_O
	CH_4_ (TraPPE-UA)^301^				
	H_2_O (TIP4P)^300^				
Shang et al.^877^	Kaolinite : Gib. and Sil. (CLAYFF)^422,423^	GCMC and MD	303–423 K / 1–30 MPa	1–20	H_2_
	H_2_ (Three-site Darkrim)^327^				
Zhang et al.^896^	Kaolinite (CLAYFF)^422,423^	GCMC	318.15 K / 0.1–30 MPa	0.5–2	H_2_ and H_2_O
	H_2_ (Two-site Cracknell)^311^				CH_4_ and H_2_O
	CH_4_ (TraPPE)^925^				H_2_, CH_4_, and H_2_O
Choudhary and Ho^898^	Pyrophyllite, Gibbsite (CLAYFF)^422,423^	MD	300 K / 0.1 MPa	1.326 and 1.626	H_2_ and H_2_O
	H_2_ (Three-site Darkrim)^327^				CO_2_ and H_2_O
	CO_2_ (TraPPE)^678^				
	H_2_O (SPC/E)^346^				
Babaei et al.^888^	Kerogen (CVFF)^426^	GCMC and MD	363.15 K / 0.1–50 MPa	Disordered	H_2_
	H_2_ (Single-site Buch)^312^				CH_4_
	CH_4_ (TraPPE-UA)^301^				H_2_ and CH_4_
Muther and Dahagi^922^	Na-Montmorillonite (CLAYFF)^422,423^	GCMC and MD	298-403 K / 1–50 MPa	1.26–1.55	H_2_
	H_2_ (Three-site Darkrim)^327^				H_2_, CO_2_, and H_2_O
	CO_2_ (Cygan Flexible)^927^				H_2_, CH_4_, and H_2_O
	CH_4_ (TraPPE-UA)^301^				
	H_2_O (Not mentioned)				
Choudhary and Ho^897^	Kaolinite : Gib. and Sil. (CLAYFF)^422,423^	MD	300 K / 5.1–30.4 MPa	n/a	H_2_, CO_2_ and Octane
	H_2_ (Two-site IFF)^313^				
	CO_2_ (TraPPE)^678^				
	C_8_H_18_ (OPLS all-atom)^428^				
Ho et al.^894^	MCM-41 Silica (Customized)^429^	GCMC	298 K / 0–1 MPa	3.4 (Cylindrical)	H_2_ and OMCTS
	H_2_ (Single-site)^894,928^				CO_2_ and OMCTS
	CO_2_ (EPM)^419^				
	OMCTS (Customized)^928^				
Liu et al.^906^	MMT (CLAYFF)^422,423^	MD	333-413 K / 2–30 MPa	3-40	H_2_
	H_2_ (Single-site Hirschfelder)^321^				H_2_ and H_2_O
	H_2_O (SPC/E)^346^				
Mashhadzadeh et al.^899^	Kaolinite (INTERFACE)^425^	MD	310–410 K / 30 MPa	1–10	H_2_ and H_2_O
	H_2_ (Two-site Yang)^325^				H_2_ and NaCl Brine
	H_2_O (SPC/E)^346^				H_2_ and MgCl_2_ Brine
	Na^+^,^371^ Cl^–^, Mg^2+^ ^929^ [1–5 M]				
Hubao et al.^907^	Calcium Silicate Hydrate (CLAYFF)^425^	MD and GCMC	300–370 K / 2.5–40 MPa	2.5–40	H_2_
	H_2_ (Single-site Hirschfelder)^321^				
Raza et al.^908^	Kerogen (PCFF++)^427^	MD and GCMC	360 K / 3–41 MPa	0.5 and 2	H_2_
	H_2_ (Single-site Waldman)^930^				
Kim et al.^909^	Illite (CLAYFF)^422,423^ and Kerogen (CVFF)^426^	MD	326 K / 30.4 MPa	n/a	H_2_, CH_4_, and H_2_O
	H_2_ (Two-site Wang)^313^				H_2_, CH_4_, CO_2_, and H_2_O
	CH_4_ (OPLS)^428^				
	CO_2_ (Not mentioned)				
	H_2_O (SPC/E)^346^				
Ghasemi et al.^910^	Pyrophillite (CLAYFF)^422,423^	MD	368.15 K / 30 MPa	1–8	H_2_ and H_2_O
	Montmorillonite (CLAYFF)^422,423^				
	Beidellite (CLAYFF)^422,423^				
	H_2_ (Two-site Yang)^325^				
	H_2_O (SPC/E)^346^				
Li et al.^931^	Calcite^432^	MD	353 K / 20 MPa	8	H_2_ and H_2_O
	H_2_ (Single-site Hirschfelder)^321^				
	H_2_O (SPC/E)^346^				
	Na^+^, Cl^–^ ^374^				
	Mg^2+^,^932^ SO_4_^–2^ ^b^ ^933^				

a Gibbsite surfaces have a -OH group : Hydrophilic

b Siloxane surfaces have an Si-O-Si group : Hydrophobic

#### 4.7.1 Competitive Adsorption on Organic and Inorganic Substrates

For applications pertaining to subsurface storage like UHS, H_2_ must stick to rock surfaces effectively but remain easy to retrieve, often competing with cushion gases like CH_4_ and CO_2_. We review articles studying the adsorption of gas mixtures in organic and inorganic nano-pores. Ho et al.^880^ performed molecular simulations to study the adsorption and diffusion of H_2_ and CH_4_ in shale systems, with a particular focus on kerogen. The study demonstrates that CH_4_ exhibits a substantially higher excess adsorption than H_2_ over the entire pressure range, with the total CH_4_ uptake reaching approximately 3.3 mmol/g at a CH_4_ pressure of ca. 100 bar. Moreover, the computed interaction energies indicate that kerogen has a much stronger affinity for CH_4_ (with a minimum interaction energy of ca. -4.3 kcal/mol) compared to H_2_ (ca. -1.8 kcal/mol), which explains the preferential adsorption of CH_4_. When simulating a competitive 1:1 CH_4_:H_2_ mixture, the kerogen selectivity for CH_4_ is found to be more than 10 to 25 times higher than that for H_2_, corroborating existing literature and experimental insights. The simulations further reveal that under conditions of low residual CH_4_ partial pressure, up to 30–35% of adsorbed CH_4_ desorbs upon H_2_ injection. The overall H_2_ uptake in kerogen is of the same order of magnitude as that observed in coal, reinforcing the broader relevance of the results. The MD simulations also predicted that the diffusion coefficient of H_2_ in the kerogen matrix is approximately 20–40 × 10^–9^ m^2^/s, which is approximately one order of magnitude higher than that for CH_4_ and CO_2_, in agreement with comparative studies. These simulation results effectively complement their NMR experiments, which have independently observed distinct signatures for free versus adsorbed H_2_ in shale. Overall, the study provides a molecular-level understanding of the competitive adsorption phenomena, highlighting that the dominance of CH_4_-kerogen interactions must be considered when designing H_2_ geological storage projects.

Zhang et al.^881^ performed GCMC simulations to explore H_2_ adsorption in shale nano-pores for both organic (kerogen) and inorganic (montmorillonite, MMT) substrates at 333.15 K and pressures up to 180 bar, finding that pure H_2_ exhibits nearly twice the excess adsorption on kerogen relative to MMT (yielding a higher absolute storage capacity) while recovery efficiency – defined as the percentage of gas retrieved when pressure is reduced from 180 bar to 3 bar – remains similar between the substrates. In competitive adsorption tests with equimolar mixtures of CH_4_ and H_2_, CH_4_ preferentially adsorbs on kerogen (with selectivity values above one) and, although CH_4_ still adsorbs more strongly in MMT, overlapping adsorption layers result in a smaller competitive difference; additionally, the presence of cushion gases dramatically reduces H_2_ storage capacity (by about 50% in 5 nm pores and up to 80% in 2 nm pores) due to H_2_ dilution and competitive displacement, with the effect intensifying as pore size decreases and CO_2_ proving more effective than CH_4_ (yielding CO_2_/H_2_ selectivity values up to 18 in 2 nm pores), thereby underscoring how pore size, substrate type, and specific gas interactions govern adsorption and recovery, which is critical for optimizing UHS processes.

Kahzadvand et al.^882^ investigated the partitioning behavior of a H_2_ and cushion gas (CO_2_ or CH_4_) mixture within clay mineral pores to assess caprock integrity during UHS. Using advanced non-equilibrium molecular simulations at 333 K and 150 bar, this study provided molecular-level insights by using constant reservoir composition MD (CRC-MD) which dynamically maintains fixed fluid compositions in designated regions, thereby overcoming limitations such as feed depletion and composition control seen in traditional methods. The results reveal that pores narrower than 0.5 nm are impermeable to both H_2_ and cushion gases, while H_2_, owing to its superior rotational degrees of freedom, begins to penetrate at pore widths between 0.5 and 0.6 nm, with a notable increase in number density observed at these dimensions. In contrast, cushion gases, particularly CO_2_, exhibit a stronger affinity for the clay surfaces and edges, dominating pore occupancy in interlayer spaces up to approximately 2 nm. Quantitatively, the simulations show that in montmorillonite and illite pores, the mole fraction of CO_2_ initially exceeds that of H_2_ at 0.6 nm for CO_2_/H_2_ mixtures, with parity reached only at larger pore sizes (around 5 nm in montmorillonite and 7 nm in illite). For H_2_/CH_4_ systems, CH_4_ fails to penetrate below 0.6 nm, highlighting a selective permeation linked to molecular size, while at 1 nm, CH_4_ occupancy peaks with relative ratios of about 0.6:0.4 compared to H_2_, before declining at larger pore sizes. The study underscores that the effectiveness of cushion gas in inhibiting H_2_ leakage is strongly dependent on pore thickness and clay surface charge, with CO_2_ showing higher adsorption owing to electrostatic interactions with negatively charged sites and exchangeable cations. Furthermore, the detailed analysis via 2D density maps illustrates that gas molecules accumulate preferentially in the mid-interlayer region and near the clay edges, which correlates well with experimental observations and prior simulation studies. New physics uncovered include the dynamic interplay between rotational freedom of H_2_ and the surface affinity of cushion gases, leading to qualitatively different partitioning behaviors in nano-pores (pore sizes < 2 nm) versus meso-pores (pore sizes > 2 nm). The CRC-MD approach thus not only clarifies the penetration thresholds (e.g., 0.5, 0.6, and 2 nm) but also reveals that for pore sizes larger than 4 nm, the gas composition approaches bulk behavior. Overall, this work provides a comprehensive molecular explanation for the dual role of cushion gases as both pressure maintenance agents and leakage barriers in H_2_ geo-storage applications.

Muther and Dahagi^883^ performed a study of H_2_ adsorption on kaolinite using GCMC simulations to elucidate adsorption mechanisms under varying geological conditions. The study validates its simulation setup by comparing the computed CO_2_ adsorption isotherms with experimental measurements taken at 298.15 K and pressures ranging from 0.2 to 1 bar, thereby establishing a robust basis for subsequent simulations. The results show that under high-pressure (10–500 bar) and elevated temperature (323–403 K) conditions, pure H_2_ adsorption on kaolinite ranges from 0.0494 to 2.237 mol/kg; specifically, H_2_ uptake increases with pressure and decreases with temperature. When H_2_ is mixed with CO_2_ or CH_4_, the simulations reveal that introducing 10–50 mol% of these gases significantly reduces H_2_ uptake because the available adsorption sites on the kaolinite are increasingly occupied by CO_2_ or CH_4_ molecules. In particular, the simulations indicate that, when present, CO_2_ displaces H_2_ more effectively than CH_4_–as evidenced by lower H_2_ density peaks-suggesting that the kaolinite surface exhibits a stronger affinity for CO_2_. Furthermore, the RDFs confirm that among the atoms in the kaolinite structure, silicon atoms contribute slightly more to the H_2_ adsorption process than do H_2_, oxygen, or aluminum atoms. Overall, the quantitative insights obtained-such as the observed linear decrease in the Henry coefficient with increasing temperature-are in good agreement with previous simulation and experimental studies on gas adsorption in clay minerals, and can help guide strategies for underground H_2_ storage. The study also demonstrates that the desorption curves closely mirror the adsorption behavior, confirming efficient H_2_ recovery without hysteresis effects.

Muther and Dahaghi^884^ performed GCMC simulations to compute the adsorption of H_2_ on hydroxylated quartz (i.e., a representative geological mineral) across a wide range of pressures and temperatures. The study utilizes a molecular-scale quartz structure that has been hydroxylated to mimic natural subsurface conditions. The simulations explore the response of H_2_ in both pure and mixed gas systems. The results show that H_2_ uptake increases linearly with pressure, while high temperatures (up to 403 K) reduce adsorption since elevated temperatures weaken the mineral’s affinity for H_2_ by increasing the kinetic energy of the molecules, reducing the stability of H_2_ retention on the quartz surface. To quantify the mineral’s affinity for H_2_, Henry coefficients were calculated, which were shown to consistently decrease with rising temperature, indicating that adsorption is less favorable when kinetic energy is higher; this decline is accompanied by higher binding energies between H_2_ and the quartz surface, reinforcing the observation that elevated temperatures reduce adsorption effectiveness. In mixed gas systems, the presence of CO_2_ and CH_4_ further displaces H_2_, with H_2_ uptake reduced to as low as 1.28 mol/kg in a 50:50 gas mixture, mirroring trends observed in other porous materials such as MOFs and zeolites, and the addition of a thin water film (ca. 1 wt%) further reduces H_2_ adsorption by ca. 3% due to water molecules preferentially occupying the adsorption sites on the hydroxylated quartz surface, highlighting the crucial role of surface hydroxyl groups in mediating gas interactions. Overall, the study emphasizes that both conditions (i.e., pressure and temperature) and compositional factors (e.g., presence of CO_2_, CH_4_, and water) are critical in controlling H_2_ adsorption on hydroxylated quartz, with the interplay between these factors ultimately determining the storage capacity and stability of H_2_ geo-storage systems. Similarly, Chen et al.^885^ used GCMC simulations to study the adsorption of CH_4_–H_2_ mixtures under conditions representative of shale gas reservoirs. A base-case scenario was set in a 4-nm slit pore at 370 K and 300 bar (in the paper, units of Fahrenheit and psi were used), while simulation conditions spanned temperatures of 339 - 400 K, and pressures up to 500 bar. It is important to note that experimental data are available only for pure CH_4_ adsorption; the computed excess adsorption capacities and adsorbed phase densities CH_4_ agree with these experiments within a deviation of 3%. The outcomes revealed that when the mole fraction of CH_4_ is high (≥50%), CH_4_ is preferentially adsorbed on both graphite and quartz surfaces, forming an adsorption layer close to the solid boundary, while H_2_ is predominantly distributed in the free phase within the pore. However, when the mole fraction of CH_4_ is low (e.g., 20%), the behavior changes: in the graphite layer, the fewer CH_4_ molecules present leave sufficient space for H_2_ to form its own adsorption layer at the surface. A similar trend is observed for the quartz surface, although the density of the H_2_ adsorption layer is notably lower compared to that in the graphite system. When water is present in the system (modeled with TIP4P), water molecules strongly interact with the solid surfaces, particularly with the carbon atoms of the graphite layer and the hydroxylated (100) face of quartz, which further suppresses CH_4_ adsorption. This suppression reinforces the free distribution of H_2_, with water forming its own distinct adsorption layer and having small direct effect on the behavior of H_2_ molecules.

Zhao et al.^886^ evaluated the feasibility of using depleted shale gas reservoirs for UHS by combining experiments with molecular simulations. A major finding was that H_2_ adsorption is well described by the Langmuir isotherm over temperatures ranging from 303.15 K to 353.15 K and pressures up to 180 bar, which indicates that only a single layer of H_2_ molecules is adsorbed on the mineral surfaces. It is noteworthy that the Langmuir fit was applied separately to experimental excess adsorption isotherms as well as simulation data. Both sets of results are in the same order of magnitude, however, slight deviations arise due to the single pore size assumption in the simulations and impurities present in the mineral samples used in the experiments. Quantitatively, the adsorption capacity decreases in the order of kaolinite > illite > calcite > dolomite > quartz. Both experimental measurements and simulations confirm that clay minerals (kaolinite and illite) exhibit capacities approximately two to three times higher than those of common minerals such as calcite, dolomite, and quartz. The overall agreement between experiments and simulations strengthens the comparative framework; yet, any minor discrepancies likely stem from differences in pore size distributions in natural samples versus the idealized models employed in simulations. Furthermore, the computed heat of adsorption was in the range of ca. 9–26 kJ/mol. This range, which lies well below the threshold for chemical adsorption, confirms that the process is dominated by physisorption. Additionally, H_2_ diffusion coefficients were computed from MD simulations. It was shown that H_2_ has the lowest diffusion coefficient in illite (ca. 0.018 × 10^–9^ m^2^/s) and the highest in dolomite (ca. 25.83 × 10^–9^ m^2^/s). This variation is attributed to the hindering effect of interlayer cations in illite and a relatively more open nano-pore structure in dolomite, which facilitates faster molecular movement. Overall, while the qualitative agreement between experiment and simulation confirms the potential of shale inorganic minerals for H_2_ storage, the significant quantitative deviations emphasize the need for further refinement in pore structure modeling, and a more detailed study of additional factors (e.g., organic matter, residual gases, water).

In a similar study, Xie et al.^887^ investigated the feasibility of UHS in depleted shale gas reservoirs by performing MD simulations that capture the complete huff-n-puff process. In that work, the simulation conditions mimic reservoir settings (333.15 K and pressures varying from 300 down to 100 bar). The simulations were designed to mimic a reservoir’s operational cycle by first reducing pressure from 300 to 150 bar to trigger CH_4_ desorption (the *huff* stage), then injecting H_2_ until a desired composition is achieved, and finally further lowering the pressure to 100 bar (the *puff* stage) to mobilize the stored H_2_. Recovery efficiency is quantified by comparing the computed H_2_ densities before and after the final pressure drop. This cyclic injection and production strategy is designed to maximize storage efficiency while enabling later retrieval of H_2_. The study compares two types of nano-pore models. The organic nano-pores are modeled using a type II-D kerogen representation derived from previous studies (i.e., a realistic representation of organic matter in shale), while the inorganic nano-pores are represented by kaolinite-based models of clay minerals. The adsorption capacities in these nano-pores are quantified by analyzing the ensemble-averaged density profiles and the distribution of gas molecules (both H_2_ and CH_4_) along the pore dimensions. In practice, the adsorption capacity is measured in terms of apparent adsorption isotherms and the average density computed from simulation snapshots at equilibrium. Furthermore, the simulations revealed that for the reservoir conditions studied, the organic pores show a unique density profile for H_2_ where the first adsorption peak is lower than the second. This behavior is opposite in inorganic pores, which can be attributed to competitive adsorption with CH_4_, which strongly adheres to the organic surface. Density profile analysis clearly corroborated these findings across the different pressure points studied. The simulation results were further validated by comparing the computed bulk densities of pure and binary gas mixtures with NIST data. In the huff-n-puff cycle, recovery efficiency was determined by monitoring the change in the average density of H_2_ before and after the puff stage; for example, recovery efficiencies were found to be 49.9% in organic meso-pores and 40.0% in organic micro-pores, in contrast with higher recovery values in inorganic pores (70.0% in meso-pores and 63.3% in micr-opores). These percentages are calculated as the ratio of the recovered H_2_ (measured as the drop in average density) to the initial injected H_2_ density.

Babaei et al.^888^ followed a hybrid GCMC/MD approach at 363.15 K and pressures up to 500 bar to investigate the adsorption and diffusion of H_2_, CH_4_, and their mixtures in type II-D kerogen, wherein realistic pore size distributions are generated through the incorporation of dummy particles. In this framework, the inherent heterogeneity of the kerogen matrix is captured via three distinct models (Model 1, Model 2, and Model 3) constructed by embedding dummy particles with diameters of 0 nm, 1 nm, and 2 nm, respectively, to represent incremental variations in pore structure. Two computational frameworks were established to quantify adsorption-induced deformation. The ”Lagrangian approach” referenced the system to its pre-adsorption state under fixed thermodynamic conditions, maintaining a constant accessible volume as a baseline, whereas the ”Eulerian approach” was used to compare the system with and without adsorption under identical mechanical conditions, rendering the pore structure pressure dependent and more sensitive to deformation at high pressures. This detailed representation was critical as variations in pore size not only affect the accessibility of adsorption sites but also determine the extent of swelling induced by gas adsorption, a key factor influencing both the storage capacity and mechanical integrity of the matrix. In the case of CH_4_ adsorption, the results indicated that the flexible kerogen models exhibited 1.4, 1.6, and 1.3 times higher adsorption in Models 1, 2, and 3, respectively, compared to their rigid counterparts. The corresponding volumetric strain (a measure of matrix expansion) ranged from 3.25% to 4.54% using the Lagrangian approach, and from 2.97% to 5.92% with the Eulerian approach at high pressures. For H_2_ adsorption, however, the kerogen showed negligible deformation overall with the matrix experiencing either slight swelling or even contraction depending on the pore size distribution, an outcome attributed to the H_2_’s small molecular size and weak adsorption affinity, combined with capillary effects and pore geometry variations. Moreover, the study revealed that H_2_ diffuses roughly one order of magnitude faster than CH_4_ (6.87 × 10^–9^ m^2^/s for H_2_ compared to 0.30 × 10^–9^ m^2^/s for CH_4_ at 5 MPa). Although CH_4_ was preferentially adsorbed, as evidenced by a selectivity greater than 1 across all pressures, this selectivity decreases with increasing pressure due to the saturation of adsorption sites, thereby reducing the differences between the two gases. Zhang et al.^889^ used GCMC simulations to investigate the adsorption behavior of H_2_/CH_4_ mixtures in two types of kaolinite nano-pores, namely, the hydrophilic gibbsite and the hydrophobic siloxane pores, with pore sizes ranging from 5 to 200 nm, at 298.15 K and pressures between 1 and 100 bar. Under dry conditions, the study demonstrated that increasing the bulk H_2_ mole fraction leads to an increased storage density for H_2_, with its adsorption varying linearly with pressure, and a corresponding decrease in CH_4_ density. Notably, when the injected mixture contained less than 20% H_2_, the adsorption process selectively favors H_2_. For instance, an injection mixture of 5% H_2_ and 95% CH_4_ yielded a H_2_/CH_4_ selectivity of approximately 2. Although both nano-pore types exhibit enhanced H_2_ selectivity under low H_2_ injection fractions, the surface chemistry plays a critical role: in gibbsite pores, strong interactions between CH_4_ molecules and the pore surface result in higher CH_4_ densities at low H_2_ fractions, whereas in siloxane pores the overall CH_4_ capacity is slightly lower, thereby improving the relative selectivity for H_2_. In terms of pore size effects, the absolute density of H_2_ remains nearly independent of the pore size because most stored H_2_ is concentrated in the middle of the pore; however, the relative selectivity, defined as the ratio of the adsorbed H_2_ to CH_4_, increases in larger pores (particularly those exceeding 100 nm) due to improved enrichment of H_2_. The presence of moisture reduces the adsorption densities of both gases in the mixed-gas scenario. In hydrophilic (gibbsite) nano-pores, water molecules are strongly attracted to the surface via hydrogen-bonding, forming a thin film along the walls, whereas in hydrophobic (siloxane) pores, water tends to form clusters that span across the pore, reducing the available free volume. Under moist conditions, for example at a water concentration of 0.2 g/cm^3^ and a bulk H_2_ mole fraction of 20%, the gibbsite pores are more favorable for H_2_ storage than their siloxane counterparts. Although both pore types exhibit reduced overall adsorption densities in the presence of water, the effective storage volume in siloxane pores is more significantly diminished due to the water clusters, which adversely affect the selective adsorption of H_2_.

A cross-comparison of these studies reveals several common quantitative trends. Muther and Dahaghi^883,884^ reported that with increasing temperature, H_2_ molecules possess greater kinetic energy, which they argued weakens attractive interactions with the adsorbent surface, thereby reducing adsorption capacity. While this reflects the interpretation of these authors, a more physically consistent explanation is that the release of H_2_ at higher temperatures arises from the increasing dominance of entropic effects, rather than from the weakening of enthalpic attractions. It should also be noted that GCMC simulations, by construction, do not capture kinetic energy and kinetic effects. Thus, the observed reduction in adsorption with temperature in GCMC results should not be attributed to the reduced molecular kinetic energy of H_2_ molecules.

Shifting our focus on the adsorption of CH_4_/H_2_ and CO_2_/H_2_ on organic and inorganic pores, we compiled Fig. 50 which shows selectivities for these mixtures collected from different simulation studies. Given the potential sensitivity of selectivities to pore size^890^ and temperature, the values are selected such that the data can be compared without significant biases introduced from varying thermodynamic conditions. From Fig. 50(a) and (b), it is clear that the selectivity of CH_4_ over H_2_ remains above one in both organic and inorganic pores. However, the magnitude of this selectivity differs substantially: organic nano-pores (e.g., kerogen) often exhibit values an order of magnitude larger than their inorganic counterparts. Organic nano-pores show higher CH_4_ selectivity because their heterogeneous surfaces provide stronger van der Waals interactions, promoting the formation of saturated, well-defined CH_4_ adsorption layers that preclude extensive H_2_ binding.^881,891^ The stronger van der Waals interactions between CH_4_ and carbon, compared to H_2_ and carbon, arise from differences in the LJ potential parameters, specifically the well-depth. For instance, Kumar et al.^892^ illustrated these differences clearly in their interaction profiles (Figure 2 in ref 892), showing that the well-depth for CH_4_-carbon interactions is approximately eight times deeper than that for H_2_-carbon interactions. This deeper potential well for CH_4_ reflects its larger molecular size and greater polarizability,^893^ both of which enhance dispersion forces relative to H_2_. In contrast, inorganic minerals, with their lower CH_4_ affinity and more uniform surfaces, result in less saturation and greater availability of sites for H_2_, thus, reducing CH_4_ selectivity.

**Figure 50:**
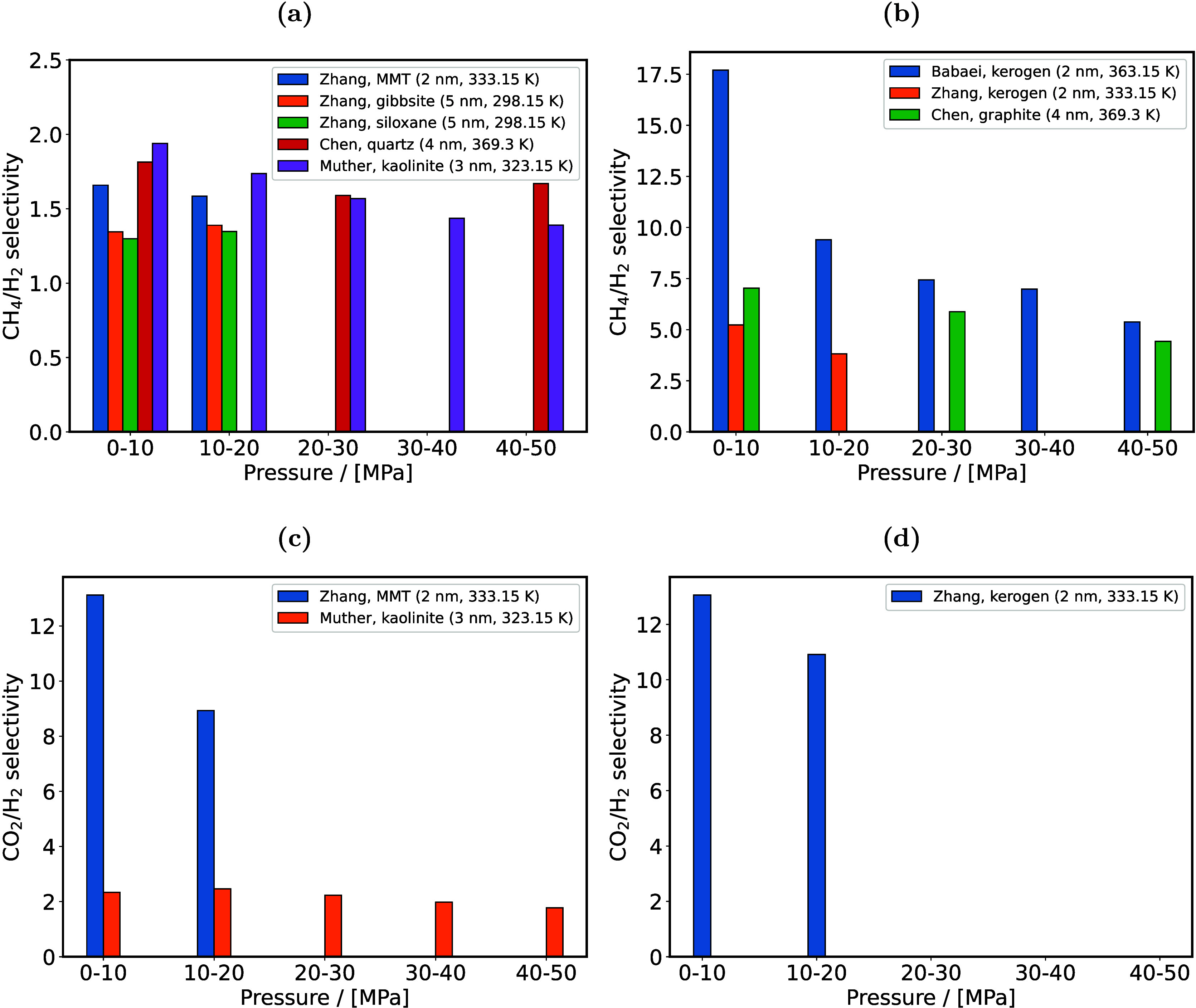
Comparison of CH_4_/H_2_ selectivities gathered from the literature for (a) inorganic and (b) organic pores. Comparison of CO_2_/H_2_ selectivities gathered from the literature for (c) inorganic and (d) organic pores. The data are taken from Zhang et al.,^881^ Chen et al.,^885^ Babaei et al.,^888^ and Muther and Dahagi.^883^ Each bar represents the mean selectivity within pressure bins of 10 MPa (0-10, 10-20, 20-30, 30-40, 40-50 MPa), calculated from data points in the corresponding pressure range. The legend indicates the study and, where applicable, the pore width (e.g., 2 nm) and temperature. Pressure values were converted from psi to MPa where necessary, and selectivity was inverted for studies where H_2_/gas was originally reported. This figure illustrates the variation in selectivity across different geological materials and gas mixtures, highlighting the impact of pore type and pressure on adsorption behavior.

As shown in Fig. 50(a) and (c), *S*_CO_2_/H_2__ selectivities are significantly higher than *S*_CH_4_/H_2__ indicating a strong preferential adsorption of CO_2_ over CH_4_ ^890^ in inorganic pores. This strong adsorption can be attributed to the strong quadrupole moment and polarizability^890^ of CO_2_. This substantial preference for CO_2_ compared to CH_4_/H_2_ systems can have significant practical implications. For instance, in underground storage, the intentional use of CO_2_ as a cushion gas would result in its retention on pore surfaces, leaving H_2_ predominantly in the free (mobile) phase, which is advantageous for recovery and purity. For organic pores, the comparison between Fig. 50(b) and (d) suggests that *S*_CO_2_/H_2__ reported by Zhang et al.^881^ generally exceeds *S*_CH_4_/H_2__, except in the data from Babaei et al.,^888^ who studied adsorption in disordered pores, leading to a pore size distribution rather than a single pore size (as in slit-pore studies).

The studies reviewed earlier suggest that beyond surface chemistry and cushion gases, pore size, pressure, and temperature all significantly impact gas/H_2_ selectivities. Zhang et al.^890^ demonstrated that *S*_CO_2_/H_2__ in Na-Montmorillonite pores vary by a factor of 3 to 4 when pore size increases from 2 to 5 nm. In sharp contrast, *S*_CH_4_/H_2__ in the disordered kerogen pores studied by Babaei et al.^888^ hardly show any change despite an increase in mean pore size from 0.4 nm (Model 1) to 2 nm (Model 3). The authors attributed this invariance to the particularly strong CH_4_-kerogen interactions overshadowing pore size effects.^888^ Another commonly observed behavior across the reviewed studies is the decrease in selectivities with increasing pressure due to the saturation of binding sites. There has not been a systematic investigation of the role of temperature in gas selectivity.

The presence of water films typically reduces H_2_ adsorption by 3 to 40%, whereas cushion gases like CO_2_ and CH_4_ form robust adsorption layers, potentially decreasing H_2_ capacity by up to 30%. H_2_ interaction energies in pure systems range from approximately −1.5 to −2.0 kcal/mol, while competing gases exhibit stronger binding (e.g., CH_4_ at −4.3 kcal/mol). Smaller pore sizes show up to 40% higher adsorption, and the maturity of organic content (kerogen) further modulates these effects. Together, these quantitative findings provide a road-map for understanding and optimizing competitive adsorption and storage performance in geological formations.

#### 4.7.2 Solubility of Hydrogen under Confinement

Solubility under confinement is a crucial topic with direct implications for UHS and other applications. As highlighted by Ho et al.,^894^ the confined environment in nano-pores - whether circular, as in some studies, or flat-walled, as in others - not only enhances gas solubility through mechanisms such as cavity formation and solvent layering, but also alters gas diffusion and clustering behavior. The studies focusing on this topic use a variety of molecular models. For example, Ho et al.^894^ studied circular pore systems to mimic realistic confined environments, whereas Bui et al.^895^ used flat-wall, slit-shaped nano-pore models that represent kaolinite or other clay minerals. Such differences in the molecular systems used allow for a rigorous comparison of how pore geometry and surface chemistry control gas uptake phenomena. A detailed information of the thermodynamic conditions, pore sizes, materials, and the force fields used in the different studies are tabulated in Table 18.

Ho et al.^894^ investigated gas uptake in a model meso-porous system where a solvent is confined within well-defined pores, providing a basis for understanding how nano-confinement affects gas-solvent interactions. The paper identifies two distinct mechanisms for over-solubility. For gases that interact strongly with the solid surface (e.g., CO_2_ on silica), there is an adsorption-driven uptake, while for gases with weak gas/solid interactions (such as H_2_), the predominant mechanism is an enhanced solubility due to the formation of low-density regions arising from solvent layering. For instance, the work explains that when only a single layer of solvent is present inside a meso-pore, adsorption at the gas/liquid interface contributes additionally to the gas uptake. Although no numerical values are provided in this study, the mechanism establishes the baseline for comparison with more detailed quantitative studies in later works, such as Bui et al.^895^ The study by Zhang et al.^896^ examined H_2_ dissolution in water-saturated caprock nano-pores mimicking natural sealing layers. Nano-confinement in these pores was shown to enhance H_2_ solubility by up to 27 times relative to bulk water. For example, Zhang et al.^896^ report that in pores with an optimal width of 0.55 nm, the dissolved H_2_ density reaches 5.614 kmol/m^3^ at 318.15 K and 300 bar, corresponding to an enhancement factor of about 27.4 times the bulk value.

In the context of UHS, Bui et al.^895^ studied H_2_ solubility in water confined within kaolinite nano-pores. Two distinct pore widths were considered: 20 Å and 10 Å. For the 20 Å wide pore, the study reports that under pressures of 100 and 200 bar, H_2_ solubility is enhanced by approximately 2.5 times compared to bulk water as shown in Fig. 51. Strikingly, in the 10 Å pore, the solubility is significantly enhanced to nearly 25 times that of the bulk (see Fig. 51). These numbers are supported by detailed water density profiles that reveal pronounced hydration layers; H_2_ preferentially accumulates in regions of low water density near the hydrophobic siloxane surface, whereas the hydrophilic gibbsite side shows a dense water layer that excludes H_2_. The enhancement is attributed to pronounced water density fluctuations that generate regions of low solvent density where H_2_ preferentially dissolves. Interestingly, while the confinement retards water diffusion, H_2_ diffusion is enhanced due to these fluctuations. The study clearly links the molecular-scale structuring of water under confinement to the observed over-solubility of H_2_.

**Figure 51:**
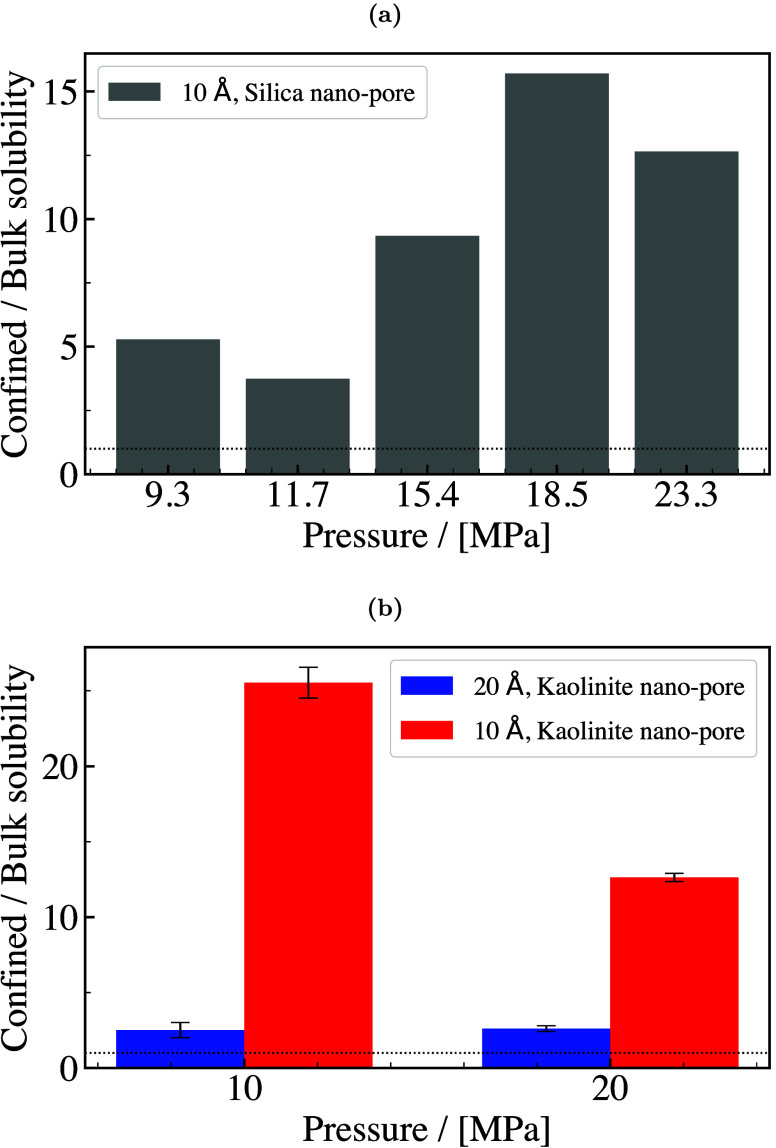
Enhancement of solubility under confinement: Ratio of gas solubility in confined water and bulk water for (a) CH_4_ and (b) H_2_. H_2_ solubilities are taken from Bui et al.^895^ and CH_4_ solubilities are taken from Phan et al.^900^ and Sakamaki et al.^904^ The nano-pores are made of Kaolinite in the study by Bui et al.^895^ (see Table 18) and Silica in Phan et al.^900^ The dotted line serves as a reference where bulk solubility equals confined solubilities.

The work by Choudhary and Ho^897^ explores a hybrid system designed to mimic depleted oil reservoirs, where oil, gas, and kaolinite interact. The study demonstrates that H_2_ dissolves more readily in oil than in water, owing to weaker H_2_-oil interactions compared with H_2_ - water interactions. Quantitatively, the simulation results indicate that the addition of as little as 5% CO_2_ (in mole fraction units) can reduce H_2_ dissolution in oil by approximately 15% because of the resulting decrease in interfacial tension. H_2_ adsorption is weak and similar on both hydrophilic and hydrophobic faces, whereas it is CO_2_ that exhibits a strong preference for the hydrophilic side. These detailed quantitative observations (e.g., the reduction percentage and the role of specific surface terminations) provide a link of the molecular-level interactions with the overall gas uptake behavior. Choudhary and Ho^898^ focused on gas clustering within hydrated nano-pores with contrasting surface chemistries. It shows that over-solubility can emerge either through adsorption at the liquid/solid interface or by gas molecules migrating into low-density regions formed by solvent structuring. For example, in hydrophobic nano-pores (represented by pyrophyllite with siloxane rings), CO_2_ exhibits a much stronger tendency to form stable dimers and larger clusters due to its higher quadrupole moment (14.2 × 10^–40^ C m^2^) compared to H_2_ (1.7 × 10^–40^ C m^2^); as a result, while CO_2_ tends to exit the pore as clusters, H_2_, with its lower polarizability (0.80 × 10^–24^ cm^3^), shows a lesser degree of clustering. Such molecular insights, supported by free energy calculations, are crucial for understanding how nano-pore dimensions and surface chemistry jointly control the aggregation processes within confined media.

The investigation by Mashhadzadeh and Faroughi^899^ used atomistic simulations to study the behavior of dilute H_2_ in water-saturated kaolinite-a mineral representative of clay-rich caprocks. Their results indicate that as the pore size decreases, for example from 100 Å down to 10–15 Å, dense layers of water and H_2_ form near the clay surface. These structured arrangements lead to a reduction of the H_2_ self-diffusion coefficient compared to the bulk phase, even as local H_2_ concentrations are elevated. The study further identifies that the presence of divalent ions (with larger hydration shells), such as Mg^2+^, decreases H_2_ mobility more significantly than monovalent ions. These findings offer useful insights into the interplay between nano-scale confinement, salinity, and molecular diffusion.

All studies discussed in this section converge to the notion that both nano-confinement and surface/interface phenomena, ranging from cavity formation, solvent layering, and density fluctuations to specific interaction strengths, are vital in enhancing H_2_ solubility (or over-solubility) in confined media. Concrete examples, showcasing the particular nature of confined H_2_ are the studies by Zhang et al.,^896^ where it was shown that a pore width of 5.5 Å at 318.15 K and 300 bar yields a H_2_ solubility enhancement of nearly 27 times relative to bulk, and by Bui et al.,^895^ where it was reported that kaolinite nano-pores with a width of 10 Å result in a 25-fold enhancement of hydrogen solubility at pressures of 100–200 bar. In the molecular simulation studies reviewed here, the differences in interfacial behavior were also highlighted, i.e., hydrophobic surfaces such as siloxane and pyrophyllite, foster the formation of gas clusters^898^ and low-density solvent regions, whereas hydrophilic surfaces, such as gibbsite and kaolinite, tend to form dense water layers that limit gas uptake. The study addressing oil–clay–gas interactions by Choudhary and Ho^897^ underscores the role of interfacial tension and mineral surface properties in modulating gas uptake in hybrid systems. It is also important to emphasize that this confinement-induced over-solubility is not unique to H_2_. Similar trends have been reported for other gases, such as CH_4_ and CO_2_, across a range of systems spanning protein–water and gas–water in industrial catalysis. As reported in the literature, CH_4_ solubility in confined water has been reported to be enhanced by up to 50 times relative to its bulk value.^900^ Likewise, CO_2_ over-solubility in confined solvents has been well documented in studies where confinement and the nature of the solid surface (e.g., hydrophobic and hydrophilic terminations) significantly alter the adsorption–desorption equilibrium.^898^

The influence of confinement on gas solubility is not limited to simple gas–solvent systems. Similar mechanisms-often linked to water density fluctuations, cavity formation, and local solvent structuring-also manifest in more complex media, such as those involving protein - water interactions. Reviews on protein hydration, such as the work by Ball,^901^ as well as studies on hydrophobic effects and water density fluctuations discussed by Rego and Patel^902^ and Jamadagni et al.,^903^ provide additional evidence that enhanced gas uptake under nano-confinement is a universal phenomenon, governed by a delicate balance between solvent layering, cavity formation, and specific gas–solid and gas–solvent interaction strengths.

#### 4.7.3 Diffusivity of Hydrogen under Confinement

Understanding mass transport mechanisms of H_2_ in geological environments is essential for evaluating both the feasibility and efficiency for a variety of applications such as UHS. This section reviews recent studies using MD simulations to investigate H_2_ transport in nano-pores inspired by geological systems. In the study by Wang et al.,^905^ EMD simulations were first used to reveal the behavior of fluid mixtures in Na-montmorillonite nano-pores, showing that water preferentially adsorbs onto the clay surfaces forming water films. At a water content of 10%, both H_2_ and CH_4_ form two distinct adsorption layers in smaller pores. As water content increases, the water film thickens by 2–3 times, driving the gas molecules into the bulk region, while the calculated selectivity coefficients for H_2_/CH_4_ (ranging from 0.70 to 0.76) indicate that the cushion gas (CH_4_) effectively reduces H_2_ loss. In contrast, NEMD simulations captured the dynamic transport behavior under applied pressure gradients, where elevated pressures induce the desorption of water molecules near the pore walls, thereby enhancing the accumulation and mobility of H_2_. The self-diffusion coefficient of H_2_ was observed to decrease by up to 53% when water content increases from 10% to 30%, with the ratio of H_2_ to CH_4_ diffusion coefficients decreasing from 2.2 to 1.7. Under high pressure gradients, a notable desorption of water molecules is observed near the pore walls that enhances the accumulation and mobility of H_2_ in these regions, a phenomenon that is consistent with recent experimental trends in enhanced multiphase flow through nanoporous media.

The study by Oliver et al.^879^ reported MD simulations of H_2_ transport in nanoporous media, focusing on both organic (graphene) and inorganic (kaolinite) slit pores under subsurface conditions. The authors investigated how H_2_ diffusion is influenced by surface chemistry and pore size (ranging from 2 to 20 nm), and pressure (from 20 to 500 bar). Notably, H_2_ adsorption occured as a monolayer (3 Å thick) on both materials, though graphene exhibited ca. 35% higher surface density than kaolinite. Despite the stronger gas–solid interaction in the case of graphene, the self-diffusion coefficients (in the order of 10^–6^ m^2^/s) were higher in graphene than in kaolinite at low pressures. This finding, supported also by the density profiles, collision times (with graphene’s collision time being approximately 2 ps longer at 20 atm), and lateral velocity autocorrelation functions that reveal more frequent wall-mediated collisions on the rougher kaolinite surface. Both materials demonstrated an exponential decrease in H_2_ self-diffusion coefficients with increasing pressure. However, at high pressures (beyond 50-100 atm), the impact of surface chemistry diminishes as gas–gas collisions begin to dominate, leading their transport properties to converge.

Zheng et al.^878^ studied the mechanisms of H_2_ diffusion in geological slit pores made of calcite, hematite, and quartz, focusing on the effects of temperature, pressure, pore size (confinement), and surface chemistry. The study demonstrated that for all three minerals, the H_2_ self–diffusion coefficient increases with temperature in the range of 298 to 350 K, and in 20 nm slit pores at 100 bar the diffusion follows a bulk regime with the relation *D* ∝ *T* ^1.5^ (supported by linear trends in the ln–ln plots with slopes equal to ca. 1.5). In contrast, increasing the pressure from 10 to 100 bar, consistently reduces the diffusion coefficient across all minerals, following a power law with fitted exponents ranging from −0.825 to −0.964, which is corroborated by analyses of normalized interaction energies and density profiles showing enhanced adsorption at higher pressures. When the pore aperture is reduced from 20 nm to 2 nm, the diffusion coefficient decreases substantially due to stronger wall-gas interactions, resulting in a shift from bulk to transition or even surface diffusion regimes. The study showed that surface roughness enhances H_2_ diffusion in larger pores by effectively enlarging the pore space while it decreases diffusion in smaller pores due to stronger adsorption. These conclusions were corroborated by comparisons of decay times from velocity autocorrelation functions and 2D density distributions. In a related work, Shang et al.^877^ performed simulations to compute the H_2_ diffusion and adsorption in confined kaolinite slits using a hybrid MD–GCMC approach. Similarly to the studies reviewed so far, the focus of this work was also on how variations in pore size, temperature, pressure, and surface mineralogy affect H_2_ properties. This study showed that when the pore size exceeds 5 nm, H_2_ is predominantly located in the bulk phase with minimal adsorption losses, whereas for pores below 5 nm, a rapid decrease in both excess adsorption and diffusion coefficient due to enhanced gas–solid interactions is observed. Increasing temperature from 303 to 423 K reduced the adsorption capacity but concomitantly increased the H_2_ diffusion coefficient, following a power law relationship, with corresponding changes in both the density profiles and the gas–solid interaction energy. Meanwhile, elevating pressure from 10 to 300 bar enhanced the storage capacity while diminishing the mobility of H_2_, as supported by the observed rise in adsorption peak densities and the inverse trend in diffusion coefficients. In terms of surface chemistry, the study by Shang et al.^877^ revealed that the positively charged gibbsite surface promotes H_2_ adsorption compared to the negatively charged siloxane surface.

Liu et al.^906^ conducted MD simulations to investigate the diffusion of H_2_ confined in montmorillonite nano-pores, with the motivation being assessing its leakage risk in geological storage settings. The study systematically examined the dependence of the self–diffusion coefficient (which were shown to be on the order of 10^–8^ m^2^/s) on temperature (333–413 K), pressure (20–300 bar), and pore size (with slit apertures ranging from 3 to 40 nm). It was observed that diffusivity increases moderately with temperature and pore size, yet decreases with higher pressures. For example, in an 8 nm slit, the diffusivity increased from approximately 8.08 × 10^–8^ m^2^/s at 353 K to 10.03 × 10^–8^ m^2^/s at 413 K, while an increase in the pressure from 2 to 300 bar led to a reduction from nearly 10 × 10^–8^ m^2^/s to 7.5 × 10^–8^ m^2^/s. The authors further demonstrated anisotropic diffusion by resolving local diffusion coefficients into components parallel and perpendicular to the clay surfaces, with density profile analyses revealing a single adsorption layer at ca. 2.8 Å from the surface. In addition, trends in H_2_ diffusion under varying moisture content and brine salinity were explored. As the average water density increases in a 3 nm slit from 0.568 to 0.758 g/cm^3^, the self–diffusion coefficient steeply decreases from 8.27 × 10^–8^ to 4.25 × 10^–8^ m^2^/s. While variations in salinity up to 8 wt% yield minor changes, at 12 wt% the diffusivity drops further (e.g., from 8.67 × 10^–8^ to 7.21 × 10^–8^ m^2^/s) due to the formation of brine droplets that hinder gas motion. Although similar trends in diffusion modulation have been reported for other fluids such as CH_4_, CO_2_, octane, and nonadecane, the study implied that the relatively moderate fluid–solid interactions of H_2_ render its pore–size sensitivity less pronounced than that of the other materials.

Using MD simulations, Mashhadzadeh and Faroughi^899^ computed H_2_ diffusion in water-saturated kaolinite nano-pores under conditions relevant to UHS. The study explored how variations in pore size (ranging from 10 to 100 Å), temperature (from 310 to 410 K), and ionic composition (salinities of 1 to 5 M, and the use of monovalent Na^+^ versus divalent Mg^2+^ ions) affect the diffusion of H_2_ under a fixed pressure of 300 bar. It was shown that reducing pore size leads to denser H_2_ and water layers at the kaolinite surface, and consequently, to a significant reduction in H_2_ diffusivity. H_2_ diffusivities dropped from ca. 7.8 × 10^–9^ m^2^/s at a confinement distance of 60 Å to ca. 2.5 × 10^–9^ m^2^/s at 10 Å. Similarly, the study demonstrated that higher temperatures promote increased H_2_ mobility, with diffusion coefficients increasing from apporixmately 4.49 × 10^–9^ m^2^/s at 310 K to 9.27 × 10^–9^ m^2^/s at 410 K. The presence of divalent ions such as Mg^2+^ further restricts H_2_ motion compared to monovalent ions (Na^+^) due to their larger hydration shells and stronger electrostatic interactions. Overall, these findings underscored the critical roles of pore size, surface chemistry, temperature, and salinity in controlling H_2_ transport in confined systems, which are important implications for UHS technologies.

Hubao et al.^907^ investigated the diffusion and distribution of H_2_ in confined calcium silicate hydrate (C-S-H) nano-pores using MD simulations, covering a wide range of conditions, namely temperatures ranging from 300 to 370 K, pressures from 25 to 400 bar, and pore sizes spanning 2.5–40 nm. The study revealed that H_2_ molecules tend to accumulate near the C-S-H walls, forming a high-density adsorption layer. The diffusion of H_2_ was shown to be strongly anisotropic, with diffusion parallel to the walls being significantly faster than in the perpendicular direction. Furthermore, increasing pressure notably elevates the density and suppresses the overall diffusion coefficient, whereas higher temperatures reduce the density and enhance mobility. Although similar observations have been already made, for confined fluids, H_2_ exhibits a distinct single adsorption layer due to its relatively weak interactions with the surface compared to materials like water and CO_2_, for which multi-layer adsorption is often reported. Finally, the work demonstrated that as the pore size increases beyond a critical threshold (ca. 20 nm), the confined diffusion gradually approaches that of the bulk phase.

Raza et al.^908^ reported self–diffusivities of H_2_ in organic-rich shale, focusing on how H_2_ transport behavior changes with pore confinement, pressure, and indirectly with surface chemistry via kerogen type. The study considers two representative kerogen types (II-A, representing immature organic matter, and II-C, representing mature organic matter) with slit pore sizes of 0.5 and 2 nm. The temperature was constant at 360 K while pressures ranged from 30 to 400 bar. The findings indicated that the dominant transport mechanism is “transitional diffusion,” characterized by Knudsen numbers between 0.64 and 9.72, where H_2_ diffusion is a balanced interplay between slip flow and molecular diffusion. Across both kerogen types, a clear trend emerges where H_2_ diffusivity declines with increasing pressure, and, importantly, larger pores (2 nm) consistently yield higher self-diffusion coefficients (ranging from ca. 0.004 to 0.02 cm^2^/s for type II-A and 0.0037 to 0.019 cm^2^/s for type II-C) compared to the 0.5 nm pores. For a fixed nano-pore size, the thermal maturity of the kerogen does not have a discernible impact on H_2_ diffusivity even though type II-A tends to retain greater H_2_ density due to stronger adsorption effects. Finally, the diffusion data were successfully regressed to a power-law model with coefficients of determination (R^2^) being in the range 79–98%, providing a continuous description of the diffusivity–pressure relationship. Note that while the study explores variations with pore size and pressure in depth, the temperature was kept constant and specific variations in surface chemical properties beyond the kerogen type were not explicitly considered.

Kim et al.^909^ also studied the H_2_ diffusion within nanoporous shale caprocks. The simulations considered both binary (H_2_/CH_4_) and ternary (H_2_/CH_4_/CO_2_) gas mixtures confined by three distinct pore types i.e., organic pores formed by kerogen, inorganic pores formed by clay, and mixed–wet pores composed of both kerogen and clay. The simulations were carried out at 300 atm and 326 K, focusing on diffusive transport in two directions: perpendicular (*x* direction) to the pore faces (which, due to periodic boundary conditions, includes diffusion through the kerogen matrix) and parallel (*yz* direction) to the pore surfaces. Graham’s law states that the rate of gas diffusion varies inversely with the square root of its molecular weight (i.e., lighter gases diffuse faster than heavier ones). However, while the expectation is that H_2_ (having very low molecular weight) should diffuse much more rapidly than CH_4_ and CO_2_, the results by Kim et al.^909^ showed that the differences are moderated by additional factors such as surface affinity, adsorption by water and ions, and restrictions imposed by the available pore space. In fact, H_2_ exhibits a self-diffusivity in the order of 10^–7^ m^2^/s in the parallel direction in clay and mixed-wet pores, whereas diffusion through kerogen is reduced by 2–3 orders of magnitude owing to its much smaller pore sizes (ca. 1 to 10 nm). These findings illustrate that, beyond the influence of molecular weight, the interplay between adsorption phenomena and pore geometry is critical for understanding H_2_ diffusivity in geologic media.

Ghasemi et al.^910^ also performed MD simulations to investigate H_2_ diffusion in confined, water–saturated clay minerals, which is a representative caprock system for UHS. The authors systematically explored the effects of pore size, surface chemistry, and interlayer cations under conditions of 368.15 K and 300 bar. The study examined five clay mineral types, namely pyrophyllite (uncharged), Na- and Ca-montmorillonite, and Na- and Ca-beidellite (both negatively charged with different charge distributions), providing insight into how H_2_ mobility is affected confinement in the pores. For the charged clays, a critical pore size of ca. 2 nm was observed for which a substantial increase in the H_2_ diffusion coefficient occurred (increase of up to 24.06% relative to bulk water), while for pyrophyllite the diffusion coefficient increased monotonically with pore expansion, indicating that is not affected by surface charge. The study also showed that the distribution of negative charges (concentrated in the octahedral layer of montmorillonite versus the tetrahedral sheets in beidellite) significantly influences the positioning of H_2_ molecules relative to the clay surfaces, thereby affecting diffusion, whereas the effect of inter-layer cations (with Na^+^ allowing slightly higher mobility compared to Ca^2+^ due to their closer association with the clay surface and smaller hydration shell) is comparatively less pronounced. By using the Einstein relation to analyze molecular trajectories, the authors corroborated that confined H_2_ diffusion is markedly different from that in bulk water and converges toward bulk behavior as pore size increases beyond 2 nm, with the diffusion coefficient for some clays approaching that in bulk water (ca. 13.07 × 10^–9^ m^2^/s) at larger spacings (up to 8 nm).

A common oversight observed in many of the studies discussed above relates to the subtlety involved in diffusion parallel to interfaces. Determining diffusion coefficients of gases and liquids confined within cavities or at inhomogeneous interfaces, such as air-water or water-solid boundaries, presents unique challenges.^911–913^ In confinement, traditional methods like the Einstein or Kubo relations (see Section 3.2.1) become insufficient. For confined liquids, the MSD is inherently limited by the size of the confinement, making it difficult to clearly define a diffusion coefficient. For systems with inhomogeneous regions, particles typically spend a finite duration within a specific area before exploring others (see also the discussion on H_2_ diffusivity in polymers in Section 4.4). Consequently, the diffusion coefficient will vary across regions, and the MSD computed for particles initially localized in a particular region will only exhibit linear behavior after sufficient time has elapsed, allowing particles to sample all available regions. Ultimately, the slope of this long-term linear behavior represents an averaged diffusion coefficient across the entire system.

Berne and co-workers developed a methodology to quantify position-dependent diffusion coefficients in interfacial or confined fluids, where conventional MSD or GK approaches fail.^914^ The method is based on the anisotropic Smoluchowski equation, which describes molecular diffusion in systems with an inhomogeneous density profile arising from the influence of the solid-liquid or liquid-vapor interface. This spatial variation in density can be expressed as a potential of mean force that enters the Smoluchowski formulation, allowing diffusion to be resolved into diffusivities parallel and perpendicular to the interface. For the parallel component, MD trajectories are analyzed within each layer while imposing virtual absorbing boundaries. The mean lateral displacement of molecules that remain within a layer yields the local in-plane diffusivity. For the perpendicular component, where the location of a molecule from the solid-liquid or liquid-vapor interface strongly affects molecular motion, Langevin dynamics simulations are performed in parallel with MD using the same potential of mean force and boundary conditions. The friction parameter in the Langevin model is adjusted until the survival probability matches that from MD, from which the perpendicular diffusivity is inferred. For layers located well within the bulk phase, where the density is uniform and unaffected by interfacial gradients, the perpendicular diffusivity is readily obtained from the exponential decay of the molecular survival probability within that region. Although accurate and physically motivated, this dual-simulation approach can be cumbersome since Langevin dynamics must be solved alongside MD for each spatial region, motivating later efforts toward simpler alternatives for evaluating perpendicular diffusivities.

Of relevance in this context is the work of Mercier Franco et al.,^911^ who proposed an analytical alternative to the dual-simulation approach. In their method, the potential of mean force associated with regions of non-uniform density is linearized, allowing the anisotropic Smoluchowski equation to be solved analytically for the perpendicular diffusivity. In this framework, the perpendicular diffusivity is inversely proportional to the molecular residence time, defined as the time integral of the survival probability, within a given layer. Hence, layers where molecules remain trapped for longer times exhibit lower perpendicular diffusivities. The predicted diffusivities for water near the liquid-vapor interface agree closely with those obtained by Liu et al.,^914^,validating the underlying approximation. This methodology was later extended by Spera et al.^915^ to methane-ethane mixtures confined in calcite nanopores, where steep density gradients arise near the walls. Their study demonstrated that the perpendicular diffusivity in the first adsorbed layer is highly sensitive to the precise definition of the interfacial boundary, highlighting the importance of accurately identifying the starting point of the adsorbed layer. In all such cases, the perpendicular diffusivity remains consistently smaller than the corresponding parallel component. An alternative approach was proposed by Předota et al.,^916^ who directly computed spatially resolved diffusivities from molecular dynamics trajectories by tracking mean-square displacements within bins parallel to the interface. To minimize artefacts from molecules crossing between bins, half of the squared displacement was apportioned to the initial and terminal bins, and diffusivities were extracted from the linear regime of the mean-square displacement over intermediate timescales. This pragmatic treatment yields well-behaved perpendicular diffusivities even in finite slab geometries and provides a numerically efficient alternative to dual-simulation or analytical methods.

The methodology proposed by Berne and colleagues (and modifications of this methodology) has successfully been applied in multiple contexts, including the diffusion of water confined within clay materials,^917–919^ and more recently in H_2_ storage studies. For instance, Bui et al.^895^ used this method to computed water diffusion parallel to surfaces at various spatial locations, close to hydrophobic and hydrophilic surfaces, and within the bulk region. Among recent hydrogen diffusion studies, only Liu et al.^906^ and Hubao et al.^907^ explicitly considered diffusivities within distinct layers, while other studies^877–879,899,905,908–910^ did not follow this layered approach.

Considering these insights, we proceed by examining how the wall-parallel (*D_xy_* - lateral) and wall-normal (*D_z_* - perpendicular) diffusivities vary with pressure and pore size. Initially, we focus on the results from Liu et al.,^906^ since^906^ do not explicitly address these diffusion dependencies. In Fig. 52(a), both *D_xy_* and *D_z_* decrease with increasing pressure, approaching *D*_bulk_. This trend signifies reduced confinement effects at higher pressures, where H_2_-H_2_ collisions dominate over pore wall interactions. Under all conditions considered, *D_xy_* remains significantly higher than *D_z_*, clearly demonstrating anisotropic diffusion due to confinement by the nano-pore walls. At low pressures, diffusion within nano-pores strongly deviates from bulk values due to prominent wall interactions. H_2_ molecules become adsorbed on the C-S-H surfaces, significantly restricting mobility, particularly perpendicular to the walls. With increasing pressure, the nano-pores become densely populated, and molecular collisions dominate diffusion behavior, diminishing confinement effects. Consequently, diffusivities (especially the lateral component - *D_xy_*) approach bulk-phase values. Fig. 52(b) shows that at larger pore sizes (20–40 nm), *D_xy_* approaches bulk values faster than *D_z_*, indicating stronger confinement effects in the perpendicular direction. Overall, larger pore sizes enhance diffusion rates and reduce anisotropy. Liu et al.^906^ reported similar anisotropic diffusion behavior in nanoporous montmorillonite, where lateral mobility closely matches bulk-phase diffusion even near surfaces, while perpendicular diffusion is significantly hindered due to confinement and boundary interactions. Lastly, as emphasized by Ghasemi et al.,^910^ it is essential to account for hydrodynamic finite-size corrections when evaluating self-diffusivities in confined environments,^499^ a point initially highlighted by Rotenberg and colleagues.^920^ Among the reviewed studies, only Ghasemi et al.^910^ explicitly considered these corrections, reporting a mere 2% impact of confinement on H_2_ self-diffusivities. We conclude by urging future studies to consider both anisotropic diffusivities and finite-size hydrodynamic effects when reporting self- or mutual diffusivities of H_2_ mixtures under confinement.

**Figure 52:**
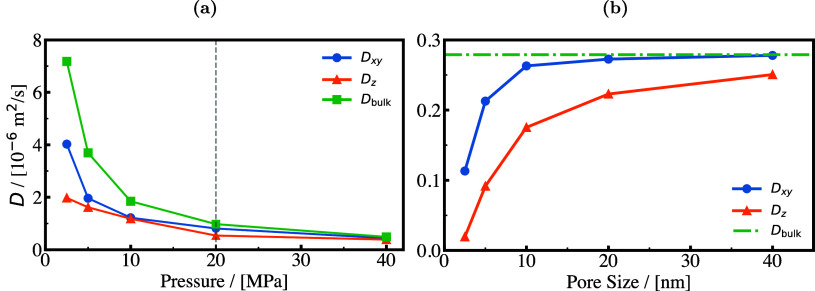
Self-diffusion coefficients of hydrogen in calcium silicate hydrate (C-S-H) pores computed with MD simulations. (a) Lateral (*D_xy_*, blue circles), perpendicular (*D_z_*, orange triangles), and bulk (*D*_bulk_, green squares) diffusion coefficients as a function of pressure at a fixed pore size of 5 nm and temperature of 340 K. A vertical dashed line indicates 20 MPa, corresponding to the conditions in (b). (b) Lateral (*D_xy_*, blue circles), perpendicular (*D_z_*, orange triangles), and bulk (*D*_bulk_, green dash-dotted line) diffusion coefficients versus pore size at a fixed pressure of 20 MPa and temperature of 340 K. All data in the plots are taken from Hubao et al.^907^.

#### 4.7.4 Hydrogen Intercalation and Leakage in Geological Nanopores

The behavior of H_2_ within geological nano-pores is highly affected by two complementary but distinct phenomena: (1) intercalation of H_2_ into hydrated clay nano-pores, and (2) H_2_ leakage through nano-pore defects and cracks. While intercalation studies primarily explore how H_2_ molecules enter and remain within clay layers, leakage studies focus on identifying mechanisms by which H_2_ may escape from these geological barriers. In this section, we first address recent advances in the energetics and molecular interactions governing H_2_ intercalation, followed by a discussion of factors influencing H_2_ leakage from nanoporous caprocks.

Ho et al.^921^ investigated H_2_ intercalation mechanisms in hydrated montmorillonite clay, relevant to H_2_ geological storage. Using meta-dynamics/MD simulations, the authors showed that dissolving H_2_ in bulk water is highly unfavorable (2.3 kcal/mol). Within clay interlayers (with spacings of 12.5 Å and 15 Å), H_2_ intercalation remains thermodynamically challenging, though slightly less so in hydrophobic regions near siloxane ring centers due to their lower local charge density. In contrast, regions with higher charge density from isomorphic substitutions (e.g., Mg replacing Al) and interlayer cations strongly repel H_2_ intercalation. Compared to H_2_, CO_2_ demonstrated significantly more favorable energetics, indicating that the limited polarizability and quadrupole moment of H_2_ severely restricts its interactions within these environments. Muther and Dahaghi^922^ extended this analysis by studying H_2_ intercalation in hydrated sodium montmorillonite (Na-MMT) clay under realistic geological conditions (1–50 MPa, 298–403 K). They observed similarly low H_2_ intercalation mole fractions (approximately 1.569 × 10^–6^ to 0.0119), attributed to the limited solubility of H_2_ in water, and its weak interactions with clay surfaces. Crucially, intercalation is significantly influenced by the hydration state, with increased uptake generally occurring during the transition from monolayer (1W, ∼12.5 Å) to bilayer hydration (2W, ∼15.5 Å), although slight decreases may result from water filling interlayer spaces. Competitive adsorption with CO_2_ and CH_4_ further reduces H_2_ uptake (by ca. 32% and 52%, respectively), highlighting complex interactions among gases that limit H_2_ storage potential but simultaneously mitigate risks to caprock integrity.

Li et al.^329^ studied H_2_ leakage through nano-cracks in kaolinite caprocks using MD simulations. The focus was on H_2_ near hydrophilic gibbsite and hydrophobic siloxane surfaces at 333.15 K and 30 MPa, varying water content and cushion gas compositions. On gibbsite surfaces, increasing water content significantly reduces H_2_ leakage–from nearly complete leakage at low water content to below 5% when water is sufficient to form dense adsorption layers. On siloxane surfaces, water distribution is less uniform, leading to variable H_2_ retention efficiency. For CO_2_, stronger adsorption in gibbsite and kaolinite aquifers enhances its collaboration with H_2_O to effectively block cracks. For CH_4_, while it helps plug H_2_ leakage on hydrophilic gibbsite by hindering entry, its role is minimal on siloxane surfaces, where the non-uniform distribution of H_2_O governs leakage control. These findings emphasize surface chemistry, water content, and gas composition as critical factors for leakage prevention. Liu et al.^923^ further explored H_2_ leakage through nanoporous shale caprock using MD and MC simulations. The authors highlighted that H_2_, due to the small molecular size (less than 1 Å), easily leaks through nano-pores, whereas larger molecules such as CH_4_ create stable adsorption layers, reducing H_2_ contact with rock surfaces. Water further reinforces caprock integrity by forming clusters through H_2_ bonding, enhancing the sealing effect in combination with CH_4_. Leakage increases with pore size, significantly intensifying beyond approximately 10.5 Å. Higher pressures accelerate leakage through distinct stages of blockage, partial penetration, and eventual breakthrough. Additionally, elevated temperatures weaken water clusters by disrupting H_2_ bonds, thereby increasing H_2_ leakage. This study underscores the critical interplay between pore geometry, surface chemistry, and environmental conditions in governing H_2_ leakage dynamics.

In summary, these studies collectively illuminate the complexities underlying H_2_ behavior in nanoporous geological formations. The low affinity of H_2_ for water and clay surfaces poses intrinsic barriers to efficient intercalation, while nano-pore surface chemistry, water content, gas composition, and operating conditions decisively influence leakage pathways. These insights from molecular simulations can significantly contribute to strategies for safer and more efficient UHS.

### 4.8 Hydrogen in Pure and Mixed Hydrates

#### 4.8.1 Hydrate–Liquid–Vapor Three-Phase Equilibria of Aqueous Hydrogen Solutions

Frankcombe and Kroes^934^ performed MD simulations using 2D, sII clathrate hydrate slabs with an occupancy equal to (1S + 4L) H_2_, in contact with a slab of H_2_ gas at a pressure of 1500 bar. The authors used different water force fields (SPC/E, TIP3P, TIP4P, TIP4P/2005, TIP4P/Ice, and TIP5P) to compare the predicted stability of the H_2_ hydrate in the simulation with the respective experimental data, and concluded that TIP5P yielded the best temperature–pressure stability prediction for the hydrate, predicting a decomposition temperature in the range 245–265 K. It should be noted, however, that this conclusion was the result of a single MD simulation, instead of using multiple replicas as discussed in detail in refs 369 and 578.

The first attempt to calculate the three-phase (H–L_*w*_ – V) equilibria (see Fig. 8 for notation) for the binary H_2_ – water system using the ”direct phase coexistence” approach was reported by Luis et al.^935^ The authors used TIP4P/Ice and the H_2_ force filed proposed by Alavi et al.^336^ (see details in Section 3.1), and reported predictions that were underestimating the three-phase equilibrium temperature by ca. 16–20 K. In a subsequent study, Luis et al.^936^ examined the effects of externally-applied static electric fields on the stability of H_2_ hydrates. The authors concluded that hydrate crystallization is prevented with a certain magnitude of electrical field, which depends on the pressure of the system. Namely, as the pressure increases, an electrical field of lower intensity is needed to inhibit crystallization.

Michalis et al.^342^ performed simulations with a modified “direct phase coexistence” approach and reported a significant improvement (i.e., by 10.5 K) in the calculation of the three-phase equilibria temperatures for the pressure range 100–300 MPa compared to the earlier study of Luis et al.^935^ The improvement was the direct result of implementing two modifications in the treatment of the H_2_ force-field. Namely, (i) the use of H_2_ LJ parameters that are a function of temperature, and (ii) the water–H_2_ guest energy interaction parameters were optimized further by using the Lorentz-Berthelot combining rules, accounting for the improved description of the solubility of H_2_ in water (i.e., Eq. ([Disp-formula eq8]) discussed in Section 3.1.1). While the simulations by Michalis et al.^342^ currently provide the most accurate description of the H–L_*w*_ – V equilibrium temperature for the binary H_2_ – water system, using the ”direct phase coexistence” approach, there is still room for improvements. In particular, there is still an under-prediction of the three-phase equilibrium temperature of the pure H_2_ hydrate by a Δ*T* that is higher than the value of 3.35 K, that the TIP4P/Ice water model under-predicts the melting point of ice I_*h*_. This is clearly shown in Fig. 53 which is constructed with data from ref 342. Another important observation made by Michalis et al.^342^, that needs future experimental/computational investigation, is the reported discrepancy between the experimental data for the three-phase equilibrium conditions for the binary H_2_ – water system of Dyadin et al.^244^ and Efimchenko et al.,^937^ especially at temperatures lower than 268 K (see Fig. 53).

**Figure 53:**
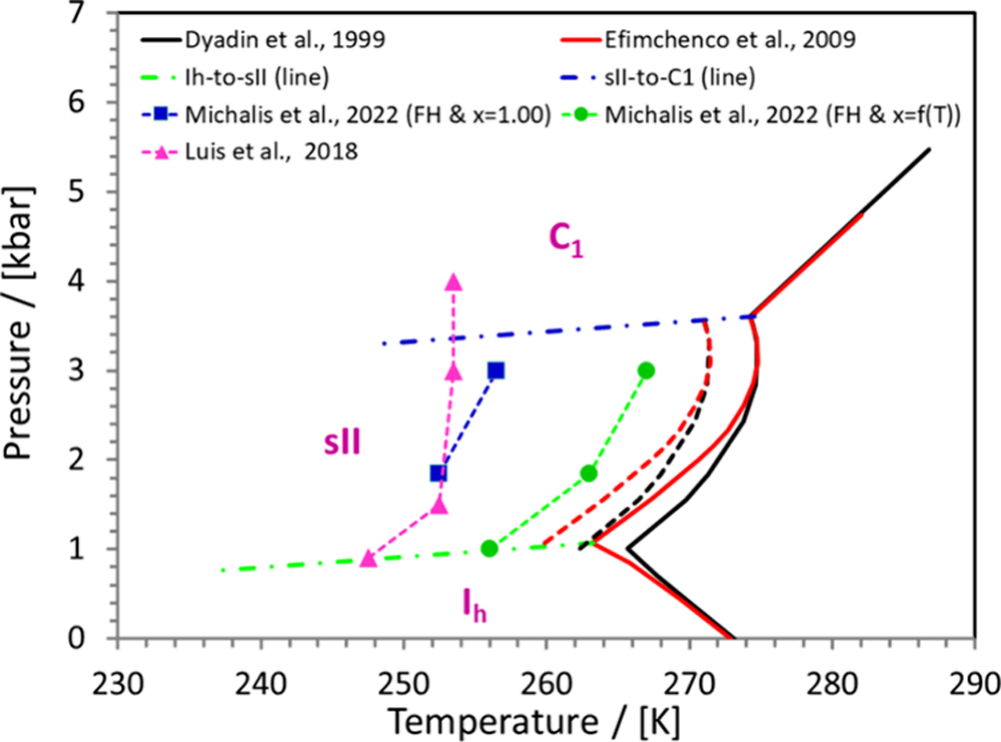
Experimental values (denoted with lines) and MD data from Michalis et al.^342^ [blue squares: Feynman–Hibbs (FH) and *χ* = 1.00; green circles: FH and *χ* = *f*(*T*)] and from Luis et al.^935^ (magenta triangles) for the three-phase coexistence temperature of the hydrogen hydrate system. The two dashed lines denote the MD-expected phase equilibrium lines corresponding to the experimental data of Dyadin et al.^244^ (black line) and Efimchenko et al.^937^ (red line). The MD-expected values presented in the figure are defined as *T*_Eq,expected_ = *T*_Eq,exp_ – 3.35 K, where *T_Eq_*_, exp_ is the experimental three-phase equilibrium temperature. Data extracted from ref 342.

Kang et al.^938^ used the “phase coexistence” approach to calculate the three phase equilibria for the mixed H_2_ + THF (5.56 mole% THF) hydrate at 100, 250, and 400 bar. The authors considered two different cases for the LJ parameters between the centers of the mass of H_2_ and the oxygen of H_2_O. Namely, the first case used LJ parameters based on ab initio calculations, while the second used the classical Lorentz-Berthelot mixing rule. As shown in Fig. 54 the ab initio-based parameters produce better agreement with the experimental measurements.

**Figure 54:**
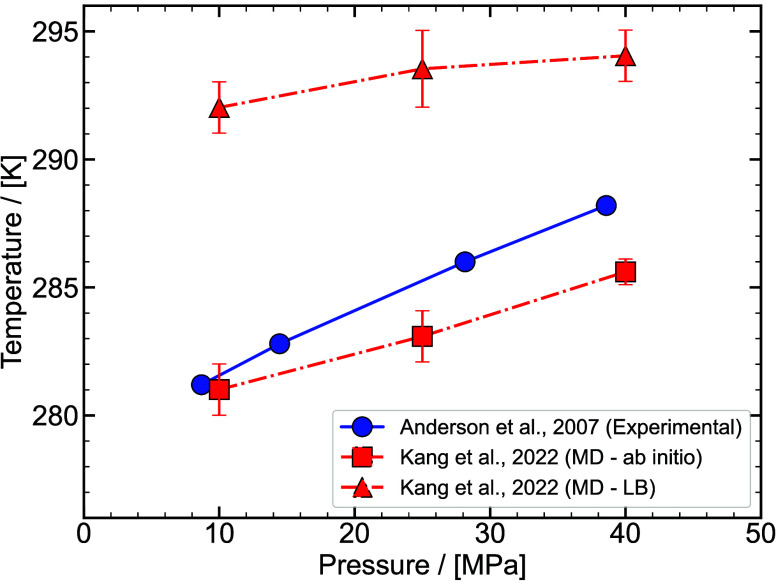
Phase equilibria (H–L_*w*_ – V) of H_2_ + THF hydrates. Blue circles denote experimental measurements (5.56 mole% THF) by Anderson et al.^939^ Red symbols denote the MD “phase coexistence” simulation values of Kang et al.^938^ Lines connecting the symbols are guides to the eye only.

#### 4.8.2 Kinetics of Hydrate Growth/Decomposition

Zhang et al.^594^ studied the growth of mixed H_2_ + CH_4_ hydrate with MD simulations, motivated by the high energy density of the particular gas mixture. The authors reported that the growth mechanism of the mixed hydrate is controlled by the solubility and diffusivity of the guest molecules. The complex interplay of diffusivity and solubility with the increasing temperature results in a growth rate and cage occupancy maximum at 250 K and a pressure equal to 50 MPa. Therefore, by an appropriate selection of *P*, *T* conditions certain storage capacities can be achieved. In a similar study Tian and Zhang^595^ studied the mixed H_2_ + THF hydrate with MD simulations and reported growth rates at (i) 50 MPa and temperatures in the range 230–270 K, and (ii) at 250 K and pressures in the range 20–110 MPa, reporting a maximum cage occupancy maximum at 260 K and a pressure equal to 50 MPa. The computed H_2_ storage capacity was shown to be correlated with the trend of the growth rate. Fig. 55a shows the hydrate growth rates computed with MD as a function of *T* (at *P* = 50 MPa), while Fig. 55b shows the calculated hydrate growth rates as a function of *P* (at *T* = 250 K) for the cases of H_2_ + CH_4_ (Zhang et al.^594^) and H_2_ + THF (Tian and Zhang^595^) mixed hydrates. Tian and Zhang^595^ observed that in MD simulations at 50 MPa, the mixed H_2_ + THF hydrate growth rates exhibit a maximum at 260 K, which is close to the temperature conditions of the maximum growth rates of pure THF hydrate. For temperatures above 260 K, a dramatic decrease is observed in the growth rate (see Fig. 55a). Notice that for the case of H_2_ + CH_4_ the maximum is obtained at 250 K. Contrary, as shown in Fig. 55b, a practically negligible dependence on pressure is observed (for constant *T* = 250 K) for the growth rates of the mixed H_2_ + THF hydrate. However, for the case of H_2_ + CH_4_, Zhang et al.^594^ reported an increase of almost 300% in the growth rate for the pressure range 20–110 MPa.

**Figure 55:**
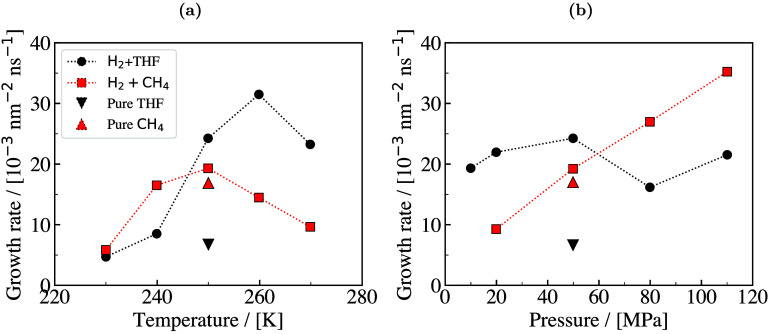
Comparison of computed growth rates of H_2_ + THF (black symbols – Tian and Zhang^595^) and H_2_ + CH_4_ (red symbols – Zhang et al.^594^) binary hydrates: (a) growth rate as a function of temperature at 50 MPa and (b) growth rate as a function of pressure at 250 K. The triangles represent the growth rates of pure THF and CH_4_ hydrates, respectively. Lines are drawn to guide the eye. The data are taken from ref 595.

While the studies of Zhang et al.^594^ and Tian and Zhang^595^ reported hydrate growth rates in units of­[nm^–2^ ns^–1^], other MD studies report growth rates in units of [cages/ns]. Such are the studies by Kang et al.^938^ for H_2_ + THF mixed hydrate, and Hu et al.^940^ for H_2_ + CH_4_ mixed hydrate. The combined results of the two studies are shown in Fig. 56, where the hydrate growth rate is plotted as a function of the THF (or CH_4_) concentration in units of [mol%]. From Fig. 56 it becomes evident that as the concentration of THF (or CH_4_) increases towards the stoichiometric value, hydrate formation rate decreases in both cases of mixed hydrates. Furthermore, similar to the conclusion reported by Tian and Zhang,^595^ a negligible effect of pressure on the growth rate can be seen in the MD simulations of Kang et al.,^938^ for the case of H_2_ + THF mixed hydrates.

**Figure 56:**
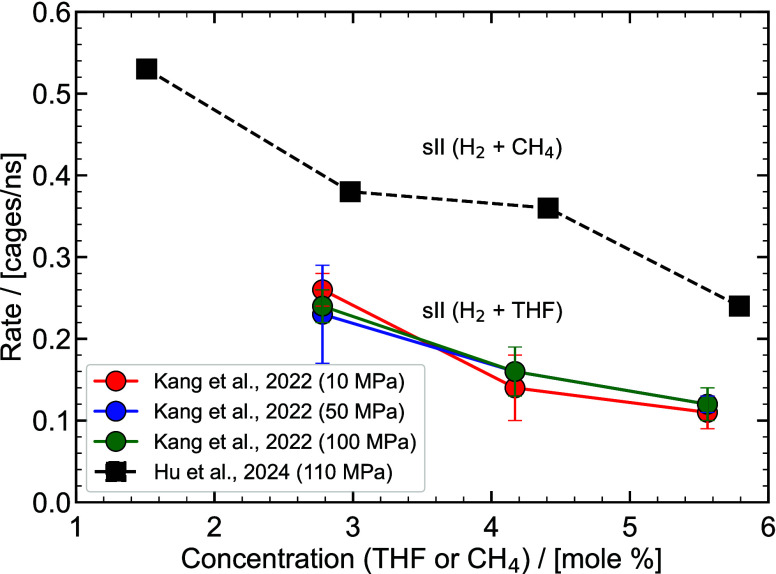
Hydrate growth rate as a function of THF (or CH_4_) concentartion. Comparison of growth rates of H_2_ + THF (red symbols – Kang et al.^938^) and H_2_ + CH_4_ (green symbols – Hu et al.^940^) binary hydrates computed with MD simulations. Lines are drawn to guide eye.

Ghaani and English^941^ performed both equilibrium and non-equilibrium MD simulations to study thermal-driven decomposition of mixed H_2_ + propane planar clathrate crystals with liquid water at 260–320 K. The authors concluded that the different surface-cavity terminations result in substantial differences in initial break-up rates. In a subsequent study, Ghaani et al.^942^ used non-equilibrium MD simulations to study the H_2_ release and uptake from/into propane planar clathrate surfaces a temperatures ranging from 180 to 273 K.^943^ reported experimental measurements and MD simulations of the mixed sII H_2_ + propane hydrate dissociation by heating or depressurization, and concluded that (i) H_2_ release from the hydrate crystal was much faster than that of propane, with experiments and simulations being in agreement, and (ii) simulations showed that hydrate dissociation induced by depressurization was slower than the case induced by heating, an observation which contradicts the experimental measurements reported in the same study. The authors tracked the number of H_2_ and propane molecules in the aqueous and hydrate phases. Only complete cages were considered for the counting of H_2_ and propane molecules in the hydrate crystal. They also tracked the number of hydrogen bonds and the potential energy. The number of hydrogen bonds was closely associated with the degree of crystallization of water molecules, reflecting, thus, the kinetics of hydrate dissociation.

#### 4.8.3 Structural and Other Properties of Hydrates

Wang et al.^180^ combined QM calculations with classical MD to compute average structure and vibrational spectra of H_2_ in pure sII H_2_ clathrate. The authors reported that the H_2_ molecules in the L cages and the singly-occupied S cages are stretched, and the vibrational mode is softened and uncoupled. Contrary, in the case of doubly-occupied S cages, the H_2_ molecules were shown to be slightly compressed, with the vibrational frequencies being close or above that of the free gas. Furthermore, a strong vibrational coupling was observed as a result of the short intermolecular distance between the two H_2_ in the S cages.

Gorman et al.^944^ performed MD simulations to investigate the dynamical and energy-related properties in sII pure H_2_ and mixed H_2_ + THF hydrates at 30 and 200 K (also at intermediate temperatures) and 0.05 kbar, using SPC/E and TIP4P/2005 water models. The study ignored the quantum effects which are expected to be important at temperatures below 150 K. Mondal et al.^334^ performed MD simulations to calculate binding energies and radial distribution functions for various cases of occupancies of the sI pure H_2_ hydrate, using the SPC/E water model. By observing the radial distribution functions of H_2_ loaded hydrates, the authors concluded that within the time-scale considered (200 fs), the H_2_ hydrate cages are not ruptured. In a later study,^945^ also performed MD simulations on structure and dynamics of high-pressure ices and filled ices, using TIP4P and TIP5P water models. This study focused on the rotational motions of both H_2_O and H_2_ molecules in the filled-ice C_2_ in addition to those in ice VII.

Rick and Freeman^364^ used three water models (SPC/E, TIP4P/Ice, and TIP4P-FQ/Ice) to predict dielectric constants, the proton order parameters, and the molecular volumes for pure Ar and H_2_ clathrate systems. The conclusion provided was that the dielectric response arises only from the water molecules as a result of the fact that the guest molecules do not have a dipole moment. English et al.^946^ performed equilibrium MD simulations to investigate thermal conduction mechanisms via the GK approach (see Section 3.2.1) for sII pure H_2_ hydrate, at 0.05 kbar and temperatures in the range 30–250 K. The authors considered the TIP4P water model, and assumed single or double occupancy of the 5^12^ cages and quadruple occupancy of the 5^12^6^4^ cages. Consistent with prior literature, in this study it was pointed out that for temperatures of ca. 150 K and below, it is expected that classical MD will not accurately capture the quantum nature of H_2_ motion. Zhao et al.^947^ performed MD simulation, as well as DFT and AIMD simulations, of spontaneous formation of quasi-one-dimensional (Q1D) H_2_ gas hydrates within single-walled carbon nanotubes (SW-CNTs) of nanometer-sized diameter (1–1.3 nm) occurring at near ambient temperature. It was observed that the guest H_2_ molecules in the Q1D hydrates are contained within a 1D nanochannel in which the H_2_ molecules form a molecule wire, while in conventional H_2_ hydrates the guest H_2_ molecules are contained in individual 3D cages.

#### 4.8.4 Hydrate Storage Capacities from Molecular Dynamics Simulations

The pioneering work of Alavi et al.^336^ was the first one to report anisotropic *NPT* MD simulations of pure sII H_2_ hydrates with flexible cages and periodic boundary conditions. H_2_ molecules were modeled using the potential developed within this study (see details in Section 3.1.1), while SPC/E force field was used for water. Water molecules of the hydrate lattice were allowed to move about their equilibrium positions. Simulations were performed at 1.013 and 2.5 kbar, temperatures in the range of 100–250 K, and for various H_2_ occupancies in each cage type, ranging from (1L + 0S) to (4L + 2S). Alavi et al. showed that the energy per unit cell for each H_2_ occupancy makes a large ”jump” when going from the single to the double occupancy of the S cage. This was interpreted as a strong indication that the double occupancy in the S cages would not be stable. A subsequent study^343^ using MD (*NPT*) simulations of pure sII H_2_ hydrates with the SPC/E water model, and a H_2_/D_2_ potential parameterised to account for QM effects of H_2_ motion at low temperatures, resulted in similar conclusions regarding the H_2_ occupancies in the L and S cages.

Alavi et al.^948^ extended their previous study of pure H_2_ hydrates^336^ to binary, THF + H_2_ sII hydrates to study the energetics of the occupancy of the H_2_ guests in the S and L cages, and again reached to similar conclusions regarding the cage occupancies. Furthermore, the simulations revealed that replacing a THF molecule in the L cages of the unit cell by a cluster of four H_2_ molecules is accompanied by an increase in unit cell total potential energy and volume. The authors examined the possibility of observing the tuning effect, discussed earlier,^247^ and concluded, based on MD data, that the formation of binary THF + H_2_ sII hydrates with large fractions of the L cages occupied by the H_2_ molecules is not likely to occur if large concentrations of THF are present in the initial hydrate-forming aqueous solution. Such an observation is consistent with MC simulations and experimental studies (e.g., see the detailed discussion in refs 177 and 248). Alavi et al.^949^ also performed classical MD simulations for the case of the binary sH methyl-tert-butylether + H_2_ clathrate hydrate, and similarly to prior literature,^336,343,948^ it was observed that the double H_2_ occupancy of the small (S) and medium (M) cages of the sH hydrate leads to a large increase in energy of the unit cell compared to the single H_2_ occupancies of these cages.

Daschbach et al.^950^ performed MD simulations in order to investigate H_2_ storage in *β*-hydroquinone clathrate and showed that higher storage capacities are obtained at lower temperatures. Zhang et al.^594^ studied the mixed sII H_2_ + CH_4_ hydrate with MD simulations, and reported cage occupancies at (i) 50 MPa and temperatures in the range 230–270 K, and (ii) at 250 K and pressures in the range 20–110 MPa. The findings were single H_2_ cage occupancies for the case of 5^12^ cages, while up to triple H_2_ occupancies for the case of 5^12^6^4^ cages. In particular, the simulations indicated that the 5^12^ cages are occupied by either single H_2_ or single CH_4_ molecules, while the 5^12^6^4^ cages are occupied by either single CH_4_ or single or double or triple H_2_ molecules. Note, however that the authors also mentioned the existence of 5^12^6^4^ cages with simultaneous occupancy of H_2_ and CH_4_, encountered especially at high temperatures and pressures. The importance of this issue will be further discussed in Section 4.8.8. In similar studies, Tian and Zhang^595^ and Kang et al.^938^ studied the mixed sII H_2_ + THF hydrate with MD simulations, focusing on occupancies at similar temperature and pressure ranges as in ref 594. The observations regarding the cages H_2_ occupancy were consistent with the prior studies. Wang et al.^951^ performed MD simulations to explore the H_2_ storage capacity of the mixed sH H_2_ + methylcyclohexane (MCH) hydrate at 240–260 K and 70–110 MPa. A H_2_ storage capacity in the range 0.5 to 1.0 wt.% at pressures between 70–110 MPa and temperatures 250 or 260 K was reported, while the storage capacity increased to 1.6–2.0 wt.% at 240 K at the same pressure range. The authors also indicated that the 5^12^ cages at the hydrate crystal boundary can prevent the diffusion of H_2_ inside the hydrate crystal phase.

The collective behavior of storage capacities of the three mixed hydrates (i.e., H_2_ + CH_4_; H_2_ + THF; and H_2_ + MCH) predicted from MD simulations as a function of pressure/temperature is shown in Fig. 57 and Fig. 58, respectively. As can be observed from Fig. 57, at a fixed temperature, the storage capacity increases with pressure. In contrast, at a fixed pressure, higher storage capacities occur for the lower temperatures. Among the mixed hydrates considered, the hydrate with the highest storage capacity is the mixed sH H_2_ + MCH, resulting from the increased amount of H_2_ diffusing into the 5^12^ and 4^3^5^6^6^3^. Such behavior for the storage capacity will be further discussed in Section 4.8.5, where storage capacities computed via MC simulations are considered. Similar conclusions can be drawn from Fig. 58. A notable exception, however, is the study of Tian and Zhang^595^ for the mixed hydrate H_2_ + THF, where it shown that at 50 MPa, the storage capacity increases as the temperature increases as a result of the tuning effect (i.e., the THF concentration is lower than the stoichiometric one (5.56 THF mol%)). The hydrate tuning effect has been a controversial issue, extensively discussed in the literature (see the reviews by Tsimpanogiannis and Economou^240^ and Alavi and Ripmeester^177^ for more details on experimental and MC studies). Only limited work has been reported using MD simulations.

**Figure 57:**
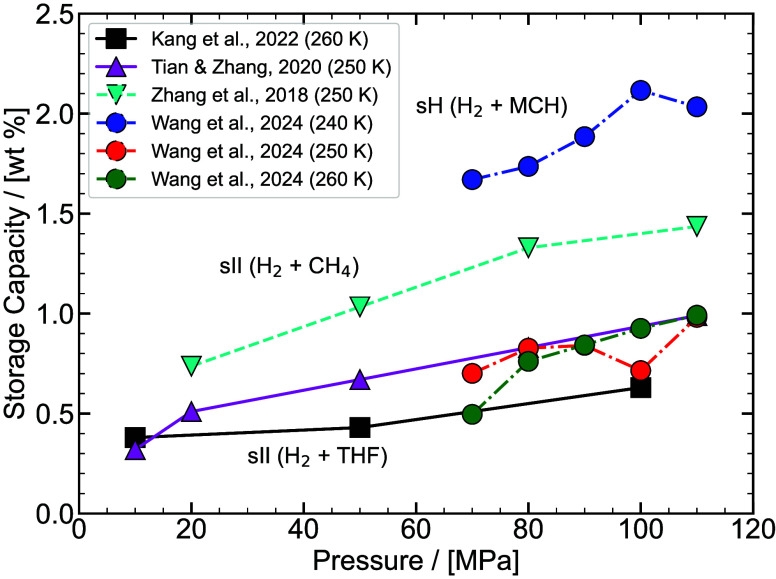
Hydrogen content (storage capacity in wt%) as a function of pressure for various temperatures and mixed hydrate systems (at stoichiometric promoter composition): (i) sII H_2_ + CH_4_ (Zhang et al.^594^ – green triangles), (ii) sII H_2_ + THF (Kang et al.^938^ – red circles; Tian and Zhang^595^ – red triangles), and (iii) sH H_2_ + MCH (Wang et al.^951^ – blue symbols). Lines connecting the symbols are to guide the eye.

**Figure 58:**
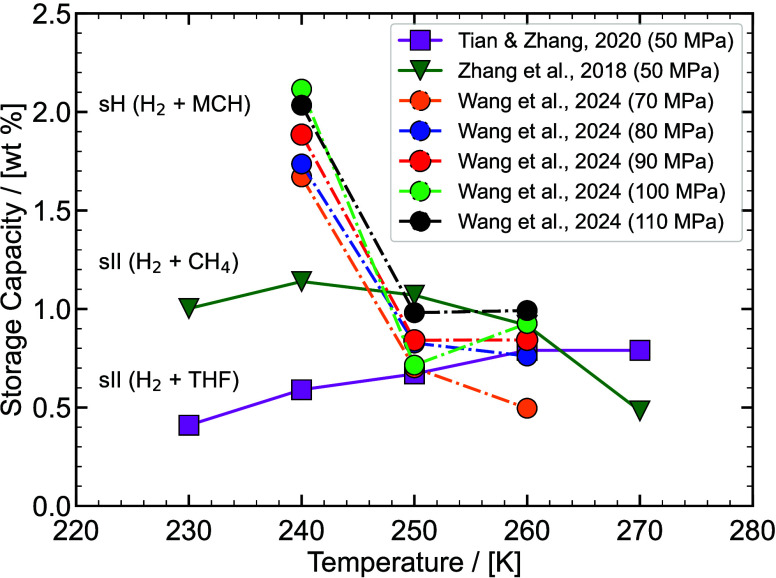
H_2_ content (storage capacity) as a function of temperature for various pressures and mixed hydrate systems (at stoichiometric promoter composition): (i) sII H_2_ + CH_4_ (Zhang et al.^594^ – green triangles), (ii) sII H_2_ + THF (Tian and Zhang^595^ – red circles), and (iii) sH H_2_ + MCH (Wang et al.^951^ – blue symbols). Lines connecting the symbols are to guide the eye.

Hu et al.^940^ studied the mixed H_2_ + CH_4_ hydrate with MD simulations, and reported the effect of the promoter (i.e., CH_4_) concentration on the H_2_ cage occupancies. Five types of cage occupancies for H_2_ molecules were observed in the MD simulations, namely: (i) single H_2_, (ii) single H_2_ – single CH_4_, (iii) double H_2_, (iv) double H_2_ – single CH_4_, and (v) triple H_2_. The 5^12^ cage can only be occupied by a single H_2_, while for the 5^12^6^4^ cage all five of the the above situations can occur. There was no strong tuning effect observed in their study. Kang et al.^938^ performed MD simulations for the mixed H_2_ + THF hydrate, and explored the effect of the promoter (i.e., THF) concentration on the H_2_ cage occupancies. In this study, tuning effect was clearly identified. Fig. 59 shows the H_2_ storage capacity of two sII mixed hydrates as a function of the promoter concentration for the two aforementioned studies. Notably, Wang et al.^951^ could not identify any tuning behavior for the mixed sH H_2_ + MCH hydrate.

**Figure 59:**
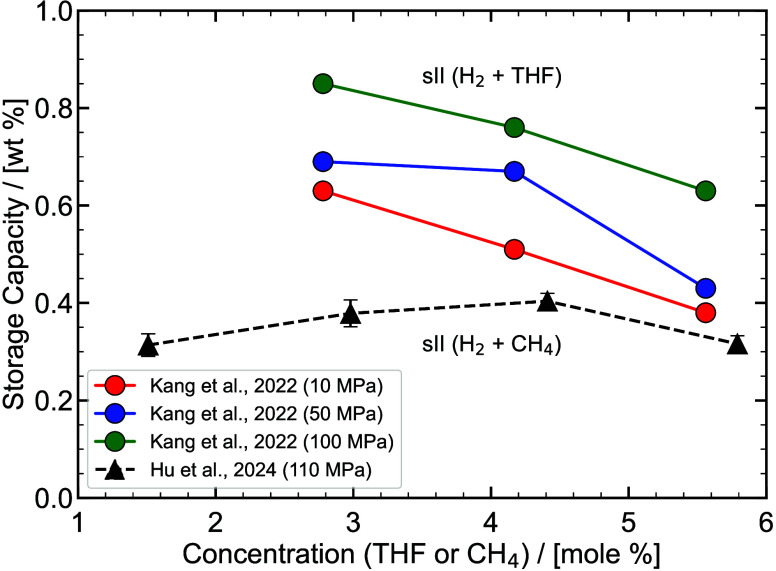
H_2_ content (storage capacity) as a function of promoter concentration: (i) sII H_2_ + CH_4_ (Hu et al.^940^ – green squares), (ii) sII H_2_ + THF (Kang et al.^938^ – red symbols). Lines connecting the symbols are to guide the eye.

As pointed out in the review article by Alavi and Ripmeester^177^, a consensus is developing based on experimental, classical MD, and MC studies, indicating that in either pure or mixed H_2_ hydrates (sI, sII, sH) the stable hydrate occupancy configuration is the one with a single H_2_ molecule in the S cages (or M cage of sH hydrates), while multiple occupancies are possible for the L cages. It should be mentioned that DFT studies (e.g., see discussion in^177^) have reported occupancies higher than classical MD or MC simulations, however, a thorough discussion on this discrepancy is beyond the scope of our review.

#### 4.8.5 Hydrate Storage Capacities from Monte Carlo Simulations

For a clathrate hydrate structure to be used as a gas-storage material, the number of guest gas molecules enclathrated inside each cavity becomes a critical factor that determines the total storage capacity of the specific hydrate structure. If quadruple occupancy of the L (5^12^6^4^) cavities and double occupancy of the S (5^12^) cavities^171^ is assmumed, the storage capacity of sII H_2_ hydrate is 5.0 wt.%. Contrary, the storage capacity drops to 3.8 wt.% H_2_ if single occupancy of the S cavities is assumed while retaining the quadruple occupancy of the L cavities.^952^ The multiple occupancy of a single cavity can significantly increase the storage capacity of the particular hydrate structure. Therefore, it is essential to know how the cavity occupancies evolve as a function of temperature, pressure and the possible presence of other co-guests (promoters or inhibitors) to accurately calculate the storage capacity of different clathrate hydrate structures. To this purpose, GCMC simulations have been proven to be a valuable tool.

The first study to report GCMC simulations of the pure sII H_2_ hydrate was by Katsumasa et al.^953^ Subsequently, Papadimitriou et al.^634^ examined the cases of (i) pure sII H_2_ hydrate, and (ii) the binary sII H_2_ + THF hydrate, Chun and Lee^954^ considered the binary sII H_2_ + THF hydrate, and Nakayama et al.^955,956^ (using the TIP5P water model) studied binary sII H_2_ + acetone hydrate. These MC studies computationally proved the single occupancy of the 5^12^ cages of structure sII, which is in good agreement with subsequent experimental measurements. For a detailed discussion on this subject, the reader is referred to ref 248. It should be noted though, that the GCMC simulations by Koh et al.^957^ showed the opposite, i.e., double occupancy in the small cages.

Tanaka and co-workers, extended their previous work on H_2_ storage in hydrates to various ices (I_*c*_, II), as well.^958,959^ After establishing the possibility of forming sII H_2_ hydrates, other hydrate structures were examined as well. Duarte et al.^960^ reported experiments of sH H_2_ hydrate formation in the presence of methylcyclohexane (MCH), 1,1-dimethylcyclohexane (DMCH) and methyl tert-butyl ether (MTBE), followed by GCMC simulations of sH H_2_ hydrate with MCH as promoter.^635^ Subsequently, sI H_2_ hydrates with promoter (ethylene oxide) were studied via means of GCMC simulations in ref 636. Both these GCMC studies, also considered the hypothetical pure sH and sI H_2_ hydrates, since they constitute the upper limiting case for the storage capacities of the two particular hydrate structures. Pure sI H_2_ hydrate was subsequently experimentally synthesized,^961^ thus, confirming the hypothesis of Papadimitriou et al.^636^ regarding the existence of the pure sI H_2_ hydrate.

Papadimitriou et al.^962^ presented an overview and a detailed discussion of the three common H_2_ hydrate structures considered in the earlier studies. The case of pure H_2_ hydrates was later revisited in a series of GCMC studies.^248,341,640^ In these studies, an elaborate and self-consistent MC-based approach was used to relate the chemical potential of the guest with the pressure of the system (see more details about this method in Section 3.3.3). This is important, since earlier studies had used a much simpler approach for the calculation of the chemical potential, based on the use of general-purpose^634–636^ or component-specific^638^ EoS.

A systematic study and quantification of the effect of the lattice constant of sII H_2_ hydrate on the storage capacity of the particular hydrate structure was reported in ref 341 while preliminary results following a simpler approach were presented in ref 634. Both studies were motivated by experimental observations^957^ indicating that the presence of different promoters in the binary ”H_2_ + Promoter” results in sII hydrates with differences in the lattice constant in the range 0.60 – 3.24% comparing to the value (1.7047 nm)^171^ of the sII pure H_2_ hydrate. The increase in the lattice constant can result in an increase in the H_2_ storage capacity by up to 3% when compared to the base case.

The effect of three water force fields (i.e., SPC/E, TIP4P/Ice, and TIP5P) on the computed storage capacities of the three most common pure H_2_ hydrates (i.e., sI, sII, and sH) was reported in ref 248. It should be noted that this study did not reveal a significant effect of the different force fields on the cage occupancies and the corresponding storage capacities (i.e., less than 5% differences were reported). In this context, the use of SPC/E for water and the single-site FH effective potential (see Eq. ([Disp-formula eq7]) in Section 3.1.1) for H_2_ was recommended for the use in GCMC simulations in order to calculate storage capacities. This conclusion is in contrast to the findings of MD simulations studies,^369,578^ where it was shown that to accurately calculate the three-phase equilibria conditions, using a water model that can accurately predict the melting point of hexagonal ice, I_*h*_, is essential. Therefore, the use of TIP4P/Ice was proposed in these studies. In a related context, Brumby et al.^633^ performed MC simulations in the GE that are in good agreement with the results reported in ref 248.

Currently, there seems to be consensus between the various MC simulation studies regarding the occupancy of the small cavities of the three most common hydrates structures. These cavities are singly occupied and cannot accommodate more than one H_2_ molecules even at the highest pressures examined (e.g., 500 MPa). Contrary, the large cavities of the sI, sII, and sH hydrates, may contain up to 2.3, 3.8, and 6.7 H_2_ molecules, respectively. The deviation between the water models regarding these occupancy values is less than 4%.

A detailed comparison of the experimental^939,963–965^ H_2_ storage capacities in binary H_2_+THF hydrates with earlier MC simulations^634,954^ is shown in Fig. 60, along with a special modification^966^ of the classical van der Waals–Platteeuw theory. Using the latter, one is capable of performing calculations at the limit of the 100% occupancy of cages for water-soluble promoters (such as THF). At this limit thermodynamic models based on the classical van der Waals–Platteeuw theory fail to provide any results.

**Figure 60:**
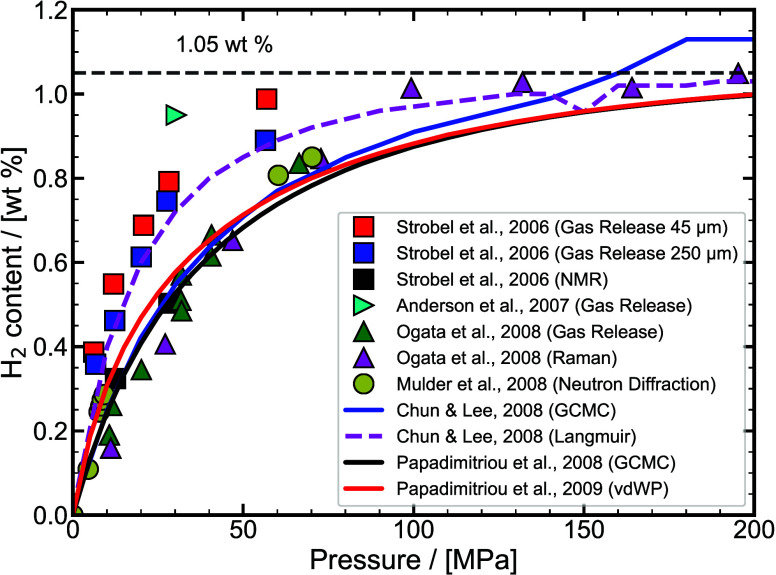
Hydrogen content as a function of pressure for the sII binary H_2_ + THF hydrate. Comparison between: (i) experimental measurements (Strobel et al.;^963^ Anderson et al.;^939^ Ogata et al.;^964^ Mulder et al.^965^), (ii) GCMC simulations (Chun and Lee^954^, Papadimitriou et al.^634^), and (iii) the modified van der Waals–Platteeuw (vdWP) theory, proposed by Papadimitriou et al.^966^ The horizontal black dashed line (at 1.05 wt.%) denotes the case where all small cavities (i.e., 100% occupancy) are occupied by a single H_2_ molecule.

An extensive comparison with results from updated MC simulation studies^248^ and additional experimental measurement^967–971^ was presented by Papadimitriou et al.^248^ for the case of binary H_2_ sII hydrates. This study presented comparisons under isothermal (i.e., 268, 274, and 278 K) conditions. Fig. 61 shows the data reported in ref 248 along with the corresponding comparison for the 255 K isotherm, using experimental data from Sugahara et al.^972,973^ and Kawamura et al.^974^ The GCMC results of Papadimitriou et al.^248^ were later used by Tsimpanogiannis et al.^975^ to develop engineering–type correlations for the H_2_ storage capacity of the three most common hydrate structures. In principle, the considered methodology could be used to examine storage in hydrates with various promoters, using the assumption that there is no change in the lattice constant resulting from the use of different promoters. This is a reasonable assumption based on the conclusions of Papadimitriou et al.,^341^ discussed earlier. This assumption could be relaxed in future studies, nevertheless, it would require a significant number of additional MC simulations to be performed to account for the change in the lattice constant of the hydrate crystals resulting from the use of different promoters.

**Figure 61:**
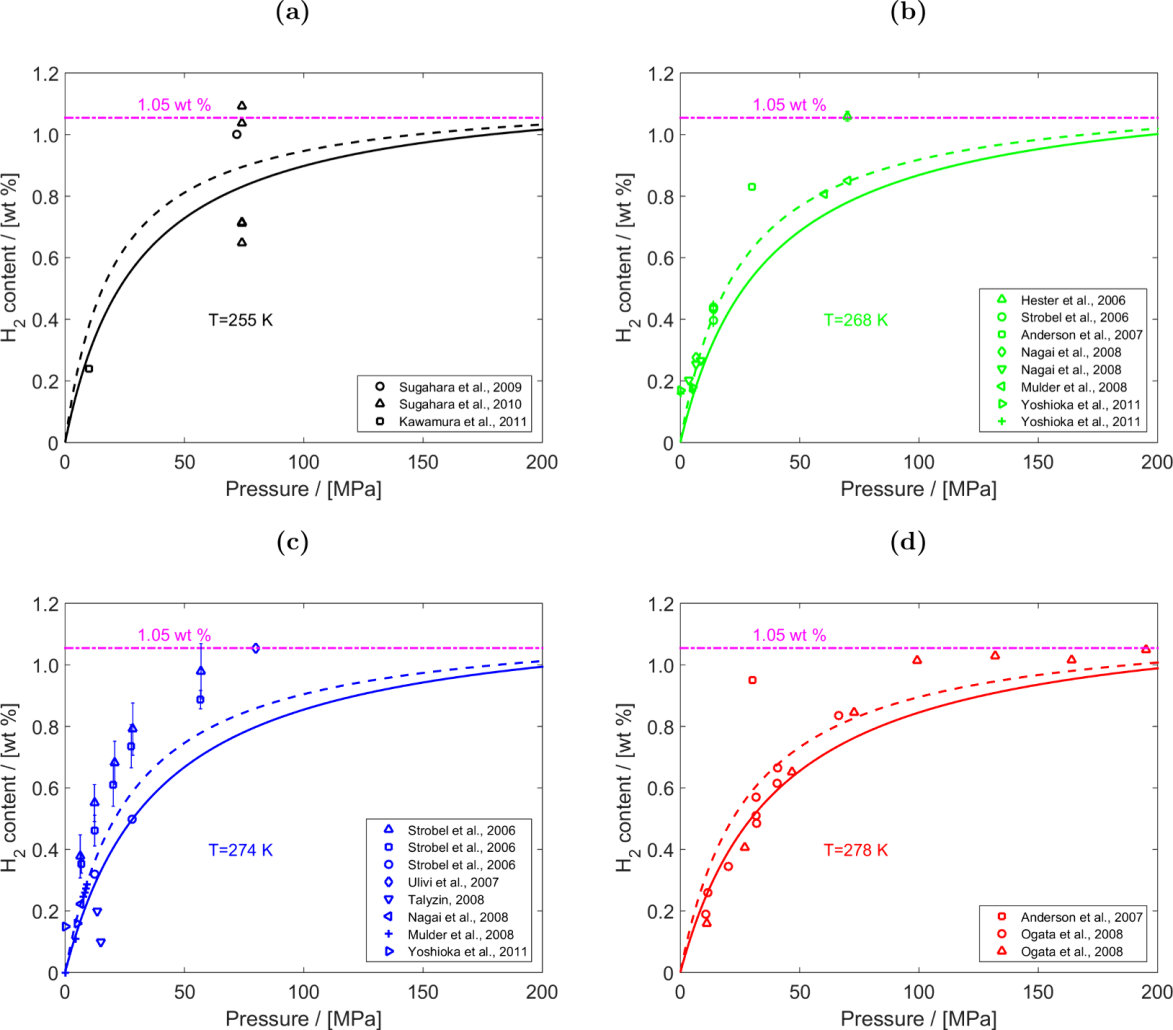
Hydrogen content (wt.%) as a function of pressure for the sII binary hydrate H_2_ + THF using the Silvera–Goldman H_2_ force field. Comparison between experimental measurements, denoted with symbols (Strobel et al.;^963^ Anderson et al.;^939^ Ogata et al.;^964^ Mulder et al.;^965^ Hester et al.;^967^ Nagai et al.;^968^ Yoshioka et al.;^969^ Ulivi et al.;^970^ Talyzin^971^), and the MC simulations by Papadimitriou et al.^248^ (denoted with lines; solid lines correspond to SPC/E and dashed lines to TIP4P/Ice). The comparison is for four different temperatures: (a) 255 K, (b) 268 K, (c) 274 K, and (d) 278 K. The magenta dashed-dotted line corresponds to the limiting case of 100% of small and large cages occupied with a single H_2_ and THF molecule respectively. The figure is reconstructed based on the data of ref 248. Error bars are shown only for cases that were reported in the original studies.

A general observation we can make from all the GCMC simulation studies reviewed here is that the US DOE specifications regarding the H_2_ storage capacity of pure H_2_ hydrates are not met when considering mobility/vehicular applications.^192,197^ Namely, the storage capacity is expected to be lower than 4 wt.%. Even lower storage capacities (i.e., less than 2 wt.%) are expected for the cases of mixed H_2_ + promoter hydrates. However, there is still significant scientific interest on H_2_ storage in clathrate hydrates for stationary applications.

#### 4.8.6 Hydrate-Based Gas Mixture Separation Efficiencies

While a considerable number of studies have focused on the experimental aspects of hydrate-based separation of H_2_ – containing mixtures,^976–979^ to the best of our knowledge, no relevant MC simulation study has been performed. MC studies for other hydrate mixtures, however, are available in literature: Glavatskiy et al.^980^ performed GCMC simulations of mixed CH_4_ + CO_2_ hydrates, motivated by the possibility of CO_2_ replacing CH_4_ within hydrate deposits. Such a process has gained significant attention lately due to the fact that it can simultaneously take care of two important issues. Namely, the energy production (i.e., CH_4_) and the sequestration of a “green-house” gas (i.e., CO_2_) within a single process. In contrast, the study by Papadimitriou et al.,^981^ which focused on computing the efficiencies of a hydrate-based gas-mixture separation process (the latter being the motivation), examined the same mixed CH_4_ + CO_2_ hydrate system, and reported good agreement between the experimental and GCMC simulations.

#### 4.8.7 Hydrate-Promoter Selection

Atamas et al.^982^ used the self-referential technique to compute the Gibbs free energy of molecular crystals. The self-referential technique utilizes the principle that the free energy is an extensive quantity. Therefore, it follows that by constructing a reversible path between two systems that contain *N* and 2*N* particles, respectively, the free energy difference between the two systems can be calculated as well as the free energy of the system itself. Subsequently, the authors examined the storage of H_2_ in sII^983^ and sH^984^ hydrates. The binary H_2_ + promoter hydrate with lower Gibbs free energy is more stable. The main purpose of both studies was to identify appropriate promoter molecules. An important conclusion of these studies is the identification of THF as one of the most promising promoters for H_2_ hydrates. Hydrate promoters are molecules that shift the hydrate equilibrium curve to more moderate conditions (i.e., lower pressures or higher temperatures). Hydrate promotion acts in the opposite direction to hydrate inhibition (i.e., a hydrate inhibitor results in shifting the hydrate equilibrium curve to higher pressures or lower temperatures).

Motivated by the need to identify promoters for storing H_2_ in clathrate hydrates, Frankcombe and Kroes^985^ introduced a new predictive computational method for classifying clathrate hydrate promoter molecules, based on the interaction energies between potential promoters and the water networks of sII and sH clathrates. To this purpose, water was modelled using the TIP5P force field. On the other hand, the promoter molecules were frozen at the geometries minimising the MP2/cc-pVTZ energy, either at the global minimum or at a metastable conformer. Ab initio calculations of the electronic structure of the promoter molecules, with the charges constrained to reproduce the calculated dipole moment of the guest molecule were performed using Gaussian 03. Atamas et al.^986^ introduced a generalization of the method by Frankcombe and Kroes,^985^ extending it to finite temperatures and pressures. The method is based on Gibbs free energy calculations of sI, sII, and sH H_2_ clathrates. Atamas et al. reported that the van der Waals volume of a potential promoter is an important parameter which can determine the type (either sII or sH) of clathrate to be formed.

Iwai and Aokawa^987^ performed MD simulations for the sII mixed H_2_ + promoter hydrates to search for potential promoters to stabilise the H_2_ hydrates. Simulations were performed at 10.1 MPa and an initial temperature 260 K (increased at a rate 0.1 K/s). A slow increase of the hydrate cell volume was initially observed, followed by a rapid increase. The temperature at which the cell volumes rapidly increased was identified as the simulated collapse temperature. The promoter (cyclobutane) which gave the highest simulated collapse temperature was identified as the one to best stabilise the hydrates.

Mi et al.^246^ performed an extensive series of equilibrium MD simulation to study the effect of various promoters (i.e., CH_4_, CO_2_, C_2_H_6_, C_3_H_8_, C_5_H_10_, and THF) on the nucleation of mixed H_2_ + promoter hydrates. Computations, up to 3 *μ*s, were performed at a pressure of 110 MPa and a temperature of 240 K to computate free energies, diffusivities, residence time correlation functions, order parameters, and H_2_ uptake. Mi et al. recommended that instead of using individual properties for identifying the suitability of a tentative promoter for enhancing the behavior of H_2_-containing hydrates, it is essential to examine the collective control of the aforementioned thermodynamic factors. The authors identified four criteria that an ideal hydrate promoter should possess: (i) High solubility in water for maximizing the guest molecule availability close to the hydrate formation front; (ii) Optimized residence time to enhance nucleation without the need for excessive mobility; (iii) Strong hydrogen bonding interactions to facilitate stable cage formation; (iv) Environmental sustainability to minimize ecological impact.

#### 4.8.8 Self-Diffusivity of Hydrogen in Hydrates

Here, we briefly review the most important experimental work in the area of diffusivity of H_2_, to lay the ground for the review of molecular simulation studies. Earlier studies^988^ considered that single H_2_ molecules were too small to be confined within the hydrate cages, and thus, were unable to provide the required stability to any of the hydrate structures. It was assumed that eventually the H_2_ molecules would be able to diffuse through the pentagonal or hexagonal faces of the cages and escape out of the hydrate crystal, ultimately leading to the complete dissociation of the hydrate. However, following the initial experimental synthesis of the H_2_ hydrate,^241,244,245^ experimental and computational evidence began to accumulate that under reduced pressure conditions, the H_2_ content of the hydrate crystal can decrease with time, as a result of H_2_ migration, however, without the complete breakdown of the hydrate crystal. The research in this direction was initially driven by the important experimental observation of Okuchi et al.^989^ The authors used a pulsed-field gradient NMR method (at high H_2_ pressures), and reported that if the pressure on the binary THF + H_2_ hydrate phase is relieved for temperatures 250 K or higher, the H_2_ guest molecules can diffuse out of the hydrate crystal while the hydrate can still remain stable. On the other hand, upon reversal of the process (i.e., re-pressurisation), H_2_ can reversibly fill some of the empty S cages of the THF hydrate by diffusing through the cage faces of the intact hydrate crystal. Okuchi et al.^989^ reported experiments for H_2_ diffusivity in hydrates for four different temperatures (250, 255, 260, and 265 K) and H_2_ pressures in the range 1–20 MPa. The experiments (see Fig. 62) show that the diffusivity decreases as pressure increases. The decrease is attributed to the hypothesis that H_2_ transport occurs only when there is a vacant guest site in the neighboring cage. Recall that at high pressures the cage occupancies increase (see Section 4.8.5), therefore the number of empty cages is reduced. The authors further suggested that the translation of the H_2_ through pentagonal H_2_O rings between the 5^12^ cages is the most likely path for the activation barrier since all 5^12^6^4^ cages are occupied by THF. Note, however, that the NMR experiments reported by Senadhere and Conradi^990,991^ failed to confirm the previous observations of Okuchi et al.^989^ for the H_2_ diffusivity via the S cages.

**Figure 62:**
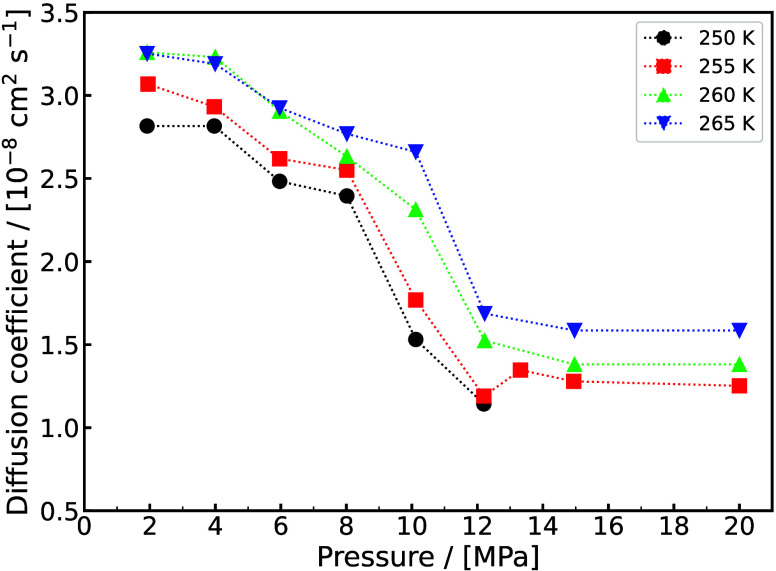
Diffusion coefficient of stored hydrogen in the hydrate as a function of pressure for different temperatures. The data are taken from Okuchi et al.^989^ The dotted lines are drawn to guide the eye.

The high mobility of H_2_ molecules within the sII S cages has been demonstrated by the inelastic neutron scattering (INS) experiments of the H_2_ + THF mixed hydrate reported by Choi et al.^992^ The authors argued that the 5^12^ cages alone, form a network of corner–sharing tetrahedral. The channels connecting the S cages are shown in Fig. 63. It is evident that the neighboring S cages line up in a [101] and symmetrical directions to form linear tunnels via which H_2_ diffuses, provided unoccupied neighboring cages are present. Information of such diffusion jumps are contained in quasi-elastic neutron scatter (QENS) signals. At low temperatures (i.e., *T* < 60 K), H_2_ molecules have not enough energy to overcome the potential barrier between the S cages. However, a sudden signal jump is observed between 60 and 65 K, persistent also at higher temperatures. This is a clear indication for the beginning of H_2_ diffusion via the [101] direction, where the S cages are aligned (i.e., H_2_ molecules have gained enough thermal energy to overcome the barrier and cross the pentagonal face connecting the neighboring S cages).

**Figure 63:**
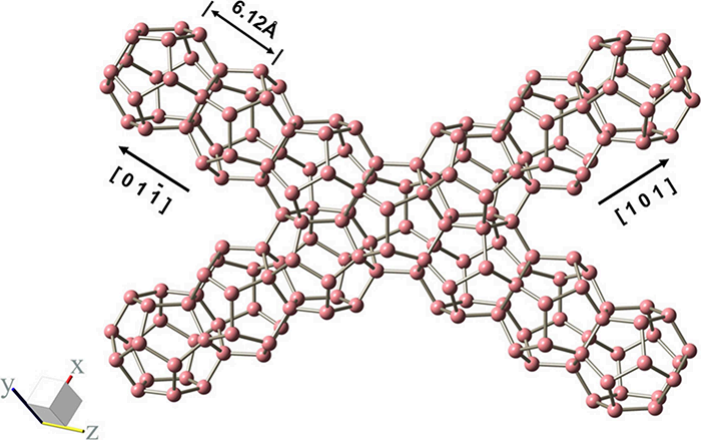
Channels formed by 5^12^ cages. Each S cage is connected to six other S cages through pentagonal faces.^992^ [The figure is reprinted from International Journal of Hydrogen Energy, Vol 35, Yong Nam Choi et al., Dynamics of hydrogen molecules in the channels of binary THF-H_2_ clathrate hydrate and its physicochemical significance on hydrogen storage, 13068–13072, Copyright (2022), with permission from Elsevier].

In a subsequent experimental study using Raman, Strobel et al.^993^ proposed an alternative scheme: Only the 5^12^6^4^ cages participate in the H_2_ diffusion via the hexagonal faces. Fig. 64 is a schematic of a portion of the sII hydrate unit cell that shows the hexagonal face-sharing L cages. The proposed scheme takes into account the DFT calculations of Alavi et al., which are discussed further, later in this Section (i.e., barriers for H_2_ diffusion equal to 25–29 kcal/mol and 5–6 kcal/mol for the S and L cages respectively).

**Figure 64:**
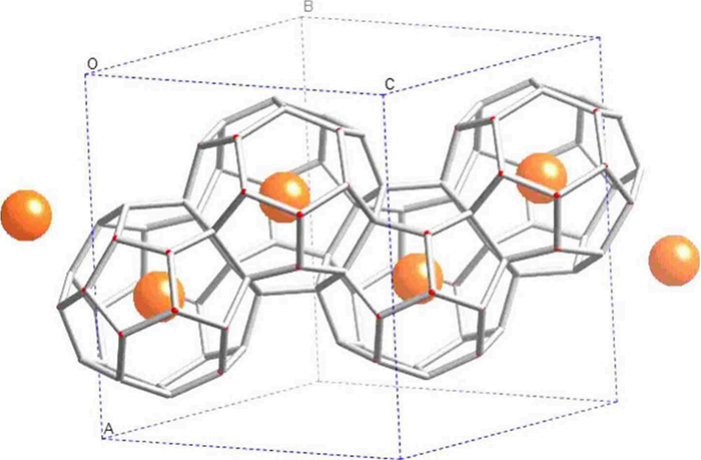
Portion of sII unit cell showing hexagonal face-sharing large cavities.^993^ [The figure is reprinted from Journal of Chemical Physics, Vol 130, Strobel and Dendy, Raman spectroscopic studies of hydrogen clathrate hydrates, 014506, Copyright (2009), with permission from AIP Publishing].

Russina et al.^994^ performed neutron scattering experiments of mixed H_2_ + THF. The authors reported significant H_2_ diffusive mobility in the L cages even at 10–50 K. Contrary, H_2_ confined in S cages were shown to be trapped at the center of the cages and, thus, were available for long range diffusion, independent of the activation barriers for the inter-cage jumps. Pefoute et al.,^995^ based on an INS study, reported that under equilibrium conditions, a single H_2_ molecule occupying the S cages of the binary THF + H_2_ sII clathrate hydrate does not diffuse outside of its cage on pico-second time-scales (i.e., an observation opposite to Choi et al.^992^). Conversely, Mulder et al.^965^ using neutron diffraction experiments, reported a value of H_2_ diffusion in the S cages of the binary THF + H_2_ hydrate equal to “ . . .at least 10^–11^ cm^2^/s at 264 K”, inferred from the time required to enter and leave hydrate crystallites with size equal to 0.4 *μ*m.

Parallel to the experimental work in this field, molecular simulation has played a pivotal role. Frankcombe and Kroes,^934^ performed equilibrium MD simulations, and showed that H_2_ molecules in the L cages can readily diffuse through the hexagonal faces, and ultimately, reach the bulk gas phase in contact with the hydrate structure. Nevertheless, H_2_ guests in the S cages have high energy barriers to migration, and thus, do not diffuse outside their cages. In a similar MD study, Cao et al.^996^ performed MD simulations to study the mixed H_2_ + THF system. The findings showed that H_2_ intercage migration from S cages occurs only during double occupancy of the S cages, while no H_2_ migration occurs during single H_2_ occupations of the S cages.

Gorman et al.^997^ reported MD simulations of the pure H_2_ and binary THF + H_2_ sII clathrate hydrates at 50 bar and temperatures up to 250 K. The authors detected diffusion of H_2_ molecules only when configurations with 2 H_2_ in the S cages were used. The S cages were observed to temporarily expand through a “breathing motion” (i.e., one of its pentagonal faces opened temporary) as one H_2_ guest migrated to a neighboring cage. The cage structure remained intact during the migration process. Gorman et al. claimed that this migration exhibits an approximate Arrhenius temperature dependence in the range 200–250 K.

Iwai and Hirata^998^ performed MD simulations of THF + H_2_ clathrates with different H_2_ occupancies in the S and L cages, at 300 and 310 K, and 10.1 MPa. The simulations revealed migration of H_2_ molecules between S cages and L cages. However, it should be noted that the observed diffusion between cages may be related to the high temperature at which the simulations were performed, which is likely outside the stability range of the binary hydrate (i.e., possible occurrence of hydrate dissociation). Geng et al.^999^ also observed the migration of H_2_ in the pure H_2_ and binary THF + H_2_ hydrates using equilibrium MD simulations. The authors concluded that these migration phenomena lead to the hydrate decomposition process. The MD data also showed that the diffusion of H_2_ is always larger than that of H_2_O molecules. Contrary, THF molecules encaged in the 5^12^6^4^ cavities perform as stabilisers because of the high interaction energy barrier restricting the motion of THF molecules.

Luis et al.^936^ investigated the effects of externally–applied static electric fields on the H_2_ diffusion in sII hydrates. The authors reported that the diffusion of H_2_ molecules occurs only through the L cages, while the H_2_ molecules diffuse, with a slight preference, in the same direction of the applied electric field.

Hasegawa et al.^1000^ performed equilibrium MD simulations of sII mixed H_2_ + SF_6_ hydrates, and reported that the diffusion of H_2_ molecules between L cages was found to occur more frequently. However, the presence of SF_6_ molecules in the L cages was proved to inhibit H_2_ diffusion. Therefore, the authors recommended that an optimal number of L cages should be occupied by SF_6_ molecules as a means to increase the H_2_ storage capacity of the mixed hydrate. Furthermore, Hasegawa et al. reported that a partial breaking of the hydrate structure was required to make room for the H_2_ to diffuse through the open pentagonal ring in the case that a H_2_ molecule diffused to/from an S cage. By contrast, no cage distortion was observed in H_2_ diffusion via a hexagonal ring from an L cage. Hasegawa et al.^1000^ also performed MD simulations of sII mixed H_2_ + tert–butylamine hydrate, where the tert–butylamine molecules occupied the L cages. The authors reported that more H_2_ molecules diffused into the hydrate as a result of a less stable, and partially distorted, hydrate structure, which created enough space for H_2_ molecules to move between adjacent cages.

Zhang et al.^594^ studied the growth of mixed H_2_ + CH_4_ hydrate with MD simulations, and observed that H_2_ can hop between the 5^12^6^4^ cages through the six-membered rings connecting the cages without any distortion of the cages, even for cases that the L cages are occupied by CH_4_. The observation of the simultaneous presence of two different guests (i.e., H_2_ and CH_4_) within the same L cage is of utmost importance since it can have a significant effect on the storage capacity of the hydrate. The possibility of the simultaneous occupancy of the L cages by two different guests was originally reported by Papadimitriou et al.^1001,1002^ for the case of the mixed sII He + THF, based on observations from GCMC simulations. Subsequently,^1003^ reported similar observations, based on experiments using *in situ* Raman spectroscopy for the mixed sII H_2_ + Ar hydrates. Waage et al.^1004^ performed MC simulations, which showed that H_2_ molecules are capable of coexisting, even in S cages, with any of the other molecules (i.e., CH_4_, CO_2_, N_2_) examined in their study.

In a similar context, Harada et al.^1005^ reported MD simulations for the case of H_2_ hydrate with crystal structure C_1_, namely ice II filled with H_2_ molecules. The authors observed fast diffusion of the H_2_ molecules in the C_1_ crystal. The H_2_ molecules were observed to migrate (via a highly anisotropic in space diffusion process) only inside the tube–like voids of the C_1_ hydrate. Harada et al. also pointed out the importance of the existence of defects or vacancies in the tube–like voids for H_2_ diffusion to occur. In the absence of vacancies no diffusion was observed. In a related study, Arman and Nugroho^1006^ reported MD simulations for the case of H_2_ hydrate with structure C_2_, namely ice I_*c*_ filled with H_2_ molecules. Additional MD simulations of anomalous H_2_ diffusion in various ices have been performed by Smirnov and Stegailov.^1007^

Except from classical MD, other atomistic-scale simulations have been conducted to study the diffusion of H_2_ in hydrate systems including, but not limited to the following: Alavi and Ripmeester^1008^ performed DFT calculations at the B3LYP and MP2 levels of theory to compute the energy required for the H_2_ molecules to migrate through a hexagonal face of the L-type cage or a pentagonal face of the S-type cage, considering both parallel and perpendicular configurations of the H_2_ molecules with respect to the examined face. They reported energy barriers to diffusion equal to 5–6 kcal/mol and 25–29 kcal/mol for the L and S cages, respectively.

The calculation of the free–energy barrier for H_2_ diffusion between cages has been been achieved using methods such as PIMD^1009^ and ab initio simulations. Typical examples can be found in the works of Trinh et al.,^182^ Cendagorta et al.,^366^ and Burnham et al.^1010–1012^ These alternative approaches are especially important at low temperatures, where H_2_ molecules are affected by quantum effects.

The molecular simulations by Trinh et al.^182^ demonstrated the importance of cage flexibility and cage occupancy on the diffusion of H_2_ between cages. Furthermore, they showed that the multiple occupancy of cages helps reduce the free energy barrier for H_2_ hopping between cages. Cendagorta et al.^366^ used the Ring Polymer Molecular Dynamics (RPMD) rate theory. This work was motivated by the fact that the energy barrier to hopping is significant compared to the thermal energy. Therefore, H_2_ diffusion is a rare event process, and thus, amenable to computation using rate theory. The PIMD simulations by Burnham and English^1010^ for the case of H_2_ diffusion between two L hydrate cages at 200 K also yielded the quantum free–energy barrier higher than the classical one, by about 2 kJ/mol.

Waage et al.^1004^ performed MC simulations to calculate the free energy barriers associated with CH_4_, CO_2_, N_2_, and H_2_ hopping through rings of water between the cages of the sI hydrate structure. The authors considered intact cages, as well as cages with H_2_ vacancies. H_2_ was shown to be capable of diffusing out from both the S or L cages.

Ghaani et al.^942^ used non-equilibrium MD simulations to examine H_2_ diffusion during release/uptake from/into propane planar clathrate surfaces in the temperature range 180–273 K. The simulations revealed that H_2_ molecules exhibited higher movement during release studies (with hydrate being in contact with a vacuum) when compared with the uptake studies (with gas H_2_ molecules located on top of the hydrate structure).

English and Burnham^1013^ considered classical MD and PIMD simulation to study the intra-cage behavior of H_2_ and D_2_ molecules in doubly occupied L cages of sII hydrates at 100 K. Krishnan et al.^1014^ examined transient, non-equilibrium inter-cage hopping, and presented a diffusional activation energy for the four nominal L cage occupancies (1 up to 4) using leakage–rate fits. Markov-chain models were utilized to study the inter-cage hopping of H_2_ and D_2_, and expressed at different temperatures and large-cage occupancies. Furthermore, the authors computed the free energy of guest “binding” in the L and S cages for all of the occupancies. Krishnan et al.^1015^ conducted micro-second-long, non-equilibrium MD simulations, in the temperature range 140–180 K, to elucidate mechanistic effects of electric–field on guest diffusivity control in hydrates. It was found that a judicious selection of the electromagnetic fields (i.e., in the microwave frequency range), could act towards the dramatic enhancement of the H_2_ release rate, without any breakup of the hydrate lattice itself. This is an important aspect for the kinetic control of large-scale H_2_ storage systems. Krishnan et al.^1016^ reported MD calculations exploring both H_2_ and D_2_ hydrates for cage occupancies and their self-diffusivity via inter–cage hopping. The authors studied these hopping phenomena as a function of temperature using a Markov chain model.

### 4.9 Interfacial Tensions of Aqueous Hydrogen Systems

Computing and analyzing the interfacial tension (IFT) between H_2_ and brine systems is crucial for the effective and safe implementation of UHS in geological formations, such as saline aquifers. IFT directly influences the capillary forces at the interface between H_2_ and reservoir fluids, governing H_2_ migration, trapping efficiency, and retention capacity within subsurface reservoirs. Accurate knowledge of IFT allows precise prediction and optimization of storage capacities and operational efficiencies, ensuring efficient H_2_ injection and withdrawal. IFT values, in addition, depend on thermodynamic and chemical conditions, including temperature, pressure, salinity, ionic composition, and the presence of cushion gases like CO_2_ and CH_4_, making its study essential for realistic reservoir modeling and design of storage strategies. Accurate IFT data support safety and risk mitigation by evaluating the sealing capability of caprocks, critical for preventing H_2_ leakage. Given the challenges associated with experimental IFT measurements under reservoir conditions, MD simulations offer valuable atomic-level insights and extensive predictive ability. Furthermore, understanding IFT helps optimize cushion gas composition and injection strategies, enhancing overall storage performance. The interplay between IFT and rock wettability significantly impacts capillary pressures, determining H_2_ stability and displacement behavior in porous formations. Therefore, a comprehensive examination of IFT is fundamental to ensuring secure, efficient and large-scale storage H_2_ in geological reservoirs. For a thorough introduction on the role of molecular simulation for designing and optimizing UHS the reader is referred to Section 2.3.4. Details on the computation of IFT via MD are provided in Section 3.2.16. A review of relevant studies is provided below.

van Rooijen et al.^630^ computed IFTs of H_2_ gas in contact with aqueous NaCl solutions, self-diffusivities and solubilities of H_2_ in aqueous NaCl solutions using MD simulations at conditions relevant to UHS and H_2_O electrolysis. The IFTs were computed for temperatures of 298 to 523 K, pressures of 1 to 600 bar, and molalities of 0 to 6 mol NaCl/kg H_2_O using the TIP4P/2005 water model, Madrid-2019 force field for NaCl, and Vrabec force field for H_2_ (for details on these force fields see Section 3.1). The results showed that IFTs do not exhibit significant pressure dependence, increase linearly with salt molality (by ca. 1.44 mN/m per mol NaCl/kg H_2_O), and decrease non-linearly with temperature. This behavior is explained by the fact that IFT is related to the density difference between the two phases, with the low density of H_2_ relative to water resulting in minimal pressure effects, while the increased density of saline water and the arrangement of ions at the interface (with anions being depleted from the bulk phase and cations being absorbed into it) strengthen the H_2_ bond network of H_2_O, leading to increased IFT. Temperature has the strongest influence on the IFT, causing a non-linear decrease due to its effect on the density difference between H_2_ and aqueous solutions. The computed IFTs were compared with available experimental data from Hosseini et al.,^1017^ showing excellent agreement with average deviations lower than 6.4% across the range of conditions studied. The authors proposed an engineering equation for IFT as a function of temperature and molality: $\gamma =c_{1}+c_{2}m+c_{3}T^{c_{4}}$ , valid for temperatures of [298, 523] K, pressures of [1, 600] bar, and molalities of [0, 6] mol NaCl/kg H_2_O. The IFT data generated in this work is particularly valuable because experimental measurements for the H_2_/H_2_O/NaCl system are scarce, especially at high pressures and temperatures, which are highly relevant to H_2_ technologies.

Omrani et al.^1018^ investigated the IFT, density, and molecular distribution properties of the H_2_ – brine systems with various salts including Na^+^, K^+^, Ca^2+^, and Mg^2+^, under reservoir conditions. IFT was computed as a function of pressure (1–30 MPa), temperature (298–373 K), and salt molality/composition (up to 5.02 mol/kg for NaCl and up to 1.91 mol/kg for other salts). The results show that IFT decreases with increasing temperature, slightly decreases with increasing pressure, and increases with higher salinity, with values ranging roughly from 55.85 to 77.54 mN/m, depending on the conditions. Physically, these trends are explained by the interfacial molecular structure where higher temperature broadens the interfacial width and enhances H_2_ – H_2_O mixing, reducing IFT, while increased salinity enriches H_2_ accumulation at the interface, thinning the interface and increasing IFT. Temperature is identified as the strongest factor influencing IFT, followed by salinity, with pressure having a comparatively minor effect. The MD simulation results for IFT showed excellent agreement with available experimental data, with deviations less than 3% when using the combined Marx force field for H_2_, TIP4P/2005 model for H_2_O, and Smith-Dang parameters for Na^+^ and Cl^–^. A general correlation for IFT as a function of reduced temperature, density difference between H_2_ and brine phases, and NaCl mole fraction was developed using genetic programming, achieving high accuracy with *R* ^2^ = 0.9783 and absolute average relative deviation below 1%. The effect of cation type was also shown to be significant, with CaCl_2_ yielding the highest IFT values and K^+^ the lowest at comparable concentrations, attributed to differences in ion hydration enthalpy and interfacial distribution.

Xie et al.^1019^ focused on accurately predicting the IFT of H_2_ + CO_2_ gas mixtures in contact with H_2_O and brines containing Na^+^ and Cl^–^ ions over a wide range of temperatures, pressures, gas compositions, and salinities. Molecular interactions were analyzed to elucidate the underlying mechanisms affecting IFT, while and ML approach was used to develop a general predictive equation. The parameters used to compute IFT included temperatures from 298 to 373 K, pressures in the range of 50 to 400 bar, gas compositions expressed as H_2_ mole fraction, and brine salinities up to 3.15 mol/kg. The IFT was shown to decrease with increasing pressure and CO_2_ content, while increasing salinity raised the IFT, with values ranging approximately between 20 and 60 mN/m depending on conditions. These trends arise from the interplay between molecular mobility of gas and water molecules (entropy) and intermolecular interactions primarily between gas molecules and water/brine ions (enthalpy). The higher pressure enhances gas solubility in the aqueous phase, which reduces the IFT, whereas the presence of salt ions (Na^+^ and Cl^–^) increases electrostatic and van der Waals interactions at the interface, thereby strengthening intermolecular forces and increasing IFT. Among the factors studied, pressure and gas composition exhibit the strongest influence on IFT behavior under the chosen conditions (relevant to UHS), with temperature showing a less dominant but still significant effect. MD simulations of IFT were validated against extensive experimental data, showing good agreement with average relative errors around 4.4%. A ML-based polynomial equation was developed to predict IFT as a function of temperature, pressure, H_2_ mole fraction, and salinity, achieving high accuracy with *R* ^2^ = 0.972 on testing data. This equation outperforms previous empirical models, especially for ternary (H_2_ + CO_2_)-water/brine systems, and is suitable for reservoir simulation applications.

Yang et al.^602^ computed IFT, contact angles, adhesion tension, density distributions, and relative adsorption in multiphase systems of H_2_ + H_2_O with silica or kerogen under various conditions. IFTs, computed via MD simulations (INTERFACE force field for H_2_ and TIP4P/2005 for H_2_O) and density gradient theory (with PC-SAFT EoS), range from approximately 25 to 72 mN/m as functions of pressure (1 to 160 MPa) and temperature (298 to 523 K). A general outcome, IFT was shown to decrease with increasing pressure and temperature, but above about 448 K and 60 MPa, IFT increased with pressure due to an inversion of H_2_ relative adsorption: at low *T* and *P*, adsorption is positive (enrichment at the interface), decreases past a pressure threshold, and becomes negative at high *T* (depletion at the interface). Temperature most strongly influences IFT by altering molecular distributions and thermal fluctuations. The INTERFACE + TIP4P/2005 combination of force fields accurately reproduces the surface tension of water, and matches experimental IFTs within 1.7 - 7.4 mN/m, while density gradient theory with PC–SAFT reproduces the same trends seen in both simulations and experiments. The authors note that the value of IFT in H_2_+H_2_O is close to the surface tensions of pure water, and higher than in CO_2_+H_2_O, CH_4_+H_2_O, or N_2_+H_2_O computed in other studies not reviewed here.

Doan et al.^1020^ studied IFT and adsorption in water–H_2_, CH_4_, and CO_2_ mixtures. IFT was computed at 1 to 70 MPa and at 300 and 323 K for pure water and binary gas mixtures. IFT was generally shown to decrease with pressure and temperature; for instance, *γ*(CO_2_ – H_2_O) value fell from ca. 62 to ca. 34 mN/m, *γ*(CH_4_ – H_2_O) from ca. 64 to ca. 53 mN/m, while *γ*(H_2_ – H_2_O) remained equal to 62-63 mN/m, showing greater temperature sensitivity. Thus, H_2_ yielded the highest IFT and CO_2_ the lowest. This decrease was attributed to enhanced gas adsorption at the water interface, especially stronger CO_2_ - water interactions compared to CH_4_ or H_2_, though detailed molecular mechanisms remain unexplored. The authors reported that pressure dominates IFT behavior, decreasing sharply up to 50 MPa and plateauing above, while temperature has a lesser effect, becoming negligible at high pressure. The computed IFTs agree well with experiments for CO_2_-H_2_O and CH_4_-H_2_O, though underestimating low-pressure values by up to 10%, and underestimating H_2_-H_2_O by 10 to 14%, likely due to the choice of force fields, inaccurate mixing rules for the intermolecular interactions, or system-size limitations. MD simulations show H_2_ in ternary H_2_ - CH_4_ - H_2_O mixtures increases IFT relative to CH_4_ - H_2_O and CO_2_ - CH_4_ - H_2_O, owing to weaker H_2_ intermolecular forces and lower interfacial adsorption.

Doan et al.^1021^ computed IFT and flow properties of water–H_2_ mixtures with cushion gases CO_2_, N_2_, and CH_4_ under geological storage conditions. IFT was computed as a function of pressure [1, 70] MPa, temperature [300, 343] K, and cushion-gas mole fractions [10 to 90]%. For H_2_/CO_2_/H_2_O, H_2_/N_2_/H_2_O, and H_2_/CH_4_/H_2_O systems, IFT decreased from ca. 64 mN/m at low pressure to ca. 42 mN/m at 70 MPa at higher pressures and temperatures. Higher H_2_ content raised IFT: N_2_-containing mixtures yield the highest IFT, while CO_2_-containing mixtures yield the lowest IFT at fixed conditions. These trends arise from gas adsorption at the H_2_O interface i.e., heavier CO_2_ reduces IFT due to stronger inter-molecular interactions and higher adsorption, CH_4_ moderately lowers IFT, and the light, inert N_2_, yields higher IFT. Pressure was shown to most strongly affect IFT, which dropped sharply up to ca. 30 MPa, and then reached a plateau for all CO_2_, N_2_, and CH_4_ mixtures across the 300 - 343 K temperature range. Temperature caused a gradual decrease in IFT (at fixed pressure), but had a weaker effect. The computed IFTs for H_2_/CO_2_/H_2_O agree with experiments within 12% at low pressures. For H_2_/N_2_/H_2_O and H_2_/CH_4_/H_2_O systems, the available experimental data are limited, making the molecular simulation predictions valuable.

Chen et al.^604^ performed molecular simulations to study the IFT, surface excess, bubble evolution, and contact angle of CO_2_, H_2_, and their mixtures with H_2_O or brine (Na^+^, Cl^–^) at 10 MPa and temperatures ranging form 300 to 400 K. CO_2_/H_2_ mixtures included H_2_/CO_2_ ratios of 1:10, 2:10, 3:10 (CO_2_ – rich) and their inverses (H_2_ – rich). IFT was computed isobarically (10 MPa) over 300 to 400 K with 4% NaCl brine. In H_2_ – rich mixtures, IFT decreased roughly linearly from ca. 65 mN/m at 300 K to ca. 50 mN/m at 400 K in both pure H_2_O and brines. CO_2_ – rich systems showed non-monotonic temperature dependence: IFT rose to ca. 20 mN/m at the range 320 - 360 K, and at higher temperatures decreased. The authors qualitatively attributed this peak to phase transitions and temperature-dependent surface adsorption, without though providing a detailed mechanistic explanation. Brine was shown to increase IFT by a few mN/m relative to pure H_2_O. CO_2_ significantly reduced IFT compared to H_2_, which has minimal impact. This is due to the stronger electrostatic and quadrupolar interactions of CO_2_ with H_2_O, influencing the anisotropy of the tensor of the virial pressure, a concept noted but not elaborated further by Chen et al.^604^ The weak van der Waals interactions of H_2_ cause smaller IFT changes. The temperature change from 300 to 400 K, yielded a 10 mN/m IFT decrease in H_2_-rich systems and non-monotonic variation of 5 mN / m in CO_2_ – rich systems, signifying temperature as the prime IFT variable. MD results agreed with experimental CO_2_ – H_2_O and H_2_ – H_2_O data (298 - 448 K, ≤ 45 MPa). The experimental data cover pure gas-water, but the trends of the mixture were shown to be consistent with the behavior of the extrapolated pure components. The simulations indicated linear temperature dependence in systems rich in H_2_, and non-monotonic in systems rich in CO_2_, with a consistently increasing IFT of brine.

Chen and Xia^605^ computed transport properties, wettability, IFT, and surface adsorption of H_2_, CO_2_, and CH_4_ interacting with H_2_O and silica under reservoir conditions. The IFT calculations were carried out at a fixed temperature of 320 K and pressures up to 60 MPa, considering pure water and silica substrates. Across all conditions, the IFT follows the order γ_CO_2_–H_2_O_ < γ_CH_4_–H_2_O_ < γ_H_2_–H_2_O_. In particular, the CO_2_-water IFT dramatically decreased by ca. 42% at 10 MPa before plateauing, the CH_4_ – water IFT decreased more gradually with pressure, and the H_2_ – water IFT remained nearly constant. These trends arise from differences in density and molecular interactions i.e., the higher density and quadrupole moment of CO_2_ enhance its surface activity, while the low density of H_2_ and weaker van der Waals forces lead to minimal variation in IFT. The primary factor influencing IFT is the density difference between the gas and water phases, which governs interfacial molecular interactions. MD-computed IFTs agree well with experimental data for CO_2_ and CH_4_ systems. For H_2_, the simulations were shown to slightly underestimate the experimentally measured IFTs, but correctly capture the qualitative behavior. No significant pressure dependence of IFT for H_2_ is observed in simulations, consistent with experimental observations of only slight decreases.

The study by Adam et al.^1022^ reported data on IFT, structure, and solubility for UHS-relevant brine (NaCl) and gas mixtures of H_2_, CO_2_, and CH_4_. IFTs were computed for pressures of 10–60 MPa, temperatures from 323.15 to 388.15 K, and salt concentrations 10-25 wt%, covering both pure gas-brine and mixed gas-brine systems. Pure gases included brine/H_2_, brine/CO_2_, and brine/CH_4_, while mixtures considered were ternary brine/H_2_/CO_2_ and quaternary brine/40% mol H_2_/60% mol cushion gases (CO_2_ or CH_4_). Pure brine/H_2_ IFT remained nearly constant (around 59 mN/m) across pressures, whereas brine/CO_2_ IFT decreased markedly from 44.4 to 30.6 mN/m, and brine/CH_4_ showed a minor drop (53.6 to 51.8 mN/m). Mixed gas systems with CO_2_ exhibited stronger pressure dependence; brine/40% mol H_2_/60% mol CO_2_ IFT decreased about 14.6%, compared to a smaller 7.4% decrease with CH_4_. Temperature significantly reduced IFT across all systems: brine/H_2_ decreased roughly 20% (67.4 to 54.3 mN/m), brine/CO_2_ from 37.1 to 31.6 mN/m, and brine/CH_4_ from 57.5 to 50.2 mN/m. Similar trends occurred in mixed systems; brine/40% mol H_2_/60% mol CO_2_ IFT dropped by 8 mN/m, and brine/40% mol H_2_/60% mol CH_4_ by 12 mN/m. Higher CO_2_ content lowered temperature sensitivity due to stronger molecular interactions requiring higher thermal energy to disrupt these intermolecular bonds. Radial distribution function analysis revealed weak hydrogen-bonding between CO_2_ oxygen and H_2_O hydrogen atoms at the interface, leading to CO_2_ clustering and reduced IFT as its mole fraction increased. Increasing salt concentration raised IFT in all systems via the salting-out effect, as ions attracted water molecules, reducing water-gas interactions. For example, increasing NaCl from 10 to 25 wt% raised IFT by approximately 3 mN/m for brine/H_2_, 4 mN/m for brine/CO_2_, and 7 mN/m for brine/CH_4_. Pressure had a moderate impact on IFT, notably in systems with higher CO_2_, whereas temperature and salinity exerted stronger effects. The computed IFT values closely agree with experimental data, validating the methodology and force fields used by Adam et al.^1022^. Engineering correlations using polynomial functions were developed to predict IFT based on pressure, temperature, CO_2_ concentration, and salinity. It is important to note that the functional form (i.e., actual cubic polynomial) of the devised equations and the coefficients were not provided by the authors, hindering the wide, practical use of the presented model.

#### 4.9.1 Handling Long-Range Dispersion Interactions

Transitioning now from the specific findings presented by the studies reviewed earlier, we collectively address the approaches followed for calculating IFTs. A primary challenge associated with the slab method of determining IFTs in MD simulations is accurately handling long-range dispersion (LJ) interactions. Two principal strategies commonly applied are semi-analytic tail corrections and explicit mesh-based methods. Tail corrections adjust IFT values post-simulation, following the approach developed by Blokhuis et al.^1023^ and elaborated by Allen and Tildesley^52^, and can significantly impact computed IFT values, sometimes by up to ca. 35% for LJ fluids. Alternatively, explicit mesh-based methods, such as smooth Particle Mesh Ewald (sPME)^1024^ and Particle-Particle Particle-Mesh (PPPM),^1025,1026^ directly compute long-range dispersion analogously to electrostatic Ewald summation, as comprehensively reviewed by Goujon et al.^1027^ Sega and Dellago^1028^ compared these approaches across seven water models, demonstrating that explicit mesh-based methods yield more accurate IFT predictions and correctly reproduce liquid and vapor densities. Furthermore, including the full dispersion interactions explicitly shifted the critical temperature of water upwards, a crucial thermodynamic effect not captured by semi-analytic tail corrections, highlighting the importance of explicitly incorporating long-range dispersion interactions.

Several studies have applied these methodologies specifically to H_2_-water and H_2_-brine systems, adopting various approaches to computing the long-range dispersion. For instance, van Rooijen et al.^630^, Xie et al.,^1019^ and Yang et al.^602^ used explicit mesh-based methods (PPPM^1025^ or PME^1029^), with Yang et al.^602^ also validating the improved accuracy against NIST data when using PPPM. Omrani et al.^1018^ truncated dispersion interactions without applying tail corrections but mitigated potential underestimations in IFT by using an unusually large LJ cutoff (2.9 nm), requiring larger simulation cells and greater computational effort. Adam et al.^1022^ also truncated dispersion interactions at 1.28 nm without long-range dispersion interactions. Doan et al.^1020,1021^ used semi-analytic tail corrections proposed by Blokhuis et al.,^1023^ Chen et al.^604^ and Chen and Xia^605^ use truncated LJ interactions without explicit tail corrections, potentially underestimating IFT values due to the omission of long-range dispersion effects. Consequently, differences in IFT predictions among these studies can often be attributed to varying treatments of long-range dispersion interactions. Although semi-analytic tail corrections provide computational efficiency, they may overlook important thermodynamic effects, whereas explicit mesh-based methods ensure higher accuracy at the expense of greater computational resources. Thus, consistently addressing long-range dispersion interactions is vital for obtaining reliable IFT predictions in H_2_-water and H_2_-brine systems.

Although here we focus on long-range dispersion in simulations of interfacial tension for H_2_-water, the same considerations arise in nanoporous frameworks (e.g., MOFs, zeolites, COFs). Whether to apply homogeneous long–range (tail) corrections in crystalline hosts remains debated in the literature. One view argues against the use of these corrections because the standard assumption that the radial distribution function approaches unity beyond the interaction cutoff radius often fails in ordered solids (the radial distribution function can remain oscillatory and even peak near the cutoff). Thus, applying a homogeneous correction may introduce spurious energetic contributions.^1030^ Moreover, in crystalline materials, the radial distribution function is inherently direction dependent. The opposing view recommends applying tail corrections to solid–fluid and fluid–fluid terms to reduce sensitivity to truncation, and to enable more consistent cross–study comparisons; substantial effects on Henry coefficients and loadings have been reported by Siperstein et al.^1031^ and Jablonka et al.^1032^

Tail corrections are, ultimately, a modeling choice. In practice, we suggest: (i) following the force field as originally parametrized (its interaction cutoff radius and any prescribed tail correction), (ii) reporting these choices explicitly in the methodology section of the study, and (iii) where feasible, demonstrating robustness with brief sensitivity tests (for example, one can vary the interaction cutoff radius and toggle the correction on or off). As a way to sidestep the truncation/tail-correction decision entirely, one can use smooth finite–range pair potentials that vanish continuously, for example, the Wang-Ramírez-Dobnikar-Frenkel (“WF”) LJ-like potential^1033^ keeping in mind that using such potentials generally requires re–fitting parameters to reproduce the target adsorbate-framework interactions.

#### 4.9.2 Importance of Force Field Selection on Interfacial Tension Computations

As extensively discussed thusfar in this review, in MD simulations aimed at accurate predictions, selecting appropriate force fields to describe molecular interactions is essential. Omrani et al.^1018^ extensively explored various force fields for H_2_ including Vrabec,^48^ Hirschfelder,^321^ modified Silvera-Goldman,^335^ 3-site Alavi,^336^ Cracknell,^311^ and Marx,^310^ and for water TIP4P/2005,^305^ TIP4P-*μ*,^47^ TIP4P OPLS/AA,^360^ SPC/E,^346^ TIP3P,^300^ and TIP5P^359^ (see Sections 3.1.1 and 3.1.2). Omrani et al.^1018^ computed IFTs under pressures of 5 and 20 MPa and temperatures of 323 and 373 K, alongside multiple NaCl models including the ones by Smith and Dang,^374^ Joung and Cheatham,^371^ the Madrid-2019,^1034^ and from Loche et al.^1035^ The combination of Marx^310^ with TIP4P/2005 ^305^ and Smith and Dang^374^ provided the best agreement with the experimental IFT data of Hosseini et al.,^1017^ with deviations being consistently lower than 5%. Notably, Omrani et al.^1018^ did not include the widely-used IFF^313^ force field for H_2_, yet emphasized the superior performance of TIP4P/2005, aligning with earlier conclusions by Vega and Miguel.^1036^

Subsequent work by Yang et al.^602^ evaluated the performance of the Hirschfelder,^321^ IFF,^313^ and Alavi^336^ H_2_ models combined with the TIP4P/2005 ^305^ water model, showing that the IFF model is the most precise in reproducing experimental IFT values. Meanwhile, Xie et al.^1019^ tested various force field combinations, observing a consistent underestimation (18%) of IFT when using the SPC/E water model. Adam et al.^1022^ also indicated that SPC/E significantly deviated from experimental IFT data when compared to TIP4P/2005. For example, in the H_2_O/H_2_ system at 323 K and 5-20 MPa, SPC/E showed discrepancies of up to 15 mN/m, whereas TIP4P/2005 remained within ±0.15 mN/m. Similar trends were observed across brine/CO_2_, brine/CH_4_, and ternary brine/H_2_/CO_2_ systems, where TIP4P/2005 consistently reproduced experimental IFT values and pressure trends, while SPC/E underestimated IFT and failed to capture its pressure dependence. The results of Xie et al.^1019^ and Adam et al.^1022^ further highlight the critical role of the water force field, noting that variations between the IFF^313^ and Alavi^336^ H_2_ force fields were less significant compared to those arising from the choice of the water model. Although Yang et al.^602^ concluded that the Alavi force field failed to capture the pressure-dependent IFT trend, their conclusion was based on limited data, raising caution. In contrast, the more comprehensive study by Xie et al.^1019^ demonstrated that while the 3-site Alavi model slightly underestimates absolute IFT values at 323 K, it does correctly reflect the pressure dependence. Collectively, these studies emphasize that selecting an accurate water force field has a greater impact on IFT predictions than the specific choice of the H_2_ force field.

Another important detail to consider when performing MD simulations of non-homogeneous systems (e.g., gas/liquid systems used for the computation of the interfacial tension of H_2_/water) is the use (or not) of analytic tail corrections.^51,52^ Such corrections are only valid for systems that the radial distribution function converges to 1 at long distances (homogeneous gas and liquid phases).^51,52^ Therefore, tail corrections are usually not used in systems containing interfaces, since this choice will produce simulation artifacts.^1037,1038^ To minimize the error introduced from not considering the long-tail of the interactions potentials, cutoff radii larger than what the chosen force fields dictate can be used. However, such a choice will increase the computational requirements (since more interaction pairs will be considered in each timestep of the simulation), while it should be explicitly mentioned in the methodology section of the study to enable the reproducibility of the results. MD simulation software packages, such as LAMMPS^762^ and GROMACS,^476,477^ allow for the computation of the long-range LJ and electrostatic interactions with the PPPM^51^ method. For more details on this approach, the reader is referred to the studies by Isele-Holder and co-workers.^1039,1040^

#### 4.9.3 Comparison between Molecular Simulations and Experiments of Hydrogen–Water Interfacial Tensions

Fig. 65(a) shows a comparison between experimental H_2_ – H_2_O IFT data from Hosseini et al.^1017^ with fitted MD data using the engineering correlations proposed by van Rooijen et al.,^630^ Omrani et al.,^1018^ and Xie et al.^1019^ Notable deviations occur primarily below 325 K. Specifically, Xie et al.^1019^ calibrated their model over a temperature range of 298–373 K and pressures of 5–40 MPa, whereas van Rooijen et al.^630^ used a broader range of 298–523 K and 0.1–60 MPa. The weak pressure dependence of IFT observed experimentally by Hosseini et al.^1017^ is qualitatively captured by Xie et al.,^1019^ although their model exhibits roughly a 5% deviation and an overestimation of the pressure sensitivity, evidenced by a steeper slope. Conversely, the model by van Rooijen et al.,^630^ assuming pressure independence, exhibits a deviation of ca. 10% from experimental data below 325 K, but aligns well (within 5%) at higher temperatures. Omrani et al.^1018^ could not be directly compared due to their fit requiring gas-liquid density differences that were not provided. Fig. 65(b) shows a comparison of the experimental IFTs reported by Chow et al.^1041^ with predictions from van Rooijen et al.^630^ and Hosseini et al.,^1017^ both achieving better than 5% agreement at temperatures above 370 K. Since Chow et al.^1041^ also provided gas and liquid densities, Omrani et al.^1018^ could be evaluated, revealing the near pressure independence of IFT, consistently with experiments, except for pressures above about 40 MPa, for which a notable decrease is observed. Overall, within their respective calibration ranges, the fit functions from Omrani et al.^1018^ and Xie et al.^1019^ achieve agreement within 1–10% of experimental values, while van Rooijen et al.^630^ cover a broader range but remain within roughly 15%. Despite variability among MD-based predictions, the computed IFT values for H_2_ – H_2_O systems consistently fall within approximately 10–15% of experimental measurements from Hosseini et al.^1017^ and Chow et al.^1041^

**Figure 65:**
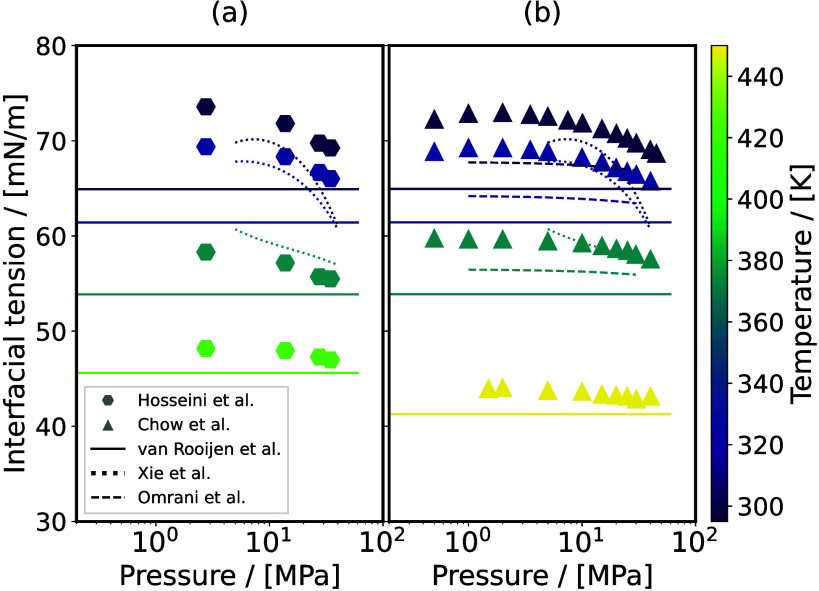
Interfacial tension of H_2_-H_2_O as a function of pressure. Comparison between experiments (from: (a) Hosseini et al.^1017^ and (b) Chow et al.^1041^ shown as markers) and engineering equations fitted with data from three different MD simulations­(from van Rooijen et al.^630^: solid line, Omrani et al.^1018^: dashed line, and Xie et al.^1019^: dashed dotted line). The colors of the markers represent the temperature at which the interfacial tension is reported. The MD simulation predictions are plotted within the range where the equation remains valid.

#### 4.9.4 Interfacial Tensions of Hydrogen–Brine Systems

Fig. 66 illustrates the IFTs of H_2_ – NaCl brine systems compiled from various studies (summarized in Table 19), collectively capturing key experimental observations such as the minimal sensitivity of IFT to pressure, the decreasing IFT with rising temperature, and increasing IFT with salt concentration. Fig. 66(a)-(d) predominantly show data points within pressure ranges of 1–50 MPa and temperatures of 300–400 K, relevant to UHS. For aqueous H_2_ – systems, there is notable consistency across multiple studies, largely attributable to the widespread adoption of the TIP4P/2005 water model. Although various H_2_ force fields (single-site versus multi-site) were used, the resulting discrepancies among the different studies^602,630,1018,1019^ remain modest. However, the presence of salt significantly increases variability among predictions. Notably, at 298 K and 10 MPa, reported IFT values for aqueous H_2_ systems without salt range from 64.9 to 68.2 mN/m across studies, whereas at a salt concentration of 3 mol NaCl/kg water yields IFTs in the range of 68.6 and 76.1 mN/m. While Omrani et al.^1018^ and Xie et al.^1019^ remain closely aligned, van Rooijen et al.^630^ deviate by ca. 5%, potentially due to their use of a single-site H_2_ model. Additionally, even studies using identical force fields for NaCl differ by around 5%, a discrepancy possibly intensified by the different choices for modeling long-range dispersion interactions relevant for correcting the IFTs (e.g., omitted in ref 1018). Overall, while TIP4P/2005 ^305^ seems to ensure consistent and reliable IFT predictions for aqueous H_2_, discrepancies emerge in H_2_ – NaCl brine simulations due to variations in H_2_ and salt force fields and methodological choices, such as neglecting long-range corrections. Despite these variations, most simulation predictions remain within a 5–10% margin, providing a reliable source of data for further experimentation, modeling, and industrial practice. Future refinements in force field development and selection, and accurate treatment of ionic interactions may further reduce these discrepancies and enhance predictive accuracy.

**Figure 66:**
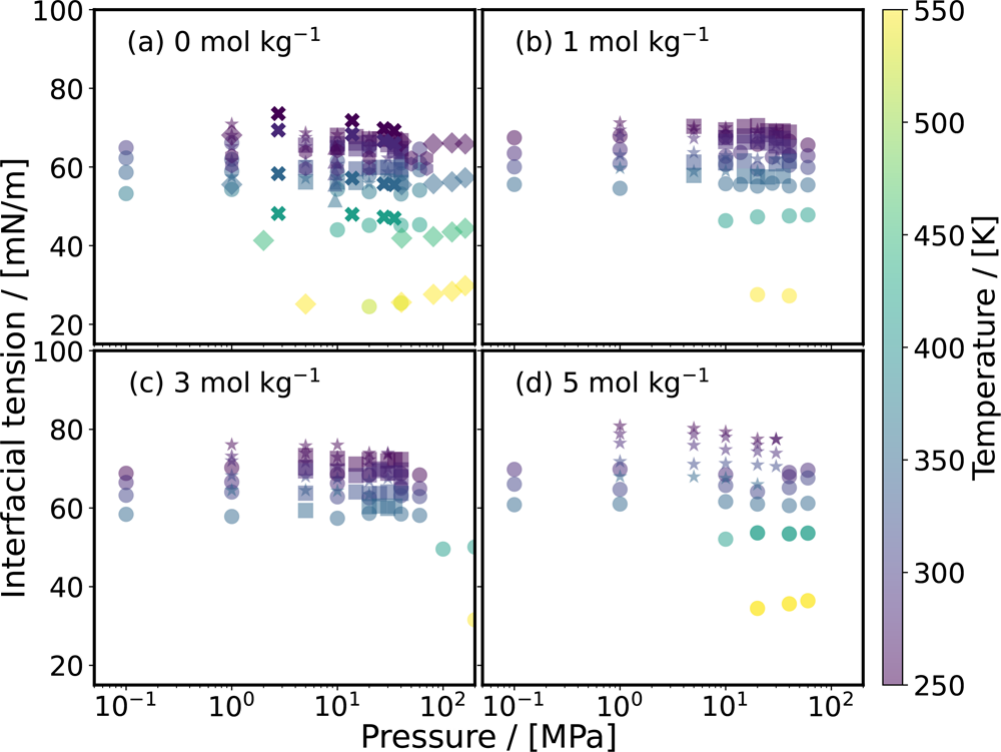
Interfacial tension of aqueous H_2_-NaCl mixtures as a function of pressure computed from MD simulations for salt concentrations of (a) 0 mol of NaCl/kg of H_2_O, (b) 1 mol of NaCl/kg of H_2_O, (c) 3 mol of NaCl/kg of H_2_O, and (d) 5 mol of NaCl/kg of H_2_O. The colors of the markers represent the temperature at which the interfacial tension is reported. The data are taken from van Rooijen et al.^630^ (circle), Omrani et al.^1018^ (star), Xie et al.^1019^ (square), Yang et al.^602^ (diamond), Doan et al.^1021^ (hexagon), and Chen et al.^604^ (triangle). To facilitate comparison between simulations and experiments for pure water-H_2_ system, the experimental data of Hosseini et al.^1017^ (shown with marker x) is added in subfigure (a).

**Table 19:** Summary of the MD Simulation Studies Investigating the IFTs of Hydrogen–Gas Mixtures in Contact with Water or Brine^a^

	System details		Thermodynamic conditions		
Study	Phases	Force fields	*p* / [MPa]	*T* / [K]	*c*_salt_ / [molal]
van Rooijen et al.^630^	H_2_(g)	Vrabec^48^	0.1 to 60	298 to 523	
	H_2_O(l)	TIP4P/2005 ^1042^	(Logarithmic)		
	Brine(l): Na^+^ and Cl^–^	Madrid-2019 ^1034^			0 to 5
Omrani et al.^1018^	H_2_(g)	Marx and Nielaba^310^	1 to 30	298 to 373	
	H_2_O(l)	TIP4P/2005 ^1042^	(Logarithmic)		
	Brine(l): K^+^, Na^+^ and Cl^–^	Smith and Dang^374,1043^			0 to 5
	Brine(l): Ca^2+^, Mg^2+^	Aqvist^1044^			0 to 5
Xie et al.^1019^	H_2_(g)	IFF^313^	5 to 40	298 to 373	
	CO_2_(g)	Zhu^926^			
	H_2_O(l)	TIP4P/2005 ^1042^	(steps of 5)		
	Brine(l): Na^+^ and Cl^–^	Smith and Dang^374,1043^			0 to 3
Yang et al.^602^	H_2_(g)	IFF^336^	1 to 160 MPa	298 to 523 K	0
	H_2_O(l)	TIP4P/2005 ^1042^			
Doan et al.^1021^	H_2_(g)	IFF^313^	1 to 70	300 to 343	
	CO_2_(g)	EPM2 ^419^			
	N_2_(g)	OPLS^360^			
	H_2_O(l)	TIP4P/2005 ^1042^			
Doan et al.^1020^	H_2_(g)	IFF^313^	1 to 70	300 and 323	0
	H_2_O(l)	TIP4P/2005 ^1042^			
Chen and Xia^605^	H_2_(g)	IFF^313^	1 to 60	320	0
	H_2_O(l)	TIP4P/2005 ^1042^			
Chen et al.^604^	H_2_(g)	IFF^313^	9.5	300, 400	2.22 (in unitls of molarity)
	CO_2_(g)	TraPPE^1045^			
	H_2_O(l)	TIP4P/2005 ^1042^			
	Brine(l): Na^+^ and Cl^–^	Madrid-2019 ^1034^			
Adam et al.^1022^	H_2_(g)	Yang^325^	10-60	323-388	1.9-4.75
	CO_2_(g)	TraPPE^1045^			
	CH_4_(g)	TraPPE^301^			
	H_2_O(l)	TIP4P/2005 ^1042^			
	Na^+^	Chen et al.^1046^			
	Cl^–^	Chen et al.^1046^			

a The salt concentrations in brine are reported in units of molality (mol salt / kg of H_2_O). g and l in the parentheses indicate gas and liquid phases, respectively.

#### 4.9.5 Interfacial Tensions of Gas Mixture–Hydrogen–Water Systems

Fig. 67 highlights the distinct differences in IFT behavior for gas mixtures involving H_2_/CO_2_ compared to those containing N_2_ or CH_4_. Specifically, the H_2_/CO_2_ mixture displays a notably steeper slope, indicating a more pronounced decrease in IFT with increasing pressure. This behavior primarily arises due to CO_2_ being near or above its critical point (7.3773 MPa and 304.15 K), where its density can fluctuate significantly, often changing by an order of magnitude within a narrow pressure interval. For instance, at 323.15 K, the density of CO_2_ can increase dramatically from around 100 kg/m^3^ at 5 MPa to roughly 1000 kg/m^3^ at 20 MPa. Such significant density changes drastically reduce the liquid-vapor density contrast, causing a marked reduction in IFT. In contrast, mixtures involving N_2_ or NH_4_ remain well within supercritical conditions over comparable pressure ranges, resulting in more moderate density variations and correspondingly flatter IFT profiles. Additionally, the presence of H_2_ does not significantly alter these general trends; the substantial decrease in IFT is predominantly driven by the supercritical transition of CO_2_. From a practical standpoint, e.g., for subsurface storage and enhanced oil recovery applications, these observations highlight the acute sensitivity of interfacial properties to pressure variations near the critical region of CO_2_.

**Figure 67:**
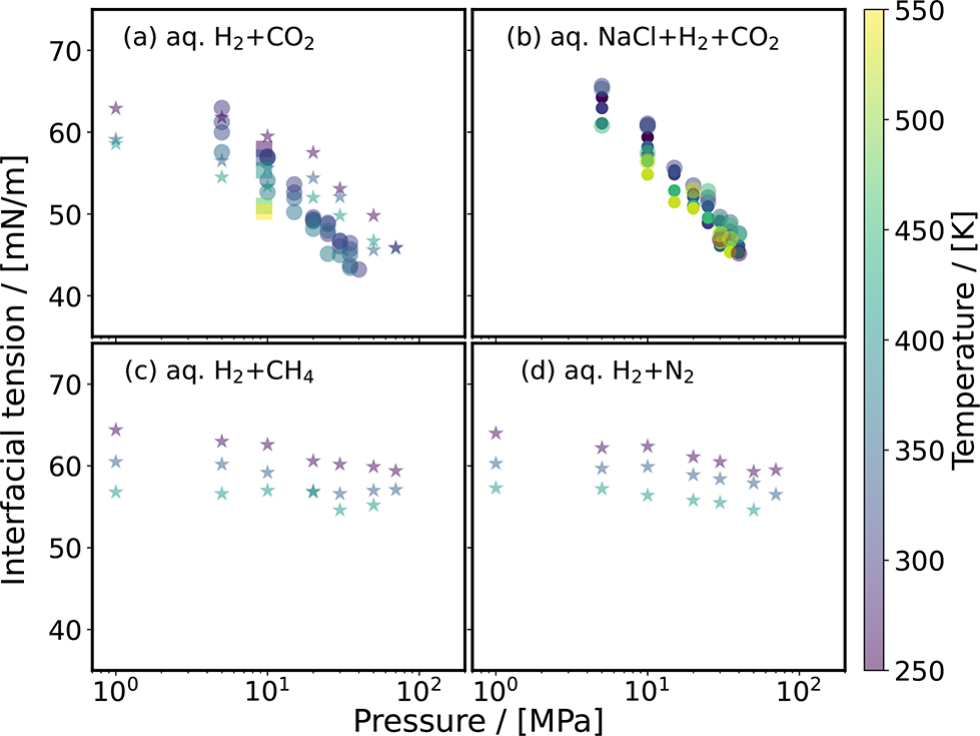
Interfacial tension of aqueous H_2_-gas and H_2_-gas-NaCl brine systems as a function of pressure computed with MD simulations. Gases considered in the H_2_-gas-H_2_O system are (a) CO_2_, (c) CH_4_, and (d) N_2_. (b) For the H_2_-gas-NaCl brine (3 mol kg^–1^), we consider only CO_2_ for comparison. The colors of the markers represent the temperature at which the interfacial tension is reported. The data are taken from Xie et al.^1019^ (circle), Doan et al.^1021^ (star), and Chen et al.^604^ (square). For comparison, a hydrogen mole fraction of 0.7 is chosen from the three different studies.

### 4.10 Contact Angles of Hydrogen–Brine–Solid Systems

Contact angle (CA) is a key metric of wettability, which governs how H_2_ gas, brine, and rock interact at nanoscale interfaces in UHS. Wettability controls capillary trapping, migration, and flow efficiency, yet experimental CA measurements under reservoir conditions are often hindered by surface roughness, contamination, and the challenges posed by extreme pressures and temperatures. MD simulations help overcome these limitations by resolving atomistic-scale interfacial structures, capturing gas adsorption, water layering, and ion-specific effects, while systematically varying pressure, temperature, salinity, gas composition, and mineral surface chemistry. When validated with experimental data, accurate force fields enable MD-derived CAs to inform larger-scale reservoir models, bridging molecular-level insights with field-scale performance. In what follows, we review studies that use MD simulations to investigate the CAs of water/brine droplets on different mineral substrates. Among these substrates, quartz surfaces are particularly important due to their prevalence in sandstone formations and their highly variable surface chemistry. To interpret the wettability trends observed in MD studies, it is useful to first outline the different types of quartz surfaces based on their degree of hydroxylation, as defined by Zhuravlev:^1047^

- Q4 Quartz Surface: Dehydroxylated (0 nm^–2^ OH), weakly hydrophobic.
- Q3 Quartz Surface: Partially hydroxylated (≈ 4.7 nm^–2^ OH), strongly hydrophilic.
- Q2 Quartz Surface: Fully hydroxylated (≈ 9.4 nm^–2^ OH), hydrophilic but with some OH-OH hydrogen bonding that slightly reduces water affinity.

Understanding these surface chemistries provides essential context for interpreting how CA, and, by extension, wettability, responds to changes in thermodynamic conditions and compositions relevant to UHS. The classification also helps prevent confusion when these surface types appear throughout the following sections.

Abdel-Azeim et al.^603^ investigated the CA of water droplets in a H_2_ gas atmosphere on caprock minerals mica, anhydrite, gypsum, and halite, examining the effects of pressure (5 to 20 MPa), temperature (298 to 323 K), and salinity (deionized water, seawater with 60000 ppm total dissolved solids, and formation water with 213000 ppm TDS). The system focuses on pure H_2_ gas without mixtures such as CH_4_ or CO_2_, and brines containing ions like Na^+^, Ca^2+^, Mg^2+^, Cl^–^, and SO_4_^2–^ are used to represent different salinities. The CAs obtained for the water droplet on these mineral surfaces in a H_2_ gas environment remain at or near zero degrees across all tested conditions, indicating strongly water-wet surfaces regardless of changes in pressure, temperature, or salinity. For example, in both MD simulations and sessile-drop experiments, the CA for water droplets on mica under H_2_ pressure up to 20 MPa at 300 K was consistently zero, and similar behavior was observed on anhydrite, gypsum, and halite surfaces. The physical mechanism underlying these CA trends is attributed to the significantly stronger interactions between water molecules and the mineral surfaces compared to the interactions between H_2_ molecules and the mineral surfaces. Water forms strong adsorption and hydrogen bonds especially on calcium sulfate minerals (gypsum and anhydrite) due to divalent cations like Ca^2+^. In contrast, H_2_ is nonpolar and exhibits weak hydrophobic interactions with the mineral surfaces, making it unable to displace water or alter wettability even at elevated pressures. Temperature and salinity influence water diffusion and spreading kinetics but do not affect the ultimate water-wet state. Among the studied factors, the intrinsic molecular interactions between water and mineral surfaces most strongly influence the CA, overriding effects from pressure, temperature, or salinity variations. CAs computed with MD simulations were validated by sessile-drop experiments on plasma-cleaned mineral surfaces, showing excellent agreement with near-zero CAs, whereas higher CAs reported in the literature were attributed to organic surface contamination on uncleaned samples. No general empirical fit function for CA as a function of pressure, temperature, and salt composition was proposed in this study.

Al-Yaseri et al.^607^ studied the CAs of water droplets on quartz and calcite mineral surfaces in a pure H_2_ gas environment over a pressure range of 3.44 to 206.8 MPa, temperatures from 298 K to 323 K, and brine salinities from deionized water up to 213000 ppm total dissolved solids, with no additional gas mixtures such as CH_4_ or CO_2_ considered. The CAs measured for the water droplet on these mineral surfaces in a H_2_ gas environment remain at or near zero degrees across all tested pressures, temperatures, and salinities, indicating a completely water-wet system; for example, CAs on quartz and calcite were zero even at the highest pressure of 206.8 MPa and formation water salinity. The MD simulations reveal that the dominant physical mechanism behind the zero CA is the significantly stronger molecular interactions between water and the mineral surfaces compared to the weaker H_2_-water and H_2_-mineral interactions, with water diffusion coefficients on quartz and calcite surfaces decreasing with increased salinity and stronger water-calcite interactions resulting in slower spreading times. Surface cleanliness was found to be the most critical factor influencing the CA measurements, as contamination can lead to artificially higher CAs, particularly on calcite surfaces. The CAs obtained experimentally using a carefully controlled cleaning protocol with air plasma treatment were in excellent agreement with MD simulations, both confirming the zero CA findings, resolving discrepancies in the literature reporting higher and variable CAs. No general empirical fit function for CA dependency on pressure, temperature, or salinity was proposed in the study.

Al-Yaseri et al.^606^ studied the wettability properties as quantified by CAs of H_2_ gas bubbles on quartz surfaces in the presence of brine containing 0.5M Na^+^ ions at room temperature and ambient pressure. The study focused on the quartz-brine-H_2_ interface, including the effect of organic acids such as Humic acid on wettability alteration. MD simulations were performed to measure the CAs of pure H_2_ bubbles on silica surfaces in deionized water and in 0.5M NaCl brine, yielding CAs of approximately 120° and 122°, respectively. These values indicate poor H_2_ wettability, consistent with a strongly water-wet quartz surface. In the simulations, the quartz surface was “aged” by introducing a model hydrophobic layer representing Humic acid decomposition products, such as naphthenic acids and hexane, adsorbed onto the silica surface. This ”aging” in MD refers to the surface modification step within the simulation framework rather than a physical experimental aging process. After this surface functionalization in the simulations, the CA of the H_2_ bubble on the hydrophobized silica surface decreased significantly to about 44°, indicating a shift toward intermediate or H_2_-wet conditions. This change is attributed to the hydrophobization of the silica surface by Humic acid, which enhances van der Waals interactions between H_2_ molecules and the modified mineral surface, thereby promoting H_2_ spreading. Experimentally, natural sandstone cores from the Lesueur Sandstone formation were aged by exposure to an aqueous Humic acid solution (10^–2^M) for 42 days at 5°C and room pressure. This laboratory aging caused a wettability shift consistent with the MD results: the CA transitioned from strongly water-wet toward an intermediate wetting state. Although exact experimental CAs were not directly measured, the wettability alteration was inferred from changes in H_2_ saturation and residual trapping, which are consistent with a CA reduction similar in magnitude to the 44° obtained in simulations. This study shows that the MD simulations capture the essential physics of wettability alteration by Humic acid.

MD simulations were conducted by Ali et al.^1048^ to investigate the effects of varying gas compositions, including H_2_, CO_2_, and CH_4_, on the wettability of kaolinite clay surfaces by brine droplets containing 10 wt% NaCl at 323 K and pressures between 5 and 40 MPa. The study focused on two distinct basal planes of kaolinite: the hydrophobic siloxane surface and the hydrophilic gibbsite surface, examining how CAs and IFTs respond to changes in pressure, temperature, and H_2_ content. On the hydrophobic siloxane surface, CAs of brine droplets in pure H_2_ environments ranged from approximately 91° to 106° over the pressure range studied, indicating an ‘intermediate wet’ behavior with relatively minor pressure dependence. When CO_2_ was introduced as a cushion gas, the CAs increased significantly, ranging from about 118° at 5 MPa to nearly 160° at 40 MPa, corresponding to a transition from weakly gas-wet to strongly gas-wet surfaces. Mixtures of H_2_ and CO_2_ showed a marked reduction in the wettability of brine compared to pure H_2_; for example, at 20 MPa, a system with 90 wt% CO_2_ exhibited a CA of approximately 106°, lower than pure CO_2_ but substantially higher than pure H_2_. The effect of CH_4_ as a cushion gas followed similar trends but was less pronounced than CO_2_, with CAs increasing by up to 15° relative to pure H_2_. Increasing pressure consistently increased CAs across all gas compositions, attributed to enhanced intermolecular interactions between the denser gas phases and the siloxane surface. Atomic density profiles revealed that CO_2_ molecules adsorb closer to the siloxane surface (at approximately 2.7 Å) compared to CH_4_ (ca. 3.1 Å) or H_2_, effectively displacing some water molecules from the surface and reducing brine wettability. Water molecules form two distinct hydration layers near the surface, with gas molecules preferentially located within the first hydration layer; higher gas densities at elevated pressures amplify gas-surface affinity, further increasing CAs. On the hydrophilic gibbsite surface, H_2_ formed cylindrical bubbles detached from the surface, indicative of strong water wettability and complete spreading of water. Increasing pressure from 20 to 80 MPa resulted in denser H_2_ bubbles with reduced radius (from approximately 50.5 Å to 33.0 Å), reflecting increased water CAs and decreased H_2_ affinity with the surface. Temperature increases from 303 K to 343 K led to reduced affinity between H_2_ and the gibbsite surface, suggesting an increase in wettability at higher temperatures due to a decrease in H_2_ density. The presence of CO_2_ in H_2_ bubbles on the gibbsite surface reduced H_2_ density near the surface, further influencing wettability trends. Regarding temperature effects on the gibbsite surface, the study indicated a less pronounced influence compared to pressure. However, higher temperatures tend to reduce gas density, and thus, gas affinity, which would increase wettability, although these effects were secondary to pressure-driven trends. These observations indicate that pressure and temperature influence wettability on the hydrophilic surface in manners similar to those on the hydrophobic siloxane surface but with overall stronger water affinity leading to complete wetting. Overall, the simulations demonstrate that the wettability of kaolinite surfaces by brine is significantly modulated by gas composition and thermodynamic conditions, with CO_2_ causing the most substantial increase in gas-wetting behavior on the hydrophobic siloxane surface, followed by CH_4_, while pure H_2_ maintains intermediate wettability. Pressure predominantly governs these effects by altering gas density and gas-surface interactions, whereas temperature has a subtler role, particularly on the hydrophilic gibbsite surface. The simulated CA trends align well with experimental observations reporting increased CAs with pressure and stronger gas wetting for CO_2_ compared to H_2_, although some discrepancies exist due to differences in surface basal planes studied.

The study by Alshammari et al.^1049^ focused on IFT and wettability of water–H_2_ systems-pure and with cushion gases (CH_4_, CO_2_)-in brines containing Na^+^, Mg^2+^, Ca^2+^, and Cl^–^. Simulations were performed at 298-323 K, 5–20 MPa, and salinities from deionized to formation water. CAs of water on calcite and silica were evaluated up to 20 MPa at ≈ 300 K. pH was represented by tuning the ionization of surface silanol groups on silica slabs: 0% ionization (all silanols protonated) reproduce acidic conditions (pH ≈ 3), whereas 10% ionization (partial deprotonation) correspond to near-neutral pH ≈ 6. This shift from a neutral to a negatively charged surface alters rock-fluid interactions, moving the quartz CA from an intermediate value toward a strongly water-wet state as water molecules increasingly displaced adsorbed CO_2_. In H_2_ gas, calcite stayed strongly water-wet (CA ≈ 0°); quartz showed CA ≈ 56 - 75°, shifting toward intermediate wetting with changing gas mixtures and surface charge. The highest CA was in H_2_/CO_2_ at low pH, as CO_2_ strongly adsorbs on protonated silica, thickening the interfacial gas layer and slowing water spread. In contrast, CH_4_ and pure H_2_ interact weakly, keeping CA lower. Barbosa et al.^1050^ reported wettability, specifically CAs, of water and brine droplets containing NaCl (up to 20 wt%) on siloxane, gibbsite, and defective siloxane surfaces of kaolinite in the presence of gases including H_2_, CH_4_, and CO_2_ across conditions relevant to underground H_2_ storage, with pressure at 10 MPa, temperature at 323 K, and varying defect densities on the substrate. The CAs of brine droplets on the siloxane surface without gas are approximately 93°, indicating hydrophobic behavior, whereas on gibbsite surfaces CAs are about 13°, showing hydrophilicity; the presence of CO_2_ significantly increases CAs (e.g., up to 131° on siloxane), while H_2_ (95°) and CH_4_ (100°) cause smaller increases. Increasing defect density on siloxane surfaces by removing up to 30% of silicon sites reduces CAs for brine droplets, e.g., from 84° to 68° in H_2_ environments and from 111° to 83° in CO_2_ environments, indicating enhanced wettability with more defects; CAs for pure water are generally lower than for brine and show greater variability. The physical mechanism behind these trends is attributed to stronger adsorption of CO_2_ on heterogeneous surfaces displacing water and altering surface hydrophilicity, the formation of silanol nests at defect sites may alter hydrophilicity, and salt ions (Na^+^, Cl^–^) interacting with the surface to reduce CAs by modifying water-surface interactions. Surface defects, particularly their density and spatial coordination, most strongly influence CAs, with machine learning models showing improved prediction when defect arrangement is considered. The simulation data of CAs for brine on siloxane and gibbsite surfaces agree well with available experimental data, supporting the validity of the MD approach.

Fatah et al.^1051^ performed MD simulations to compute the wettability and flow of H_2_, CH_4_, CO_2_, and N_2_ against 57-670 ppm brine (Na^+^, Mg^2+^, Ca^2+^, Cl^–^, SO_4_^2–^) on calcite at 25 °C and 1000 psi. In all cases the water droplet spread completely (CA ≈0°), confirming calcite is strongly water-wet; seawater-calcite interaction energies dwarfed gas-rock and water-gas terms. Although CO_2_ adsorbed most strongly and its brine IFT was far lower than for H_2_, CH_4_, or N_2_, these differences did not alter the CA. Hence gas type and brine composition exert negligible influence on calcite wettability, which is governed by the mineral’s hydrophilicity, an insight vital for underground H_2_-storage design.

Ghafari et al.^1052^ used MD simulations to examine water CAs on silica surfaces in brine/gas environments under subsurface conditions (10 - 30 MPa, 333 - 413 K), with H_2_ mole fractions varying from 0 to 1 and silica surface charges from 0 to -0.12 C m^–2^. Binary gas mixtures of H_2_ with CO_2_, CH_4_, or N_2_ were considered at *x*_H_2__=[0.0, 0.3, 0.7, 1.0], showing that increasing the mole fraction of cushion gases, especially CO_2_, raises the CA, an effect amplified at higher pressures and lower temperatures. Five distinct silica surface models (Q2, Q3, and Q3/Q4 types) with silanol densities between 2.35 and 9.58 nm^–2^ were tested, with *α*-quartz (101) at 5.9 nm^–2^ serving as the reference substrate. Surface charges were introduced by deprotonating a fraction of surface silanol (Si-OH) groups: 0% ionization (all silanols protonated) represents acidic conditions, whereas 10% ionization (partial deprotonation) mimics near-neutral pH. On neutral silica in pure H_2_, the CA remains low and nearly independent of pressure and temperature. For neutral surfaces, the four cushion gases increase the contact angle in the following order: CO_2_ > CH_4_ > N_2_ > H_2_. This trend arises from competitive gas adsorption, CO_2_ has the strongest affinity for silica–forming a thicker adsorbed layer that impedes water spreading under high–pressure, low-temperature conditions while CH_4_ and N_2_ exhibit weaker adsorption, and H_2_ the weakest. The surface charge exerts the most pronounced influence: As ionization increases to -0.12 C m^–2^, the silica becomes strongly water-wet (CA → 0°) regardless of the composition, pressure, or temperature of the gas, because deprotonation enhances the electrostatic interactions between rocks and water that displace the adsorbed gas. Simulations of *α*-quartz (101) based on the INTERFACE force field closely reproduce experimental CA data, correcting the overestimation of hydrophilicity reported when using CLAYFF or DDEC force fields. These results emphasize that accurate force field selection and realistic surface characterization are essential for predicting wettability in brine–gas–rock systems relevant to UHS.

Atomistic MD simulations were conducted by Phan et al.^1053^ to investigate the wetting properties of brine droplets containing 20 wt% Na^+^ and 1 wt% K^+^ on talc and the hydroxylated basal face of kaolinite (kaoOH) substrates in gas mixtures comprising H_2_ and cushion gases (CH_4_ or CO_2_) at geological conditions of 15 MPa and 333 K, with varying gas compositions including pure H_2_, 75 mol% H_2_ - 25 mol% cushion gas, 60 mol% H_2_ - 40 mol% cushion gas, and pure cushion gas. The CAs of brine droplets on talc surfaces were found to be strongly hydrophobic with values greater than 90°, increasing from approximately 102.6° in pure H_2_ to about 143.4° with pure CO_2_, and to ca. 112.4° with pure CH_4_, whereas on the kaoOH surface the CAs remained consistently low, between 11.4° and 15.8°, regardless of gas composition. The authors attribute these trends to the differences in clay-brine interactions and surface chemistry, where talc’s hydrophobic siloxane basal face leads to more favorable cavity formation near the surface (lower free energy) and thus higher CAs, while kaoOH’s hydrophilic hydroxylated surface exhibits stronger water adsorption and higher free energy of cavity formation, resulting in lower CAs. Among the factors studied, the nature of the clay surface chemistry most strongly influences the CA. The simulation results for CAs on kaoOH closely agree with experimental data reporting values near 15° to 20°, while differences with some experiments on kaolinite siloxane faces are noted, likely due to variation in the specific basal planes studied.

Yang et al.^602^ studied interfacial properties of the H_2_ + H_2_O and H_2_ + H_2_O + solid systems with silica and kerogen substrates over a broad range of temperatures (298 to 523K) and pressures (1 to 160 MPa). The study focused on pure H_2_ gas mixtures without additional gases such as CH_4_ or CO_2_ and did not address salt concentration effects in brine; CAs of H_2_O droplets on silica and kerogen surfaces in a H_2_ gas environment were analyzed. The CAs of the H_2_O droplet on silica surfaces ranged from approximately 21.8° to 49.0° and generally increased with pressure while decreasing with temperature, with less pronounced temperature and pressure effects at low temperatures (298 and 373 K). For kerogen surfaces, the CAs ranged from 0° (fully water-wet) to about 61.9°, showing a decrease with increasing temperature and an increase with pressure, with CAs increasing from 0° to 31.4° as pressure rose from 5 to 160 MPa at 523 K. These CA trends were explained through variations in the IFT between H_2_O-rich and H_2_-rich phases and adhesion tensions representing fluid-solid interactions; the adhesion tension is defined as the difference between the IFT of the solid with the H_2_-rich phase and that with the water-rich phase, reflecting the fluid-solid interaction contribution to wettability. Adhesion tensions decreased significantly with temperature and showed moderate pressure dependence on silica but decreased with pressure on kerogen, especially at lower temperatures. The strongest influence on CAs was identified as the balance between fluid-fluid IFT and fluid-solid adhesion tension, which both vary with temperature and pressure. CAs obtained from MD simulations were consistent with previous simulation studies and showed qualitative agreement with experimental trends, although absolute values for silica were somewhat higher likely due to differences in surface silanol group density; no explicit quantitative fit to experimental CAs was reported.

The MD study by Yao et al.^1054^ examined bulk and interfacial properties (IFT and water–silica CAs) in H_2_/brine systems, i.e., NaCl, KCl, CaCl_2_, over 323 - 423 K, 14 - 150 MPa, and up to 5.4 mol/kg salt, and provided comparisons with CO_2_/brine/silica systems. CAs on silica in H_2_/brine span roughly 42.5° to 72.2°, as salt increases from 0 to 5.4 mol/kg in the order KCl < NaCl ≈ CaCl_2_. Pressure and temperature effects on the angle are otherwise minor. By measuring cos *θ* (CA) and *γ*_WG_(brine-gas surface tension), the authors use Neumann’s equation,^1055^ with an empirical constant *β*, to evaluate *γ*_SG_ (solid-gas surface tension) and *γ*_SW_ (solid-brine surface tension), though *γ*_SG_ and *γ*_SW_ are challenging to directly obtain. They report *γ*_SG_ remains effectively constant with salinity (whether imposed or derived is unclear from their article), and as a result *γ*_SW_ decreases as salt concentration rises. Consequently, the adhesion tension (*γ*_SG_ – *γ*_SW_) falls with salinity, explaining the larger CAs. Beyond noting these interdependencies, the study does not draw further conclusions from the trend in *γ*_SW_ alone. It seems that ion exclusion (negative surface excess) raises *γ*_WG_ and shifts the force balance at the contact line and drives the wettability behavior. Salt concentration thus most strongly controls the CA by altering adhesion tension. The computed angles and IFTs compared reasonably well with experimental data for H_2_/NaCl brine on silica, though some discrepancies remain, likely due to force–field limitations.

Yu et al.^1056^ performed MD simulations to compute the CAs of water droplets on organic-rich shale surfaces, represented by graphene, in pure H_2_ environments and H_2_-CH_4_ and H_2_-CO_2_ gas mixtures, over pressure ranges from 5 to 40 MPa and temperature ranges from 292 to 343 K, with gas mole fractions varying from 25% to 100% H_2_; salt concentration and specific brine ion effects are not explicitly modeled but are noted as future considerations. The CA of water on these substrates generally decreases with increasing temperature by up to about 5.8° at an intermediate pressure of 5 MPa and increases with increasing H_2_ pressure by roughly 20° from 5 to 40 MPa, indicating a shift toward less water-wet conditions; in CO_2_-H_2_ mixtures, CAs remain high at 180° (fully gas-wet) when the H_2_ fraction is below 25% and sharply decrease to strongly water-wet values around 22° as H_2_ fraction approaches 100%, while in CH_4_-H_2_ mixtures, the system transitions from moderately gas-wet to strongly water-wet as H_2_ fraction increases, with a neutrally wet state defined by CAs near 90° at about 75% CH_4_. These trends are explained by molecular interactions where increased temperature (292–343 K) reduces H_2_ density and adsorption due to higher kinetic energy, weakening H_2_-surface interactions and lowering CAs, whereas increased pressure (5–40 MPa) enhances H_2_ density and adsorption, increasing CA; competitive adsorption in gas mixtures causes preferential adsorption of CO_2_ and CH_4_ over H_2_, influencing wettability and CA behavior. CA results from molecular simulations agree qualitatively with experimental data on H_2_-organic shale systems but show variations attributed to differences in rock composition, surface roughness, and experimental uncertainties.

The study by Zhang et al.^1057^ focused on CAs of H_2_ with brines of varying salinity (up to about 1 mol/L NaCl) on mineral substrates quartz, calcite, halite, and montmorillonite, across pressure ranges of 5 to 25 MPa and temperatures from 300 to 400 K. The CAs of water on these mineral surfaces in a H_2_ gas environment were examined using MD simulations under these reservoir-like conditions. CAs generally increase with pressure; for example, CAs for quartz increased from approximately 25° at 5 MPa to about 40° at 25 MPa. Temperature effects on CAs are less pronounced. For all substrates, CA slightly decreases (by ca. 5°) when temperature increases from 300 to 400 K. Salinity increases tend to raise CAs by ca. 5°, except for calcite where the effect is negligible, less than 1° change up to 1 mol/L NaCl. The authors observe that the variation in the bulk density of H_2_ with pressure correlates with the cosine of the contact angle: as the bulk density of H_2_ increases with pressure (from ca. 5 kg/m^3^ to 20 kg/m^3^ between 5 and 25 MPa), the cosine of the contact angle decreases. Electrostatic interactions and the electrical double layer on charged minerals like montmorillonite enhance water wettability strongly, contributing up to 50% of the total interaction energy. Among the investigated factors, pressure, via its effect on H_2_ density, exerts the strongest influence on CA. The simulated CAs were compared to various experimental measurements, showing reasonable agreement especially for clean mineral surfaces, with discrepancies attributed to organic surface contamination and surface heterogeneity in experiments. The authors propose a theoretical model based on a sharp-kink approximation that relates CA to H_2_ density, IFT, and water-mineral interaction energy, which can predict trends of CA as functions of pressure, temperature, and salinity.

Using MD simulations Zheng et al.^609^ studied the wettability of pure water droplets on quartz substrates in the presence of H_2_ gas under various geo-storage conditions, covering pressures from 1 to 30 MPa and temperature fixed at 338 K. The study focuses on pure H_2_ environments and considers hydroxylation states of quartz surfaces (Q2, Q3, and Q4 quartz). The CA of water on fully hydroxylated quartz surfaces in a H_2_ environment fluctuates between approximately 30.7° and 37.1° across the pressure range studied, with CAs increasing from 4.5° on partially hydroxylated quartz (Q3) to 34° on fully hydroxylated quartz (Q2), and reaching 94.7° on dehydroxylated quartz (Q4), indicating a transition from hydrophilic to weakly hydrophobic behavior depending on surface chemistry. The authors attribute these CA trends primarily to the flexibility of the quartz substrate and the arrangement and area density of surface hydroxyl groups, where hydrogen bonding probability between water and hydroxyl groups strongly dictates hydrophilicity; the CA increase with hydroxyl density is nonmonotonic due to intramolecular hydrogen bonding among surface hydroxyls reducing available bonding sites for water. The simulated CAs fall within the range of experimental measurements reported in the literature, confirming the validity of the MD models, although discrepancies in pressure dependence of CA observed experimentally are suggested to arise from surface roughness and experimental conditions. No general empirical fit function for CA as a function of pressure, temperature, or salt composition is proposed in this work.

The study by Zheng et al.^608^ focused on the wettability of quartz surfaces with varying surface chemistries and morphologies under subsurface H_2_ geo-storage conditions, examining the effects of pressure up to 1000 MPa, temperature at 338 K, NaCl concentrations from 0 to 1.0 mol%, and gas mixtures including pure H_2_ and H_2_-CH_4_ mixtures with CH_4_ mole fractions up to 50 mol%. CAs of water droplets on fully hydroxylated, half-methylated, fully methylated, pristine, and rough quartz surfaces in H_2_ and H_2_-CH_4_ environments are quantified to understand wetting behavior. For fully hydroxylated (non-methylated) quartz surfaces, the water CA remains consistently near 34° across NaCl concentrations, showing negligible influence from salinity. Similarly, the addition of CH_4_ as cushion gas only slightly increases the CA to about 36.1°, indicating minimal wettability change. With pressure variation up to 30 MPa, the CA on fully hydroxylated quartz fluctuates modestly between approximately 30.7° and 37.1°, driven primarily by the pinning effect due to surface heterogeneity at low pressures, and transitions to H_2_-quartz interaction dominance at higher pressures. The fully hydroxylated rough quartz surfaces also exhibit a distinct jump in CA between 38 and 70 MPa, corresponding to a transition in the water film state on the rough surface. Moving to half-methylated quartz surfaces, CAs decrease from about 45.9° to 31.2° as NaCl concentration rises to 0.5 mol%. In H_2_-CH_4_ mixtures, the half-methylated quartz surfaces show a notable CA decrease from 45.9° to 32° with CH_4_ mole fraction increasing to 20 mol%, followed by a slight increase at higher CH_4_ content. The variation of CA with pressure on half-methylated quartz is non-monotonic, increasing from 41.2° to 49.3° between 1 and 20 MPa, then slightly decreasing at 30 MPa; this behavior is attributed to a competition between the pinning effect caused by surface heterogeneity (including organic ligand distribution) at low pressures and H_2_-quartz interactions at higher pressures. Finally, for fully methylated quartz surfaces, the CA of water increases significantly with methylation degree, rising from about 32° at 12.5% methylation to 96.4° at 87.5% methylation. Interestingly, a slight decrease in CA from 34° to about 32° is observed when methylation increases from 0 to 12.5%, explained by the dual role of methyl groups: while their hydrophobic nature reduces surface hydrophilicity at high methylation, at low methylation they suppress hydrogen bonding among surface hydroxyl groups, thereby enhancing hydrophilicity. The study highlights that methylation degree is the strongest factor influencing the CA, whereas pressure and salinity effects are strongly dependent on the quartz surface chemistry and morphology. Simulated CAs for water on fully hydroxylated quartz in H_2_ at 10 MPa and 338 K are ca. 34°, aligning well with experimental values ranging from 30° to 40°. This research reconciles prior experimental discrepancies by considering detailed variations in surface morphology and chemistry.

Chen et al.^604^ computed interfacial properties including CA, IFT, surface excess, and bubble morphology of CO_2_/H_2_ mixtures in contact with brine containing Na^+^ and Cl^–^ ions and hydrophilic silica surfaces, over pressure of 10 MPa and temperature range 300-400 K, with gas mixtures varying in H_2_ mole fraction from pure H_2_ to CO_2_-rich systems and brine salt concentration of ca. 2.22 molal. The CAs of CO_2_ and H_2_ bubbles on hydrophilic silica in H_2_O and brine environments are greater than 100°, with CO_2_ showing smaller CAs (e.g., 109.8° to 134.3° for CO_2_ and up to 156.8° for H_2_) indicating stronger wetting affinity to silica compared to H_2_; salts reduce CA, particularly at low temperatures, and CA for CO_2_ increases with temperature while that of H_2_ is less sensitive. The authors attribute CA trends to molecular interactions where CO_2_ has stronger electrostatic and hydrogen bonding interactions with H_2_O and silica surfaces due to its quadrupole moment, while H_2_ interacts weakly via van der Waals forces; NaCl ions modify surface polarity by forming an electrical double layer reducing CA, and temperature affects dissolution and diffusivity impacting wetting behavior. CAs predicted by MD simulations are consistent with available experimental data, showing similar trends in temperature and pressure dependence, although direct quantitative comparison is limited by experimental uncertainties and system complexities.

MD simulations were performed by Chen and Xia^605^ to investigate the transport and interfacial properties including density, viscosity, diffusion coefficient, wettability, IFT, and surface adsorption of H_2_, CO_2_, and CH_4_ gases interacting with H_2_O and silica substrates under typical subsurface reservoir conditions at a temperature of 320 K and pressures up to 60 MPa. The study focused on CAs of water droplets on two types of silica substrates, hydrophilic Q2 and hydrophobic Q4, in environments containing pure H_2_, CO_2_, or CH_4_ gases. The CA of water on both Q2 and Q4 silica substrates generally increased with gas pressure for CO_2_ and CH_4_, reaching plateaus between 10 and 30 MPa, with CO_2_ showing the highest CAs, followed by CH_4_, and then H_2_, which had minimal effect even up to 60 MPa. For example, the CA on hydrophobic Q4 silica was about 106° without gas and increased to approximately 142° in a CO_2_ environment at 10 MPa, whereas H_2_ caused negligible changes in CA under all pressures. The physical mechanism explaining these trends arises from the differences in intermolecular interactions: CO_2_ exhibits strong electrostatic interactions with silica due to its quadrupole moment, leading to higher wettability and increased CAs, while H_2_ interacts weakly via van der Waals forces and has low adsorption capability on silica surfaces, resulting in little alteration of the water CA. The CA correlates inversely with the gas–water IFT, which remains nearly constant for H_2_ but decreases significantly with pressure for CO_2_. Gas pressure, particularly of CO_2_, is the most influential factor affecting CA, while substrate hydrophilicity or hydrophobicity also plays a significant role in baseline CA values. MD results for CAs show good qualitative agreement with available experimental data for CO_2_ and CH_4_ systems, although some discrepancies exist regarding the pressure dependence of CAs in H_2_ environments.

Huang et al.^1058^ performed MD simulations to study CAs of deionized water and brine droplets containing Na^+^ and Cl^–^ ions on fully hydroxylated *α*-quartz (Q2 surface) substrates under varying gas environments including pure H_2_, CH_4_, and CO_2_ gases. The simulations are conducted at a temperature of 333 K and a pressure of 20 MPa, with brine salinities of ca. 1.5 M and 3.5 M, mimicking typical geological conditions relevant for underground gas storage and recovery. The CA of water droplets on quartz in the presence of different gases follows the order *θ*_H_2__ < *θ*_CH_4__<*θ*_CO_2__, with all values below 50°, indicating a strongly hydrophilic surface; the presence of salt ions generally increases the CA regardless of the gas type, thus reducing wettability. This trend is explained by the authors through interaction energy analyses showing that gas-quartz interactions decay faster than gas - water interactions, with the gas-quartz interaction energies ordered as CO_2_ > CH_4_ > H_2_, while water-quartz interactions remain relatively constant and dominated by electrostatic forces (≈ 95%), minimally affected by salt ions; the increased CA with salinity is attributed primarily to the augmented gas–water IFT caused by salt addition. The gas species type most strongly influences the CA, with CO_2_ producing the largest values due to its stronger van der Waals and electrostatic interactions with quartz, and salt concentration also plays a significant role by increasing IFT and thus CA. The computed CAs are consistent with experimental observations showing quartz surfaces are more hydrophilic in H_2_ environments and become less hydrophilic with increasing salinity.

#### 4.10.1 Effect of Substrate Material, Pressure, Temperature, and Salinity on the Computation of Contact Angles

Based on the studies reviewed above, it is clear that the CA of a water droplet varies with substrate, salt concentration, pressure, temperature, and surrounding gas composition. In Fig. 68(a), CAs collected from multiple studies^602,604,605,607,609,1052,1054,1056–1058^ are shown. These data are shown irrespective of the pressure and temperature conditions to isolate substrate effects. A clear trend emerges where all substrates except Q4 Quartz are hydrophilic (water-wet in UHS terminology), with CAs below 60 degrees. In contrast, Q4 Quartz shows CA values of ca. 100 degrees, indicating weak hydrophobic (gas-wet) behavior. This aligns with its dehydroxylated surface having negligible hydroxyl group density (0 nm^–2^),^1047^ which limits hydrogen bonding. Also evident in Fig. 68(a) is the consistency across studies, i.e., CAs for Q2 Quartz, Q3/Q4 Quartz, and Q4 Quartz agree within 20 degrees. The key message is that CAs can range from 0 to 120 degrees depending on the substrate, with substrate effects outweighing those of pressure and temperature. To further emphasize this, Fig. 68(b) compiles CA data for water or brine droplets, regardless of pressure, temperature, salt concentration, or surrounding gas. Here, CAs span 0 to 180 degrees. Non-wetting substrates such as the siloxane surface of Kaolinite, Q4 Quartz, Talc, and Humic acid stand out clearly. Graphene also shows a non-wetting trend in the study by Yu et al.,^1056^ with CAs above 90 degrees due to exposure to CO_2_, which reduces water adsorption. Similarly, Ali et al.^1048^ report brine droplets on Q4 Quartz with CAs ranging from 100 to 180 degrees depending on the CO_2_ content in the environment surrounding the bubble. Chen and Xia^605^ further show that water on Q4 Quartz in CO_2_ exhibits a CA increase of nearly 60 degrees with pressure. In summary, surface chemistry has the strongest influence on CA, followed by the effect of CO_2_ composition on substrates like Graphene^1056^ and Q4 Quartz.^1048^

**Figure 68:**
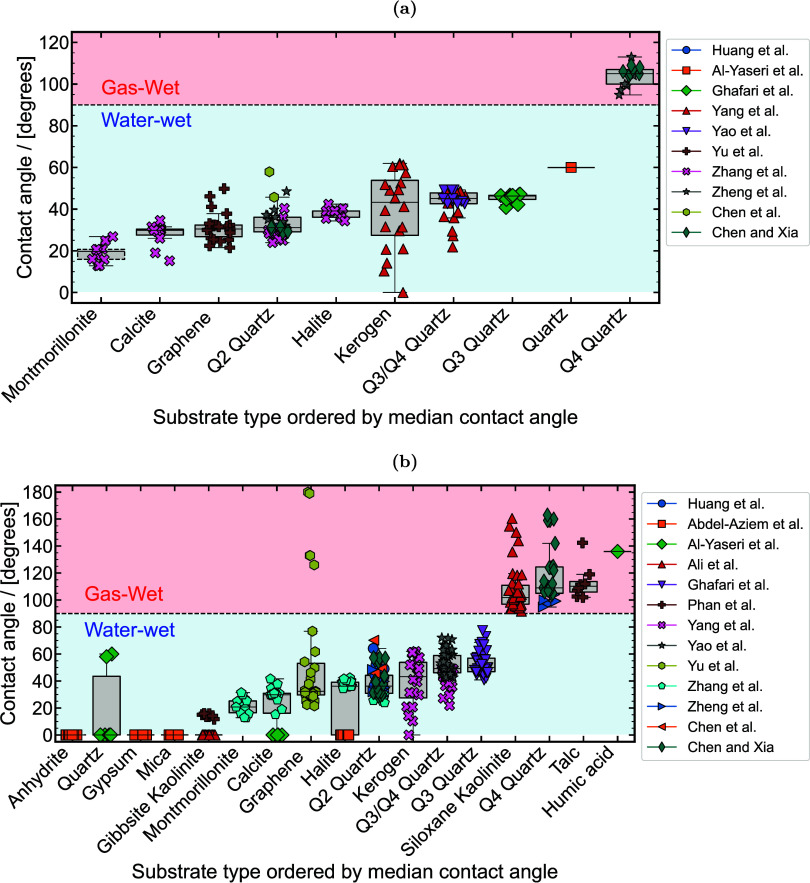
(a) Data for contact angles of pure water droplets immersed in pure H_2_ for different substrates collected from multiple studies. Angles below 90° indicate water-wet surfaces, while those above 90° indicate gas-wet surfaces. All reported contact angles are shown regardless of temperature and pressure. (b) Data for contact angles of water/brine droplets immersed in a gas environment (pure H_2_, H_2_-CO_2_, H_2_-CH_4_) collected from different sources. The complete list of studies and their temperature ranges are listed in Table 20.

In Fig. 69(a) and Fig. 69(b), the effect of pressure across different substrates is shown. Clearly, CA increases with pressure. Data from Yang et al.^602^ in Fig. 69(a) (small and large plus symbols) show CA rising from 0 to 30 degrees on Kerogen and from 20 to 40 degrees on Q3/Q4 Quartz as pressure increases from 1 to 160 MPa. A similar trend is reported by Zhang et al.,^1057^ where CA increases from 25° to 40° at *T* = 300 K. Notably, Zheng et al.^608^ computed CAs over a wide pressure range (1 - 1000 MPa) on Q4 Quartz and observed a maximum variation of only about 20 degrees. In contrast, Ghafari et al.^1052^ have shown that CAs are weakly dependent on pressure shown specifically for water droplets on Q3 Quartz (pink triangles in Fig. 69(a)). In Fig. 69(b), contact angles on Calcite, Montmorillonite, and Halite from Zhang et al.^1057^ show a modest increase with pressure. By contrast, Yu et al.^1056^ in Fig. 69(b), and Chen et al.^604^ in Fig. 69(a), report no clear pressure dependence. Overall, pressure tends to increase CA, although the effect is relatively weak. The underlying mechanism, as suggested by several authors, involves gas adsorption density increasing more strongly near the substrate than that of the liquid, due to gas compressibility. This enhances gas–substrate interactions, shifting the interfacial balance and increasing CA.

**Figure 69:**
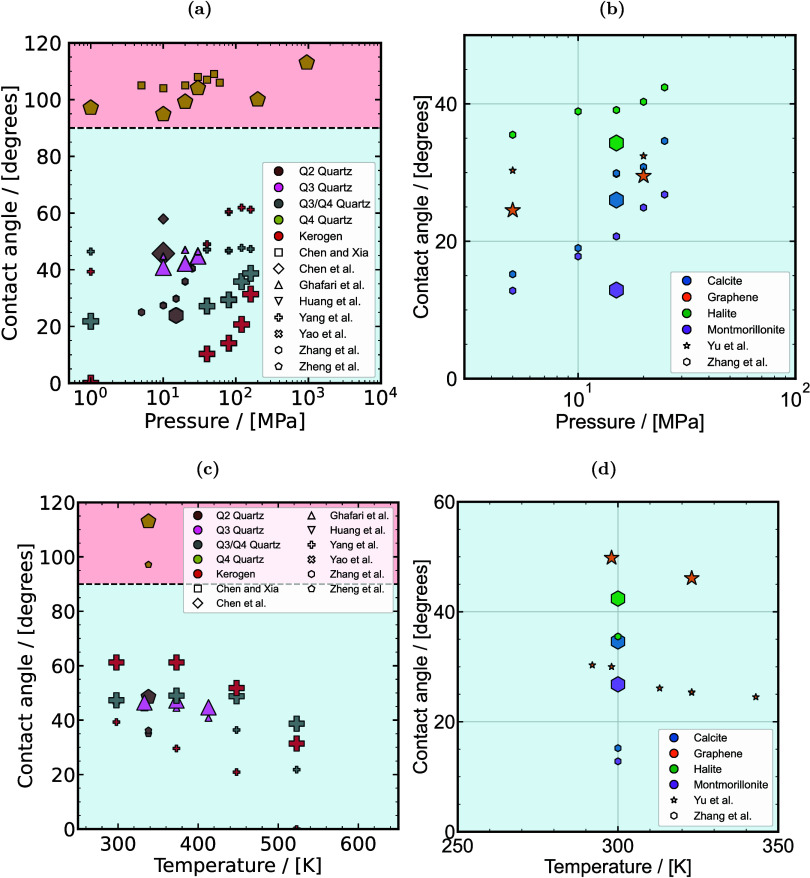
Computed contact angles of pure water droplets immersed in H_2_ as a funtion of pressure (a: low pressure data and b: high-pressures data) and temperature (c and d: all pressures reported) compiled from multiple studies. Angles below 90° denote water-wet surfaces, while those above 90° denote gas-wet surfaces. From each study, only the data with the lowest and highest pressures/temperatures are shown. The marker size represents the magnitude of pressure/temperature: In (a) and (b) large and small symbols represent high and low temperatures, respectively, and in (c) and (d) large and small symbols represent high and low pressures, respectively. A complete list of the studies and the respective temperature ranges are provided in Table 20.

The effect of temperature on the CAs of water droplets is shown in Fig. 69(c) and Fig. 69(d). One can observe that increasing temperature reduces CA, enhancing water-wetness. In the study by Yang et al.^602^, water droplets on Kerogen show a clear decrease in CA with temperature: at 160 MPa (large plus symbols), CA drops from 60° to 30°, and at 1 MPa (small plus symbols), from 40° to 0°. On Q3/Q4 Quartz, however, the reduction is less pronounced. At 1 MPa, CA decreases from approximately 45° to 20°, and at 160 MPa, from 45° to 40°, indicating substrate-dependent variation with temperature. Yang et al.^602^ attribute this trend to weakened H_2_ adsorption at higher temperatures, where entropic contributions dominate, allowing water to spread more readily by displacing gas molecules—a mechanism likely applicable to other gases as well. Similarly, CA variation on Q3 Quartz (pink triangles) in Ghafari et al.^1052^ is minimal. As shown in Fig. 69(d), Yu et al.^1056^ also reported a decrease of about 10° in CA for water on graphene as temperature increases from 260 to 340 K. As a concluding remark we can say that rising temperature weakens gas adsorption, facilitating water spreading and reducing contact angles.

The effect of salt concentration on the CAs of water droplets is shown in Fig. 70. It can be observed that increasing salt concentration raises CA by reducing water-wetness. In Fig. 70(a), the CAs of NaCl brine droplets from Yao et al.^1054^ (Q3/Q4 Quartz) and Zhang et al.^1057^ (Montmorillonite, Calcite, Halite, and Quartz, overlapping markers) show a clear increasing trend with salt concentration. Huang et al.^1058^ reported a similar rise on Q2 Quartz. Thus, despite variations in substrate type, CAs consistently increase with salt concentration. Notable exceptions include Al-Yaseri et al.^607^ and Chen et al.,^1046^ where CA remains largely unchanged with increasing salt. In Fig. 70(b), Yao et al.^1054^ show that KCl exhibits a similar trend on Q3/Q4 Quartz. Therefore, increasing the concentration of either NaCl or KCl appears to similarly increase CA. This rise is attributed to ion accumulation in the water bulk, which lowers the water-H_2_ IFT.^1054^ At the same time, the adhesion tension (difference between solid-brine and solid-gas interfacial energies) adjusts such that its ratio with the water-H_2_ IFT decreases, leading to a higher CA, consistent with Young’s equation.

**Figure 70:**
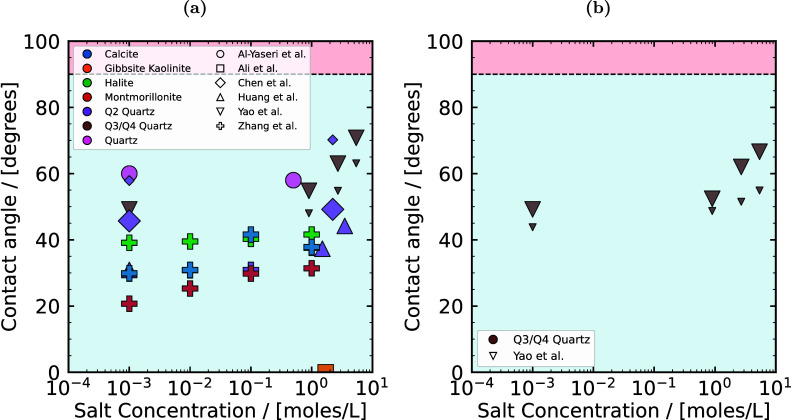
Data for contact angles of pure water droplets immersed in pure H_2_ as a function of salt concentration of (a) NaCl and (b) KCl, compiled from multiple studies. Angles below 90° indicate water - wet surfaces (blue shade), while those above 90° indicate gas - wet surfaces (red shade). All reported pure-water CAs from each study are included, regardless of pressure and temperature. The CA data for the largest pressure and largest temperature is represented by larger - sized symbol and the smaller symbol represents the CA at the lowest pressure and lowest temperature by the same study. All CA data at intermediate pressures and temperatures are omitted for visual clarity. The complete list of studies and their pressure ranges are listed in Table 20.

**Table 20:** Summary of the MD Simulation Studies Investigating the CAs in aqueous brine-H_2_ Gas Mixture-Solid Systems.^a^

	System details		Thermodynamic conditions		
Study	Species	Force field	*p* [MPa]	*T* [K]	*c*_salt_ [mol / L of solvent]
Abdel-Azeim et al.^603^	Flexible H_2_	IFF^313^	5 and 20	300 and 323	0
	H_2_O	SPC/E^346^			
	KAl_2_(Si_3_Al)O_10_(OH)_2_ (Mica)	ClayFF^422^			
	CaSO_4_.2H_2_O (Gypsum)	ClayFF^422^			
	CaSO_4_ (Anhydrite)	ClayFF^422^			
	NaCl (Halite)	ClayFF^422^			
	Brine : Na^+^, Ca^2+^, Mg^2+^	Aqvist^1044^			0.001, 0.007
	Brine : SO_4_^2–^, Cl^–^, HCO_3_^–^	Chandrashekhar^1059^			0.001, 0.007
Huang et al.^1058^	H_2_	Alavi^1060^	20	333	1.5 and 3.5
	CO_2_	Aimei et al.^926^			
	CH_4_	TraPPE^301^			
	H_2_O	OPC4 ^1061^			
	Na^+^Cl^–^ and	Yagasaki et al.^1062^			
	Quartz (Q2)	INTERFACE^1063^			
Ali et al.^1048^	H_2_	Buch^312^	5–80	303–343	1.7
		Marx^310^			
	CO_2_	Cygan et al.^927^			
	CH_4_	^301^			
	H_2_O	SPCE^346^			
	Na^+^Cl^–^ and	Joung and Cheatham^371^			
	Kaolinite	^422^			
Chen and Xia^605^	H_2_	IFF^313^	5 to 60	320	0
	H_2_O	TIP4P/2005 ^1042^	(steps of 5)		
	SiO_4_ (Q^2^ Silica)	INTERFACE^425^			
	SiO_4_ (Q^4^ Silica)	INTERFACE^425^			
Chen et al.^604^	H_2_	IFF^313^	10	300, 400	0 and 2.22
	H_2_O	TIP4P/2005 ^1042^			
	SiO_4_ (Q^2^ Silica)	INTERFACE^425^			
	Brine : Na^+^ and Cl^–^	Madrid-2019 ^1034^	10	300, 400	NA
Al-Yaseri et al.^606^	H_2_ (G)	IFF^313^		300	
	H_2_O	SPC/E^346^			
	Na^+^	Aqvist^1044^			0.5
	Cl^–^	Chandrashekhar^1059^			0.5
	Humic Acid	OPLS-AA^360^			
	SiO_4_	CLAYFF^422^			
Al-Yaseri et al.^607^	H_2_	IFF^313^	5, 20	300, 323	
	H_2_O	SPC/E^346^			
	Na^+^, Ca^2+^, Mg^2+^,	Aqvist^1044^			0.001, 0.007
	Cl^–^	Chandrashekhar^1059^			
	SiO_4_ (S, Silica)	CLAYFF^422^			
	CaCO_3_ (S, Calcite)	Xiao^1064^			
Yang et al.^602^	H_2_	IFF^1060^	1 to 160 MPa	298 to 523 K	0
	H_2_O	TIP4P/2005 ^1042^			
	SiO_4_ (Q^3^/Q^4^ Silica)	INTERFACE^422^			
	C_175_H_102_O_9_N_4_S_2_ (II-D, Kerogen)	CVFF^427^			
Zheng et al.^608^	H_2_	Three-site model^1060^	1 to 30 MPa	338 K	0, 0.5, 1
	CH_4_	OPLS-AA^360,1065^			
	H_2_O	SPC/E^346^			
	Na^+^, Cl^–^	Joung and Cheatham^371^			
	SiO_4_ (Quartz) (Q^3^/Q^4^ Silica)	CLAYFF^422^			
Zheng et al.^609^	H_2_	Three-site model^1060^	1, 10, 20, and 30	338	0
	H_2_O	SPC/E^346^			
	SiO_4_ (S, Q^3^/Q^4^ Silica)	CLAYFF^422^			
Alshammari et al.^1049^	H_2_	IFF^313^	0.1–70	293–373	NA
	CH_4_	OPLS-AA^360,1059^			
	CO_2_	Cygan et al.^927^			
	H_2_O	SPCE^346^			
	Na^+^Cl^–^	OPLS-AA^360^			
	Mg^2+^	Aqvist^1044^			
	Calcite	Xiao et al.^1064^			
	Silica	INTERFACE^1063^			
Barbosa et al.^1050^	H_2_	Buch^312^	10	323	20 wt%
	CO_2_	Cygan et al.^927^			
	CH_4_	TraPPE^301^			
	H_2_O	SPCE^346^			
	Na^+^Cl^–^	Joung and Cheatham^371^			
	Kaolinite (siloxane, gibbsite)	CLAYFF^422^			
Fatah et al.^1051^	H_2_	IFF^313^	6.9	298.15	NA
	N_2_	IFF^313^			
	CO_2_	Cygan et al.^927^			
	CH_4_	OPLS-AA^360^			
	Na^+^, Mg^2+^, Ca^2+^	Aqvist^1044^			
	Cl^–^	OPLS-AA^360^			
	SO_4_^2–^	Cannon et al.^1066^			
Ghafari et al.^1052^	H_2_	IFF^313^	10–30	333–413	0
	CO_2_	Cygan et al.^927^			
	CH_4_	TraPPE^301^			
	N_2_	IFF^313^			
	H_2_O	SPC/E^346^			
	Na^+^Cl^–^	Smith and Dang^374^			
	Silica	INTERFACE^1063^			
		CLAYFF^422^			
		DDEC^1067^			
		BKS^1068^			
Phan et al.^1053^	H_2_	Lopez-Lazaro et al.^675^	15	333	3.4 NaCl and 0.13 KCl
	CO_2_	EPM2 ^419^			
	CH_4_	TraPPE^301^			
	H_2_O	SPC/E^346^			
	Na^+^, K^+^, Cl^–^	Dang^1043^			
	Talc	CLAYFF^422^			
	Kaolinite	CLAYFF^422^			
Yao et al.^1054^	H_2_	IFF^313^	14–150	323–423	0–5.4
	H_2_O	TIP4P/2005 ^305^			
	Na^+^, K^+^, Cl^–^	CLAYFF,^422^ Madrid-2019 ^1034^			
	Silica	INTERFACE^1063^			
Yu et al.^1056^	H_2_	Hirschfelder et al.^321^	5–40	292–343	
	CO_2_	CVFF^1069^			
	CH_4_	CVFF^1069^			
	H_2_O	SPC/E^346^			
	Graphene	Not mentioned			
Zhang et al.^1057^	H_2_	IFF^313^	5–25	300-400	0–1 mol/L
	H_2_O	SPC/E^346^			
	Halite NaCl	Joung and Cheatham^371^			
	Quartz	CLAYFF^422^			
	Calcite	Xiao et al.^1064^			
	Montmorillonite	CLAYFF^422^			
	Na^+^Cl^–^	Smith and Dang^374^			

a The salt concentrations in brine are reported in units of molality, or kgs of salt per kg of solvent (H_2_O). The gaseous (G), liquid (L) and the solid (S) phases and the respective force fields used to model the interactions between the like molecules are provided. The final column lists the thermodynamic conditions at which CAs were computed. Note that the salt concentrations reported by Barbosa et al.^1050^ and Zhang et al.^1057^ are in weight percentage and mol/L, respectively

### 4.11 Atomistic Modeling of Hydrogen Embrittlement

HE poses a critical threat to structural integrity by severely degrading the ductility and toughness of metallic materials, leading to premature brittle fracture even under low external stresses. At the atomistic scale, HE involves several distinct, yet interconnected mechanisms, as shown in Fig. 71, all of which have been intensively studied using MD simulations.

**Figure 71:**
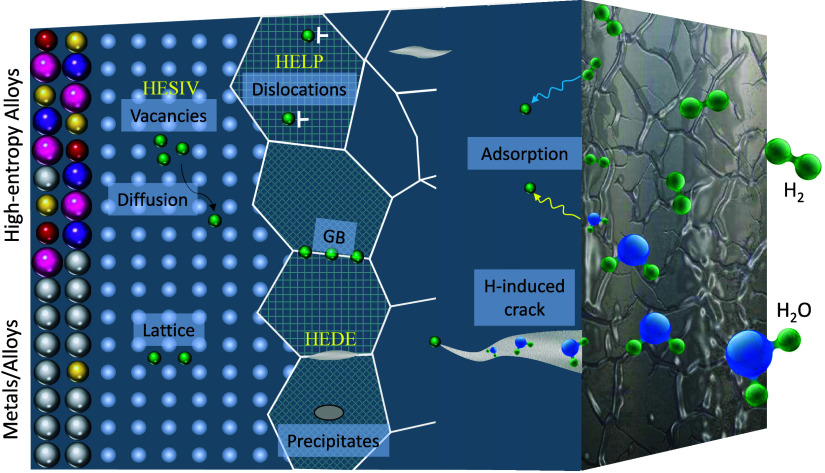
Illustration of hydrogen embrittlement mechanisms in high-performance materials (metals, alloys, and high-entropy alloys) with microstructural defects. Key processes include: (1) hydrogen adsorption, trapping, and diffusion; (2) hydrogen-defect interactions; and (3) the progression of hydrogen-induced damage. The figure emphasizes the interplay of these factors and their role in activating HE mechanisms, HEDE,^1070^ HELP,^1071^ HESIV.^1072^

H-enhanced decohesion (HEDE)^1070^ occurs when H atoms segregate and accumulate at interfaces, GBs, and atomic-scale defects, significantly lowering the cohesive strength between atomic planes, and thus, promoting brittle intergranular or cleavage fracture. On the other hand, H-enhanced localized plasticity (HELP)^1071^ describes the H-driven reduction of barriers for dislocation motion, leading to increased dislocation activity. While HELP initially facilitates plastic deformation, it ultimately results in severe strain localization and accelerated crack initiation due to locally intense deformation. Additionally, H-enhanced strain-induced vacancy formation (HESIV)^1072^ emerges when H atoms stabilize vacancies, effectively lowering vacancy formation energies, which generates a higher density of defects and provides further preferential sites for crack initiation and growth. H also affects crack propagation dynamics directly by promoting dislocation emission or, conversely, inhibiting dislocation nucleation near crack tips, depending on the H concentration and the local stress state. Furthermore, H-dislocation interactions modify dislocation core structures and mobility, affecting overall material plasticity, fracture toughness, and resistance to fatigue crack growth. MD simulations uniquely enable direct visualization and quantification of these atomistic phenomena, providing detailed insights into the role of H at different length scales, defect configurations, and loading conditions. The complexity of H-induced effects is further magnified by the random and complex chemical environments, characteristic of high-entropy alloys (HEAs). For this reason, ML approaches have emerged as critical tools for navigating these complexities. In the following sections, we explore these mechanisms in detail and review the relevant literature.

#### 4.11.1 Atomic Hydrogen Diffusion in Metallic Materials

H diffusion is critical for understanding and mitigating the detrimental effects of HE, as the migration of H atoms within the material microstructure precedes the degradation of mechanical properties. MD and KMC are commonly used to study H diffusion, with the simplest case being diffusion in a perfect lattice. For example, Zhou et al.^545^ conducted a parametric study using MD simulations to investigate H diffusion in fcc Al (diffusion barriers and coefficients). This work provides a foundational reference for MD studies of H diffusion. However, MD simulations depend on empirical force fields, such as the EAM potential, which often lack sufficient accuracy as thoroughly discussed in Section 3.1. An alternative approach is KMC simulations with predefined energy barriers obtained from DFT-based NEB calculations. A key advantage of KMC is the ability to incorporate ZPE corrections into the energy barriers, accounting for harmonic quantum effects. Yang and Oyeniyi^1073^ performed KMC simualtions with ZPE–corrected barriers to study H diffusion in bcc W, achieving excellent agreement with experimental results. To fully capture quantum effects, e.g., proton tunneling, more advanced techniques such as PIMD and higher-accuracy force fields are required. Kimizuka et al.^546^ demonstrated that in bcc Fe, quantum effects not only substantially reduce the H diffusion barrier but also alter the preferred diffusion pathway, even at room temperature.

Defects, such as vacancies, impurities, dislocations, GBs, and other interfaces play a critical role in H diffusion, as they significantly alter the energy landscape of diffusion pathways. MD simulations using EAM potentials have revealed key insights into these effects. The study by Zhu and Oda^1074^ revealed that in bcc Fe, vacancies act as strong trapping sites, substantially reducing the effective H diffusivity. The authors showed that while bulk-dissolved H atoms dominate diffusion, trapped H at vacancies contributes negligibly to long–range diffusion of H. Similar suppression of H diffusion due to vacancies and dislocations has been observed in bcc W through MD simulations.^1075^ The influence of GBs has also been extensively studied, particularly in nano-grained materials, where a large fraction of atoms resides near interfaces. Zhou et al.^1076^ systematically investigated H diffusion in nanograined Fe by means of MD simualtions, and showed that decreasing grain size restricts H diffusion due to the increased density of triple junctions.

Beyond the inherent inaccuracies of empirical force fields, such as EAM, another critical limitation in modeling H diffusion near defects is the constrains of accessible time-scales by MD simulations. Given the femtosecond–scale time steps required to resolve H diffusion, an impractically large number of MD steps would be needed to capture diffusion near defects, particularly at room and low temperatures. To overcome these challenges, KMC simulations incorporating DFT-derived diffusion barriers have emerged as a powerful alternative for studying H-defect interactions. Implementing this approach requires either precomputing all possible H diffusion pathways around defects to construct an event table for KMC or using advanced on-the-fly methods like the kinetic Activation-Relaxation Technique (k-ART) to dynamically identify diffusion paths during simulations. Zhou et al.^541^ performed classical KMC simulations with DFT-derived barriers to investigate H diffusion across various GBs in fcc Ni. The authors demonstrated that high–angle GBs enhance H diffusion, whereas low-angle GBs hinder it. In a separate study, Zhou et al.^653^ combined k-ART with KMC to examine H pipe diffusion along both edge and screw dislocations in fcc Ni (Fig. 72) and bcc Fe. This advanced computational framework enabled simulations spanning micro-second to second time-scales, revealing an unexpected suppression of H diffusion along dislocation cores, a phenomenon inaccessible to conventional MD simulations due to time-scale limitations.

**Figure 72:**
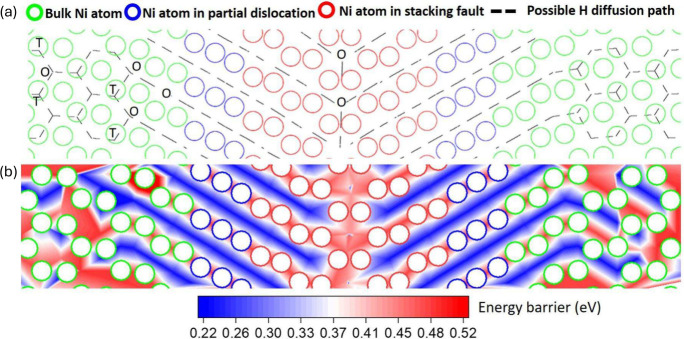
(a) Possible atomic hydrogen diffusion pathways on the glide plane and (b) the corresponding diffusion barrier contour map for an edge dislocation in fcc Ni. Octahedral (O) and tetrahedral (T) sites are indicated, with tetrahedral sites overlapping the host Ni atoms. Diffusion hops between adjacent sites are connected by dashed lines. [Reprinted from Acta Materialia, Vol 268, Xiao Zhou et al., Critical assessment of hydrogen pipe diffusion at dislocations in metals, 119758, Copyright (2024), with permission from Elsevier].

From a methodological standpoint, both MD and KMC present distinct advantages and limitations for studying H diffusion in metals. MD captures the true dynamic evolution of the system, but its accuracy is inherently constrained by the quality of the force field. Moreover, MD becomes computationally prohibitive at low temperatures when high diffusion barriers necessitate excessively long simulation times. In contrast, KMC readily incorporates DFT–derived energy barriers with ZPE corrections, offering improved accuracy for H diffusion processes. Nevertheless, conventional KMC implementations neglect the temperature dependence of diffusion barriers and face two significant challenges: (1) the need to explicitly define all possible H diffusion pathways and the associated barriers within defective regions, and (2) the low–barrier problem where simulations may become trapped in local energy basins as discussed by Andersen et al.^650^ These limitations underscore the urgency for advanced MD and KMC approaches capable of addressing such complexities in H diffusion studies. MLIPs (introduced in Section 3.1.5) show promise for addressing these challenges, combining accuracy and efficiency. A detailed discussion of these advanced methods will be presented in Section 4.11.7.

#### 4.11.2 Atomic Hydrogen Trapping and Hydrogen-Mediated Vacancy Behaviors

H diffusion in metals and alloys is strongly governed by trapping phenomena, whereby H atoms become confined in sites with high escape barriers that retard their migration. These traps include interstitial impurities, such as carbon and nitrogen, substitutional solutes (e.g., Nb, Ti, Mg, Sc, and Y), intrinsic crystal defects including vacancies, nanovoids, dislocations, and GBs, and secondary-phase interfaces formed by carbides (TiC, V_4_C_3_, NbC, Mo_2_C) or dispersed oxides, as reveled by Sagar et al.,^1077^ Zhang et al.,^1078^ Liu et al.^1079^ and Wang et al.^1080^ H trapping at GBs or precipitate–matrix interfaces can lead to HEDE, in which H reduces the cohesive strength of the GBs and the interfaces. DFT calculations and experimental measurements indicate that lattice defects (dislocations and GBs) and precipitates with coherent interfaces, typically exhibit trapping energies below 50 kJ/mol (often regarded as the reversibility limit), while semi- or incoherent precipitates can trap H much more strongly, with energies reaching up to 129 kJ/mol. For a thorough review of the available DFT and experimental studies the reader is referred elswhere.^1081^

H profoundly alters vacancy behavior in metals. The associated HE mechanism is known as HESIV, which hypothesizes that the density and clustering of vacancies is enhanced in the presence of H. Consequently, the vacancies can coalesce to form micro-voids, which in turn may combine to form larger voids leading to a decrease in ductile crack growth resistance. Hayward and Fu^544^ combined MD and DFT to show that in bcc Fe, vacancy formation energies drop sharply as local H concentration increases, becoming negative at high coverage, and thus, provide an atomistic explanation for HESIV. Echeverri Restrepo et al.^1082^ used parallel-replica dynamics alongside NEB calculations to quantify mono-vacancy diffusivity in bcc Fe with 0–3 H atoms. This study revealed that a single H raises the activation energy, slowing diffusion, whereas two to three H atoms lower the barrier, restoring or even enhancing vacancy mobility. Du et al.^1083^ used a potential-of-mean-force method to investigate vacancies in Cu and Pd, and demonstrated that H enrichment at the saddle point (positive H Gibbs excess energy) lubricates vacancy migration, and significantly boosts diffusivity. These studies demonstrate that H creates superabundant vacancies by lowering formation energies, traps them at low coverage but enhances their diffusion at high coverage via saddle-point enrichment, and therefore, accelerates void nucleation and growth, leading to HE.

#### 4.11.3 Dislocation Nucleation and Mobility

H exerts a dual, system–dependent influence on incipient plasticity. In many fcc metals it facilitates dislocation nucleation by lowering the shear modulus, the line energy, and—when it enters the lattice—the intrinsic and extrinsic stacking-fault energies. These trends underlie the HELP signature reported by Barnoush and Vehoff^1084^ during nano-indentation, where the first “pop-in” event is a direct measure of the nucleation of dislocation half-loops beneath the indenter. The MD study by Zhou et al.^547^ refined this picture: in fcc Ni and Pd the pop-in load drops, primarily because interstitial H produces an isotropic lattice swelling that acts as a pre-strain, while little direct H–dislocation interaction is required. The GCMC simulations of Yu et al.^552^ revealed that the H-induced increase in the core radius and decrease in the core energy of dislocations are the key factors affecting dislocation activities including dislocation nucleation, emission, mobility, and reaction that leads to HE. Equally well documented are cases where H suppresses dislocation nucleation. Early NEB calculations for fcc Ni crack tips performed by Wen et al.^1085^ showed that adsorbed H raises the activation energy, and therefore, the stress-intensity factor for loop emission, an effect traced to a marked reduction in the activation volume when H decorates the embryo (i.e., the initial, critical-sized nucleus of a dislocation loop). Yin et al.^548^ studied Ag–H nanowires both experimentally and by means of MD/NEB. The authors showed that surface–adsorbed H at ledges and steps increases the critical stress for loop formation, with the computed activation barrier rising accordingly. Here, H lowers the local surface energy, and relaxes the tensile stress concentration, effectively “locking” the most efficient surface sources. Using MD simulations coupled with NEB calculations, Li et al.^549^ systematically elucidated the dual effect of H on dislocation nucleation: surface-segregated H raises the activation barrier, thus, inhibiting dislocation emission, while bulk-dissolved H lowers the barrier, thereby, promoting nucleation.

H can also profoundly alter the mobility of preexisting dislocations in metals, a topic that has been extensively explored using MD and other atomistic simulation approaches. Early DFT calculations combined with a line tension model by Itakura et al.^1086^ showed that the effect of H on screw dislocation glide in bcc Fe depends sensitively on temperature, H concentration and applied stress. In that study, H both promoted kink nucleation, thereby softening the lattice, and trapped kinks behind the dislocation core, thereby producing a hardening effect. For a bulk H concentration of 0.1 parts per million (ppm), the softening window extended from ca. 200 to 300 K; at ten ppm it shifted to a range of 300 to 400 K. The subsequent NEB calculations and room temperature MD simulations of Yin et al.^548^ confirmed these predictions. At a H line density of 0.4 per nanometer, the kink nucleation barrier decreased from 0.47 to 0.42 eV, and the critical shear stress for glide decreased from 32 to 22 MPa, consistent with experimental observations of H enhanced screw dislocation mobility. MD simulations by Song and Curtin^1087^ revealed that H slows edge dislocation motion in bcc Fe by rapidly forming Cottrell atmospheres, which exert a strong solute drag effect over a wide concentration range. Kapci et al.^550^ conducted GCMC simulations coupled with MD to further show that increasing the H concentration raises the critical stress required for edge dislocation depinning, underscoring the pervasive influence of H on dislocation based plasticity in Fe.

#### 4.11.4 Crack Propagation and Grain Boundary Decohesion

Song and Curtin^276^ used atomistic MD and GCMC to show that, under stress, H concentrates at crack tips in single- and bi-crystal Ni to form nano-scale hydrides that suppress dislocation emission and inhibit crack-tip blunting. Song and Curtin^1088^ then integrated H diffusion kinetics with criteria for dislocation emission and cleavage into a single nondimensional parameter that, without any fitting constants, predicts the ductile-to-brittle transition across different Fe-based alloys based on H concentration, temperature and loading rate. For fcc Ni, Tehranchi and Curtin^442^ showed that H segregation reduces both the critical stress for dislocation emission and the cleavage fracture stress intensity at a range of GBs, yet under conditions of only initial equilibrium segregation, and no active diffusion, a ductile-to-brittle transition was not observed, and the Σ3(111) twin boundary remained essentially immune to HE. In a follow-up study, Tehranchi et al.^1089^ explicitly coupled H nano-diffusion onto freshly-created crack surfaces with mechanical, thermodynamic and kinetic modelling to derive a quantitative, parameter-free criterion that predicts the onset of HE for both crack propagation and GB cleavage. The mechanistic and predictive frameworks developed in these studies are essential for guiding the design of HE–resistant materials. Jung et al.^553^ conducted systematic MD simulations of crack propagation along GBs in bcc Fe, with and without H. The findings showed that the effects of H on crack growth vary significantly with GB character. Specifically, the Σ3 coherent twin boundary (CTB) markedly retards crack propagation, owing to its capacity to accommodate plastic deformation, indicating that CTBs can substantially enhance resistance to HE. These results underscore the promise of GB engineering as a strategy for improving materials resistance to HE.

Several atomistic MD studies have quantitatively assessed H–induced decohesion in Ni GBs. Tehranchi and Curtin^1090^ systematically investigated 110 symmetric–tilt boundaries in fcc Ni, computing both the theoretical strength (rigid separation) and the yield strength (onset of dislocation emission), and showed that equilibrium H segregation has negligible impact on either metric. Li et al.^1091^ extended this analysis to a broader spectrum of GB characters, reporting that, under equilibrium H coverage, the maximum tensile strength and fracture energy of the Σ5(210)­100 and Σ17(530)­100 boundaries decrease by only 6.6% and 15.8%, respectively. Crucially, by incorporating plastic deformation into the simulations, Li et al.^1091^ demonstrated that the synergy between H–facilitated decohesion and dislocation activity can notably amplify embrittlement. Huang et al.^1092^ further combined a polyhedral packing–unit identification of all interstitial trap sites with atomistic segregation energies and Rice–Wang thermodynamics to predict work–of–separation reductions under different fracture rates: in the fast–fracture (constant concentration) limit H lowers the work of separation by 10%, whereas the reduction approaches 50% in the slow-fracture (constant chemical potential) limit. Collectively, these works show that equilibrium H segregation alone weakens Ni GBs only modestly, while H diffusion kinetics and plasticity critically govern the full extent of HE.

### 4.11.5 Hydrogen Effects on Complex Deformations

MD simulations have become indispensable for clarifying how H modulates nanoscale plasticity and fracture. Focusing on dislocation-GB interactions, Adlakha and Solanki^1093^ showed that interstitial H raises the activation barrier for screw-dislocation transmission across 111 tilt GBs in bcc Fe. Using [110] symmetric tilt GBs, Li et al.^1094^ showed that H shifts the reaction pathway from dislocation transmission to dislocation absorption, concomitantly increasing the initiation barrier. Tensile loading of H-segregated bicrystals conducted by Wan et al.^1095^ further reveals complex coupled deformation mechanisms, providing an atomistic basis for embrittlement scenarios governed by dislocation-GB interactions. In polycrystalline models, the H effects on the mechanical properties become even richer. Zhou et al.^1096^ demonstrated that decreasing the grain size in bcc Fe enhances resistance to HE, both by diluting H concentration at GBs and by activating a broader spectrum of GB-mediated deformation modes. For fcc Ni, Kuhr et al.^1097^ observed an H-induced increase in yield strength at small strains, followed by accelerated dislocation emission from GB sources at larger strains. MD simulations of penta-twinned Ag nanowires by Yin et al.^548^ revealed that surface-adsorbed H not only elevates the yield strength but also shifts the failure mode from distributed plasticity to localized necking. Surprisingly, MD simulations of HCP Mg polycrystals by Ji et al.^1098^ revealed that H can enhance ductility without sacrificing strength.

From the perspective of multiscale modeling, Peng et al.^292^ employed a concurrent atomistic–continuum (CAC) framework to simulate crack initiation at H-charged GBs in bi-crystalline bcc Fe, bridging atomistic H diffusion, nanoscale GB cavitation/cracking, and mesoscale dislocation activity in a single model. The authors showed that tensile loading drives H toward the GB, where plasticity-induced clustering of H (PICH) acts as a mechanistic link between HELP and HEDE, greatly increasing local stress concentrations and weakening GB cohesion. Distinct failure modes emerge depending on GB character: Σ3 boundaries undergo micro-twinning–assisted void nucleation and coalescence, while Σ9 boundaries fail via crack initiation and growth concurrent with dislocation emission. By capturing long-range stress fields at sub-micron scales, their CAC simulations predict significantly lower GB cohesive strengths than conventional MD, and provides parameters for cohesive zone models, underscoring the necessity of multiscale simulation to reliably describe HE.

### 4.11.6 HE in High-Entropy Alloys

High-entropy alloys (HEAs), also known as multi-principal element alloys (MPEAs), have garnered significant research attention across various hydrogen-related fields, spanning H production, storage, and HE prevention. Recent experimental studies demonstrate exceptional HE resistance in HEAs, where H-charged specimens maintain higher strength while retaining comparable plasticity to their H-free counterparts (Luo et al.^1099,1100^ and Li et al.^1101^). Two primary mechanisms have been proposed to explain this behavior: (1) significantly reduced H diffusion due to the complex chemical environment in HEAs, and (2) H-induced reduction in stacking fault energy, promoting stacking fault formation during deformation. These findings have spurred extensive investigations into H solubility, diffusion behavior, and deformation mechanisms in HEAs with H.

Unlike conventional metals and simple alloys, HEAs exhibit a non-uniform distribution of H solution energies even within an otherwise pristine lattice. The random spatial arrangement of multiple metallic elements produces a correspondingly random potential-energy landscape for interstitial H. DFT has therefore become the method of choice for quantifying site-to-site variations in solution energies and migration barriers. For equiatomic FeCuCrMnMo, Ren et al.^1102^ demonstrated that the pronounced lattice distortion intrinsic to HEAs leads to large fluctuations in H migration barriers. In fcc FeMnCrCoNi, Xie et al.^1103^ likewise reported a broad distribution of local solution energies; trapping in low-energy sites raises the effective diffusion barrier and thus slows long-range diffusion. Extending these insights, Yin et al.^1104^ showed that chemical short-range order (CSRO) in fcc CrCoNi provides particularly deep traps, making CSRO an additional and tunable determinant of H diffusivity.

H diffusion in HEAs is inherently more intricate than in elemental metals. Because the governing timescales far exceed those accessible to direct AIMD, a variety of ML approaches have been developed to retain DFT-level fidelity at tractable cost. A comprehensive survey of ML techniques is given in Section 4.11.7. In fcc FeMnCrCoNi, Zhou et al.^654^ trained ML models on DFT data to predict H solution energies and migration barriers within the random chemical environment; these energetics subsequently informed KMC simulations of long-range diffusion. Their analysis shows that H most stably occupies the octahedral site, while larger Co, Cr, and Mn fractions reduce the diffusion coefficient, whereas additional Fe or Ni has the opposite effect. In bcc MoNbTaW, Shuang et al.^468^ integrated an MLIP, neural-network KMC, and symbolic regression into a unified framework capable of accessing experimental timescales for H diffusion. The study reveals a previously unreported super-Arrhenius temperature dependence, traced to the spontaneous emergence of nanoscale clusters enriched in H-favourable elements (Nb, Ta), as shown in Fig. 73. Disruption of these clusters by CSRO accelerates H diffusivity, thereby underscoring the critical interplay between local chemical order and diffusion kinetics.

**Figure 73:**
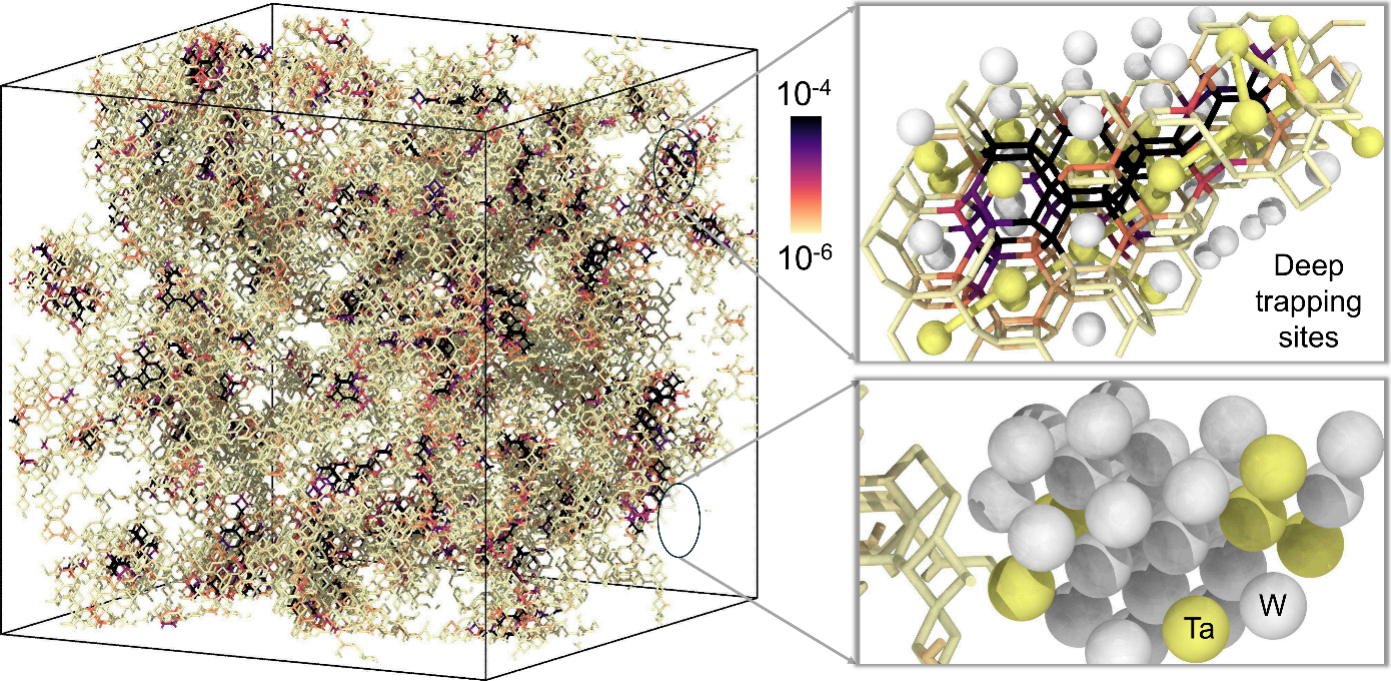
Visualization of H visitation frequencies at accessible tetrahedral (T) interstitial sites in Ta_20_W_80_ at 500 K. Frequencies are calculated by normalizing the visitation count of each T site by the total number of jumps in KMC simulations. The top-right and bottom-right panels highlight the metallic environments near trapping sites and inaccessible sites, respectively. H diffusion pathways between adjacent T sites are represented as atomic bonds. [Reprinted from Acta Materialia, Vol 289, Shuang et al.,^468^ Decoding the hidden dynamics of super-Arrhenius hydrogen diffusion in multi-principal element alloys via machine learning, 120924, Copyright Elsevier (2025) (open access article distributed under the terms of the Creative Commons license - CC BY 4.0)].

H profoundly alters the deformation mechanisms and mechanical properties of HEAs. Using an EAM potential parameterized for FeNiCr-H, Zhou et al.^444^ showed through extensive MD simulations that H statistically widens stacking faults by lowering both intrinsic and extrinsic stacking-fault energies. Building on the same potential, Guo et al.^1105^ uncovered a dual influence of H on twinning: it facilitates homogeneous twin nucleation yet suppresses surface-initiated twinning. To provide higher-fidelity benchmarks beyond classical potentials, DFT calculations have long been employed. Xie et al.^1103^ demonstrated that electron transfer from H to the lattice reduces both unstable and stable stacking-fault energies, thereby favoring deformation twinning. Systematic DFT calculations by Zhou and Curtin^1106^ for 304/316 stainless steels and FeMnCrCoNi HEAs quantified H adsorption, surface energies, and GB fracture energies, illuminating the atomistic origins of HE resistance. The same DFT-guided strategy, coupled with mechanistic modelling, subsequently identified CoNiV as an exceptionally HE resistant alloy.^1107^ In a related study, Tan et al.^1108^ showed that CTBs in fcc HEAs retard crack propagation and thus enhance HE resistance. To combine MD length- and time-scales with DFT accuracy, Zhou et al.^469^ recently developed a MLIP for FeCoNiCrMn-H. Their simulations revealed that H lowers stacking-fault energy and promotes twinning, while dynamically formed local chemical ordering (LCO) traps H to create LCO–H complexes that pin dislocations more effectively than LCO alone. Moreover, segregation-induced Cr enrichment and Fe depletion at GBs raise the GB fracture energy, further improving HE resistance.

### 4.11.7 Machine Learning in Hydrogen Embrittlement Studies

The emergence of AI has revolutionized materials research (see also Section 4.6.2), with ML becoming a major tool for advancing our understanding of HE in metallic systems. ML techniques are transforming all aspects of HE research, spanning all scales from atomistic simulations of H diffusion to macroscopic predictions of mechanical properties in H-charged materials. Among various ML applications, the development of MLIP represents one of the most significant advances, enabling accurate large-scale simulations while maintaining near-DFT level accuracy. MLIPs typically require sophisticated ML architectures containing approximately 10^3^ trainable parameters that are optimized against extensive DFT datasets. Widely used MLIPs are the neural network potential (NNP),^460^ Gaussian approximation potential (GAP),^461^ spectral neighbor analysis potential (SNAP),^1109^ moment tensor potential (MTP),^462^ and atomic cluster expansion (ACE).^1110^

Kwon et al.^465^ developed MTPs to investigate H diffusion in pristine bcc metals (Nb, Fe, W), using PIMD to demonstrate the significant influence of nuclear quantum effects below 500 K across a 100–1000 K temperature range. Similar progress in fcc systems includes NNPs coupled with PIMD, which captured the quantum mechanical aspects of nonlinear H isotope diffusion in Pd.^464^ The Bravais-Inspired Gradient-Domain Machine Learning (BIGDML) approach^1111^ achieved comparable accuracy for H diffusion in Pd, while utilizing compact DFT datasets (10–200 structures). Other applications include H diffusion in hcp Mg and Ti hydrides using MTP, MACE, and CHGNet potentials.^466^ Complementing these diffusion studies, Meng et al.^467^ created a general-purpose MLIP for Fe-H systems capable of modeling diverse phenomena including surface processes, defect-mediated diffusion, and H-influenced plasticity.

Additionally, MLIPs have emerged as particularly powerful tools for investigating HE in HEAs, where they successfully capture the complex chemical interactions between H and multiple elements. The field advanced significantly through the work of Shuang et al.^468^ who used D-optimality-based dataset construction to develop a high-accuracy MTP that comprehensively describes H-MoNbTaW interactions across the whole compositional space, including chemical short-range ordering effects (Fig. 74a-c). This MTP enabled the discovery of super-Arrhenius H diffusion behavior in bcc HEAs. Subsequent developments include a Deep Potential model capable of simulating coupled H diffusion and H-influenced plastic deformation in fcc FeMnCrCoNi HEAs, demonstrating the expanding capabilities of ML approaches for these challenging multicomponent systems.^469^

**Figure 74:**
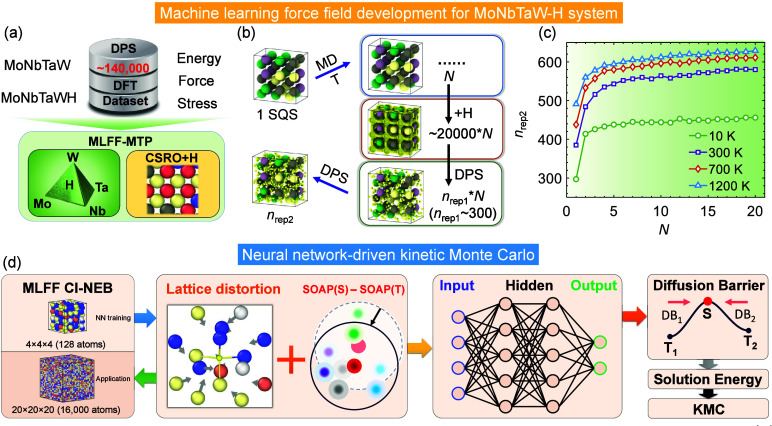
Machine learning-driven exploration of H diffusion with DFT-level accuracy. (a) Workflow for MLIP development. (b) Schematic of the distorted pathway sampling (DPS) method at various temperatures, where *n_rep1_* and *n_rep2_* denote selected configurations after initial and secondary DPS operations, respectively, and *N* represents the number of distorted high-entropy alloy (HEA) configurations at temperature *T*. Large colored spheres indicate metallic atoms, while small yellow spheres represent H atoms. (c) Evolution of representative configurations (*n_rep2_*) through a two-stage DPS process, showing the count of distorted HEA configurations (*N*) at different temperatures. (d) Neural network-driven KMC (NN-KMC) methodology flowchart. [Reprinted from Acta Materialia, Vol 289, Shuang et al.^468^ Decoding the hidden dynamics of super-Arrhenius hydrogen diffusion in multi-principal element alloys via machine learning, 120924, Copyright Elsevier (2025) (open access article distributed under the terms of the Creative Commons license - CC BY 4.0)].

The significance of ML in HE studies extends beyond MLIP applications. Leveraging large-scale DFT datasets and the Sure Independence Screening and Sparsifying Operator (SISSO) algorithm, Korostelev et al.^1112^ developed robust analytical models for accurate prediction of H absorption and migration energetics in metal alloys. For H diffusion specifically, Shuang et al.^468^ implemented a neural-network KMC framework capable of computing hundreds of millions of DFT-accurate diffusion barriers (Fig. 74d), enabling unprecedented long-timescale simulations. The same work further applied SISSO to derive analytical models for H diffusivity in bcc HEAs, providing fundamental insights into diffusion mechanisms within complex chemical environments. Another work by Zhou et al.^654^ established an ML–KMC framework to investigate H diffusion in fcc FeMnCrCoNi HEAs, employing whale optimization algorithms to identify atomic configurations with minimized H diffusivity. Tang et al.^1113^ developed a reinforcement learning–guided transition kinetics simulator and a low-energy state sampler that leverage neural networks to predict transition rates, automatically uncovering the most probable diffusion pathways without explicit barrier calculations and enabling efficient long-timescale atomistic simulations.

## 5 Challenges and Future Outlook

### 5.1 Future Considerations on Molecular Simulation of Hydrogen in Geological Formations: From Molecules to Reservoir-Scale

Accurate prediction of H_2_-brine IFTs, adsorption energies, and transport properties requires high-quality experimental data, mainly for training force fields and validating MD simulations. Based on, but not only limited to, our analysis of the available molecular simulation literature reviewed in Sections 4.7.1, 4.9, and 4.10, significant discrepancies in MD results can be seen. These often stem from the varying treatment of electrostatic interactions, cut-off distances, choice of force fields, surface model, all of which must be carefully validated against experiments. These discrepancies become more pronounced when modeling multicomponent systems (e.g., H_2_ mixed with H_2_O, CO_2_, CH_4_, and N_2_) across an extended range of pressures, temperatures, and salinities. Therefore, a key research direction that we are confident should be explored in the future is the systematic investigation of IFTs, contact angles, and other interfacial properties under varying conditions, using both MD simulations and experimental measurements to refine interaction parameters. In parallel, ML methods, such as the approach demonstrated by Omrani et al.^1114^, show great potential for developing predictive models that capture the dependencies of e.g., IFT on gas composition, thermodynamic conditions, and salinity. Nevertheless, robust experimental datasets for a wide range of H_2_ properties (both for the pure component and mixtures) remain essential to validate these computational frameworks, ensuring accuracy and applicability for real–world processes such as UHS and CO_2_ sequestration.

A thorough understanding of H_2_ wettability in subsurface environments requires accurate experimental measurements, molecular modeling, and reservoir-scale simulations. Although significant progress has been made in molecular modeling, particularly in the parameterization of force fields and the characterization of mineral surface functionalities, important challenges remain. Addressing these is crucial for advancing H_2_ storage technologies and improving our predictive capability of H_2_ behavior at reservoir-scales. Some existing studies have already highlighted discrepancies in pressure-dependent behavior (e.g., Alavi^336^ vs. IFF Wang et al.^313^ models). Thus, future work should focus on systematically validating and refining force fields, especially for high-pressure regimes, against comprehensive experimental datasets. Efforts could include re-parameterizing H_2_ models to better capture the pressure sensitivity observed in H_2_–H_2_O interfacial systems. Also, many simulations assume uniform silanol distributions (e.g., Q^2^, Q^3^, Q^4^). This approach, although providing a simple foundation, it does not reflect the actual rock surfaces (i.e., natural formations exhibit heterogeneous surfaces and multi-mineral interfaces). Extending MD simulations to capture such compositional complexities spanning organic matter, multivalent ions, and varying functional-group densities, will yield more accurate predictions of in-situ wettability. Discrepancies between simulation papers in contact-angle computations (e.g., Q^2^ silica) emphasize the importance of robust sampling and sufficient equilibration. Although some experimental data exist for H_2_–H_2_O–solid contact angles, coordinated efforts where experiments and simulations target identical minerals, surface treatments, and thermodynamic conditions are urgently needed. Such collaborative studies would reduce uncertainties in force-field validation and identify key parameters controlling H_2_ wettability under realistic subsurface conditions.

While molecular simulations have greatly helped the community in understanding the intrinsic H_2_ – H_2_O – solid interactions, they are still almost exclusively performed in ideal, nm-scale slit pores or on perfectly flat crystal faces. Since molecular simulation cannot reach the cm-scale representative elementary volumes (REVs), multiphase transport modelling cannot be accurately performed. Natural reservoir rocks exhibit tortuous pore networks, angular throats, surface roughness, and mosaics of mineral and organic coatings. Together these features generate broad, hysteretic distributions of apparent contact angles that may depart markedly from the single, intrinsic value obtained on a smooth, chemically uniform surface.

Lastly, it is important to point out is the scarcity of atomistic work on dynamic phenomena directly relevant to H_2_ storage, e.g., advancing/receding contact angles, meniscus motion, and capillary trapping. In classical MD, Newton’s equations of motion are integrated and momentum is conserved, thereby, recovering Navier–Stokes hydrodynamics at the nm–scale. Thus, MD could in principle capture such processes without added constitutive assumptions. However, in practice, the required length– and time–scales are prohibitive since even a simple cylindrical pore with a length of a few mm would contain on the order of billions of atoms. Moreover, the reliability of existing force fields for transient, far-from-equilibrium configurations, such as rapidly moving three-phase contact lines, has not been evaluated. Systematic benchmarks that compare dynamic contact angles and capillary entry pressures predicted from MD with well-controlled micro-fluidic experiments, are therefore, urgently needed (even before tackling complex rock geometries). In practice, this means using MD to supply key inputs such as the variation of interfacial tension and contact angle with pressure, temperature, and fluid composition to larger-scale solvers (e.g., pore network, lattice Boltzmann, or volume-of-fluid models) that run on cm–scale digital rock geometries. Reliable predictions of H_2_ migration, trapping efficiency, and long–term storage security in geological formations are only possible if the atomistic inputs are carefully validated and combined with simulations at the pore scale and continuum, while guided by experiments.

### 5.2 Machine Learning Perspective on Atomistic Modeling of Hydrogen Embrittlement

ML has significantly advanced our ability to study H diffusion and HE in metals and alloys by providing interatomic potentials that approach first-principles accuracy. As discussed in Section 4.11.7, traditional studies have largely relied on empirical force fields, most commonly variants of EAM or modified EAM potentials, to describe H-metal interactions. While these potentials can capture qualitative trends, they often fail to reproduce quantitatively accurate H binding energies and diffusion barriers, particularly in the presence of crystalline defects such as vacancies, dislocations, and GBs. The emergence of MLIPs, such as NNP,^460^ GAP,^461^ SNAP,^1109^ MTP,^462^ and ACE,^1110^ has begun to address these shortcomings. By training on diverse data sets of H-metal atomic configurations (drawn from DFT), MLIPs can reliably predict H interactions with pristine lattices and with a variety of defects. In practice, this means that vacancy formation energies, dislocation core structures, and GB binding sites can be characterized with near-DFT accuracy, enabling more predictive modeling of H diffusion coefficients and embrittlement mechanisms.

One major challenge in developing MLIPs for H-metal systems is adequately sampling the vast configurational space that arises from complex H-metal environments—particularly around defects and during dynamic processes. Domain expertise alone cannot exhaustively enumerate all relevant local environments. Active learning techniques, which iteratively identify and add the most informative new configurations during MD, have proven useful in accelerating potential development.^1115^ More advanced strategies, such as uncertainty–driven active learning, further enhance efficiency by focusing the sampling on rare events and underrepresented local environments,^1116^ thus, reducing the total number of required DFT calculations. An alternative sampling strategy is to generate a broad, pre-computed pool of H-metal configurations before any DFT calculations are performed.^466^ Universal MLIPs (uMLIPs) can serve as “sampling engines”, driving MD simulations at scale to explore configurational space. Table 21 provides a list with the most important uMLIPs to date, along with information of the associated datasets and archtectures. Although these uMLIPs do not match the accuracy of DFT, they excel at rapidly producing physically relevant atomic structures. Once a diverse pool of configurations is generated, single-point DFT calculations on these snapshots suffice to train a specialized H-metal MLIP, streamlining the workflow.

**Table 21:** State-of-the-Art uMLIP Architectures and DFT Datasets

Architecture	Organization	Dataset	# of structures
M3GNet^1117^	UC San Diego	MPtri	1.58M
CHGNet^1118^	UC Berkeley		
MACE^1119^	University of Cambridge	Alex(sAlex)	30.50M (10.45M)
Orb^1120,1121^	Orbital Materials		
MatterSim^1122^	Microsoft Research AI for Science	OMat24	100.82M
GRACE^1123^	ICAMS, RUB		
SevenNet^1124^	Seoul National University	MatPES-PBE	40M
DPA3 ^1125^	AI for Science Institute, Beijing		
eqV2 ^1126^	FairChem at Meta	MatPES-r2SCAN	40M
eSEN^1127^	FairChem at Meta		

uMLIPs are MLIPs trained on extensive datasets spanning diverse elements, chemical environments, and material classes. Unlike specialized MLIPs (e.g., ACE or MTP) tailored to specific systems, uMLIPs prioritize broad transferability, achieved through training on hundreds of millions of DFT-labeled structures and architectures with hundreds of millions of parameters. Despite their general-purpose nature, modern uMLIPs can approach the accuracy of specialized MLIPs,^1128^ enabling applications ranging from high-throughput materials screening to defect dynamics simulations. Prominent uMLIPs include M3GNet,^1117^ CHGNet,^1118^ Orb,^1120,1121^ eqV2,^1126^ MACE,^1119^ DPA3,^1125^ SevenNet,^1124^ MatterSim^1122^ and eSEN,^1127^ all built on comprehensive DFT collections exceeding one hundred million structures. These collections include the Materials Project trajectory (MPtrj) dataset with 1.58 million structures,^1118^ the Alexandria (Alex) dataset with 30.5 million structures or its subsampled version sAlex with 10.45 million structures,^1129^ as well as the Open Materials 2024 (OMat24) dataset with 100.82 million structures.^1126^ Whereas MPtrj and Alex (sAlex) focus primarily on near equilibrium configurations, OMat24 emphasizes far from equilibrium structures, making it the most extensive inorganic materials dataset to date. More recently two new datasets computed with the Perdew Burke Ernzerhof (PBE) functional (MatPES-PBE) and the revised regularized strongly constrained and appropriately normed (r2SCAN) functional (MatPES-r2SCAN) have become available to further improve consistency and accuracy of next generation uMLIPs.^1130^ Among current models, the eqV2 and eSEN variants trained on OMat24 by the FairChem group at Meta rank among the best for modeling general defects and hydrogen–metal interactions.^1128^ These developments underscore the transformative potential of uMLIP-driven computational modeling in the materials science community by delivering accuracy comparable to DFT and enabling high fidelity predictions of H related trapping, embrittlement, storage and production processes at unprecedented scales.

The main limitation in developing specialized MLIPs is the vast amount of DFT data required, while uMLIPs face inference-speed constraints that typically limit them to systems of tens of thousands of atoms.^1131^ A promising avenue to overcome both challenges is model distillation—deriving a smaller, task specific MLIP from a pretrained uMLIP can bypass much of the DFT sampling effort.^1132^ The critical challenge which lies here is quantifying the uncertainty of uMLIP predictions in order to ensure that the distilled model inherits accurate energy and force predictions without introducing significant errors. Developing robust uncertainty metrics for uMLIPs will therefore be essential. Furthermore, ML techniques extend beyond interatomic potentials to higher-level property predictions. For example, supervised learning models can predict H-trapping energies and migration barriers directly from local structural and chemical descriptors. This capability is especially valuable for studying H diffusion and trapping in complex alloys—such as random solid solutions and HEAs, where enumerating every local environment and associated DFT calculation becomes impractical.^468^ By combining ML-based property estimators with MLIPs, the community can build multiscale frameworks that predict H diffusion, trapping, and HE susceptibility in real-world engineering alloys with unprecedented accuracy.

### 5.3 Challenges in Force Field and Method Development for Predicting Properties of Liquid Hydrogen and Hydrogen Bubbles

This review highlighted many studies investigating the bulk fluid behavior of H_2_. Based on our survey and analysis, clear knowledge gaps can be identified. Some of these gaps are especially relevant for technological applications and, thus, of interest to the chemical industry. Such a case is molecular modeling H_2_ liquefaction. This requires force fields that can accurately capture phase transitions and the ortho-to-para conversion of H_2_. To achieve this accuracy, a force field should automatically adjust the interaction parameters, for example as a function of the temperature. Classical models with such features could be inspired by the approach of Buch,^312^ which uses a quadrupole interaction term to model H_2_ at very low temperatures (1 to 3 K) (see Section 3.1.1). MLIPs can also provide approaches to simulate this behavior. Recent advances in MLIPs for magnetic materials resulted to inter-atomic potentials that are spin-state aware.^1133^ A similar approach could provide accurate predictions of the ortho-to-para conversion as this would directly address the physical change within the H_2_ molecule. Such an advanced model can then be combined with PIMD techniques to predict H_2_ properties relevant to H_2_ liquifaction and transport (i.e. heat capacity, thermal conductivity, viscosity, self-diffusion).

While the main focus of this review is on classical simulation methods of H_2_, we are convinced that MLIPs will blur the barrier between classical and quantum simulations. MLIPs allow for large scale simulations of reactive systems, the modeling of which was limited to AIMD simulations. Recent studies into Grotthuss transfer in water^105,1134–1137^ have shown that MLIPs show a great potential in studying reactive systems. These studies revealed that both the details of the Grotthuss transfer events (i.e., energy barriers, local environment required for Grotthuss transfer), as well as the effects of these reactions on transport properties (i.e., self-diffusion coefficients and electrical conductivities) can be predicted. As discussed in Section 4.1.6, the electro-osmotic drag of H_2_ in nafion membranes also requires accurate modeling of Grotthuss transfer. Only the study by Choe et al.^129^ showed success in computing the EOD, as it combined AIMD and classical MD methods. Reactive MLIPs could provide a more efficient way to compute the EOD for larger system sizes. This will accelerate the simulations by orders of magnitude, and could lead to optimized membrane designs where the structure of the membrane promotes Grotthuss transfer, while minimizing the drag the H_2_ experiences from the membrane.

H_2_ dissolution in aqueous solutions is another area of great interest for advancing H_2_ economy (e.g., for water electrolysis, subsurface storage, fuel cells). The development of fast polarizable force fields for H_2_, H_2_O, and ions (such as the water force field) can lead to accurate descriptions of H_2_ bubble formation in aqueous electrolyte solutions. Capturing bubble formation provides valuable insight into the amount of water that can be dissolved in H_2_ gas. The pressure inside H_2_ bubbles is the highest at the moment of formation, as it inversely scales with bubble radius. Rahbari et al.^110^ demonstrated that water solubility in H_2_ decreases with increasing pressure, making the initial stages of bubble formation critical for predicting water impurities in the H_2_ gas produced by the electrolyzer. Accurate molecular simulations if bubble formation under varying electrolyte compositions can lead to better informed design decisions for electrolysis setups. While MLIPs have shown great promise, we believe that there are still fundamental challenges that need to be addressed before MLIPs can be used in such simulations. Simulating bubble formation requires large systems, and while MLIPs provide significant speed-up compared to AIMD, they still require significant computational resources compared to classical force fields. Additionally, the long-ranged interactions in such systems pose challenges for MLIPs, since many of them implement a (smoothened) cut-off distance for all inter-atomic interactions. This leads to inaccuracies in systems where electrostatic interactions are dominant.^1138^ Some support for long-ranged interactions has been implemented into several MLIPs.^1138–1140^ Further development into MLIPs with less computational requirements, while maintaining the accuracy of the long-ranged interactions, is needed to make MLIPs suitable for simulating bubble formation in aqueous solutions. Lastly, MLIPs require accurate DFT data to train the model. While modern ab initio exchange correlation functionals (i.e. metaGGA and hybrid density functionals) have shown great improvement, they still have significant limitations. Recent studies showed that MLIPs trained on metaGGA functionals yield accurate trends in the computed properties. However, a relatively constant shift in temperature is observed with computations using MLIPs, for example in viscosity^1141^ or melting temperature.^1142^ While theses MLIPs are useful for predicting trends or providing insight into the underlying physics, they might not yet be suitable for predicting properties for industrial applications. Therefore, for MLIPs to become more broadly applicable, a key breakthrough will be the development of training frameworks that incorporate both DFT data and experimental measurements instead of relying on more and more accurate and expensive DFT methods.

### 5.4 Pathways to Advance Research on Hydrogen Hydrates through Molecular Simulation

As thoroughly discussed in Section 4.8, molecular simulation is an indispensable tool for studying H_2_ hydrates. As such, advancing H_2_ technologies utilizing hydrate systems can be aided by exploring novel simulation routes. For example, investigating hydrate structural effects as a result of using different promoters could lead to increased H_2_ storage capacities. Such studies can be coupled with conventional/novel crystal engineering approaches and techniques. The partial replacement of H_2_O molecules by another species could result in modifying the lattice in way that larger cages could provide increased storage capacity. Other routes towards increasing H_2_ storage capacity in hydrates are the screening of hypothetical structures (following the example of nanoporous materials such as MOFs and zeolites) and developing techniques which can reduce H_2_ “leakage” rate out of the hydrate structure. Reducing the number of faces in a given hydrate crystal would function towards the reduction of “leakage”. Alternatively, the use of certain ice structures could provide a solution, since channels can be more efficient storage structures than cages with faces. Studying the effect of flexible cages on H_2_ storage capacity and leakage with molecular simulations, is another promising route.

To reliably predict thermophysical properties of hydrate systems requires a multi-scale approach, i.e., from the atomistic up to the continuum-level. To this end, developing hybrid computational tools, which couple multi-scale modeling methods is essential. Such tools can incorporate information obtained from ab-initio calculations, classical molecular and coarse-grained simulations, and ML predictive methods (e.g., using MLIPs (see also Sections 3.1.5 and 5.2) and/or physics-informed ML approaches).

Other promising future directions for molecular simulation research on H_2_ hydrates are the investigation of confinement effects on the kinetics and thermodynamics of pure and mixed H_2_ hydrate in solid materials, systematic exploration of semiclathrates and hydrate-based H_2_ + CO_2_ gas mixture separations, and coupling hydrate formation/dissociation with industrial processes such as LNG gasification and desalination.

### 5.5 Importance of Open-Source Software for Studying Properties of H_2_ in Polymeric and Composite Materials

Evidently, the vast majority of the studies reviewed in Section 4.4 were conducted using commercial software (mainly Materials Studio^1143^). The use of commercial software instead of open-source comes with advantages and disadvantages. Major advantages are the ease of setting systems up (which is especially useful in the study of polymers and other highly viscous systems), the available pre- and post-processing options, access to a user interface (which may be particularly important in a corporate setting), professional support, and the overall less steep learning curve compared to an open-source code. Additionally, commercial software, such as Materials Studio^1143^ and MAPS,^1144^ provides access to proprietary force fields (e.g., COMPASS^316^). However, the relatively high price and the inaccessibility to the source code, and therefore, the inherent limitation in expanding, debugging, or modifying the scientific software, are the major drawbacks of commercial molecular simulation codes.

Another important point to consider is that since the early days of molecular simulation, algorithms and methods have been developed with an open-knowledge/source mentality, and mainly financed via national funds in United States and EU. Later on, when molecular simulations became more popular and commercial software arose, these algorithms and methods were incorporated in proprietary software. A characteristic example is the pioneering work by Theodorou and Suter,^1145–1148^ who developed efficient methods for modeling polymeric systems, essentially allowing for the creation of initial configurations and relaxation of long hydrocarbons. Another, broader, example showcasing how open-source can aid the progress in the simulation community is LAMMPS,^473,475^ one of the most widely used MD simulators, which is maintained by Sandia National Laboratories. As a final remark, codes such as LAMMPS and GROMACS,^476,477^ being open to modification and enrichment by the simulation community worldwide, usually offer high performance, a plethora of features, access to new methods sooner, and easier implementation of new force fields compared to commercial software. Therefore, open-source software is considered one of the cornerstones of the molecular simulation field. The expansion and maintenance of open-source software should continue to be a priority of both research universities and governmental education bodies.

### 5.6 Transparency and Reproducibility in Molecular Simulation Workflows

In our review, we encountered several challenges related to the clarity and consistency of force field parametrizations in the literature of H_2_ molecular simulation. In some studies, the rationale behind the choice of molecular models or interaction parameters was insufficiently explained. While such choices may be driven by computational efficiency, availability in simulation packages, or alignment with specific properties (e.g., density, viscosity, or interfacial tension), a clear and explicit justification is essential for scientific transparency. Ideally, this should include reference to the properties against which the force field was tuned, as well as known limitations in transferability or range of validity. Moreover, we observed that some studies do not cite the original source of the force field, but instead reference secondary literature that cited the original work. A notable example is the widely used force field from Hirschfelder,^321^ which in its original formulation includes four different parameter variants (see relevant discussion in Section 3.1.1). In cases where studies cite a derivative reference rather than the primary source, it remains unclear whether the authors are fully aware of the specific force field variant used or its original context. We strongly recommend that future studies consult and cite the original work when reporting force field parameters and their origin, to ensure both scientific accuracy and reproducibility.

Another critical issue we observed is the limited availability of raw simulation data. While some studies commendably include detailed results (in tabulated or other form) along with uncertainty estimates, others provide only plots with densely populated data points and no accessible underlying values. This practice significantly hampers reproducibility, introduces uncertainty in data extraction, and complicates efforts to benchmark or validate results across independent studies. We therefore advocate for rigorous data reporting standards, wherein all key outputs (e.g., pressure, energy, interfacial tension, contact angle, diffusion coefficients) are made available in machine–readable formats, along with metadata describing simulation settings. Such transparency not only enhances the credibility of the published work but also prevents unnecessary duplication of computational efforts in future studies. Promoting reproducibility and interpretability through open, well–documented datasets is essential if molecular simulation is to serve as a reliable and cumulative tool for advancing H_2_ technologies.

## References

[1] Palo D. R., Dagle R. A., Holladay J. D. (2007). Methanol Steam Reforming for Hydrogen
Production. Chemical Reviews.

[2] Gunathilake C., Soliman I., Panthi D., Tandler P., Fatani O., Ghulamullah N. A., Marasinghe D., Farhath M., Madhujith T., Conrad K., Du Y., Jaroniec M. (2024). A comprehensive review
on hydrogen production, storage, and applications. Chem. Soc. Rev..

[3] Juangsa F. B., Irhamna A. R., Aziz M. (2021). Production of ammonia as potential
hydrogen carrier: Review on thermochemical and electrochemical processes. International Journal of Hydrogen Energy.

[4] Aziz M. (2021). Liquid Hydrogen:
A Review on Liquefaction, Storage, Transportation, and Safety. Energies.

[5] Cecere D., Giacomazzi E., Ingenito A. (2014). A review on hydrogen industrial aerospace
applications. International Journal of Hydrogen
Energy.

[6] Boldrini A., Koolen D., Crijns-Graus W., van den Broek M. (2024). The impact
of decarbonising the iron and steel industry on European power and
hydrogen systems. Applied Energy.

[7] Nemmour A., Inayat A., Janajreh I., Ghenai C. (2023). Green hydrogen-based
E-fuels (E-methane, E-methanol, E-ammonia) to support clean energy
transition: A literature review. International
Journal of Hydrogen Energy.

[8] Bacatelo M., Capucha F., Ferrão P., Margarido F., Bordado J. (2024). Carbon-neutral cement: The role of
green hydrogen. International Journal of Hydrogen
Energy.

[9] Bailera M., Kezibri N., Romeo L. M., Espatolero S., Lisbona P., Bouallou C. (2017). Future applications of hydrogen production
and CO_2_ utilization for energy storage: Hybrid Power to
Gas-Oxycombustion power plants. International
Journal of Hydrogen Energy.

[10] Hydrogen, International Renewable Energy Agency (IRENA). https://www.irena.org/Energy-Transition/Technology/Hydrogen#:~:text=Hydrogen%20is%20produced%20on%20a,of%20a%20mix%20of%20gases.. Accessed: 2024-07-22.

[11] Hydrogen Production Processes, Office of Energy Efficiency & Renewable Energy, U.S. Department of Energy. https://energy.sandia.gov/programs/fossil-energy/subsurface-storage/geologic-hydrogen-capabilities/geologic-hydrogen/, Accessed: 2024-07-22.

[12] Hasiuk, F. ; Conley, D. M. Exploring Geologic Hydrogen: A New Frontier for Affordable, Reliable Energy Security, Sandia National Laboratories. https://www.energy.gov/eere/fuelcells/hydrogen-production-processes, Accessed: 2025-7-12.

[13] Lamy C., Millet P. (2020). A critical review on
the definitions used to calculate
the energy efficiency coefficients of water electrolysis cells working
under near ambient temperature conditions. Journal
of Power Sources.

[14] Boettcher S. W. (2024). Introduction
to Green Hydrogen. Chemical Reviews.

[15] Brauns J., Turek T. (2020). Alkaline Water Electrolysis
Powered by Renewable Energy: A Review. Processes.

[16] Mazloomi K., Gomes C. (2012). Hydrogen as an energy
carrier: Prospects and challenges. Renewable
and Sustainable Energy Reviews.

[17] Nazir H., Muthuswamy N., Louis C., Jose S., Prakash J., Buan M. E., Flox C., Chavan S., Shi X., Kauranen P., Kallio T., Maia G., Tammeveski K., Lymperopoulos N., Carcadea E., Veziroglu E., Iranzo A., Kannan A. M. (2020). Is the H_2_ economy realizable
in the foreseeable future? Part II: H_2_ storage, transportation,
and distribution. International Journal of Hydrogen
Energy.

[18] Holladay J., Hu J., King D., Wang Y. (2009). An overview of hydrogen production
technologies. Catalysis Today.

[19] Lim D.-W., Ha J., Oruganti Y., Moon H. R. (2021). Hydrogen separation and purification
with MOF-based materials. Materials Chemistry
Frontiers.

[20] Morales-Ospino R., Celzard A., Fierro V. (2023). Strategies to recover and minimize
boil-off losses during liquid hydrogen storage. Renewable and Sustainable Energy Reviews.

[21] Yu H., Díaz A., Lu X., Sun B., Ding Y., Koyama M., He J., Zhou X., Oudriss A., Feaugas X., Zhang Z. (2024). Hydrogen Embrittlement as a Conspicuous
Material Challenge-Comprehensive Review and Future Directions. Chemical Reviews.

[22] Jena P. (2011). Materials
for Hydrogen Storage: Past, Present, and Future. Journal of Physical Chemistry Letters.

[23] Züttel A. (2003). Materials
for Hydrogen Storage. Materials Today.

[24] Chalk S. G., Miller J. F. (2006). Key challenges and
recent progress in batteries, fuel
cells, and hydrogen storage for clean energy systems. Journal of Power Sources.

[25] Haug P., Koj M., Turek T. (2017). Influence
of process conditions on gas purity in alkaline
water electrolysis. International Journal of
Hydrogen Energy.

[26] Zarghami A., Deen N., Vreman A. (2020). CFD modeling
of multiphase flow in
an alkaline water electrolyzer. Chemical Engineering
Science.

[27] Directive 2014/34/EU of the European Parliament and of the Council of 26 February 2014 on the harmonisation of the laws of the Member States relating to equipment and protective systems intended for use in potentially explosive atmospheres (recast) Text with EEA relevance. Official Journal of the European Union **2014-02-26**, *L 96/309*.

[28] Directive 2014/68/EU of the European Parliament and of the Council of 15 May 2014 on the harmonisation of the laws of the Member States relating to the making available on the market of pressure equipment (recast) Text with EEA relevance. Official Journal of the European Union **2014-05-15**, *L 189/164*.

[29] Tham M. J., Walker R. D., Gubbins K. E. (1970). Diffusion of oxygen
and hydrogen in aqueous potassium hydroxide solutions. The Journal of Physical Chemistry.

[30] Zhang C., Fan F.-R. F., Bard A. J. (2009). Electrochemistry
of oxygen in concentrated
NaOH solutions: solubility, diffusion coefficients, and superoxide
formation. Journal of the American Chemical
Society.

[31] Shi K., Meng X., Xiao S., Chen G., Wu H., Zhou C., Jiang S., Chu P. K. (2021). MXene Coatings:
Novel Hydrogen Permeation Barriers for Pipe Steels. Nanomaterials.

[32] Alanazi A., Ali M., Bawazeer S., Yekeen N., Hoteit H. (2022). Evaluation of cubic,
PC-SAFT, and GERG2008 equations of state for accurate calculations
of thermophysical properties of hydrogen-blend mixtures. Energy Reports.

[33] Alanazi A., Bawazeer S., Ali M., Keshavarz A., Hoteit H. (2022). Thermodynamic modeling of hydrogen-water systems with
gas impurity at various conditions using cubic and PC-SAFT equations
of state. Energy Conversion and Management:
X.

[34] Hassanpouryouzband A., Joonaki E., Edlmann K., Heinemann N., Yang J. (2020). Thermodynamic and transport properties
of hydrogen containing streams. Scientific Data.

[35] van
Westen T., Bauer G., Gross J. (2025). Corresponding-states
framework for classical and quantum fluids—Beyond Feynman-Hibbs. The Journal of Chemical Physics.

[36] Nasrifar K. (2010). Comparative
study of eleven equations of state in predicting the thermodynamic
properties of hydrogen. International Journal
of Hydrogen Energy.

[37] Figueroa J. D., Bayona S. G., Orozco G. A. (2025). A study
of the thermodynamic properties
of H_2_ using EOS and molecular simulation methods. International Journal of Hydrogen Energy.

[38] Leachman J. W., Jacobsen R. T., Penoncello S. G., Lemmon E. W. (2009). Fundamental Equations
of State for Parahydrogen, Normal Hydrogen, and Orthohydrogen. Journal of Physical and Chemical Reference Data.

[39] Bai-gang S., Dong-heng Z., Fu-Shui L. (2012). A new equation of state for hydrogen
gas. International Journal of Hydrogen Energy.

[40] Lozano-Martín D., Moreau A., Chamorro C. R. (2022). Thermophysical properties of hydrogen
mixtures relevant for the development of the hydrogen economy: Review
of available experimental data and thermodynamic models. Renewable Energy.

[41] Park B. H., Chae C. K. (2022). Development of correlation equations
on hydrogen properties
for hydrogen refueling process by machine learning approach. International Journal of Hydrogen Energy.

[42] Economou I. G. (2002). Statistical
Associating Fluid Theory: A Successful Model for the Calculation of
Thermodynamic and Phase Equilibrium Properties of Complex Fluid Mixtures. Industrial & Engineering Chemistry Research.

[43] Diamantonis N. I., Boulougouris G. C., Mansoor E., Tsangaris D. M., Economou I. G. (2013). Evaluation of cubic,
SAFT, and PC-SAFT equations of
state for the vapor-liquid equilibrium modeling of CO_2_ mixtures
with other gases. Ind. Eng. Chem. Res..

[44] Gross J., Sadowski G. (2001). Perturbed-Chain SAFT:
An equation of state based on
a perturbation theory for chain molecules. Ind.
Eng. Chem. Res..

[45] Li K., Ye Z., Liang X. (2025). PC-SAFT-Type Equations of State Revisited: Quantifying
the Value of Base Model Adjustments through Universal Criteria Benchmarks. Industrial & Engineering Chemistry Research.

[46] Rahbari A., Brenkman J., Hens R., Ramdin M., Van Den
Broeke L. J., Schoon R., Henkes R., Moultos O. A., Vlugt T. J. H. (2019). Solubility of water in hydrogen at high pressures:
a molecular simulation study. Journal of Chemical
& Engineering Data.

[47] Rahbari A., Garcia-Navarro J. C., Ramdin M., van den
Broeke L. J. P., Moultos O. A., Dubbeldam D., Vlugt T. J. H. (2021). Effect of Water
Content on Thermodynamic Properties of Compressed Hydrogen. J. Chem. Eng. Data.

[48] Köster A., Thol M., Vrabec J. (2018). Molecular models for
the hydrogen
age: hydrogen, nitrogen, oxygen, argon, and water. J. Chem. Eng. Data.

[49] Sesé L. M. (1994). Study of
the Feynman-Hibbs effective potential against the path-integral formalism
for Monte Carlo simulations of quantum many-body Lennard-Jones systems. Mol. Phys..

[50] Chen J., Li X.-Z., Zhang Q., Probert M. I., Pickard C. J., Needs R. J., Michaelides A., Wang E. (2013). Quantum simulation
of low-temperature metallic liquid hydrogen. Nature communications.

[51] Frenkel, D. ; Smit, B. Understanding Molecular Simulation: From Algorithms to Applications, 3rd ed.; Elsevier: San Diego, United States, 2023.

[52] Allen, M. P. ; Tildesley, D. J. Computer Simulation of Liquids, 2nd ed.; Oxford University Press: Oxford, 2017.

[53] McQuarrie, D. A. ; Simon, J. D. Physical Chemistry: A Molecular Approach; University Science Books: Sausalito, California, 1997.

[54] Vega C., Sanz E., Abascal J. L. F., Noya E. G. (2008). Determination of
phase diagrams via computer simulation: methodology and applications
to water, electrolytes and proteins. J. Phys.:
Condens. Matt..

[55] Allen M. P. (2000). Long-range
behaviour in liquid crystals by computer simulation. J. Mol. Liq..

[56] Chew P. Y., Reinhardt A. (2023). Phase diagrams—Why they matter
and how to predict
them. J. Chem. Phys..

[57] Reinhardt A., Cheng B. (2021). Quantum-mechanical exploration of the phase diagram of water. Nature Communications.

[58] Reinhardt A., Bethkenhagen M., Coppari F., Millot M., Hamel S., Cheng B. (2022). Thermodynamics of high-pressure ice phases explored with atomistic
simulations. Nature Communications.

[59] Marrink S. J., Risselada H. J., Yefimov S., Tieleman D. P., de Vries A. H. (2007). The MARTINI
Force Field: Coarse Grained Model for Biomolecular Simulations. The Journal of Physical Chemistry B.

[60] Souza P. C. T., Alessandri R., Barnoud J., Thallmair S., Faustino I., Grunewald F., Patmanidis I., Abdizadeh H., Bruininks B. M. H., Wassenaar T. A., Kroon P. C., Melcr J., Nieto V., Corradi V., Khan H. M., Domański J., Javanainen M., Martinez-Seara H., Reuter N., Best R. B., Vattulainen I., Monticelli L., Periole X., Tieleman D. P., de Vries A. H., Marrink S. J. (2021). Martini 3: a general purpose force
field for coarse-grained
molecular dynamics. Nature Methods.

[61] Hoogerbrugge P. J., Koelman J. M. V. A. (1992). Simulating
Microscopic Hydrodynamic Phenomena with
Dissipative Particle Dynamics. Europhysics Letters.

[62] Jenko F. (2025). Accelerating
fusion research via supercomputing. Nature Reviews
Physics.

[63] Lu G.-H., Zhou H.-B., Becquart C. S. (2014). A review
of modelling and simulation
of hydrogen behaviour in tungsten at different scales. Nuclear Fusion.

[64] Wang X.-Y., Wang Y.-N., Xu K., Dai F.-Z., Liu H.-F., Lu G.-H., Wang H. (2023). Deep neural network potential for
simulating hydrogen blistering in tungsten. Physical Review Materials.

[65] Li X.-C., Li Y.-W., Huang H.-X., Li Y.-H., Zhou H.-B., Lu G.-H., Deng H., Zhou H.-S., Luo G.-N. (2025). Analytical
WH, HH and HHe interatomic potentials for a WH-He system. Journal of Nuclear Materials.

[66] Brenner D. W. (1990). Empirical
potential for hydrocarbons for use in simulating the chemical vapor
deposition of diamond films. Physical review
B.

[67] Li X.-C., Shu X., Liu Y.-N., Gao F., Lu G.-H. (2011). Modified analytical
interatomic potential for a W-H system with defects. Journal of Nuclear Materials.

[68] Thompson A. P., Swiler L. P., Trott C. R., Foiles S. M., Tucker G. J. (2015). Spectral
neighbor analysis method for automated generation of quantum-accurate
interatomic potentials. Journal of Computational
Physics.

[69] El-Adawy M., Dalha I. B., Ismael M. A., Al-Absi Z. A., Nemitallah M. A. (2024). Review
of Sustainable Hydrogen Energy Processes: Production, Storage, Transportation,
and Color-Coded Classifications. Energy &
Fuels.

[70] Incer-Valverde J., Korayem A., Tsatsaronis G., Morosuk T. (2023). “Colors”
of hydrogen: Definitions and carbon intensity. Energy Conversion and Management.

[71] Manabe A., Kashiwase M., Hashimoto T., Hayashida T., Kato A., Hirao K., Shimomura I., Nagashima I. (2013). Basic study of alkaline water electrolysis. Electrochimica Acta.

[72] Haug P., Kreitz B., Koj M., Turek T. (2017). Process modelling of
an alkaline water electrolyzer. Int. J. Hydrogen
Energy.

[73] de
Groot M. T., Kraakman J., Garcia Barros R. L. (2022). Optimal
operating parameters for advanced alkaline water electrolysis. Int. J. Hydrogen Energy.

[74] Oikonomidis S., Ramdin M., Moultos O. A., Bos A., Vlugt T. J. H., Rahbari A. (2023). Transient modelling of a multi-cell
alkaline electrolyzer
for gas crossover and safe system operation. Int. J. Hydrogen Energy.

[75] Abdin Z., Webb C., Gray E. (2017). Modelling
and simulation of an alkaline
electrolyser cell. Energy.

[76] Hammoudi M., Henao C., Agbossou K., Dubé Y., Doumbia M. (2012). New multi-physics approach for modelling
and design
of alkaline electrolyzers. International Journal
of Hydrogen Energy.

[77] Bouwman P. (2014). Electrochemical
Hydrogen Compression (EHC) solutions for hydrogen infrastructure. Fuel Cells Bulletin.

[78] Bampaou M., Panopoulos K. D., Papadopoulos A. I., Seferlis P., Voutetakis S. (2018). An electrochemical
hydrogen compression model. Chem. Eng. Trans..

[79] de
Miguel N., Acosta B., Moretto P., Ortiz Cebolla R. (2016). Influence
of the gas injector configuration on the temperature evolution during
refueling of on-board hydrogen tanks. Int. J.
Hydrogen Energy.

[80] NASA , Fueling Protocols for Light Duty Gaseous Hydrogen Surface Vehicles. https://www.sae.org/standards/content/j2601_202005/, 2020; Accessed: 01-12-2020.

[81] Ji M., Wei Z. (2009). A review of
water management in polymer electrolyte membrane fuel
cells. Energies.

[82] Chatenet M., Pollet B. G., Dekel D. R., Dionigi F., Deseure J., Millet P., Braatz R. D., Bazant M. Z., Eikerling M., Staffell I., Balcombe P., Shao-Horn Y., Schäfer H. (2022). Water electrolysis: from textbook
knowledge to the
latest scientific strategies and industrial developments. Chem. Soc. Rev..

[83] Haug P., Koj M., Turek T. (2017). Influence of process conditions on gas purity in alkaline
water electrolysis. Int. J. Hydrogen Energy.

[84] Haug P., Kreitz B., Koj M., Turek T. (2017). Process modelling of
an alkaline water electrolyzer. Int. J. Hydrogen
Energy.

[85] Bodner M., Hofer A., Hacker V. (2015). H_2_ generation from alkaline
electrolyzer. Wiley Interdisciplinary Reviews:
Energy and Environment.

[86] David M., Ocampo-Martínez C., Sánchez-Peña R. (2019). Advances in
alkaline water electrolyzers: A review. Journal
of Energy Storage.

[87] Solovey V., Shevchenko A., Zipunnikov M., Kotenko A., Khiem N. T., Tri B. D., Hai T. T. (2022). Development
of high pressure membraneless
alkaline electrolyzer. International Journal
of Hydrogen Energy.

[88] Ulleberg Ø. (2003). Modeling
of advanced alkaline electrolyzers: a system simulation approach. International Journal of Hydrogen Energy.

[89] Merle G., Wessling M., Nijmeijer K. (2011). Anion exchange
membranes for alkaline
fuel cells: A review. Journal of Membrane Science.

[90] Haug P., Kreitz B., Koj M., Turek T. (2017). Process modelling of
an alkaline water electrolyzer. International
Journal of Hydrogen Energy.

[91] Himmelblau D. M. (1964). Diffusion
of Dissolved Gases in Liquids. Chemical Reviews.

[92] Kusoglu A., Weber A. Z. (2017). New Insights into
Perfluorinated Sulfonic-Acid Ionomers. Chem.
Rev..

[93] Arntsen C., Savage J., Tse Y.-L., Voth G. A. (2016). Simulation of proton
transport in proton exchange membranes with reactive molecular dynamics. Fuel Cells.

[94] Rahbari A., Hartkamp R., Moultos O. A., Bos A., van den
Broeke L. J. P., Ramdin M., Dubbeldam D., Lyulin A. V., Vlugt T. J. H. (2022). Electro-osmotic Drag and Thermodynamic
Properties of Water in Hydrated Nafion Membranes from Molecular Dynamics. J. Phys. Chem. C.

[95] Zhang G., Yang G., Shen Q., Li S., Li Z., Liao J., Jiang Z., Wang H., Zhang H., Ye W. (2022). Study on the transport performance degradation of Nafion membrane
due to the presence of Na^+^ and Ca^2+^ using molecular
dynamics simulations. Journal of Power Sources.

[96] Li H., You J., Cheng X., Yan X., Shen S., Zhang J. (2023). New insight
into the effect of Co^2+^ contamination on local oxygen transport
in PEMFCs. Chemical Engineering Journal.

[97] Zhu Z., Paddison S. J. (2022). Perspective: Morphology
and ion transport in ion-containing
polymers from multiscale modeling and simulations. Frontiers in Chemistry.

[98] Wang Y., Si C., Zhang X., Wang X., He W. (2022). Electro-osmotic drag
coefficient of Nafion membrane with low water Content for Proton exchange
membrane fuel cells. Energy Reports.

[99] Sengupta S., Lyulin A. V. (2019). Molecular Modeling
of Structure and Dynamics of Nafion
Protonation States. J. Phys. Chem. B.

[100] Lyulin A. V., Sengupta S., Varughese A., Komarov P., Venkatnathan A. (2020). Effect of Annealing on Structure
and Diffusion in Hydrated Nafion Membranes. ACS Appl. Polym. Mater..

[101] Ozmaian M., Naghdabadi R. (2014). Molecular dynamics simulation study
of glass transition in hydrated Nafion. Journal
of Polymer Science Part B: Polymer Physics.

[102] Tuckerman M. E., Laasonen K., Sprik M., Parrinello M. (1994). Ab initio
simulations of water and water ions. Journal
of Physics: Condensed Matter.

[103] Tuckerman M. E., Marx D., Parrinello M. (2002). The nature
and transport mechanism of hydrated hydroxide ions in aqueous solution. Nature.

[104] Marx D. (2006). Proton Transfer 200 Years after von Grotthuss: Insights from Ab Initio
Simulations. ChemPhysChem.

[105] Lagerweij V. J., Bougueroua S., Habibi P., Dey P., Gaigeot M.-P., Moultos O. A., Vlugt T. J. H. (2025). From Grotthuss
Transfer to Conductivity: Machine Learning Molecular Dynamics of Aqueous
KOH. The Journal of Physical Chemistry B.

[106] Dohrn R., Brunner G. (1995). High-pressure fluid-phase
equilibria:
Experimental methods and systems investigated (1988-1993). Fluid Phase Equilib..

[107] Christov M., Dohrn R. (2002). High-pressure fluid phase equilibria:
Experimental methods and systems investigated (1994-1999). Fluid Phase Equilib..

[108] Dohrn R., Peper S., Fonseca J. M. (2010). High-pressure fluid-phase
equilibria: Experimental methods and systems investigated (2000-2004). Fluid Phase Equilib..

[109] Fonseca J. M., Dohrn R., Peper S. (2011). High-pressure fluid-phase
equilibria: Experimental methods and systems investigated (2005-2008). Fluid Phase Equilib..

[110] Rahbari A., Brenkman J., Hens R., Ramdin M., Broeke L. J. V. D., Schoon R., Henkes R., Moultos O. A., Vlugt T. J. H. (2019). Solubility of water in hydrogen at high pressures:
A Molecular Simulation study. J. Chem. Eng.
Data.

[111] Habibi P., Dey P., Vlugt T. J. H., Moultos O. A. (2024). Effect
of Dissolved KOH and NaCl on the solubilities of water in Hydroen:
A Monte Carlo Study. The Journal of Chemical
Physics.

[112] Habibi P., Rahbari A., Blazquez S., Vega C., Dey P., Vlugt T. J. H., Moultos O. A. (2022). A New Force
Field for OH- for Computing
Thermodynamic and Transport Properties of H_2_ and O_2_ in Aqueous NaOH and KOH Solutions. J. Phys. Chem. B.

[113] Habibi P., Postma J. R. T., Padding J. T., Dey P., Vlugt T. J. H., Moultos O. A. (2023). Thermodynamic and Transport Properties
of H_2_/H_2_O/NaB­(OH)_4_ Mixtures Using
the Delft Force Field (DFF/B­(OH)_4_
^‑^). Industrial & Engineering Chemistry Research.

[114] Kwon S. H., Kang H., Sohn Y.-J., Lee J., Shim S., Lee S. G. (2021). Molecular dynamics simulation study
on the effect of perfluorosulfonic acid side chains on oxygen permeation
in hydrated ionomers of PEMFCs. Scientific Reports.

[115] Awulachew S., Nigussa K. (2023). Molecular dynamics
simulation of
ion dynamics within PEM Fuel Cells. Chemical
Physics Letters.

[116] Cui S., Liu J., Selvan M. E., Keffer D. J., Edwards B. J., Steele W. V. (2007). A Molecular
Dynamics Study of a Nafion Polyelectrolyte
Membrane and the Aqueous Phase Structure for Proton Transport. J. Phys. Chem. B.

[117] Venkatnathan A., Devanathan R., Dupuis M. (2007). Atomistic Simulations
of Hydrated Nafion and Temperature Effects on Hydronium Ion Mobility. J. Phys. Chem. B.

[118] Hofmann D. W., Kuleshova L., D’Aguanno B. (2008). Molecular
dynamics simulation of hydrated Nafion with a reactive force field
for water. Journal of Molecular Modeling.

[119] Hofmann D., Kuleshova L., D’Aguanno B. (2010). Theoretical
simulations of proton conductivity: Basic principles for improving
the proton conductor. Journal of Power Sources.

[120] Brunello, G. F. ; Mateker, W. R. ; Lee, S. G. ; Choi, J. I. ; Jang, S. S. Effect of temperature on structure and water transport of hydrated sulfonated poly (ether ether ketone): A molecular dynamics simulation approach. Journal of Renewable and Sustainable Energy 2011, 3.043111 10.1063/1.3608912

[121] Brunello G., Lee S. G., Jang S. S., Qi Y. (2009). A molecular
dynamics simulation study of hydrated sulfonated poly (ether ether
ketone) for application to polymer electrolyte membrane fuel cells:
Effect of water content. Journal of Renewable
and Sustainable Energy.

[122] Lins R. D., Devanathan R., Dupuis M. (2011). Modeling the nanophase
structural dynamics of phenylated sulfonated poly ether ether ketone
ketone (Ph-SPEEKK) membranes as a function of hydration. J. Phys. Chem. B.

[123] Devanathan R., Idupulapati N., Dupuis M. (2012). Molecular modeling
of the morphology and transport properties of two direct methanol
fuel cell membranes: Phenylated sulfonated poly (ether ether ketone
ketone) versus Nafion. Journal of Materials
Research.

[124] Cha J. (2020). Morphological
effect of side chain on H_3_O^+^ transfer
inside polymer electrolyte membranes across polymeric chain via molecular
dynamics simulation. Scientific Reports.

[125] Flottat T., Latour B., Goujon F., Hauret P., Malfreyt P. (2023). Investigating percolation and clustering
effects on
aquivion and nafion membranes at the molecular scale. Int. J. Hydrogen Energy.

[126] Xian L., Li Z., Li S., Chen L., Tao W.-Q. (2023). Elucidating the impact mechanism
of temperature and
water content on thermal conductivity of hydrated Nafion membranes
by molecular dynamics simulation. International
Journal of Heat and Mass Transfer.

[127] Din X.-D., Michaelides E. E. (1998). Transport
processes of water and
protons through micropores. AIChE Journal.

[128] Yan L., Ji X., Lu W. (2008). Molecular
Dynamics Simulations of
Electroosmosis in Perfluorosulfonic Acid Polymer. J. Phys. Chem. B.

[129] Choe Y.-K., Tsuchida E., Ikeshoji T., Yamakawa S., Hyodo S.-a. (2008). Nature
of Water Transport and Electro-Osmosis in Nafion:
Insights from First-Principles Molecular Dynamics Simulations under
an Electric Field. J. Phys. Chem. B.

[130] Keskin S., Sholl D. S. (2009). Assessment of a
Metal-Organic Framework
Membrane for Gas Separations Using Atomically Detailed Calculations:
CO_2_, CH_4_, N_2_, H_2_ Mixtures
in MOF-5. Industrial & Engineering Chemistry
Research.

[131] Bayati B., Ghorbani A., Ghasemzadeh K., Iulianelli A., Basile A. (2019). Study on the Separation of H_2_ from CO_2_ Using a ZIF-8 Membrane by Molecular Simulation
and Maxwell-Stefan Model. Molecules.

[132] Duan S., Li D., Yang X., Niu C., Sun S., He X., Shan M., Zhang Y. (2023). Experimental
and molecular
simulation study of a novel benzimidazole-linked polymer membrane
for efficient H_2_/CO_2_ separation. Journal of Membrane Science.

[133] Hiscock, K. M. ; Bense, V. F. Hydrogeology: Principles and Practice, 3rd Edition; Wiley-Blackwell, 2021.

[134] Barrer R. M., Chio H. T. (1965). Solution and diffusion of gases and
vapors in silicone rubber membranes. Journal
of Polymer Science Part C: Polymer Symposia.

[135] Karki S., Hazarika G., Yadav D., Ingole P. G. (2024). Polymeric
membranes for industrial applications: Recent progress, challenges
and perspectives. Desalination.

[136] Robeson L. M. (2008). The upper bound revisited. Journal
of Membrane Science.

[137] Koros W. J., Zhang C. (2017). Materials for next-generation molecularly
selective synthetic membranes. Nature Materials.

[138] Krishna R. (2018). Methodologies for screening and selection
of crystalline
microporous materials in mixture separations. Separation and Purification Technology.

[139] Adil K., Belmabkhout Y., Pillai R. S., Cadiau A., Bhatt P. M., Assen A. H., Maurin G., Eddaoudi M. (2017). Gas/vapour
separation using ultra-microporous metal-organic frameworks: insights
into the structure/separation relationship. Chem. Soc. Rev..

[140] Perez-Carbajo J., Matito-Martos I., Balestra S. R. G., Tsampas M. N., van de
Sanden M. C. M., Delgado J. A., Águeda V. I., Merkling P. J., Calero S. (2018). Zeolites for CO_2_-CO-O_2_ Separation to Obtain CO_2_-Neutral Fuels. ACS Applied Materials & Interfaces.

[141] Ackley M. W., Rege S. U., Saxena H. (2003). Application
of natural
zeolites in the purification and separation of gases. Microporous and Mesoporous Materials.

[142] Sircar S., Golden T., Rao M. (1996). Activated
carbon for
gas separation and storage. Carbon.

[143] Erdos M., Geerdink D. F., Martin-Calvo A., Pidko E. A., van den Broeke L. J.
P., Calero S., Vlugt T. J. H., Moultos O. A. (2021). In Silico Screening of Zeolites for
High-Pressure Hydrogen Drying. ACS Applied Materials
& Interfaces.

[144] Jeong H.-K. (2021). Metal-organic framework membranes: Unprecedented opportunities
for gas separations. AIChE Journal.

[145] Beuerle F., Gole B. (2018). Covalent Organic Frameworks
and Cage
Compounds: Design and Applications of Polymeric and Discrete Organic
Scaffolds. Angewandte Chemie International Edition.

[146] Yang Z., Wu Z., Peh S. B., Ying Y., Yang H., Zhao D. (2023). Mixed-Matrix Membranes
Containing
Porous Materials for Gas Separation: From Metal-Organic Frameworks
to Discrete Molecular Cages. Engineering.

[147] Li W. (2019). Metal-organic framework membranes:
Production, modification, and
applications. Progress in Materials Science.

[148] Ahmad N. N. R., Lee Y., Abdul Hamid M. R., Mohd Ghazi T. I., Nasir R., Leo C. P., Koh S. P., Pasupuleti J., Tiong S. K. (2023). Unlocking the potential of metal-organic
frameworks-based mixed matrix membranes for hydrogen separation and
purification. Journal of Industrial and Engineering
Chemistry.

[149] Aksu G. O., Erucar I., Haslak Z. P., Keskin S. (2022). Accelerating
discovery of COFs for CO_2_ capture and H_2_ purification
using structurally guided computational screening. Chemical Engineering Journal.

[150] Ignacz G., Bader L., Beke A. K., Ghunaim Y., Shastry T., Vovusha H., Carbone M. R., Ghanem B., Szekely G. (2025). Machine learning for the advancement of membrane science
and technology: A critical review. Journal of
Membrane Science.

[151] Chen Y., Zhao G., Yoon S., Habibi P., Hong C. S., Li S., Moultos O. A., Dey P., Vlugt T. J. H., Chung Y. G. (2024). Computational
Exploration of Adsorption-Based
Hydrogen Storage in Mg-Alkoxide Functionalized Covalent-Organic Frameworks
(COFs): Force-Field and Machine Learning Models. ACS Applied Materials & Interfaces.

[152] Chung Y. G., Camp J., Haranczyk M., Sikora B. J., Bury W., Krungleviciute V., Yildirim T., Farha O. K., Sholl D. S., Snurr R. Q. (2014). Computation-Ready,
Experimental Metal-Organic Frameworks: A Tool To Enable High-Throughput
Screening of Nanoporous Crystals. Chemistry
of Materials.

[153] Aksu G. O., Keskin S. (2023). Advancing CH_4_/H_2_ separation with covalent organic frameworks by combining
molecular
simulations and machine learning. J. Mater.
Chem. A.

[154] Daglar H., Keskin S. (2022). Combining Machine Learning and Molecular
Simulations to Unlock Gas Separation Potentials of MOF Membranes and
MOF/Polymer MMMs. ACS Applied Materials &
Interfaces.

[155] Aksu G. O., Keskin S. (2025). The COF Space: Materials Features,
Gas Adsorption, and Separation Performances Assessed by Machine Learning. ACS Materials Letters.

[156] Altintas C., Keskin S. (2023). On the shoulders of
high-throughput
computational screening and machine learning: Design and discovery
of MOFs for H_2_ storage and purification. Materials Today Energy.

[157] Demir H., Daglar H., Gulbalkan H. C., Aksu G. O., Keskin S. (2023). Recent advances
in computational
modeling of MOFs: From molecular simulations to machine learning. Coordination Chemistry Reviews.

[158] Altintas C., Altundal O. F., Keskin S., Yildirim R. (2021). Machine Learning
Meets with Metal Organic Frameworks for Gas Storage and Separation. Journal of Chemical Information and Modeling.

[159] Park Y., Kang J.-H., Moon D.-K., Jo Y. S., Lee C.-H. (2021). Parallel and series multi-bed pressure
swing adsorption
processes for H2 recovery from a lean hydrogen mixture. Chemical Engineering Journal.

[160] Ribeiro A. M., Grande C. A., Lopes F. V., Loureiro J. M., Rodrigues A. E. (2008). A parametric study of layered bed PSA for hydrogen
purification. Chemical Engineering Science.

[161] Sloan, E. D. J. ; Koh, C. A. Clathrate Hydrates of Natural Gases, 3rd ed.; Taylor & Francis Group: Boca Raton, FL, 2008.

[162] Sloan E. D. (2003). Fundamental principles and applications
of natural gas hydrates. Nature.

[163] Khokhar A., Gudmundsson J., Sloan E. (1998). Gas storage in structure
H hydrates. Fluid Phase Equilibria.

[164] Thomas S., Dawe R. A. (2003). Review of ways to
transport natural
gas energy from countries which do not need the gas for domestic use. Energy.

[165] Mi F., He Z., Pang J., Moultos O. A., Vlugt T. J. H., Ning F. (2024). Molecular Insights into Hybrid CH_4_ Physisorption-Hydrate
Formation in Spiral Halloysite Nanotubes: Implications for Energy
Storage. ACS Applied Materials & Interfaces.

[166] Fang B., Lü T., Li W., Moultos O. A., Vlugt T. J. H., Ning F. (2024). Microscopic insights
into poly- and
mono-crystalline methane hydrate dissociation in Na-montmorillonite
pores at static and dynamic fluid conditions. Energy.

[167] Brewer P. G., Friederich G., Peltzer E. T., Orr F. M. (1999). Direct experiments
on the ocean disposal of fossil
fuel CO_2_. Science.

[168] Mi F., Li W., Pang J., Moultos O. A., Ning F., Vlugt T. J. H. (2024). Molecular Insights
into the Microscopic Behavior of
CO_2_ Hydrates in Oceanic Sediments: Implications for Carbon
Sequestration. The Journal of Physical Chemistry
C.

[169] Ngan Y. T., Englezos P. (1996). Concentration of mechanical pulp
mill effluents and NaCl solutions through propane hydrate formation. Industrial & Engineering Chemistry Research.

[170] Babu P., Nambiar A., He T., Karimi I. A., Lee J. D., Englezos P., Linga P. (2018). A review of
clathrate
hydrate based desalination to strengthen energy-water nexus. ACS Sustainable Chemistry & Engineering.

[171] Mao W. L., Mao H.-k., Goncharov A. F., Struzhkin V. V., Guo Q., Hu J., Shu J., Hemley R. J., Somayazulu M., Zhao Y. (2002). Hydrogen clusters in
clathrate hydrate. Science.

[172] Veluswamy H. P., Kumar R., Linga P. (2014). Hydrogen storage
in
clathrate hydrates: Current state of the art and future directions. Applied Energy.

[173] Babu P., Linga P., Kumar R., Englezos P. (2015). A review of
the Hydrate Based Gas Separation (HBGS) process for carbon dioxide
pre-combustion capture. Energy.

[174] Kastanidis P., Tsimpanogiannis I. N., Romanos G. E., Stubos A. K., Economou I. G. (2019). Recent advances
in experimental measurements of mixed-gas
three-phase hydrate equilibria for gas mixture separation and energy-related
applications. Journal of Chemical & Engineering
Data.

[175] Tsimpanogiannis I. N., Costandy J., Kastanidis P., El Meragawi S., Michalis V. K., Papadimitriou N. I., Karozis S. N., Diamantonis N. I., Moultos O. A., Romanos G. E. (2018). Using clathrate
hydrates for gas storage and gas-mixture separations: Experimental
and computational studies at multiple length scales. Molecular Physics.

[176] Hassanpouryouzband A., Joonaki E., Farahani M. V., Takeya S., Ruppel C., Yang J., English N. J., Schicks J. M., Edlmann K., Mehrabian H., Aman Z. M., Tohidi B. (2020). Gas hydrates
in sustainable chemistry. Chemical Society Reviews.

[177] Alavi S., Ripmeester J. A. (2017). Simulations
of hydrogen gas in clathrate
hydrates. Molecular Simulation.

[178] Patchkovskii S., Tse J. S. (2003). Thermodynamic stability
of hydrogen
clathrates. Proceedings of the National Academy
of Sciences.

[179] Tribello G. A., Slater B. (2009). A theoretical examination of known
and hypothetical clathrate hydrate materials. The Journal of Chemical Physics.

[180] Wang J., Lu H., Ripmeester J. A., Becker U. (2010). Molecular-dynamics
and first-principles calculations
of raman spectra and molecular and electronic structure of hydrogen
clusters in hydrogen clathrate hydrate. The
Journal of Physical Chemistry C.

[181] Willow S. Y., Xantheas S. S. (2012). Enhancement of hydrogen storage capacity
in hydrate lattices. Chemical Physics Letters.

[182] Trinh T. T., Waage M. H., Van Erp T. S., Kjelstrup S. (2015). Low barriers
for hydrogen diffusion in sII clathrate. Physical
Chemistry Chemical Physics.

[183] van
der Waals J., Platteeuw J. (1958). Clathrate solutions. Advances in Chemical Physics.

[184] Parrish W. R., Prausnitz J. M. (1972). Dissociation
pressures of gas hydrates
formed by gas mixtures. Industrial & Engineering
Chemistry Process Design and Development.

[185] Holder G., Zetts S., Pradhan N. (1988). Phase behavior
in systems
containing clathrate hydrates: a review. Reviews
in Chemical Engineering.

[186] Ballard A., Sloan E. (2002). The next generation of
hydrate prediction: I. Hydrate standard states and incorporation of
spectroscopy. Fluid Phase Equilibria.

[187] Medeiros F. D. A., Segtovich I. S. V., Tavares F. W., Sum A. K. (2020). Sixty years
of the van der Waals and Platteeuw model for clathrate hydrates—A
critical review from its statistical thermodynamic basis to its extensions
and applications. Chemical Reviews.

[188] Baltus R. E., Counce R. M., Culbertson B. H., Luo H., DePaoli D. W., Dai S., and D. C. D. (2005). Examination of
the Potential of Ionic Liquids for Gas Separations. Separation Science and Technology.

[189] Ramdin M., de Loos T. W., Vlugt T. J. H. (2012). State-of-the-Art
of CO_2_ Capture with Ionic Liquids. Industrial & Engineering Chemistry Research.

[190] Designer Solvents. Chemical & Engineering News Archive 1998, 76, 32–37.

[191] Han X., Armstrong D. W. (2007). Ionic Liquids in Separations. Accounts of Chemical Research.

[192] U.S. Department of Energy , Hydrogen and Fuel Cell Technologies Office. https://www.energy.gov/eere/fuelcells/hydrogen-storage, 2023; Accessed: 03-01-2024.

[193] U.S. Department of Energy , Hydrogen Storage Fact Sheet. https://www.energy.gov/eere/fuelcells/articles/hydrogen-storage-fact-sheet, 2017; Accessed: 03-01-2024.

[194] Bell I. H., Wronski J., Quoilin S., Lemort V. (2014). Pure and Pseudo-pure
Fluid Thermophysical Property Evaluation and the Open-Source Thermophysical
Property Library CoolProp. Industrial &
Engineering Chemistry Research.

[195] Pizzutilo E., Acher T., Reuter B., Will C., Schäfer S. (2024). Subcooled
liquid hydrogen technology for heavy-duty
trucks. World Electric Vehicle Journal.

[196] Safe, Fast and Simple: Daimler Truck and Linde Set New Standard for Liquid Hydrogen Refueling Technolog. https://www.daimlertruck.com/en/newsroom/pressrelease/safe-fast-and-simple-daimler-truck-and-linde-set-new-standard-for-liquid-hydrogen-refueling-technology-52581266#:~:text=Daimler%20Truck%20and%20Linde%20Engineering,costs%20and%20superior%20energy%20efficiency.. Accessed: October 15, 2025.

[197] DOE Technical Targets for Onboard Hydrogen Storage for Light-Duty Vehicles. https://www.energy.gov/eere/fuelcells/doe-technical-targets-onboard-hydrogen-storage-light-duty-vehicles, Accessed: October 15, 2025.

[198] Ahmed A., Seth S., Purewal J., Wong-Foy A. G., Veenstra M., Matzger A. J., Siegel D. J. (2019). Exceptional hydrogen
storage achieved by screening nearly half a million metal-organic
frameworks. Nature communications.

[199] Durbin D., Malardier-Jugroot C. (2013). Review of
Hydrogen Storage Techniques
for on Board Vehicle Applications. International
Journal of Hydrogen Energy.

[200] Eberle U., Felderhoff M., Schueth F. (2009). Chemical and Physical
Solutions for Hydrogen Storage. Angewandte Chemie
International Edition.

[201] Sdanghi G., Maranzana G., Celzard A., Fierro V. (2019). Review of
the current technologies and performances of hydrogen compression
for stationary and automotive applications. Renew. Sust. Energy Rev..

[202] Abe J., Popoola A., Ajenifuja E., Popoola O. (2019). Hydrogen energy, economy
and storage: Review and recommendation. International
Journal of Hydrogen Energy.

[203] Perspective on hydrogen energy carrier and its automotive applications. International Journal of Hydrogen Energy 2014 39, 8482–8494.

[204] Møller K. T., Jensen T. R., Akiba E., wen Li H. (2017). Hydrogen -
A sustainable energy carrier. Progress in Natural
Science: Materials International.

[205] Staffell I., Scamman D., Abad A. V., Balcombe P., Dodds P. E., Ekins P., Shah N., Ward K. R. (2019). The role
of hydrogen and fuel cells in the global energy system. Energy & Environmental Science.

[206] Schlapbach L., Züttel A. (2001). Hydrogen-storage
materials for mobile
applications. Nature.

[207] Kuroki T., Sakoda N., Shinzato K., Monde M., Takata Y. (2018). Prediction of transient temperature
of hydrogen flowing
from pre-cooler of refueling station to inlet of vehicle tank. Int. J. Hydrogen Energy.

[208] Najdi, R. A. ; Shaban, T. G. ; Mourad, M. J. ; Karaki, S. H. Hydrogen production and filling of fuel cell cars. 2016 3rd International Conference on Advances in Computational Tools for Engineering Applications (ACTEA). 2016; pp 43-48.

[209] de Miguel N., Acosta B., Baraldi D., Melideo R., Ortiz Cebolla R., Moretto P. (2016). The role of initial tank temperature
on refuelling of on-board hydrogen tanks. Int.
J. Hydrogen Energy.

[210] Adolf, J. ; Balzer, C. ; Louis, J. Energy of the future? Sustainable mobility through fuel cells and H_2_; Shell Hydrogen Study 2017; DOI: Technical Report 10.13140/RG.2.2.31848.57604.

[211] Manoharan Y., Hosseini S. E., Butler B., Alzhahrani H., Senior B. T. F., Ashuri T., Krohn J. (2019). Hydrogen Fuel
Cell
Vehicles; Current Status and Future Prospect. Applied Sciences.

[212] Hammerschlag R., Mazza P. (2005). Questioning hydrogen. Energy Policy.

[213] Das L. (1996). On-board hydrogen storage systems for automotive application. Int. J. Hydrogen Energy.

[214] Bauer A., Mayer T., Semmel M., Guerrero
Morales M. A., Wind J. (2019). Energetic evaluation of hydrogen
refueling stations with liquid or gaseous stored hydrogen. Int. J. Hydrogen Energy.

[215] Rivard E., Trudeau M., Zaghib K. (2019). Hydrogen Storage
for
Mobility: A Review. Materials.

[216] Moradi R., Groth K. M. (2019). Hydrogen storage
and delivery: Review
of the state of the art technologies and risk and reliability analysis. Int. J. Hydrogen Energy.

[217] Smolinka, T. ; Garche, J. Electrochemical power sources: fundamentals, systems, and applications: hydrogen production by water electrolysis; Elsevier: Radarweg 29, PO Box 211, 1000 AE Amsterdam, The Netherlands, 2021.

[218] Fang Q., Ji D. (2023). Molecular simulation of hydrogen
permeation behavior in liner polymer materials of Type hydrogen storage
vessels. Materials Today Communications.

[219] Preuster P., Papp C., Wasserscheid P. (2017). Liquid Organic
Hydrogen Carriers (LOHCs): Toward a Hydrogen-free Hydrogen Economy. Accounts of Chemical Research.

[220] Ghotia S., Kumar P., Srivastava A. K. (2025). A review
on 2D materials: unveiling next-generation hydrogen storage solutions,
advancements and prospects. Journal of Materials
Science.

[221] Sunnardianto G. K., Bokas G., Hussein A., Walters C., Moultos O. A., Dey P. (2021). Efficient Hydrogen Storage in Defective
Graphene and its Mechanical Stability: A Combined Density Functional
Theory and Molecular Dynamics Simulation Study. International Journal of Hydrogen Energy.

[222] Habibi P., Vlugt T. J. H., Dey P., Moultos O. A. (2021). Reversible
Hydrogen Storage in Metal-Decorated Honeycomb Borophene Oxide. ACS Applied Materials & Interfaces.

[223] Bhatia S. K., Myers A. L. (2006). Optimum Conditions
for Adsorptive
Storage. Langmuir.

[224] Chan K. S., Miller M. A., Peng X. (2018). First-Principles
Computational
Study of Hydrogen Storage in Silicon Clathrates. Materials Research Letters.

[225] Lee H., Choi W. I., Nguyen M. C., Cha M.-H., Moon E., Ihm J. (2007). Ab Initio Study of Dihydrogen Binding
in Metal-Decorated Polyacetylene
for Hydrogen Storage. Phys. Rev. B.

[226] Wang L., Chen X., Du H., Yuan Y., Qu H., Zou M. (2018). First-Principles Investigation
on Hydrogen Storage
Performance of Li, Na and K Decorated Borophene. Applied Surface Science.

[227] Suh M. P., Park H. J., Prasad T. K., Lim D.-W. (2012). Hydrogen
Storage in Metal-Organic Frameworks. Chemical
Reviews.

[228] Zhang X., Liu P., Zhang Y. (2023). The application
of
MOFs for hydrogen storage. Inorganica Chimica
Acta.

[229] Klopčič N., Grimmer I., Winkler F., Sartory M., Trattner A. (2023). A review on metal hydride
materials
for hydrogen storage. Journal of Energy Storage.

[230] Schneemann A., White J. L., Kang S., Jeong S., Wan L. F., Cho E. S., Heo T. W., Prendergast D., Urban J. J., Wood B. C., Allendorf M. D., Stavila V. (2018). Nanostructured Metal Hydrides for Hydrogen Storage. Chemical Reviews.

[231] Lai Q., Sun Y., Wang T., Modi P., Cazorla C., Demirci U. B., Ares Fernandez J. R., Leardini F., Aguey-Zinsou K.-F. (2019). How to
Design Hydrogen Storage Materials? Fundamentals, Synthesis, and Storage
Tanks. Advanced Sustainable Systems.

[232] Sordakis K., Tang C., Vogt L. K., Junge H., Dyson P. J., Beller M., Laurenczy G. (2018). Homogeneous
Catalysis for Sustainable Hydrogen Storage in Formic Acid and Alcohols. Chemical Reviews.

[233] Orimo S.-i., Nakamori Y., Eliseo J. R., Zuttel A., Jensen C. M. (2007). Complex Hydrides for Hydrogen Storage. Chemical Reviews.

[234] Park J., Ha J., Muhammad R., Lee H. K., Balderas-Xicohtencatl R., Cheng Y., Ramirez-Cuesta A. J., Streppel B., Hirscher M., Moon H. R., Oh H. (2023). 20 K H_2_ Physisorption
on Metal-Organic Frameworks with Enhanced Dormancy
Compared to Liquid Hydrogen Storage. ACS Applied
Energy Materials.

[235] Habibi P., Saji T. H., Vlugt T. J. H., Moultos O. A., Dey P. (2022). Hydrogen dissociation in Li-decorated borophene and borophene hydride:
An ab-initio study. Applied Surface Science.

[236] Hector L., Herbst J. (2008). Density functional
theory for hydrogen
storage materials: successes and opportunities. Journal of Physics: Condensed Matter.

[237] Peng B., Zhang H., Shao H., Xu Y., Ni G., Zhang R., Zhu H. (2016). Phonon transport properties of two-dimensional
group-IV materials from ab initio calculations. Physical Review B.

[238] Memarian F., Fereidoon A., Darvish Ganji M. (2015). Graphene Young’s
modulus: Molecular mechanics and DFT treatments. Superlattices and Microstructures.

[239] Kuhs W., Chazallon B., Radaelli P., Pauer F. (1997). Cage occupancy
and compressibility of deuterated N_2_-clathrate hydrate
by neutron diffraction. Journal of Inclusion
Phenomena and Molecular Recognition in Chemistry.

[240] Tsimpanogiannis I. N., Economou I. G. (2018). Monte Carlo simulation
studies of
clathrate hydrates: A review. The Journal of
Supercritical Fluids.

[241] Vos W. L., Finger L. W., Hemley R. J., Mao H.-k. (1993). Novel H_2_-H_2_O clathrates at high pressures. Physical Review Letters.

[242] Udachin K. A., Lipkowski J., Tkacz M. (1994). Double clathrate hydrates
with helium and hydrogen. Supramolecular Chemistry.

[243] Dyadin Y. A., Aladko E. Y. (1995). The phase diagram
of the water-hydrogen
system in the crystallization field of solid solutions based on ices
Ih and II at high pressures. Journal of Inclusion
Phenomena and Molecular Recognition in Chemistry.

[244] Dyadin Y. A., Larionov E. G., Manakov A. Y., Zhurko F. V., Aladko E. Y., Mikina T. V., Komarov V. Y. (1999). Clathrate
hydrates
of hydrogen and neon. Mendeleev Communications.

[245] Florusse L. J., Peters C. J., Schoonman J., Hester K. C., Koh C. A., Dec S. F., Marsh K. N., Sloan E. D. (2004). Stable low-pressure hydrogen clusters stored in a binary
clathrate hydrate. Science.

[246] Mi F., Ning F., Vlugt T. J. H., Moultos O. A. (2025). Molecular insight
into hydrogen storage in clathrate hydrates: The effect of different
promoters on the spontaneous nucleation of hydrogen hydrates studied
via microsecond-scale molecular dynamics simulations. Chemical Engineering Journal.

[247] Lee H., Lee J.-w., Kim D. Y., Park J., Seo Y.-T., Zeng H., Moudrakovski I. L., Ratcliffe C. I., Ripmeester J. A. (2005). Tuning clathrate hydrates for hydrogen
storage. Nature.

[248] Papadimitriou N. I., Tsimpanogiannis I. N., Economou I. G., Stubos A. K. (2017). Storage
of H_2_ in clathrate hydrates: Evaluation of different force-fields
used in Monte Carlo simulations. Molecular Physics.

[249] Struzhkin V. V., Militzer B., Mao W. L., Mao H.-k., Hemley R. J. (2007). Hydrogen storage in molecular clathrates. Chemical Reviews.

[250] Manakov A. Y., Skiba S. (2007). Application of clathrate compounds
for hydrogen storage. Russian Journal of General
Chemistry.

[251] English N. J., MacElroy J. (2015). Perspectives on molecular simulation
of clathrate hydrates: Progress, prospects and challenges. Chemical Engineering Science.

[252] Both A. K., Gao Y., Zeng X. C., Cheung C. L. (2021). Gas hydrates
in confined space of nanoporous materials: new frontier in gas storage
technology. Nanoscale.

[253] Davoodabadi A., Mahmoudi A., Ghasemi H. (2021). The potential
of hydrogen
hydrate as a future hydrogen storage medium. Iscience.

[254] Gupta A., Baron G. V., Perreault P., Lenaerts S., Ciocarlan R.-G., Cool P., Mileo P. G., Rogge S., Van Speybroeck V., Watson G. (2021). Hydrogen clathrates:
Next generation hydrogen storage materials. Energy Storage Materials.

[255] Moon S., Lee Y., Seo D., Lee S., Hong S., Ahn Y.-H., Park Y. (2021). Critical hydrogen concentration
of hydrogen-natural gas blends in clathrate hydrates for blue hydrogen
storage. Renewable and Sustainable Energy Reviews.

[256] Zhang Y., Bhattacharjee G., Kumar R., Linga P. (2022). Solidified
hydrogen storage (Solid-HyStore) via clathrate hydrates. Chemical Engineering Journal.

[257] Tanaka H., Matsumoto M. (2013). Statistical mechanical approach to
the thermodynamic stability of clathrate hydrates. Liquid Polymorphism.

[258] Enerdata, 2022 Edition: Annual benchmarks and long-term impacts. 2022; https://www.enerdata.net/publications/reports-presentations/world-energy-trends.html, Accessed: October 15, 2025.

[259] Hematpur, H. ; Abdollahi, R. ; Rostami, S. ; Haghighi, M. ; Blunt, M. J. Review of underground hydrogen storage: Concepts and challenges. Adv. Geo-Energy Res. 2023, 7, 111-131. 10.46690/ager.2023.02.05

[260] Hassanpouryouzband A., Joonaki E., Edlmann K., Haszeldine R. S. (2021). Offshore
geological storage of hydrogen: is this our best option to achieve
net-zero?. ACS Energy Letters.

[261] Heinemann N., Alcalde J., Miocic J. M., Hangx S. J., Kallmeyer J., Ostertag-Henning C., Hassanpouryouzband A., Thaysen E. M., Strobel G. J., Schmidt-Hattenberger C. (2021). Enabling large-scale
hydrogen storage in porous media-the scientific challenges. Energy & Environmental Science.

[262] Krevor S., De Coninck H., Gasda S. E., Ghaleigh N. S., de Gooyert V., Hajibeygi H., Juanes R., Neufeld J., Roberts J. J., Swennenhuis F. (2023). Subsurface carbon dioxide and hydrogen
storage for a sustainable energy future. Nat.
Rev. Earth Environ..

[263] Zivar D., Kumar S., Foroozesh J. (2021). Underground
hydrogen storage: A comprehensive review. Int.
J. Hydrogen Energy.

[264] Pan B., Yin X., Ju Y., Iglauer S. (2021). Underground hydrogen
storage: Influencing parameters and future outlook. Adv. Colloid Interface Sci..

[265] Fernandez-Prini, R. ; Harvey, A. ; Palmer, D. Aqueous Systems at Elevated Temperatures and Pressures 1st Edition; 2004.

[266] Taylor, R. ; Krishna, R. Multicomponent mass transfer, 1st ed.; John Wiley & Sons, 1993; Vol. 2.

[267] Krishna R., Wesselingh J. (1997). The Maxwell-Stefan approach to mass
transfer. Chemical Engineering Science.

[268] Wang L., Jin Z., Huang X., Liu R., Su Y., Zhang Q. (2024). Hydrogen Adsorption in Porous Geological
Materials:
A Review. Sustainability.

[269] Gong Y., Wang S.-H., Zhang Z.-Y., Yang X.-L., Yang Z.-G., Yang H.-G. (2021). Degradation of sunlight exposure
on the high-density polyethylene (HDPE) pipes for transportation of
natural gases. Polymer Degradation and Stability.

[270] Wang H., Shah J., Hawwat S.-E., Huang Q., Khatami A. (2024). A comprehensive review of polyethylene
pipes: Failure
mechanisms, performance models, inspection methods, and repair solutions. Journal of Pipeline Science and Engineering.

[271] Yersak T. A., Baker D. R., Yanagisawa Y., Slavik S., Immel R., Mack-Gardner A., Herrmann M., Cai M. (2017). Predictive model for depressurization-induced
blistering of type IV tank liners for hydrogen storage. International Journal of Hydrogen Energy.

[272] Johnson W. H. (1875). On some remarkable changes produced
in iron and steel
by the action of hydrogen and acids. Nature.

[273] Pressouyre G. (1980). Trap theory of hydrogen embrittlement. Acta Metallurgica.

[274] Louthan M., Caskey G., Donovan J., Rawl D. (1972). Hydrogen embrittlement
of metals. Materials Science and Engineering.

[275] Seita M., Hanson J. P., Gradečak S., Demkowicz M. J. (2015). The dual role of coherent twin boundaries in hydrogen
embrittlement. Nature Communications.

[276] Song J., Curtin W. (2011). A nanoscale mechanism
of hydrogen
embrittlement in metals. Acta Materialia.

[277] Murakami Y., Kanezaki T., Mine Y. (2010). Hydrogen effect
against
hydrogen embrittlement. Metallurgical and Materials
Transactions A.

[278] Wang M., Akiyama E., Tsuzaki K. (2007). Effect of hydrogen
on the fracture behavior of high strength steel during slow strain
rate test. Corrosion Science.

[279] Park G. T., Koh S. U., Jung H. G., Kim K. Y. (2008). Effect
of microstructure on the hydrogen trapping efficiency and hydrogen
induced cracking of linepipe steel. Corrosion
Science.

[280] Turnbull A. (2015). Perspectives
on hydrogen uptake, diffusion and trapping. International Journal of Hydrogen Energy.

[281] Suzuki N., Ishii N., Miyagawa T., Harada H. (1993). Estimation
of delayed fracture property of steels. Tetsu-to-Hagané.

[282] Han Y., Jing H., Xu L. (2012). Welding heat
input effect on the
hydrogen permeation in the X80 steel welded joints. Materials Chemistry and Physics.

[283] Bhadeshia H. K. D. H. (2016). Prevention of hydrogen embrittlement
in steels. ISIJ international.

[284] Young K. T., Smith C., Krentz T. M., Hitchcock D. A., Vogel E. M. (2021). Graphene synthesized by chemical
vapor deposition as
a hydrogen isotope permeation barrier. Carbon.

[285] Takahashi J., Kawakami K., Tarui T. (2012). Direct observation
of hydrogen-trapping sites in vanadium carbide precipitation steel
by atom probe tomography. Scripta Materialia.

[286] Haley D., Merzlikin S. V., Choi P., Raabe D. (2014). Atom probe
tomography observation of hydrogen in high-Mn steel and silver charged
via an electrolytic route. international journal
of hydrogen energy.

[287] Chen Y.-S., Haley D., Gerstl S. S., London A. J., Sweeney F., Wepf R. A., Rainforth W. M., Bagot P. A., Moody M. P. (2017). Direct observation of individual
hydrogen atoms at trapping sites in a ferritic steel. Science.

[288] Chen Y.-S., Lu H., Liang J., Rosenthal A., Liu H., Sneddon G., McCarroll I., Zhao Z., Li W., Guo A., Cairney J. M. (2020). Observation of hydrogen trapping at dislocations, grain
boundaries, and precipitates. Science.

[289] Maxelon M., Pundt A., Pyckhout-Hintzen W., Barker J., Kirchheim R. (2001). Interaction of hydrogen and deuterium
with dislocations in palladium as observed by small angle neutron
scattering. Acta materialia.

[290] Malard B., Remy B., Scott C., Deschamps A., Chene J., Dieudonné T., Mathon M. (2012). Hydrogen trapping by
VC precipitates and structural defects in a high strength Fe-Mn-C
steel studied by small-angle neutron scattering. Materials Science and Engineering: A.

[291] Yu H., Díaz A., Lu X., Sun B., Ding Y., Koyama M., He J., Zhou X., Oudriss A., Feaugas X., Zhang Z. (2024). Hydrogen Embrittlement
as a Conspicuous
Material ChallengeComprehensive Review and Future Directions. Chemical Reviews.

[292] Peng Y., Phan T., Zhai H., Xiong L., Zhang X. (2025). Multiscale computational analysis of crack initiation at the grain
boundaries in hydrogen-charged bi-crystalline alpha-iron. International Journal of Plasticity.

[293] Falcone P. M., Hiete M., Sapio A. (2021). Hydrogen economy and
sustainable development goals: Review and policy insights. Current Opinion in Green and Sustainable Chemistry.

[294] Al Ghafri S. Z., Munro S., Cardella U., Funke T., Notardonato W., Trusler J. P. M., Leachman J., Span R., Kamiya S., Pearce G., Swanger A., Rodriguez E. D., Bajada P., Jiao F., Peng K., Siahvashi A., Johns M. L., May E. F. (2022). Hydrogen liquefaction:
a review of
the fundamental physics, engineering practice and future opportunities. Energy Environ. Sci..

[295] Han S. S., Kang J. K., Lee H. M., van Duin A. C. T., Goddard I., William A. (2005). Liquefaction of H_2_ molecules
upon exterior surfaces of carbon nanotube bundles. Applied Physics Letters.

[296] Tuckerman, M. Statistical Mechanics: Theory and Molecular Simulation; Oxford Graduate Texts; OUP Oxford, 2010.

[297] Rapaport, D. The Art of Molecular Dynamics Simulation, 2nd ed.; Cambridge University Press, 2004.

[298] Landau, D. P. ; Binder, K. A Guide to Monte Carlo Simulations in Statistical Physics, 4th ed.; Cambridge University Press: Cambridge, United Kingdom, 2015.

[299] Evans, D. ; Morriss, G. Statistical Mechanics of Nonequilibrium Liquids, 2nd ed.; Cambridge University Press, 2008.

[300] Jorgensen W. L., Chandrasekhar J., Madura J. D., Impey R. W., Klein M. L. (1983). Comparison of simple potential functions for simulating
liquid water. J. Chem. Phys..

[301] Martin M. G., Siepmann J. I. (1998). Transferable Potentials
for Phase
Equilibria. 1. United-Atom Description of n-Alkanes. The Journal of Physical Chemistry B.

[302] Blazquez S., Conde M. M., Abascal J. L. F., Vega C. (2022). The Madrid-2019
force field for electrolytes in water using TIP4P/2005 and scaled
charges: Extension to the ions F^–^, Br^–^, I^–^, Rb^+^, and Cs^+^. The Journal of Chemical Physics.

[303] Wallqvist, A. ; Mountain, R. D. Reviews in Computational Chemistry; John Wiley & Sons, Ltd, 1999; pp 183–247.

[304] Stillinger F. H., Rahman A. (1974). Improved simulation
of liquid water
by molecular dynamics. The Journal of Chemical
Physics.

[305] Abascal J.
L. F., Vega C. (2005). A general
purpose model for the condensed
phases of water: TIP4P/2005. J. Chem. Phys..

[306] Jorgensen W. L., Tirado-Rives J. (1988). The OPLS [optimized
potentials for
liquid simulations] potential functions for proteins, energy minimizations
for crystals of cyclic peptides and crambin. Journal of the American Chemical Society.

[307] Wang J., Wolf R. M., Caldwell J. W., Kollman P. A., Case D. A. (2004). Development and testing of a general
amber force field. J. Comput. Chem..

[308] Bartolomeu R. A., Franco L. F. (2020). Thermophysical properties
of supercritical
H_2_ from Molecular Dynamics simulations. International Journal of Hydrogen Energy.

[309] Barraco M., Neyertz S., Benes N. E., Brown D. (2023). Comparison
of Eight Classical Lennard-Jones-Based H_2_ Molecular Models
in the Gas Phase at Temperatures and Pressures Relevant to Hydrogen
On-Board Storage Tanks. The Journal of Physical
Chemistry A.

[310] Marx D., Nielaba P. (1992). Path-integral Monte Carlo techniques
for rotational motion in two dimensions: quenched, annealed, and no-spin
quantum-statistical averages. Phys. Rev. A.

[311] Cracknell R. F. (2001). Molecular simulation of hydrogen
adsorption in graphitic
nanofibres. Phys. Chem. Chem. Phys..

[312] Buch V. (1994). Path integral simulations of mixed
para-D_2_ and ortho-D_2_ clusters: The orientational
effects. J. Chem. Phys..

[313] Wang S., Hou K., Heinz H. (2021). Accurate and
Compatible
Force Fields for Molecular Oxygen, Nitrogen, and Hydrogen to Simulate
Gases, Electrolytes, and Heterogeneous Interfaces. Journal of Chemical Theory and Computation.

[314] Śmiechowski M. (2015). Molecular hydrogen solvated in water-A
computational
study. The Journal of Chemical Physics.

[315] Yang J., Ren Y., Tian A.-m., Sun H. (2000). COMPASS Force
Field for 14 Inorganic Molecules, He, Ne, Ar, Kr, Xe, H_2_, O_2_, N_2_, NO, CO, CO_2_, NO_2_, CS_2_, and SO_2_, in Liquid Phases. The Journal of Physical Chemistry B.

[316] Sun H. (1998). COMPASS: an ab initio force-field
optimized for condensed-phase applications
overview with details on alkane and benzene compounds. The Journal of Physical Chemistry B.

[317] Getman R. B., Bae Y.-S., Wilmer C. E., Snurr R. Q. (2012). Review
and Analysis of Molecular Simulations of Methane, Hydrogen, and Acetylene
Storage in Metal Organic Frameworks. Chemical
Reviews.

[318] Boccara, N. Essentials of Mathematica: With Applications to Mathematics and Physics; Springer: New York, 2007; pp 445–448.

[319] Mason E. A., Rice W. E. (1954). The Intermolecular
Potentials of
Helium and Hydrogen. The Journal of Chemical
Physics.

[320] Lenhard J., Stephan S., Hasse H. (2024). On the History
of the
Lennard-Jones Potential. Annalen der Physik.

[321] Hirschfelder, J. O. ; Curtiss, C. F. ; Bird, R. B. Molecular Theory of Gases and Fluids; Wiley: New York, 1954.

[322] Rzepka M., Lamp P., de la Casa-Lillo M. A. (1998). Physisorption
of Hydrogen on Microporous Carbon and Carbon Nanotubes. J. Phys. Chem. B.

[323] Ferrando N., Ungerer P. (2007). Hydrogen/hydrocarbon phase equilibrium
modelling with a cubic equation of state and a Monte Carlo method. Fluid Phase Equilib..

[324] Caviedes D., Cabria I. (2022). Grand Canonical Monte Carlo simulations
of the hydrogen storage capacities of slit-shaped pores, nanotubes
and torusenes. Int. J. Hydrogen Energy.

[325] Yang Q., Zhong C. (2005). Molecular simulation
of adsorption
and diffusion of hydrogen in metal- organic frameworks. The Journal of Physical Chemistry B.

[326] Engin C., Vrabec J., Hasse H. (2011). On the difference
between
a point multipole and an equivalent linear arrangement of point charges
in force field models for vapour-liquid equilibria; partial charge
based models for 59 real fluids. Mol. Phys..

[327] Darkrim F., Vermesse J., Malbrunot P., Levesque D. (1999). Monte Carlo simulations of nitrogen and hydrogen physisorption
at high pressures and room temperature. Comparison with experiments. J. Chem. Phys..

[328] Marx D., Nielaba P. (1992). Path-integral Monte Carlo techniques
for rotational motion in two dimensions: Quenched, annealed, and no-spin
quantum-statistical averages. Physical Review
A.

[329] Li Z., Li T., Meng L., Wang X., Sun H., Zhang M., Kou J. (2025). Molecular
mechanisms of hydrogen
leakage and blockage in kaolinite nano-cracks for underground hydrogen
storage. Physics of Fluids.

[330] Bouanich J.-P. (1992). Site-site
Lennard-Jones potential parameters for N_2_, O_2_, H_2_, CO and CO_2_. Journal
of Quantitative Spectroscopy and Radiative Transfer.

[331] Sun Y., DeJaco R. F., Li Z., Tang D., Glante S., Sholl D. S., Colina C. M., Snurr R. Q., Thommes M., Hartmann M., Siepmann J. I. (2021). Fingerprinting
diverse nanoporous
materials for optimal hydrogen storage conditions using meta-learning. Science Advances.

[332] Belof J. L., Stern A. C., Space B. (2008). An accurate and transferable
intermolecular diatomic hydrogen potential for condensed phase simulation. J. Chem. Theory Comput..

[333] Belof J. L., Stern A. C., Space B. (2009). A predictive model
of hydrogen sorption for metal-organic materials. The Journal of Physical Chemistry C.

[334] Mondal S., Ghosh S., Chattaraj P. (2013). A molecular
dynamics study on sI hydrogen hydrate. Journal
of Molecular Modeling.

[335] Silvera I. F., Goldman V. V. (1978). The isotropic intermolecular potential
for H_2_ and D_2_ in the solid and gas phases. J. Chem. Phys..

[336] Alavi S., Ripmeester J., Klug D. (2005). Molecular-dynamics
study of structure II hydrogen clathrates. The
Journal of Chemical Physics.

[337] Levesque D., Gicquel A., Darkrim F. L., Kayiran S. B. (2002). Monte Carlo
simulations of hydrogen storage in carbon nanotubes. Journal of Physics: Condensed Matter.

[338] Gu C., Gao G.-H., Yu Y.-X., Nitta T. (2002). Simulation
for separation
of hydrogen and carbon monoxide by adsorption on single-walled carbon
nanotubes. Fluid Phase Equilibria.

[339] Sesé L. M. (1993). Feynman-Hibbs quantum effective potentials
for Monte
Carlo simulations of liquid neon. Molecular
Physics.

[340] Kumar A. A., Bhatia S. K. (2005). Quantum effect induced reverse kinetic
molecular sieving in microporous materials. Physical Review Letters.

[341] Papadimitriou N. I., Tsimpanogiannis I. N., Economou I. G., Stubos A. K. (2016). The effect
of lattice constant on the storage capacity of hydrogen hydrates:
a Monte Carlo study. Molecular Physics.

[342] Michalis V. K., Economou I. G., Stubos A. K., Tsimpanogiannis I. N. (2022). Phase equilibria
molecular simulations of hydrogen hydrates via the direct phase coexistence
approach. The Journal of Chemical Physics.

[343] Alavi S., Klug D., Ripmeester J. (2008). Simulations
of structure II H_2_ and D_2_ clathrates: potentials
incorporating quantum corrections. The Journal
of Chemical Physics.

[344] Wang W. (2003). Atomic-potential parameters for H_2_ and D_2_:
quantum corrections in the calculation of second-virial coefficients. Journal of Quantitative Spectroscopy and Radiative Transfer.

[345] Vega C., Abascal J. L. (2011). Simulating water
with rigid non-polarizable
models: a general perspective. Phys. Chem. Chem.
Phys..

[346] Berendsen H. J. C., Grigera J. R., Straatsma T. P. (1987). The missing
term in effective pair potentials. Journal of
Physical Chemistry.

[347] Zeron I., Abascal J., Vega C. (2019). A force field of Li^+^, Na^+^, K^+^, Mg^2+^, Ca^2+^, Cl^–^, and SO_4_
^2‑^ in
aqueous solution based on the TIP4P/2005 water model and scaled charges
for the ions. The Journal of Chemical Physics.

[348] Tsimpanogiannis I. N., Moultos O. A., Franco L. F. M., Spera M. B. M., Erdos M., Economou I. G. (2019). Self-diffusion coefficient
of bulk
and confined water: a critical review of classical molecular simulation
studies. Molecular Simulation.

[349] Abascal J. L., Vega C. (2005). A general purpose model
for the condensed
phases of water: TIP4P/2005. The Journal of
Chemical Physics.

[350] Habibi P., Polat H. M., Blazquez S., Vega C., Dey P., Vlugt T. J. H., Moultos O. A. (2024). Accurate
Free Energies of Aqueous
Electrolyte Solutions from Molecular Simulations with Non-polarizable
Force Fields. The Journal of Physical Chemistry
Letters.

[351] Wagner W., Pruß A. (2002). The IAPWS formulation 1995 for the
thermodynamic properties of ordinary water substance for general and
scientific use. J. Phys. Chem. Ref. Data.

[352] Lemmon, E. W. ; Huber, M. L. ; McLinden, M. O. NIST reference fluid thermodynamic and transport properties-REFPROP. NIST Standard Reference Database 2002, 23, v7.

[353] Rick S. W. (2004). A reoptimization of the five-site
water potential (TIP5P)
for use with Ewald sums. J. Chem. Phys..

[354] Jiang H., Moultos O. A., Economou I. G., Panagiotopoulos A. Z. (2016). Hydrogen-Bonding
Polarizable Intermolecular Potential Model for Water. Journal of Physical Chemistry B.

[355] Kiss P. T., Baranyai A. (2013). A Systematic Development
of a Polarizable
Potential of Water. Journal of Chemical Physics.

[356] Laury M. L., Wang L.-P., Pande V. S., Head-Gordon T., Ponder J. W. (2015). Revised parameters for the AMOEBA
polarizable atomic
multipole water model. J. Phys. Chem. B.

[357] Xiong Y., Izadi S., Onufriev A. V. (2022). Fast Polarizable
Water Model for Atomistic Simulations. J. Chem.
Theory Comput..

[358] Abascal J. L. F., Sanz E., García
Fernández R., Vega C. (2005). A potential model for the study of
ices and amorphous water: TIP4P/Ice. The Journal
of Chemical Physics.

[359] Mahoney M. W., Jorgensen W. L. (2000). A five-site
model for liquid water
and the reproduction of the density anomaly by rigid, nonpolarizable
potential functions. J. Chem. Phys..

[360] Jorgensen W. L., Maxwell D. S., Tirado-Rives J. (1996). Development
and testing of the OPLS all-atom force field on conformational energetics
and properties of organic liquids. J. Amer.
Chem. Soc..

[361] Mark P., Nilsson L. (2001). Structure and dynamics
of the TIP3P,
SPC, and SPC/E water models at 298 K. J. Phys.
Chem. A.

[362] Habershon S., Markland T. E., Manolopoulos D. E. (2009). Competing
quantum effects in the dynamics of a flexible water model. The Journal of Chemical Physics.

[363] Rick S. W. (2001). Simulations
of ice and liquid water over a range of
temperatures using the fluctuating charge model. The Journal of Chemical Physics.

[364] Rick S. W., Freeman D. L. (2010). Proton disorder
and the dielectric
constant of type II clathrate hydrates. The
Journal of Chemical Physics.

[365] Rick S. W., Stuart S. J., Berne B. J. (1994). Dynamical
fluctuating
charge force fields: Application to liquid water. The Journal of Chemical Physics.

[366] Cendagorta J. R., Powers A., Hele T. J., Marsalek O., Bačić Z., Tuckerman M. E. (2016). Competing
quantum effects in the
free energy profiles and diffusion rates of hydrogen and deuterium
molecules through clathrate hydrates. Physical
Chemistry Chemical Physics.

[367] García
Fernández R., Abascal J. L., Vega C. (2006). The melting
point of ice Ih for common water models calculated from direct coexistence
of the solid-liquid interface. The Journal of
Chemical Physics.

[368] Conde M., Rovere M., Gallo P. (2017). High precision
determination
of the melting points of water TIP4P/2005 and water TIP4P/Ice models
by the direct coexistence technique. The Journal
of Chemical Physics.

[369] Conde M., Vega C. (2010). Determining the three-phase
coexistence
line in methane hydrates using computer simulations. The Journal of Chemical Physics.

[370] Zeron I. M., Abascal J. L. F., Vega C. (2019). A force field of Li^+^,
Na^+^, K^+^, Mg^2+^, Ca^+^, Cl^–^, and SO_4_
^2‑^ in
aqueous solution based on the TIP4P/2005 water model and scaled charges
for the ions. J. Chem. Phys..

[371] Joung I. S., Cheatham T. E. (2008). Determination of alkali and halide
monovalent ion parameters for use in explicitly solvated biomolecular
simulations. The Journal of Physical Chemistry
B.

[372] Kiss P. T., Baranyai A. (2014). A new polarizable force
field for
alkali and halide ions. The Journal of Chemical
Physics.

[373] Dočkal J., Lísal M., Moučka F. (2022). Polarizable
force fields for accurate molecular simulations of aqueous solutions
of electrolytes, crystalline salts, and solubility: Li^+^, Na^+^, K^+^, Rb^+^, F^–^, Cl^–^, Br^–^, I^–^. Journal of Molecular Liquids.

[374] Smith D. E., Dang L. X. (1994). Computer simulations
of NaCl association
in polarizable water. The Journal of Chemical
Physics.

[375] Jensen K.
P., Jorgensen W. L. (2006). Halide,
Ammonium, and Alkali Metal
Ion Parameters for Modeling Aqueous Solutions. Journal of Chemical Theory and Computation.

[376] Straatsma T. P., Berendsen H. J. C. (1988). Free
energy of ionic hydration: Analysis
of a thermodynamic integration technique to evaluate free energy differences
by molecular dynamics simulations. J. Chem.
Phys..

[377] Dang L. X. (1992). Development of nonadditive intermolecular
potentials
using molecular dynamics: Solvation of Li^+^ and F^–^ ions in polarizable water. The Journal of
Chemical Physics.

[378] Reif M. M., Hünenberger P.
H. (2011). Computation of methodology-independent
single-ion solvation properties from molecular simulations. IV. Optimized
Lennard-Jones interaction parameter sets for the alkali and halide
ions in water. The Journal of Chemical Physics.

[379] Leontyev I. V., Stuchebrukhov A. A. (2009). Electronic
continuum model for molecular
dynamics simulations. The Journal of Chemical
Physics.

[380] Leontyev I. V., Stuchebrukhov A. A. (2010). Electronic
Continuum Model for Molecular
Dynamics Simulations of Biological Molecules. Journal of Chemical Theory and Computation.

[381] Leontyev I. V., Stuchebrukhov A. A. (2012). Polarizable
Mean-Field Model of Water
for Biological Simulations with AMBER and CHARMM Force Fields. Journal of Chemical Theory and Computation.

[382] Leontyev I., Stuchebrukhov A. (2011). Accounting
for electronic polarization
in non-polarizable force fields. Phys. Chem.
Chem. Phys..

[383] Kostal V., Jungwirth P., Martinez-Seara H. (2023). Nonaqueous
Ion Pairing Exemplifies the Case for Including Electronic Polarization
in Molecular Dynamics Simulations. J. Phys.
Chem. Lett..

[384] Blazquez S., Conde M. M., Vega C. (2023). Scaled charges for
ions: An improvement but not the final word for modeling electrolytes
in water. The Journal of Chemical Physics.

[385] Blazquez S., Zeron I., Conde M., Abascal J., Vega C. (2020). Scaled charges at work: Salting out
and interfacial tension of methane
with electrolyte solutions from computer simulations. Fluid Phase Equilib..

[386] Shi W., Maginn E. J. (2008). Atomistic Simulation of the Absorption of Carbon Dioxide
and Water in the Ionic Liquid 1-n-Hexyl-3-methylimidazolium Bis­(trifluoromethylsulfonyl)­imide
([hmim]­[Tf2N]. The Journal of Physical Chemistry
B.

[387] Shi W., Maginn E. J. (2008). Molecular Simulation
and Regular Solution Theory Modeling
of Pure and Mixed Gas Absorption in the Ionic Liquid 1-n-Hexyl-3-methylimidazolium
Bis­(Trifluoromethylsulfonyl)­amide ([hmim]­[Tf2N]). The Journal of Physical Chemistry B.

[388] Kelkar M. S., Maginn E. J. (2007). Calculating the
Enthalpy of Vaporization
for Ionic Liquid Clusters. The Journal of Physical
Chemistry B.

[389] Sambasivarao S. V., Acevedo O. (2009). Development
of OPLS-AA Force Field
Parameters for 68 Unique Ionic Liquids. Journal
of Chemical Theory and Computation.

[390] Koller T., Ramos J., Garrido N. M., Fröba A. P., and I. G. E. (2012). Development of a united-atom force
field for 1-ethyl-3-methylimidazolium
tetracyanoborate ionic liquid. Molecular Physics.

[391] Singh R., Marin-Rimoldi E., Maginn E. J. (2015). A Monte Carlo Simulation
Study To Predict the Solubility of Carbon Dioxide, Hydrogen, and Their
Mixture in the Ionic Liquids 1-Alkyl-3-methylimidazolium bis­(trifluoromethanesulfonyl)­amide
([Cnmim+]­[Tf2N-], n = 4, 6). Industrial &
Engineering Chemistry Research.

[392] Doherty B., Zhong X., Acevedo O. (2018). Virtual Site OPLS Force
Field for Imidazolium-Based Ionic Liquids. The
Journal of Physical Chemistry B.

[393] Cui K., Yethiraj A., Schmidt J. R. (2019). Influence of Charge
Scaling on the
Solvation Properties of Ionic Liquid Solutions. The Journal of Physical Chemistry B.

[394] Celebi A. T., Vlugt T. J. H., Moultos O. A. (2019). Structural,
Thermodynamic,
and Transport Properties of Aqueous Reline and Ethaline Solutions
from Molecular Dynamics Simulations. The Journal
of Physical Chemistry B.

[395] Celebi A. T., Vlugt T. J. H., Moultos O. A. (2021). Thermal conductivity
of aqueous solutions of reline, ethaline, and glyceline deep eutectic
solvents; a molecular dynamics simulation study. Molecular Physics.

[396] Verma A. K., Thorat A. S., Shah J. K. (2025). Predicting Ionic
Conductivity of Imidazolium-Based Ionic Liquid Mixtures Using Quantum-Mechanically
Derived Partial Charges in the Condensed Phase. The Journal of Physical Chemistry B.

[397] Habibi P., Rahbari A., Blazquez S., Vega C., Dey P., Vlugt T. J. H., Moultos O. A. (2022). A New Force
Field for OH^–^ for Computing Thermodynamic and Transport
Properties of H_2_ and O_2_ in Aqueous NaOH and
KOH Solutions. J. Phys. Chem. B.

[398] Nezbeda I., Moučka F., Smith W. R. (2016). Recent progress
in molecular simulation of aqueous electrolytes: force fields, chemical
potentials and solubility. Molecular Physics.

[399] Rappe A. K., Casewit C. J., Colwell K. S., Goddard W. A. I., Skiff W. M. (1992). UFF, a full periodic table force
field for molecular
mechanics and molecular dynamics simulations. Journal of the American Chemical Society.

[400] Mayo S. L., Olafson B. D., Goddard W. A. (1990). DREIDING:
a generic
force field for molecular simulations. The Journal
of Physical Chemistry.

[401] Vanduyfhuys L., Vandenbrande S., Verstraelen T., Schmid R., Waroquier M., Van Speybroeck V. (2015). QuickFF: A
program for a quick and easy derivation of force fields for metal-organic
frameworks from ab initio input. Journal of
Computational Chemistry.

[402] Coupry D. E., Addicoat M. A., Heine T. (2016). Extension of the Universal
Force Field for Metal-Organic Frameworks. Journal
of Chemical Theory and Computation.

[403] Verploegh R. J., Kulkarni A., Boulfelfel S. E., Haydak J. C., Tang D., Sholl D. S. (2019). Screening Diffusion
of Small Molecules in Flexible Zeolitic Imidazolate Frameworks Using
a DFT-Parameterized Force Field. The Journal
of Physical Chemistry C.

[404] Weng T., Schmidt J. R. (2019). Flexible and Transferable ab Initio
Force Field for Zeolitic Imidazolate Frameworks: ZIF-FF. The Journal of Physical Chemistry A.

[405] Sun H., Jin Z., Yang C., Akkermans R. L. C., Robertson S. H., Spenley N. A., Miller S., Todd S. M. (2016). COMPASS
II: extended coverage for polymer and drug-like molecule databases. Journal of Molecular Modeling.

[406] Sun H., Mumby S. J., Maple J. R., Hagler A. T. (1994). An ab initio
CFF93
all-atom force field for polycarbonates. Journal
of the American Chemical society.

[407] Voyiatzis E., Stroeks A. (2022). Atomistic Modeling
of Hydrogen and
Oxygen Solubility in Semicrystalline PA-6 and HDPE Materials. The Journal of Physical Chemistry B.

[408] Direct Force Field. https://www.aeontechnology.com/index.php, Accessed: October 15, 2025.

[409] O’Connor T. C., Andzelm J., Robbins M. O. (2015). AIREBO-M: A reactive
model for hydrocarbons at extreme pressures. The Journal of Chemical Physics.

[410] Mabuchi T., Tokumasu T. (2014). Effect of bound state of water on
hydronium ion mobility in hydrated Nafion using molecular dynamics
simulations. The Journal of Chemical Physics.

[411] Smith J. S., Borodin O., Smith G. D. (2004). A Quantum
Chemistry
Based Force Field for Poly­(dimethylsiloxane). The Journal of Physical Chemistry B.

[412] Ungerer P., Beauvais C., Delhommelle J., Boutin A., Rousseau B., Fuchs A. H. (2000). Optimization of
the anisotropic united atoms intermolecular potential for n-alkanes. The Journal of Chemical Physics.

[413] Memari P., Lachet V., Klopffer M.-H., Flaconneche B., Rousseau B. (2012). Gas mixture solubilities in polyethylene
below its
melting temperature: Experimental and molecular simulation studies. Journal of Membrane Science.

[414] Bobbitt N. S., Snurr R. Q. (2019). Molecular modelling
and machine learning
for high-throughput screening of metal-organic frameworks for hydrogen
storage. Molecular Simulation.

[415] Heinen J., Dubbeldam D. (2018). On flexible
force fields for metal-organic
frameworks: Recent developments and future prospects. Wiley Interdisciplinary Reviews: Computational Molecular Science.

[416] Franz D., Forrest K. A., Pham T., Space B. (2016). Accurate H_2_ Sorption Modeling in the rht-MOF NOTT-112
Using Explicit
Polarization. Crystal Growth & Design.

[417] Krokidas P., Moncho S., Brothers E. N., Castier M., Economou I. G. (2018). Tailoring the gas separation efficiency
of metal organic
framework ZIF-8 through metal substitution: A computational study. Physical Chemistry Chemical Physics.

[418] Krokidas P., Moncho S., Brothers E. N., Economou I. G. (2020). Defining
New Limits in Gas Separations Using Modified ZIF Systems. ACS Applied Materials & Interfaces.

[419] Harris J. G., Yung K. H. (1995). Carbon Dioxide’s
Liquid-Vapor
Coexistence Curve And Critical Properties as Predicted by a Simple
Molecular Model. The Journal of Physical Chemistry.

[420] Siu S. W. I., Pluhackova K., Böckmann R. A. (2012). Optimization
of the OPLS-AA Force Field for Long Hydrocarbons. Journal of Chemical Theory and Computation.

[421] Gonzalez-Salgado D., Vega C. (2016). A new intermolecular
potential for
simulations of methanol: The OPLS/2016 model. J. Chem. Phys..

[422] Cygan R. T., Liang J.-J., Kalinichev A. G. (2004). Molecular
models of hydroxide, oxyhydroxide, and clay phases and the development
of a general force field. J. Phys. Chem. B.

[423] Cygan R. T., Greathouse J. A., Kalinichev A. G. (2021). Advances
in clayff molecular simulation of layered and nanoporous materials
and their aqueous interfaces. The Journal of
Physical Chemistry C.

[424] Kerisit S. (2011). Water structure at hematite-water interfaces. Geochimica et Cosmochimica Acta.

[425] Heinz H., Lin T.-J., Kishore R. M., Emami F. S. (2013). Thermodynamically
consistent force fields for the assembly of inorganic, organic, and
biological nanostructures: the INTERFACE force field. Langmuir.

[426] Hagler A., Lifson S., Dauber P. (1979). Consistent force field
studies of intermolecular forces in hydrogen-bonded crystals. 2. A
benchmark for the objective comparison of alternative force fields. Journal of the American Chemical Society.

[427] Ungerer P., Collell J., Yiannourakou M. (2015). Molecular
modeling of the volumetric and thermodynamic properties of kerogen:
Influence of organic type and maturity. Energy
& Fuels.

[428] Jorgensen W. L., Maxwell D. S., Tirado-Rives J. (1996). Development
and testing of the OPLS all-atom force field on conformational energetics
and properties of organic liquids. J. Amer.
Chem. Soc..

[429] Siboulet B., Coasne B., Dufreche J.-F., Turq P. (2011). Hydrophobic
transition in porous amorphous silica. The Journal
of Physical Chemistry B.

[430] Ungerer P., Rigby D., Leblanc B., Yiannourakou M. (2014). Sensitivity
of the aggregation behaviour of asphaltenes to molecular weight and
structure using molecular dynamics. Molecular
Simulation.

[431] Xiao S., Edwards S. A., Grater F. (2011). A new transferable
forcefield for simulating the mechanics of CaCO_3_ crystals. The Journal of Physical Chemistry C.

[432] Raiteri P., Gale J. D., Quigley D., Rodger P. M. (2010). Derivation
of an accurate force-field for simulating the growth of calcium carbonate
from aqueous solution: A new model for the calcite- water interface. The Journal of Physical Chemistry C.

[433] Daw M. S., Baskes M. I. (1983). Semiempirical, Quantum
Mechanical
Calculation of Hydrogen Embrittlement in Metals. Phys. Rev. Lett..

[434] Daw M. S., Baskes M. I. (1984). Embedded-atom method: Derivation
and application to impurities, surfaces, and other defects in metals. Phys. Rev. B.

[435] Hale L. M., Wong B. M., Zimmerman J. A., Zhou X. W. (2013). Atomistic potentials for palladium-silver hydrides. Modelling and Simulation in Materials Science and Engineering.

[436] Angelo J. E., Moody N. R., Baskes M. I. (1995). Trapping
of hydrogen
to lattice defects in nickel. Modelling and
Simulation in Materials Science and Engineering.

[437] Ramasubramaniam A., Itakura M., Carter E. A. (2009). Interatomic
potentials
for hydrogen in *α*-iron based on density functional
theory. Phys. Rev. B.

[438] Wen M. (2021). A new interatomic potential describing Fe-H and H-H
interactions
in bcc iron. Computational Materials Science.

[439] Kumar P., Ludhwani M. M., Das S., Gavini V., Kanjarla A., Adlakha I. (2023). Effect of hydrogen
on plasticity
of *α*-Fe: A multi-scale assessment. International Journal of Plasticity.

[440] Zhou X., Zimmerman J., Wong B., Hoyt J. (2008). An embedded-atom
method interatomic potential for Pd-H alloys. Journal of Materials Research.

[441] Bonny G., Grigorev P., Terentyev D. (2014). On the binding
of nanometric hydrogen-helium clusters in tungsten. Journal of Physics: Condensed Matter.

[442] Tehranchi A., Curtin W. (2017). Atomistic study of
hydrogen embrittlement
of grain boundaries in nickel: I. Fracture. Journal of the Mechanics and Physics of Solids.

[443] Mason D. R., Nguyen-Manh D., Lindblad V. W., Granberg F. G., Lavrentiev M. Y. (2023). An empirical
potential for simulating hydrogen isotope
retention in highly irradiated tungsten. Journal
of Physics: Condensed Matter.

[444] Zhou X., Nowak C., Skelton R., Foster M., Ronevich J., San Marchi C., Sills R. (2022). An Fe-Ni-Cr-H interatomic
potential and predictions of hydrogen-affected stacking fault energies
in austenitic stainless steels. International
Journal of Hydrogen Energy.

[445] Baskes M. I. (1992). Modified embedded-atom potentials
for cubic materials
and impurities. Phys. Rev. B.

[446] Lee B.-J., Baskes M. I. (2000). Second nearest-neighbor
modified
embedded-atom-method potential. Phys. Rev. B.

[447] Ko W.-S., Shim J.-H., Lee B.-J. (2011). Atomistic
modeling
of the Al-H and Ni-H systems. Journal of Materials
Research.

[448] Shim J.-H., Ko W.-S., Kim K.-H., Lee H.-S., Lee Y.-S., Suh J.-Y., Cho Y. W., Lee B.-J. (2013). Prediction
of hydrogen permeability in V-Al and V-Ni alloys. Journal of Membrane Science.

[449] Lee B.-J., Jang J.-W. (2007). A modified embedded-atom method interatomic
potential for the Fe-H system. Acta Materialia.

[450] Shim J.-H., Lee Y.-S., Fleury E., Cho Y. W., Ko W.-S., Lee B.-J. (2011). A modified embedded-atom
method interatomic
potential for the V-H system. Calphad.

[451] Lee B.-M., Lee B.-J. (2014). A Comparative Study
on Hydrogen Diffusion
in Amorphous and Crystalline Metals Using a Molecular Dynamics Simulation. Metallurgical and Materials Transactions A.

[452] Mishin Y., Mehl M., Papaconstantopoulos D. (2005). Phase stability
in the Fe-Ni system: Investigation by first-principles calculations
and atomistic simulations. Acta Materialia.

[453] Apostol F., Mishin Y. (2010). Angular-dependent interatomic
potential
for the aluminum-hydrogen system. Phys. Rev.
B.

[454] Starikov S., Smirnova D., Pradhan T., Gordeev I., Drautz R., Mrovec M. (2022). Angular-dependent interatomic
potential
for large-scale atomistic simulation of the Fe-Cr-H ternary system. Phys. Rev. Mater..

[455] Smirnova D., Starikov S., Vlasova A. (2018). New interatomic potential
for simulation of pure magnesium and magnesium hydrides. Computational Materials Science.

[456] Pettifor D. G., Oleinik I. I. (1999). Analytic bond-order
potentials beyond
Tersoff-Brenner. I. Theory. Phys. Rev. B.

[457] Ward D. K., Zhou X. W., Wong B. M., Doty F. P., Zimmerman J. A. (2012). Analytical bond-order potential for
the cadmium telluride
binary system. Phys. Rev. B.

[458] Zhou X. W., Ward D. K., Foster M. E. (2018). A bond-order potential
for the Al-Cu-H ternary system. New J. Chem..

[459] Zhou X. W., Ward D. K., Foster M., Zimmerman J. A. (2015). An analytical
bond-order potential for the copper-hydrogen binary system. Journal of Materials Science.

[460] Behler J., Parrinello M. (2007). Generalized
Neural-Network Representation
of High-Dimensional Potential-Energy Surfaces. Phys. Rev. Lett..

[461] Bartok A. P., Payne M. C., Kondor R., Csányi G. (2010). Gaussian Approximation
Potentials: The Accuracy of Quantum Mechanics, without the Electrons. Phys. Rev. Lett..

[462] Shapeev A. V. (2016). Moment
Tensor Potentials: A Class of Systematically
Improvable Interatomic Potentials. Multiscale
Modeling & Simulation.

[463] Mishin Y. (2021). Machine-learning
interatomic potentials for materials
science. Acta Materialia.

[464] Kimizuka H., Thomsen B., Shiga M. (2022). Artificial neural network-based
path integral simulations of hydrogen isotope diffusion in palladium. Journal of Physics: Energy.

[465] Kwon H., Shiga M., Kimizuka H., Oda T. (2023). Accurate description
of hydrogen diffusivity in bcc metals using machine-learning moment
tensor potentials and path-integral methods. Acta Materialia.

[466] Qi J., Ko T. W., Wood B. C., Pham T. A., Ong S. P. (2024). Robust
training of machine learning interatomic potentials with dimensionality
reduction and stratified sampling. npj Computational
Materials.

[467] Meng F.-S., Du J.-P., Shinzato S., Mori H., Yu P., Matsubara K., Ishikawa N., Ogata S. (2021). General-purpose neural
network interatomic potential for the *α*-iron
and hydrogen binary system: Toward atomic-scale understanding of hydrogen
embrittlement. Phys. Rev. Mater..

[468] Shuang F., Ji Y., Wei Z., Dong C., Gao W., Laurenti L., Dey P. (2025). Decoding the
hidden dynamics of super-Arrhenius
hydrogen diffusion in multi-principal element alloys via machine learning. Acta Materialia.

[469] Zhou X.-Y., Wu H.-H., Zhou M., Wang L., Lookman T., Mao X. (2025). Enhanced hydrogen embrittlement resistance
of FeCoNiCrMn multi-principal element alloys via local chemical ordering
and grain boundary segregation. Acta Materialia.

[470] Pun G. P. P., Batra R., Ramprasad R., Mishin Y. (2019). Physically informed artificial neural networks for
atomistic modeling of materials. Nature Communications.

[471] Pun G. P. P., Yamakov V., Hickman J., Glaessgen E. H., Mishin Y. (2020). Development of a general-purpose
machine-learning interatomic
potential for aluminum by the physically informed neural network method. Phys. Rev. Mater..

[472] Lin Y.-S., Pun G. P. P., Mishin Y. (2022). Development of a physically-informed
neural network interatomic potential for tantalum. Computational Materials Science.

[473] Thompson A. P., Aktulga H. M., Berger R., Bolintineanu D. S., Brown W. M., Crozier P. S., in ’t
Veld P. J., Kohlmeyer A., Moore S. G., Nguyen T. D., Shan R., Stevens M. J., Tranchida J., Trott C., Plimpton S. J. (2022). LAMMPS
- a flexible simulation tool for particle-based materials modeling
at the atomic, meso, and continuum scales. Comp.
Phys. Comm..

[474] Shuang F., Liu K., Ji Y., Gao W., Laurenti L., Dey P. (2025). Modeling extensive defects in metals
through classical potential-guided sampling and automated configuration
reconstruction. npj Computational Materials.

[475] Plimpton S. (1995). Fast parallel algorithms for short-range
molecular
dynamics. Journal of Computational Physics.

[476] Berendsen H. J., van der Spoel D., van Drunen R. (1995). GROMACS: A
message-passing parallel molecular dynamics implementation. Computer physics communications.

[477] Hess B., Kutzner C., Van Der
Spoel D., Lindahl E. (2008). GROMACS 4: algorithms for highly
efficient, load-balanced,
and scalable molecular simulation. J. Chem.
Theory Comput..

[478] LAMMPS Development Team, LAMMPS Documentation. https://docs.lammps.org/Intro_website.html, 2025; Large-scale Atomic/Molecular Massively Parallel Simulator.

[479] Todorov I. T., Smith W., Trachenko K., Dove M. T. (2006). DL_POLY_3: new dimensions in molecular dynamics simulations
via massive parallelism. Journal of Materials
Chemistry.

[480] Dubbeldam D., Calero S., Ellis D. E., Snurr R. Q. (2016). RASPA:
molecular simulation software for adsorption and diffusion in flexible
nanoporous materials. Mol. Simul..

[481] Humphrey W., Dalke A., Schulten K. (1996). VMD - Visual
Molecular
Dynamics. Journal of Molecular Graphics.

[482] Hanwell M. D., Curtis D. E., Lonie D. C., Vandermeersch T., Zurek E., Hutchison G. R. (2012). Avogadro:
an advanced semantic chemical
editor, visualization, and analysis platform. Journal of Cheminformatics.

[483] Dubbeldam D., Calero S., Vlugt T. J. H. (2018). iRASPA: GPU-accelerated
visualization software for materials scientists. Mol. Simul..

[484] Stukowski A. (2010). Visualization
and analysis of atomistic simulation
data with OVITO-the Open Visualization Tool. Modelling and Simulation in Materials Science and Engineering.

[485] Cummings P. T., Evans D. J. (1992). Nonequilibrium molecular
dynamics
approaches to transport properties and non-Newtonian fluid rheology. Industrial & Engineering Chemistry Research.

[486] Maginn E. J., Messerly R. A., Carlson D. J., Roe D. R., Elliot J. R. (2018). Best Practices for Computing Transport
Properties 1.
Self-Diffusivity and Viscosity from Equilibrium Molecular Dynamics
[Article v1.0]. Living Journal of Computational
Molecular Science.

[487] Zwanzig R. (1965). Time-Correlation Functions and Transport
Coefficients
in Statistical Mechanics. Annu. Rev. Phys. Chem..

[488] Hagen M. H. J., Lowe C. P., Frenkel D. (1995). Long Time
Tails in
Stress Correlation Functions. 25 Years Non-Equilibrium Stat. 25 Years of Non-Equilibrium Statistical Mechanics.

[489] van der Hoef M. A., Frenkel D. (1990). Long-time Tails of
the Velocity Autocorrelation
Function in Two- and Three-dimensional Lattice-gas Cellular Automata:
A Test of Mode-Coupling Theory. Phys. Rev. A.

[490] Maginn E. J., Messerly R. A., Carlson D. J., Roe D. R., Elliot J. R. (2020). Best practices for computing transport
properties 1.
Self-diffusivity and viscosity from equilibrium molecular dynamics
[article v1. 0]. Living Journal of Computational
Molecular Science.

[491] Jamali S. H., Wolff L., Becker T. M., Groen M. D., Ramdin M., Hartkamp R., Bardow A., Vlugt T. J. H., Moultos O. A. (2019). OCTP: A Tool for On-the-Fly Calculation of Transport
Properties of Fluids with the Order- n Algorithm in LAMMPS. J. Chem. Inf. Model..

[492] Humbert M. T., Zhang Y., Maginn E. J. (2019). PyLAT: Python LAMMPS
Analysis Tools. Journal of Chemical Information
and Modeling.

[493] Dubbeldam D., Ford D. C., Ellis D. E., Snurr R. Q. (2009). A new perspective
on the order-n algorithm for computing correlation functions. Mol. Simul..

[494] Cussler, E. L. Diffusion Coefficients and Diffusion of Interacting Species, 2nd ed.; Cambridge University Press: Cambridge, 2009.

[495] Kärger J., Valiullin R., Brandani S., Caro J., Chmelik C., Chmelka B. F., Coppens M.-O., Farooq S., Freude D., Jobic H., Kruteva M., Mangano E., Pini R., Price W. S., Rajendran A., Ravikovitch P. I., Sastre G., Snurr R. Q., Stepanov A. G., Vasenkov S., Wang Y., Weckhuysen B. M. (2025). Diffusion
in nanoporous materials with special consideration of the measurement
of determining parameters (IUPAC Technical Report). Pure and Applied Chemistry.

[496] Moultos O. A., Zhang Y., Tsimpanogiannis I. N., Economou I. G., Maginn E. J. (2016). System-size corrections for self-diffusion
coefficients calculated from molecular dynamics simulations: The case
of CO_2_, n-alkanes, and poly (ethylene glycol) dimethyl
ethers. J. Chem. Phys..

[497] Dunweg B., Kremer K. (1993). Molecular dynamics simulation of
a polymer chain in solution. The Journal of
Chemical Physics.

[498] Yeh I.-C., Hummer G. (2004). System-size dependence of diffusion
coefficients and viscosities from molecular dynamics simulations with
periodic boundary conditions. J. Phys. Chem.
B.

[499] Celebi A.
T., Jamali S. H., Bardow A., Vlugt T. J. H., Moultos O. A. (2021). Finite-size effects
of diffusion coefficients computed
from molecular dynamics: a review of what we have learned so far. Molecular Simulation.

[500] Jamali S. H., Bardow A., Vlugt T. J. H., Moultos O. A. (2020). Generalized
form for finite-size corrections in mutual diffusion coefficients
of multicomponent mixtures obtained from equilibrium molecular dynamics
simulation. J. Chem. Theory Comput..

[501] Erdos M., Frangou M., Vlugt T. J. H., Moultos O. A. (2021). Diffusivity
of *α*-, *β*-, *γ*-cyclodextrin and the inclusion complex of *β* -cyclodextrin: Ibuprofen in aqueous solutions; A molecular dynamics
simulation study. Fluid Phase Equilibria.

[502] Jamali S. H., Hartkamp R., Bardas C., Sohl J., Vlugt T. J. H., Moultos O. A. (2018). Shear viscosity
computed from the
finite-size effects of self-diffusivity in equilibrium molecular dynamics. J. Chem. Theory Comput..

[503] Jamali S. H., Wolff L., Becker T. M., Bardow A., Vlugt T. J. H., Moultos O. A. (2018). Finite-size effects of binary mutual
diffusion coefficients from molecular dynamics. J. Chem. Theory Comput..

[504] Fick A. (1855). Über Diffusion. Annalen
der Physik.

[505] Liu X., Schnell S. K., Simon J.-M., Bedeaux D., Kjelstrup S., Bardow A., Vlugt T. J. H. (2011). Fick Diffusion Coefficients of Liquid
Mixtures Directly Obtained from Equilibrium Molecular Dynamics. J. Phys. Chem. B.

[506] Liu X., Martín-Calvo A., McGarrity E., Schnell S. K., Calero S., Simon J.-M., Bedeaux D., Kjelstrup S., Bardow A., Vlugt T. J. H. (2012). Fick
Diffusion
Coefficients in Ternary Liquid Systems from Equilibrium Molecular
Simulations. Ind. Eng. Chem. Res..

[507] Liu X., Schnell S. K., Simon J.-M., Krüger P., Bedeaux D., Kjelstrup S., Bardow A., Vlugt T. J. H. (2013). Diffusion
Coefficients from Molecular Dynamics Simulations in Binary and Ternary
Mixtures. Int. J. Thermophys..

[508] Keffer D. J., Gao C. Y., Edwards B. J. (2005). On the
relationship
between Fickian diffusivities at the continuum and molecular levels. J. Phys. Chem. B.

[509] Wolff L., Jamali S. H., Becker T. M., Moultos O. A., Vlugt T. J. H., Bardow A. (2018). Prediction of Composition-Dependent
Self-Diffusion Coefficients in Binary Liquid Mixtures: The Missing
Link for Darken-Based Models. Industrial &
Engineering Chemistry Research.

[510] Taylor R., Kooijman H. A. (1991). Composition derivatives of activity
coefficient models (for the estimation of thermodynamic factors in
diffusion). Chem. Eng. Commun..

[511] Liu X., Bardow A., Vlugt T. J. H. (2011). Multicomponent
Maxwell-Stefan Diffusivities
at Infinite Dilution. Ind. Eng. Chem. Res..

[512] Maginn E. J., Bell A. T., Theodorou D. N. (1993). Transport
diffusivity of methane in silicalite from equilibrium and nonequilibrium
simulations. J. Phys. Chem..

[513] Tsige M., Grest G. S. (2004). Molecular dynamics
simulation of
solvent-polymer interdiffusion: Fickian diffusion. J. Comput. Phys..

[514] Tsige M., Grest G. S. (2004). Interdiffusion of solvent into glassy
polymer films: A molecular dynamics study. J.
Comput. Phys..

[515] Jamali S. H., Wolff L., Becker T. M., De Groen M., Ramdin M., Hartkamp R., Bardow A., Vlugt T. J. H., Moultos O. A. (2019). OCTP: A
tool for on-the-fly calculation of transport
properties of fluids with the order-n algorithm in LAMMPS. Journal of Chemical Information and Modeling.

[516] Jamali S. H., Wolff L., Becker T. M., Bardow A., Vlugt T. J. H., Moultos O. A. (2018). Finite-Size Effects
of Binary Mutual
Diffusion Coefficients from Molecular Dynamics. Journal of Chemical Theory and Computation.

[517] Bird, R. B. ; Stewart, W. E. ; Lightfoot, E. N. Transport Phenomena, 2nd ed.; John Wiley & Sons, New York, 2007.

[518] Cussler, E. L. Diffusion: Mass transfer in Fluid Systems, 3rd ed.; Cambridge University Press: Cambridge, 2009.

[519] Krishna R., van Baten J. M. (2016). Describing
diffusion in fluid mixtures
at elevated pressures by combining the Maxwell-Stefan formulation
with an equation of state. Chemical Engineering
Science.

[520] Liu X., Schnell S. K., Simon J.-M., Krüger P., Bedeaux D., Kjelstrup S., Bardow A., Vlugt T. J. H. (2013). Diffusion
Coefficients from Molecular Dynamics Simulations in Binary and Ternary
Mixtures. International Journal of Thermophysics.

[521] Liu X., Schnell S. K., Simon J.-M., Bedeaux D., Kjelstrup S., Bardow A., Vlugt T. J. H. (2011). Fick
Diffusion Coefficients of Liquid
Mixtures Directly Obtained From Equilibrium Molecular Dynamics. The Journal of Physical Chemistry B.

[522] Liu X., Martín-Calvo A., McGarrity E., Schnell S. K., Calero S., Simon J.-M., Bedeaux D., Kjelstrup S., Bardow A., Vlugt T. J. H. (2012). Fick
Diffusion
Coefficients in Ternary Liquid Systems from Equilibrium Molecular
Dynamics Simulations. Industrial & Engineering
Chemistry Research.

[523] Liu X., Vlugt T. J. H., Bardow A. (2011). Maxwell-Stefan Diffusivities in Binary
Mixtures of Ionic Liquids with Dimethyl Sulfoxide (DMSO) and H_2_O. The Journal of Physical Chemistry
B.

[524] Liu X., Bardow A., Vlugt T. J. H. (2011). Multicomponent
Maxwell-Stefan Diffusivities
at Infinite Dilution. Industrial & Engineering
Chemistry Research.

[525] Kozlova S., Mialdun A., Ryzhkov I., Janzen T., Vrabec J., Shevtsova V. (2019). Do ternary liquid mixtures exhibit
negative main Fick diffusion coefficients?. Physical Chemistry Chemical Physics.

[526] Su Y., Saric D., Guevara-Carrion G., Zhang Y., He M., Vrabec J. (2025). Fick and Maxwell-Stefan
diffusion of the liquid mixture
cyclohexane + toluene + acetone + methanol and its subsystems. Chemical Engineering Science.

[527] Karger, J. ; Ruthven, M. D. Diffusion in Zeolites and Other Microporous Solids; Wiley: New York, 1992.

[528] Skoulidas A. I., Sholl D. S. (2003). Molecular Dynamics Simulations of
Self-Diffusivities, Corrected Diffusivities, and Transport Diffusivities
of Light Gases in Four Silica Zeolites To Assess Influences of Pore
Shape and Connectivity. The Journal of Physical
Chemistry A.

[529] Gulbalkan H. C., Haslak Z. P., Altintas C., Uzun A., Keskin S. (2022). Assessing CH_4_/N_2_ separation potential
of MOFs, COFs, IL/MOF, MOF/Polymer, and COF/Polymer composites. Chemical Engineering Journal.

[530] Ruiz-Montero M. J., Frenkel D., Brey J. J. (1997). Efficient
schemes
to compute diffusive barrier crossing rates. Molecular Physics.

[531] June R. L., Bell A. T., Theodorou D. N. (1991). Transition-state
studies of xenon and sulfur hexafluoride diffusion in silicalite. The Journal of Physical Chemistry.

[532] Kästner J. (2011). Umbrella sampling. WIREs Computational
Molecular Science.

[533] Verploegh R. J., Nair S., Sholl D. S. (2015). Temperature and
Loading-Dependent Diffusion of Light Hydrocarbons in ZIF-8 as Predicted
Through Fully Flexible Molecular Simulations. Journal of the American Chemical Society.

[534] Voter A. F., Doll J. D. (1985). Dynamical corrections
to transition
state theory for multistate systems: Surface self-diffusion in the
rare-event regime. The Journal of Chemical Physics.

[535] Dubbeldam D., Beerdsen E., Vlugt T. J. H., Smit B. (2005). Molecular
simulation of loading-dependent diffusion in nanoporous materials
using extended dynamically corrected transition state theory. The Journal of Chemical Physics.

[536] Krokidas P., Moncho S., Brothers E. N., Castier M., Jeong H. K., Economou I. G. (2018). On the Efficient Separation of Gas
Mixtures with the Mixed-Linker Zeolitic-Imidazolate Framework-7-8. ACS Applied Materials and Interfaces.

[537] Cheng B., Frenkel D. (2020). Computing the Heat
Conductivity of
Fluids from Density Fluctuations. Phys. Rev.
Lett..

[538] Ding Y., Yu H., Lin M., Ortiz M., Xiao S., He J., Zhang Z. (2024). Hydrogen trapping
and
diffusion in polycrystalline nickel: The spectrum of grain boundary
segregation. Journal of Materials Science &
Technology.

[539] Ito K., Tanaka Y., Tsutsui K., Sawada H. (2023). Analysis of grain-boundary
segregation of hydrogen in bcc-Fe polycrystals via a nano-polycrystalline
grain-boundary model. Computational Materials
Science.

[540] Zhou X., Marchand D., McDowell D. L., Zhu T., Song J. (2016). Chemomechanical Origin of Hydrogen Trapping at Grain
Boundaries in
fcc Metals. Phys. Rev. Lett..

[541] Zhou X., Mousseau N., Song J. (2019). Is Hydrogen
Diffusion
along Grain Boundaries Fast or Slow? Atomistic Origin and Mechanistic
Modeling. Phys. Rev. Lett..

[542] Wang S., Martin M. L., Robertson I. M., Sofronis P. (2016). Effect of hydrogen environment on the separation of
Fe grain boundaries. Acta Materialia.

[543] Jiang D. E., Carter E. A. (2004). Diffusion of interstitial
hydrogen
into and through bcc Fe from first principles. Phys. Rev. B.

[544] Hayward E., Fu C.-C. (2013). Interplay between
hydrogen and vacancies
in *α*-Fe. Phys. Rev. B.

[545] Zhou X. W., El Gabaly F., Stavila V., Allendorf M. D. (2016). Molecular
Dynamics Simulations of Hydrogen Diffusion in Aluminum. The Journal of Physical Chemistry C.

[546] Kimizuka H., Mori H., Ogata S. (2011). Effect of
temperature
on fast hydrogen diffusion in iron: A path-integral quantum dynamics
approach. Phys. Rev. B.

[547] Zhou X., Ouyang B., Curtin W., Song J. (2016). Atomistic
investigation of the influence of hydrogen on dislocation nucleation
during nanoindentation in Ni and Pd. Acta Materialia.

[548] Yin S., Cheng G., Chang T.-H., Richter G., Zhu Y., Gao H. (2019). Hydrogen embrittlement
in metallic nanowires. Nature Communications.

[549] Li J., Gu T., Zhang Y., Chen D. (2025). Dual Role of Hydrogen
Effect on Surface Dislocation Nucleation in Nickel. Journal of Applied Mechanics.

[550] Kapci M. F., Yu P., Marian J., Liu G., Shen Y., Li Y., Bal B. (2024). Edge dislocation depinning
from hydrogen atmosphere in *α*-iron. Scripta Materialia.

[551] Sofronis P., McMeeking R. (1989). Numerical analysis of hydrogen transport
near a blunting crack tip. Journal of the Mechanics
and Physics of Solids.

[552] Yu P., Cui Y., zhen Zhu G., Shen Y., Wen M. (2020). The key role
played by dislocation core radius and energy in hydrogen interaction
with dislocations. Acta Materialia.

[553] Jung S.-P., Kwon Y., Lee C. S., Lee B.-J. (2018). Influence
of hydrogen on the grain boundary crack propagation in bcc iron: A
molecular dynamics simulation. Computational
Materials Science.

[554] Marx D. (2006). Proton Transfer 200 Years after von
Grotthuss: Insights from Ab Initio
Simulations. ChemPhysChem.

[555] Hassanali A., Prakash M. K., Eshet H., Parrinello M. (2011). On the Recombination
of Hydronium and Hydroxide Ions in Water. Proceedings
of the National Academy of Sciences.

[556] Jiao K., Li X. (2011). Water transport in
polymer electrolyte
membrane fuel cells. Progress in Energy and
Combustion Science.

[557] Ise M., Kreuer K., Maier J. (1999). Electroosmotic drag
in polymer electrolyte
membranes: an electrophoretic NMR study. Solid
State Ionics.

[558] Dickinson E. J. F., Smith G. (2020). Modelling the Proton-Conductive Membrane
in Practical Polymer Electrolyte Membrane Fuel Cell (PEMFC) Simulation:
A Review. Membranes.

[559] Zawodzinski T. A., Davey J., Valerio J., Gottesfeld S. (1995). The water
content dependence of electro-osmotic drag in proton-conducting polymer
electrolytes. Electrochim. Acta..

[560] Pivovar B. S. (2006). An overview of electro-osmosis in
fuel cell polymer
electrolytes. Polymer.

[561] Kirkwood J. G., Buff F. P. (1951). The statistical
mechanical theory
of solutions. I. J. Chem. Phys..

[562] Schnell S. K., Vlugt T. J. H., Simon J.-M., Bedeaux D., Kjelstrup S. (2012). Thermodynamics of small systems embedded
in a reservoir:
a detailed analysis of finite size effects. Mol. Phys..

[563] Schnell S. K., Liu X., Simon J.-M., Bardow A., Bedeaux D., Vlugt T. J. H., Kjelstrup S. (2011). Calculating
Thermodynamic Properties from Fluctuations at Small Scales. J. Phys. Chem. B.

[564] Schnell S. K., Vlugt T. J. H., Simon J.-M., Bedeaux D., Kjelstrup S. (2011). Thermodynamics
of a small system in a *μ*T reservoir. Chem. Phys. Lett..

[565] Wedberg R., O’Connell J. P., Peters G. H., Abildskov J. (2010). Accurate Kirkwood-Buff
integrals from molecular simulations. Mol. Simul..

[566] Wedberg R., O’Connell J. P., Peters G. H., Abildskov J. (2011). Total and
direct correlation function integrals from molecular simulation of
binary systems. Fluid Phase Equilib..

[567] Krüger P., Schnell S. K., Bedeaux D., Kjelstrup S., Vlugt T. J. H., Simon J.-M. (2013). Kirkwood-Buff Integrals
for Finite
Volumes. J. Phys. Chem. Lett..

[568] Simon J.-M., Krüger P., Schnell S. K., Vlugt T. J. H., Kjelstrup S., Bedeaux D. (2022). Kirkwood-Buff Integrals: From fluctuations
in finite volumes to the thermodynamic limit. J. Comput. Phys..

[569] Dawass N., Krüger P., Schnell S. K., Moultos O. A., Economou I. G., Vlugt T. J. H., Simon J.-M. (2020). Kirkwood-Buff Integrals
Using Molecular Simulation: Estimation of Surface Effects. Nanomaterials.

[570] Dawass N., Krüger P., Schnell S. K., Simon J.-M., Vlugt T. J. H. (2019). Kirkwood-Buff
integrals from molecular simulation. Fluid Phase
Equilib..

[571] Schnell S. K., Englebienne P., Simon J.-M., Krüger P., Balaji S. P., Kjelstrup S., Bedeaux D., Bardow A., Vlugt T. J. H. (2013). How to apply the Kirkwood-Buff theory to individual
species in salt solutions. Chem. Phys. Lett..

[572] Balaji S. P., Schnell S. K., McGarrity E. S., Vlugt T. J. H. (2013). A direct method for calculating thermodynamic factors
for liquid mixtures using the Permuted Widom Test Particle Insertion
Method. Mol. Phys..

[573] Balaji S. P., Schnell S. K., Vlugt T. J. H. (2013). Calculating
thermodynamic
factors of ternary and multicomponent mixtures using the Permuted
Widom Test Particle Insertion Method. Theor.
Chem. Acc..

[574] Ben-Naim, A. Molecular Theory of Solutions; Oxford University Press, 2006.

[575] Widom B. (1963). Some topics in the theory of fluids. J. Comput.
Phys..

[576] Hulikal Chakrapani T., Hajibeygi H., Moultos O. A., Vlugt T. J. H. (2024). Calculating
Thermodynamic Factors for Diffusion Using the Continuous Fractional
Component Monte Carlo Method. Journal of Chemical
Theory and Computation.

[577] Ladd A., Woodcock L. (1977). Triple-point coexistence properties
of the Lennard-Jones system. Chemical Physics
Letters.

[578] Michalis V. K., Costandy J., Tsimpanogiannis I. N., Stubos A. K., Economou I. G. (2015). Prediction
of the phase equilibria
of methane hydrates using the direct phase coexistence methodology. The Journal of Chemical Physics.

[579] Costandy J., Michalis V. K., Tsimpanogiannis I. N., Stubos A. K., Economou I. G. (2015). The role
of intermolecular interactions
in the prediction of the phase equilibria of carbon dioxide hydrates. The Journal of Chemical Physics.

[580] Michalis V. K., Tsimpanogiannis I. N., Stubos A. K., Economou I. G. (2016). Direct
phase coexistence molecular dynamics study of the phase equilibria
of the ternary methane-carbon dioxide-water hydrate system. Physical Chemistry Chemical Physics.

[581] Mohr S., Pétuya R., Wylde J., Sarria J., Purkayastha N., Ward Z., Bodnar S., Tsimpanogiannis I. N. (2021). Size dependence
of the dissociation process of spherical hydrate particles via microsecond
molecular dynamics simulations. Physical Chemistry
Chemical Physics.

[582] Mak T. C., McMullan R. K. (1965). Polyhedral clathrate hydrates. X.
Structure of the double hydrate of tetrahydrofuran and hydrogen sulfide. The Journal of Chemical Physics.

[583] McMullan R. K., Jeffrey G. (1965). Polyhedral clathrate
hydrates. IX.
Structure of ethylene oxide hydrate. The Journal
of Chemical Physics.

[584] Bernal J. D., Fowler R. H. (1933). A theory of water and ionic solution,
with particular reference to hydrogen and hydroxyl ions. The Journal of Chemical Physics.

[585] Sarupria S., Debenedetti P. G. (2011). Molecular
dynamics study of carbon
dioxide hydrate dissociation. The Journal of
Physical Chemistry A.

[586] Takeuchi F., Hiratsuka M., Ohmura R., Alavi S., Sum A. K., Yasuoka K. (2013). Water proton configurations in structures
I, II, and H clathrate hydrate unit cells. The
Journal of Chemical Physics.

[587] English N.
J., Lauricella M., Meloni S. (2014). Massively parallel
molecular dynamics simulation of formation of clathrate-hydrate precursors
at planar water-methane interfaces: Insights into heterogeneous nucleation. The Journal of Chemical Physics.

[588] Blazquez S., M Conde M., Vega C., Sanz E. (2023). Growth rate
of CO_2_ and CH_4_ hydrates by means of molecular
dynamics simulations. The Journal of Chemical
Physics.

[589] Vatamanu J., Kusalik P. G. (2008). Heterogeneous crystal
growth of methane
hydrate on its sII [001] crystallographic face. The Journal of Physical Chemistry B.

[590] Yagasaki T., Matsumoto M., Tanaka H. (2016). Mechanism of slow crystal
growth of tetrahydrofuran clathrate hydrate. The Journal of Physical Chemistry C.

[591] Báez L. A., Clancy P. (1994). Computer simulation
of the crystal
growth and dissolution of natural gas hydrates. Annals of the New York Academy of Sciences.

[592] Fidler J., Rodger P. (1999). Solvation structure
around aqueous
alcohols. The Journal of Physical Chemistry
B.

[593] Rodger P., Forester T., Smith W. (1996). Simulations
of the
methane hydrate/methane gas interface near hydrate forming conditions
conditions. Fluid Phase Equilibria.

[594] Zhang Z., Kusalik P. G., Guo G.-J. (2018). Molecular
insight
into the growth of hydrogen and methane binary hydrates. The Journal of Physical Chemistry C.

[595] Tian H., Zhang Z. (2020). Revealing the growth
of H_2_ + THF binary hydrate through molecular simulations. Energy & Fuels.

[596] Guo G.-J., Zhang Y.-G., Liu C.-J., Li K.-H. (2011). Using the
face-saturated incomplete cage analysis to quantify the cage compositions
and cage linking structures of amorphous phase hydrates. Physical Chemistry Chemical Physics.

[597] Ghoufi A., Malfreyt P., Tildesley D. J. (2016). Computer
modelling of the surface tension of the gas-liquid and liquid-liquid
interface. Chem. Soc. Rev..

[598] Irving J., Kirkwood J. G. (1950). The statistical
mechanical theory
of transport processes. IV. The equations of hydrodynamics. J. Chem. Phys..

[599] Hill T. L. (1952). Statistical
thermodynamics of the transition region
between two phases. I. Thermodynamics and quasi-thermodynamics. J. Phys. Chem..

[600] Kirkwood J. G., Buff F. P. (1949). The statistical mechanical theory
of surface tension. J. Chem. Phys..

[601] Javanbakht G., Sedghi M., Welch W., Goual L. (2015). Molecular
dynamics simulations of CO_2_/water/quartz interfacial properties:
impact of CO_2_ dissolution in water. Langmuir.

[602] Yang Y., Nair A. K. N., Zhu W., Sang S., Sun S. (2023). Molecular
Perspectives of Interfacial Properties of the Hydrogen+
Water Mixture in Contact with Silica or Kerogen. J. Mol. Liq..

[603] Abdel-Azeim S., Al-Yaseri A., Norrman K., Patil P., Qasim A., Yousef A. (2023). Wettability
of Caprock-H_2_-Water: Insights from Molecular Dynamic Simulations
and Sessile-Drop
Experiment. Energy & Fuels.

[604] Chen C., Xia J., Bahai H. (2023). Effect of
the Temperature
on Interfacial Properties of CO_2_/H_2_ Mixtures
Contacting with Brine and Hydrophilic Silica by Molecular Dynamics
Simulations. Energy & Fuels.

[605] Chen C., Xia J. (2024). A comparative study
on transport
and interfacial physics of H_2_/CO_2_/CH_4_ interacting with H_2_O and/or silica by molecular dynamics
simulation. Phys. Fluids.

[606] Al-Yaseri A., Esteban L., Giwelli A., Abdel-Azeim S., Sarout J., Sarmadivaleh M. (2023). Impact of wettability on storage
and recovery of hydrogen gas in the lesueur sandstone formation (Southwest
hub project, Western Australia). Int. J. Hydrogen
Energy.

[607] Al-Yaseri A., Abdel-Azeim S., Al-Hamad J. (2023). Wettability of water-H_2_-quartz and water-H_2_-calcite experiment and molecular
dynamics simulations: Critical assessment. Int.
J. Hydrogen Energy.

[608] Zheng R., Germann T. C., Huang L., Mehana M. (2024). Driving mechanisms
of quartz wettability alteration under in-situ H2 geo-storage conditions:
Role of organic ligands and surface morphology. International Journal of Hydrogen Energy.

[609] Zheng R., Germann T. C., Gross M., Mehana M. (2024). Molecular
insights into the impact of surface chemistry and pressure on quartz
wettability: resolving discrepancies for hydrogen geo-storage. ACS Sustainable Chemistry & Engineering.

[610] Martin M. G. (2013). MCCCS Towhee: a tool for Monte Carlo
molecular simulation. Molecular Simulation.

[611] Hens R., Rahbari A., Caro-Ortiz S., Dawass N., Erdos M., Poursaeidesfahani A., Salehi H. S., Celebi A. T., Ramdin M., Moultos O. A., Dubbeldam D., Vlugt T. J. H. (2020). Brick-CFCMC: Open Source Software
for Monte Carlo Simulations of Phase and Reaction Equilibria Using
the Continuous Fractional Component Method. J. Chem. Inf. Model..

[612] Polat H. M., Salehi H. S., Hens R., Wasik D. O., Rahbari A., de Meyer F., Houriez C., Coquelet C., Calero S., Dubbeldam D., Moultos O. A., Vlugt T. J. H. (2021). New
Features of the Open Source Monte Carlo Software Brick-CFCMC: Thermodynamic
Integration and Hybrid Trial Moves. J. Chem.
Inf. Model..

[613] Dubbeldam D., Torres-Knoop A., Walton K. S. (2013). On the inner workings
of Monte Carlo codes. Mol. Simul..

[614] Binder, K. Monte Carlo Methods in Statistical Physics; Springer-Verlag: Berlin, 1986.

[615] Fichthorn K. A., Weinberg W. H. (1991). Theoretical foundations of dynamical
Monte Carlo simulations. The Journal of Chemical
Physics.

[616] Panagiotopoulos A. Z. (1987). Direct
determination of phase coexistence properties
of fluids by Monte Carlo simulation in a new ensemble. Mol. Phys..

[617] Widom B. (1963). Some topics in the theory of fluids. The Journal
of Chemical Physics.

[618] Rahbari A., Hens R., Ramdin M., Moultos O. A., Dubbeldam D., Vlugt T. J. H. (2021). Recent advances in the continuous
fractional component Monte Carlo methodology. Mol. Simul..

[619] Sandler, S. I. An Introduction to Applied Statistical Thermodynamics; John Wiley & Sons: Hoboken, New Jersey, 2011.

[620] Widom B. (1982). Potential-distribution theory and
the statistical mechanics of fluids. J. Phys.
Chem..

[621] Boulougouris G. C., Economou I. G., Theodorou D. N. (2001). Calculation
of the chemical potential of chain molecules using the staged particle
deletion scheme. J. Chem. Phys..

[622] Boulougouris G. C., Economou I. G., Theodorou D. N. (1999). On the
calculation of the chemical potential using the particle deletion
scheme. Mol. Phys..

[623] Coskuner O., Deiters U. K. (2006). Hydrophobic interactions
by Monte
Carlo simulations. Zeitschrift für Phys.
Chem..

[624] Rahbari A., Poursaeidesfahani A., Torres-Knoop A., Dubbeldam D., Vlugt T. J. H. (2018). Chemical potentials of water, methanol,
carbon dioxide and hydrogen sulphide at low temperatures using Continuous
Fractional Component Gibbs ensemble Monte Carlo. Mol. Simul..

[625] Lu N., Singh J. K., Kofke D. A. (2003). Appropriate methods to combine forward
and reverse free-energy perturbation averages. J. Chem. Phys..

[626] Shi W., Maginn E. J. (2007). Continuous Fractional Component Monte Carlo: an adaptive
biasing method for open system atomistic simulations. J. Chem. Theory Comput..

[627] Nezbeda I., Kolafa J. (1991). A new version of the insertion particle
method for determining the chemical Potential by Monte Carlo simulation. Mol. Simul..

[628] Escobedo F. A., de Pablo J. J. (1996). Expanded grand canonical and Gibbs
ensemble Monte Carlo simulation of polymers. J. Chem. Phys..

[629] Shi W., Maginn E. J. (2007). Continuous Fractional Component Monte Carlo: An adaptive
biasing method for open system atomistic simulations. J. Chem. Theory Comput..

[630] van
Rooijen W. A., Habibi P., Xu K., Dey P., Vlugt T. J. H., Hajibeygi H., Moultos O. A. (2024). Interfacial Tensions,
Solubilities, and Transport Properties of the H_2_/H_2_O/NaCl System: A Molecular Simulation Study. Journal of Chemical & Engineering Data.

[631] Wang F., Landau D. P. (2001). Efficient, multiple-range
random
walk algorithm to calculate the density of states. Phys. Rev. Lett..

[632] Rahbari A., Hens R., Dubbeldam D., Vlugt T. J. H. (2019). Improving the accuracy of computing chemical potentials
in CFCMC simulations. Mol. Phys..

[633] Brumby P. E., Yuhara D., Hasegawa T., Wu D. T., Sum A. K., Yasuoka K. (2019). Cage occupancies, lattice
constants,
and guest chemical potentials for structure II hydrogen clathrate
hydrate from Gibbs ensemble Monte Carlo simulations. The Journal of Chemical Physics.

[634] Papadimitriou N., Tsimpanogiannis I., Papaioannou A. T., Stubos A. (2008). Evaluation of the hydrogen-storage
capacity of pure
H_2_ and binary H_2_-THF hydrates with Monte Carlo
simulations. The Journal of Physical Chemistry
C.

[635] Papadimitriou N., Tsimpanogiannis I., Peters C., Papaioannou A. T., Stubos A. (2008). Hydrogen storage in
sH hydrates: A Monte Carlo study. The Journal
of Physical Chemistry B.

[636] Papadimitriou N., Tsimpanogiannis I., Stubos A. (2010). Monte Carlo study of
sI hydrogen hydrates. Molecular Simulation.

[637] Tsimpanogiannis I. N., Papadimitriou N. I., Stubos A. K. (2012). On the limitation
of the van der Waals-Platteeuw-based thermodynamic models for hydrates
with multiple occupancy of cavities. Molecular
Physics.

[638] Papadimitriou N. I., Tsimpanogiannis I. N., Papaioannou A. T., Stubos A. K. (2008). Monte Carlo study
of sII and sH argon hydrates with
multiple occupancy of cages. Molecular Simulation.

[639] Papadimitriou N. I., Tsimpanogiannis I. N., Economou I. G., Stubos A. K. (2014). Influence
of combining rules on the cavity occupancy of clathrate hydrates by
Monte Carlo simulations. Molecular Physics.

[640] Papadimitriou N., Tsimpanogiannis I., Economou I., Stubos A. (2015). Evaluation
of the efficiency of clathrate hydrates in storing energy gases. Journal of Physics: Conference Series.

[641] Papadimitriou N. I., Tsimpanogiannis I. N., Economou I. G., Stubos A. K. (2016). Storage
of methane in clathrate hydrates: Monte Carlo simulations of sI hydrates
and comparison with experimental measurements. Journal of Chemical & Engineering Data.

[642] Sandler, S. I. Chemical, biochemical, and engineering thermodynamics, 4th ed.; John Wiley & Sons: Hoboken, N.J., USA, 2006.

[643] Moran, M. J. ; Shapiro, H. N. Fundamentals of Engineering thermodynamics, 5th ed.; John Wiley & Sons: West Sussex, England, 2006.

[644] Jorgensen W. L. (1986). Optimized
intermolecular potential functions for liquid
alcohols. J. Phys. Chem..

[645] Lagache M., Ungerer P., Boutin A., Fuchs A. H. (2001). Prediction
of thermodynamic derivative properties of fluids by Monte Carlo simulation. Phys. Chem. Chem. Phys..

[646] Reid, R. C. ; Prausnitz, J. M. ; Poling, B. E. The Properties of Gases and Liquids, 5th ed.; McGraw-Hill: New York, 2001.

[647] Johnson, R. D., III 2019; NIST Computational Chemistry Comparison and Benchmark Database. NIST Standard Reference Database Number 101 Release 20, Editor: Johnson, R. D., III http://cccbdb.nist.gov/.

[648] Rahbari A., Josephson T. R., Sun Y., Moultos O. A., Dubbeldam D., Siepmann J. I., Vlugt T. J. H. (2020). Multiple Linear
Regression and Thermodynamic Fluctuations are Equivalent for Computing
Thermodynamic Derivatives from Molecular Simulation. Fluid Phase Equilib..

[649] Voter A. F. (2007). INTRODUCTION
TO THE KINETIC MONTE CARLO METHOD. Radiation
Effects in Solids. Dordrecht.

[650] Andersen, M. ; Panosetti, C. ; Reuter, K. A Practical Guide to Surface Kinetic Monte Carlo Simulations. Frontiers in Chemistry 2019, 7, 10.3389/fchem.2019.00202. PMC6465329 31024891

[651] Henkelman G., Uberuaga B. P., Jonsson H. (2000). A climbing
image nudged
elastic band method for finding saddle points and minimum energy paths. The Journal of Chemical Physics.

[652] Vineyard G. H. (1957). Frequency factors and isotope effects
in solid state
rate processes. Journal of Physics and Chemistry
of Solids.

[653] Zhou X., Ou P., Mousseau N., Song J. (2024). Critical assessment
of hydrogen pipe diffusion at dislocations in metals. Acta Materialia.

[654] Zhou X.-Y., Zhu J.-H., Wu Y., Yang X.-S., Lookman T., Wu H.-H. (2022). Machine learning assisted design
of FeCoNiCrMn high-entropy alloys with ultra-low hydrogen diffusion
coefficients. Acta Materialia.

[655] Zhang J., Clennell M. B., Sagotra A., Pascual R. (2023). Molecular
dynamics simulation and machine learning for predicting hydrogen solubility
in water: Effects of temperature, pressure, finite system size and
choice of molecular force fields. Chemical Physics.

[656] Chabab S., Théveneau P., Coquelet C., Corvisier J., Paricaud P. (2020). Measurements and predictive
models of high-pressure
H_2_ solubility in brine (H_2_O+NaCl) for underground
hydrogen storage application. Int. J. Hydrogen
Energy.

[657] Hydrogen in Water and Aqueous Electrolyte Solutions; Haynes, W. M. , Ed.; Pergamon Press, 1981; Vol. 5/6; IUPAC Solubility Data Series.

[658] Alvarez J., Crovetto R., Fernández-Prini R. (1988). The Dissolution
of N_2_ and of H_2_ in Water from Room Temperature
to 640 K. Berichte der Bunsengesellschaft für
physikalische Chemie.

[659] Sabo D., Rempe S. B., Greathouse J. A., Martin M. G. (2006). Molecular studies of the structural properties of hydrogen
gas in bulk water. Molecular Simulation.

[660] Sabo D., Varma S., Martin M. G., Rempe S. B. (2008). Studies
of the Thermodynamic Properties of Hydrogen Gas in Bulk Water. The Journal of Physical Chemistry B.

[661] Buch V., Devlin J. P. (1993). Preferential adsorption
of ortho-H_2_ with respect to para-H_2_ on the amorphous
ice surface. J. Chem. Phys..

[662] Urukova I., Vorholz J., Maurer G. (2005). Solubility
of CO_2_, CO, and H_2_ in the Ionic Liquid [bmim]­[PF6]
from
Monte Carlo Simulations. J. Phys. Chem. B.

[663] Salehi H. S., Hens R., Moultos O. A., Vlugt T. J. H. (2020). Computation
of gas solubilities in choline chloride urea and choline chloride
ethylene glycol deep eutectic solvents using Monte Carlo simulations. J. Mol. Liq..

[664] Liu X., Bara J. E., Turner C. H. (2021). Understanding Gas Solubility of Pure
Component and Binary Mixtures within Multivalent Ionic Liquids from
Molecular Simulations. J. Phys. Chem. B.

[665] Steele W. A. (1973). The physical interaction of gases
with crystalline
solids: I. Gas-solid energies and properties of isolated adsorbed
atoms. Surface Science.

[666] Tjatjopoulos G. J., Feke D. L., Mann J. A. J. (1988). Molecule-micropore
interaction potentials. J. Phys. Chem..

[667] Siderius D. W., Gelb L. D. (2011). Extension of the
Steele 10-4-3 potential
for adsorption calculations in cylindrical, spherical, and other pore
geometries. J. Chem. Phys..

[668] Hendriks E., Kontogeorgis G. M., Dohrn R., de Hemptinne J.-C., Economou I. G., Zilnik L. F., Vesovic V. (2010). Industrial Requirements
for Thermodynamics and Transport Properties. Industrial & Engineering Chemistry Research.

[669] de Hemptinne J.-C., Kontogeorgis G. M., Dohrn R., Economou I. G., ten Kate A., Kuitunen S., Fele Zilnik L., De Angelis M. G., Vesovic V. (2022). A View on the Future
of Applied Thermodynamics. Industrial &
Engineering Chemistry Research.

[670] Naseri Boroujeni S., Maribo-Mogensen B., Liang X., Kontogeorgis G. M. (2024). Theoretical
and practical investigation of ion-ion association in electrolyte
solutions. The Journal of Chemical Physics.

[671] Novak N., Yang F., Olsen M. D., Liang X., von Solms N., Economou I. G., Castier M., de Hemptinne J.-C., Panagiotopoulos A. Z., Kontogeorgis G. M. (2025). Contributions
to ionic activity coefficients:
A review and comparison of equations of state with molecular simulations. Fluid Phase Equilibria.

[672] Kerkache H., Hoang H., Cézac P., Galliéro G., Chabab S. (2024). The solubility of H_2_ in
NaCl brine at high pressures and high temperatures: Molecular simulation
study and thermodynamic modeling. Journal of
Molecular Liquids.

[673] Weisenberger S., Schumpe A. (1996). Estimation of gas solubilities in
salt solutions at temperatures from 273 K to 363 K. AIChE J..

[674] Torín-Ollarves G. A., Trusler J. M. (2021). Solubility of hydrogen
in sodium chloride brine at high pressures. Fluid Phase Equilib..

[675] Lopez-Lazaro C., Bachaud P., Moretti I., Ferrando N. (2019). Predicting
the phase behavior of hydrogen in NaCl brines by Molecular Simulation
for geological applications. BSGF-Earth Sci.
Bulletin.

[676] Zhang J., Clennell M. B., Dewhurst D. N. (2023). Transport
Properties
of NaCl in Aqueous Solution and Hydrogen Solubility in Brine. The Journal of Physical Chemistry B.

[677] Hulikal Chakrapani T., Hajibeygi H., Moultos O. A., Vlugt T. J. H. (2024). Mutual
Diffusivities of Mixtures of Carbon Dioxide and Hydrogen and Their
Solubilities in Brine: Insight from Molecular Simulations. Industrial & Engineering Chemistry Research.

[678] Potoff J. J., Siepmann J. I. (2001). Vapor-liquid equilibria
of mixtures
containing alkanes, carbon dioxide, and nitrogen. AIChE Journal.

[679] Setschenow J. (1889). Über die konstitution der salzlösungen
auf grund ihres verhaltens zu kohlensäure. Zeitschrift für Physikalische Chemie.

[680] Postma J. R. T., Habibi P., Dey P., Vlugt T. J. H., Moultos O. A., Padding J. T. (2025). Densities, Viscosities,
and Self-Diffusion
Coefficients of Aqueous Mixtures of NaBH_4_, NaB­(OH)_4_, and NaOH Using the BH_4_
^‑^ Delft Force Field (DFF/BH_4_
^‑^). Journal of Chemical & Engineering Data.

[681] Maslennikova, V. Y. ; Goryunova, N. ; Subbotina, L. ; Tsiklis, D. The solubility of water in compressed hydrogen. Russian Journal of Physical Chemistry 1976, 50, 240–243.

[682] Bartlett E. P. (1927). The concentration of water vapor in compressed hydrogen,
Nitrogen and a mixture of these gases in the presence of condensed
water. J. Amer. Chem. Soc..

[683] Wiebe R., Gaddy V. L. (1934). The Solubility of
Hydrogen in Water
at 0, 50, 75 and 100° from 25 to 1000 Atmospheres. J. Am. Chem. Soc..

[684] Rouha M., Nezbeda I., Hruby J., Moučka F. (2018). Higher Virial
Coefficients of Water. J. Mol. Liq..

[685] Kunz O., Wagner W. (2012). The GERG-2008 Wide-Range
Equation
of State for Natural Gases and Other Mixtures: An Expansion of GERG-2004. Journal of Chemical & Engineering Data.

[686] Yang X., Xu J., Wu S., Yu M., Hu B., Cao B., Li J. (2018). A molecular dynamics
simulation study
of PVT properties for H_2_O/H_2_/CO_2_ mixtures
in near-critical and supercritical regions of water. International Journal of Hydrogen Energy.

[687] Zhao X., Jin H. (2019). Investigation of hydrogen
diffusion
in supercritical water: A molecular dynamics simulation study. Int. J. Heat Mass Transfer.

[688] Tsimpanogiannis I. N., Maity S., Celebi A. T., Moultos O. A. (2021). Engineering
Model for Predicting the Intradiffusion Coefficients of Hydrogen and
Oxygen in Vapor, Liquid, and Supercritical Water based on Molecular
Dynamics Simulations. Journal of Chemical &
Engineering Data.

[689] Polat H. M., Coelho F. M., Vlugt T. J. H., Mercier
Franco L. F., Tsimpanogiannis I. N., Moultos O. A. (2024). Diffusivity of CO_2_ in H_2_O: A Review of Experimental Studies and Molecular
Simulations in the Bulk and in Confinement. Journal of Chemical & Engineering Data.

[690] Wang S., Zhou T., Pan Z., Trusler J. P. M. (2023). Diffusion
Coefficients of N_2_O and H_2_ in Water at Temperatures
between 298.15 and 423.15 K with Pressures up to 30 MPa. Journal of Chemical & Engineering Data.

[691] Kallikragas D. T., Plugatyr A. Y., Svishchev I. M. (2014). High temperature
diffusion coefficients for O_2_, H_2_, and OH in
water, and for pure water. J. Chem. Eng. Data.

[692] Abascal J. L., Vega C. (2005). A general purpose model
for the condensed
phases of water: TIP4P/2005. J. Chem. Phys..

[693] Vega C. (2015). Water: one molecule, two surfaces,
one mistake. Mol. Phys..

[694] Moultos O. A., Tsimpanogiannis I. N., Panagiotopoulos A. Z., Economou I. G. (2014). Atomistic Molecular Dynamics Simulations
of CO_2_ Diffusivity in H_2_O for a Wide Range of
Temperatures
and Pressures. The Journal of Physical Chemistry
B.

[695] Zhang C., Lu C., Jing Z., Wu C., Piquemal J.-P., Ponder J. W., Ren P. (2018). AMOEBA Polarizable
Atomic Multipole Force Field for Nucleic Acids. Journal of Chemical Theory and Computation.

[696] Moultos O. A., Tsimpanogiannis I. N. (2023). Predictive
model for the intra-diffusion
coefficients of H_2_ and O_2_ in vapour H_2_O based on data from molecular dynamics simulations. Molecular Physics.

[697] Tsimpanogiannis I. N., Jamali S. H., Economou I. G., Vlugt T. J. H., Moultos O. A. (2020). On the validity of the Stokes-Einstein
relation for
various water force fields. Molecular Physics.

[698] Tsimpanogiannis I. N., Moultos O. A. (2023). Is Stokes-Einstein
relation valid
for the description of intra-diffusivity of hydrogen and oxygen in
liquid water?. Fluid Phase Equilibria.

[699] Kerkache H., Hoang H., Nguyen T. K. N., Geoffroy-Neveux A., Nieto-Draghi C., Cézac P., Chabab S., Galliéro G. (2025). Assessment
of H_2_ diffusivity in water and brine for underground storage:
A molecular dynamics approach. International
Journal of Hydrogen Energy.

[700] Blazquez S., Abascal J. L. F., Lagerweij J., Habibi P., Dey P., Vlugt T. J. H., Moultos O. A., Vega C. (2023). Computation of Electrical Conductivities of Aqueous Electrolyte Solutions:
Two Surfaces, One Property. Journal of Chemical
Theory and Computation.

[701] Avula N. V. S., Klein M. L., Balasubramanian S. (2023). Understanding
the Anomalous Diffusion of Water in Aqueous Electrolytes Using Machine
Learned Potentials. The Journal of Physical
Chemistry Letters.

[702] Zhu S., Robinson G. W. (1991). Structure and dynamics of liquid
water between plates. J. Comput. Phys..

[703] Yan L., Zhu S., Ji X., Lu W. (2007). Proton Hopping in Phosphoric
Acid Solvated Nafion Membrane: A Molecular Simulation Study. J. Phys. Chem. B.

[704] Springer T. E., Zawodzinski T. A., Gottesfeld S. (1991). Polymer Electrolyte
Fuel Cell Model. J. Electrochem. Soc..

[705] Sahu P., Ali S. M. (2025). Electric Field Assisted
Migration
of H^+^ and Cu^+^/Cu^2+^ Ions through the
Nafion in Cu-Cl Electrolyzer for Hydrogen Generation: Molecular Dynamics
Simulations. Journal of Electroanalytical Chemistry.

[706] Lasala S., Samukov K., Mert Polat H., Lachet V., Herbinet O., Privat R., Jaubert J.-N., Moultos O. A., De Ras K., Vlugt T. J. H. (2024). Application of
thermodynamics at different scales to describe the behaviour of fast
reacting binary mixtures in vapour-liquid equilibrium. Chemical Engineering Journal.

[707] van Duin A. C. T., Dasgupta S., Lorant F., Goddard W. A. (2001). ReaxFF:
A Reactive Force Field for Hydrocarbons. The
Journal of Physical Chemistry A.

[708] Senftle T. P., Hong S., Islam M. M., Kylasa S. B., Zheng Y., Shin Y. K., Junkermeier C., Engel-Herbert R., Janik M. J., Aktulga H. M., Verstraelen T., Grama A., van Duin A. C. T. (2016). The ReaxFF reactive force-field:
development, applications and future directions. npj Computational Materials.

[709] Brenner D. W., Shenderova O. A., Harrison J. A., Stuart S. J., Ni B., Sinnott S. B. (2002). A second-generation reactive empirical bondorder (REBO)
potential energy expression for hydrocarbons. Journal of Physics: Condensed Matter.

[710] Polat H. M., Lasala S., de Meyer F., Houriez C., Moultos O. A., Vlugt T. J. H. (2024). Scaling towards the critical point
in the combined reaction/Gibbs ensemble. Fluid
Phase Equilibria.

[711] Stuart S. J., Tutein A. B., Harrison J. A. (2000). A reactive potential
for hydrocarbons with intermolecular interactions. The Journal of Chemical Physics.

[712] Xiao, J. ; Liu, K. ; Du, Y. ; Jin, Z. ; Lu, H. Measurement on and Correlation of VLE of H_2_-N_2_-Ar System. Chemical Engineering (China) 1990, 18, 8–12.

[713] Streett W., Calado J. (1978). Liquid-vapour equilibrium for hydrogen+
nitrogen at temperatures from 63 to 110 K and pressures to 57 MPa. The Journal of Chemical Thermodynamics.

[714] Calado J., Streett W. (1979). Liquid—-vapor
equilibrium
in the system H_2_ Ar at temperatures from 83 to 141 K and
pressures to 52 MPa. Fluid Phase Equilibria.

[715] Pierre J., Walker H.-W. Y., Riedemann A. (2022). Clapeyron.jl:
An Extensible, Open-Source Fluid Thermodynamics Toolkit. Ind. Eng. Chem. Res..

[716] Raju D., Ramdin M., Vlugt T. J. H. (2024). Thermophysical
Properties and Phase Behavior of CO_2_ with Impurities: Insight
from Molecular Simulations. Journal of Chemical
& Engineering Data.

[717] Tsang C., Street W. (1981). Phase equilibria in the H_2_/CO_2_ system at temperatures from 220 to 290 K and pressures
to 172 MPa. Chemical Engineering Science.

[718] Zhang J., Clennell M. B., Chen Y. (2024). New analytical
thermodynamic
models developed for pure H_2_, CH_4_, CO_2_ and H_2_ containing mixtures based on molecular simulations. International Journal of Hydrogen Energy.

[719] Hernández-Gomez R., Tuma D., Pérez E., Chamorro C. R. (2018). Accurate Experimental (p, *ρ*,
and T) Data for the Introduction of Hydrogen into the Natural Gas
Grid (II): Thermodynamic Characterization of the Methane-Hydrogen
Binary System from 240 to 350 K and Pressures up to 20 MPa. Journal of Chemical & Engineering Data.

[720] Cheng S., Shang F., Ma W., Jin H., Sakoda N., Zhang X., Guo L. (2019). Density Data of Two
(H_2_ + CO_2_) Mixtures and a (H_2_ + CO_2_ + CH_4_) Mixture by a Modified Burnett Method at
Temperature 673 K and Pressures up to 25 MPa. Journal of Chemical & Engineering Data.

[721] Chen B., Potoff J. J., Siepmann J. I. (2001). Monte Carlo
calculations
for alcohols and their mixtures with alkanes. Transferable potentials
for phase equilibria. 5. united-atom description of primary, secondary,
and tertiary alcohols. J. Phys. Chem. B.

[722] Makrodimitri Z. A., Unruh D. J. M., Economou I. G. (2011). Molecular
Simulation
of Diffusion of Hydrogen, Carbon Monoxide, and Water in Heavy n-Alkanes. Journal of Physical Chemistry B.

[723] Giraudet C., Klein T., Zhao G., Rausch M. H., Koller T. M., Fröba A. P. (2018). Thermal,
Mutual, and Self-Diffusivities
of Binary Liquid Mixtures Consisting of Gases Dissolved in n-Alkanes
at Infinite Dilution. The Journal of Physical
Chemistry B.

[724] Wilke C. R., Chang P. (1955). Correlation of diffusion coefficients
in dilute solutions. AIChE Journal.

[725] Wu W., Klein T., Kerscher M., Rausch M. H., Koller T. M., Giraudet C., Fröba A. P. (2019). Diffusivities
in 1-Alcohols Containing
Dissolved H_2_, He, N_2_, CO, or CO_2_ Close
to Infinite Dilution. The Journal of Physical
Chemistry B.

[726] Rodden J. B., Erkey C., Akgerman A. (1988). Diffusion Coefficients
for Binary Supercritical Mixtures of n-Alcohols in Carbon Dioxide. Journal of Chemical Engineering Data.

[727] Matthews M. A., Rodden J. B., Akgerman A. (1987). High-Temperature
Diffusion,
Viscosity, and Density Measurements in n-Hexadecane. Journal of Chemical Engineering Data.

[728] Klein T., Lenahan F. D., Kerscher M., Jander J. H., Rausch M. H., Koller T. M., Fröba A. P. (2021). Viscosity
and Interfacial Tension of Binary Mixtures of n-Hexadecane with Dissolved
Gases Using Surface Light Scattering and Equilibrium Molecular Dynamics
Simulations. Journal of Chemical & Engineering
Data.

[729] Klein T., Lenahan F. D., Zhai Z., Kerscher M., Jander J. H., Koller T. M., Rausch M. H., Fröba A. P. (2022). Viscosity
and Interfacial Tension of Binary Mixtures Consisting of Linear, Branched,
Cyclic, or Oxygenated Hydrocarbons with Dissolved Gases Using Surface
Light Scattering and Equilibrium Molecular Dynamics Simulations. International Journal of Thermophysics.

[730] Kerscher M., Klein T., Preuster P., Wasserscheid P., Koller T. M., Rausch M. H., Fröba A. P. (2022). Influence
of dissolved hydrogen on the viscosity and interfacial tension of
the liquid organic hydrogen carrier system based on diphenylmethane
by surface light scattering and molecular dynamics simulations. International Journal of Hydrogen Energy.

[731] Saric D., Guevara-Carrion G., Vrabec J. (2022). Thermodynamics of supercritical
carbon dioxide mixtures across the Widom line. Phys. Chem. Chem. Phys..

[732] Merker T., Engin C., Vrabec J., Hasse H. (2010). Molecular
model for carbon dioxide optimized to vapor-liquid equilibria. The Journal of Chemical Physics.

[733] Ye Y., Ahn C. C., Witham C., Fultz B., Liu J., Rinzler A. G., Colbert D., Smith K. A., Smalley R. E. (1999). Hydrogen
adsorption and cohesive energy of single-walled carbon nanotubes. Applied Physics Letters.

[734] Morales M. A., McMahon J. M., Pierleoni C., Ceperley D. M. (2013). Nuclear Quantum
Effects and Nonlocal Exchange-Correlation
Functionals Applied to Liquid Hydrogen at High Pressure. Phys. Rev. Lett..

[735] Cheng B., Paxton A. T., Ceriotti M. (2018). Hydrogen Diffusion
and Trapping in *α*-Iron: The Role of Quantum
and Anharmonic Fluctuations. Phys. Rev. Lett..

[736] Lenosky T. J., Kress J. D., Collins L. A., Kwon I. (1997). Molecular
dynamics simulations of compressed liquid hydrogen. Journal of Quantitative Spectroscopy and Radiative Transfer.

[737] Collins L. A., Kress J. D., Bickham S. R., Lenosky T. J., N J. T. (2000). Molecular dynamics simulations of
compressed hydrogen. High Pressure Research.

[738] Holst B., Redmer R., Gryaznov V. K., Fortov V. E., Iosilevskiy I. L. (2012). Hydrogen and deuterium in shock wave
experiments, ab
initio simulations and chemical picture modeling. The European Physical Journal D.

[739] Tian C., Liu F., Yuan H., Chen H., Kuan A. (2019). First-order liquid-liquid phase transition in compressed hydrogen
and critical point. The Journal of Chemical
Physics.

[740] Redmer R., Röpke G., Kuhlbrodt S., Reinholz H. (2001). Hopping conductivity in dense hydrogen
fluid. Phys. Rev. B.

[741] Liu X., Yu Y., Hou C., Ding J. (2024). Effects of
the wall
temperature on the boiling process and the molecular dynamics behavior
of the liquid hydrogen on a flat aluminum wall. Int. J. Hydrogen Energy.

[742] Greenfield M. L. (2020). Representing polymer molecular structure
using molecular
simulations for the study of liquid sorption and diffusion. Current Opinion in Chemical Engineering.

[743] Vergadou N., Theodorou D. N. (2019). Molecular
Modeling Investigations
of Sorption and Diffusion of Small Molecules in Glassy Polymers. Membranes.

[744] Zhao J., Wang X., Yang Q., Yin H., Zhao B., Zhang S., Wu C. (2022). Molecular dynamics
simulation of H_2_ in amorphous polyethylene system: H_2_ diffusion in various PE matrices and bubbling during rapid
depressurization. International Journal of Hydrogen
Energy.

[745] Li J., Zhao X., Liang J., Zhao C., Feng N., Guo G., Zhou Z. (2024). Molecular Dynamics Simulation of Hydrogen Barrier Performance
of Modified Polyamide 6 Lining of IV Hydrogen Storage Tank with Graphene. Polymers.

[746] Su Y., Lv H., Feng C., Zhang C. (2024). Hydrogen permeability
of polyamide 6 as the liner material of Type IV hydrogen storage tanks:
A molecular dynamics investigation. International
Journal of Hydrogen Energy.

[747] Pant P. V. K., Boyd R. H. (1993). Molecular-dynamics
simulation of
diffusion of small penetrants in polymers. Macromolecules.

[748] Zheng D., Li J., Liu B., Yu B., Yang Y., Han D., Li J., Huang Z. (2022). Molecular
dynamics investigations into the hydrogen permeation mechanism of
polyethylene pipeline material. Journal of Molecular
Liquids.

[749] Shi W., Siefert N. S., Morreale B. D. (2015). Molecular
Simulations of CO_2_, H_2_, H_2_O, and
H_2_S Gas Absorption
into Hydrophobic Poly­(dimethylsiloxane) (PDMS) Solvent: Solubility
and Surface Tension. J. Phys. Chem. C.

[750] Zhang X., Zhai L., Li H., Qi G., Gao X., Yang W. (2024). Molecular Simulation Study on the
Hydrogen Permeation
Behavior and Mechanism of Common Polymers. Polymers.

[751] Deas T. M., Hofer H. H., Dole M. (1972). Solubility
of Hydrogen in Polyethylene by a Semimicro Method. Macromolecules.

[752] Na Zhang, D. ; Ding, N. ; Zhang, Z. ; Cai, X. ; Dong Shao, X. ; Bu Li, H. Hydrogen Permeation Behavior of Polyethylene Liner for Type IV Vessel. Advances in New and Renewable Energy 2022, 10, 15–19.

[753] Sun Y., Lv H., Zhou W., Zhang C. (2020). Research on hydrogen
permeability of polyamide 6 as the liner material for type IV hydrogen
storage tank. International Journal of Hydrogen
Energy.

[754] Lu C., Ni S., Chen W., Liao J., Zhang C. (2010). A molecular
modeling study on small molecule gas transportation in poly (chloro-p-xylylene). Computational Materials Science.

[755] Kucukpinar E., Doruker P. (2006). Molecular simulations
of gas transport
in nitrile rubber and styrene butadiene rubber. Polymer.

[756] Tan J., Chen C., Liu Y., Wu J., Wu D., Zhang X., He X., She Z., He R., Zhang H. (2020). Molecular simulations of gas transport in hydrogenated nitrile butadiene
rubber and ethylene-propylene-diene rubber. RSC Adv..

[757] Tan J.-H., Chen C.-L., Liu Y.-W., Wu J.-Y., Wu D., Zhang X., She Z.-H., He R., Zhang H.-L. (2020). Molecular
simulations of gas transport in hydrogenated nitrile butadiene rubber. Journal of Polymer Research.

[758] Stalker M. R., Grant J., Yong C. W., Ohene-Yeboah L. A., Mays T. J., S C. P. (2021). Molecular simulation of hydrogen
storage and transport in cellulose. Molecular
Simulation.

[759] Yi Y., Bi P., Zhao X., Wang L. (2018). Molecular dynamics
simulation of diffusion of hydrogen and its isotopic molecule in polystyrene. Journal of Polymer Research.

[760] Zhao J., Li X., Wang X., Zhang Q., Yang Q., Yin H., Zhang S., Wu C. (2023). Insights into
the solubility of H_2_ in various polyethylene matrices at
high pressure: A coarse-grained MC/MD study. International Journal of Hydrogen Energy.

[761] Wilson M. A., Frischknecht A. L. (2022). High-pressure
hydrogen decompression
in sulfur crosslinked elastomers. International
Journal of Hydrogen Energy.

[762] Plimpton S. (1995). Fast parallel algorithms for short-range
molecular
dynamics. J. Comput. Phys..

[763] Brownell M., Frischknecht A. L., Wilson M. A. (2022). Subdiffusive High-Pressure
Hydrogen Gas Dynamics in Elastomers. Macromolecules.

[764] Wilson M. A., Winter I. S., Frischknecht A. L. (2025). Effects
of high-pressure hydrogen exposure on filler-elastomer adhesion. International Journal of Hydrogen Energy.

[765] Lorenz C., Webb E., Stevens M., Chandross M., Grest G. (2005). Frictional dynamics of perfluorinated
self-assembled monolayers on
amorphous SiO_2_. Tribology Letters.

[766] Ramdin M., de Loos T. W., Vlugt T. J. H. (2012). State-of-the-art
of CO_2_ capture with ionic liquids. Ind. Eng. Chem. Res..

[767] Shi W., Sorescu D. C., Luebke D. R., Keller M. J., Wickramanayake S. (2010). Molecular
Simulations and Experimental Studies of Solubility and Diffusivity
for Pure and Mixed Gases of H_2_, CO_2_, and Ar
Absorbed in the Ionic Liquid 1-n-Hexyl-3-methylimidazolium Bis­(Trifluoromethylsulfonyl)­amide
([hmim]­[Tf_2_N]). J. Phys. Chem. B.

[768] Shi W., Sorescu D. C. (2010). Molecular simulations
of CO_2_ and H_2_ sorption into ionic liquid 1-n-Hexyl-3-methylimidazolium
Bis­(trifluoromethylsulfonyl)­amide
([hmim]­[Tf_2_N]) confined in carbon nanotubes. J. Phys. Chem. B.

[769] Ramdin M., Balaji S. P., Vicent-Luna J. M., Gutiérrez-Sevillano J. J., Calero S., de Loos T. W., Vlugt T. J. H. (2014). Solubility of
the precombustion Gases CO_2_, CH_4_, CO, H_2_, N_2_, and H_2_S in the ionic liquid [bmim]­[Tf2N]
from Monte Carlo simulations. J. Phys. Chem.
C.

[770] Liu H., Maginn E. (2011). A molecular dynamics
investigation of the structural
and dynamic properties of the ionic liquid 1-n-butyl-3-methylimidazolium
bis­(trifluoromethanesulfonyl)­imide. The Journal
of Chemical Physics.

[771] Wittich B., Deiters U. K. (2010). Calculating Thermodynamic
Properties
of an Ionic Liquid with Monte Carlo Simulations with an Orthorhombic
and a Cubic Simulation Box. The Journal of Physical
Chemistry B.

[772] Shah J. K., Maginn E. J. (2004). A Monte Carlo simulation
study of
the ionic liquid 1-n-butyl-3-methylimidazolium hexafluorophosphate:
liquid structure, volumetric properties and infinite dilution solution
thermodynamics of CO_2_. Fluid Phase
Equilibria.

[773] Klein T., Piszko M., Lang M., Mehler J., Schulz P. S., Rausch M. H., Giraudet C., Koller T. M., Fröba A. P. (2020). Diffusivities in Binary Mixtures of [AMIM]­[NTf2] Ionic
Liquids with the Dissolved Gases H_2_, He, N_2_,
CO, CO_2_, or Kr Close to Infinite Dilution. Journal of Chemical & Engineering Data.

[774] Zhai Z., Hantal G., Cherian A., Bergen A., Chu J., Wick C. R., Meyer K., Smith A.-S., Koller T. M. (2024). Influence
of molecular hydrogen on bulk and interfacial properties of three
imidazolium-based ionic liquids by experiments and molecular dynamics
simulations. International Journal of Hydrogen
Energy.

[775] Seidl V., Bosch M., Paap U., Livraghi M., Zhai Z., Wick C. R., Koller T. M., Wasserscheid P., Maier F., Smith A.-S., Bachmann J., Steinrück H.-P., Meyer K. (2022). Bis-polyethylene glycol-functionalized imidazolium ionic liquids:
A multi-method approach towards bulk and surface properties. Journal of Ionic Liquids.

[776] Rivera-Pousa A., Lois-Cuns R., Otero-Lema M., Montes-Campos H., Méndez-Morales T., Varela L. M. (2024). Size Matters:
A Computational Study of Hydrogen Absorption in Ionic Liquids. Journal of Chemical Information and Modeling.

[777] Yang Q., Zhong C. (2006). Molecular Simulation
of Carbon Dioxide/Methane/Hydrogen
Mixture Adsorption in Metal-Organic Frameworks. The Journal of Physical Chemistry B.

[778] Liu B., Yang Q., Xue C., Zhong C., Chen B., Smit B. (2008). Enhanced Adsorption
Selectivity of Hydrogen/Methane Mixtures in Metal-Organic
Frameworks with Interpenetration: A Molecular Simulation Study. The Journal of Physical Chemistry C.

[779] Wu X., Huang J., Cai W., Jaroniec M. (2014). Force field for ZIF-8
flexible frameworks: atomistic simulation of adsorption, diffusion
of pure gases as CH_4_, H_2_, CO_2_ and
N_2_. RSC Adv..

[780] Keskin S. (2012). Adsorption, Diffusion, and Separation
of CH_4_/H_2_ Mixtures in Covalent Organic Frameworks:
Molecular
Simulations and Theoretical Predictions. The
Journal of Physical Chemistry C.

[781] Song M. K., No K. T. (2007). Molecular simulation of hydrogen
adsorption in organic zeolite. Catalysis Today.

[782] Deeg K. S., Gutiérrez-Sevillano J. J., Bueno-Pérez R., Parra J. B., Ania C. O., Doblaré M., Calero S. (2013). Insights on the Molecular Mechanisms of Hydrogen Adsorption
in Zeolites. J. Phys. Chem. C.

[783] Yilmaz G., Keskin S. (2012). Predicting the Performance
of Zeolite
Imidazolate Framework/Polymer Mixed Matrix Membranes for CO_2_, CH_4_, and H_2_ Separations Using Molecular Simulations. Industrial & Engineering Chemistry Research.

[784] Bobbitt N. S., Chen J., Snurr R. Q. (2016). High-Throughput
Screening of Metal-Organic Frameworks for Hydrogen Storage at Cryogenic
Temperature. Journal of Physical Chemistry C.

[785] Moosavi S. M., Nandy A., Jablonka K. M., Ongari D., Janet J. P., Boyd P. G., Lee Y., Smit B., Kulik H. J. (2020). Understanding the diversity of the
metal-organic framework
ecosystem. Nature communications.

[786] Wilmer C. E., Leaf M., Lee C. Y., Farha O. K., Hauser B. G., Hupp J. T., Snurr R. Q. (2012). Large-scale
screening
of hypothetical metal-organic frameworks. Nature
Chemistry.

[787] Avci G., Erucar I., Keskin S. (2020). Do New MOFs Perform
Better for CO_2_ Capture and H_2_ Purification?
Computational Screening of the Updated MOF Database. ACS Applied Materials & Interfaces.

[788] Allen F. H. (2002). The Cambridge Structural Database:
a quarter of a million
crystal structures and rising. Acta Crystallographica
B.

[789] Altundal O. F., Haslak Z. P., Keskin S. (2021). Combined GCMC,
MD,
and DFT Approach for Unlocking the Performances of COFs for Methane
Purification. Industrial & Engineering Chemistry
Research.

[790] Altintas C., Avci G., Daglar H., Nemati Vesali
Azar A., Erucar I., Velioglu S., Keskin S. (2019). An extensive comparative
analysis of two MOF databases: high-throughput screening of computation-ready
MOFs for CH_4_ and H_2_ adsorption. J. Mater. Chem. A.

[791] Chung Y. G., Camp J., Haranczyk M., Sikora B. J., Bury W., Krungleviciute V., Yildirim T., Farha O. K., Sholl D. S., Snurr R. Q. (2014). Computation-ready,
experimental metal-organic frameworks: A tool to enable high-throughput
screening of nanoporous crystals. Chemistry
of Materials.

[792] Moghadam P. Z., Li A., Wiggin S. B., Tao A., Maloney A. G., Wood P. A., Ward S. C., Fairen-Jimenez D. (2017). Development
of a Cambridge Structural Database Subset: A Collection of Metal-Organic
Frameworks for Past, Present, and Future. Chemistry
of Materials.

[793] Aksu G. O., Daglar H., Altintas C., Keskin S. (2020). Computational
Selection of High-Performing Covalent Organic Frameworks for Adsorption
and Membrane-Based CO_2_/H_2_ Separation. The Journal of Physical Chemistry C.

[794] Yan T., Lan Y., Tong M., Zhong C. (2019). Screening and Design
of Covalent Organic Framework Membranes for CO_2_/CH_4_ Separation. ACS Sustainable Chemistry
and Engineering.

[795] Avci G., Altintas C., Keskin S. (2021). Metal Exchange Boosts
the CO_2_ Selectivity of Metal Organic Frameworks Having
Zn-Oxide Nodes. The Journal of Physical Chemistry
C.

[796] Abdulkadir B., Mohd Zaki R., Abd Wahab A., Miskan S., Nguyen A.-T., Vo D.-V. N., Setiabudi H. (2024). A concise
review on surface and structural modification of porous zeolite scaffold
for enhanced hydrogen storage. Chinese Journal
of Chemical Engineering.

[797] Manda T., Barasa G. O., Louis H., Irfan A., Agumba J. O., Lugasi S. O., Pembere A. M. (2024). A data-guided approach
for the evaluation of zeolites for hydrogen storage with the aid of
molecular simulations. Journal of Molecular
Modeling.

[798] Liang P., Cao Y., Tai B., Zhang L., Shu H., Li F., Chao D., Du X. (2017). Is borophene a suitable
anode material for sodium ion battery?. Journal
of Alloys and Compounds.

[799] Osman A. I., Nasr M., Eltaweil A. S., Hosny M., Farghali M., Al-Fatesh A. S., Rooney D. W., Abd El-Monaem E. M. (2024). Advances
in hydrogen storage materials: harnessing innovative technology, from
machine learning to computational chemistry, for energy storage solutions. International Journal of Hydrogen Energy.

[800] Chung Y. G., Haldoupis E., Bucior B. J., Haranczyk M., Lee S., Zhang H., Vogiatzis K. D., Milisavljevic M., Ling S., Camp J. S., Slater B., Siepmann J. I., Sholl D. S., Snurr R. Q. (2019). Advances,
Updates, and Analytics
for the Computation-Ready, Experimental Metal-Organic Framework Database:
CoRE MOF 2019. Journal of Chemical & Engineering
Data.

[801] Deeg K. S., Damasceno Borges D., Ongari D., Rampal N., Talirz L., Yakutovich A. V., Huck J. M., Smit B. (2020). In Silico
Discovery of Covalent Organic Frameworks for Carbon Capture. ACS Applied Materials & Interfaces.

[802] Exploring the structure-property relationships of covalent organic frameworks for noble gas separations. Chemical Engineering Science 2017, 168, 456–464.

[803] Hellenbrandt M. (2004). The Inorganic
Crystal Structure Database (ICSD)-Present
and Future. Crystallography Reviews.

[804] Grazulis S., Chateigner D., Downs R. T., Yokochi A. F. T., Quiros M., Lutterotti L., Manakova E., Butkus J., Moeck P., Le Bail A. (2009). Crystallography
Open Database - an
open-access collection of crystal structures. Journal of Applied Crystallography.

[805] Groom C. R., Bruno I. J., Lightfoot M. P., Ward S. C. (2016). The Cambridge Structural Database. Acta Crystallographica Section B.

[806] Moosavi S. M., Jablonka K. M., Smit B. (2020). The Role of
Machine
Learning in the Understanding and Design of Materials. Journal of the American Chemical Society.

[807] Bucior B. J., Bobbitt N. S., Islamoglu T., Goswami S., Gopalan A., Yildirim T., Farha O. K., Bagheri N., Snurr R. Q. (2019). Energy-based descriptors to rapidly
predict hydrogen storage in metal-organic frameworks. Mol. Syst. Des. Eng..

[808] Dureckova H., Krykunov M., Aghaji M. Z., Woo T. K. (2019). Robust
Machine Learning Models for Predicting High CO_2_ Working
Capacity and CO_2_/H_2_ Selectivity of Gas Adsorption
in Metal Organic Frameworks for Precombustion Carbon Capture. The Journal of Physical Chemistry C.

[809] Yang W., Liang H., Peng F., Liu Z., Liu J., Qiao Z. (2019). Computational screening of metal-organic
framework
membranes for the separation of 15 gas mixtures. Nanomaterials.

[810] Shi Z., Yang W., Deng X., Cai C., Yan Y., Liang H., Liu Z., Qiao Z. (2020). Machine-learning-assisted
high-throughput computational screening of high performance metal-organic
frameworks. Mol. Syst. Des. Eng..

[811] Bai X., Shi Z., Xia H., Li S., Liu Z., Liang H., Liu Z., Wang B., Qiao Z. (2022). Machine-Learning-Assisted
High-Throughput computational screening of Metal-Organic framework
membranes for hydrogen separation. Chemical
Engineering Journal.

[812] Hertäg L., Bux H., Caro J., Chmelik C., Remsungnen T., Knauth M., Fritzsche S. (2011). Diffusion
of CH_4_ and H_2_ in ZIF-8. Journal of Membrane Science.

[813] He G., Dakhchoune M., Zhao J., Huang S., Agrawal K. V. (2018). Electrophoretic
Nuclei Assembly for Crystallization of High-Performance Membranes
on Unmodified Supports. Advanced Functional
Materials.

[814] Li Y.-S., Liang F.-Y., Bux H., Feldhoff A., Yang W.-S., Caro J. (2010). Molecular Sieve Membrane:
Supported
Metal-Organic Framework with High Hydrogen Selectivity. Angewandte Chemie International Edition.

[815] Huang A., Liang F., Steinbach F., Caro J. (2010). Preparation and separation properties of LTA membranes by using 3-aminopropyltriethoxysilane
as covalent linker. Journal of Membrane Science.

[816] Guo H., Zhu G., Hewitt I. J., Qiu S. (2009). “Twin Copper
Source” Growth of Metal-Organic Framework Membrane: Cu_3_(BTC)_2_ with High Permeability and Selectivity for
Recycling H_2_. Journal of the American
Chemical Society.

[817] Chang H., Wang Y., Xiang L., Liu D., Wang C., Pan Y. (2018). Improved H_2_/CO_2_ separation performance on mixed-linker
ZIF-7 polycrystalline membranes. Chemical Engineering
Science.

[818] Pulyalina A., Polotskaya G., Rostovtseva V., Pientka Z., Toikka A. (2018). Improved hydrogen
separation using
hybrid membrane composed of nanodiamonds and P84 copolyimide. Polymers.

[819] Safak Boroglu M., Yumru A. B. (2017). Gas separation performance
of 6FDA-DAM-ZIF-11
mixed-matrix membranes for H_2_/CH_4_ and CO_2_/CH_4_ separation. Separation
and Purification Technology.

[820] Lanč M., Sysel P., Soltys M., Stepánek F., Fonod K., Klepic M., Vopička O., Lhotka M., Ulbrich P., Friess K. (2018). Synthesis, preparation
and characterization of novel hyperbranched 6FDA-TTM based polyimide
membranes for effective CO_2_ separation: Effect of embedded
mesoporous silica particles and siloxane linkages. Polymer.

[821] Xue Q., Pan X., Li X., Zhang J., Guo Q. (2017). Effective
enhancement of gas separation performance in mixed matrix membranes
using core/shell structured multi-walled carbon nanotube/graphene
oxide nanoribbons. Nanotechnology.

[822] Iulianelli A., Algieri C., Donato L., Garofalo A., Galiano F., Bagnato G., Basile A., Figoli A. (2017). New PEEK-WC
and PLA membranes for H_2_ separation. International Journal of Hydrogen Energy.

[823] Macchione M., Jansen J. C., De Luca G., Tocci E., Longeri M., Drioli E. (2007). Experimental analysis
and simulation
of the gas transport in dense Hyflon^®^ AD60X membranes:
Influence of residual solvent. Polymer.

[824] Wijenayake S. N., Panapitiya N. P., Nguyen C. N., Huang Y., Balkus K. J., Musselman I. H., Ferraris J. P. (2014). Composite membranes
with a highly selective polymer skin for hydrogen separation. Separation and Purification Technology.

[825] Song Q., Nataraj S. K., Roussenova M. V., Tan J. C., Hughes D. J., Li W., Bourgoin P., Alam M. A., Cheetham A. K., Al-Muhtaseb S. A., Sivaniah E. (2012). Zeolitic imidazolate framework (ZIF-8) based polymer
nanocomposite membranes for gas separation. Energy Environ. Sci..

[826] Kim E. Y., Kim H. S., Kim D., Kim J., Lee P. S. (2019). Preparation
of mixed matrix membranes containing ZIF-8
and UiO-66 for multicomponent light gas separation. Crystals.

[827] Li Z., Yang P., Yan S., Fang Q., Xue M., Qiu S. (2019). A Robust Zeolitic Imidazolate
Framework Membrane with High H_2_/CO_2_ Separation
Performance under Hydrothermal
Conditions. ACS Applied Materials & Interfaces.

[828] Velioglu S., Keskin S. (2019). Simulation of H_2_/CH_4_ mixture permeation through MOF membranes using
non-equilibrium
molecular dynamics. J. Mater. Chem. A.

[829] Cacho-Bailo F., Catalán-Aguirre S., Etxeberría-Benavides M., Karvan O., Sebastian V., Téllez C., Coronas J. (2015). Metal-organic framework membranes
on the inner-side
of a polymeric hollow fiber by microfluidic synthesis. Journal of Membrane Science.

[830] Wang Y., Jin H., Ma Q., Mo K., Mao H., Feldhoff A., Cao X., Li Y., Pan F., Jiang Z. (2020). A MOF Glass Membrane for Gas Separation. Angewandte
Chemie International Edition.

[831] Li W., Su P., Zhang G., Shen C., Meng Q. (2015). Preparation
of continuous NH_2_-MIL-53 membrane on ammoniated polyvinylidene
fluoride hollow fiber for efficient H_2_ purification. Journal of Membrane Science.

[832] Huang A., Wang N., Kong C., Caro J. (2012). Organosilica-Functionalized
Zeolitic Imidazolate Framework ZIF-90 Membrane with High Gas-Separation
Performance. Angewandte Chemie International
Edition.

[833] Kang Z., Fan L., Wang S., Sun D., Xue M., Qiu S. (2017). In situ confinement
of free linkers within a stable
MOF membrane for highly improved gas separation properties. CrystEngComm.

[834] Kong C., Du H., Chen L., Chen B. (2017). Nanoscale
MOF/organosilica membranes on tubular ceramic substrates for highly
selective gas separation. Energy Environ. Sci..

[835] Aguado S., Nicolas C.-H., Moizan-Baslé V., Nieto C., Amrouche H., Bats N., Audebrand N., Farrusseng D. (2011). Facile synthesis of an ultramicroporous MOF tubular
membrane with selectivity towards CO_2_. New J. Chem..

[836] Al-Maythalony B. A., Shekhah O., Swaidan R., Belmabkhout Y., Pinnau I., Eddaoudi M. (2015). Quest for Anionic MOF Membranes:
Continuous sod-ZMOF Membrane with CO_2_ Adsorption-Driven
Selectivity. Journal of the American Chemical
Society.

[837] Qian Q., Wu A. X., Chi W. S., Asinger P. A., Lin S., Hypsher A., Smith Z. P. (2019). Mixed-Matrix
Membranes Formed from
Imide-Functionalized UiO-66-NH_2_ for Improved Interfacial
Compatibility. ACS Applied Materials & Interfaces.

[838] Feng S., Bu M., Pang J., Fan W., Fan L., Zhao H., Yang G., Guo H., Kong G., Sun H., Kang Z., Sun D. (2020). Hydrothermal
stable ZIF-67 nanosheets
via morphology regulation strategy to construct mixed-matrix membrane
for gas separation. Journal of Membrane Science.

[839] Esposito E., Bruno R., Monteleone M., Fuoco A., Ferrando Soria J., Pardo E., Armentano D., Jansen J. C. (2020). Glassy PEEK-WC vs. rubbery Pebax® 1657 polymers:
Effect on the gas transport in CuNi-MOF based mixed matrix membranes. Applied Sciences.

[840] Fan Y., Yu H., Xu S., Shen Q., Ye H., Li N. (2020). Zn­(II)-modified
imidazole containing polyimide/ZIF-8 mixed matrix
membranes for gas separations. Journal of Membrane
Science.

[841] Fan H., Peng M., Strauss I., Mundstock A., Meng H., Caro J. (2021). MOF-in-COF molecular
sieving membrane
for selective hydrogen separation. Nature Communications.

[842] Gao X., Zhang J., Huang K., Zhang J. (2018). ROMP for Metal-Organic
Frameworks: An Efficient Technique toward Robust and High-Separation
Performance Membranes. ACS Applied Materials
& Interfaces.

[843] Ashtiani S., Khoshnamvand M., Bouša D., Sturala J., Sofer Z., Shaliutina-Kolešová A., Gardenö D., Friess K. (2021). Surface and interface engineering
in CO_2_-philic based UiO-66-NH2-PEI mixed matrix membranes
via covalently bridging PVP for effective hydrogen purification. International Journal of Hydrogen Energy.

[844] Diestel L., Wang N., Schulz A., Steinbach F., Caro J. (2015). Matrimid-Based Mixed Matrix Membranes:
Interpretation and Correlation
of Experimental Findings for Zeolitic Imidazolate Frameworks as Fillers
in H_2_/CO_2_ Separation. Industrial & Engineering Chemistry Research.

[845] Zhuang G.-L., Wey M., hsin Tseng H. (2015). Theand Polyimide
in MOF-Incorporated PPO-silica mixed-matrix membranes produced via
the in situ sol-gel method for H_2_/CO_2_ separation.
II: Effect of thermal annealing treatment. Chemical
Engineering Research & Design.

[846] Jia Y., Liu P., Liu Y., Zhang D., Ning Y., Xu C., Zhang Y. (2023). In-situ interfacial
crosslinking of NH_2_-MIL-53
and polyimide in MOF-incorporated mixed matrix membranes for efficient
H_2_ purification. Fuel.

[847] Wijenayake S. N., Panapitiya N. P., Versteeg S. H., Nguyen C. N., Goel S., Balkus K. J. J., Musselman I. H., Ferraris J. P. (2013). Surface Cross-Linking of ZIF-8/Polyimide
Mixed Matrix
Membranes (MMMs) for Gas Separation. Industrial
& Engineering Chemistry Research.

[848] Nabais A. R., Martins A. P., Alves V. D., Crespo J. G., Marrucho I. M., Tomé L. C., Neves L. A. (2019). Poly­(ionic liquid)-based
engineered mixed matrix membranes for CO_2_/H_2_ separation. Separation and Purification Technology.

[849] Cheng J., Wang Y., Liu N., Hou W., Zhou J. (2020). Enhanced CO_2_ selectivity of mixed matrix
membranes with
carbonized Zn/Co zeolitic imidazolate frameworks. Applied Energy.

[850] Zhao D., Ren J., Qiu Y., Li H., Hua K., Li X., Deng M. (2015). Effect of graphene oxide on the behavior
of poly­(amide-6-b-ethylene oxide)/graphene oxide mixed-matrix membranes
in the permeation process. Journal of Applied
Polymer Science.

[851] Bux H., Liang F., Li Y., Cravillon J., Wiebcke M., Caro J. (2009). Zeolitic Imidazolate
Framework Membrane
with Molecular Sieving Properties by Microwave-Assisted Solvothermal
Synthesis. Journal of the American Chemical
Society.

[852] Li Y.-S., Bux H., Feldhoff A., Li G.-L., Yang W.-S., Caro J. (2010). Controllable Synthesis
of Metal-Organic
Frameworks: From MOF Nanorods to Oriented MOF Membranes. Advanced Materials.

[853] Huang A., Bux H., Steinbach F., Caro J. (2010). Molecular-Sieve Membrane with Hydrogen Permselectivity: ZIF-22 in
LTA Topology Prepared with 3-Aminopropyltriethoxysilane as Covalent
Linker. Angewandte Chemie International Edition.

[854] Bux H., Feldhoff A., Cravillon J., Wiebcke M., Li Y.-S., Caro J. (2011). Oriented Zeolitic Imidazolate
Framework-8 Membrane with Sharp H_2_/C_3_H_8_ Molecular Sieve Separation. Chemistry of Materials.

[855] Zhang F., Zou X., Gao X., Fan S., Sun F., Ren H., Zhu G. (2012). Hydrogen Selective
NH2-MIL-53­(Al)
MOF Membranes with High Permeability. Advanced
Functional Materials.

[856] Huang K., Dong Z., Li Q., Jin W. (2013). Growth of
a ZIF-8 membrane on the inner-surface of a ceramic hollow fiber via
cycling precursors. Chem. Commun..

[857] Wang N., Mundstock A., Liu Y., Huang A., Caro J. (2015). Amine-modified Mg-MOF-74/CPO-27-Mg
membrane with enhanced H_2_/CO_2_ separation. Chemical Engineering
Science.

[858] Knebel A., Friebe S., Bigall N. C., Benzaqui M., Serre C., Caro J. (2016). Comparative Study of MIL-96­(Al) as
Continuous Metal-Organic Frameworks Layer and Mixed-Matrix Membrane. ACS Applied Materials & Interfaces.

[859] Peng Y., Li Y., Ban Y., Yang W. (2017). Two-Dimensional
Metal-Organic Framework Nanosheets for Membrane-Based Gas Separation. Angewandte Chemie International Edition.

[860] Carta M., Malpass-Evans R., Croad M., Rogan Y., Jansen J. C., Bernardo P., Bazzarelli F., McKeown N. B. (2013). An Efficient Polymer Molecular Sieve
for Membrane Gas
Separations. Science.

[861] Han S. H., Kwon H. J., Kim K. Y., Seong J. G., Park C. H., Kim S., Doherty C. M., Thornton A. W., Hill A. J., Lozano A. E., Berchtold K. A., Lee Y. M. (2012). Tuning microcavities in thermally rearranged polymer
membranes for CO_2_ capture. Phys.
Chem. Chem. Phys..

[862] Do Y. S., Seong J. G., Kim S., Lee J. G., Lee Y. M. (2013). Thermally
rearranged (TR) poly­(benzoxazole-co-amide)
membranes for hydrogen separation derived from 3,3’-dihydroxy-4,4’-diamino-biphenyl
(HAB), 4,4’-oxydianiline (ODA) and isophthaloyl chloride (IPCl). Journal of Membrane Science.

[863] Pesiri D. R., Jorgensen B., Dye R. C. (2003). Thermal optimization
of polybenzimidazole meniscus membranes for the separation of hydrogen,
methane, and carbon dioxide. Journal of Membrane
Science.

[864] Scholes C. A., Smith K. H., Kentish S. E., Stevens G. W. (2010). CO_2_ capture
from pre-combustion processes—Strategies for
membrane gas separation. International Journal
of Greenhouse Gas Control.

[865] Yang T., Shi G. M., Chung T.-S. (2012). Symmetric and Asymmetric
Zeolitic Imidazolate Frameworks (ZIFs)/Polybenzimidazole (PBI) Nanocomposite
Membranes for Hydrogen Purification at High Temperatures. Advanced Energy Materials.

[866] Yang T., Xiao Y., Chung T.-S. (2011). Poly-/metal-benzimidazole
nano-composite membranes for hydrogen purification. Energy Environ. Sci..

[867] Zhu L., Swihart M. T., Lin H. (2018). Unprecedented size-sieving ability
in polybenzimidazole doped with polyprotic acids for membrane H_2_/CO_2_ separation. Energy Environ.
Sci..

[868] Shan M., Liu X., Wang X., Yarulina I., Seoane B., Kapteijn F., Gascon J. (2018). Facile manufacture
of porous organic framework membranes for precombustion CO_2_ capture. Science Advances.

[869] Kang Z., Peng Y., Qian Y., Yuan D., Addicoat M. A., Heine T., Hu Z., Tee L., Guo Z., Zhao D. (2016). Mixed Matrix Membranes (MMMs) Comprising
Exfoliated
2D Covalent Organic Frameworks (COFs) for Efficient CO_2_ Separation. Chemistry of Materials.

[870] Fu J., Das S., Xing G., Ben T., Valtchev V., Qiu S. (2016). Fabrication of COF-MOF Composite
Membranes and Their Highly Selective
Separation of H_2_/CO_2_. Journal of the American Chemical Society.

[871] Fan H., Mundstock A., Feldhoff A., Knebel A., Gu J., Meng H., Caro J. (2018). Covalent Organic Framework-Covalent
Organic Framework Bilayer Membranes for Highly Selective Gas Separation. Journal of the American Chemical Society.

[872] Ying Y., Liu D., Ma J., Tong M., Zhang W., Huang H., Yang Q., Zhong C. (2016). A GO-assisted
method for the preparation of ultrathin covalent organic framework
membranes for gas separation. J. Mater. Chem.
A.

[873] Tang Y., Feng S., Fan L., Pang J., Fan W., Kong G., Kang Z., Sun D. (2019). Covalent organic frameworks
combined with graphene oxide to fabricate membranes for H_2_/CO_2_ separation. Separation and
Purification Technology.

[874] Das S., Ben T. (2018). A [COF-300]-[UiO-66] composite membrane
with remarkably
high permeability and H_2_/CO_2_ separation selectivity. Dalton Trans..

[875] Fan H., Peng M., Strauss I., Mundstock A., Meng H., Caro J. (2020). High-Flux Vertically Aligned 2D Covalent
Organic Framework Membrane with Enhanced Hydrogen Separation. Journal of the American Chemical Society.

[876] Yang Q., Zhong C. (2006). Molecular simulation
of carbon dioxide/methane/hydrogen
mixture adsorption in metal-organic frameworks. J. Phys. Chem. B.

[877] Shang Z., Yang Y., Zhang L., Sun H., Zhong J., Zhang K., Yao J. (2024). Hydrogen adsorption
and diffusion behavior in kaolinite slit for underground hydrogen
storage: A hybrid GCMC-MD simulation study. Chemical Engineering Journal.

[878] Zheng R., Germann T. C., Gross M., Mehana M. (2024). Hydrogen Diffusion
in Slit Pores: Role of Temperature, Pressure, Confinement, and Roughness. Energy & Fuels.

[879] Oliver M. C., Zheng R., Huang L., Mehana M. (2024). Molecular
simulations of hydrogen diffusion in underground porous media: Implications
for storage under varying pressure, confinement, and surface chemistry
conditions. International Journal of Hydrogen
Energy.

[880] Ho T. A., Dang S. T., Dasgupta N., Choudhary A., Rai C. S., Wang Y. (2024). Nuclear magnetic
resonance and molecular
simulation study of H_2_ and CH_4_ adsorption onto
shale and sandstone for hydrogen geological storage. International Journal of Hydrogen Energy.

[881] Zhang M., Yang Y., Pan B., Liu Z., Jin Z., Iglauer S. (2024). Molecular simulation on H_2_ adsorption in
nanopores and effects of cushion gas: Implications for underground
hydrogen storage in shale reservoirs. Fuel.

[882] Kahzadvand K., Ghasemi M., Yazaydin A. O., Babaei M. (2024). Risk of H_2_ Leakage into Caprock and the
Role of Cushion Gas as a Barrier
in H_2_ Geo-Storage: A Molecular Simulation Study. The Journal of Physical Chemistry C.

[883] Muther T., Dahagi A. K. (2024). Monte-Carlo simulations
on H_2_ adsorption in kaolinite nanopore in the presence
of CO_2_ and CH_4_ gases. Fuel.

[884] Muther T., Dahaghi A. K. (2024). Calculation of hydrogen
adsorption
isotherms and Henry coefficients with mixed CO_2_ and CH_4_ gases on hydroxylated quartz surface: Implications to hydrogen
geo-storage. Journal of Energy Storage.

[885] Chen F., Wang S., Dejam M., Nasrabadi H. (2024). Molecular
Simulation of Competitive Adsorption of Hydrogen and Methane: Analysis
of Hydrogen Storage Feasibility in Depleted Shale Gas Reservoirs. SPE Journal.

[886] Zhao Y., Quan D., Wang C., Wu R., Zhang K., Bi J. (2025). Experiments and molecular simulations
study on hydrogen adsorption and diffusion behavior of the inorganic
mineral surfaces of shale. International Journal
of Hydrogen Energy.

[887] Xie C., Huang J., Li Y., Tian S., Zhao H. (2025). Molecular
Simulation on H_2_ Huff-n-Puff in a Depleted Shale Gas Reservoir
and Its Implications on Underground Hydrogen Storage. Energy & Fuels.

[888] Babaei S., Coasne B., Ostadhassan M. (2025). Adsorption-Induced
Deformation in Microporous Kerogen by Hydrogen and Methane: Implications
for Underground Hydrogen Storage. Langmuir.

[889] Zhang H., Diao R., Luo X., Xie Q. (2023). Molecular
Simulation of H_2_/CH_4_ Mixture Storage and Adsorption
in Kaolinite Nanopores for Underground Hydrogen Storage. ACS Omega.

[890] Zhang M., Yang Y., Pan B., Liu Z., Jin Z., Iglauer S. (2024). Molecular simulation on H_2_ adsorption in
nanopores and effects of cushion gas: Implications for underground
hydrogen storage in shale reservoirs. Fuel.

[891] Xie C., Huang J., Li Y., Tian S., Zhao H. (2025). Molecular
Simulation on H_2_ Huff-n-Puff in a Depleted Shale Gas Reservoir
and Its Implications on Underground Hydrogen Storage. Energy & Fuels.

[892] Kumar K. V., Müller E. A., Rodriguez-Reinoso F. (2012). Effect of
pore morphology on the adsorption of methane/hydrogen mixtures on
carbon micropores. The Journal of Physical Chemistry
C.

[893] Selim M., El-Nabarawy T. A. (1980). A general
relationship between adsorption
of hydrocarbons and their polarizabilities on activated carbon. Carbon.

[894] Ho L. N., Clauzier S., Schuurman Y., Farrusseng D., Coasne B. (2013). Gas uptake in solvents confined in
mesopores: Adsorption versus enhanced solubility. The Journal of Physical Chemistry Letters.

[895] Bui K. Q., Bao Le T. T., Barbosa G. D., Papavassiliou D. V., Razavi S., Striolo A. (2024). Molecular Density Fluctuations
Control
Solubility and Diffusion for Confined Aqueous Hydrogen. The Journal of Physical Chemistry Letters.

[896] Zhang H., Luo X., Yang D., Liu K., Xie Q., Diao R. (2023). Molecular simulation of H_2_ loss by dissolution
in caprock water-saturated nanopores under the nanoconfinement effect
for underground hydrogen storage. Energy &
Fuels.

[897] Choudhary A., Ho T. A. (2024). Roles of kaolinite-oil-gas molecular
interactions in hydrogen storage within depleted reservoirs. Chemical Engineering Journal.

[898] Choudhary A., Ho T. A. (2024). Confinement-induced clustering of
H_2_ and CO_2_ gas molecules in hydrated nanopores. Physical Chemistry Chemical Physics.

[899] Mashhadzadeh A. H., Faroughi S. A. (2025). Atomistic simulation
of dilute hydrogen
in water-saturated kaolinite nanopores: Implications for underground
hydrogen storage. International Journal of Hydrogen
Energy.

[900] Phan A., Cole D. R., Striolo A. (2014). Aqueous methane in
slit-shaped silica nanopores: high solubility and traces of hydrates. The Journal of Physical Chemistry C.

[901] Ball P. (2008). Water as an active constituent in
cell biology. Chemical reviews.

[902] Rego N. B., Patel A. J. (2022). Understanding hydrophobic
effects:
Insights from water density fluctuations. Annual
Review of Condensed Matter Physics.

[903] Jamadagni S. N., Godawat R., Garde S. (2011). Hydrophobicity
of proteins
and interfaces: Insights from density fluctuations. Annual review of chemical and biomolecular engineering.

[904] Sakamaki R., Sum A. K., Narumi T., Ohmura R., Yasuoka K. (2011). Thermodynamic properties of methane/water
interface
predicted by molecular dynamics simulations. The Journal of Chemical Physics.

[905] Wang S., Pan S., Tang Y., Mu Y., Gao Y., Wang K. (2024). Hydrogen-methane
transport in clay nanopores: Insights
from molecular dynamics simulations. International
Journal of Hydrogen Energy.

[906] Liu J., Wang S., Javadpour F., Feng Q., Cha L. (2022). Hydrogen diffusion
in clay slit: Implications for the geological storage. Energy & Fuels.

[907] A H., Yang Z., Chen Y., Hu R., Wood C. D., Kang Q., Chen Y.-F. (2024). H_2_ diffusion
in cement
nanopores and its implication for underground hydrogen storage. Journal of Energy Storage.

[908] Raza A., Alafnan S., Glatz G., Arif M., Mahmoud M., Rezk M. G. (2022). Hydrogen diffusion in organic-rich
porous media: implications for hydrogen geo-storage. Energy & Fuels.

[909] Kim C., Devegowda D., Dang S. T., Mehana M. (2025). Modeling the diffusivity
of hydrogen and the associated cushion gas in depleted hydrocarbon
reservoir caprocks. International Journal of
Hydrogen Energy.

[910] Ghasemi M., Omrani S., Mahmoodpour S., Zhou T. (2022). Molecular dynamics simulation of hydrogen diffusion in water-saturated
clay minerals; implications for Underground Hydrogen Storage (UHS). International Journal of Hydrogen Energy.

[911] Mercier Franco L. F., Castier M., Economou I. G. (2016). Diffusion
in Homogeneous
and in Inhomogeneous Media: A New Unified Approach. Journal of Chemical Theory and Computation.

[912] Franco L. F. M., Castier M., Economou I. G. (2016). Anisotropic
parallel
self-diffusion coefficients near the calcite surface: A molecular
dynamics study. The Journal of Chemical Physics.

[913] Santos M. S., Franco L. F. M., Castier M., Economou I. G. (2018). Molecular
Dynamics Simulation of n-Alkanes and CO_2_ Confined by Calcite
Nanopores. Energy & Fuels.

[914] Liu P., Harder E., Berne B. (2004). On the calculation
of diffusion coefficients
in confined fluids and interfaces with an application to the liquid-
vapor interface of water. The Journal of Physical
Chemistry B.

[915] Spera M.
B., Braga F. N., Bartolomeu R. A., Economou I. G., Franco L. F. (2022). Diffusion of fluids
confined in carbonate
minerals: A molecular dynamics simulation study for carbon dioxide
and methane-ethane mixture within calcite. Fuel.

[916] Předota M., Bandura A., Cummings P., Kubicki J., Wesolowski D., Chialvo A., Machesky M. L. (2004). Electric
double
layer at the rutile (110) surface. 1. Structure of surfaces and interfacial
water from molecular dynamics by use of ab initio potentials. The Journal of Physical Chemistry B.

[917] Botan A., Rotenberg B., Marry V., Turq P., Noetinger B. (2011). Hydrodynamics
in clay nanopores. The Journal of Physical Chemistry
C.

[918] Marry V., Rotenberg B., Turq P. (2008). Structure and dynamics
of water at a clay surface from molecular dynamics simulation. Physical Chemistry Chemical Physics.

[919] Rotenberg B., Marry V., Vuilleumier R., Malikova N., Simon C., Turq P. (2007). Water and ions in clays:
Unraveling the interlayer/micropore exchange using molecular dynamics. Geochimica et Cosmochimica Acta.

[920] Simonnin P., Noetinger B., Nieto-Draghi C., Marry V., Rotenberg B. (2017). Diffusion
under confinement: Hydrodynamic
finite-size effects in simulation. Journal of
Chemical Theory and Computation.

[921] Ho T. A., Jove-Colon C. F., Wang Y. (2023). Low hydrogen solubility
in clay interlayers limits gas loss in hydrogen geological storage. Sustainable Energy & Fuels.

[922] Muther T., Dahaghi A. K. (2024). Molecular insights
into hydrogen
intercalation with carbon dioxide and methane in hydrated clay: Implications
for hydrogen geo-storage seal integrity. International
Journal of Hydrogen Energy.

[923] Liu J., Zhang T., Sun S. (2024). Molecular
mechanisms of hydrogen
leakage through caprock in moisture and residual gas conditions: A
Molecular Dynamics-Monte Carlo study. Physics
of Fluids.

[924] Michels A., De Graaff W., Ten Seldam C. (1960). Virial coefficients
of hydrogen and deuterium at temperatures between -175 °C and
+150 °C. Conclusions from the second virial coefficient with
regards to the intermolecular potential. Physica.

[925] Chen B., Siepmann J. I. (1999). Transferable potentials
for phase
equilibria. 3. Explicit-hydrogen description of normal alkanes. The Journal of Physical Chemistry B.

[926] Zhu A., Zhang X., Liu Q., Zhang Q. (2009). A fully flexible potential
model for carbon dioxide. Chinese Journal of
Chemical Engineering.

[927] Cygan R. T., Romanov V. N., Myshakin E. M. (2012). Molecular simulation
of carbon dioxide capture by montmorillonite using an accurate and
flexible force field. The Journal of Physical
Chemistry C.

[928] Ayappa K., Mishra R. K. (2007). Freezing of fluids
confined between
mica surfaces. The Journal of Physical Chemistry
B.

[929] Chen B., Jiang H., Liu X., Hu X. (2017). Molecular
insight into water desalination across multilayer graphene oxide membranes. ACS Applied Materials & Interfaces.

[930] Waldman M., Hagler A. T. (1993). New combining rules
for rare gas
van der Waals parameters. Journal of Computational
Chemistry.

[931] Li X., Huo T., Wei K., Yan Z., Zhu L., Xue Q. (2024). The feasibility of
hydrogen storage in aquifers: A molecular dynamics
simulation. Fuel.

[932] Nan Y., Li W., Jin Z. (2021). Ion valency and concentration
effect
on the structural and thermodynamic properties of brine-decane interfaces
with anionic surfactant (SDS). The Journal of
Physical Chemistry B.

[933] Williams C. D., Burton N. A., Travis K. P., Harding J. H. (2014). The Development
of a Classical Force Field To Determine the Selectivity of an Aqueous
Fe^3+^-EDA Complex for TcO_4_
^‑^ and SO_4_
^2‑^. Journal of
Chemical Theory and Computation.

[934] Frankcombe T. J., Kroes G.-J. (2007). Molecular dynamics simulations of
type-sII hydrogen clathrate hydrate close to equilibrium conditions. The Journal of Physical Chemistry C.

[935] Luis D., Romero-Ramirez I., González-Calderon A., Lopez-Lemus J. (2018). The coexistence
temperature of hydrogen clathrates:
A molecular dynamics study. The Journal of Chemical
Physics.

[936] Luis D., González-Calderon A., Lopez-Lemus J. (2023). Effects of
externally-applied static electric fields on hydrogen hydrates: a
molecular dynamics study. Molecular Simulation.

[937] Efimchenko V., Antonov V., Barkalov O., Klyamkin S., Tkacz M. (2009). Two triple points in the H_2_O-H_2_ system. High Pressure Research.

[938] Kang D. W., Lee W., Ahn Y.-H., Lee J. W. (2022). Exploring
tuning phenomena of THF-H_2_ hydrates via molecular dynamics
simulations. Journal of Molecular Liquids.

[939] Anderson R., Chapoy A., Tohidi B. (2007). Phase relations
and
binary clathrate hydrate formation in the system H_2_-THF-H_2_O. Langmuir.

[940] Hu W., Tian X., Chen C., Cheng C., Zhu S., Zhang J., Qi T., Jin T., Wu X. (2024). Molecular
dynamic simulation of H_2_-CH_4_ binary hydrate
growth induced by methane hydrate. Fuel.

[941] Ghaani M. R., English N. J. (2019). Hydrogen-/propane-hydrate
decomposition:
thermodynamic and kinetic analysis. Molecular
Physics.

[942] Ghaani M. R., Takeya S., English N. J. (2020). Hydrogen storage
in propane-hydrate: Theoretical and experimental study. Applied Sciences.

[943] Wang P., Li K., Yang J., Zhu J., Zhao Y., Teng Y. (2021). Experimental
and theoretical study
on dissociation thermodynamics and kinetics of hydrogen-propane hydrate. Chemical Engineering Journal.

[944] Gorman P. D., English N. J., MacElroy J. (2011). Dynamical and energetic
properties of hydrogen and hydrogen-tetrahydrofuran clathrate hydrates. Physical Chemistry Chemical Physics.

[945] Yagasaki T., Himoto K., Nakamura T., Matsumoto M., Tanaka H. (2015). Structure, dynamics and thermodynamic
stability of
high-pressure ices and clathrate hydrates. Molecular
Simulation.

[946] English N. J., Gorman P. D., MacElroy J. (2012). Mechanisms for thermal
conduction in hydrogen hydrate. The Journal
of Chemical Physics.

[947] Zhao W., Wang L., Bai J., Francisco J. S., Zeng X. C. (2014). Spontaneous formation of one-dimensional
hydrogen gas
hydrate in carbon nanotubes. Journal of the
American Chemical Society.

[948] Alavi S., Ripmeester J., Klug D. (2006). Molecular-dynamics
simulations of binary structure II hydrogen and tetrahydrofurane clathrates. The Journal of Chemical Physics.

[949] Alavi S., Ripmeester J., Klug D. (2006). Molecular dynamics
simulations of binary structure H hydrogen and methyl-tert-butylether
clathrate hydrates. The Journal of Chemical
Physics.

[950] Daschbach J. L., Chang T.-M., Corrales L. R., Dang L. X., McGrail P. (2006). Molecular mechanisms of hydrogen-loaded *β*-hydroquinone clathrate. The
Journal of Physical
Chemistry B.

[951] Wang Y., Yin K., Lang X., Fan S., Li G., Yu C., Wang S. (2021). Hydrogen storage in
sH binary hydrate:
Insights from molecular dynamics simulation. International Journal of Hydrogen Energy.

[952] Lokshin K. A., Zhao Y., He D., Mao W. L., Mao H.-K., Hemley R. J., Lobanov M. V., Greenblatt M. (2004). Structure
and dynamics of hydrogen molecules in the novel clathrate hydrate
by high pressure neutron diffraction. Physical
Review Letters.

[953] Katsumasa K., Koga K., Tanaka H. (2007). On the thermodynamic
stability of hydrogen clathrate hydrates. The
Journal of Chemical Physics.

[954] Chun D.-H., Lee T.-Y. (2008). Molecular simulation
of cage occupancy
and selectivity of binary THF-H_2_ sII hydrate. Molecular Simulation.

[955] Nakayama T., Koga K., Tanaka H. (2009). Augmented stability
of hydrogen clathrate hydrates by weakly polar molecules. The Journal of Chemical Physics.

[956] Nakayama, T. ; Matsumoto, M. ; Tanaka, H. On the thermodynamic stability of hydrogen hydrates in the presence of promoter molecules. AIP Conference Proceedings. 2013; pp 46–52.

[957] Koh D.-Y., Kang H., Jeon J., Ahn Y.-H., Park Y., Kim H., Lee H. (2014). Tuning cage dimension
in clathrate hydrates for hydrogen multiple occupancy. The Journal of Physical Chemistry C.

[958] Hakim L., Koga K., Tanaka H. (2010). Phase behavior
of different
forms of ice filled with hydrogen molecules. Physical Review Letters.

[959] Hakim L., Koga K., Tanaka H. (2010). Thermodynamic stability
of hydrogen hydrates of ice Ic and II structures. Physical Review B.

[960] Duarte A. R. C., Shariati A., Rovetto L. J., Peters C. J. (2008). Water cavities
of sH clathrate hydrate stabilized by molecular hydrogen: Phase equilibrium
measurements. The Journal of Physical Chemistry
B.

[961] Grim R. G., Kerkar P. B., Shebowich M., Arias M., Sloan E. D., Koh C. A., Sum A. K. (2012). Synthesis
and characterization of sI clathrate hydrates containing hydrogen. The Journal of Physical Chemistry C.

[962] Papadimitriou N., Tsimpanogiannis I., Stubos A. (2010). Computational approach
to study hydrogen storage in clathrate hydrates. Colloids and Surfaces A: Physicochemical and Engineering Aspects.

[963] Strobel T. A., Taylor C. J., Hester K. C., Dec S. F., Koh C. A., Miller K. T., Sloan E. (2006). Molecular
hydrogen
storage in binary THF-H_2_ clathrate hydrates. The Journal of Physical Chemistry B.

[964] Ogata K., Hashimoto S., Sugahara T., Moritoki M., Sato H., Ohgaki K. (2008). Storage capacity
of hydrogen in tetrahydrofuran
hydrate. Chemical Engineering Science.

[965] Mulder F. M., Wagemaker M., Van Eijck L., Kearley G. J. (2008). Hydrogen in porous tetrahydrofuran
clathrate hydrate. ChemPhysChem.

[966] Papadimitriou N., Tsimpanogiannis I., Stubos A. (2009). Gas content of binary
clathrate hydrates with promoters. The Journal
of Chemical Physics.

[967] Hester K. C., Strobel T. A., Sloan E. D., Koh C. A., Huq A., Schultz A. J. (2006). Molecular hydrogen
occupancy in binary THF-H_2_ clathrate hydrates by high resolution
neutron diffraction. The Journal of Physical
Chemistry B.

[968] Nagai Y., Yoshioka H., Ota M., Sato Y., Inomata H., Smith R. L., Peters C. J. (2008). Binary
hydrogen-tetrahydrofuran clathrate hydrate formation kinetics and
models. AIChE Journal.

[969] Yoshioka H., Ota M., Sato Y., Watanabe M., Inomata H., Smith R. L., Peters C. J. (2011). Decomposition
kinetics and recycle of binary hydrogen-tetrahydrofuran clathrate
hydrate. AIChE Journal.

[970] Ulivi L., Celli M., Giannasi A., Ramirez-Cuesta A., Bull D., Zoppi M. (2007). Quantum rattling of
molecular hydrogen
in clathrate hydrate nanocavities. Physical
Review B—Condensed Matter and Materials Physics.

[971] Talyzin A. (2008). Feasibility of H_2_-THF-H_2_O clathrate
hydrates for hydrogen storage applications. International Journal of Hydrogen Energy.

[972] Sugahara T., Haag J. C., Prasad P. S., Warntjes A. A., Sloan E. D., Sum A. K., Koh C. A. (2009). Increasing
hydrogen
storage capacity using tetrahydrofuran. Journal
of the American Chemical Society.

[973] Sugahara T., Haag J. C., Warntjes A. A., Prasad P. S., Sloan E. D., Koh C. A., Sum A. K. (2010). Large-cage
occupancies
of hydrogen in binary clathrate hydrates dependent on pressures and
guest concentrations. The Journal of Physical
Chemistry C.

[974] Kawamura T., Takeya S., Ohtake M., Yamamoto Y. (2011). Enclathration
of hydrogen by organic-compound clathrate hydrates. Chemical Engineering Science.

[975] Tsimpanogiannis I. N., Economou I. G., Stubos A. K. (2020). A Practical
Methodology
to Estimate the H_2_ Storage Capacity of Pure and Binary
Hydrates Based on Monte Carlo Simulations. Journal
of Chemical & Engineering Data.

[976] Luo Y., Liu A., Guo X., Sun Q., Yang L. (2015). Experiment
on the continuous recovery of H_2_ from hydrogenation plant
off-gas via hydrate formation in tetra-n-butyl ammonium bromide solution. International Journal of Hydrogen Energy.

[977] Li Q., Fan S., Chen Q., Yang G., Chen Y., Li L., Li G. (2019). Experimental
and process simulation of hydrate-based
CO_2_ capture from biogas. Journal
of Natural Gas Science and Engineering.

[978] Misawa T., Ishikawa T., Takeya S., Alavi S., Ohmura R. (2023). Continuous hydrate-based CO_2_ separation
from H_2_ + CO_2_ gas mixture using cyclopentane
as co-guest. Journal of Industrial and Engineering
Chemistry.

[979] Lee Y., Lee S., Seo D., Moon S., Ahn Y.-H., Park Y. (2024). Highly efficient
separation and equilibrium recovery of H_2_/CO_2_ in hydrate-based pre-combustion CO_2_ capture. Chemical Engineering Journal.

[980] Glavatskiy K., Vlugt T. J. H., Kjelstrup S. (2012). Toward a possibility
to exchange CO_2_ and CH_4_ in sI clathrate hydrates. The Journal of Physical Chemistry B.

[981] Papadimitriou N. I., Tsimpanogiannis I. N., Economou I. G., Stubos A. K. (2018). Monte Carlo
simulations of the separation of a binary gas mixture (CH_4_ + CO_2_) using hydrates. Physical
Chemistry Chemical Physics.

[982] Atamas A., Koudriachova M. V., de Leeuw S. W., Sweatman M. B. (2011). Monte Carlo
calculations of the free energy of ice-like structures using the self-referential
method. Molecular Simulation.

[983] Atamas A. A., Cuppen H. M., Koudriachova M. V., de Leeuw S. W. (2013). Monte Carlo calculations of the free energy of binary
sII hydrogen clathrate hydrates for identifying efficient promoter
molecules. The Journal of Physical Chemistry
B.

[984] Atamas A.
A., Koudriachova M. V., de Leeuw S. W., Cuppen H. M. (2014). Free energy
calculations for identifying efficient promoter molecules of binary
sH hydrogen clathrates. The Journal of Physical
Chemistry C.

[985] Frankcombe T. J., Kroes G.-J. (2011). A new method
for screening potential
sII and sH hydrogen clathrate hydrate promoters with model potentials. Physical Chemistry Chemical Physics.

[986] Atamas A. A., de Leeuw S. W., Cuppen H. M. (2015). A method
distinguishing
between guest molecules that can form sI, sII, and sH hydrogen clathrates. RSC Advances.

[987] Iwai Y., Aokawa R. (2015). Stability analysis for binary sII
hydrogen-promoter hydrates by molecular dynamics simulation. Molecular Simulation.

[988] Davidson, D. In Water in Crystalline Hydrates Aqueous Solutions of Simple Nonelectrolytes; Franks, F. , Ed.; Plenum: New York, 1973; pp 115–234.

[989] Okuchi T., Moudrakovski I. L., Ripmeester J. (2007). Efficient
storage of hydrogen fuel into leaky cages of clathrate hydrate. Applied Physics Letters.

[990] Senadheera L., Conradi M. S. (2007). Rotation
and diffusion of H_2_ in hydrogen-ice clathrate by 1H NMR. The Journal
of Physical Chemistry B.

[991] Senadheera L., Conradi M. S. (2008). Hydrogen NMR of H_2_-TDF-D_2_O Clathrate. The Journal of Physical
Chemistry B.

[992] Choi Y. N., Park J. S., Strässle T., Yeon S.-H., Park Y., Lee H. (2010). Dynamics of hydrogen
molecules in the channels of binary THF-H_2_ clathrate hydrate
and its physicochemical significance on hydrogen storage. International Journal of Hydrogen Energy.

[993] Strobel T. A., Sloan E. D., Koh C. A. (2009). Raman spectroscopic
studies of hydrogen clathrate hydrates. The
Journal of Chemical Physics.

[994] Russina M., Kemner E., Mezei F. (2016). Intra-cage
dynamics
of molecular hydrogen confined in cages of two different dimensions
of clathrate hydrates. Scientific Reports.

[995] Pefoute E., Kemner E., Soetens J., Russina M., Desmedt A. (2012). Diffusive motions of molecular hydrogen
confined in
THF clathrate hydrate. The Journal of Physical
Chemistry C.

[996] Cao H., English N. J., MacElroy J. (2013). Diffusive
hydrogen inter-cage migration
in hydrogen and hydrogen-tetrahydrofuran clathrate hydrates. The Journal of Chemical Physics.

[997] Gorman P. D., English N. J., MacElroy J. (2012). Dynamical cage behaviour
and hydrogen migration in hydrogen and hydrogen-tetrahydrofuran clathrate
hydrates. The Journal of Chemical Physics.

[998] Iwai Y., Hirata M. (2012). Molecular dynamics
simulation of
diffusion of hydrogen in binary hydrogen-tetrahydrofuran hydrate. Molecular Simulation.

[999] Geng C.-Y., Han Q.-Z., Wen H., Dai Z.-Y., Song C.-H. (2010). Molecular dynamics simulation on the decomposition
of type SII hydrogen hydrate and the performance of tetrahydrofuran
as a stabiliser. Molecular Simulation.

[1000] Hasegawa T., Brumby P. E., Yasuoka K., Sum A. K. (2020). Mechanism
for H_2_ diffusion in sII hydrates by molecular dynamics
simulations. The Journal of Chemical Physics.

[1001] Papadimitriou N. I., Tsimpanogiannis I. N., Stubos A. K., Martin A., Rovetto L. J., Peters C. J. (2010). Unexpected
behavior of helium as
guest gas in sII binary hydrates. The Journal
of Physical Chemistry Letters.

[1002] Papadimitriou N. I., Tsimpanogiannis I. N., Stubos A. K., Martín A., Rovetto L. J., Florusse L. J., Peters C. J. (2011). Experimental and
computational investigation of the sII binary He-THF hydrate. The Journal of Physical Chemistry B.

[1003] Amano S., Tsuda T., Hashimoto S., Sugahara T., Ohgaki K. (2010). Competitive cage occupancy of hydrogen
and argon in structure-II hydrates. Fluid Phase
Equilibria.

[1004] Waage M. H., Trinh T. T., van Erp T. S. (2018). Diffusion of gas
mixtures in the sI hydrate structure. The Journal
of Chemical Physics.

[1005] Harada A., Arman Y., Miura S. (2019). Molecular
dynamics
study on fast diffusion of hydrogen molecules in filled ice II. Journal of Molecular Liquids.

[1006] Arman Y., Nugroho B. (2021). Molecular dynamics study of hydrogen
diffusion in the C2 Hydrogen Hydrates. Journal
of Physics: Conference Series.

[1007] Smirnov G. S., Stegailov V. V. (2015). Anomalous diffusion of guest molecules
in hydrogen gas hydrates. High Temperature.

[1008] Alavi S., Ripmeester J. (2007). Hydrogen-gas
migration through clathrate
hydrate cages. Angewandte Chemie - International
Edition.

[1009] Parrinello M., Rahman A. (1984). Study of an F center
in molten KCl. The Journal of Chemical Physics.

[1010] Burnham C. J., English N. J. (2016). Free-energy calculations
of the intercage
hopping barriers of hydrogen molecules in clathrate hydrates. The Journal of Physical Chemistry C.

[1011] Burnham C. J., Futera Z., English N. J. (2017). Quantum
and classical
inter-cage hopping of hydrogen molecules in clathrate hydrate: Temperature
and cage-occupation effects. Physical Chemistry
Chemical Physics.

[1012] Burnham C. J., Futera Z., English N. J. (2018). Study of hydrogen-molecule
guests in type II clathrate hydrates using a force-matched potential
model parameterised from ab initio molecular dynamics. The Journal of Chemical Physics.

[1013] English N. J., Burnham C. J. (2021). Intra-cage structure, vibrations
and tetrahedral-site hopping of H_2_ and D_2_ in
doubly-occupied 5^12^6^4^ cages in sII clathrate
hydrates from path-integral and classical molecular dynamics. Applied Sciences.

[1014] Krishnan Y., Ghaani M. R., English N. J. (2021). Hydrogen and deuterium
molecular escape from clathrate hydrates:“Leaky” microsecond-molecular-dynamics
predictions. The Journal of Physical Chemistry
C.

[1015] Krishnan Y., Rosingana P. G., Ghaani M. R., English N. J. (2022). Controlling
hydrogen release from remaining-intact Clathrate hydrates by electromagnetic
fields: molecular engineering via microsecond non-equilibrium molecular
dynamics. RSC Advances.

[1016] Krishnan Y., Ghaani M. R., Desmedt A., English N. J. (2021). Hydrogen
inter-cage hopping and cage occupancies inside hydrogen hydrate: Molecular-dynamics
analysis. Applied Sciences.

[1017] Hosseini M., Fahimpour J., Ali M., Keshavarz A., Iglauer S. (2022). H_2_-brine interfacial tension as a function
of salinity, temperature, and pressure; implications for hydrogen
geo-storage. Journal of Petroleum Science and
Engineering.

[1018] Omrani S., Ghasemi M., Singh M., Mahmoodpour S., Zhou T., Babaei M., Niasar V. (2023). Interfacial
Tension-Temperature-Pressure-Salinity
Relationship for the Hydrogen-Brine System under Reservoir Conditions:
Integration of Molecular Dynamics and Machine Learning. Langmuir.

[1019] Xie M., Zhang M., Jin Z. (2024). Machine Learning-Based
Interfacial
Tension Equations for (H_2_ + CO_2_)-Water/Brine
Systems over a Wide Range of Temperature and Pressure. Langmuir.

[1020] Doan Q. T., Keshavarz A., Miranda C. R., Behrenbruch P., Iglauer S. (2023). Molecular dynamics simulation of interfacial tension
of the CO_2_-CH_4_-water and H_2_-CH_4_-water systems at the temperature of 300 K and 323 K and pressure
up to 70 MPa. Journal of Energy Storage.

[1021] Doan Q. T., Keshavarz A., Miranda C. R., Behrenbruch P., Iglauer S. (2024). A prediction of interfacial
tension by using molecular
dynamics simulation: A study on effects of cushion gas (CO_2_, N_2_ and CH_4_) for Underground Hydrogen Storage. Int. J. Hydrogen Energy.

[1022] Adam A. M., Bahamon D., Al Kobaisi M., Vega L. F. (2024). Molecular dynamics simulations of the interfacial tension
and the solubility of brine/H_2_/CO_2_ systems:
Implications for underground hydrogen storage. International Journal of Hydrogen Energy.

[1023] Blokhuis E. M., Bedeaux D., Holcomb C. D., Zollweg J. A. (1995). Tail corrections
to the surface tension of a Lennard-Jones liquid-vapour interface. Mol. Phys..

[1024] Essmann U., Perera L., Berkowitz M. L., Darden T., Lee H., Pedersen L. G. (1995). A smooth particle
mesh Ewald method. J. Comput. Phys..

[1025] Hockney, R. ; Eastwood, J. Computer Simulation Using Particles; CRC Press: New York, 1988.

[1026] Isele-Holder R. E., Mitchell W., Ismail A. E. (2012). Development and
application of a particle-particle particle-mesh Ewald method for
dispersion interactions. J. Chem. Phys..

[1027] Goujon F., Ghoufi A., Malfreyt P., Tildesley D. J. (2015). Controlling
the long-range corrections in atomistic Monte Carlo simulations of
two-phase systems. J. Chem. Theory Comput..

[1028] Sega M., Dellago C. (2017). Long-range dispersion
effects on
the water/vapor interface simulated using the most common models. J. Phys. Chem. B.

[1029] Wennberg C. L., Murtola T., Páll S., Abraham M. J., Hess B., Lindahl E. (2015). Direct-space corrections
enable fast and accurate Lorentz-Berthelot combination rule Lennard-Jones
lattice summation. J. Chem. Theory Comput..

[1030] Macedonia M. D., Maginn E. J. (1999). A biased grand canonical
Monte Carlo
method for simulating adsorption using all-atom and branched united
atom models. Mol. Phys..

[1031] Siperstein F., Myers A., Talu O. (2002). Long range
corrections
for computer simulations of adsorption. Mol.
Phys..

[1032] Jablonka K. M., Ongari D., Smit B. (2019). Applicability of tail
corrections in the molecular simulations of porous materials. J. Chem. Theory Comput..

[1033] Wang X., Ramírez-Hinestrosa S., Dobnikar J., Frenkel D. (2020). The Lennard-Jones potential: when (not) to use it. Physical Chemistry Chemical Physics.

[1034] Zeron I. M., Abascal J. L. F., Vega C. (2019). A force field
of Li^+^, Na^+^, K^+^, Mg^2+^,
Ca^2+^, Cl^–^, and SO_4_
^2‑^ in
aqueous solution based on the TIP4P/2005 water model and scaled charges
for the ions. J. Chem. Phys..

[1035] Loche P., Steinbrunner P., Friedowitz S., Netz R. R., Bonthuis D. J. (2021). Transferable
Ion Force Fields in
Water from a Simultaneous Optimization of Ion Solvation and Ion-Ion
Interaction. J. Phys. Chem. B.

[1036] Vega C., Miguel E. D. (2007). Surface tension
of the most popular
models of water by using the test-area simulation method. J. Chem. Phys..

[1037] Papavasileiou K. D., Moultos O. A., Economou I. G. (2018). Predictions of water/oil
interfacial tension at elevated temperatures and pressures: A molecular
dynamics simulation study with biomolecular force fields. Fluid Phase Equilib..

[1038] Salehi H. S., Moultos O. A., Vlugt T. J. H. (2021). Interfacial Properties
of Hydrophobic Deep Eutectic Solvents with Water. J. Phys. Chem. B.

[1039] Isele-Holder R. E., Mitchell W., Hammond J. R., Kohlmeyer A., Ismail A. E. (2013). Reconsidering Dispersion Potentials:
Reduced Cutoffs
in Mesh-Based Ewald Solvers Can Be Faster Than Truncation. Journal of Chemical Theory and Computation.

[1040] Isele-Holder R. E., Mitchell W., Ismail A. E. (2012). Development
and
application of a particle-particle particle-mesh Ewald method for
dispersion interactions. The Journal of Chemical
Physics.

[1041] Chow Y. F., Maitland G. C., Trusler J. M. (2018). Interfacial
tensions
of (H_2_O + H_2_) and (H_2_O + CO_2_ + H_2_) systems at temperatures of (298-448) K and pressures
up to 45 MPa. Fluid Phase Equilibria.

[1042] Abascal J. L. F., Vega C. (2005). A general purpose model
for the condensed
phases of water: TIP4P/2005. J. Comput. Phys..

[1043] Dang L. X. (1995). Mechanism and thermodynamics of ion
selectivity in
aqueous solutions of 18-crown-6 ether: a molecular dynamics study. Journal of the American Chemical Society.

[1044] Aqvist J. (1990). Ion-water interaction potentials
derived from free
energy perturbation simulations. J. Phys. Chem..

[1045] Potoff J. J., Siepmann J. I. (2001). Vapor-liquid equilibria
of mixtures
containing alkanes, carbon dioxide, and nitrogen. AIChE J..

[1046] Chen C., Dong B., Zhang N., Li W., Song Y. (2016). Pressure and
Temperature Dependence of Contact Angles for CO_2_/Water/Silica
Systems Predicted by Molecular Dynamics Simulations. Energy & Fuels.

[1047] Zhuravlev L. (2000). The surface chemistry of amorphous
silica. Zhuravlev
model. Colloids and Surfaces A: Physicochemical
and Engineering Aspects.

[1048] Ali A., Cole D. R., Striolo A. (2024). Cushion gas effects
on clay-hydrogen-brine
wettability at conditions relevant to underground gas storage. International Journal of Hydrogen Energy.

[1049] Alshammari S., Abdel-Azeim S., Al-Yaseri A., Qasim A. (2024). The Influence of CH_4_ and
CO_2_ on the Interfacial
Tension of H_2_-Brine, Water-H_2_-Rock Wettability,
and Their Implications on Geological Hydrogen Storage. Energy Fuels.

[1050] Barbosa G. D., Bui K. Q., Papavassiliou D. V., Razavi S., Striolo A. (2025). Wettability of Chemically Heterogeneous
Clay Surfaces: Correlation between Surface Defects and Contact Angles
as Revealed by Machine Learning. ACS Appl. Mater.
Interfaces.

[1051] Fatah A., Al-Yaseri A., Alshammari S., Al-Qasim A. S. (2024). Gas Injection Dynamics
and Hydrogen Storage in Carbonate
Reservoirs: Core-Flooding and Molecular Simulation Study. Energy Fuels.

[1052] Ghafari M. A., Ghasemi M., Niasar V., Babaei M. (2024). Wetting Preference
of Silica Surfaces in the Context of Underground Hydrogen Storage:
A Molecular Dynamics Perspective. Langmuir.

[1053] Phan A., Barker V., Hassanpouryouzband A., Ho T. A. (2025). Simulation insights into wetting properties of hydrogen-brine-clay
for hydrogen geo-storage. Journal of Energy
Storage.

[1054] Yao X., Narayanan Nair A. K., Che Ruslan M. F. A., Sun S., Yan B. (2025). Interfacial properties
of the hydrogen+brine
system in the presence of hydrophilic silica. International Journal of Hydrogen Energy.

[1055] Kwok D. Y., Neumann A. W. (1999). Contact angle measurement
and contact
angle interpretation. Advances in colloid and
interface science.

[1056] Yu X., Rao S., Zhang L., Li Y., Liu C., Yang M., Chen Z. (2025). In-situ wettability alteration of
organic-rich shale caprock in hydrogen with cushion gas: Implications
for hydrogen geo-storage. International Journal
of Hydrogen Energy.

[1057] Zhang S., Tan D., Zhu H., Zhang W. (2025). Molecular
dynamic simulations on the hydrogen wettability of caprock: Considering
effects of mineralogy, pressure, temperature and salinity. International Journal of Hydrogen Energy.

[1058] Huang F., Yang Y., Kang S., Wang K., Zhang M. (2024). Microscopic insights into water wetting
behaviors and physical origin
on *α*-quartz exposed to varying underground
gas species. Chemical Engineering Journal.

[1059] Chandrasekhar J., Spellmeyer D. C., Jorgensen W. L. (1984). Energy
component analysis for dilute aqueous solutions of lithium (1+), sodium
(1+), fluoride (1-), and chloride (1-) ions. J. Am. Chem. Soc..

[1060] Alavi S., Ripmeester J., Klug D. (2005). Molecular-dynamics
study of structure II hydrogen clathrates. J.
Chem. Phys..

[1061] Izadi S., Anandakrishnan R., Onufriev A. V. (2014). Building water models:
A different approach. J. Phys. Chem. Lett..

[1062] Yagasaki T., Matsumoto M., Tanaka H. (2020). Lennard-Jones parameters
determined to reproduce the solubility of NaCl and KCl in SPC/E, TIP3P,
and TIP4P/2005 water. Journal of Chemical Theory
and Computation.

[1063] Emami F. S., Puddu V., Berry R. J., Varshney V., Patwardhan S. V., Perry C. C., Heinz H. (2014). Force field and a surface
model database for silica to simulate interfacial properties in atomic
resolution. Chemistry of Materials.

[1064] Xiao S., Edwards S. A., Gräter F. (2011). A new transferable
forcefield for simulating the mechanics of CaCO_3_ crystals. J. Phys. Chem. C.

[1065] Kaminski G., Duffy E. M., Matsui T., Jorgensen W. L. (1994). Free energies
of hydration and pure liquid properties of hydrocarbons from the OPLS
all-atom model. The Journal of Physical Chemistry.

[1066] Cannon W. R., Pettitt B. M., McCammon J. A. (1994). Sulfate
anion in
water: model structural, thermodynamic, and dynamic properties. The Journal of Physical Chemistry.

[1067] Senanayake H. S., Wimalasiri P. N., Godahewa S. M., Thompson W. H., Greathouse J. A. (2023). Ab initio-derived
force field for amorphous silica
interfaces for use in molecular dynamics simulations. The Journal of Physical Chemistry C.

[1068] Van Beest B., Kramer G. J., Van Santen R. (1990). Force fields
for silicas and aluminophosphates based on ab initio calculations. Physical review letters.

[1069] Dauber-Osguthorpe P., Roberts V. A., Osguthorpe D. J., Wolff J., Genest M., Hagler A. T. (1988). Structure and energetics
of ligand binding to proteins: Escherichia coli dihydrofolate reductase-trimethoprim,
a drug-receptor system. Proteins: Structure,
Function, and Bioinformatics.

[1070] Pfeil L. B. (1926). The effect of occluded hydrogen on the tensile strength
of iron. Proceedings of the Royal Society of
London. Series A, Containing Papers of a Mathematical and Physical
Character.

[1071] Beachem C. (1972). A new model
for hydrogen-assisted cracking (hydrogen
”embrittlement”). Metallurgical
Transactions.

[1072] Nagumo M., Nakamura M., Takai K. (2001). Hydrogen thermal desorption
relevant to delayed-fracture susceptibility of high-strength steels. Metallurgical and Materials Transactions A.

[1073] Yang X., Oyeniyi W. O. (2017). Kinetic Monte Carlo
simulation of
hydrogen diffusion in tungsten. Fusion Engineering
and Design.

[1074] Zhu D., Oda T. (2016). Trap effect of vacancy on hydrogen diffusivity in bcc-Fe. Journal of Nuclear Materials.

[1075] Wang L.-F., Shu X., Lin D.-Y., Lu G.-H., Song H.-F. (2020). Molecular dynamics studies of hydrogen
diffusion in
tungsten at elevated temperature: Concentration dependence and defect
effects. International Journal of Hydrogen Energy.

[1076] Zhou X.-Y., Zhu J.-H., Wu H.-H. (2021). Molecular
dynamics
studies of the grain-size dependent hydrogen diffusion coefficient
of nanograined Fe. International Journal of
Hydrogen Energy.

[1077] Sagar S., Sluiter M. H., Dey P. (2024). First - Principles
study of hydrogen - Carbide interaction in bcc Fe. International Journal of Hydrogen Energy.

[1078] Zhang B., Xiong K., Wang M., Liu Z., Shen K., Mao Y., Chen H. (2024). Grain boundary alloying
segregation to resist hydrogen embrittlement in BCC-Fe steels: Atomistic
insights into solute-hydrogen interactions. Scripta Materialia.

[1079] Liu P.-Y., Zhang B., Niu R., Lu S.-L., Huang C., Wang M., Tian F., Mao Y., Li T., Burr P. A., Lu H., Guo A., Yen H.-W., Cairney J. M., Chen H., Chen Y.-S. (2024). Engineering metal-carbide
hydrogen traps in steels. Nature Communications.

[1080] Wang Y., Sharma B., Xu Y., Shimizu K., Fujihara H., Hirayama K., Takeuchi A., Uesugi M., Cheng G., Toda H. (2022). Switching nanoprecipitates
to resist
hydrogen embrittlement in high-strength aluminum alloys. Nature Communications.

[1081] Chen Y.-S., Huang C., Liu P.-Y., Yen H.-W., Niu R., Burr P., Moore K. L., Martínez-Pañeda E., Atrens A., Cairney J. M. (2025). Hydrogen trapping and embrittlement
in metals - A review. International Journal
of Hydrogen Energy.

[1082] Echeverri Restrepo S., Lambert H., Paxton A. T. (2020). Effect
of hydrogen
on vacancy diffusion. Phys. Rev. Mater..

[1083] Du J.-P., Geng W. T., Arakawa K., Li J., Ogata S. (2020). Hydrogen-Enhanced Vacancy Diffusion in Metals. The Journal of Physical Chemistry Letters.

[1084] Barnoush A., Vehoff H. (2010). Recent developments
in the study
of hydrogen embrittlement: Hydrogen effect on dislocation nucleation. Acta Materialia.

[1085] Wen M., Li Z., Barnoush A. (2013). Atomistic Study of
Hydrogen Effect
on Dislocation Nucleation at Crack Tip. Advanced
Engineering Materials.

[1086] Itakura M., Kaburaki H., Yamaguchi M., Okita T. (2013). The effect of hydrogen atoms on the screw dislocation mobility in
bcc iron: A first-principles study. Acta Materialia.

[1087] Song J., Curtin W. (2014). Mechanisms of hydrogen-enhanced
localized
plasticity: An atomistic study using *α*-Fe as
a model system. Acta Materialia.

[1088] Song J., Curtin W. A. (2013). Atomic mechanism
and prediction of
hydrogen embrittlement in iron. Nature Materials.

[1089] Tehranchi A., Zhou X., Curtin W. (2020). A decohesion
pathway
for hydrogen embrittlement in nickel: Mechanism and quantitative prediction. Acta Materialia.

[1090] Tehranchi A., Curtin W. A. (2017). Atomistic study of hydrogen embrittlement
of grain boundaries in nickel: II. Decohesion. Modelling and Simulation in Materials Science and Engineering.

[1091] Li J., Lu C., Pei L., Zhang C., Wang R. (2020). Atomistic
investigation of hydrogen induced decohesion of Ni grain boundaries. Mechanics of Materials.

[1092] Huang S., Chen D., Song J., McDowell D. L., Zhu T. (2017). Hydrogen embrittlement
of grain boundaries in nickel: an atomistic
study. Npj Comput. Mater..

[1093] Adlakha I., Solanki K. N. (2016). Critical assessment of hydrogen effects
on the slip transmission across grain boundaries in *α*-Fe. Proceedings of the Royal Society A: Mathematical,
Physical and Engineering Sciences.

[1094] Li J., Lu C., Pei L., Zhang C., Wang R. (2020). Hydrogen-modified
interaction between lattice dislocations and grain boundaries by atomistic
modelling. International Journal of Hydrogen
Energy.

[1095] Wan L., Geng W. T., Ishii A., Du J.-P., Mei Q., Ishikawa N., Kimizuka H., Ogata S. (2019). Hydrogen embrittlement
controlled by reaction of dislocation with grain boundary in alpha-iron. International Journal of Plasticity.

[1096] Zhou X.-Y., Yang X.-S., Zhu J.-H., Xing F. (2020). Atomistic
simulation study of the grain-size effect on hydrogen embrittlement
of nanograined Fe. International Journal of
Hydrogen Energy.

[1097] Kuhr B., Farkas D., Robertson I. M. (2016). Atomistic
studies of hydrogen effects on grain boundary structure and deformation
response in FCC Ni. Computational Materials
Science.

[1098] Ji Y., Shuang F., Ni Z., Yao C., Li X., Fu X., Chen Z., Li X., Dong C. (2024). Discerning the duality
of H in Mg: H-induced damage and ductility. International Journal of Plasticity.

[1099] Luo H., Lu W., Fang X., Ponge D., Li Z., Raabe D. (2018). Beating hydrogen with its own weapon: Nano-twin gradients
enhance
embrittlement resistance of a high-entropy alloy. Materials Today.

[1100] Luo H., Sohn S. S., Lu W., Li L., Li X., Soundararajan C. K., Krieger W., Li Z., Raabe D. (2020). A strong and
ductile medium-entropy alloy resists hydrogen embrittlement and corrosion. Nature Communications.

[1101] Li X., Yin J., Zhang J., Wang Y., Song X., Zhang Y., Ren X. (2022). Hydrogen embrittlement and failure
mechanisms of multi-principal element alloys: A review. Journal of Materials Science & Technology.

[1102] Ren X., Shi P., Zhang W., Wu X., Xu Q., Wang Y. (2019). Swamps of hydrogen in equiatomic
FeCuCrMnMo alloys: First-principles
calculations. Acta Materialia.

[1103] Xie Z., Wang Y., Lu C., Dai L. (2021). Sluggish hydrogen diffusion
and hydrogen decreasing stacking fault energy in a high-entropy alloy. Materials Today Communications.

[1104] Yin X., Liu X., Chen H., Chen S. (2023). Hydrogen segregation
by local chemical ordering structure in CrCoNi medium-entropy alloys:
A first principle study. Materials Today Communications.

[1105] Guo J., Xu S., Chen D. (2023). Elucidating
the hydrogen influence
on twin nucleation in FeNiCr medium-entropy alloy. Extreme Mechanics Letters.

[1106] Zhou X., Curtin W. A. (2020). First principles study of the effect
of hydrogen in austenitic stainless steels and high entropy alloys. Acta Materialia.

[1107] Zhou X., Tehranchi A., Curtin W. A. (2021). Mechanism and Prediction
of Hydrogen Embrittlement in fcc Stainless Steels and High Entropy
Alloys. Phys. Rev. Lett..

[1108] Tan A. M. Z., Li Z., Zhao Y., Ramamurty U., Gao H. (2024). Modeling the improved hydrogen embrittlement
tolerance of twin boundaries
in face-centered cubic complex concentrated alloys. Journal of the Mechanics and Physics of Solids.

[1109] Thompson A., Swiler L., Trott C., Foiles S., Tucker G. (2015). Spectral neighbor analysis method
for automated generation
of quantum-accurate interatomic potentials. Journal of Computational Physics.

[1110] Drautz R. (2019). Atomic cluster expansion for accurate
and transferable
interatomic potentials. Phys. Rev. B.

[1111] Sauceda H. E., Gálvez-González L. E., Chmiela S., Paz-Borbon L. O., Muller K.-R., Tkatchenko A. (2022). BIGDML-Towards
accurate quantum machine learning force fields for materials. Nature Communications.

[1112] Korostelev V., Wagner J., Klyukin K. (2023). Simple local environment
descriptors for accurate prediction of hydrogen absorption and migration
in metal alloys. J. Mater. Chem. A.

[1113] Tang H., Li B., Song Y., Liu M., Xu H., Wang G., Chung H., Li J. (2024). Reinforcement
Learning-Guided
Long-Timescale Simulation of Hydrogen Transport in Metals. Advanced Science.

[1114] Omrani S., Ghasemi M., Mahmoodpour S., Shafiei A., Rostami B. (2022). Insights from
molecular dynamics
on CO_2_ diffusion coefficient in saline water over a wide
range of temperatures, pressures, and salinity: CO_2_ geological
storage implications. J. Mol. Liq..

[1115] Zhang L., Lin D.-Y., Wang H., Car R., E W. (2019). Active learning of uniformly accurate interatomic potentials
for
materials simulation. Phys. Rev. Mater..

[1116] Kulichenko M., Barros K., Lubbers N., Li Y. W., Messerly R., Tretiak S., Smith J. S., Nebgen B. (2023). Uncertainty-driven
dynamics for active learning of interatomic potentials. Nature Computational Science.

[1117] Chen C., Ong S. P. (2022). A universal graph
deep learning interatomic
potential for the periodic table. Nature Computational
Science.

[1118] Deng B., Zhong P., Jun K., Riebesell J., Han K., Bartel C. J., Ceder G. (2023). CHGNet as a pretrained universal
neural network potential for charge-informed atomistic modelling. Nature Machine Intelligence.

[1119] Batatia, I. A foundation model for atomistic materials chemistry. arXiv 2024, 10.48550/arXiv.2401.00096 . 41230846

[1120] Neumann, M. ; Gin, J. ; Rhodes, B. ; Bennett, S. ; Li, Z. ; Choubisa, H. ; Hussey, A. ; Godwin, J. Orb: A Fast, Scalable Neural Network Potential. arXiv 2024, 10.48550/arXiv.2410.22570.

[1121] Rhodes, B. ; Vandenhaute, S. ; Simkus, V. ; Gin, J. ; Godwin, J. ; Duignan, T. ; Neumann, M. Orb-v3: atomistic simulation at scale. arXiv 2025, 10.48550/arXiv.2504.06231.

[1122] Yang, H. ; Hu, C. ; Zhou, Y. ; Liu, X. ; Shi, Y. ; Li, J. ; Li, G. ; Chen, Z. ; Chen, S. ; Zeni, C. ; Horton, M. ; Pinsler, R. ; Fowler, A. ; Zugner, D. ; Xie, T. ; Smith, J. ; Sun, L. ; Wang, Q. ; Kong, L. ; Liu, C. ; Hao, H. ; Lu, Z. MatterSim: A Deep Learning Atomistic Model Across Elements, Temperatures and Pressures. arXiv 2024, 10.48550/arXiv.2504.04967.

[1123] Bochkarev A., Lysogorskiy Y., Drautz R. (2024). Graph Atomic Cluster
Expansion for Semilocal Interactions beyond Equivariant Message Passing. Phys. Rev. X.

[1124] Park Y., Kim J., Hwang S., Han S. (2024). Scalable Parallel
Algorithm for Graph Neural Network Interatomic Potentials in Molecular
Dynamics Simulations. Journal of Chemical Theory
and Computation.

[1125] Zhang, D. ; Peng, A. ; Cai, C. ; Li, W. ; Zhou, Y. ; Zeng, J. ; Guo, M. ; Zhang, C. ; Li, B. ; Jiang, H. ; Zhu, T. ; Jia, W. ; Zhang, L. ; Wang, H. A Graph Neural Network for the Era of Large Atomistic Models. arXiv 2025, 10.48550/arXiv.2506.01686.

[1126] Barroso-Luque, L. ; Shuaibi, M. ; Fu, X. ; Wood, B. M. ; Dzamba, M. ; Gao, M. ; Rizvi, A. ; Zitnick, C. L. ; Ulissi, Z. W. Open Materials 2024 (OMat24) Inorganic Materials Dataset and Models. arXiv 2024, 10.48550/arXiv.2410.12771. 42230827

[1127] Fu, X. ; Wood, B. M. ; Barroso-Luque, L. ; Levine, D. S. ; Gao, M. ; Dzamba, M. ; Zitnick, C. L. Learning Smooth and Expressive Interatomic Potentials for Physical Property Prediction. arXiv 2025, 10.48550/arXiv.2502.12147.

[1128] Shuang F., Wei Z., Liu K., Gao W., Dey P. (2025). Universal machine learning interatomic potentials poised
to supplant
DFT in modeling general defects in metals and random alloys. Machine Learning: Science and Technology.

[1129] Schmidt J., Cerqueira T. F., Romero A. H., Loew A., Jäger F., Wang H.-C., Botti S., Marques M. A. (2024). Improving
machine-learning models in materials science through large datasets. Materials Today Physics.

[1130] Kaplan, A. D. ; Liu, R. ; Qi, J. ; Ko, T. W. ; Deng, B. ; Riebesell, J. ; Ceder, G. ; Persson, K. A. ; Ong, S. P. A Foundational Potential Energy Surface Dataset for Materials. arXiv 2025, 10.48550/arXiv.2503.04070.

[1131] Wines D., Choudhary K. (2025). CHIPS-FF: Evaluating Universal Machine
Learning Force Fields for Material Properties. ACS Materials Letters.

[1132] Zhang D., Liu X., Zhang X., Zhang C., Cai C., Bi H., Du Y., Qin X., Peng A., Huang J. (2024). DPA-2: a large atomic model as a
multi-task learner. npj Computational Materials.

[1133] Novikov I., Grabowski B., Körmann F., Shapeev A. (2022). Magnetic Moment Tensor Potentials
for Collinear Spin-Polarized
Materials Reproduce Different Magnetic States of Bcc Fe. npj Computational Materials.

[1134] Hellström M., Behler J. (2017). Structure of aqueous
NaOH solutions:
insights from neural-network-based molecular dynamics simulations. Physical Chemistry Chemical Physics.

[1135] Hellström M., Behler J. (2016). Concentration-Dependent
Proton Transfer
Mechanisms in Aqueous NaOH Solutions: From Acceptor-Driven to Donor-Driven
and Back. Journal of Physical Chemistry Letters.

[1136] Gomez A., Thompson W. H., Laage D. (2024). Neural-Network-Based
Molecular Dynamics Simulations Reveal That Proton Transport in Water
Is Doubly Gated by Sequential Hydrogen-Bond Exchange. Nature Chemistry.

[1137] Shao Y., Hellström M., Yllö A., Mindemark J., Hermansson K., Behler J., Zhang C. (2020). Temperature
Effects on the Ionic Conductivity in Concentrated Alkaline Electrolyte
Solutions. Physical Chemistry Chemical Physics.

[1138] Cheng B. (2025). Latent Ewald Summation for Machine
Learning of Long-Range Interactions. npj Computational
Materials.

[1139] Anstine D. M., Isayev O. (2023). Machine Learning Interatomic
Potentials
and Long-Range Physics. The Journal of Physical
Chemistry A.

[1140] Zhang L., Wang H., Muniz M. C., Panagiotopoulos A. Z., Car R., E W. (2022). A Deep Potential Model with Long-Range Electrostatic
Interactions. Journal of Chemical Physics.

[1141] Malosso C., Zhang L., Car R., Baroni S., Tisi D. (2022). Viscosity in Water from First-Principles
and Deep-Neural-Network
Simulations. npj Computational Materials.

[1142] Zhang L., Wang H., Car R., E W. (2021). Phase Diagram
of a Deep Potential Water Model. Physical Review
Letters.

[1143] Materials Studio; D.S. BIOVIA, Dassault Systèmes: San Diego. https://www.3ds.com/products/biovia/materials-studio, Accessed October 15, 2025.

[1144] Maps Platform, SCIENOMICS. https://www.scienomics.com/maps-platform/, Accessed October 15, 2025.

[1145] Theodorou D. N., Suter U. W. (1985). Detailed molecular structure of a
vinyl polymer glass. Macromolecules.

[1146] Theodorou D. N., Suter U. W. (1985). Shape of unperturbed
linear polymers:
polypropylene. Macromolecules.

[1147] Theodorou D. N., Suter U. W. (1985). Geometrical considerations
in model
systems with periodic boundaries. The Journal
of Chemical Physics.

[1148] Theodorou D. N., Suter U. W. (1986). Atomistic modeling of mechanical
properties of polymeric glasses. Macromolecules.

